# Establish and validate a clinical predictive model for pediatric gastrointestinal endoscopy

**DOI:** 10.3389/fmed.2026.1906112

**Published:** 2026-08-13

**Authors:** Bingyan Zhang, Jing Wu, Lifang Chen, Lianyu Zhou, Maojuan Li, Huixian Song, Rui Zhu, Hao Liang, Tingting Chen, Baihong Long, Fengrui Zhang, Junkun Niu

**Affiliations:** 1 Department of Gastroenterology, The First Affiliated Hospital of Kunming Medical University, Kunming, China; 2 Yunnan Province Clinical Research Center for Digestive Diseases, Kunming, China; 3 Department of Gastroenterology, The People's Hospital of Xishuangbanna Dai Nationality Autonomous Prefecture, Jinghong, Yunnan, China; 4 Qujing Central Hospital of Yunnan Province, Department of Gastroenterology, Qujing, China

**Keywords:** establishment, gastrointestinal endoscopy, pediatric, prediction model, validation

## Abstract

**Aim:**

Determining appropriate endoscopic referrals in children is challenging due to the lack of validated prediction tools. This study aimed to develop and validate a predictive model to support decision-making in pediatric endoscopy.

**Methods:**

This single-center retrospective study included hospitalized children aged 2–14 years who underwent first-time gastrointestinal endoscopy at the First Affiliated Hospital of Kunming Medical University between January 2009 and June 2023. Demographic characteristics, clinical presentations, laboratory results, imaging examinations, and endoscopic findings were collected. Patients were randomly assigned to training (70%) and validation (30%) cohorts. Candidate variables were selected using univariate analysis followed by the least absolute shrinkage and selection operator (LASSO) regression. A multivariate logistic regression model was then constructed. Model performance was evaluated using receiver operating characteristic (ROC) curve analysis, accuracy, and decision curve analysis (DCA).

**Results:**

A total of 1,425 children were included, of whom 64.9% had abnormal endoscopic findings. Three prediction models for gastrointestinal endoscopy (GIE), esophagogastroduodenoscopy (EGD), and colonoscopy achieved AUCs of 0.673, 0.686, and 0.701 in the training cohort, and 0.623–0.627 in the validation cohort. All models demonstrated modest predictive performance and clinical net benefit.

**Conclusions:**

We developed and internally validated three prediction models using routine clinical data to estimate the risk of positive endoscopic findings in children. These tools may support risk stratification and referral decisions, but prospective external validation is required before clinical use.

## Introduction

The incidence of pediatric gastrointestinal disorders has risen in recent years, influenced by environmental changes, dietary modifications, and increasing academic pressure. Gastrointestinal endoscopy (GIE) plays a pivotal role in the diagnosis and management of these conditions. Over the past four decades, innovations in endoscopic instrumentation and anesthesia have markedly advanced the field of pediatric endoscopy (1). A single-center study by Franciosi et al. documented a 12-fold increase in first-time pediatric gastroscopies between 1985 and 2005 (2). This upward trend likely reflects improved diagnostic capabilities rather than a true increase in incidence of disease. Nonetheless, the expanded use of endoscopy has enhanced diagnostic sensitivity and broadened the spectrum of identifiable gastrointestinal pathologies in children.

It is important to recognize that children are not merely “small adults”—they exhibit distinct physiological, anatomical and pathological characteristics. The pediatric gastrointestinal tract presents specific challenges, including narrower luminal diameters, thinner intestinal walls and reduced patient cooperation, all of which contribute to higher procedural risks compared with adult endoscopy. According to data from the Pediatric Clinical Outcomes Research Initiative, complication rates are 2.3% for esophagogastroduodenoscopy (EGD) and 1.1% for colonoscopy in children (3). Adverse events related to anesthesia and sedation are particularly common, accounting for approximately 60% of all complications, ranging from transient oxygen desaturation and aspiration to more severe outcomes such as respiratory arrest or shock (4–6). In support of this, a large-scale study of 9,577 procedures reported postoperative complications in 2.6% of cases, with procedure-related infections and perforations being the most frequent, typically manifesting as fever, abdominal or chest pain, or sore throat (5). Given the heightened vulnerability of pediatric patients, any adverse event can lead to substantial physical and psychological harm.

Most centers currently follow pediatric GIE guidelines from the American Society for Gastrointestinal Endoscopy (ASGE) and European Society for Pediatric Gastroenterology, Hepatology and Nutrition (ESPGHAN) and the European Society of Gastrointestinal Endoscopy (ESGE). However, the generalizability of these western protocols is limited by differences in healthcare systems and referral pathways, variations in disease prevalence and etiology, and disparities in resource availability, and existing studies have largely focused on indications and diagnostic yields rather than predictive modeling. While Mazigh et al. proposed a 4-parameter scoring system (including endoscopy within 24 h of bleeding, rebleeding, nonsteroidal anti-inflammatory drug use, and positive gastric lavage) that effectively predicted positive gastroscopic findings [area under the curve (AUC) = 0.837] in upper gastrointestinal bleeding (7), predictive models for other common pediatric gastrointestinal symptoms remain scarce. Despite its central role in diagnosis and management of gastrointestinal disorders, the inappropriate utilization of GIE is a widespread issue, particularly in developing countries (8). In addition, rising demand for pediatric endoscopy has substantially increased healthcare costs in many developed nations (9). Miele et al. found that nearly 25% of pediatric endoscopic procedures lacked clinical justification (10), underscoring the urgent need for stricter adherence to indication criteria. Furthermore, nonspecific symptoms, extraintestinal pathologies and psychological factors contribute to low endoscopic positivity rates. China, as a developing country with a large pediatric population, currently lacks nationally standardized guidelines for pediatric GIE, which may lead to the inappropriate use of endoscopy, resulting in unnecessary examinations that impose both physical inconvenience and financial burdens on patients and their families. Accurately determining endoscopic indications remains a major challenge for pediatricians, highlighting the need for ancillary tools to enhance diagnostic yield and avoid futile procedures. At present, no validated model exists to predict positive endoscopic findings in children. Thus, the establishment and validation of a reliable tool to guide pediatric endoscopy referral represents a pressing scientific and clinical priority.

To address this evidence gap, we developed and internally validated a comprehensive prediction model for positive endoscopic findings in children using routinely available clinical and laboratory data. The model integrates symptoms, physical signs, and laboratory parameters to estimate individual risk, with the goal of supporting risk stratification in pediatric GIE. It may serve as an adjunctive reference to help prioritize children at higher pretest probability. Future external validation and model refinement are needed before clinical implementation.

## Methods

### Patient population

**Figure 1:**
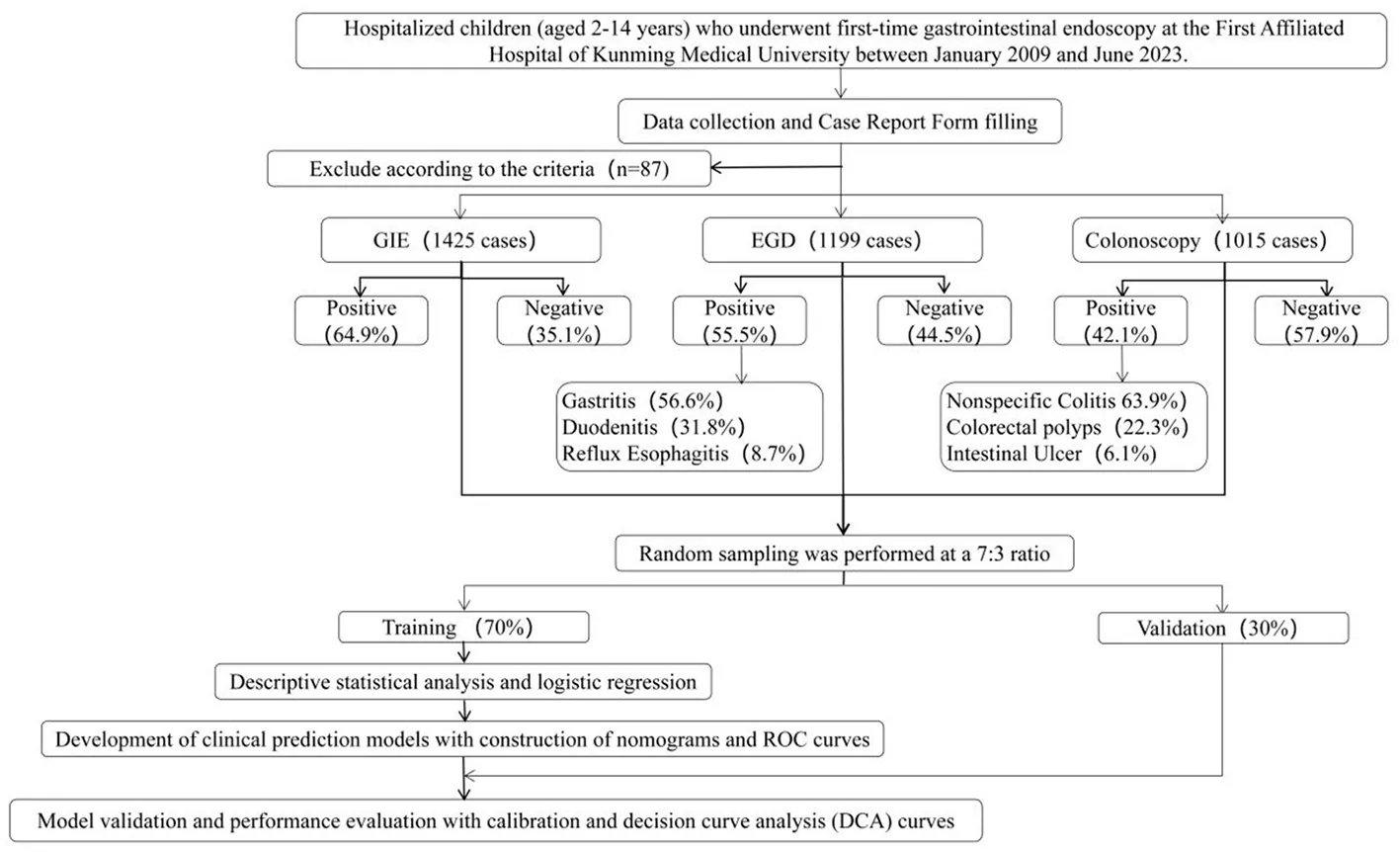
Flowchart of patient selection and experimental procedures.

This was a single-center retrospective study conducted at the First Affiliated Hospital of Kunming Medical University. Hospitalized children aged 2–14 years who underwent first-time gastrointestinal endoscopy between January 2009 and June 2023 were included. The following exclusion criteria were applied: (1) patients with missing or incomplete key statistical indicators; (2) those unable to cooperate with GIE, resulting in incomplete endoscopic procedures; (3) individuals younger than 2 years or older than 14 years; (4) patients undergoing repeat; and (5) non-hospitalized patients. Endoscopic positivity was defined as the presence of visually identifiable abnormalities during endoscopy and/or relevant histopathological findings. Endoscopic abnormalities included, but were not limited to, esophagitis, gastritis, duodenitis and peptic ulcer. Histopathological anomalies were primarily defined as moderate to severe inflammation or Helicobacter pylori infection, confirmed via immunohistochemical staining, or any abnormality necessitating active intervention, scheduled surveillance, or a change in clinical management (11). Conversely, negative findings included mild non-specific gastritis or duodenitis in the absence of H. pylori infection or erosive lesions, as well as minor alterations that did not influence patient management. The study protocol was approved by the Institutional Review Board of the first affiliated hospital of kunming medical university (Approval No. 2022-L-181). The patient selection process is summarized in Figure 1.

### Data collection

A case collection form (see Appendix 1) was designed to collect data from children aged 2–14 years who underwent GIE for the first time in the First Affiliated Hospital of Kunming Medical University from January 2009 to June 2023. A total of 108 variables were collected for each patient using standardized Case Report Forms (CRFs), including information on demographic and anthropometric data (age, sex, weight, height, etc.), clinical symptoms and signs (abdominal pain, hematochezia, melena, diarrhea, vomiting, and other gastrointestinal manifestations), laboratory parameters (complete blood count, biochemical markers, inflammatory markers, urinalysis, and stool examinations, etc., imaging findings (abdominal ultrasound, computed tomography (CT), and other relevant radiological examinations), medical history, and medication [comorbidities, previous surgeries, and long-term medication use (defined as regular use of any medication for ≥3 months)].

### Statistical analysis

All statistical analyses were performed using SPSS 26.0 (Statistical Package for the Social Sciences 26 for Windows) and R software (version 4.2; R Foundation). Patients with missing outcome or key demographic data were excluded. Variables with >50% missingness (iron, ferritin, transferrin) were excluded. For categorical variables, missingness was treated as a separate category. For remaining variables (< 20% missing), multiple imputation was performed, which is the recommended method for handling missing data in clinical research as it preserves statistical power, reduces bias, and properly accounts for uncertainty (12). Each variable with missingness was imputed using all other variables in the analysis model as predictors, including the outcome. Linear regression (or predictive mean matching) for continuous variables, logistic regression for binary variables, and multinomial/ordinal logistic regression for categorical variables. Patients were randomly allocated to a training cohort (70%) or a validation cohort (30%) using computer-generated random numbers (seed = 2,023,062,912). Random splitting was chosen over temporal validation as the long study period lacked a clinically meaningful time-based cut-off, and random allocation ensured representativeness of the full period while preserving statistical power. This approach is consistent with the Transparent Reporting of a Multivariable Prediction Model for Individual Prognosis or Diagnosis (TRIPOD) recommendations for internal validation.

Categorical variables are presented as percentages and compared using the chi-square test. Normally distributed continuous variables are expressed as mean ± standard (Mean ± SD) and compared with the Student's *t*-test (equal variances) or Welch's *t*-test (unequal variances), respectively. A two-sided *P* value of < 0.05 was considered statistically significant.

In the training cohort, variables showing a significance level of *P* < 0.1 in univariate analyses were incorporated into a LASSO regression model for feature selection. To prevent overfitting and minimize the influence of multicollinearity, we performed LASSO regression with 10-fold cross-validation. The optimal penalty parameter (λ) was determined using the 1-standard error (1-SE) rule, yielding a parsimonious set of the most robust predictors for the subsequent multivariable analysis. The selected variables were then used to build a multivariate logistic regression model, which was presented as a nomogram. The model's performance was evaluated by calibration (using the Hosmer-Lemeshow test and calibration curves), discriminative ability (using the receiver operating characteristic curve), and clinical utility (using decision curve analysis) in both cohorts.

## Result

### Demographic statistics

A total of 2,308 GIEs were performed on 1,512 children aged 2–14 years. After excluding 87 cases (53 due to repeated endoscopies, 2 for incomplete examinations, and 32 for incomplete data), 1,425 patients were ultimately included in the study. The mean age was 10.6 years (range: 2–14 years), and 58.4% of them were male. Demographic characteristics are presented in Table 1. A total of 1,199 EGD procedures and 1,015 colonoscopy procedures were performed. Of these, and 789 patients underwent both procedures during the same endoscopic session. Accordingly, 410 patients underwent EGD alone, and 226 patients underwent colonoscopy alone. Among these, 925 children (64.9%) had positive findings on GIE, the positive rates of EGD and colonoscopy are respectively 666 (55.5%) and 427 (42.1%). Ultimately, the top five positive findings in EGD were gastritis (56.6%), duodenitis (31.8%), reflux esophagitis (8.7%), peptic ulcer (7.1%), and anaphylactoid purpura (3.5%) (Table 2). The leading positive findings in colonoscopy included nonspecific enteritis (63.9%), colorectal polyps (22.3%), intestinal ulcers (6.1%), and inflammatory bowel disease (IBD), accounting for 3.8% (Table 3).

**Table 1:** Baseline patient characteristics.

Characteristics	*n* (%)
Age	
2–3 y	24 (1.7)
3–12 y	991 (69.5)
12–14 y	410 (28.8)
Sex	
Male	832 (58.4)
Female	593 (41.6)
Types of endoscopes	
GIE	789 (55.4)
EGD	1,199 (84.1)
Colonoscopy	1,015 (71.2)
Endoscopic result	
Positive GIE	925 (64.9)
Positive EGD	666 (55.5)
Positive colonoscopy	427 (42.1)

**Table 2:** The positive diagnosis of EGD.

Variable	Cases (*n*)	Ratio (%)
Gastritis	377	56.6
Duodenitis	212	31.8
Reflux esophagitis	58	8.7
Peptic ulcer	47	7.1
Anaphylactoid purpura	23	3.5
Polyp	16	2.4
Esophageal/fundus varices	15	2.3
Achalasia	14	2.1
Mycotic esophagitis	12	1.8
Non-specific esophagitis	6	0.9
Eosinophilic esophagitis	3	0.5
Barrett's esophagus	3	0.5
Other	82	12.3

**Table 3:** The positive diagnosis of colonoscopy.

Variable	Cases (*n*)	Ratio (%)
Nonspecific colitis	272	63.9
Colorectal polyps	95	22.3
Intestinal ulcer	26	6.1
IBD	16	3.8
Infective enteritis	8	1.9
Eosinophilic colitis	1	0.2
Anaphylactoid purpura	1	0.2
Other	48	11.3

### Statistical analysis and model development

The basic characteristics of the training cohort population are presented in Table 4. The collected demographic information (sex, age, height, weight, and ethnicity) indicated a predominance of males and individuals of the Han ethnicity, with age categorized into 2–3, 3–12, and 12–14-year groups. The training cohort for the GIE group comprised 998 cases, including 648 positive and 350 negative cases, a separate validation cohort included 427 cases. In the EGD group, the training cohort contained 837 patients, with 466 positive and 371 negative cases. The validation cohort consisted of 362 cases. For the colonoscopy group, the training cohort enrolled 708 cases, of which 298 were positive and 410 were negative, and the validation cohort comprised 307 cases.

**Table 4:** Baseline characteristics of patients in the training cohort stratified by endoscopy type.

Variable	GIE group			EGD group			Colonoscopy group		
	Total (*N* = 998)	Negative (*N* = 350)	Positive (*N* = 648)	Total (*N* = 837)	Negative (*N* = 371)	Positive (*N* = 466)	Total (*N* = 708)	Negative (*N* = 410)	Positive (*N* = 298)
Sex, *n* (%)									
Female	408 (41.0)	150 (43.0)	258 (40.0)	352 (42.0)	167 (45.0)	185 (40.0)	300 (42.0)	181 (44.0)	119 (40.0)
Male	590 (59.0)	200 (57.0)	390 (60.0)	485 (58.0)	204 (55.0)	281 (60.0)	408 (58.0)	229 (56.0)	179 (60.0)
Age, *n* (%)									
2–3 years	13 (1.3)	3 (0.9)	10 (1.6)	9 (1.1)	5 (1.3)	4 (0.9)	13 (1.8)	4 (1.0)	9 (3.0)
3–12 years	690 (69.1)	270 (77.1)	420 (64.8)	586 (70.0)	287 (77.4)	299 (64.1)	505 (71.3)	299 (72.9)	206 (69.1)
12–14 years	295 (29.6)	77 (22.0)	218 (33.6)	242 (28.9)	79 (21.3)	163 (35.0)	190 (26.9)	107 (26.1)	83 (27.9)
Nation, *n* (%)									
Minority	213 (21.0)	76 (22.0)	137 (21.0)	209 (25.0)	89 (24.0)	120 (26.0)	183 (26.0)	117 (29.0)	66 (22.0)
Han	785 (79.0)	27 (78.0)	511 (79.0)	628 (75.0)	282 (76.0)	346 (74.0)	525 (74.0)	293 (71.0)	232 (78.0)
Anthropometrics									
Weight, kg (mean ± SD)	37 ± 15	35 ± 13	38 ± 15	37 ± 15	36 ± 14	38 ± 15	37 ± 15	37 ± 14	38 ± 17
Height, cm (mean ± SD)	143 ± 17	141 ± 17	145 ± 18	144 ± 16	141 ± 16	145 ± 16	143 ± 18	143 ± 16	143 ± 19
BMI, Kg/m^2^ (mean ± SD)	17.4 ± 4.3	17.1 ± 3.6	17.6 ± 4.5	17.4 ± 4.3	17.4 ± 4.1	17.5 ± 4.5	17.5 ± 4.5	17.4 ± 4.2	17.8 ± 4.8

Data presented as *n* (%) for categorical variables and Mean ± SD for continuous variables.

### Univariate analysis for positive endoscopic findings

Results of the univariate analysis for positive endoscopic findings are presented separately in Tables 5a–c for GIE, EGD, and colonoscopy. The related factors with a *P*-value of < 0.1 in univariate analysis were included in the LASSO regression analyses. Significant predictors in the GIE cohort were identified, encompassing anthropometric measures, vital signs, gastrointestinal symptoms (e.g., melena, hematochezia), inflammatory markers (e.g., neutrophils, hs-CRP), laboratory markers (e.g., ALB, serum calcium), and stool abnormalities. The EGD cohort was characterized by predictors indicative of upper GI-related symptoms and signs, specifically vomiting, hematemesis, and melena. In contrast, the colonoscopy cohort was defined by a strong association with hematochezia, aminosalicylate use, and positive stool findings. These findings informed subsequent feature selection for predictive modeling.

**Table 5a:** Univariate analysis of the GIE.

Variable	Negative (*N* = 350)	Positive (*N* = 648)	*P*
Anthropometric			
Weight, kg	35 ± 13	38 ± 15	0.003
Height, cm	141 ± 17	145 ± 18	0.003
BMI, kg/m^2^	17.1 ± 3.6	17.6 ± 4.5	0.087
Symptoms and signs, *n* (%)			
Abdominal pain	296 (84.6)	503 (77.6)	0.009
Melena	11 (3.1)	40 (6.2)	0.042
Hematochezia	17 (4.9)	70 (10.8)	0.002
Abdominal tenderness	272 (77.7)	463 (71.5)	0.032
Pale appearance	9 (2.6)	40 (6.2)	0.015
Symptoms of anemia	7 (2.0)	33 (5.1)	0.022
History, *n* (%)			
Respiratory history	26 (7.4)	29 (4.5)	0.053
Long-term medication history	6 (1.7)	28 (4.3)	0.036
Laboratory markers			
White blood cell (WBC), × 10^9^/L	5.94 (5.12, 7.39)	6.24 (4.92, 7.82)	0.039
Neutrophil percentage, %	46 (40, 56)	51 (42, 60)	< 0.001
Lymphocyte percentage, %	43 (35, 50)	39 (30, 47)	< 0.001
Monocyte, × 10^9^/L	0.38 (0.29, 0.49)	0.40 (0.29, 0.52)	0.056
Erythrocyte, × 10^1^^2^/L	4.74 ± 0.52	4.65 ± 0.67	0.032
Hemoglobin, g/L	134 ± 15	131 ± 20	0.041
Albumin (ALB), g/L	42.8 ± 4.1	41.8 ± 5.3	0.003
Globulin (GLB), g/L	26.2 ± 4.2	27.1 ± 4.9	0.004
γ-glutamyl transferase (γ-GGT), U/L	12 (10,15)	13 (10-17)	0.075
Alkaline phosphatase, U/L	200 (159, 254)	186 (130, 239)	0.004
High-sensitivity CRP (hsCRP), mg/L	0.50 (0.50, 0.75)	0.50 (0.50, 1.50)	0.003
Sodium ion, mmol/L	141.8 ± 3.0	141.4 ± 3.1	0.045
Chloride ion, mmol/L	105.9 ± 2.8	105.2 ± 3.5	0.006
Serum calcium, mmol/L	2.42 ± 0.11	2.38 ± 0.14	< 0.001
Serum iron, μmol/L	14 (11-19)	13 (9-18)	0.032
Stool tests, *n* (%)			
Abnormal routine stool	34 (9.7)	117 (18.1)	< 0.001
Occult blood in stool	34 (9.7)	114 (17.6)	< 0.001

^1^Data are presented as *n* (%) for categorical variables; Median (Q25, Q75) for non-normally distributed continuous variables; Mean ± SD for normally distributed continuous variables. ^2^Statistical comparisons were performed using: Pearson's Chi-squared test for categorical variables; Wilcoxon rank sum test (Mann-Whitney U) for non-normally distributed continuous variables; Independent *t*-test for normally distributed continuous variables.

**Table 5b:** Univariate analysis of the EGD.

Variable	Negative (*N* = 371)	Positive (*N* = 466)	*P*
Anthropometric			
Weight, kg	36 ± 14	38 ± 15	0.016
Height, cm	141 ± 16	145 ± 16	< 0.001
Symptoms and signs, *n* (%)			
Abdominal pain	333 (89.8)	354 (76.0)	< 0.001
Vomiting	95 (25.6)	158 (33.9)	0.010
Diarrhea	27 (7.3)	21 (4.5)	0.090
Haematemesis	8 (2.2)	28 (6.0)	0.009
Melena	11 (3.0)	35 (7.5)	0.006
Skin manifestation	13 (3.5)	29 (6.2)	0.077
Oral ulcer/rash/abdominal mass	7 (1.9)	5 (1.1)	0.003
Abdominal tenderness	299 (80.6)	336 (72.1)	0.005
Pale appearance	13 (3.5)	29 (6.2)	0.077
History, *n* (%)			
Respiratory history	33 (8.9)	20 (4.3)	0.008
Laboratory markers			
White blood cell (WBC), × 10^9^/L	6.01 (5.01, 7.60)	6.30 (4.82, 7.86)	0.086
Neutrophil percentage, %	48 (41, 57)	52 (42, 61)	0.003
Lymphocyte percentage, %	41 (34, 49)	38 (29, 47)	0.001
Albumin (ALB), g/L	42.7 ± 4.1	41.7 ± 5.6	0.003
γ-glutamyl transferase (γ-GGT), U/L	12 (10-15)	13 (10-17)	0.060
Alkaline phosphatase, U/L	204 (165, 252)	185 (130, 238)	< 0.001
High-sensitivity CRP (hsCRP), mg/L	0.50 (0.50, 0.90)	0.500 (0.50, 1.39)	0.014
Sodium ion, mmol/L	141.9 ± 2.9	141.4 ± 3.3	0.015
Chloride ion, mmol/L	105.80 ± 2.99	105.16 ± 3.52	0.007
Serum calcium, mmol/L	2.41 ± 0.11	2.37 ± 0.16	< 0.001
Positive WBC in urine	43 (11.6)	34 (7.3)	0.035
Stool tests, *n* (%)			
Abnormal routine stool	45 (12.1)	75 (16.1)	0.070
Occult blood in stool	43 (11.6)	72 (15.5)	0.074

^1^Data are presented as *n* (%) for categorical variables; Median (Q25, Q75) for non-normally distributed continuous variables; Mean ± SD for normally distributed continuous variables. ^2^Statistical comparisons were performed using: Pearson's Chi-squared test for categorical variables; Wilcoxon rank sum test (Mann-Whitney U) for non-normally distributed continuous variables; Independent *t*-test for normally distributed continuous variables.

**Table 5c:** Univariate analysis of the colonoscopy.

Variable	Negative (*N* = 410)	Positive (*N* = 298)	*P*
Symptoms and signs			
Temperature, °C	36.55 ± 0.27	36.60 ± 0.39	0.037
Abdominal pain	379 (92.4)	235 (78.9)	< 0.001
Nausea	134 (32.7)	62 (20.8)	< 0.001
Abdominal distension	43 (10.5)	20 (6.7)	0.084
Hematochezia	20 (4.9)	56 (18.8)	< 0.001
Abdominal tenderness	352 (85.9)	212 (71.1)	< 0.001
History			
Long-term medication history	11 (2.7)	16 (5.4)	0.071
Aminosalicylic acid history	1 (0.2)	7 (2.3)	0.033
Laboratory markers			
Neutrophil percentage, %	46 (39, 55)	49 (42, 57)	0.013
Lymphocyte percentage, %	44 (35, 50)	40 (33, 48)	0.007
Hemoglobin, g/L	135 ± 16	132 ± 20	0.030
MCV, fL	85.0 ± 5.4	84.2 ± 5.9	0.060
MCH, pg	28.62 ± 3.19	28.02 ± 2.31	0.007
MCHC, g/L	334 ± 15	332 ± 14	0.044
Albumin (ALB), g/L	43.2 ± 3.8	42.1 ± 4.8	< 0.001
Globulin (GLB), g/L	26.2 ± 3.7	27.7 ± 5.1	< 0.001
Alanine aminotransferase (ALT), U/L	12 (9-16)	12 (9-17)	0.002
Aspartate aminotransferase (AST), U/L	20 (16-24)	21 (17-25)	0.011
High-sensitivity CRP (hsCRP), mg/L	0.50 (0.50, 0.70)	0.500 (0.50, 1.69)	0.007
Blood urea nitrogen (BUN), mmol/L	4.24 (3.50, 5.15)	3.85 (3.23, 4.65)	0.001
Sodium ion, mmol/L	142.22 ± 2.78	141.84 ± 2.59	0.071
Chloride ion, mmol/L	106.03 ± 2.87	105.48 ± 3.05	0.017
Serum iron, μmol/L	14 (11-19)	12 (8-17)	0.005
Total iron binding capacity, μmol/L	51 (46, 56)	49 (43, 55)	< 0.001
Transferrin, g/L	2.47 ± 0.35	2.34 ± 0.48	0.003
Stool tests			
Abnormal routine stool	41 (10.0)	74 (24.8)	< 0.001
Occult blood in stool	38 (9.3)	71 (23.8)	< 0.001

^1^Data are presented as *n* (%) for categorical variables; Median (Q25, Q75) for non-normally distributed continuous variables; Mean ± SD for normally distributed continuous variables. ^2^Statistical comparisons were performed using: Pearson's Chi-squared test for categorical variables; Wilcoxon rank sum test (Mann-Whitney U) for non-normally distributed continuous variables; Independent *t*-test for normally distributed continuous variables. MCV, mean corpuscular volume; MCH, mean corpuscular hemoglobin; MCHC, mean corpuscular hemoglobin concentration

### LASSO regression screening variables and model construction

**Figure 2:**
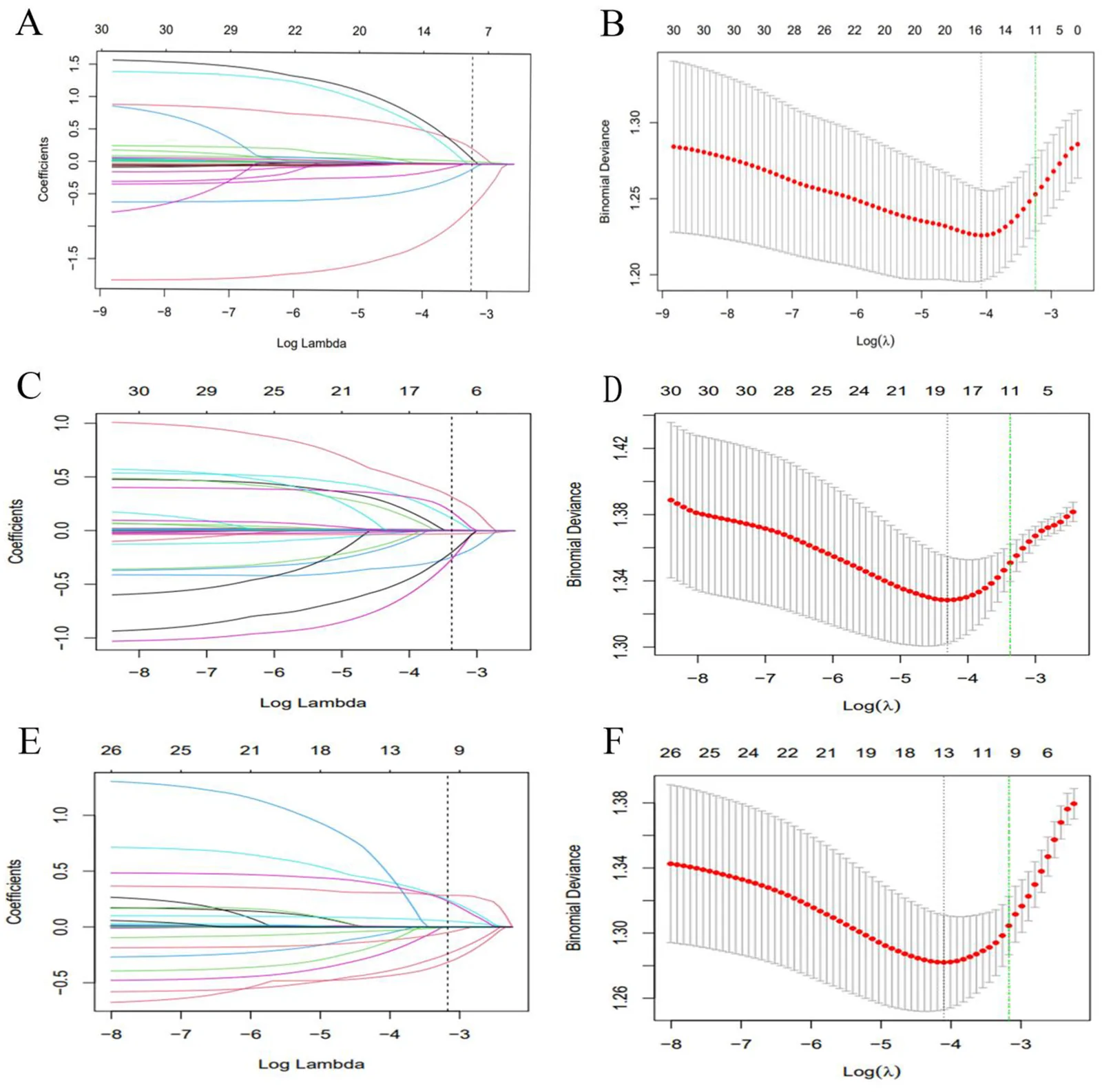
LASSO regression with 10-fold cross-validation for feature selection in the GIE, EGD, and colonoscopy groups. The optimal λ was determined using the 1-SE rule (one standard error above the minimum cross-validated error) to balance model complexity and predictive performance. Panels A, C, and E show the cross-validation error curves for the GIE, EGD, and colonoscopy cohorts, respectively, with the optimal λ indicated by dotted vertical lines. Panels B, D, and F display the corresponding LASSO coefficient profiles, showing how each variable's coefficient changes with log (λ); vertical dotted lines mark the optimal λ values. Variables with non-zero coefficients at the optimal λ were selected for model development. GIE group (**A, B;** λ = 0.03903): 11 variables were retained. EGD group (**C, D;** λ = 0.03423): 11 variables were retained. Colonoscopy group (**E, F**; λ = 0.04196): 9 variables were retained.

Serum iron, ferritin, and transferrin were precluded from further consideration prior to formal modeling, on account of significant inter-group differences on univariate screening due to excessive missingness. Subsequently, LASSO regression was employed to mitigate overfitting, reduce multicollinearity, and select the most informative subset of variables for the final predictive model. The variables were fitted with multiple logistic regression models (Figure 2).

Following regularization via LASSO, a subset of 11 variables was retained for the GIE group (λ = 0.03903), comprising weight, height, hematochezia, respiratory disease history, long-term medication use, neutrophil percentage, lymphocyte percentage, albumin, globulin, hs-CRP, and serum calcium (Figures 2A, B). The EGD group (λ = 0.03423) retained 11 features: height, abdominal pain, vomiting, diarrhea, melena, oral ulcer/rash/abdominal mass, ALB, γ- GGT, hs-CRP, serum calcium, and alkaline phosphatase (Figures 2C, D). For the colonoscopy group (λ = 0.04196), 9 variables were incorporated: abdominal pain, hematochezia, abdominal tenderness, MCH, globulin, ALT, BUN, abnormal stool routine, and fecal occult blood. These selected variables were used to develop predictive model in their respective cohorts (Figures 2E, F).

Multivariable logistic regression was performed using the LASSO-selected variables to identify independent predictors of positive endoscopic findings for each group. The regression coefficients, adjusted odds ratios (ORs) with 95% confidence intervals (CIs), and *P* values are summarized in Tables 6–8.

**Table 6:** Multivariable logistic regression analysis of the GIE.

Variables	B	S.E.	OR	95% CI	*P*
Weight	0.0019	0.009	1.002	0.984, 1.021	0.8
Height	0.0112	0.008	1.011	0.996, 1.027	0.15
Hematochezia	0.8906	0.347	2.436	1.273, 5.016	0.010
Respiratory history	−0.5636	0.329	0.569	0.297, 1.089	0.087
Long-term medication history	1.5987	0.637	4.947	1.632, 21.583	0.012
Neutrophil percentage	0.0146	0.021	1.015	0.972, 1.058	0.5
Lymphocyte percentage	−0.0004	0.023	1.000	0.955, 1.045	>0.9
Albumin (ALB)	0.0284	0.023	1.029	0.983, 1.076	0.2
Globulin (GLB)	0.0536	0.021	1.055	1.013, 1.101	0.012
High-sensitivity CRP (hsCRP)	0.0152	0.008	1.015	1.002, 1.036	0.069
Serum calcium	−2.3494	0.803	0.095	0.019, 0.448	0.003
Constant	1.1576	2.755	3.182	0.015, 766.496	0.7

CI, confidence interval; OR, odds ratio; S.E., standard error.

Multivariable logistic regression was performed using the 11 variables selected by LASSO for the GIE group. As shown in Table 6, hematochezia, long-term medication use and globulin were identified as independent positive predictors of positive endoscopic findings. Conversely, serum calcium was a protective factor. The remaining LASSO-selected variables (weight, height, respiratory disease history, neutrophil percentage, lymphocyte percentage, albumin, and hs-CRP) did not reach statistical significance (all *P* > 0.05) but were retained in the model to optimize overall predictive performance, consistent with the principle of maintaining model parsimony while maximizing discriminative ability.

For the EGD group (Table 7), height (OR = 1.017, 95% CI: 1.006–1.028, *P* = 0.002) and vomiting (OR = 1.870, 95% CI: 1.278–2.756, *P* = 0.001) were identified as independent predictors of positive endoscopic findings. Notably, abdominal pain (OR = 0.558, 95% CI: 0.336–0.910, *P* = 0.021), diarrhea (OR = 0.379, 95% CI: 0.180–0.769, *P* = 0.008), and ALP (OR = 0.997, 95% CI: 0.995–1.000, *P* = 0.037) were protective factors. Other LASSO-selected variables, including melena, oral ulcer/rash/abdominal mass, ALB, γ-GGT, hs-CRP, and serum calcium, did not reach statistical significance (all *P* > 0.05) but were retained in the model for optimal predictive performance.

**Table 7:** Multivariable logistic regression analysis of the EGD.

Variables	B	S.E.	OR	95%CI	*P*
Height	0.0168	0.005	1.017	1.006, 1.028	0.002
Abdominal pain	−0.5836	0.253	0.558	0.336, 0.910	0.021
Vomiting	0.6258	0.196	1.870	1.278, 2.756	0.001
Diarrhea	−0.9711	0.368	0.379	0.180, 0.769	0.008
Melena	0.8556	0.529	2.353	0.895, 7.412	0.11
Oral ulcer/rash/abdominal mass	0.7040	0.408	2.022	0.930, 4.686	0.085
Albumin (ALB)	−0.0168	0.027	0.983	0.932, 1.037	0.5
γ-glutamyl transferase (γ-GGT)	0.0093	0.005	1.009	1.002, 1.021	0.052
High-sensitivity CRP (hsCRP)	0.0077	0.008	1.008	0.994, 1.025	0.3
Serum calcium	−1.0123	0.859	0.363	0.066, 1.914	0.2
Alkaline phosphatase	−0.0026	0.001	0.997	0.995, 1.000	0.037
Constant	1.5922	1.931	4.914	0.120, 233.147	0.4

CI, confidence interval; OR, odds ratio; S.E., standard error.

For the colonoscopy group (Table 8), hematochezia (OR = 2.622, 95% CI: 1.386–5.074, *P* = 0.003), globulin (OR = 1.084, 95% CI: 1.041–1.131, *P* < 0.001), and ALT (OR = 1.020, 95% CI: 1.006–1.036, *P* = 0.007) were identified as independent positive predictors of positive endoscopic findings. Conversely, abdominal tenderness (OR = 0.597, 95% CI: 0.369–0.968, *P* = 0.036) and BUN (OR = 0.810, 95% CI: 0.705–0.927, *P* = 0.003) were protective factors. The remaining LASSO-selected variables (abdominal pain, MCH, abnormal routine stool, and occult blood in stool) did not reach statistical significance (all *P* > 0.05) but were retained in the model for optimal predictive performance.

**Table 8:** Multivariable logistic regression analysis of the colonoscopy.

Variables	B	S.E.	OR	95% CI	*P*
Abdominal pain	−0.4670	0.3090	0.627	0.341, 1.150	0.13
Hematochezia	0.9640	0.3295	2.622	1.386, 5.074	0.003
Abdominal tenderness	−0.5151	0.2453	0.597	0.369, 0.968	0.036
MCH	−0.0345	0.0355	0.966	0.898, 1.030	0.3
Globulin (GLB)	0.0810	0.0210	1.084	1.041, 1.131	< 0.001
Alanine aminotransferase (ALT)	0.0199	0.0074	1.020	1.006, 1.036	0.007
Blood urea nitrogen (BUN)	−0.2111	0.0699	0.810	0.705, 0.927	0.003
Abnormal routine stool	0.6383	0.7279	1.893	0.432, 8.315	0.4
Occult blood in stool	0.1873	0.7500	1.206	0.264, 5.490	0.8
Constant	−0.3469	1.2826	0.707	0.063, 9.326	0.8

CI, confidence interval; OR, odds ratio; S.E., standard error.

**Figure 3:**
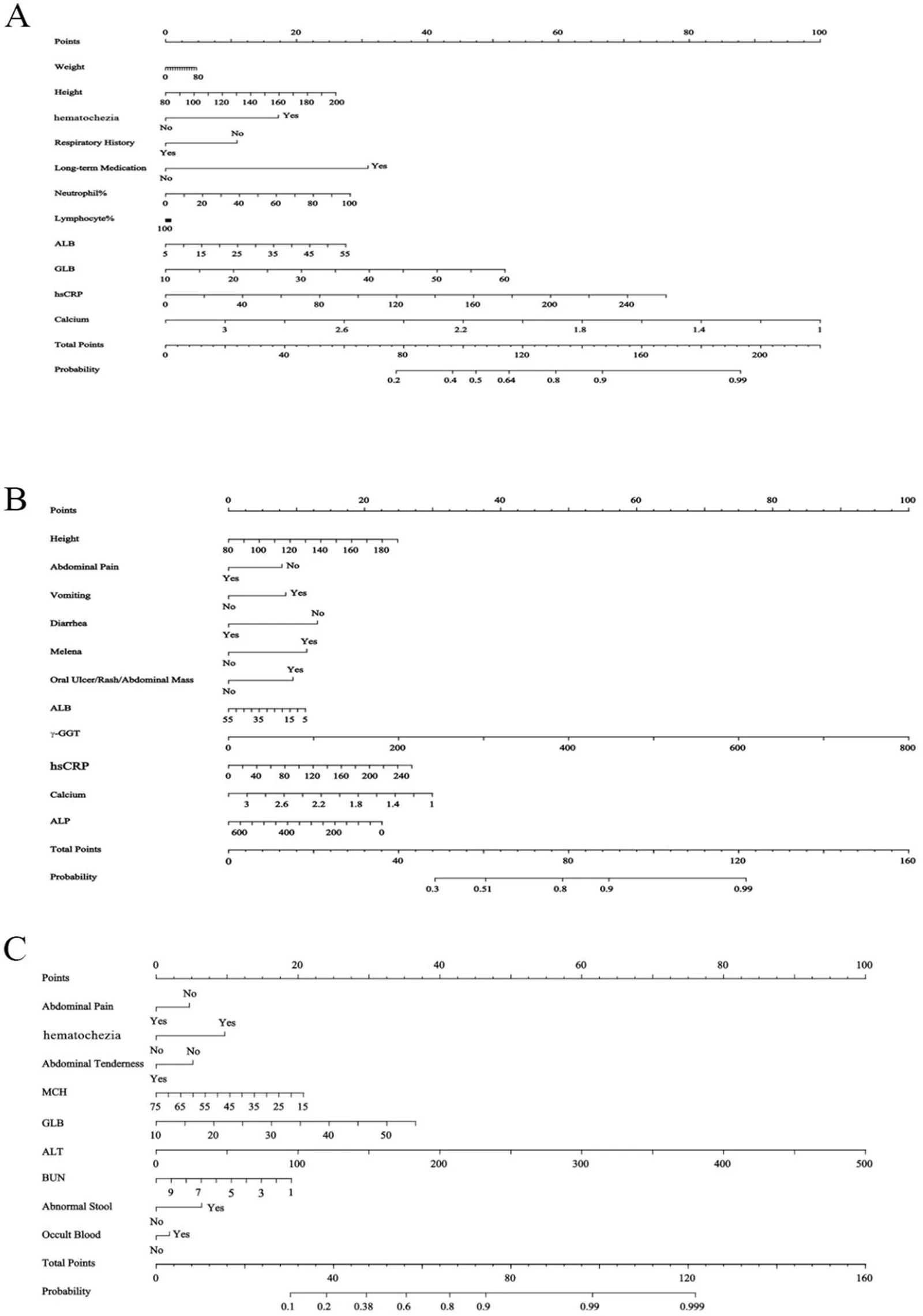
The models were visualized via column-line plots (nomograms) to facilitate intuitive risk stratification through an integrated scoring system. Each predictor variable is assigned a quantified score corresponding to its value, represented by vertical lines on the top scale of the nomogram. The aggregate score derived from all variables was subsequently projected onto the bottom prediction line to estimate the corresponding probability of a positive endoscopic finding.

In the GIE group, the linear predictor was 1.1576 + 0.0019 × weight + 0.0112 × height + 0.8906 × hematochezia + (−0.5636) × respiratory history + 1.5987 × long-term medication + 0.0146 × neutrophil% + (−0.0004) × lymphocyte% + 0.0284 × ALB + 0.0536 × GLB + 0.0152 × hsCRP + (−2.3494) × serum calcium. In order to facilitate clinical practice, a nomogram was constructed using the regression modeling strategies package prediction model (Figure 3A). A nomogram score of 104 (out of a maximum 220), corresponding to a 50% endoscopic positivity represents the threshold for recommending endoscopic evaluation. In the EGD group, the linear predictor was 1.5922 + 0.0168 × height + (−0.5836) × abdominal pain + 0.6258 × vomiting + (−0.9711) × diarrhea + 0.8556 × melena + 0.7040 × oral ulcer/rash/abdominal mass + (−0.0168) × ALB + (−0.0093) × γ- GGT + 0.0077 × hsCRP + (−1.0123) × serum calcium + (−0.0026) × alkaline phosphatase. As the nomogram shown (Figure 3B), the total score of the nomogram was 160 points. The nomogram associates a score of 60 with a 51% probability of positive EGD findings. Thus, a score greater than 60 serves as an indication for the procedure. In the colonoscopy group, the linear predictor was −0.3469 + (−0.4670) × abdominal pain + 0.9640 × hematochezia + (−0.5151) × abdominal tenderness + (−0.0345) × MCH + 0.0810 × GLB + 0.0199 × ALT + (−0.2111) × BUN + 0.6383 × abnormal stool + 0.1873 × occult blood. The nomogram was shown in Figure 3C. When the total score reached 56, the predicted probability of a positive colonoscopy finding was 0.60.

### Verify and evaluate the logistic regression model

**Figure 4:**
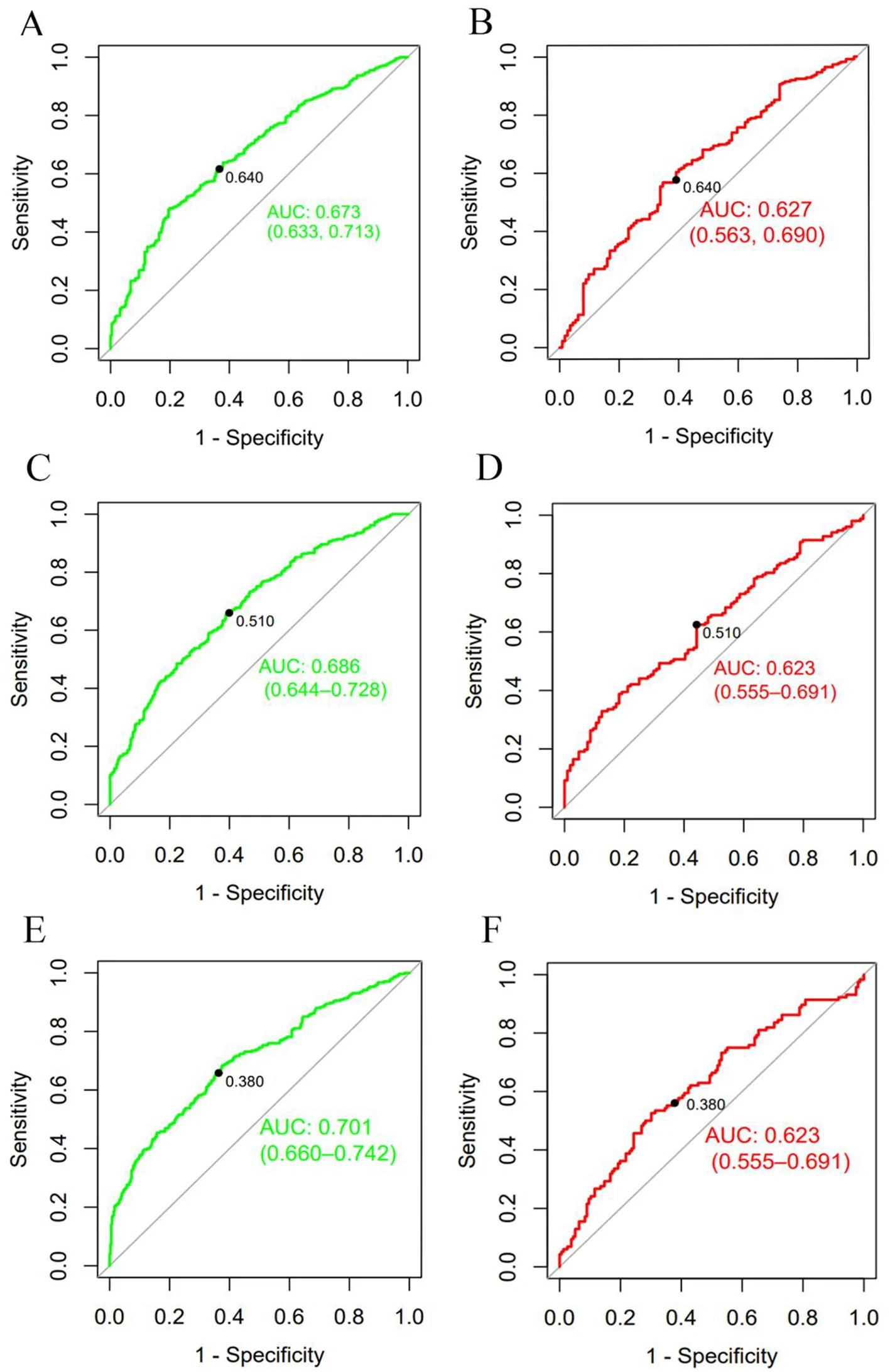
ROC curves for evaluating the discriminative ability. In the overall GIE cohort, the training and validation AUCs were 0.673 (95% CI: 0.633–0.713, **(A)** and 0.627 (95% CI: 0.563–0.690, **(B)**, respectively, with an optimal probability cut-off of 0.64. The EGD model showed AUCs of 0.686 (95% CI: 0.644–0.728, **(C)** in training and 0.623 (95% CI: 0.555–0.691, **(D)** in validation, using a cut-off value of 0.51. For the colonoscopy cohort, the model achieved the highest training AUC of 0.701 (95% CI: 0.660–0.742, **(E)** with a validation AUC of 0.623 (95% CI: 0.555–0.691, **(F)**, employing an optimal cut-off of 0.38.

Model performance was evaluated using the area under the receiver operating characteristic curve (AUC) (Figure 4). A predicted probability of ≥0.5 was used as the default classification threshold, consistent with standard practice for binary prediction models. In addition, the optimal cut-off for each cohort was determined using the Youden index, which maximizes the sum of sensitivity and specificity. These optimal thresholds represent the predicted probability values that best discriminate between positive and negative endoscopic findings.

**Figure 5:**
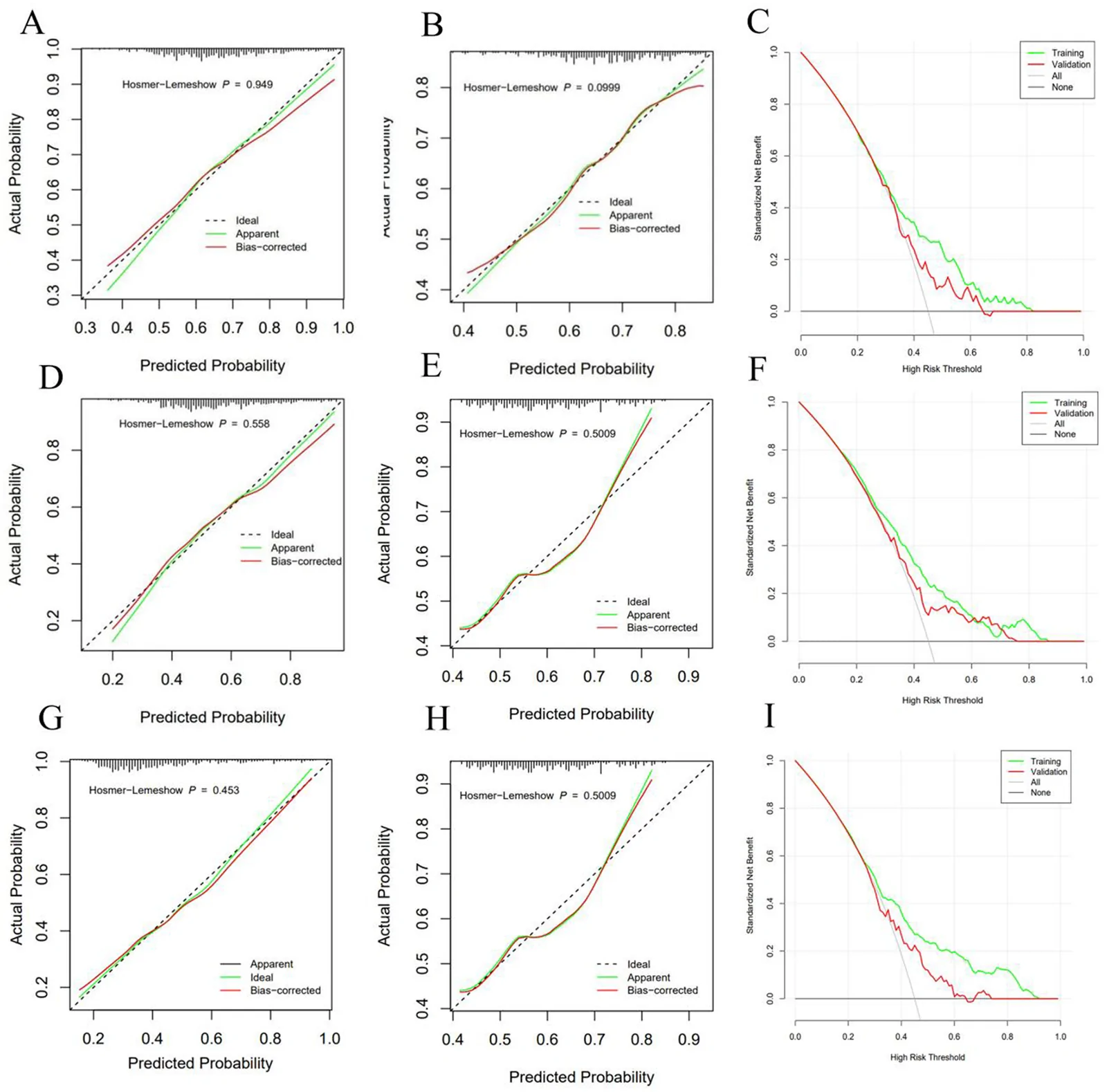
Model performance in the GIE group and its EGD and colonoscopy subgroups. **(A–C)** Calibration curves and DCA for the GIE group (training/validation); **(D–F)** EGD subgroup; **(G–I)** colonoscopy subgroup. Brier scores < 0.25 indicated good calibration. DCA showed net benefit across multiple threshold ranges, with optimal cut-offs of 0.51 (EGD) and 0.38 (colonoscopy).

In the overall GIE group, the training and validation AUCs were 0.673 (95% CI: 0.633–0.713, A) and 0.627 (95% CI: 0.563–0.690, B), respectively, with an optimal probability cut-off of 0.64. At this threshold, the validation performance for predicting positive endoscopic findings yielded a sensitivity of 57.66%, a specificity of 60.71%, a positive predictive value (PPV) of 74.42%, and a negative predictive value (NPV) of 41.98% (Table 9). The EGD model showed AUCs of 0.686 (95% CI: 0.644–0.728, C) in training and 0.623 (95% CI: 0.555–0.691, D) in validation, using a cut-off value of 0.51. At this threshold, the validation performance for predicting positive gastroscopic findings yielded a sensitivity of 62.50%, a specificity of 55.77%, a PPV of 67.38%, and a NPV of 50.43% (Table 9). For the colonoscopy group, the model achieved the highest training AUC of 0.701 (95% CI: 0.660–0.742, E) with a validation AUC of 0.623 (95% CI: 0.555–0.691, F), employing an optimal cut-off of 0.38. At this threshold, the validation performance for predicting positive colonoscopy findings yielded a sensitivity of 56.03%, a specificity of 62.18%, a PPV of 52.42%, and an NPV of 65.54% (Table 9). All models showed good calibration (Brier score < 0.25) and excellent fit (Hosmer-Lemeshow test *P* > 0.05). DCA indicated positive net benefit across clinically relevant threshold ranges, suggesting potential utility in supporting risk stratification, though this should be interpreted cautiously given the modest discriminative performance of the models (Figure 5).

**Table 9:** Model performance in training and validation cohorts.

Group	Cohort	AUC (95% CI)	Cut-off	Sensitivity (%)	Specificity (%)	PPV (%)	NPV (%)
GIE	Training	0.673 (0.633–0.713)	0.64	61.63%	63.35%	76.65%	45.82%
GIE	Validation	0.627 (0.563–0.690)	0.64	57.66%	60.71%	74.42%	41.98%
EGD	Training	0.686 (0.644–0.728)	0.51	65.98%	60.07%	67.17%	58.78%
EGD	Validation	0.623 (0.555–0.691)	0.51	62.50%	55.77%	67.38%	50.43%
Colonoscopy	Training	0.701 (0.660–0.742)	0.38	65.82%	63.69%	57.46%	71.43%
Colonoscopy	Validation	0.623 (0.555–0.691)	0.38	56.03%	62.18%	52.42%	65.54%

PPV, positive predictive value; NPV, negative predictive value.

The Brier score of the calibration training cohort and the verification cohort of the evaluation model calibration was less than 0.25. The calibration curve fitted relatively closely with the diagonal, and the prediction curve was close to the actual observation curve, indicating that the model calibration degree was good (Hosmer-Lemeshow *P* > 0.05, Figure 5). The clinical risk-benefit of the model was further evaluated via the decision curve analysis to predict the model (Figure 5). In the GIE group, the net benefit of the model exceeded zero within threshold probability ranges of 0.2–0.82 in the training cohort and 0.22–0.71 in the validation cohort (Figure 5C), suggesting useful clinical applicability within these intervals. For the EGD subgroup, the model showed a net benefit in the threshold ranges of 0.2–0.86 (training) and 0.32–0.75 (validation), with an optimal cut-off value of 0.51 (Figure 5F), supporting its clinical utility in this context. Similarly, in the colonoscopy subgroup, the net benefit was observed between thresholds of 0.25–0.90 (training) and 0.30–0.65 (validation), with a cut-off value of 0.38 (Figure 5I), indicating that the model is clinically applicable within this range.

## Discussion

Pediatric gastrointestinal endoscopy is essential for diagnosing and managing gastrointestinal disorders in children. However, as an invasive procedure, selecting appropriate candidates remains challenging, with no validated predictive models currently available. This study identified factors associated with positive endoscopic findings and developed three clinical prediction models (for GIE, EGD and colonoscopy) using readily available clinical and laboratory parameters. These models were validated for accuracy, sensitivity, and clinical applicability, offering a practical tool to guide endoscopic decision-making in children.

The overall positive endoscopic diagnostic rate was 64.9% (EGD: 55.5%; colonoscopy: 42.1%), consistent with previous reports (e.g., Miele et al.: 54% for EGD, 61.1% for colonoscopy) (10). However, disease patterns in our study differed from Western populations. While celiac disease (53.3%) and H. pylori infection (13.7%) were predominant in Miele et al.'s study (10), our patients most commonly exhibited gastritis (56.6%), duodenitis (31.8%), reflux esophagitis (8.7%), and non-specific colitis (63.9%, with IBD accounting for 3.8%). These differences likely reflect ethnic, dietary, and lifestyle variations. Given the extended study timeframe institutional experience with childhood IBD was limited in the early years of the study. Notably, non-specific colitis represented a substantial proportion of abnormal colonoscopy findings [8–27% in prior studies (13, 14)], suggesting potential undiagnosed IBD cases. Pediatric IBD often lacks classical endoscopic or histologic features compared to adult-onset disease (15), highlighting the need for improved diagnostic criteria in children.

The finding that abdominal pain was the most common indication for endoscopy is consistent with the results of a national multicenter study (11). However, detection rates were lower in patients with isolated abdominal pain than in those with additional symptoms (11). Functional gastrointestinal disorders are increasingly common in children (16). Endoscopy is typically reserved for suspected organic etiology, with diagnostic yields ranging from 11% to 56% in recurrent abdominal pain (17–19). Our identification of abdominal pain as a protective factor suggests possible overuse of endoscopy for this symptom alone, underscoring the need for comprehensive assessment incorporating laboratory and imaging data. Patricia et al. reported 39% of children with abdominal pain had normal colonoscopy, supporting restraint in the absence of other clinical features (20). Albumin, globulin, transferrin, and ferritin were strongly associated with endoscopic positivity, though the literature is limited. These proteins reflect nutritional and inflammatory status and may help identify occult chronic diseases (17).

Protective factors for positive endoscopic findings included serum calcium. Serum calcium was protective for both upper and lower endoscopy, suggesting low calcium may be linked to endoscopic abnormalities. Impaired calcium metabolism has been reported in chronic GI diseases (21), and vitamin D/calcium may help maintain intestinal homeostasis and prevent digestive disorders (22, 23). Independent risk factors were hematochezia, long-term medication use and globulin. Non-independent risk factors included weight, height, respiratory disease history, neutrophil percentage, lymphocyte percentage, albumin, and hs-CRP.

For EGD, protective factors included abdominal pain, diarrhea, and ALP. Independent risk factors were height and vomiting, melena, oral ulcer/rash/abdominal mass, ALB, γ-GGT, hs-CRP, and serum calcium were retained in the model. They lacked statistical significance in multivariable analysis, possibly due to the low prevalence of related conditions. A Canadian cohort study similarly identified age >13 years, vomiting, and hypoalbuminemia as predictors of positive upper endoscopy (24), aligning with our findings.

For colonoscopy, rectal bleeding, hypoalbuminemia, and elevated erythrocyte sedimentation rate were significant predictors in a study by Noble et al. (24). In our cohort, protective factors included abdominal tenderness and BUN. Independent risk factors were hematochezia, globulin, and ALT; non-independent risk factors were abdominal pain, MCH, abnormal routine stool, and occult blood in stool—retained due to clinical relevance to lower GI disorders. Elevated globulin may indicate subclinical celiac disease (25), or autoimmune conditions like IBD. In our study, globulin was an independent risk factor for positive colonoscopy, though disease-specific stratification was not performed.

We developed prediction models based on identified factors. Although some variables in the final model did not reach statistical significance in the multivariable logistic regression, they were retained because they were selected by LASSO regularization and contributed to the overall predictive performance. In prediction model development, variable selection based solely on *P* values is not recommended, as it may lead to overfitting and loss of calibration. ROC analysis showed acceptable discrimination (AUC > 0.6) (26). The AUCs ranged from 0.62 to 0.70 across the three cohorts, indicating modest discriminative performance. These values are comparable to those reported in similar pediatric prediction studies and reflect the inherent difficulty of predicting endoscopic findings from clinical and laboratory data alone. At the optimal cut-offs, the models achieved sensitivities of approximately 55–66% and specificities of 55–64%, suggesting they may offer modest support as a triage aid rather than a definitive diagnostic tool. Importantly, the clinical value of a prediction model should not be judged by AUC alone but by whether it provides incremental information beyond clinical judgment. At the reported performance levels, the models may assist in identifying children with higher pretest probability, potentially reducing unnecessary procedures while preserving diagnostic yield. This potential is further supported by DCA, which demonstrated positive net benefit across clinically relevant threshold ranges.

Several factors may have contributed to the modest discriminative performance observed in this study. First, the heterogeneous indications for endoscopy—ranging from chronic abdominal pain and hematochezia to suspected inflammatory bowel disease—encompassed a diverse population with varied underlying pathologies, increasing outcome variability and complicating prediction. Second, although we adopted a clinically meaningful definition of positive findings, its broad scope (including esophagitis, gastritis, peptic ulcer, H. pylori infection, polyps, and IBD) may have introduced heterogeneity within the outcome itself, making it more difficult for the model to identify a consistent set of predictors. Third, the retrospective design limited standardization of data collection and may have introduced information bias, as some variables were not systematically recorded. Fourth, the single-center population restricts generalizability, as patient demographics, referral patterns, and disease spectrum may differ across institutions. Fifth, evolving practice patterns over the 14 -year study period (2009–2023) including advances in endoscopic equipment, changes in diagnostic criteria (e.g., for H. pylori infection and eosinophilic esophagitis), and shifts in referral thresholds—may have introduced temporal heterogeneity that the model could not fully capture. Finally, the absence of potentially important predictors not collected in this study (e.g., novel biomarkers, genetic factor) may have further constrained the model's discriminative ability.

An AUC of approximately 0.62 indicates that the model captures only a modest proportion of the variability in endoscopic outcomes, reflecting the inherent complexity and heterogeneity of pediatric gastrointestinal disease.

In conclusion, we developed prediction models for positive endoscopic findings in three pediatric endoscopy cohorts. The models demonstrated modest discriminative ability and limited NPV at the 0.5 threshold, particularly for the GIE and EGD cohorts. At present, these models are not suitable for guiding avoidance of endoscopy. However, they may provide modest support for risk stratification and identifying higher-risk patients. Future studies should focus on improving NPV and specificity, or consider reframing the objective as a model for predicting negative endoscopic findings, to more directly address the clinical goal of reducing unnecessary procedures. Nonetheless, given the modest discrimination, these models are not yet for independent clinical use. External validation in prospective, multicenter cohorts is essential before any clinical application can be considered.

### Innovations and limitations

While international guidelines outline pediatric endoscopic indications (6, 27), many Chinese centers still follow adult guidelines. This study provides the first large-scale retrospective analysis over 15 years and introduces novel risk assessment models for pediatric endoscopy. To our knowledge, this is the first study to develop and validate three distinct prediction models based on separate correlation analyses, with predictors weighted by regression coefficients. Prévost et al. reported frequent inappropriate use of upper endoscopy in pediatric outpatients (28). Our study focused on inpatients; this may assist clinicians in identifying children with a higher probability of positive endoscopic findings, thereby supporting more informed decision-making regarding the indication for endoscopy. However, further studies with a negative-outcome design are needed to determine whether the model can reliably identify patients who could safely forgo the procedure.

Limitations include its single-center retrospective design and potential information bias. Inclusion of subjective variables and reliance on guardian-reported symptoms in young children may affect consistency. Due to the inclusion of 108 variables in this study, certain variables such as serum iron, ferritin, transferrin, etc. were significant in the univariate analysis, and iron deficiency was found to increase the rate of endoscopic diagnosis in children in a study by Fachler et al (29). Studies suggest fecal calprotectin and serum albumin are biomarkers for gastrointestinal graft-vs.-host disease (GI-GVHD) (30), supporting endoscopy for suspected GVHD in children. Although fecal calprotectin is an established, quantifiable inflammation marker in IBD (31), its role in pediatric gastrointestinal disorders is poorly studied (31, 32). Addressing this research gap in future by applying this assay in high-risk children could improve endoscopic diagnostics. To ensure the quality of diagnosis, although all endoscopists are professionally trained, heterogeneity cannot be completely eliminated due to the subjectivity of endoscopists. Due to the long-time span of this study, biopsies were not routinely taken for early endoscopy and therefore no separate analysis of histological findings was performed, which may have resulted in some underdiagnosis.

A major limitation of this study is the lack of external validation. While we employed a hold-out internal validation set to assess model performance, internal validation using data from the same center may still produce optimistic estimates due to shared patient characteristics, practice patterns, and data collection methods. External validation in independent, prospective, multi-center cohorts is therefore essential to confirm the models' generalizability and to assess their performance in real-world clinical settings.

### Future research directions

Future work should prioritize prospective, multicenter designs with standardized data collection to reduce bias and enhance generalizability. Pediatric endoscopy often includes routine biopsies to improve diagnostic yield (33, 34), as anesthesia and repeat procedure risks outweigh biopsy risks (35). Standardizing biopsy protocols and incorporating histology could enhance model performance. Including established indications such as growth failure, anorexia nervosa, and unexplained irritability may improve model sensitivity and specificity (6, 27).

To address the limitations of inter-center variability and lack of standardized protocols, multicenter data-sharing platforms and uniform procedures should be established. Narrowing the outcome definition to more homogeneous disease categories (e.g., IBD vs. polyps) may improve discrimination, as might the incorporation of novel biomarkers, genetic information, or advanced imaging features. Temporal validation using more recent cohorts would help assess stability amid evolving practice patterns, and model updating or recalibration techniques could adapt the models to local populations.

Ultimately, the clinical utility of these models can only be established through prospective implementation studies that evaluate their impact on clinical decision-making and patient outcomes, rather than through retrospective analyses alone. A well-designed prospective, multicenter study with standardized clinical variables and endoscopic outcomes would further validate and refine the models, improve generalizability, increase predictive accuracy, and support evidence-based decision-making in pediatric endoscopy.

## Data Availability

The original contributions presented in the study are included in the article/supplementary material, further inquiries can be directed to the corresponding authors.
