# Supplementary material for: Establish and validate a clinical predictive model for pediatric gastrointestinal endoscopy
Source: Front Med (Lausanne). 2026 Aug 13;13:1906112. doi: 10.3389/fmed.2026.1906112 (PMC13522975; doi:10.3389/fmed.2026.1906112)
Supplement: Supplementary file 1 [file Data_Sheet_1.pdf]

住院号	性别	年龄	民族	住址	体重	身高	BMI	体温
1334139	1	2	1	云南省红	27.0	134.0	15.0	36.7
1333450	1	2	1	曲靖市陆	30.0	159.0	11.9	36.6
1340260	0	2	1	曲靖市马	68.0	156.0	27.9	36.8
1335426	0	2	1	曲靖市会	25.0	130.0	14.8	36.8
1334911	0	3	1	大理州弥	50.0	160.0	19.5	36.5
1333312	0	2	1	思茅市思	27.0	141.0	13.6	36.5
1341265	1	2	1	德宏州维	34.0	140.0	17.3	36.5
1332170	1	3	1	德宏瑞丽	45.0	162.0	17.1	36.8
1329559	1	3	1	贵州省安	35.0	148.0	16.0	37.0
1328608	1	2	1	盘县马坝	22.0	145.0	10.5	36.6
1326173	1	2	1	临沧凤庆	33.0	140.0	16.8	36.8
1320708	0	3	1	玉溪易门	52.0	170.0	18.0	36.8
1320018	1	2	1	昭通市巧	26.0	128.0	15.9	36.8
1319648	0	2	1	德宏州芒	30.0	135.0	16.5	36.6
1319357	0	2	1	曲靖会泽	19.0	130.0	11.2	37.0
1318420	1	2	1	曲靖市陆	30.0	159.0	11.9	36.8
1317924	0	2	1	云南省昆	22.0	145.0	10.5	36.6
1317757	1	2	1	贵州盘县	60.0	170.0	20.8	36.8
1317257	1	3	1	贵州兴义	44.0	165.0	16.2	36.5
1316641	0	2	1	寻甸县金	26.0	142.0	12.9	36.5
1316184	1	3	1	楚雄市鹿	43.0	170.0	14.9	36.7
1316168	1	3	1	宣威西宁	46.0	160.0	18.0	36.8
1315870	1	2	0	临沧市云	26.0	148.0	11.9	36.5
1315426	1	2	1	临沧云县	24.0	140.0	12.2	36.5
1313838	1	2	1	思茅和骨	18.5	130.0	10.9	36.9
1313455	1	2	1	云南省保	30.0	145.0	14.3	36.2
1313156	1	2	1	楚雄武定	31.0	148.0	14.2	36.8
1311649	1	2	1	昭通市巧	20.0	151.0	8.8	37.0
1309933	1	2	1	禄劝县屏	34.0	146.0	16.0	36.5
1308658	1	2	1	李向东*南	20.0	119.0	14.1	36.8
1307902	0	2	1	楚雄牟定	19.0	137.0	10.1	36.8
1301009	1	2	0	红河石屏	22.0	146.0	10.3	36.5
1300615	1	2	1	曲靖市富	30.0	145.0	14.3	36.8
1298464	1	2	1	临沧市凤	80.0	158.0	32.0	36.8
1298048	0	3	1	楚雄州禄	57.0	160.0	22.3	36.2
1296259	1	2	1	临沧市临	32.0	130.0	18.9	37.0
1295765	0	2	0	景洪市景	22.0	115.0	16.6	36.4
1289830	0	2	1	临沧耿马	33.0	150.0	14.7	36.5
1285480	0	2	1	保山市隆	22.0	135.0	12.1	36.5
1284233	1	3	1	玉溪市通	46.0	150.0	20.4	36.6
1284066	1	2	1	曲靖市师	29.0	155.0	12.1	36.5
1282516	0	3	1	呈贡县七	48.0	160.0	18.8	37.0
1282209	1	3	1	春城路250	54.0	170.0	18.7	36.7
1281557	1	2	1	昭通巧家	32.0	120.0	22.2	36.6
1281495	1	2	1	曲靖市富	32.0	135.0	17.6	36.3
1278575	1	2	1	环城西路1	45.0	140.0	23.0	36.5
1276979	0	2	1	宣威普立	32.0	134.0	17.8	36.8
1276794	0	3	1	泸西白水	43.0	150.0	19.1	36.4
1275191	0	3	0	怒江州兰	53.0	165.0	19.5	36.5
1274796	0	2	1	华山东路8	43.0	154.0	18.1	36.8
1274291	1	3	1	富源县大	30.0	140.0	15.3	36.7
1273536	1	2	1	贵州威宁	32.0	145.0	15.2	37.0
1273249	0	3	0	大理大理	29.0	142.0	14.4	36.4

1273212	1	3	0 怒江州兰坪	35.0	140.0	17.9	36.7
1272768	1	3	1 昭通市昭阳	57.0	164.0	21.2	36.6
1272039	1	2	1 昭通市镇雄	50.0	147.0	23.1	36.5
1270625	1	3	1 梁河县小厂	57.0	146.0	26.7	36.6
1270144	1	2	1 楚雄州姚安	24.0	131.0	14.0	36.0
1269622	1	3	1 五华区西翥	62.0	170.0	21.5	36.0
1267829	0	3	1 昆明市晋宁	60.0	162.0	22.9	36.0
1265764	1	2	1 昆明市盘龙	37.0	115.0	28.0	36.5
1261941	1	2	1 曲靖市陆良	37.5	154.0	15.8	37.0
1261726	1	2	1 昭通市威信	26.0	130.0	15.4	36.8
1260712	0	1	1 昭通市彝良	28.0	100.0	28.0	37.3
1259749	1	3	1 大理弥渡县	50.0	149.0	22.5	36.7
1258870	1	3	0 云南省昆明	53.0	169.0	18.6	36.6
1256932	1	2	1 呈贡县沿沟	30.0	125.0	19.2	36.5
1256528	1	2	1 安宁市安宁	23.0	120.0	16.0	36.8
1249135	1	2	1 昭通市巧家	58.0	140.0	29.6	36.8
1247443	0	2	1 昆明市高新	17.0	115.0	12.9	36.5
1242845	0	3	1 普洱市田坝	49.0	152.0	21.2	36.5
1241543	0	3	1 玉溪市江川	50.0	158.0	20.0	36.7
1241257	1	2	1 德宏州景谷	35.0	140.0	17.9	36.5
1236842	0	2	1 昭通市镇雄	55.0	152.0	23.8	36.7
1235417	0	3	1 楚雄八角乡	32.0	158.0	12.8	36.8
1235313	0	2	1 红河州个旧	24.0	119.0	16.9	37.0
1234865	1	3	1 大理祥云县	45.0	107.0	39.3	36.6
1234555	1	3	1 曲靖陆良县	37.0	146.0	17.4	36.3
1232130	1	2	1 宣威市乐丰	40.0	156.0	16.4	36.0
1231569	1	3	1 宣威市格宜	40.0	130.0	23.7	36.6
1230899	1	2	1 玉溪市尚义	50.0	155.0	20.8	36.2
1228474	0	2	1 黑林铺农場	18.0	116.0	13.4	36.5
1226814	1	3	1 昭通市鲁甸	44.0	169.0	15.4	36.5
1226778	1	2	1 玉溪通海县	38.0	141.0	19.1	38.1
1222555	1	2	1 昆明市嵩明	48.0	142.0	23.8	36.7
1216697	0	3	1 贵州威宁县	38.0	150.0	16.9	36.2
1216349	1	2	1 昆明市寻甸	39.0	130.0	23.1	36.8
1213804	1	3	1 曲靖市会泽	65.0	165.0	23.9	36.4
1213384	0	2	1 昭通市镇雄	22.0	130.0	13.0	36.8
1211445	0	2	1 曲靖市师宗	19.5	112.0	15.5	36.5
1211131	0	2	1 泸水县园丁	16.0	105.0	14.5	36.8
1211016	1	2	0 迪庆香格里	23.0	130.0	13.6	36.6
1210588	0	2	1 五华区普吉	42.0	135.0	23.0	36.2
1210434	1	2	1 昭通巧家县	26.0	130.0	15.4	36.5
1210232	1	3	1 贵州盘县新	29.0	147.0	13.4	37.0
1209315	1	2	1 崇明县小街	59.0	150.0	26.2	36.3
1208934	0	3	1 昆明市东川	34.0	150.0	15.1	36.5
1208700	1	3	1 曲靖市麒麟	55.0	163.0	20.7	36.4
1207891	1	3	1 石林县以旅	26.0	149.0	11.7	36.6
1205354	0	3	1 临沧市永德	50.0	162.0	19.1	36.5
1204453	1	2	1 贵州盘县红	20.0	103.0	18.9	36.7
1204280	0	2	1 昆明市西山	25.0	127.0	15.5	37.7
1203946	0	2	1 昆明市东川	21.0	115.0	15.9	36.6
1203823	1	2	0 昆明市石林	55.0	156.0	22.6	36.5
1203379	1	2	1 临沧市凤庆	31.0	138.0	16.3	36.5
1199413	1	2	1 昆明市五华	24.0	135.0	13.2	36.2
1197023	1	2	1 昆明市官渡	32.0	145.0	15.2	36.0

1193712	0	2	0	普洱市墨	19.5	106.0	17.4	36.6
1192472	0	2	0	丽江市古	52.0	160.0	20.3	36.6
1188192	1	3	0	红河州泸	42.0	156.0	17.3	37.1
1187284	0	2	0	楚雄州姚	39.0	152.0	16.9	36.8
1184518	1	2	1	宣威市田	32.0	145.0	15.2	36.7
1183327	1	3	1	贵州省六	42.0	155.0	17.5	36.4
1180998	0	3	1	曲靖富源	26.0	135.0	14.3	36.5
1179289	0	2	1	昆明市滇	32.0	142.0	15.9	36.5
1178407	1	2	0	红河县大	29.0	145.0	13.8	37.0
1177786	0	2	0	红河州弥	41.0	150.0	18.2	36.7
1175590	1	2	1	晋宁县昆	36.0	150.0	16.0	36.5
1174824	1	3	1	昭通市镇	50.0	158.0	20.0	37.2
1173738	1	3	1	昭通市永	28.5	130.0	16.9	36.6
1172698	1	2	1	丘北县树	36.0	150.0	16.0	36.5
1172063	0	2	1	陶义昌**	25.0	132.0	14.3	36.5
1171265	0	2	0	玉溪新平	34.0	142.0	16.9	36.8
1170794	1	2	1	昭通市镇	30.0	140.0	15.3	36.6
1161575	1	3	0	禄丰县中	40.0	155.0	16.6	36.4
1159942	1	3	1	香格里拉	47.0	153.0	20.1	36.8
1159856	1	2	1	德宏州梁	29.0	140.0	14.8	36.8
1158497	1	2	1	普洱市景	30.0	136.0	16.2	37.0
1155118	1	3	1	昭通市彝	45.0	142.0	22.3	37.0
1150490	1	2	1	曲靖师宗	18.0	120.0	12.5	37.0
1149335	1	3	1	德宏州芒	40.0	150.0	17.8	37.0
1148710	1	2	1	曲靖市会	30.0	140.0	15.3	37.0
1144664	1	3	0	景洪市东	47.0	160.0	18.4	36.6
1143415	1	2	1	普洱景谷	35.0	150.0	15.6	36.5
1143050	1	2	0	怒江州福	30.0	147.0	13.9	36.8
1140448	1	2	1	宣威市龙	20.0	135.0	11.0	36.6
1137655	0	3	1	昆明市呈	52.0	155.0	21.6	36.5
1136462	1	3	0	普洱市镇	22.0	140.0	11.2	36.5
1136316	1	3	1	昆明市嵩	40.0	170.0	13.8	38.0
1134363	0	3	1	昭通市巧	50.0	160.0	19.5	36.3
1133564	1	3	1	保山市昌	35.0	145.0	16.6	37.0
1133261	1	2	1	禄劝县马	29.0	139.0	15.0	36.2
1132659	1	2	1	新兴路29	75.0	165.0	27.5	36.5
1131855	0	2	1	金洲湾蓝	18.0	120.0	12.5	37.0
1130413	0	2	0	凤庆县营	27.0	142.0	13.4	36.5
1127235	0	3	1	昆明宜良	47.0	150.0	20.9	36.7
1127083	1	2	1	学府路651	25.0	122.0	16.8	37.0
1125174	1	2	1	西山区船	23.0	120.0	16.0	36.8
1124596	1	2	0	临沧凤庆	28.0	135.0	15.4	36.3
1116248	0	2	1	马龙县鸡	26.0	135.0	14.3	36.8
1108964	0	3	1	玉溪市澄	27.0	134.0	15.0	37.0
1105979	1	2	0	西双版纳	50.0	146.0	23.5	36.5
1104854	1	2	1	文山州广	34.0	150.0	15.1	36.6
1101403	1	2	1	红河弥勒	34.0	142.0	16.9	36.8
1094401	0	3	1	丽江市古	56.0	164.0	20.8	36.5
1092929	0	2	1	保山隆阳	42.0	161.0	16.2	36.5
1086122	0	3	1	云南省曲	45.0	150.0	20.0	36.3
1085895	1	2	1	石林县石	36.0	148.0	16.4	36.4
1084665	0	2	1	保山市龙	30.0	132.0	17.2	36.7
1083417	0	3	1	牟定县凤	47.0	155.0	19.6	36.5
1080989	1	2	1	金实小区	19.5	119.0	13.8	37.0

1080335	0	2	1	晋宁县晋坊	47.0	153.0	20.1	36.5
1079167	0	2	1	贵州盘县;	30.0	145.0	14.3	37.0
1077021	1	3	1	玉溪市通淅	30.0	141.0	15.1	36.5
1075091	1	3	0	贵州省盘县	52.0	152.0	22.5	37.0
1074489	0	2	1	东风西路2	34.0	156.0	14.0	36.4
1073910	1	2	1	昆明市晋宁	31.0	135.0	17.0	37.2
1070758	1	3	1	昆钢老东区	67.0	170.0	23.2	36.3
1067739	0	2	1	玉溪市峨山	25.0	143.0	12.2	37.0
1067275	0	2	1	会泽县鲁纟	30.0	121.0	20.5	36.8
1064602	0	2	1	禄劝县马房	40.0	150.0	17.8	36.5
1061260	0	2	1	富源县黄氵	60.0	157.0	24.3	36.8
1061157	1	2	1	嵩明小街衤	31.0	155.0	12.9	36.8
1058737	1	3	1	东川区乌龙	31.0	140.0	15.8	36.7
1057435	1	2	1	大理剑川县	19.5	121.0	13.3	37.0
1051979	1	2	1	昭通市彝良	26.0	144.0	12.5	36.8
1048826	1	2	1	宣威市龙潭	21.0	117.0	15.3	36.7
1046511	0	2	1	楚雄州南华	19.0	120.0	13.2	36.5
1040915	1	2	1	弥勒县西山	32.0	148.0	14.6	36.5
1040720	1	3	1	宣威市东乡	50.0	148.0	22.8	36.7
1033104	1	3	1	丽江市永胜	40.0	150.0	17.8	36.5
1032709	1	3	0	丽江宁蒗县	30.0	140.0	15.3	36.7
1032425	0	2	0	宣威来宾钅	35.0	149.0	15.8	36.5
1031727	0	2	1	保山昌宁县	27.5	141.0	13.8	36.5
1025371	1	3	1	建水县坡头	51.0	165.0	18.7	36.0
1017364	1	2	1	富源县大河	27.0	137.0	14.4	37.0
1015661	0	2	1	高新开发区	30.0	132.0	17.2	36.7
1014148	0	3	1	玉溪市江川	48.0	160.0	18.8	35.8
1013879	1	2	1	寻甸县凤合	26.0	150.0	11.6	39.0
1011555	0	3	0	禄丰县彩云	50.0	155.0	20.8	37.0
991974	0	3	1	祥云县下庄	28.0	142.0	13.9	36.5
990391	1	2	1	双柏县二六	35.0	135.0	19.2	36.4
989507	0	2	1	官渡区大木	22.0	132.0	12.6	37.0
986953	1	2	1	大理巍山县	32.0	156.0	13.1	36.8
982761	1	2	0	大理洱源娃	35.0	145.0	16.6	36.8
973937	0	2	0	保山昌宁阝	25.0	130.0	14.8	36.5
972763	1	3	1	曲靖南苑小	54.0	160.0	21.1	37.0
966669	1	3	1	昆明市禄劝	32.0	141.0	16.1	36.2
966454	1	2	1	宣威市东山	23.0	120.0	16.0	36.8
966346	0	2	0	昆明第19中	23.0	135.0	12.6	36.8
962963	1	3	1	曲靖麒麟区	46.0	154.0	19.4	36.2
961123	1	2	0	昭通昭阳区	26.0	132.0	14.9	38.2
960138	0	2	1	云南牟定县	24.0	139.0	12.4	37.8
957608	1	2	1	曲靖市会泽	26.0	140.0	13.3	37.1
955214	1	3	1	玉溪市新平	24.0	135.0	13.2	36.8
954155	1	3	1	红河州元江	40.0	155.0	16.6	35.8
953122	0	2	1	禄丰县碧城	44.0	156.0	18.1	36.8
950335	0	2	1	大姚县石羊	22.0	126.0	13.9	36.4
948136	1	3	0	大理剑川县	63.0	160.0	24.6	36.8
939761	0	3	1	大理鹤庆六	60.0	165.0	22.0	36.3
937732	0	2	1	宜良县汤池	24.0	134.0	13.4	37.0
931237	1	2	0	云南省勐腊	20.5	135.0	11.2	37.0
930300	1	2	1	昆明市五华	24.5	136.0	13.2	35.6
930247	1	2	1	云南梁河县	35.0	136.0	18.9	36.2
927294	0	3	1	晋宁县新街	42.5	148.0	19.4	36.5

924988	1	3	1	昆明市鼓	43.0	165.0	15.8	36.7
922714	0	2	1	呈贡马金	16.0	108.0	13.7	36.2
922243	0	2	1	昆明市五	24.0	132.0	13.8	36.5
920623	1	3	1	石屏县异	67.0	163.0	25.2	36.8
918744	1	2	1	富民县景	28.5	142.0	14.1	36.6
916496	0	2	1	昭通市下	36.0	143.0	17.6	37.3
917145	1	3	0	鲁甸县文	32.0	140.0	16.3	36.7
916637	0	2	1	昭通市下	22.0	121.0	15.0	36.8
914962	1	3	1	昆明市富	45.0	160.0	17.6	36.8
912344	1	3	1	贵州盘县	40.5	160.0	15.8	36.8
911710	1	2	1	昆明市北	21.0	125.0	13.4	36.8
906702	0	2	0	禄劝贫山	39.0	148.0	17.8	36.6
906699	0	3	1	富源富村	44.0	148.0	20.1	36.5
905732	1	2	1	昭通市昭	40.0	152.0	17.3	37.8
905144	1	2	1	晋宁昆阳	22.5	135.0	12.3	36.5
904178	1	3	1	砚山县平	45.0	160.0	17.6	36.7
903357	1	2	1	云南省昆	15.5	120.0	10.8	36.7
903346	0	3	1	云南鹤庆	57.0	163.0	21.5	36.6
902668	1	2	1	玉溪中卫	23.0	133.0	13.0	37.0
902608	1	2	1	德宏州梁	28.0	156.0	11.5	36.5
898607	0	3	1	昭通市巧	50.0	152.0	21.6	37.2
897823	1	2	1	云南省宣	22.0	130.0	13.0	36.2
897671	1	2	1	云南省宾	24.0	145.0	11.4	37.0
897404	1	2	1	云南省镇	23.0	130.0	13.6	36.7
892004	0	2	0	贵州省硝	35.0	158.0	14.0	36.4
891107	1	2	1	云南省宣	35.0	150.0	15.6	36.7
884675	0	2	1	云南省宜	27.5	150.0	12.2	36.5
883233	1	2	1	云南省昭	30.0	129.0	18.0	36.3
876006	1	2	1	昆明市人	27.0	137.0	14.4	35.8
873881	0	2	1	云南省普	35.0	142.0	17.4	36.3
872304	1	2	1	云南省玉	17.0	112.0	13.6	36.8
871188	1	2	0	云南省普	27.0	130.0	16.0	36.5
869633	1	2	1	云南省昭	34.0	152.0	14.7	37.4
862750	0	3	1	云南省鹤	52.0	165.0	19.1	37.5
861832	1	3	0	云南省镇	52.0	158.0	20.8	36.6
861399	0	3	0	云南省昭	30.0	150.0	13.3	36.8
861311	1	3	0	云南省昆	42.0	155.0	17.5	36.4
849933	1	2	0	昆明市世	31.0	156.0	12.7	37.0
837625	1	2	1	云南省昆	18.0	110.0	14.9	36.6
828895	1	3	1	云南省昆	40.0	153.0	17.1	38.2
826287	1	3	0	云南省昆	42.0	156.0	17.3	37.8
819288	0	2	0	云南省安	23.5	140.0	12.0	37.3
807680	1	3	1	贵州盘县	44.0	155.0	18.3	36.4
804862	1	3	1	云南省曲	45.0	158.0	18.0	36.5
802891	1	2	1	云南省昆	38.0	135.0	20.9	35.8
753593	1	2	1	云南省镇	24.0	132.0	13.8	36.6
753092	1	2	1	云南省昭	26.0	140.0	13.3	36.8
751941	1	3	1	云南省腾	30.0	147.0	13.9	36.5
747160	1	2	1	云南省昆	40.0	145.0	19.0	36.3
746460	0	2	1	云南省弥	12.0	90.0	14.8	36.2
745427	1	3	1	云南省昆	37.0	150.0	16.4	37.0
744540	1	2	0	云南省大	34.0	143.0	16.6	36.8
740834	0	2	1	云南省曲	35.0	127.0	21.7	36.8
739843	1	2	0	云南省普	20.0	135.0	11.0	37.3

738037	1	2	1	云南省曲	22.0	120.0	15.3	38.1
738021	0	2	1	云南省楚	30.0	132.0	17.2	36.0
735574	0	2	1	云南省普	35.5	110.0	29.3	36.6
735296	1	2	0	云南省普	22.0	135.0	12.1	37.0
733778	1	2	1	云南省大	27.0	135.0	14.8	36.3
733133	1	2	1	云南省昆	37.0	141.0	18.6	36.6
732564	1	3	1	云南省文	36.0	141.0	18.1	36.3
731838	1	2	0	云南省曲	36.0	133.0	20.4	36.5
729793	1	2	1	云南省楚	27.0	130.0	16.0	36.8
729142	1	2	1	云南省昆	25.0	120.0	17.4	36.2
729106	1	2	1	云南省曲	23.0	115.0	17.4	36.5
728485	1	2	0	云南省楚	35.0	135.0	19.2	37.0
727084	0	3	1	云南省昆	30.0	159.0	11.9	37.2
723271	0	3	1	云南省曲	39.0	160.0	15.2	36.4
721814	1	3	1	云南省曲	40.0	155.0	16.6	37.2
720214	1	3	1	云南省保	45.0	162.0	17.1	36.5
719272	1	2	1	云南省丽	13.0	115.0	9.8	36.5
715431	1	3	1	云南省大	50.0	158.0	20.0	36.3
712497	0	2	1	云南省宣	40.0	155.0	16.6	36.5
711537	0	2	1	云南省宣	20.0	110.0	16.5	36.6
710652	1	2	1	云南省昭	31.0	145.0	14.7	36.3
708778	0	2	0	云南省楚	22.0	136.0	11.9	36.5
706263	0	2	0	云南省昆	27.0	120.0	18.8	36.5
696040	1	2	1	云南省保	27.0	136.0	14.6	36.7
692470	1	2	1	云南省禄	27.0	120.0	18.8	36.6
688636	0	2	1	云南省大	28.0	105.0	25.4	36.4
688604	1	2	0	云南省盈	16.0	102.0	15.4	36.7
666307	1	2	1	云南省昭	25.0	106.0	22.2	36.9
665212	1	3	1	云南省曲	52.0	158.0	20.8	36.5
664307	1	3	1	云南省丽	55.0	160.0	21.5	36.3
656457	1	2	1	云南省德	20.0	125.0	12.8	36.3
651236	1	3	1	云南省曲	32.0	145.0	15.2	36.8
631428	1	3	1	云南省昭	45.0	154.0	19.0	36.8
630976	1	2	1	云南省曲	25.0	142.0	12.4	36.8
630104	1	2	1	云南省玉	29.0	150.0	12.9	37.2
629988	1	2	1	云南省丽	39.0	129.0	23.4	36.5
629949	1	2	1	云南省曲	25.0	125.0	16.0	38.5
556764	1	2	1	云南省曲	21.0	128.0	12.8	36.3
2812311	1	2	0	楚雄州禄	47.0	145.0	22.4	36.6
2811232	1	2	1	云南省曲	30.0	156.0	12.3	36.5
2810448	1	3	1	云南省昆	35.0	155.0	14.6	36.5
2810434	0	3	1	云南省楚	50.0	155.0	20.8	36.5
2809342	1	1	1	云南省普	11.0	80.0	17.2	36.8
2808167	0	3	0	普洱市景	47.0	155.0	19.6	36.3
2806841	0	1	1	昆明市泰	11.0	93.0	12.7	35.5
2805684	1	2	0	昆明市子	20.0	131.0	11.7	36.5
2804551	0	3	0	昆明市五	47.0	161.0	18.1	36.9
2804305	1	3	1	云南省宣	45.0	160.0	17.6	36.5
2803393	0	2	0	景洪市南	45.0	150.0	20.0	36.8
2801511	1	2	1	昆明市五	24.0	145.0	11.4	36.8
2801402	0	3	0	财大西区1	57.0	161.0	22.0	36.5
2801092	1	2	1	云南曲靖	49.0	155.0	20.4	36.3
2801057	1	1	1	云南省昆	38.0	148.0	17.3	36.5
2800863	1	1	1	安宁市大	21.0	142.0	10.4	36.8

2800821	1	2	1	宣威市美	41.0	150.0	18.2	36.7
2800324	1	2	1	昆明市呈	76.0	156.0	31.2	36.8
2798697	1	2	1	云南省宣	34.0	152.0	14.7	36.7
2798667	0	2	1	昆明市嵩	50.0	167.0	17.9	36.8
2798241	1	2	1	会泽县瑞	34.0	139.0	17.6	36.7
2798140	1	2	1	昆明市北	36.0	130.0	21.3	36.8
2798136	1	3	1	云南省昆	35.0	155.0	14.6	36.8
2797947	1	3	1	昆明市西	59.0	168.0	20.9	36.8
2796384	0	2	1	红河州泸	16.0	107.0	14.0	36.8
2796333	0	3	1	云南省昆	48.0	160.0	18.8	36.8
2795933	0	2	1	楚雄州法	30.0	150.0	13.3	36.8
2795589	0	3	1	云南省曲	38.0	140.0	19.4	36.9
2795298	0	2	1	云南省昆	49.0	140.0	25.0	36.8
2793440	1	3	1	贵州省水	30.0	146.0	14.1	36.8
2793061	1	2	1	贵州毕节	32.0	145.0	15.2	36.8
2792872	0	2	1	云南省红	23.0	125.0	14.7	36.7
2792845	1	3	0	云南省昆	40.0	163.0	15.1	36.3
2792220	0	2	0	楚雄州禄	31.0	140.0	15.8	36.5
2791774	1	3	1	贵州省兴	31.5	155.0	13.1	36.8
2791267	1	2	1	昆明市官	73.0	167.0	26.2	36.8
2789404	1	2	1	云南省文	20.0	127.0	12.4	36.8
2787040	0	3	1	贵州省安	45.0	156.0	18.5	36.3
2785241	1	2	1	安宁市福	19.0	117.0	13.9	38.8
2784095	0	3	1	云南省麒	41.0	156.0	16.8	36.0
2783210	1	3	1	宣威市西	35.0	160.0	13.7	36.8
2782555	0	2	0	云南省玉	20.0	116.0	14.9	36.9
2781814	0	2	0	楚雄州大	30.0	152.0	13.0	36.8
2780595	1	2	1	昆明市石	28.0	125.0	17.9	36.8
2778911	1	2	1	临沧市云	35.0	158.0	14.0	36.1
2776877	1	2	1	普洱市镇	33.0	141.0	16.6	36.6
2776612	1	2	1	宣威市羊	22.0	128.0	13.4	36.8
2775768	0	2	1	云南省文	15.0	112.0	12.0	37.8
2774228	1	3	0	云南省迪	40.0	158.0	16.0	36.7
2773592	0	3	1	昭通市昭	50.0	154.0	21.1	36.8
2771802	1	2	0	贵州省威	46.0	142.0	22.8	36.5
2769463	1	1	1	思茅区思	11.0	80.0	17.2	38.5
2768235	0	3	0	楚雄彝族	52.0	158.0	20.8	38.9
2767998	1	2	0	昆明市盘	24.0	130.0	14.2	36.2
2767301	0	2	1	呈贡江尾	42.0	151.0	18.4	36.3
2766501	1	2	1	保山市龙	32.0	135.0	17.6	36.8
2766002	1	2	1	昆明市新	48.0	135.0	26.3	36.2
2764334	1	2	0	昆明市官	22.0	95.0	24.4	36.5
2761941	1	3	1	曲靖市会	54.0	152.0	23.4	36.5
2761934	1	2	1	昆明市嵩	33.0	148.0	15.1	36.6
2761738	0	2	0	红河州弥	42.0	142.0	20.8	36.4
2761050	1	2	1	昆明市寻	42.0	137.0	22.4	36.5
2760607	1	2	1	云南省丽	29.0	138.0	15.2	36.2
2760255	0	2	1	昆明市五	21.0	124.0	13.7	36.8
2759704	0	2	1	云南省红	37.0	149.0	16.7	36.8
2757168	1	3	1	吴井路白	70.0	185.0	20.5	36.8
2756548	0	2	1	云南省曲	21.0	136.0	11.4	36.7
2755616	0	2	1	宣威市西	33.0	152.0	14.3	36.5
2754833	0	3	1	东川攀枝	56.0	163.0	21.1	36.8
2754811	1	2	1	云南省昆	53.0	146.0	24.9	36.7

2754793	0	3	1	云南省昆	63.0	156.0	25.9	36.6
2752573	1	3	0	云南省迪	48.0	172.0	16.2	36.8
2751978	1	2	0	云南省红	55.0	160.0	21.5	36.8
2751571	1	3	1	云南省昆	43.0	165.0	15.8	36.6
2750453	0	3	0	云南省玉	48.0	157.0	19.5	36.5
2750324	0	2	1	曲靖市师	43.0	152.0	18.6	36.8
2750245	0	2	1	云南省保	30.0	152.0	13.0	36.5
2749874	0	2	1	昆明市嵩	47.0	160.0	18.4	36.8
2749541	1	2	0	云南省楚	21.0	140.0	10.7	36.8
2748790	1	2	1	曲靖市会	28.0	150.0	12.4	36.5
2747692	0	3	1	昆明市晋	41.0	151.0	18.0	36.5
2746854	1	2	1	昆明市穿	40.0	100.0	40.0	36.5
2746782	1	2	1	曲靖市马	30.0	152.0	13.0	36.8
2745740	0	3	1	云南省玉	55.0	162.0	21.0	36.7
2743540	1	3	1	保山市龙	38.0	155.0	15.8	36.3
2743233	1	2	0	云南省贵	20.0	120.0	13.9	36.8
2742367	1	2	1	云南省陆	30.0	152.0	13.0	36.3
2741860	1	3	1	宣威市来	55.0	165.0	20.2	36.6
2741839	1	2	1	保山市施	27.0	130.0	16.0	36.5
2741029	1	3	1	昭通市巧	31.0	142.0	15.4	36.5
2740974	1	2	0	思茅地区	40.0	130.0	23.7	36.5
2739867	0	2	1	宣威市得	32.0	132.0	18.4	36.5
2739031	0	2	0	昆明市经	23.0	125.0	14.7	36.7
2738427	1	2	0	文山壮族	23.0	135.0	12.6	36.6
2738401	0	2	0	云南省宜	32.0	141.0	16.1	36.5
2738043	0	3	1	昆明市东	34.0	138.0	17.9	36.6
2738039	1	2	1	曲靖市会	43.0	162.0	16.4	36.5
2737205	0	3	1	文山州丘	45.0	160.0	17.6	36.5
2736606	1	2	0	云南省蒙	26.0	136.0	14.1	36.8
2734803	1	3	1	贵州省水	43.0	160.0	16.8	36.4
2732021	0	2	1	贵州省威	26.0	134.0	14.5	36.8
2731836	1	2	1	云南省迪	26.0	135.0	14.3	36.8
2731523	1	2	0	普洱市景	28.0	135.0	15.4	36.2
2731251	0	2	1	曲靖市富	33.0	145.0	15.7	36.5
2730750	0	2	1	云南省昭	16.0	120.0	11.1	36.5
2728885	0	2	1	云南省红	20.0	110.0	16.5	36.6
2728580	1	3	1	玉溪市江	45.0	165.0	16.5	36.5
2728536	1	3	1	丽江市玉	50.0	170.0	17.3	36.3
2727936	1	2	1	云南省保	30.0	135.0	16.5	36.8
2726205	0	2	1	昆明市官	34.0	147.0	15.7	36.8
2724743	1	2	1	云南省保	29.0	145.0	13.8	36.6
2723621	1	2	0	云南省丽	23.0	123.0	15.2	36.6
2721588	0	3	1	昆明市寻	53.0	157.0	21.5	36.5
2720354	0	3	1	贵州省普	28.0	120.0	19.4	36.6
2719955	1	2	1	云南省龙	30.0	134.0	16.7	36.3
2719469	1	2	1	云南省保	32.0	139.0	16.6	36.5
2719399	0	3	1	云南省昆	43.0	150.0	19.1	36.5
2714247	1	3	0	云南省普	60.0	165.0	22.0	36.3
2712601	1	2	1	昆明市禄	29.5	132.0	16.9	36.4
2710321	1	3	1	云南省昆	98.0	193.0	26.3	36.6
2707867	1	2	1	云南省昭	28.0	130.0	16.6	39.0
2706070	1	3	1	云南省红	65.0	164.0	24.2	37.0
2703988	1	3	0	云南省昆	90.0	162.0	34.3	36.8
2702490	1	3	0	云南省曲	50.0	170.0	17.3	36.9

2702377	1	2	1	贵州六盘z	26.5	130.0	15.7	36.5
2699905	1	2	1	云南省楚z	29.0	141.0	14.6	36.5
2699847	1	3	0	云南省保L	72.0	170.0	24.9	36.5
2699653	1	2	1	重庆铜梁	23.0	125.0	14.7	36.5
2699134	1	3	1	云南省红i	63.0	164.0	23.4	36.3
2698587	0	2	0	昭通市镇z	43.0	150.0	19.1	36.5
2698051	1	2	0	云南省临i	50.0	141.0	25.1	36.4
2695146	0	2	1	云南省保L	48.0	155.0	20.0	36.8
2694364	1	2	1	云南省大I	29.0	142.0	14.4	37.5
2692832	1	3	1	云南省丽i	45.0	160.0	17.6	36.7
2690521	1	2	1	云南省昭i	30.0	144.0	14.5	36.5
2690498	1	2	0	云南省楚z	20.0	118.0	14.4	36.8
2690133	1	2	1	昭通市镇z	24.0	125.0	15.4	36.5
2687648	0	2	1	云南省昆明	47.0	150.0	20.9	36.5
2687588	1	2	1	临沧市德3	27.0	132.0	15.5	36.5
2687330	1	3	1	峨山县化z	50.0	168.0	17.7	36.5
2686241	1	3	1	云南省曲i	48.0	161.0	18.5	36.8
2685464	0	3	1	楚雄州姚3	50.0	153.0	21.4	36.5
2684473	0	2	1	昆明市东J	24.0	132.0	13.8	36.5
2683879	0	3	1	昆明市官i	72.0	163.0	27.1	36.7
2683375	0	3	1	昆明市环J	45.0	150.0	20.0	36.6
2682089	0	2	1	云南省昭i	29.0	137.0	15.5	36.5
2681432	0	3	0	云南省楚z	50.0	153.0	21.4	36.5
2681331	0	2	1	贵州省六z	26.0	130.0	15.4	36.7
2681228	0	2	0	云南省昭i	24.0	155.0	10.0	36.5
2680982	0	2	0	云南省玉i	33.0	162.0	12.6	36.5
2680423	1	2	1	保山市昌3	13.0	105.0	11.8	36.5
2680366	0	2	1	云南省曲i	34.0	144.0	16.4	36.6
2679018	1	2	1	昆明市西L	30.0	135.0	16.5	36.5
2678680	1	2	1	贵州省普3	22.5	125.0	14.4	36.5
2678644	1	2	1	云南省曲i	21.0	130.0	12.4	36.2
2678223	0	2	0	云南省石J	30.0	136.0	16.2	36.5
2677436	1	2	0	云南省楚z	34.0	156.0	14.0	36.5
2677066	0	2	1	云南省曲i	36.0	148.0	16.4	36.0
2676612	1	2	1	云南省个I	30.0	145.0	14.3	36.5
2676232	1	2	1	昆明市西L	32.0	148.0	14.6	36.5
2675312	0	2	0	云南省红i	21.0	123.0	13.9	36.5
2675244	1	2	0	云南省大I	28.0	145.0	13.3	36.0
2674667	1	2	0	云南省临i	32.0	132.0	18.4	36.5
2673173	1	2	1	云南省昭i	32.0	145.0	15.2	36.6
2671927	1	2	1	楚雄州大z	61.0	156.0	25.1	36.5
2670638	0	2	0	云南省墨i	32.0	144.0	15.4	36.5
2670384	1	2	1	贵州省威3	30.0	137.0	16.0	36.5
2670314	0	2	0	云南省文L	27.0	128.0	16.5	36.5
2670261	0	1	1	官渡区小z	11.0	90.0	13.6	36.7
2669777	1	2	1	昭通市苏3	31.0	150.0	13.8	36.8
2669058	1	2	1	云南省曲i	33.0	130.0	19.5	36.5
2668289	1	2	0	云南省丽i	32.0	130.0	18.9	36.5
2667600	0	3	1	贵州省大z	45.0	154.0	19.0	36.5
2666225	0	2	1	昆明市官i	21.0	110.0	17.4	36.5
2665734	0	3	1	云南省昭i	42.0	162.0	16.0	36.5
2665429	0	3	1	云南省曲i	44.0	158.0	17.6	36.2
2665338	1	2	1	云南省宣J	36.5	135.0	20.0	35.5
2664979	1	2	0	曲靖市马z	37.0	163.0	13.9	35.5

2664599	0	2	1 云南省玉溪	45.0	130.0	26.6	36.5
2663931	0	3	1 云南省昆明	52.0	165.0	19.1	36.8
2658689	0	3	1 云南省曲靖	40.0	140.0	20.4	36.8
2657960	1	2	1 曲靖市罗平	28.0	127.0	17.4	36.3
2656367	1	3	1 曲靖市富源	32.0	138.0	16.8	36.5
2656175	1	2	0 云南省红河	22.0	140.0	11.2	36.8
2655490	1	3	1 云南省玉溪	52.0	165.0	19.1	36.5
2655024	1	2	1 曲靖市将军	20.0	118.0	14.4	36.5
2652758	1	2	1 云南省昭通	20.0	115.0	15.1	36.2
2649823	0	3	0 辽宁省绥中	41.0	145.0	19.5	36.6
2646137	0	2	1 官渡区五环	12.0	98.0	12.5	36.3
2644677	0	3	1 云南省丽江	45.0	140.0	23.0	36.6
2643736	1	2	1 云南省昭通	32.0	132.0	18.4	36.5
2143579	0	3	1 普洱市景东	30.0	150.0	13.3	36.6
2137521	1	3	1 云南省玉溪	38.0	156.0	15.6	36.5
2133374	0	3	1 云南省保山	40.0	156.0	16.4	36.2
2127706	1	1	1 会泽县新街	9.0	85.0	12.5	36.3
2126387	0	2	1 昭通市镇雄	27.0	142.0	13.4	36.8
2125162	0	3	0 云南省大理	32.0	144.0	15.4	37.0
2124692	1	3	1 景东安定镇	42.0	152.0	18.2	36.0
2120416	0	2	1 通海县纳古	42.0	143.0	20.5	36.3
2116940	1	3	0 云南省江川	50.0	176.0	16.1	37.1
2115719	1	3	1 云南省寻甸	33.0	145.0	15.7	36.3
2109634	0	3	1 宣威得禄乡	39.0	162.0	14.9	36.3
2098999	0	2	0 红河州石屏	31.0	142.0	15.4	37.0
2096357	1	3	1 云南省普洱	42.0	159.0	16.6	36.8
2095833	1	2	1 贵州兴义市	23.0	128.0	14.0	36.9
2092570	1	3	0 红河州泸西	39.0	155.0	16.2	37.8
2090224	0	2	0 楚雄武定县	26.0	146.0	12.2	36.4
2088587	0	3	1 云南省昭通	35.5	146.0	16.7	37.0
2087084	1	3	1 云南省曲靖	30.0	165.0	11.0	36.5
2086196	0	2	1 云南省昆明	37.0	154.0	15.6	39.3
2086187	1	2	0 云南省昆明	15.0	105.0	13.6	36.8
2085593	1	2	0 云南省文山	24.0	115.0	18.1	38.1
2083289	0	3	1 云南省昆明	54.0	157.0	21.9	36.4
2081026	1	2	1 贵州纳雍县	32.0	135.0	17.6	36.5
2080934	1	2	1 曲靖市恩洪	28.0	145.0	13.3	36.8
2080896	1	3	1 曲靖富源县	35.0	167.0	12.5	38.5
2080424	0	2	1 云南省昆明	30.0	137.0	16.0	36.9
2080094	0	3	0 贵州六盘水	50.0	160.0	19.5	36.5
2079988	0	3	1 玉溪市峨山	43.0	164.0	16.0	36.8
2079923	1	2	1 云南省昆明	37.0	154.0	15.6	36.6
2078305	1	2	1 会泽县田坝	40.0	152.0	17.3	36.6
2076402	0	3	1 普洱市景东	32.0	140.0	16.3	36.5
2075728	0	2	1 曲靖师宗彩	23.0	130.0	13.6	36.8
2074108	1	2	0 楚雄大姚金	32.0	142.0	15.9	36.8
2073876	0	2	1 昭通大关县	35.0	150.0	15.6	36.8
2072603	0	2	0 红河石屏县	36.0	147.0	16.7	37.0
2071484	0	2	1 云南省保山	21.0	126.0	13.2	37.0
2070779	0	3	0 玉溪新平县	57.0	158.0	22.8	36.8
2070514	0	3	1 云南省昆明	54.0	160.0	21.1	36.4
2070069	1	3	1 云南省保山	37.0	159.0	14.6	36.8
2069940	1	3	1 云南省玉溪	45.0	155.0	18.7	37.2
2068675	1	2	1 云南省昆明	60.0	158.0	24.0	36.5

2068117	0	2	0	贵州省盘县	20.0	110.0	16.5	37.0
2067904	1	2	0	云南省临沧	43.0	158.0	17.2	36.8
2067025	1	2	1	曲靖富源县	25.0	130.0	14.8	36.7
2066305	0	3	1	富源县黄泥	46.0	152.0	19.9	36.8
2066007	1	2	1	楚雄鹿城	30.0	139.0	15.5	36.5
2065788	1	2	0	云南省临沧	15.0	112.0	12.0	36.5
2065596	1	2	1	昆明市海	19.0	121.0	13.0	36.5
2064315	1	2	1	昭通镇雄	21.0	135.0	11.5	36.9
2064235	1	2	1	大理州祥	38.0	145.0	18.1	36.8
2063687	1	2	1	昭通市巧	25.0	128.0	15.3	36.6
2062164	0	3	0	峨山县双	44.0	152.0	19.0	35.9
2061505	1	2	1	五华区红	32.0	154.0	13.5	36.0
2060219	1	2	1	云南省昆	16.0	100.0	16.0	36.6
2059726	1	2	1	楚雄州牟	32.0	162.0	12.2	36.8
2058040	1	3	0	红河州泸	34.0	153.0	14.5	36.8
2057070	1	2	1	贵州六盘	48.0	168.0	17.0	36.8
2057007	1	2	1	云南省楚	36.0	140.0	18.4	36.6
2056840	1	2	0	云南省昆	22.0	132.0	12.6	36.5
2055724	1	2	0	贵州兴义	23.5	135.0	12.9	36.9
2055535	0	2	1	云南省昆	43.0	147.0	19.9	36.6
2055488	1	2	0	云南省德	29.0	145.0	13.8	36.4
2055262	0	2	1	保山市昌	35.0	158.0	14.0	36.4
2053181	0	2	1	云南省昆	40.0	152.0	17.3	36.5
2050422	0	2	1	云南省保	36.0	159.0	14.2	36.5
2050365	1	2	0	曲靖会泽	36.0	150.0	16.0	36.4
2048613	1	2	0	香格里拉	33.0	145.0	15.7	37.3
2048151	1	2	1	云南省普	42.0	152.0	18.2	37.0
2046953	0	3	1	德宏芒市	32.0	168.0	11.3	37.3
2046107	0	2	1	菊花村26	28.0	145.0	13.3	36.8
2043911	1	2	1	昭通镇雄	26.0	135.0	14.3	36.5
2043545	0	2	1	教场西路1	44.0	156.0	18.1	36.7
2042756	0	2	1	会泽县娜	33.0	130.0	19.5	36.4
2042319	0	2	1	曲靖市富	33.0	145.0	15.7	36.8
2041927	1	3	1	富源县后	35.0	165.0	12.9	36.4
2037037	0	2	1	昭通巧家	26.0	128.0	15.9	36.8
2032200	0	2	1	杨利*嵩明	33.0	155.0	13.7	36.6
2028510	0	2	0	个旧市沙	12.0	95.0	13.3	36.6
2027365	0	3	1	呈贡县马	52.0	149.0	23.4	36.5
2026565	1	3	1	蒙自瑞凤	44.0	158.0	17.6	36.5
2019722	1	2	1	新亚洲体	43.0	150.0	19.1	36.5
2019451	1	2	0	临沧市永	30.0	161.0	11.6	36.2
2016258	1	2	1	富源大栗	29.8	145.0	14.2	36.5
2014220	1	2	1	春苑小区	29.0	137.0	15.5	36.8
2014198	1	3	1	武定县已	34.0	155.0	14.2	36.9
2013466	1	2	1	保山腾冲	25.0	122.0	16.8	36.1
2013412	0	3	0	楚雄永仁	38.0	162.0	14.5	36.1
2011602	1	3	1	祥云县沙	40.0	159.0	15.8	36.5
2010514	1	2	1	保山施甸	40.0	151.0	17.5	36.8
2010512	1	2	1	保山施甸	50.0	153.0	21.4	36.5
2009361	1	2	1	剑川县金	38.5	150.0	17.1	36.6
2006834	0	2	1	东风东路	25.0	145.0	11.9	36.5
2006600	0	2	1	小石坝东	40.0	156.0	16.4	36.6
2006194	1	2	1	镇雄县陈	50.0	165.0	18.4	36.5
2004270	1	2	1	昆明市农	25.0	145.0	11.9	36.5

2002531	0	2	1 大树营中	22.0	145.0	10.5	36.4
2002210	0	2	1 昌宁县紫	35.0	156.0	14.4	36.6
2001923	1	2	1 禄劝县撒	29.5	137.0	15.7	37.8
2001318	0	3	0 普洱市威	34.0	148.0	15.5	36.5
2000670	0	2	1 红河建水	40.0	155.0	16.6	36.5
1345332	0	2	1 世纪城元	27.5	145.0	13.1	36.5
1342116	1	3	1 晋宁县有	40.0	160.0	15.6	36.7
2814662	1	3	1 宣威市格	40.0	168.0	14.2	36.2
2817361	1	2	1 贵州省盘	16.0	110.0	13.2	36.8
2817721	0	2	1 腾冲县北	26.0	130.4	15.3	36.6
2818450	1	3	1 贵州省水	50.0	167.0	17.9	36.8
2818662	0	3	1 云南省红	42.0	152.0	18.2	36.8
2820689	1	2	1 云南省玉	28.0	140.0	14.3	36.5
2820758	0	2	1 云南省曲	23.0	130.0	13.6	36.8
2820279	0	2	0 云南省文	20.0	115.0	15.1	36.3
2820651	1	2	1 昆明市西	51.0	152.0	22.1	36.3
2821929	1	3	1 福建省福	54.0	153.0	23.1	36.7
2822140	0	3	1 昆明市官	46.0	155.0	19.1	36.4
2822882	1	3	1 迪庆州维	39.0	165.0	14.3	37.3
2820722	0	3	1 楚雄州禄	38.0	155.0	15.8	36.8
2824944	0	2	1 云南省昆	13.5	102.0	13.0	36.7
2825111	1	2	1 创意英国	47.0	171.0	16.1	36.5
2826303	1	2	0 丽江市古	34.0	144.0	16.4	36.8
2827739	0	3	0 大理白族	45.0	160.0	17.6	38.8
2828855	1	2	1 昆明市五	45.5	151.0	20.0	36.5
2827708	1	2	1 楚雄彝族	35.0	146.0	16.4	36.7
2829721	1	3	1 云南省红	38.0	164.0	14.1	36.7
2830496	0	2	0 个旧市鸡	18.0	105.0	16.3	36.2
2830866	1	2	1 云南省军	23.0	123.0	15.2	36.6
2832947	1	3	1 昆明市北	45.0	155.0	18.7	36.8
2829044	0	2	0 大理祥云	35.0	152.0	15.1	37.0
2837548	0	2	1 昆明市官	25.0	140.0	12.8	36.7
2838187	1	2	1 楚雄市禄	31.0	147.0	14.3	36.5
2836417	0	2	0 文山州广	22.0	158.0	8.8	36.5
2837368	1	2	0 昆明市五	33.0	140.0	16.8	36.6
2839233	1	2	1 昆明市呈	56.0	152.0	24.2	36.7
2838623	1	2	1 昆明市王	48.0	163.0	18.1	36.5
2842062	0	2	1 昆明市官	48.0	135.0	26.3	36.6
2841156	1	2	1 昆明市五	29.0	135.0	15.9	36.8
2845049	1	2	0 迪庆藏族	26.0	130.0	15.4	36.2
2843028	1	2	1 昆明市西	25.0	138.0	13.1	36.6
2843805	1	2	1 文山壮族	24.0	120.0	16.7	37.1
2845561	1	2	1 德宏州芒	42.0	161.0	16.2	36.5
2845365	1	2	1 昭通市盐	38.0	157.0	15.4	36.5
2846791	0	2	1 云南省昆	30.0	125.0	19.2	36.6
2848101	1	2	1 昆明市西	50.0	163.0	18.8	36.6
2848750	0	2	1 昆明市五	18.0	116.0	13.4	36.4
2849528	1	3	1 保山市隆	34.0	149.0	15.3	36.5
2849646	1	2	1 昆明市禄	41.0	146.0	19.2	36.6
2850284	1	2	1 文山市广	43.0	163.0	16.2	36.9
2849540	1	2	1 师宗县雄	24.0	130.0	14.2	36.3
2850857	1	2	0 红河州屏	25.0	130.0	14.8	36.7
2851330	0	3	1 宣威市文	35.0	148.0	16.0	36.5
2880243	0	2	0 昆明市西	27.0	115.0	20.4	36.2

2881107	0	3	1	云南省曲	45.0	153.0	19.2	36.5
2881816	1	2	1	昆明市盘	28.5	143.0	13.9	36.0
2882688	0	3	1	昆明市官	36.5	162.0	13.9	36.4
2882088	1	2	1	云南省普	32.0	145.0	15.2	36.6
2855513	0	2	1	曲靖市陆	22.0	132.0	12.6	36.6
2856135	0	2	1	贵州省兴	22.0	114.0	16.9	36.8
2856084	1	2	1	大理南涧	34.0	139.0	17.6	36.5
2856471	0	2	1	曲靖市会	27.0	130.0	16.0	36.5
2857181	1	3	1	云南省丽	53.0	153.0	22.6	36.5
2856834	1	2	1	临沧市临	42.0	155.0	17.5	36.7
2857244	0	2	1	曲靖市会	20.0	112.0	15.9	36.5
2857787	1	2	0	临沧市云	25.0	130.0	14.8	36.7
2858638	0	2	1	云南省昭	32.5	154.0	13.7	36.5
2858151	1	2	1	贵州省盘	34.0	140.0	17.3	36.4
2858942	1	2	1	云南曲靖	51.0	160.0	19.9	36.7
2858202	1	2	1	云南省曲	62.5	160.0	24.4	36.5
2860384	0	2	1	临沧市云	32.0	128.0	19.5	36.0
2860898	1	3	1	云南省红	29.0	135.0	15.9	36.2
2863538	1	2	1	云南省红	30.0	130.0	17.8	36.3
2863908	1	2	1	云南省昆	33.0	141.0	16.6	36.6
2861043	0	2	1	云南省石	21.5	120.0	14.9	36.3
2863708	0	3	1	红河州建	64.0	154.0	27.0	36.5
2865007	1	2	1	宣威市热	16.5	115.0	12.5	36.8
2865774	0	3	1	云南省文	38.0	153.0	16.2	36.8
2865295	0	3	1	云南省昆	30.0	136.0	16.2	36.3
2865473	1	3	1	云南省玉	54.0	167.0	19.4	36.5
2865321	0	2	1	镇雄县	35.0	140.0	17.9	36.2
2866157	1	2	1	云南省曲	35.0	150.0	15.6	36.6
2867197	1	3	0	云南大理	54.0	179.0	16.9	36.8
2863807	1	3	1	云南省玉	40.0	157.0	16.2	36.2
2868104	1	2	1	云南省红	27.0	125.0	17.3	36.8
2970412	0	2	1	云南省曲	26.0	130.0	15.4	36.8
2869130	1	2	0	云南省普	21.0	118.0	15.1	38.2
2869462	1	2	1	普洱市镇	24.0	120.0	16.7	36.6
2869977	1	3	1	曲靖市会	60.0	158.0	24.0	36.5
2873519	1	3	1	曲靖市会	54.0	165.0	19.8	36.6
2970525	1	3	1	通海县秀	42.0	154.0	17.7	38.5
2873606	1	2	1	云南省曲	42.0	143.0	20.5	36.6
2874738	0	2	1	昆明市西	31.0	152.0	13.4	36.5
2877023	0	3	0	云南省安	50.0	172.0	16.9	36.3
2876827	0	2	1	临沧市镇	24.0	119.0	16.9	36.6
2877936	0	2	1	昭通市镇	42.0	150.0	18.7	36.2
2878550	1	3	1	云南省楚	49.0	165.0	18.0	36.7
2887287	0	2	1	曲靖市陆	27.0	137.0	14.4	36.7
2888140	1	3	1	福建省晋	65.0	176.0	21.0	37.0
2890547	1	2	0	云南省玉	29.0	153.0	12.4	36.8
2889138	0	2	0	五华区棕	21.0	113.0	16.4	37.2
2878499	0	2	1	云南省楚	25.0	133.0	14.1	36.5
2893381	0	2	0	云南省文	30.0	135.0	16.5	36.2
2893457	1	2	1	昭通市镇	45.0	165.0	16.5	36.5
2894568	1	2	1	宣威市	78.0	172.0	26.4	36.9
2895787	0	2	1	云南省昭	18.0	116.0	13.4	36.7
2895812	0	2	1	云南省昆	24.0	127.0	14.9	36.7
2896868	0	2	1	宣威市乐	37.5	150.0	16.7	36.2

2898046	0	2	1	临沧市云县	41.0	151.0	18.0	36.6
2896399	0	3	0	大理祥云县	45.0	160.0	17.6	36.6
2899506	1	2	0	云南省怒江	18.0	115.0	13.6	36.8
2899536	1	2	0	普洱市墨江	22.0	146.0	10.3	36.7
2900672	0	2	1	昆明市五华	22.0	120.0	15.3	36.7
2900664	0	3	0	楚雄彝族自	45.0	156.0	18.5	36.0
2872860	1	3	1	临沧市永德	78.0	167.0	28.0	36.9
2902583	1	3	1	盘龙区环城	45.0	146.0	21.1	36.6
2872817	0	3	1	昭通市镇雄	40.0	160.0	15.6	36.9
2901798	1	3	0	云南省普洱	40.0	150.0	17.8	36.6
2903821	1	2	1	晋宁县晋城	24.0	121.0	16.4	36.8
2903885	1	2	0	红河州金平	24.0	118.0	17.2	36.5
2904075	1	2	1	云南省曲靖	34.0	148.0	15.5	36.6
2905471	0	2	1	昆明市富民	50.0	160.0	19.5	36.7
2906271	1	2	1	云南省红河	25.0	125.0	16.0	36.0
2906276	1	2	1	云南省楚雄	28.0	140.0	14.3	37.1
2906279	1	3	0	云南省大理	57.0	163.0	21.5	37.1
2907142	1	2	1	云南省曲靖	24.0	135.0	13.2	36.8
2906919	0	2	1	云南省昆明	64.0	157.0	26.0	36.8
2909676	0	2	1	云南省昆明	27.5	142.0	13.6	36.7
2909290	1	2	1	云南省昆明	43.0	151.0	18.9	36.8
2910956	0	3	1	云南省昆明	47.0	152.0	20.3	37.5
2911985	1	2	0	云南省昆明	19.0	120.0	13.2	36.5
2912137	1	3	1	云南省曲靖	36.0	150.0	16.0	36.6
2911544	0	2	1	云南省昭通	36.0	155.0	15.0	36.4
2911776	1	2	1	云南省昆明	68.5	164.0	25.5	36.5
2912552	1	2	0	云南省普洱	21.0	120.0	14.6	36.5
2913254	0	2	1	云南省曲靖	22.5	129.0	13.5	36.7
2914490	1	2	1	云南省曲靖	40.0	130.0	23.7	36.8
2914091	0	3	1	云南省临沧	53.0	161.0	20.4	36.5
2915092	1	2	1	云南省红河	31.0	144.0	14.9	36.7
2916335	1	2	1	云南省曲靖	27.0	130.0	16.0	36.5
2914221	0	3	1		47.5	158.0	19.0	36.6
2915603	1	3	0	云南省昭通	36.0	155.0	15.0	36.3
2917965	1	2	1	云南省曲靖	35.0	135.0	19.2	36.5
2915766	0	3	1	云南省昆明	46.0	158.0	18.4	36.5
2918272	1	2	0	云南省大理	32.0	135.0	17.6	36.8
2919459	0	3	0	云南省昆明	48.0	168.0	17.0	36.3
2919485	1	2	1	云南省普洱	29.0	135.0	15.9	36.3
2921286	0	2	1	泸西多衣村	40.0	138.0	21.0	36.3
2921038	0	2	1	云南省昆明	45.0	162.0	17.1	36.9
2920183	1	2	0	云南省楚雄	32.0	144.0	15.4	36.3
2921284	0	2	1	云南省楚雄	32.0	141.0	16.1	36.6
2921240	1	2	1	云南省昆明	45.0	155.0	18.7	36.0
2921621	1	3	1	云南省昆明	51.0	175.0	16.7	36.6
2922130	1	3	0	云南省丽江	55.0	175.0	18.0	36.7
2922715	1	3	1	云南省昆明	55.0	174.0	18.2	36.3
2923098	1	3	1	云南省昆明	53.0	170.0	18.3	36.0
2923163	1	2	0	昆明市寻甸	30.0	132.0	17.2	36.5
2921744	1	2	1	云南省曲靖	32.0	144.0	15.4	36.8
2921878	0	2	0	昆明市民航	26.0	138.0	13.7	36.5
2925029	1	2	1	保山昌宁县	36.0	148.0	16.4	36.5
2925196	1	2	0	云南省昭通	22.0	117.0	16.1	36.9
2925206	0	2	1	云南省曲靖	27.0	135.0	14.8	36.5

2925377	1	2	1	会泽县大	31.0	142.0	15.4	36.8
2925705	1	2	0	云南省昆	28.5	137.0	15.2	36.7
2919116	0	2	1	云南省玉	34.0	153.0	14.5	38.0
2926038	0	3	0	云南省德	65.0	161.0	25.1	36.4
2927632	0	3	0	云南省临	35.0	155.0	14.6	36.2
2927724	1	2	1	云南省玉	56.0	142.0	27.8	37.0
2928235	0	3	1	云南省昆	41.0	153.0	17.5	36.6
2918762	1	2	1	云南省曲	36.0	154.0	15.2	36.7
2891012	1	2	1	昆明市晋	51.0	154.0	21.5	37.0
2931358	1	3	1	云南省临	25.0	135.0	13.7	36.8
2931205	1	2	0	临沧市云	28.0	143.0	13.7	36.8
2931184	0	2	0		35.0	135.0	19.2	36.2
2931436	0	2	0	云南省德	27.0	138.0	14.2	36.9
2931536	1	2	1	云南省昆	21.0	115.0	15.9	36.8
2932354	1	2	1	云南省昆	22.0	125.0	14.1	36.5
2931667	0	2	0	云南省临	17.3	115.0	13.1	36.8
2931579	1	2	0	云南省昆	32.0	105.0	29.0	36.6
2887980	1	2	1	昆明市禄	21.0	126.0	13.2	36.5
2932215	0	2	1	云南省曲	23.0	125.0	14.7	36.7
2932426	1	2	1	腾冲市	16.0	105.0	14.5	36.3
2931868	0	3	0	云南省昆	32.0	168.0	11.3	36.5
2930579	1	3	1	云南省昭	53.0	172.0	17.9	36.5
2932723	0	3	1	云南省昭	51.5	165.0	18.9	36.8
2934006	0	2	0	云南省西	74.0	158.0	29.6	36.8
2932618	0	3	1	昆明市西	28.0	134.0	15.6	36.5
2933719	0	2	1	昭通市镇	23.0	131.0	13.4	36.5
2933802	1	2	1	昭通市镇	39.0	150.0	17.3	36.6
2934204	1	3	0	德宏盈江	44.0	161.0	17.0	36.8
2935151	0	2	0	云南省丽	29.0	125.0	18.6	36.0
2935311	1	2	1	云南省昭	26.0	130.0	15.4	36.4
2936349	0	3	1	宣威市	60.0	165.0	22.0	36.9
2934313	1	3	1	曲靖市陆	52.0	155.0	21.6	36.3
2936109	0	2	1	昆明市呈	30.0	130.0	17.8	36.4
2936100	1	2	1	曲靖市富	33.0	140.0	16.8	36.8
2936223	0	3	1	曲靖市师	43.0	159.0	17.0	36.4
2937974	1	2	0	昆明市盘	28.0	135.0	15.4	36.2
2938955	1	2	1	云南省昭	23.0	120.0	16.0	36.9
2940445	1	2	1	楚雄州大	45.0	140.0	23.0	36.8
2941427	1	3	1	云南省普	42.0	155.0	17.5	36.7
2943069	0	2	1	云南省昆	14.0	101.0	13.7	36.2
2943231	1	2	1	昆明市五	18.0	110.0	14.9	36.5
2946329	1	2	1	昭通市镇	38.0	140.0	19.4	36.3
2946757	1	2	1	临沧市凤	34.0	141.0	17.1	36.8
2946636	0	2	1	曲靖市陆	29.0	130.0	17.2	36.1
2947178	1	2	1	贵州省毕	20.0	125.0	12.8	36.5
2947139	1	2	1	曲靖市会	41.0	130.0	24.3	36.5
2947408	0	2	1		30.0	130.0	17.8	36.1
2947415	1	2	1	云南省曲	39.0	150.0	17.3	36.3
2948131	1	2	1	玉溪市峨	64.0	161.0	24.7	36.2
2948613	0	3	0	云南省临	55.0	160.0	21.5	36.8
2948253	1	2	1	云南省昭	21.0	121.0	14.3	36.8
2948827	1	2	1	曲靖市宣	32.0	143.0	15.6	36.2
2951004	1	3	1	云南省楚	35.0	145.0	16.6	36.3
2950338	1	2	0	云南省临	45.0	153.0	19.2	36.6

2951675	1	2	1 云南省大玛	42.0	160.0	16.4	36.2
2951335	0	3	1 昆明市嵩明	47.0	170.0	16.3	36.0
2952632	1	2	1 昆明市五华	43.0	145.0	20.5	36.0
2954202	1	2	1 云南省昆明	50.0	167.0	17.9	36.3
2954252	1	3	1 云南省大玛	40.0	160.0	15.6	36.8
2955147	1	3	1 曲靖市会泽	43.0	150.0	19.1	36.8
2929555	1	2	1 云南省昆明	70.0	168.0	24.8	36.6
2954835	0	3	1 曲靖市会泽	43.0	158.0	17.2	36.0
2873606	1	2	1 曲靖市宣威	45.0	152.0	19.5	36.5
2955528	1	2	1 昭通市镇雄	37.0	144.0	17.8	36.5
2956255	0	2	1 昆明市盘龙	33.0	135.0	18.1	36.6
2957837	0	2	0 云南省临沧	35.0	150.0	15.6	36.9
2957572	1	3	1 昆明市富民	42.0	150.0	18.7	36.8
2956823	1	3	0 云南省楚雄	59.0	168.0	20.9	36.5
2958211	1	2	0 云南省文山	29.0	140.0	14.8	36.8
2958385	1	3	1 贵州省盘县	43.0	156.0	17.7	36.8
2957690	1	2	1 贵州省毕节	34.0	147.0	15.7	36.8
2956716	0	3	1 云南省昆明	47.0	158.0	18.8	37.0
2958479	1	3	1 五华区昆明	125.0	183.0	37.3	36.6
2960109	1	2	1 昭通市南beh	45.5	155.0	18.9	36.3
2960795	1	3	0 云南省红河	45.0	160.0	17.6	36.7
2957490	1	2	1 曲靖市师宗	49.0	150.0	21.8	36.6
2954449	1	2	1 玉溪市通海	55.0	156.0	22.6	38.4
2962682	1	3	1 昆明市五华	38.0	151.0	16.7	36.5
2962168	0	2	1 昆明市五华	25.0	138.0	13.1	36.6
2963924	0	2	1 曲靖市会泽	26.0	125.0	16.6	36.5
2965877	1	2	1 曲靖市罗平	42.0	162.0	16.0	36.7
2966319	0	2	0 云南省大玛	25.0	130.0	14.8	36.2
2967127	0	2	1 曲靖市会泽	44.0	145.0	20.9	36.5
2964909	0	2	1 云南省昆明	32.0	153.0	13.7	36.4
2964160	1	2	0 云南省昭通	35.0	145.0	16.6	36.2
2967852	1	2	1 云南省楚雄	32.0	145.0	15.2	36.3
2968212	0	2	0 云南省大玛	20.0	127.0	12.4	36.6
2966243	0	2	0 云南省建水	28.0	140.0	14.3	35.4
2968257	0	2	0 云南省西山	34.0	135.0	18.7	36.2
2968515	0	2	1 昆明市盘龙	16.0	115.0	12.1	36.8
2969117	0	2	1 云南省昆明	14.0	103.0	13.2	36.5
2968849	0	2	0 云南省昆明	42.0	157.0	17.0	36.2
2969028	1	2	1 云南省昆明	44.0	145.0	20.9	36.3
3000322	0	3	1 昭通市彝良	48.0	152.0	20.8	36.6
2968844	0	2	0 云南省昆明	26.0	137.0	13.9	36.5
2968746	1	3	0 四川省甘孜	37.0	140.0	18.9	36.6
3001630	1	3	0 云南省丽江	51.0	175.0	16.7	36.5
3003968	1	2	1 云南省昭通	35.0	145.0	16.6	36.5
3005591	1	2	1 曲靖市会泽	36.0	140.0	18.4	36.5
3006656	1	2	1 云南省大玛	36.0	135.0	19.8	36.8
3008056	1	2	1 曲靖市罗平	21.0	140.0	10.7	36.2
3008506	0	3	1 云南省文山	48.0	155.0	20.0	36.7
3008660	0	3	1 云南省大玛	46.0	155.0	19.1	36.5
2956149	0	3	1 云南省红河	62.0	163.0	23.3	36.3
3009359	0	2	1 云南省昭通	22.0	130.0	13.0	36.4
3009914	1	2	1 云南省昆明	23.0	135.0	12.6	36.5
3010313	1	2	1 昆明市盘龙	25.0	135.0	13.7	36.4
3011972	0	2	1 云南省昆明	31.0	148.0	14.2	36.7

3008696	0	3	1 云南省文	51.0	158.0	20.4	36.4
3012437	1	2	1 云南省昭	28.0	140.0	14.3	36.4
3013245	0	2	1 贵州省六	20.0	120.0	13.9	36.5
3017227	0	2	0 云南省怒	30.0	130.0	17.8	36.6
3018051	1	1	0 文山州富	15.0	95.0	16.6	36.5
3020758	0	2	1 云南省昆	19.0	107.0	16.6	36.8
3022015	1	2	0 云南省怒	54.0	148.0	24.7	36.5
3023720	0	3	0 云南省临	72.0	160.0	28.1	36.5
2915928	1	3	1 云南省红	96.0	170.0	33.2	36.5
3025844	1	3	1 云南省昆	64.0	168.0	22.7	36.3
3026712	0	2	0 云南省景	38.0	148.0	17.3	36.1
3026526	1	2	1 楚雄大姚	28.0	135.0	15.4	36.2
3028001	0	3	1 云南省昆	42.0	160.0	16.4	36.5
3028183	1	2	1 曲靖市宣	62.0	155.0	25.8	36.4
3027664	0	2	1 云南省昆	25.0	125.0	16.0	36.5
3028963	0	2	1 云南省昭	53.0	156.0	21.8	36.4
3029824	1	3	1 曲靖市会	44.0	160.0	17.2	36.2
3030303	0	2	1 云南省昆	23.0	127.0	14.3	36.6
2951461	1	2	1 云南省昭	34.0	129.0	20.4	36.6
3031445	1	2	1 云南省昭	30.0	140.0	15.3	36.8
3031375	1	2	0 文山州丘	25.0	130.0	14.8	36.6
3032144	0	3	1 云南省丽	50.0	160.0	19.5	36.5
3026344	1	3	1 云南省昆	46.0	160.0	18.0	36.3
3033482	0	3	1 云南省昭	81.0	152.0	35.1	36.8
3033988	1	2	1 云南省昆	40.0	130.0	23.7	36.2
3034276	1	2	1 贵州省毕	35.0	140.0	17.9	36.8
3036440	1	2	1 楚雄姚安	53.0	158.0	21.2	37.1
3018524	1	2	1 云南省昆	28.0	140.0	14.3	36.4
3036850	0	2	0 云南省昆	24.0	124.0	15.6	36.5
3028873	0	2	1 云南省临	22.0	125.0	14.1	36.5
3037767	0	3	1 云南省昆	48.0	158.0	19.2	36.2
3037327	0	2	1 昭通市镇	33.0	145.0	15.7	36.5
3037438	1	3	0 云南省丽	48.0	168.0	17.0	36.3
3037601	1	2	1 云南省昆	33.0	141.0	16.6	36.6
3038133	1	3	0 云南省丽	49.0	163.0	18.4	36.7
3038603	1	2	0 贵州省黔	21.0	120.0	14.6	36.3
3038556	1	2	0 云南省昆	43.0	150.0	19.1	36.5
3037574	1	2	1 云南省楚	23.0	135.0	12.6	36.2
3039188	0	2	1 云南省昆	29.0	140.0	14.8	36.3
3038903	1	3	1 曲靖市会	43.0	155.0	17.9	36.3
3038921	0	3	0 云南省昆	43.0	155.0	17.9	36.5
3039666	1	3	1 云南省红	39.0	161.0	15.0	36.5
3039502	0	2	1 云南省昭	25.5	135.0	14.0	36.3
3040001	0	3	0 云南省红	62.0	154.0	26.1	36.4
3040173	1	2	0 云南省临	54.0	158.0	21.6	36.4
2943156	0	2	1 云南省昭	26.0	124.0	16.9	36.9
3043045	0	2	1 云南省昆	46.0	150.0	20.4	36.5
3043889	1	2	1 云南省红	30.0	145.0	14.3	36.5
3044443	1	2	1 曲靖市会	35.0	140.0	17.9	36.5
3024150	0	3	0 曲靖市会	45.0	150.0	20.0	39.3
3045898	0	2	1 曲靖市陆	22.5	126.0	14.2	36.2
3045216	0	2	1 云南省玉	45.0	157.0	18.3	36.2
3042581	0	2	1 昆明市五	29.0	129.0	17.4	36.5
3045225	0	2	1 昆明市五	25.0	130.0	14.8	36.5

3046620	1	2	1 贵州省贵阝	37.0	147.0	17.1	36.5
3046295	0	2	1 云南省昭通	26.0	112.0	20.7	36.8
3047149	0	2	1 云南省保山	40.0	152.0	17.3	36.5
3047985	1	3	1 云南省昆明	58.0	167.0	20.8	36.5
3048408	1	2	0 云南省红河	37.0	145.0	17.6	36.8
3048885	0	2	0 云南省昆明	25.0	135.0	13.7	36.4
3049629	0	2	0 云南省丽江	31.0	139.0	16.0	36.6
3050150	0	3	1 云南省昭通	48.0	155.0	20.0	36.4
3049267	0	2	1 云南省昆明	31.0	145.0	14.7	36.2
3051031	1	2	1 云南省曲靖	14.2	100.0	14.2	36.3
3051313	0	3	1 云南省昆明	58.0	165.0	21.3	36.3
3051376	0	2	0 云南省楚雄	27.0	135.0	14.8	36.0
3052720	1	2	1 云南省曲靖	33.0	145.0	15.7	36.3
3053239	0	2	1 云南省昆明	30.0	138.0	15.8	36.5
3053341	1	2	1 云南省楚雄	53.0	146.0	24.9	36.4
3053196	1	2	1 云南省昆明	17.0	109.0	14.3	36.5
3054785	1	3	0 云南省大理	65.0	162.0	24.8	36.5
3053038	0	2	1 云南省大理	38.0	140.0	19.4	36.2
3056326	0	2	1 云南省昆明	40.0	156.0	16.4	36.7
3057839	1	2	0 云南省昆明	33.0	140.0	16.8	36.8
3058894	1	2	1 云南省大理	42.0	151.0	18.4	36.3
3059475	0	2	1 云南省昆明	36.0	150.0	16.0	36.5
3059829	1	2	1 云南省昆明	28.0	141.0	14.1	36.2
3060255	0	2	1 云南省昆明	30.0	148.0	13.7	36.2
3060698	1	3	1 云南省保山	64.0	157.0	26.0	36.2
3060709	1	3	1 云南省文山	60.0	168.0	21.3	37.1
3061863	0	2	0 云南省保山	55.0	160.0	21.5	36.3
3060745	0	2	1 云南省昭通	42.0	150.0	18.7	36.1
3061786	1	2	1 云南省昆明	31.0	146.0	14.5	36.2
3062237	1	2	1 云南省昆明	21.0	128.0	12.8	36.5
3043797	0	3	1 云南省曲靖	27.0	145.0	12.8	36.6
3067244	0	2	0 云南省丽江	27.5	142.0	13.6	36.1
3068629	0	2	1 云南省昆明	29.0	143.0	14.2	36.5
3069634	0	2	1 云南省大理	27.0	138.0	14.2	36.8
3069825	1	2	1 云南省昆明	45.0	136.0	24.3	36.2
3069139	1	3	1 云南省西双	63.0	175.0	20.6	36.5
3070111	1	2	1 云南省曲靖	24.0	110.0	19.8	36.8
3068626	1	3	1 云南省保山	47.0	153.0	20.1	36.3
3069396	1	2	0 云南省大理	25.0	126.0	15.7	36.5
3070974	0	3	1 四川省自贡	54.0	157.0	21.9	36.3
3072730	0	2	1 云南省昆明	53.0	160.0	20.7	36.3
3076882	1	2	1 云南省曲靖	21.0	119.0	14.8	36.2
3078023	0	2	0 云南省大理	46.0	145.0	21.9	36.3
3077892	0	2	0 云南省大理	52.0	165.0	19.1	36.4
3077941	1	2	1 云南省楚雄	23.0	119.0	16.2	36.8
3080138	1	2	1 云南省临沧	68.0	158.0	27.2	36.6
3081299	1	2	1 贵州省水城	75.0	148.0	34.2	36.1
3081470	0	3	0 云南省普洱	58.0	163.0	21.8	36.5
3080630	1	2	1 云南省昆明	66.0	161.0	25.5	36.4
3082111	0	3	0 云南省怒江	47.5	155.0	19.8	36.0
3082084	1	2	1 贵州省黔西	55.0	170.0	19.0	36.9
3082074	0	2	1 新疆维吾尔	58.0	163.0	21.8	36.5
3082300	1	2	1 贵州省毕节	36.0	150.0	16.0	36.6
3084942	0	2	1 云南省文山	29.0	130.0	17.2	36.5

3084725	1	2	0	云南省临沧	32.0	144.0	15.4	36.1
3085408	0	2	0	云南省楚雄	32.0	145.0	15.2	36.4
3085625	1	2	0	云南省大理	17.0	117.0	12.4	36.3
3088183	1	1	0	云南省昆明	14.0	96.0	15.2	36.5
3089143	0	2	1	云南省曲靖	40.0	160.0	15.6	36.5
3092553	1	3	1	云南省曲靖	34.0	150.0	15.1	36.3
3094275	1	2	0	云南省文山	24.0	120.0	16.7	36.3
3093170	1	3	1	云南省曲靖	82.0	175.0	26.8	36.3
3095263	0	2	1	云南省昆明	20.0	117.0	14.6	36.3
3095102	1	3	1	云南省曲靖	68.0	158.0	27.2	36.5
3096486	0	2	1	云南省昆明	47.0	159.0	18.6	36.1
3096288	1	2	1	云南省曲靖	45.0	145.0	21.4	36.2
3096596	1	3	0	云南省楚雄	33.0	156.0	13.6	36.3
3096363	1	3	1	云南省德宏	47.0	165.0	17.3	36.4
3097342	1	2	1	云南省临沧	36.0	130.0	21.3	36.4
3098724	1	2	0	丽江市永胜	32.5	140.0	16.6	36.5
3098585	1	3	1	云南省保山	45.0	165.0	16.5	36.3
3100382	1	2	1	昆明市西山	41.0	155.0	17.1	36.5
3100978	0	2	1	曲靖市宣威	44.0	155.0	18.3	36.2
3100903	0	2	0	云南省临沧	35.0	142.0	17.4	36.5
3102623	1	3	1	云南省临沧	37.5	158.0	15.0	36.1
3102559	1	2	1	云南省丽江	101.0	170.0	34.9	36.4
3102949	1	3	1	曲靖市会泽	40.0	160.0	15.6	36.4
3103442	1	2	1	云南省临沧	35.0	142.0	17.4	36.3
3103412	1	2	1	云南省楚雄	26.0	128.0	15.9	36.2
3104767	0	3	1	云南省楚雄	60.0	164.0	22.3	36.6
3105586	1	2	0	云南省临沧	29.0	130.0	17.2	36.5
3106969	0	2	1	云南省临沧	16.0	117.0	11.7	36.3
3108463	1	3	0	云南省宣威	51.0	155.0	21.2	36.4
3107073	0	2	1	云南省昆明	35.0	147.0	16.2	36.5
3108857	0	2	1	四川省崇州	28.0	136.0	15.1	36.3
3108888	0	2	0	云南省临沧	36.0	145.0	17.1	36.4
3109323	0	2	1	昆明市金宁	26.0	126.0	16.4	36.5
3109694	0	2	0	云南省临沧	29.0	146.0	13.6	36.5
3111700	1	2	1	曲靖市会泽	34.0	131.0	19.8	36.5
3110929	0	2	1	曲靖市马龙	18.0	106.0	16.0	36.0
3112483	1	2	1	曲靖市会泽	29.0	115.0	21.9	36.5
3112311	0	2	1	曲靖市会泽	28.0	130.0	16.6	36.0
3115195	0	2	1	云南省大理	30.0	138.0	15.8	36.5
3118218	1	2	1	云南省德宏	36.0	143.0	17.6	36.5
3119737	0	3	1	云南省曲靖	50.0	160.0	19.5	36.5
3064802	0	2	1	云南省玉溪	24.0	135.0	13.2	36.5
3118031	1	2	1	云南省曲靖	36.0	164.0	13.4	36.4
3120460	1	3	1	云南省曲靖	56.0	165.0	20.6	36.5
3121195	1	2	1	昆明市西山	50.0	148.0	22.8	36.7
3124004	1	2	1	云南省西双	29.0	143.0	14.2	36.5
3123916	0	2	1	云南省临沧	23.0	145.0	10.9	36.6
3125285	0	3	1	临沧市凤庆	42.0	155.0	17.5	36.1
3123858	0	2	0	曲靖市宣威	25.0	153.0	10.7	36.6
3129348	0	2	1	昆明市五华	20.0	121.0	13.7	36.5
3128925	1	3	1	昭通市巧家	45.0	158.0	18.0	36.6
3128966	0	2	1	昭通市巧家	19.0	110.0	15.7	36.8
3129629	1	3	1	昆明市安宁	31.5	147.0	14.6	36.6
3129699	0	2	1	昆明市五华	65.0	163.0	24.5	36.4

3130490	1	3	1	昭通市镇	41.0	146.0	19.2	37.3
3131094	0	2	1	四川省凉	23.0	126.0	14.5	36.5
3132117	0	1	0	广西壮族	15.0	100.0	15.0	36.5
3132024	1	3	1	昆明市呈	40.0	158.0	16.0	36.9
3132039	0	2	1	昆明市东	45.0	145.0	21.4	36.6
3132134	0	2	1	昆明市晋	21.0	115.0	15.9	36.7
3136125	1	2	1	昆明市富	58.0	158.0	23.2	36.1
3135982	0	3	0	昆明市五	27.0	133.0	15.3	36.6
3135896	1	3	0	临沧市凤	45.6	151.0	20.0	36.8
3136911	1	3	1	大理白族	60.0	175.0	19.6	36.5
3137398	1	2	1	楚雄彝族	35.0	137.0	18.6	36.7
3108888	0	2	0	临沧市	31.0	147.0	14.3	36.9
3137871	0	2	0	昆明市五	20.0	120.0	13.9	36.4
3139032	0	2	0	红河哈尼	25.0	120.0	17.4	36.5
3139308	1	2	1	昆明市五	22.0	126.0	13.9	36.4
3140886	0	2	1	昆明市	24.5	136.0	13.2	36.8
3140511	0	2	0	腾冲市	75.0	162.0	28.6	36.8
2958211	1	2	0	文山壮族	38.5	147.0	17.8	36.5
3142523	1	2	1	临沧市	32.0	138.0	16.8	36.8
3143191	1	2	0	昆明市盘	21.0	120.0	14.6	36.5
3143547	0	2	1	昆明市嵩	26.0	130.0	15.4	36.5
3141326	0	2	1	玉溪市	23.0	131.0	13.4	36.5
3146402	0	3	0	丽江市古	33.0	144.0	15.9	36.3
3146877	0	2	1	玉溪市华	21.0	128.0	12.8	36.2
3166532	1	2	1	贵州省六	29.0	130.0	17.2	36.4
3147631	1	2	1	玉溪市通	38.0	141.0	19.1	36.4
3148879	0	2	1	昆明市五	100.0	165.0	36.7	37.1
3148079	1	3	0	普洱市墨	49.0	168.0	17.4	36.5
3150809	1	2	1	昭通市巧	22.0	123.0	14.5	36.7
3151310	1	2	1	昭通市巧	23.0	130.0	13.6	36.4
3151829	0	2	0	六盘水市	27.0	135.0	14.8	36.4
3151833	1	2	1	贵州省黔	30.0	135.0	16.5	36.5
3152864	1	2	1	贵州省黔	23.0	137.0	12.3	36.7
3152319	0	2	1	玉溪市红	44.0	155.0	18.3	36.1
3153129	0	2	1	昆明市官	60.0	163.0	22.6	36.5
3154062	1	2	1	昭通市镇	23.0	121.0	15.7	36.6
3152785	0	2	0	昆明市安	29.5	139.0	15.3	36.8
3154960	1	2	1	昆明市盘	25.0	140.0	12.8	36.6
3154386	0	2	1	昆明市嵩	23.0	139.0	11.9	36.6
3156217	1	2	0	红河哈尼	32.0	144.0	15.4	36.5
3037574	1	2	1	楚雄彝族	26.0	139.0	13.5	36.2
3155941	1	2	1	昭通市镇	29.0	135.0	15.9	36.5
3156577	0	2	1	昆明市东	37.0	135.0	20.3	36.7
3152934	1	2	1	昆明市呈	22.0	130.0	13.0	36.8
3152020	1	2	0	贵州省六	38.0	152.0	16.4	36.3
3159846	1	2	1	曲靖市宣	25.0	120.0	17.4	36.8
3160349	1	3	0	贵州省黔	42.5	155.0	17.7	36.5
3160939	1	2	1	西双版纳	17.0	110.0	14.0	36.7
3161226	1	2	1	贵州省黔	31.0	142.0	15.4	36.6
3163142	1	3	1	云南省凤	32.0	151.0	14.0	37.5
3163207	1	2	1	昆明市五	21.0	125.0	13.4	36.6
3163763	0	2	1	昆明市富	42.0	158.0	16.8	36.5
3163891	0	2	1	昭通市	30.0	131.0	17.5	36.4
3163173	1	2	1	红河哈尼	41.0	135.0	22.5	36.4

3164486	0	2	0 昆明市	24.0	130.0	14.2	36.6
3165063	1	2	1 云南省大理	26.0	127.0	16.1	36.7
3166003	1	2	1 楚雄彝族自	28.0	128.0	17.1	36.5
3164990	0	2	1 昆明市西山	16.0	115.0	12.1	36.5
3166899	0	2	0 大理白族自	42.5	151.0	18.6	36.5
3166428	1	2	0 贵州省黔东	25.0	120.0	17.4	36.5
3167152	1	2	1 昆明市	33.0	132.0	18.9	36.6
3168216	1	2	0 贵州省黔南	30.0	140.0	15.3	36.8
3169244	0	2	1 昆明市富民	25.0	130.0	14.8	36.5
3169466	1	2	1 昆明市五华	23.0	128.0	14.0	36.5
3169783	0	2	1 昆明市石林	40.0	148.0	18.3	37.1
3170761	1	2	1 云南省大理	30.0	141.0	15.1	36.4
3170944	0	3	1 昆明市嵩明	66.0	160.0	25.8	36.9
3170951	1	3	1 昆明市五华	63.8	183.0	19.1	36.6
3171094	1	2	1 楚雄彝族自	39.0	140.0	19.9	36.2
3172415	1	2	1 楚雄彝族自	41.0	160.0	16.0	36.4
3172370	1	3	0 文山壮族苗	40.0	162.0	15.2	36.4
3173318	1	3	1 昆明市西山	33.0	154.0	13.9	36.5
3173833	0	1	1 昆明市五华	14.0	95.0	15.5	37.0
3152934	1	2	1 昆明市呈贡	24.0	130.0	14.2	36.5
3174969	1	2	0 大理白族自	35.5	135.0	19.5	36.5
3176815	0	2	1 曲靖市宣威	22.0	122.0	14.8	36.8
3151349	1	2	1 昆明市五华	30.0	136.0	16.2	36.5
3177931	0	3	0 红河哈尼族	52.0	160.0	20.3	36.5
3175641	1	3	1 云南省曲靖	51.0	170.0	17.6	36.5
3177982	1	2	1 昆明市五华	28.0	127.7	17.2	36.6
3178240	0	2	0 大理白族自	13.1	107.0	11.4	36.5
3175547	1	2	0 贵州省六盘	27.5	129.0	16.5	36.6
3180082	0	2	1 大理白族自	22.0	128.0	13.4	36.8
3180984	0	2	0 结肠息肉	50.0	155.0	20.8	36.3
3181014	1	2	1 云南省大理	16.0	100.0	16.0	36.6
3165988	1	2	0 云南省西双	17.8	120.0	12.4	36.5
2933719	0	3	1 昆明市安宁	32.0	137.0	17.0	36.8
3183409	0	1	1 昆明市嵩明	15.0	101.0	14.7	36.6
3182695	1	2	1 曲靖市会泽	38.0	140.0	19.4	36.6
3183722	0	1	0 昆明市官渡	15.0	103.0	14.1	36.4
3183111	0	2	1 昆明市五华	13.0	135.0	7.1	36.5
3183754	1	1	0 昭通市鲁甸	10.0	82.0	14.9	36.5
3184598	0	2	1 昆明市官渡	25.0	142.0	12.4	36.6
2915916	1	2	1 宜良县	22.0	125.0	14.1	36.6
3185613	0	2	1 昆明市五华	30.0	135.0	16.5	36.6
3185908	1	2	1 曲靖市宣威	27.0	135.0	14.8	36.6
3184627	0	2	1 昭通市镇雄	29.0	135.0	15.9	36.5
3186877	0	2	1 昆明市官渡	23.0	132.0	13.2	36.0
3187939	0	2	0 云南省西双	28.5	137.0	15.2	36.7
3188271	1	2	1 昆明市五华	16.5	115.0	12.5	36.5
3190215	1	2	1 昆明市官渡	23.0	128.0	14.0	36.5
3150270	0	2	0 红河哈尼族	21.0	121.0	14.3	36.5
3103412	1	2	1 楚雄彝族自	30.0	132.0	17.2	36.6
3193253	0	2	1 昆明市禄劝	45.0	143.0	22.0	36.5
3193282	0	2	0 云南省西双	23.0	120.0	16.0	36.6
3193309	0	2	1 曲靖市罗平	47.0	158.0	18.8	36.5
3194098	1	2	1 玉溪市	30.0	130.0	17.8	36.4
3193467	0	3	1 昆明市	43.0	151.0	18.9	36.5

3195021	0	2	1	曲靖市会泽	50.0	143.0	24.5	36.5
3196309	0	2	1	昆明市	16.5	115.0	12.5	36.4
3196017	0	2	1	曲靖市陆良	17.3	108.0	14.8	36.5
3195448	1	2	1	普洱市景谷	21.0	111.0	17.0	36.9
3196656	1	2	0	临沧市永德	40.0	172.0	13.5	36.6
3197964	1	2	1	丽江市华坪	28.0	137.0	14.9	36.6
3197875	1	2	1	昆明市五华	53.0	140.0	27.0	36.4
3199208	1	2	1	曲靖市宣威	50.0	148.0	22.8	36.1
3199884	0	3	1	昆明市晋宁	50.0	170.0	17.3	36.5
3200291	1	2	1	云南省泸西	57.0	165.0	20.9	36.5
3201894	1	2	1	昆明市	49.0	145.0	23.3	36.6
3202466	1	2	1	曲靖市陆良	33.0	132.0	18.9	36.5
3201970	0	3	1	昆明市西山	65.0	165.0	23.9	36.3
3202516	1	3	1	文山壮族苗	52.0	150.0	23.1	36.4
3202779	0	2	1	昆明市五华	40.0	154.0	16.9	36.5
3203263	0	2	0	临沧市凤庆	40.0	145.0	19.0	36.7
3202580	1	2	1	昆明市寻甸	94.0	165.0	34.5	36.8
3204193	1	3	1	昭通市大关	54.5	172.0	18.4	36.7
3204339	1	2	0	昭通市大关	31.0	126.0	19.5	36.5
3205529	0	2	0	楚雄彝族自	27.0	125.0	17.3	36.6
3204848	1	2	1	昆明市富民	57.0	152.0	24.7	36.7
3204965	1	3	1	昆明市寻甸	39.0	156.0	16.0	36.4
3205776	1	2	0	红河哈尼族	36.0	155.0	15.0	36.5
3205809	0	2	1	临沧市临翔	24.0	125.0	15.4	36.5
3206548	0	2	0	香格里拉市	25.0	135.0	13.7	36.5
3204871	1	2	0	文山壮族苗	30.0	133.0	17.0	36.9
3207530	0	2	0	昆明市五华	33.0	145.0	15.7	36.6
3208044	0	2	1	文山壮族苗	18.5	126.0	11.7	36.5
3207648	0	2	1	红河哈尼族	32.0	140.0	16.3	36.5
3208246	0	3	1	昭通市巧家	43.0	157.0	17.4	36.9
3207935	0	2	1	昆明市五华	80.0	155.0	33.3	36.2
3208003	0	2	1	昆明市富民	30.0	150.0	13.3	36.4
3208189	0	2	1	昆明市西山	62.0	160.0	24.2	36.6
3208292	1	2	1	红河州泸西	26.0	125.0	16.6	36.6
3208504	1	2	0	昆明市西山	23.0	130.0	13.6	36.4
3208526	0	2	1	曲靖市陆良	45.0	156.0	18.5	36.5
3208753	0	2	1	红河哈尼族	32.0	150.0	14.2	36.8
3209436	1	2	1	临沧市凤庆	24.5	130.0	14.5	36.3
3210523	1	3	1	贵州省威宁	33.0	139.0	17.1	36.3
3210990	1	2	0	楚雄彝族自	44.0	140.0	22.4	36.6
3211921	0	2	1	曲靖市宣威	41.0	151.0	18.0	36.3
3211240	0	3	0	楚雄彝族自	39.0	168.0	13.8	36.1
3211613	0	2	1	临沧市镇康	32.0	134.0	17.8	36.6
3211910	1	3	1	红河哈尼族	47.0	156.0	19.3	36.5
3212108	1	2	1	曲靖市会泽	42.0	156.0	17.3	36.2
3212594	1	2	1	昆明市盘龙	48.0	145.0	22.8	36.5
3213875	0	2	0	玉溪市通海	40.0	154.0	16.9	36.6
3214344	0	2	1	昆明市安宁	46.0	162.0	17.5	36.5
3215012	1	3	0	临沧市永德	124.0	182.0	37.4	36.6
3215363	1	2	1	昆明市五华	39.0	143.0	19.1	36.3
3216059	0	2	0	丽江市永胜	44.0	155.0	18.3	36.3
3215792	1	3	0	楚雄彝族自	49.0	163.0	18.4	36.6
3216417	1	2	1	临沧市永德	30.0	142.0	14.9	37.0
3217064	1	2	0	楚雄彝族自	41.0	146.0	19.2	36.5

3216413	0	2	0 临沧市永德	40.0	153.0	17.1	37.1
3212930	1	2	1 昆明市五华	50.0	168.0	17.7	36.9
3217798	1	2	0 红河哈尼族	39.0	139.0	20.2	36.6
3218003	1	2	1 昆明市五华	21.0	120.0	14.6	36.9
3219375	1	2	1 楚雄彝族自	30.0	133.0	17.0	36.3
3219590	0	2	1 昆明市寻甸	49.0	155.0	20.4	36.5
3220691	0	2	1 保山市龙陵	45.0	165.0	16.5	36.5
3220809	0	3	1 云南省昆明	34.0	155.0	14.2	36.9
3221525	1	3	0 临沧市临翔	45.0	165.0	16.5	36.3
3222571	1	2	0 楚雄彝族自	55.0	141.0	27.7	36.5
3222968	1	2	0 楚雄彝族自	49.0	156.0	20.1	36.7
3225773	0	2	1 昭通市镇雄	28.0	136.0	15.1	36.3
3225790	1	2	1 昆明市西山	31.0	135.0	17.0	36.3
3222570	0	2	0 文山州富宁	21.0	120.0	14.6	36.5
3227696	0	2	1 昆明市呈贡	45.0	154.0	19.0	36.8
3222484	0	3	1 宣威市	54.0	158.0	21.6	36.4
3228311	0	2	1 大理白族自	45.0	150.0	20.0	36.5
3141326	0	2	1 玉溪市红塔	27.5	138.0	14.4	36.5
3229939	1	2	1 昆明市五华	24.0	145.0	11.4	37.0
2915916	1	2	1 昆明市宜良	23.5	129.0	14.1	36.6
3230744	1	2	0 云南省怒江	40.0	137.0	21.3	36.5
3230875	0	2	0 文山壮族苗	23.0	130.0	13.6	36.8
3231961	1	2	1 昆明市晋宁	31.0	138.0	16.3	36.5
3231295	1	3	0 昆明市五华	55.0	163.0	20.7	36.6
3232731	1	3	1 昭通市	44.0	160.0	17.2	36.5
2926904	1	1	1 红河州泸西	10.0	84.0	14.2	36.3
3234510	1	2	1 昭通市镇雄	35.0	135.0	19.2	36.6
3209436	1	2	1 临沧市凤庆	21.7	129.0	13.0	36.9
3236022	0	2	1 贵州省六盘	34.5	130.0	20.4	36.3
3236749	0	3	1 玉溪市元江	52.0	156.0	21.4	36.6
3236388	0	2	1 湖南省常德	105.0	163.0	39.5	36.3
3237047	1	3	1 贵州省安龙	42.0	158.0	16.8	36.6
3176815	0	2	1 曲靖市宣威	27.0	123.0	17.8	36.8
3101527	1	2	1 昆明市五华	33.0	146.0	15.5	36.0
3239633	0	2	0 临沧市云县	25.0	123.0	16.5	36.6
3239037	1	3	1 玉溪市澄江	75.0	168.0	26.6	36.5
3240128	0	3	1 昆明市安宁	22.0	120.0	15.3	36.5
3240117	1	2	0 昆明市富民	43.0	145.0	20.5	36.3
3241260	0	2	1 曲靖市富源	27.0	125.0	17.3	36.5
3241742	1	2	1 昆明市安宁	48.0	152.0	20.8	36.6
3241774	0	2	1 昆明市呈贡	20.0	115.0	15.1	36.4
3241848	1	3	1 曲靖市富源	52.0	170.0	18.0	36.4
3243120	0	2	1 昆明市	32.5	149.0	14.6	36.2
3228324	1	2	1 贵州省黔南	34.0	130.0	20.1	36.7
3243177	0	2	1 昆明市呈贡	37.5	155.0	15.6	36.7
3242975	0	2	1 昭通市镇雄	22.0	126.0	13.9	36.3
3220691	0	3	1 保山市龙陵	47.0	165.0	17.3	36.5
3244207	0	3	1 昆明市五华	39.0	158.0	15.6	36.3
3244740	1	2	0 昆明市呈贡	22.0	127.0	13.6	36.5
3244693	1	2	1 曲靖市陆良	61.0	150.0	27.1	36.9
3244696	1	3	1 普洱市宁洱	36.0	150.0	16.0	36.5
3158571	1	2	1 曲靖市宣威	41.0	156.0	16.8	36.4
3245338	1	2	0 西双版纳傣	60.0	148.0	27.4	36.8
3246478	0	2	1 昆明市呈贡	36.0	150.0	16.0	36.8

3246118	1	2	1	昆明市寻钅	48.0	145.0	22.8	36.3
3246545	1	2	1	大理市鹤庆	35.0	140.0	17.9	36.5
3245911	0	3	0	昆明市五华	65.0	168.0	23.0	39.4
3248618	1	2	1	昆明市	16.0	106.0	14.2	36.4
3248754	1	2	0	文山州丘北	34.0	135.0	18.7	36.8
3248646	1	2	1	楚雄彝族自	37.0	139.0	19.2	36.4
3219375	1	2	1	楚雄彝族自	35.0	134.0	19.5	36.4
3222484	0	3	1	宣威市	53.0	160.0	20.7	36.5
3248814	1	3	1	曲靖市会泽	60.0	175.0	19.6	36.6
3237206	0	2	1	昭通市镇雄	18.0	118.0	12.9	36.8
3249693	1	2	1	昆明市五华	30.0	135.0	16.5	36.5
3249893	0	2	1	昆明市官渡	25.0	129.0	15.0	36.5
3246739	1	3	1	宣威市龙场	60.0	168.0	21.3	36.4
3217259	1	2	1	昆明市	77.0	160.0	30.1	36.8
3250731	1	2	0	大理白族自	30.0	120.0	20.8	36.7
3251492	1	2	0	玉溪市易门	32.0	126.0	20.2	36.4
3252123	0	2	0	建水县	31.0	149.0	14.0	37.1
3244466	0	2	1	昆明市五华	38.0	145.0	18.1	36.2
3252702	1	2	0	昆明市盘龙	38.0	142.0	18.8	36.4
3252854	1	3	1	昆明市嵩明	92.0	173.0	30.7	36.5
3252742	0	3	1	昆明市五华	58.0	171.0	19.8	37.6
3253242	1	2	1	贵州省毕节	20.0	117.0	14.6	36.2
3252916	0	2	1	昆明市西山	31.0	144.0	14.9	36.2
3254029	1	2	1	楚雄彝族自	24.0	122.0	16.1	37.0
3254223	0	2	1	临沧市临翔	32.0	137.0	17.0	36.8
3254205	1	1	1	昭通市威信	15.0	97.0	15.9	37.6
3251529	0	1	1	云南省丽江	15.0	104.0	13.9	36.7
3254948	0	3	1	贵州省水城	32.0	151.0	14.0	37.0
3255682	1	2	1	昆明市五华	27.0	130.0	16.0	36.6
3255701	1	2	1	昆明市	23.0	115.0	17.4	36.3
3255440	1	2	1	昆明市五华	47.0	150.0	20.9	36.3
3256291	1	2	1	贵州省盘县	28.0	133.0	15.8	36.5
3257695	0	2	1	昭通巧家县	16.0	115.0	12.1	36.5
3258140	1	2	0	云南省大理	29.0	132.0	16.6	36.6
3258826	1	2	1	云南省昆明	60.0	150.0	26.7	36.4
3258751	1	2	0	楚雄彝族自	15.0	103.0	14.1	36.5
3258747	1	1	0	昆明市五华	15.0	100.0	15.0	36.5
3260985	0	3	1	贵州省毕节	40.0	160.0	15.6	36.0
3260000	1	2	0	昆明市五华	47.0	110.0	38.8	36.5
2889138	0	2	0	昆明市五华	21.0	113.0	16.4	37.2
3261025	0	3	1	昆明市五华	46.0	155.0	19.1	36.5
3261555	1	2	1	昆明市呈贡	35.0	145.0	16.6	37.0
3262996	1	2	1	昆明市五华	23.0	132.0	13.2	36.7
3263499	0	2	0	楚雄彝族自	36.0	140.0	18.4	36.8
3264576	0	2	1	昆明市官渡	30.0	140.0	15.3	36.7
3265179	1	3	1	曲靖市陆良	35.0	160.0	13.7	36.7
3265117	1	3	1	曲靖市富源	57.0	163.0	21.5	36.4
3265559	0	3	1	昆明市禄劝	35.0	145.0	16.6	36.7
3265178	1	3	0	昆明市禄劝	28.0	130.0	16.6	36.7
3011175	1	2	1	昆明市	32.0	150.0	14.2	36.6
3266231	1	2	1	曲靖市富源	52.0	164.0	19.3	36.5
3266271	0	3	1	昆明市宜良	54.0	160.0	21.1	36.7
3264900	1	2	1	文山壮族苗	34.0	135.0	18.7	36.7
3203475	1	2	1	昆明市	50.0	172.0	16.9	37.4

3267270	0	1	1	楚雄彝族自	11.0	90.0	13.6	36.5
3176815	0	2	1	曲靖市宣威	26.0	128.0	15.9	36.6
3266218	0	2	1	昭通市巧家	34.0	105.0	30.8	37.2
3267448	1	2	1	贵州省六盘	26.0	125.0	16.6	36.5
3265384	0	2	1	普洱市镇沅	30.0	144.0	14.5	36.6
3267928	0	2	0	大理白族自	22.0	131.0	12.8	36.5
3251251	0	3	1	腾冲市新华	44.0	158.0	17.6	36.7
3268811	0	2	1	昆明市寻甸	28.0	140.0	14.3	36.4
3270062	0	2	1	昆明市五华	35.0	147.0	16.2	36.7
3269512	0	2	1	昆明市五华	26.0	142.0	12.9	36.5
3271052	1	2	1	昆明市	49.0	145.0	23.3	36.5
3270905	1	2	1	昆明市官渡	19.0	116.0	14.1	37.0
3270984	0	2	1	云南省昆明	35.0	149.0	15.8	36.4
3271017	0	1	0	云南省临沧	11.0	87.0	14.5	36.7
2925206	0	3	1	曲靖市会泽	33.0	145.0	15.7	36.7
3271739	1	2	1	保山市施甸	42.0	164.0	15.6	36.5
3271758	1	2	1	曲靖市	29.0	143.0	14.2	36.6
3272272	1	2	1	云南省保山	42.0	141.0	21.1	36.1
3271628	0	2	1	昆明市五华	21.0	118.0	15.1	36.4
3272192	0	3	0	红河哈尼族	77.0	163.0	29.0	36.5
3272371	0	2	0	昆明市富民	41.0	145.0	19.5	36.7
3272519	0	2	0	曲靖市师宗	28.0	144.0	13.5	36.6
3272684	0	2	1	曲靖市麒麟	32.0	145.0	15.2	36.6
3271135	1	2	1	曲靖市宣威	26.0	130.0	15.4	36.4
3272340	1	2	0	昆明市盘龙	71.0	166.0	25.8	36.5
3272362	0	2	0	昆明市	28.0	140.0	14.3	36.5
3273454	1	2	1	昆明市五华	65.0	156.0	26.7	36.4
3273875	1	2	1	曲靖市麒麟	21.0	119.0	14.8	36.6
3274451	0	2	1	临沧市永德	28.0	127.0	17.4	36.6
3274563	0	2	1	临沧市云县	36.5	154.0	15.4	36.5
3273241	1	2	0	昆明市五华	33.0	120.0	22.9	36.5
3270809	0	3	1	昆明市官渡	56.0	163.0	21.1	36.6
3275000	0	2	1	曲靖市陆良	52.0	160.0	20.3	36.5
3270162	0	2	1	云南省大理	41.0	146.0	19.2	36.5
3274893	1	3	1	临沧市凤庆	34.0	148.0	15.5	36.7
3275069	0	3	1	昆明市宜良	63.0	176.0	20.3	36.5
3275883	1	2	0	临沧市永德	36.0	140.0	18.4	36.6
3275768	1	3	1	昆明市	97.0	177.0	31.0	36.7
3274615	0	2	1	红河哈尼族	26.3	139.0	13.6	36.8
3276387	1	2	0	昆明市五华	36.5	153.0	15.6	36.8
3274474	1	2	0	昆明市五华	24.0	137.0	12.8	36.7
3274585	1	2	1	昆明市五华	25.0	125.0	16.0	36.6
3276057	1	2	0	昆明市安宁	26.0	137.0	13.9	36.5
3276574	1	3	1	红河州泸西	59.0	173.0	19.7	37.2
3277216	1	2	1	昆明市	27.0	137.0	14.4	36.5
3276449	0	3	1	昆明市官渡	55.0	165.0	20.2	36.6
3276656	1	2	1	曲靖市富源	56.0	153.0	23.9	36.6
3088783	0	3	0	昆明市五华	62.0	168.0	22.0	36.6
3277361	0	2	0	大理白族自	105.0	171.0	35.9	36.5
3272381	1	2	1	昆明市五华	20.0	115.0	15.1	36.3
3277540	0	2	1	大理白族自	26.5	133.0	15.0	36.7
3277879	0	2	0	昆明市五华	40.0	155.0	16.6	36.6
3276929	0	2	0	贵州省六盘	34.0	145.0	16.2	36.6
3275957	0	3	1	曲靖市陆良	43.0	156.0	17.7	36.8

3278902	1	3	1	文山州丘オ	42.0	156.0	17.3	37.4
3278810	1	2	1	昆明市五华	27.5	140.0	14.0	36.5
3277846	1	2	0	红河哈尼放	45.0	155.0	18.7	36.5
3279680	0	2	1	宣威市	23.0	135.0	12.6	36.7
3279224	0	2	0	红河哈尼放	43.0	153.0	18.4	36.7
3280123	1	2	1	西双版纳	35.3	152.0	15.3	36.7
3280398	1	2	1	曲靖市宣威	34.0	142.0	16.9	36.6
3281246	0	2	1	昆明市五华	19.0	114.0	14.6	36.5
3278759	0	2	1	贵州省毕节	31.0	140.0	15.8	36.5
3281332	1	2	1	昆明市西山	24.0	127.0	14.9	36.5
3279756	0	2	1	宣威市	27.0	138.0	14.2	36.5
3281937	0	2	1	临沧市凤庆	52.0	152.0	22.5	36.6
3281364	1	2	1	曲靖市会泽	45.0	132.0	25.8	36.3
3282689	0	3	1	临沧市临翔	58.0	160.0	22.7	36.5
3280516	1	3	0	昆明市五华	73.0	185.0	21.3	36.4
3284302	0	3	1	昆明市五华	47.0	156.0	19.3	36.6
3244207	0	3	1	昆明市五华	34.0	155.0	14.2	36.2
3283638	1	3	1	文山州广南	43.0	153.0	18.4	36.8
3284396	0	2	1	湖南省双峰	51.0	164.0	19.0	36.5
3282700	1	3	1	曲靖市宣威	57.5	167.0	20.6	36.5
3283233	1	2	0	普洱市耿马	30.0	145.0	14.3	36.9
3284384	1	3	1	曲靖市宣威	67.0	174.0	22.1	36.5
3286272	0	2	1	昆明市西山	21.0	123.0	13.9	36.5
3208370	0	3	1	昆明市盘龙	54.0	157.0	21.9	36.6
3168216	1	2	0	贵州省兴义	31.5	145.0	15.0	36.5
3287037	1	2	1	红河哈尼放	25.0	125.0	16.0	36.5
3287480	1	3	1	宣威市	55.0	172.0	18.6	36.7
3287427	1	2	1	云南省德宏	30.0	137.0	16.0	36.4
3285686	1	1	0	云南省昆明	14.0	98.0	14.6	36.5
3050161	1	2	1	云南省罗平	30.0	135.0	16.5	36.5
3287899	1	2	1	昆明市五华	72.0	155.0	30.0	36.2
3287951	1	2	1	昆明市五华	19.5	137.0	10.4	36.9
3288542	0	3	1	昆明市呈贡	47.0	153.0	20.1	36.8
3289124	0	3	1	昭通市巧家	35.0	145.0	16.6	36.5
3287967	1	3	1	贵州省兴义	63.0	166.0	22.9	36.7
3259328	0	2	1	昆明市呈贡	33.5	145.0	15.9	36.0
3289987	0	2	0	昆明市禄劝	27.0	135.0	14.8	36.5
3289176	1	2	1	昆明市五华	30.0	140.0	15.3	36.4
3289882	0	2	0	昆明市宜良	23.0	125.0	14.7	36.6
3289894	1	1	0	云南省文山	14.0	95.0	15.5	36.5
3275518	1	2	1	河北省任丘	30.0	140.0	15.3	36.5
3291120	1	2	1	昆明市呈贡	33.0	145.0	15.7	36.2
3289941	0	2	1	云南省大理	12.0	95.0	13.3	37.5
3288775	0	3	1	楚雄彝族自	47.0	160.0	18.4	36.5
3084900	0	2	1	曲靖市马龙	30.0	146.0	14.1	37.2
3291589	1	3	1	宣威市	60.0	175.0	19.6	36.2
3291993	0	3	0	红河哈尼放	69.0	168.0	24.4	36.3
3291881	1	3	1	昆明市五华	47.5	165.0	17.4	36.6
2868314	1	2	0	昆明市	29.0	145.0	13.8	36.9
3293893	1	3	1	昆明市盘龙	53.0	170.0	18.3	36.8
3294125	0	3	1	昆明市盘龙	60.0	162.0	22.9	36.3
3294176	0	3	0	贵州省黔西	54.0	157.0	21.9	36.3
3000105	1	2	1	玉溪市澄江	27.0	135.0	14.8	36.6
3294902	0	3	1	昆明市晋宁	58.0	164.0	21.6	36.8

3294126	1	2	1	贵州省遵义	15.0	102.0	14.4	36.5
3294324	1	2	1	文山市广南	25.0	130.0	14.8	36.7
3296292	0	2	1	楚雄彝族自	29.0	133.0	16.4	36.7
3294863	0	2	1	云南省大理	53.0	160.0	20.7	36.7
3297982	0	2	1	昆明市晋宁	25.0	130.0	14.8	36.6
3297654	1	2	1	昆明市五华	58.0	156.0	23.8	36.6
3298557	1	2	1	昆明市五华	33.0	148.0	15.1	36.6
3299794	1	2	1	昆明市	33.0	145.0	15.7	36.5
3297695	1	2	0	昆明市呈贡	42.0	152.0	18.2	36.4
3300184	1	2	1	曲靖市会泽	17.0	95.0	18.8	36.6
3301037	1	2	1	云南省德宏	33.0	137.0	17.6	36.7
3151874	0	2	1	昆明市五华	28.5	145.0	13.6	37.2
3301922	1	2	1	昆明市官渡	41.0	144.0	19.8	36.2
3300797	0	2	1	云南省楚雄	17.0	123.0	11.2	36.7
3302810	1	3	1	昭通市镇雄	56.0	171.0	19.2	36.6
3302010	0	2	1	曲靖市会泽	45.0	161.0	17.4	36.1
3303505	1	1	1	昭通市巧家	13.5	94.0	15.3	36.5
3304018	1	2	0	临沧市永德	28.0	135.0	15.4	36.7
3303836	1	3	1	昭通市镇雄	37.0	152.0	16.0	36.5
3271135	1	2	1	曲靖市宣威	26.0	134.0	14.5	36.5
3304640	0	2	1	保山市昌宁	34.0	150.0	15.1	36.6
3304452	1	2	1	临沧市沧源	19.0	111.0	15.4	36.4

脉搏	呼吸	血压	腹痛	腹胀	恶心	呕吐	吞咽困难	黄疸
95	22	1	1	0	0	0	0	0
87	24	1	1	0	0	0	0	0
88	20	1	1	0	0	0	0	0
108	22	1	1	0	0	0	0	0
88	22	1	0	0	0	1	0	0
105	18	3	0	0	0	0	0	0
74	22	1	1	0	0	0	0	0
95	20	2	1	0	0	0	0	0
100	20	1	1	0	0	0	0	0
96	22	1	1	0	0	0	0	0
88	22	1	1	0	0	0	0	0
104	18	1	1	0	0	0	0	0
96	22	1	0	0	0	0	0	0
96	20	1	1	0	0	0	0	0
110	20	1	1	0	0	0	0	0
86	22	1	1	0	0	0	0	0
116	20	1	0	0	0	0	0	0
105	20	1	0	0	0	0	0	0
64	18	1	1	0	0	0	0	0
74	22	1	1	0	0	0	0	0
86	18	3	0	0	0	1	0	0
81	21	3	1	0	0	0	0	0
90	20	1	1	0	0	0	0	0
68	22	1	1	0	0	0	0	0
76	24	3	1	0	0	0	0	0
82	20	1	1	0	0	0	0	0
82	21	3	1	0	0	1	0	0
126	20	3	1	0	0	1	0	0
90	20	1	1	0	0	0	0	0
88	22	1	1	0	0	0	0	0
95	21	1	1	0	0	0	0	0
91	20	3	1	0	0	1	0	0
74	20	1	1	0	0	0	0	0
90	20	1	1	0	0	0	0	0
55	18	1	1	0	0	0	0	0
90	22	1	1	0	0	0	0	0
96	23	3	0	0	0	0	0	0
92	21	1	0	0	1	0	0	0
115	21	3	1	1	0	0	0	0
101	21	1	1	0	0	0	0	0
90	18	3	1	0	0	0	0	0
76	25	1	1	0	0	0	0	0
100	21	2	1	0	0	0	0	0
85	20	1	1	0	0	0	0	0
86	20	1	1	0	0	0	0	0
80	18	1	1	0	0	1	0	0
84	21	1	1	0	0	0	0	0
54	18	2	0	0	0	1	0	0
98	22	1	1	0	0	0	0	0
81	20	1	1	0	0	0	0	0
88	18	1	0	0	0	0	0	0
80	22	1	1	0	0	0	0	0
64	18	1	1	0	0	0	0	0

88	18	1	0	0	0	0	0	0
113	23	1	0	0	1	1	0	0
100	22	1	1	0	0	0	0	0
90	18	2	0	0	0	0	0	0
100	20	1	1	0	0	0	0	0
95	20	1	1	0	0	0	0	0
82	18	1	1	0	0	0	0	0
90	21	3	1	0	0	0	0	0
65	20	1	1	0	0	0	0	0
90	20	1	1	0	0	0	0	0
130	30	1	1	0	0	1	0	0
85	21	1	1	0	0	0	0	0
80	21	1	1	0	0	0	0	0
84	20	1	1	0	0	0	0	0
96	20	1	1	0	0	0	0	0
90	20	1	0	0	0	0	0	0
92	23	1	0	0	0	0	0	0
104	20	1	0	0	0	0	0	0
78	17	1	0	0	0	0	0	0
96	20	1	0	0	0	0	0	0
110	18	1	1	0	0	0	0	0
74	24	1	1	0	0	1	0	0
80	18	1	0	0	0	1	0	0
86	20	2	1	1	0	0	0	0
68	18	1	1	0	0	0	0	0
75	20	1	1	0	0	0	0	0
80	22	1	0	0	0	1	0	0
85	21	1	0	0	0	0	0	0
90	20	1	0	0	0	0	0	0
89	20	3	0	1	0	0	0	0
96	20	3	0	0	0	0	0	0
93	23	1	1	0	1	1	0	0
106	22	3	0	0	0	0	0	0
88	22	1	1	0	0	0	0	0
78	18	1	1	0	0	0	0	0
106	20	3	1	0	0	1	0	0
82	22	1	0	0	0	0	0	0
92	23	1	0	0	0	0	0	0
92	20	1	1	0	0	0	0	0
69	17	1	1	0	0	0	0	0
82	22	1	0	0	0	0	0	0
86	18	1	1	0	0	0	0	0
89	22	1	1	0	0	0	0	0
88	22	1	1	0	0	0	0	0
67	20	2	1	0	0	0	0	0
94	18	1	0	0	0	1	0	0
100	20	1	1	0	1	1	0	0
110	20	2	0	0	0	0	0	0
108	23	1	1	0	0	1	0	0
88	22	1	1	0	0	0	0	0
78	20	1	1	0	0	0	0	0
80	22	1	1	0	0	0	0	0
94	22	1	0	0	0	0	0	0
110	22	1	0	0	0	0	0	0

90	20	3	0	0	0	0	0	0
86	20	1	0	0	0	0	0	0
93	21	1	1	0	0	0	0	0
86	19	1	1	0	0	0	0	0
98	21	1	1	0	0	0	0	0
108	20	1	1	0	0	0	0	0
90	22	1	1	0	0	0	0	0
80	22	1	1	0	0	0	0	0
120	23	3	0	0	0	0	0	0
86	22	1	0	0	0	0	0	0
70	20	2	1	0	0	1	0	0
72	19	2	1	0	0	1	0	0
85	20	1	1	0	0	0	0	0
82	18	1	0	0	0	1	0	0
90	18	1	0	0	1	1	0	0
82	20	1	1	0	0	0	0	0
82	22	1	1	0	0	0	0	0
88	22	3	1	0	0	0	0	0
79	19	1	1	0	0	0	0	0
100	29	1	1	0	0	0	0	0
80	20	1	1	0	0	1	0	0
116	20	3	0	0	0	0	0	0
80	22	3	0	0	0	0	0	0
102	20	1	1	0	0	0	0	0
90	20	1	0	0	0	0	0	0
102	21	3	0	0	0	0	0	0
85	18	1	1	0	0	0	0	0
85	25	1	1	0	1	1	0	0
84	19	1	1	0	0	1	0	0
78	21	1	1	0	0	0	0	0
95	22	1	1	0	0	1	0	0
98	20	1	1	0	0	0	0	0
96	19	1	1	0	0	0	0	0
72	18	1	1	0	0	0	0	0
98	20	1	1	0	0	0	0	0
84	20	1	1	0	0	0	0	0
90	22	1	1	0	0	1	0	0
90	22	1	1	1	0	0	0	0
117	20	2	1	0	0	0	0	0
72	20	1	1	0	0	0	0	0
140	24	3	0	0	0	0	0	0
84	20	1	1	0	0	0	0	0
72	20	1	1	0	0	0	0	0
100	18	2	0	0	0	0	0	0
84	20	1	1	0	0	0	0	0
98	22	1	1	0	0	0	0	0
70	20	1	0	1	0	0	0	0
72	20	1	1	0	0	0	0	0
84	20	1	1	0	0	0	0	0
78	20	1	1	0	0	0	0	0
84	20	1	1	0	0	1	0	0
102	24	1	1	0	0	0	0	0
100	22	1	1	0	0	0	0	0
86	22	3	0	0	0	1	0	0

84	20	1	0	0	0	1	0	0
84	22	1	1	0	0	0	0	0
150	35	2	1	0	0	0	0	0
70	22	1	1	0	0	0	0	0
92	20	1	0	0	0	0	0	0
80	20	1	0	0	0	1	0	0
80	20	1	1	0	0	0	0	0
114	24	1	0	0	0	1	0	0
110	24	1	0	0	0	0	0	0
84	20	1	1	0	0	0	0	0
79	21	1	1	0	0	0	0	0
79	21	2	0	1	0	0	0	0
97	20	1	0	0	0	0	0	0
120	30	3	0	0	0	0	0	0
100	24	1	1	0	0	0	0	0
78	21	3	1	0	0	0	0	0
80	20	3	1	0	0	0	0	0
96	22	1	1	0	0	0	0	0
112	25	1	1	0	0	0	0	0
88	19	1	1	0	0	0	0	0
66	18	3	1	0	0	0	0	0
96	22	1	0	0	0	0	0	0
94	22	3	1	0	0	0	0	0
68	17	3	0	0	1	0	0	0
74	20	3	0	0	0	0	0	0
112	22	3	1	0	0	1	0	0
70	18	1	1	0	0	0	0	0
100	28	3	1	0	0	0	0	0
120	23	1	0	0	0	0	0	0
102	25	1	0	0	0	0	0	0
86	20	1	1	0	0	0	0	0
80	22	1	0	0	0	1	0	0
89	23	3	0	0	0	0	0	0
89	26	1	0	0	0	0	0	0
90	21	1	1	0	0	0	0	0
65	20	1	1	0	0	0	0	0
60	18	1	0	0	0	0	0	0
90	23	1	1	0	0	0	0	0
100	24	1	1	0	0	1	0	0
90	21	3	1	0	0	0	0	0
108	20	1	0	0	0	1	0	0
78	24	3	1	0	0	0	0	0
86	22	3	1	0	0	0	0	0
78	18	1	1	0	0	0	0	0
80	18	3	1	1	0	0	0	0
98	22	1	1	0	0	0	0	0
80	18	3	0	0	0	0	0	0
84	18	1	0	0	0	0	0	0
100	20	2	0	0	0	0	0	0
86	22	1	1	0	0	1	0	0
94	28	3	0	0	0	0	0	0
74	24	1	0	0	0	1	0	0
86	20	1	0	0	0	0	0	0
88	20	3	1	0	0	0	0	0

72	22	1	1	0	0	0	0	0
95	22	3	0	0	0	0	0	0
72	20	1	1	0	0	0	0	0
76	19	2	0	0	0	0	0	0
69	26	3	1	0	0	0	0	0
98	20	3	1	0	0	1	0	0
96	19	2	1	0	0	0	0	0
118	22	1	1	0	0	0	0	0
98	20	3	1	0	0	0	0	0
100	23	1	0	0	0	0	0	0
110	24	1	1	0	0	0	0	0
83	24	1	1	0	0	0	0	0
84	22	3	1	0	0	0	0	0
84	22	3	1	0	0	0	0	0
110	21	1	0	0	0	0	0	0
80	20	1	0	0	0	0	0	0
96	22	3	1	0	0	1	0	0
96	18	1	0	0	0	0	0	0
82	22	1	1	0	0	0	0	0
112	24	1	0	0	0	0	0	0
70	18	1	0	0	0	0	0	1
109	25	1	1	0	0	0	0	0
86	20	1	0	0	0	1	0	0
101	22	1	1	0	0	1	0	0
86	24	1	1	0	0	0	0	0
90	20	1	1	0	0	0	0	0
87	21	1	0	0	0	1	0	0
86	20	1	1	0	0	0	0	0
89	22	3	1	0	0	0	0	0
86	20	1	0	0	0	0	0	0
90	20	2	1	0	0	0	0	0
90	20	1	0	0	0	0	0	0
85	20	1	0	0	0	0	0	0
100	22	3	0	0	0	0	0	0
115	22	1	0	0	0	0	0	0
100	23	1	1	0	0	0	0	0
125	24	2	1	0	0	0	0	0
90	20	1	1	0	0	0	0	0
120	22	3	0	0	0	0	0	0
86	18	3	0	0	0	0	0	0
98	22	3	1	0	0	0	0	0
111	24	3	0	0	0	0	0	0
98	17	1	0	0	0	0	0	0
92	20	1	1	0	0	0	0	0
92	20	3	0	0	1	0	0	0
78	18	2	0	0	0	0	0	0
82	19	1	1	0	0	0	0	1
90	20	1	1	0	0	0	0	0
70	20	1	1	0	0	0	0	0
80	20	3	0	0	0	0	0	0
80	16	1	1	0	0	0	0	0
82	20	1	1	0	0	1	0	0
76	20	3	1	0	0	0	0	0
130	24	3	0	0	0	0	0	0

104	25	1	0	0	0	0	0	0
76	22	1	1	0	0	0	0	0
100	23	3	0	0	0	0	0	0
105	20	3	1	0	0	0	0	0
112	18	1	0	0	0	1	0	0
80	18	1	1	0	0	0	0	0
86	20	1	1	0	0	0	0	0
104	23	1	0	0	0	0	0	0
108	18	1	1	0	0	0	0	0
85	20	3	1	0	0	0	0	0
88	22	3	1	0	0	0	0	0
86	20	1	1	0	0	0	0	0
82	18	1	0	0	0	0	0	0
88	22	2	0	0	0	1	0	0
92	22	3	1	0	0	1	0	0
76	18	3	1	0	0	0	0	0
86	28	1	0	0	0	0	0	0
102	21	1	0	0	0	0	0	0
80	20	1	0	0	0	0	0	0
118	30	1	0	0	0	0	0	0
76	20	1	1	0	0	0	0	0
70	20	3	1	0	0	0	0	0
84	20	1	1	0	0	0	0	0
98	23	3	0	0	0	1	0	0
80	22	3	1	0	0	0	0	0
82	18	1	0	0	0	0	1	0
96	22	3	0	0	0	0	0	0
90	22	1	0	0	0	1	0	0
90	23	1	1	0	0	0	0	0
106	21	1	1	0	0	0	0	0
77	20	1	1	0	0	0	0	0
62	18	1	0	0	0	1	0	0
84	20	1	1	0	0	0	0	0
76	20	1	1	0	0	0	0	0
100	22	1	0	0	0	0	0	0
76	24	1	1	0	0	0	0	0
84	20	3	1	0	0	0	0	0
86	20	1	1	0	0	0	0	0
105	21	1	1	0	0	0	0	0
102	20	1	1	0	0	0	0	0
92	20	1	1	0	0	1	0	0
72	20	1	1	0	0	0	0	0
118	25	1	1	0	0	0	0	0
64	20	2	1	0	0	0	0	0
96	24	3	0	0	0	0	0	0
94	30	1	1	0	0	0	0	0
74	22	1	1	0	0	0	0	0
66	18	1	0	0	0	0	0	0
80	19	1	1	0	0	0	0	0
78	20	1	1	0	0	0	0	0
60	20	1	1	0	0	0	0	0
68	21	1	1	0	0	0	0	0
92	22	1	1	0	0	0	0	0
96	20	1	1	0	0	0	0	0

65	20	1	0	0	0	1	0	0
60	20	1	1	0	0	0	0	0
88	20	1	1	0	0	0	0	0
98	20	1	1	0	0	0	0	0
86	22	1	1	0	0	0	0	0
80	20	1	1	0	0	0	0	0
80	22	1	1	0	0	1	0	0
90	20	1	1	0	0	0	0	0
95	24	3	1	0	0	0	0	0
58	22	1	1	0	0	0	0	0
100	20	1	0	0	0	1	0	0
80	20	1	1	0	0	0	0	0
82	20	1	1	0	0	0	0	0
92	22	1	1	0	0	0	0	0
75	20	1	1	0	0	0	0	0
82	20	1	0	0	1	0	0	0
72	18	1	1	0	0	1	0	0
88	25	1	1	0	0	0	0	0
80	20	1	1	0	0	0	0	0
90	22	1	0	0	0	0	0	0
88	20	1	1	0	0	0	0	0
69	20	1	1	0	0	0	0	0
100	20	1	1	0	0	0	0	0
51	18	1	1	0	0	0	0	0
88	20	1	1	0	0	0	0	0
105	26	1	1	0	0	0	0	0
90	20	1	1	0	0	0	0	0
92	20	3	0	0	0	0	0	0
60	18	3	0	1	0	0	0	0
86	18	1	1	0	0	0	0	0
120	18	3	1	0	0	0	0	0
88	23	1	1	0	0	0	0	0
78	20	1	1	0	0	0	0	0
125	22	1	1	0	0	1	0	0
86	21	1	0	0	0	0	0	0
167	27	1	1	0	0	0	0	0
161	32	2	0	0	0	0	0	0
88	18	1	1	0	0	0	0	0
86	20	1	1	0	0	0	0	0
74	22	1	1	0	0	0	0	0
89	22	1	1	0	0	0	0	0
84	21	1	0	0	0	1	0	0
80	19	1	1	0	0	0	0	0
86	20	1	0	0	0	0	1	0
78	20	1	0	0	0	0	0	0
96	21	1	0	0	0	1	0	0
85	21	1	1	0	0	0	0	0
84	22	1	1	0	0	1	0	0
82	22	3	1	0	0	0	0	0
84	22	1	1	0	0	0	0	0
85	21	1	1	0	0	0	0	0
80	22	1	1	0	0	0	0	0
90	22	1	1	0	0	0	0	0
85	21	1	1	0	0	0	0	0

78	18	2	1	1	0	0	0	0
90	22	1	1	0	0	0	0	0
98	21	1	0	0	0	0	0	0
76	19	1	1	1	0	1	0	0
100	20	3	0	0	0	0	0	0
84	24	1	0	0	0	0	0	0
92	20	1	1	0	0	0	0	0
98	20	2	1	0	0	0	0	0
94	22	1	1	0	0	0	0	0
83	20	1	1	0	0	0	0	0
108	22	1	0	0	0	0	0	0
100	22	1	1	0	0	0	0	0
92	20	2	1	0	0	0	0	0
88	19	1	1	0	0	0	0	0
88	20	1	0	0	0	1	0	0
80	20	1	0	0	0	1	0	0
90	22	1	0	0	0	1	0	0
107	18	2	0	0	0	0	0	0
86	23	1	0	1	0	0	0	0
86	20	1	1	0	0	0	0	0
80	22	1	1	0	0	0	0	0
80	20	1	1	0	0	0	0	0
118	22	3	1	0	0	0	0	0
94	22	1	1	0	0	0	0	0
100	23	1	0	0	0	1	0	0
90	21	1	1	0	0	0	0	0
100	20	1	1	0	0	0	0	0
83	18	1	1	0	0	0	0	0
116	21	1	0	0	0	0	0	0
83	22	1	1	0	0	0	0	0
96	22	1	1	0	0	0	0	0
100	23	1	1	0	0	0	0	0
92	22	1	1	0	0	0	0	0
96	22	1	1	0	0	0	0	0
96	22	1	0	0	0	0	0	0
100	22	1	1	0	0	0	0	0
80	20	1	0	0	0	0	0	0
101	20	1	0	0	0	0	0	0
84	18	1	0	0	0	0	0	0
100	20	3	1	0	0	0	0	0
78	20	3	1	0	0	1	0	0
86	20	1	1	0	0	0	0	0
75	18	1	1	0	0	1	0	0
100	22	1	1	0	0	0	0	0
90	20	1	0	0	0	0	0	0
115	20	1	0	0	0	0	0	0
100	23	1	0	0	0	1	0	0
72	18	1	0	0	0	0	0	0
83	21	1	1	0	0	0	0	0
80	18	2	0	0	0	0	0	0
150	35	2	0	0	0	0	0	0
70	18	1	0	0	0	1	0	0
83	18	1	1	0	0	0	0	0
68	19	1	1	0	0	0	0	0

83	17	1	1	0	0	0	0	0
98	20	1	1	0	0	0	0	0
84	20	2	1	0	0	0	0	0
83	24	1	1	0	0	0	0	0
86	21	1	0	0	0	0	1	0
100	21	1	1	0	0	0	0	0
80	22	1	1	0	0	0	0	0
84	20	1	1	0	0	0	0	0
86	20	3	1	0	0	0	0	0
96	22	1	1	0	0	0	0	0
102	20	1	1	0	0	0	0	0
100	21	3	1	0	0	0	0	0
85	23	1	1	0	0	0	0	0
88	20	1	1	0	0	0	0	0
83	20	1	1	0	0	0	0	0
70	23	1	1	0	0	0	0	0
91	20	1	1	0	0	0	0	0
75	18	1	1	0	0	0	0	0
70	23	1	1	0	0	0	0	0
85	22	2	1	0	0	0	0	0
98	19	3	0	0	0	0	0	0
100	20	1	1	0	0	0	0	0
78	18	1	1	0	0	1	0	0
105	21	2	1	0	0	0	0	0
100	22	1	1	0	0	0	0	0
62	21	1	1	0	0	0	0	0
110	21	1	0	0	1	0	0	0
85	22	1	1	0	0	0	0	0
85	20	1	1	0	0	0	0	0
100	23	2	1	0	0	0	0	0
85	20	1	0	0	0	0	1	0
98	21	1	1	0	0	0	0	0
102	23	1	1	0	0	0	0	0
90	21	1	1	0	0	0	0	0
101	22	1	1	0	0	0	0	0
88	21	1	1	0	0	0	0	0
102	22	1	1	0	0	0	0	0
70	20	1	1	0	0	1	0	0
82	22	1	1	0	0	0	0	0
82	22	1	1	0	0	0	0	0
89	22	2	1	0	0	0	0	0
100	22	1	1	0	0	1	0	0
100	22	1	1	0	0	0	0	0
100	25	1	1	0	0	0	0	0
108	26	1	0	0	0	1	0	0
95	23	1	1	0	0	0	0	0
80	21	1	1	0	0	0	0	0
100	22	1	1	0	0	0	0	0
102	21	1	1	1	0	0	0	0
102	22	1	1	0	0	0	0	0
72	19	1	1	0	0	0	0	0
86	19	1	1	0	0	1	0	0
82	22	1	1	0	0	0	0	0
100	23	1	1	0	0	0	0	0

86	21	1	1	0	0	1	0	0
96	21	1	1	0	0	0	0	0
92	20	1	1	0	0	0	0	0
86	20	1	1	0	0	0	0	0
81	19	1	1	0	0	0	0	0
80	22	1	1	1	0	0	0	0
74	20	1	0	0	0	0	0	0
100	22	1	0	0	0	0	0	0
85	20	1	0	0	0	0	0	0
78	21	1	0	0	0	0	0	0
76	20	1	1	0	0	0	0	0
123	21	1	1	1	0	0	0	0
98	18	3	1	0	0	0	0	0
76	18	1	1	0	0	0	0	0
108	20	1	1	0	0	0	0	0
64	18	1	1	0	0	0	0	0
86	20	1	0	0	0	0	0	0
78	20	2	1	0	0	0	0	0
80	20	1	1	0	0	0	0	0
89	20	1	1	0	0	0	0	0
67	20	1	1	0	0	0	0	0
88	19	1	1	0	0	0	0	0
84	20	3	0	0	0	0	0	0
88	21	1	1	0	0	0	0	0
110	23	1	1	0	0	0	0	0
80	19	1	0	1	0	0	0	0
90	22	1	0	0	0	0	0	0
104	21	1	0	0	0	0	0	0
80	22	1	1	0	0	0	0	0
104	20	3	0	0	0	0	1	0
84	22	1	1	0	0	0	0	0
122	28	1	1	0	0	0	0	0
120	28	1	1	0	0	0	0	0
80	20	1	1	0	0	1	0	0
130	22	1	1	0	0	0	0	0
78	20	1	1	1	0	0	0	0
62	20	1	1	0	0	1	0	0
102	23	1	1	0	0	0	0	0
84	21	2	1	0	0	0	0	0
82	16	1	1	0	0	0	0	0
70	20	1	1	0	0	0	0	0
72	18	2	0	0	0	0	0	0
72	20	1	1	0	0	0	0	0
100	22	1	1	0	0	0	0	0
81	20	1	1	0	0	0	0	0
66	13	1	1	0	0	0	0	0
70	22	1	1	0	0	0	0	0
98	21	1	1	0	0	0	0	0
93	25	3	1	0	0	0	0	0
93	23	1	1	0	0	0	0	0
97	20	3	0	0	0	1	0	0
90	21	1	1	0	0	0	0	0
115	24	3	0	0	0	0	0	0
85	22	2	1	0	0	0	0	0

90	24	1	1	0	0	0	0	0
86	20	1	1	0	0	0	0	0
80	22	1	1	0	0	0	0	0
62	20	1	1	0	0	0	0	0
80	20	1	1	0	0	0	0	0
85	22	1	0	0	0	0	0	0
90	20	1	1	0	0	0	0	0
85	22	1	1	0	0	0	0	0
62	20	1	1	0	0	0	0	0
86	25	1	1	0	0	0	0	0
98	21	1	0	0	1	1	0	0
88	22	1	1	0	0	0	0	0
110	24	1	0	0	0	0	0	0
88	22	1	1	0	0	0	0	0
98	19	1	1	0	0	0	0	0
94	22	1	1	0	0	0	0	0
88	23	1	1	0	0	0	0	0
82	22	1	1	0	0	0	0	0
88	21	1	1	0	0	0	0	0
53	18	1	1	0	0	0	0	0
84	20	1	0	0	0	0	0	0
96	23	2	1	0	0	0	0	0
83	22	1	1	0	0	0	0	0
84	22	1	0	0	0	0	0	0
86	21	1	1	0	0	0	0	0
84	22	3	1	0	0	0	0	0
112	22	1	1	0	0	0	0	0
101	23	2	1	0	0	0	0	0
88	20	2	1	0	0	0	0	0
85	22	1	1	0	0	0	0	0
86	22	1	1	0	0	0	0	0
90	20	1	1	0	0	0	0	0
92	20	1	0	1	0	0	0	0
92	23	1	0	0	0	0	0	0
116	28	1	1	0	0	0	0	0
88	26	1	0	0	0	0	0	0
110	30	1	1	0	0	0	0	0
85	22	1	1	0	0	0	0	0
96	21	1	0	1	0	0	0	0
84	22	1	1	0	0	0	0	0
85	18	1	1	0	0	0	0	0
84	22	1	1	0	0	0	0	0
92	23	1	1	0	0	0	0	0
68	18	1	0	0	0	0	0	0
79	21	1	0	0	0	0	0	0
56	20	1	1	0	0	0	0	0
85	22	3	1	0	0	0	0	0
80	24	1	1	0	0	0	0	0
85	22	1	0	0	0	1	0	0
85	20	1	1	0	0	0	0	0
85	22	3	1	0	0	0	0	0
70	20	1	0	0	0	0	0	0
85	22	1	1	0	0	0	0	0
98	23	1	1	0	0	0	0	0

98	25	1	1	0	0	0	0	0
80	18	3	1	0	0	0	0	0
80	23	1	1	0	0	0	0	0
68	20	3	1	0	0	0	0	0
100	20	1	1	0	0	0	0	0
90	21	1	1	0	0	0	0	0
96	23	1	1	0	0	0	0	0
70	20	1	1	1	0	1	0	0
74	19	3	0	0	0	1	0	0
82	21	3	1	0	0	1	0	0
89	20	1	1	1	1	1	0	0
82	22	1	1	0	1	0	0	0
62	21	3	1	1	1	1	0	0
86	21	1	1	0	0	0	0	0
86	20	1	1	0	1	0	0	0
86	21	1	0	0	0	0	0	0
82	18	1	0	0	1	0	0	0
120	30	1	0	0	1	1	0	0
70	22	1	1	0	0	0	0	0
80	18	3	1	0	1	1	0	1
90	24	1	0	0	0	0	0	0
98	25	1	1	0	0	0	0	0
95	25	1	1	0	1	1	0	0
92	20	1	1	0	1	0	0	0
86	20	1	1	0	0	0	0	0
90	23	1	1	0	1	1	0	0
126	19	3	1	0	0	0	0	0
94	22	3	1	0	0	0	0	0
92	22	1	1	0	0	1	0	0
86	22	1	1	0	0	0	0	0
80	20	1	1	0	0	0	0	0
97	24	1	1	0	0	1	0	0
85	21	1	1	0	1	0	0	0
82	22	2	1	0	1	1	0	0
71	20	1	1	0	1	1	0	0
82	20	1	1	0	1	0	0	0
82	20	1	1	0	1	0	0	0
82	22	1	1	0	0	1	0	0
88	22	1	1	0	0	0	0	0
98	20	3	1	0	1	0	0	0
84	21	1	1	0	0	0	0	0
92	22	1	1	0	0	0	0	0
102	22	1	1	0	0	0	0	0
78	20	1	0	0	1	0	0	0
89	22	1	1	0	1	0	0	0
88	22	1	1	0	0	0	0	0
98	22	3	1	0	0	1	0	0
80	22	1	1	0	0	0	0	0
85	22	1	1	0	0	0	0	0
90	20	1	1	0	0	0	0	0
70	20	1	1	0	0	1	0	0
102	20	1	1	0	0	1	0	0
104	20	1	1	0	1	0	0	0
105	22	1	1	0	1	0	0	0

116	20	1	1	0	0	1	0	0
110	26	1	1	0	0	1	0	0
81	18	1	1	0	1	0	0	0
84	23	1	1	0	0	0	0	0
88	22	1	1	0	0	0	0	0
92	22	1	1	0	1	0	0	0
80	22	1	1	0	1	0	0	0
84	20	1	1	0	0	0	0	0
90	21	1	1	0	1	1	0	0
82	21	1	1	0	1	0	0	0
100	24	1	1	0	0	1	0	0
88	20	1	0	0	0	0	0	0
92	20	1	1	0	0	0	0	0
88	20	1	1	0	1	0	0	0
86	21	1	0	0	0	0	0	0
96	20	1	1	0	0	0	0	0
84	20	1	1	0	0	0	0	0
92	21	1	1	1	1	1	0	0
95	21	1	1	0	1	1	0	0
92	21	1	1	0	0	1	0	0
90	22	1	1	0	1	0	0	0
92	20	1	0	0	0	0	0	0
90	23	1	1	0	1	0	0	0
90	20	1	1	0	1	1	0	0
98	23	1	1	0	0	0	0	0
81	20	1	1	0	1	1	0	0
90	22	1	1	0	0	1	0	0
96	20	1	1	0	0	1	0	0
96	20	1	1	0	0	0	0	0
85	22	1	0	0	1	0	0	0
94	23	3	1	0	1	1	0	0
123	18	3	1	0	0	1	0	0
74	20	1	1	0	0	0	0	0
102	22	1	0	0	0	0	0	0
84	20	1	1	0	0	0	0	0
74	20	1	1	0	0	0	0	0
113	21	1	0	0	0	0	0	0
96	21	1	1	0	0	0	0	0
100	24	1	1	0	0	0	0	0
86	18	2	1	1	1	1	0	0
85	23	1	1	1	0	0	0	0
88	25	1	1	0	0	0	0	0
66	25	3	0	0	0	1	0	0
75	20	1	0	0	0	0	0	0
110	21	2	1	1	0	0	0	0
74	20	2	1	0	0	1	0	0
112	22	1	1	0	1	1	0	0
104	20	1	1	0	1	0	0	0
93	20	1	1	0	0	0	0	0
98	22	2	0	0	0	0	0	0
92	23	1	1	0	0	0	0	0
84	22	1	1	0	0	0	0	0
84	24	1	1	0	0	0	0	0
105	20	1	0	0	0	0	0	0

92	20	3	0	0	0	0	0	0
86	20	1	0	0	0	0	0	0
95	18	1	1	0	0	1	0	0
90	22	1	1	0	0	0	0	0
86	21	3	1	0	0	1	0	0
86	20	3	1	0	0	0	0	0
89	22	1	1	0	0	0	0	0
95	21	1	1	0	0	0	0	0
84	21	1	1	1	1	1	0	0
82	20	1	1	0	0	0	0	0
85	22	1	1	0	0	1	0	0
83	23	1	1	0	0	0	0	0
84	22	3	1	0	0	0	0	0
120	20	3	0	0	0	0	0	0
86	22	1	0	0	1	1	0	0
92	21	1	1	0	0	0	0	0
78	22	2	1	0	1	1	0	0
85	22	3	1	0	0	0	0	0
88	22	1	1	0	1	1	0	0
82	21	1	1	1	0	0	0	0
83	21	1	1	0	1	0	0	0
90	20	1	0	0	1	1	0	0
82	21	1	1	0	1	0	0	0
84	18	1	1	0	0	0	0	0
98	23	3	1	0	1	1	0	0
80	20	1	1	0	0	0	0	0
84	22	1	1	1	1	0	0	0
86	23	2	1	0	1	1	0	0
87	21	1	1	1	1	1	0	0
76	22	1	1	0	1	0	0	0
88	25	3	1	1	1	1	0	0
98	22	1	1	0	0	0	0	0
82	22	1	1	0	0	0	0	0
74	20	1	1	1	0	0	0	0
85	22	1	0	0	0	1	0	0
78	21	1	1	1	1	1	0	0
88	22	3	1	0	1	0	0	0
92	20	1	1	0	1	1	0	0
86	25	2	1	0	1	1	0	0
110	23	1	0	0	0	1	0	0
76	20	1	1	0	1	0	0	0
84	20	1	1	0	1	0	0	0
92	22	1	1	0	1	0	0	0
92	20	1	1	0	1	0	0	0
88	23	1	0	1	0	0	0	0
68	20	3	0	0	0	0	0	0
88	20	1	1	0	1	0	0	0
105	20	1	1	0	1	1	0	0
90	23	1	1	0	1	1	0	0
82	22	1	0	0	0	0	0	0
84	21	1	0	0	0	0	0	0
90	19	1	1	0	1	0	0	0
80	20	1	1	0	0	1	0	0
84	20	1	1	0	0	0	0	0

89	22	1	1	0	0	1	0	0
88	22	1	1	0	1	0	0	0
92	24	1	0	0	0	0	0	0
84	20	1	1	0	0	0	0	0
85	23	1	1	0	1	0	0	1
86	22	1	0	0	0	1	0	0
84	20	1	1	0	1	1	0	0
86	21	1	0	0	0	0	0	0
108	20	1	0	0	0	1	0	0
81	20	1	1	0	0	1	0	0
116	21	2	1	0	0	1	0	0
92	21	1	1	0	1	1	0	0
96	22	3	1	0	1	0	0	0
82	20	1	0	0	0	0	0	0
110	27	3	1	0	1	0	0	0
100	23	3	0	0	0	1	0	0
82	20	3	1	1	0	0	0	0
85	26	1	1	0	0	0	0	0
92	23	1	0	0	0	0	0	0
96	24	1	0	0	0	1	0	0
80	18	3	0	0	1	0	0	0
88	19	2	0	0	0	0	0	0
86	22	1	1	0	0	0	0	0
88	23	2	1	0	0	1	0	0
141	29	1	0	0	0	0	0	0
88	21	3	1	0	0	0	0	0
76	22	1	1	0	0	0	0	0
80	23	1	1	0	0	0	0	0
90	22	1	1	0	0	0	0	0
82	21	1	1	0	1	1	0	0
69	20	1	0	0	0	0	0	0
90	20	1	1	0	1	1	0	1
96	21	1	1	0	0	0	0	0
88	21	1	0	0	1	1	0	0
95	21	1	1	0	0	0	0	0
88	22	1	1	0	0	0	0	0
99	23	1	1	0	1	1	0	0
90	24	1	1	0	0	1	0	0
96	22	3	1	0	0	0	0	0
96	24	1	1	1	0	0	0	0
104	23	3	0	0	0	1	0	0
89	21	3	1	0	1	1	0	0
88	21	1	1	0	0	0	0	0
75	23	1	1	1	1	0	0	0
109	21	1	1	0	0	1	0	0
86	21	1	1	0	0	0	0	0
85	21	1	1	0	0	0	0	0
84	18	1	1	0	0	1	0	0
72	21	1	1	0	0	0	0	0
96	21	1	1	0	1	0	0	0
102	21	1	1	1	0	0	0	0
90	24	1	1	0	0	1	0	0
110	22	1	1	1	1	1	0	0
88	22	1	1	0	0	1	0	0

80	20	1	1	1	1	1	0	0
77	18	1	1	0	1	0	0	0
60	22	1	1	0	0	1	0	0
84	22	1	1	1	1	1	0	0
100	23	2	0	0	0	0	0	0
92	21	3	1	1	1	1	0	0
90	25	3	1	0	1	1	0	0
93	22	1	1	0	1	0	0	0
88	20	1	1	0	0	0	0	0
92	20	1	1	0	0	1	0	0
100	18	1	1	0	1	1	0	0
95	23	1	1	1	0	0	0	0
92	20	1	1	0	1	0	0	0
86	23	1	0	0	1	1	0	0
94	21	1	1	0	0	1	0	0
78	20	1	1	1	1	0	0	0
92	23	1	1	0	1	1	0	0
122	22	1	0	0	0	0	0	0
104	22	2	1	0	1	1	0	0
84	20	1	1	0	0	0	0	0
80	22	3	0	0	0	0	0	0
88	21	2	1	1	1	0	0	0
130	29	1	1	0	0	1	0	0
86	20	1	1	0	1	0	0	0
88	21	3	0	0	0	1	0	0
90	20	1	1	0	1	1	0	0
67	21	3	0	0	0	0	0	0
90	20	1	1	0	0	0	0	0
92	21	1	1	0	1	0	0	0
74	21	1	1	0	0	0	0	0
83	20	1	1	0	0	0	0	0
76	20	1	1	0	0	1	0	0
101	22	1	1	0	0	0	0	0
83	21	1	1	0	0	0	0	0
96	20	3	1	0	0	1	0	0
102	23	1	1	0	0	0	0	0
94	22	1	0	0	0	0	0	0
90	20	3	1	0	0	0	0	0
88	21	1	1	0	0	1	0	0
82	20	1	1	0	0	0	0	0
95	24	1	1	0	1	0	0	0
90	20	1	0	0	0	0	0	0
76	19	1	0	0	0	0	0	0
86	22	1	1	0	1	0	0	0
98	22	1	1	0	0	0	0	0
92	21	1	1	0	1	1	0	0
98	22	1	0	1	0	1	0	0
112	20	2	1	0	0	0	0	0
81	20	1	1	0	0	0	1	0
86	20	1	1	0	1	0	0	0
86	22	1	1	0	0	1	0	0
98	22	1	1	0	0	0	0	0
104	24	1	1	0	0	0	0	0
87	21	1	1	0	0	1	0	0

100	20	1	0	0	0	0	0	0
108	21	3	1	0	0	0	0	0
92	25	1	1	0	0	0	0	0
121	24	1	1	1	1	1	0	0
102	22	3	1	0	0	0	0	0
102	23	1	0	0	0	0	0	0
91	21	1	1	0	0	0	0	0
78	20	1	1	0	1	1	0	0
95	21	1	1	0	0	0	0	0
91	20	1	1	0	1	0	0	0
90	22	1	1	0	0	0	0	0
86	22	1	1	0	1	1	0	0
82	20	1	1	0	1	1	0	0
88	20	1	1	0	0	0	0	0
110	20	1	1	0	0	1	0	0
92	20	1	0	0	0	0	0	0
85	20	1	1	1	1	0	0	0
90	24	1	1	0	0	1	0	0
65	25	1	1	0	0	0	0	0
89	20	1	1	0	1	1	0	0
84	21	1	1	0	1	1	0	0
83	21	1	1	1	0	0	0	0
120	20	2	1	0	1	0	0	0
86	21	1	1	1	0	0	0	0
95	20	1	1	0	1	0	0	0
86	23	1	1	0	0	0	0	0
84	21	1	1	0	0	1	0	0
88	21	1	1	0	0	0	0	0
92	23	1	1	0	1	1	0	0
91	21	1	1	0	1	0	0	0
97	20	1	1	0	1	1	0	0
90	20	1	1	0	1	0	0	0
82	19	1	1	0	1	1	0	0
87	22	3	1	0	0	1	0	0
90	22	1	1	0	0	0	0	0
91	22	1	1	0	0	1	0	0
65	20	1	0	0	0	1	0	0
97	22	1	0	0	0	0	0	0
88	21	1	1	0	1	0	0	0
64	16	1	1	0	0	0	0	0
80	21	1	1	0	1	0	0	0
89	20	1	1	0	1	1	0	0
59	21	3	1	0	0	1	0	0
88	20	1	1	0	0	0	0	0
85	22	1	1	0	0	1	0	0
85	21	1	1	0	1	1	0	0
89	20	3	1	0	1	0	0	0
89	22	1	1	0	1	1	0	0
84	20	1	1	0	0	0	0	0
132	24	3	0	0	0	1	0	0
88	21	3	1	0	0	0	0	0
64	19	1	1	0	1	1	0	0
86	21	1	1	0	0	1	0	0
82	20	3	1	0	0	0	0	0

84	21	1	1	0	1	0	0	0
88	22	3	1	1	1	0	0	0
89	21	1	1	0	0	0	0	0
84	20	1	1	1	1	1	0	0
72	18	1	1	0	0	1	0	0
82	20	1	1	0	1	0	0	0
91	22	1	1	1	1	0	0	0
97	21	1	1	0	1	0	0	0
80	21	1	1	0	1	0	0	0
101	24	3	1	0	0	0	0	0
88	21	1	1	1	1	0	0	0
80	20	1	1	0	0	1	0	0
84	20	1	1	0	1	1	0	0
86	22	3	1	0	0	0	0	0
95	22	1	1	0	1	1	0	0
78	20	1	1	0	0	1	0	0
78	20	2	1	1	1	0	0	0
89	24	3	0	0	1	0	0	0
84	18	2	1	1	1	1	0	0
89	23	1	1	0	1	1	0	0
90	21	1	1	0	0	0	0	0
80	23	1	1	0	1	1	0	0
98	22	1	1	0	1	1	0	0
84	21	1	1	0	0	0	0	0
94	22	1	1	1	1	1	0	0
112	21	1	0	0	0	0	0	0
93	20	1	1	0	0	0	0	0
89	23	1	1	0	0	1	0	0
78	21	1	1	0	1	1	0	0
90	22	3	1	0	0	0	0	0
82	20	1	1	0	1	1	0	0
100	20	1	1	0	0	0	0	0
86	20	3	1	0	1	0	0	0
94	23	1	1	0	0	0	0	0
89	22	1	1	0	0	0	0	0
89	20	1	1	0	0	0	0	0
98	22	3	1	0	0	1	0	0
110	20	1	1	0	0	0	0	0
80	21	1	0	0	0	0	0	0
80	21	1	1	0	1	1	0	0
84	20	1	1	0	0	1	0	0
87	21	1	1	0	0	1	0	0
86	21	1	1	0	1	0	0	0
96	21	1	1	0	1	1	0	0
98	23	1	1	0	0	0	0	0
108	22	1	1	0	0	0	0	0
102	22	1	1	0	0	1	0	0
86	21	1	1	0	0	0	0	0
70	18	1	1	0	0	0	0	0
104	22	1	1	0	1	0	0	0
105	21	1	1	0	1	0	0	0
88	23	1	1	0	0	0	0	0
100	20	1	1	0	0	0	0	0
84	22	1	1	0	0	0	0	0

88	20	1	1	0	1	1	0	0
107	20	1	1	1	1	0	0	0
89	23	1	1	0	0	0	0	0
140	36	1	1	0	0	1	0	0
64	20	1	1	0	0	0	0	0
96	21	1	1	0	1	0	0	0
89	21	1	1	0	0	0	0	0
99	20	1	1	0	0	0	0	0
63	21	1	1	0	0	0	0	0
128	20	1	1	0	0	0	0	0
80	23	1	1	0	1	1	0	0
98	20	1	0	1	0	0	0	0
104	20	1	1	0	1	1	0	0
96	21	1	1	0	0	0	0	0
100	21	1	1	0	0	1	0	0
72	22	1	1	0	1	1	0	0
98	18	1	0	0	0	1	0	0
112	21	2	1	0	0	0	0	0
82	20	1	1	0	0	0	0	0
80	20	2	1	0	0	0	0	0
96	20	1	1	0	0	1	0	0
108	22	2	1	0	1	1	0	0
82	20	1	1	0	0	0	0	0
110	21	1	1	0	1	0	0	0
90	22	3	1	0	1	1	0	0
102	20	2	1	0	1	1	0	0
92	22	2	0	0	0	0	0	0
98	23	1	1	0	0	0	0	0
70	20	2	1	0	0	0	0	0
102	20	1	1	0	0	1	0	0
98	23	1	1	0	1	1	0	0
100	20	1	1	0	1	0	0	0
95	20	1	1	0	1	1	0	0
102	21	1	1	1	0	0	0	0
82	21	2	1	0	0	1	0	0
87	20	1	1	0	0	0	0	0
99	20	1	1	0	0	0	0	0
96	21	1	1	0	0	1	0	0
98	21	1	1	0	0	0	0	0
98	20	1	1	0	0	0	0	0
90	20	1	1	0	0	0	0	0
82	20	1	1	0	0	0	0	0
98	24	1	1	0	1	0	0	0
75	18	1	1	0	0	0	0	0
92	23	1	1	0	1	1	0	0
105	22	1	1	0	1	0	0	0
94	22	1	1	0	1	1	0	0
77	21	1	0	0	0	1	1	0
92	23	1	1	0	0	0	0	0
95	22	1	1	0	0	1	0	0
108	22	1	1	0	0	0	0	0
100	22	3	1	0	0	0	0	0
95	22	1	1	0	0	0	0	0
92	22	3	1	0	0	0	0	0

114	19	2	1	0	1	1	0	0
89	20	3	1	0	0	0	0	0
110	24	3	0	0	0	0	0	0
90	20	1	1	0	0	1	0	0
94	21	1	1	0	0	1	0	0
110	21	1	1	0	1	1	0	0
113	22	1	1	0	0	0	0	0
103	21	1	1	1	1	0	0	0
82	21	1	1	0	0	0	0	0
67	20	3	0	0	0	0	1	0
82	24	1	1	0	0	0	0	0
112	22	1	1	1	1	1	0	0
103	21	1	1	0	0	0	0	0
95	21	2	0	0	0	0	0	0
98	21	1	1	0	0	0	0	0
90	20	1	1	0	0	0	0	0
86	20	1	1	0	0	0	0	0
112	22	1	1	0	0	1	0	0
108	22	1	1	0	0	0	0	0
78	20	1	1	0	0	1	0	0
80	21	1	1	0	0	0	0	0
90	22	1	1	0	0	0	0	0
80	20	1	1	0	0	0	0	0
96	20	1	1	1	1	1	0	0
98	21	1	1	0	1	0	0	0
103	21	1	1	0	1	0	0	0
92	24	1	1	0	1	0	0	0
90	20	1	1	0	0	0	0	0
103	21	1	1	0	0	0	0	0
98	21	1	0	0	0	1	1	0
95	22	1	1	0	1	1	0	0
90	20	1	1	0	1	1	0	0
81	20	1	1	0	1	0	0	0
93	20	1	1	0	1	0	0	0
88	18	1	1	0	0	0	0	0
88	20	1	1	0	0	0	0	0
74	21	1	1	1	1	1	0	0
95	25	1	1	0	1	1	0	0
90	22	1	1	0	0	0	0	0
97	22	1	1	0	1	0	0	0
97	22	1	0	0	0	0	0	0
83	22	1	1	0	0	0	0	0
88	22	1	1	0	1	1	0	0
85	18	1	1	0	0	0	1	0
86	22	1	1	0	1	0	0	0
82	22	1	1	0	0	0	0	0
87	23	1	1	0	0	1	0	0
88	22	1	1	1	1	1	0	0
82	22	1	1	1	1	0	0	0
123	19	1	1	0	0	0	0	0
96	22	1	1	0	0	1	0	0
87	22	1	1	0	1	1	0	0
92	21	1	1	1	0	0	0	0
93	20	1	0	0	0	0	0	0

96	21	1	1	0	1	0	0	0
88	20	1	1	0	1	1	0	0
76	20	1	1	0	1	0	0	0
82	25	3	0	0	1	1	0	0
109	22	1	1	0	1	1	0	0
97	20	1	1	0	1	1	0	0
102	18	1	1	0	1	1	0	0
100	19	3	1	0	0	0	0	0
72	21	1	1	0	0	0	0	0
100	21	1	1	0	0	0	0	0
99	22	1	1	0	1	1	0	0
80	21	2	1	0	0	0	0	0
76	20	1	1	0	0	0	0	0
89	21	1	1	0	1	1	0	0
90	21	1	1	0	0	0	0	0
106	21	1	1	1	1	1	0	0
88	21	1	1	0	0	0	0	0
92	23	1	1	0	0	0	0	0
96	26	1	1	0	0	1	0	0
89	18	1	0	0	0	1	1	0
80	20	1	1	0	0	0	0	0
109	22	1	1	0	0	1	0	0
69	20	1	1	1	1	1	0	0
80	22	1	1	0	1	0	0	0
87	21	1	1	0	1	1	0	0
90	20	1	1	0	0	0	0	0
95	32	1	0	0	0	0	0	0
89	21	1	1	0	0	1	0	0
83	22	1	1	1	0	0	0	0
82	20	1	0	0	0	0	0	0
107	21	1	1	0	0	0	0	0
88	20	1	1	0	1	1	0	0
90	20	1	1	0	0	0	0	0
89	21	1	1	0	0	0	0	0
84	20	1	1	0	0	1	0	0
92	24	1	0	1	0	0	0	0
84	20	1	1	0	0	0	0	0
110	28	1	0	0	0	0	0	0
82	18	1	1	0	1	0	0	0
89	21	1	1	0	0	0	0	0
89	22	1	0	0	1	1	0	0
88	21	1	1	0	1	0	0	0
79	20	1	1	0	1	0	0	0
86	21	1	1	0	0	0	0	0
90	20	1	1	0	0	1	0	0
92	20	1	0	0	0	0	0	0
90	20	1	1	0	0	0	0	0
98	20	1	1	0	1	0	0	0
73	22	1	1	0	0	1	0	0
90	20	1	1	0	1	0	0	0
105	21	1	1	0	0	0	0	0
82	20	1	1	0	0	1	0	0
108	22	1	1	0	0	1	0	0
92	22	1	1	0	0	0	0	0

88	23	1	1	0	1	1	0	0
86	21	1	1	0	0	0	0	0
80	20	1	1	0	0	0	0	0
90	22	1	1	1	1	0	0	0
90	20	1	1	0	1	1	0	0
89	21	1	1	0	0	0	0	0
90	20	1	1	0	0	0	0	0
90	22	1	1	0	1	1	0	0
97	21	1	0	1	0	0	0	0
109	22	1	1	1	1	1	0	0
89	18	1	1	0	0	0	0	0
97	23	1	1	0	1	1	0	0
80	20	1	1	0	0	0	0	0
92	22	1	1	0	1	0	0	0
67	22	1	1	0	1	1	0	0
72	20	1	1	0	1	1	0	0
88	21	1	1	1	1	1	0	0
88	21	1	1	0	0	1	0	0
85	20	1	0	0	0	0	0	0
104	23	1	1	0	0	0	0	0
87	22	1	1	0	0	0	0	0
92	22	1	1	1	1	0	0	0
92	22	1	1	0	0	0	0	0
98	23	1	1	0	0	1	0	0
92	21	1	1	0	0	1	0	0
89	20	3	0	0	0	1	0	0
91	22	1	1	0	1	0	0	0
90	20	1	1	0	1	1	0	0
92	21	1	1	0	1	0	0	0
90	21	1	1	0	0	0	0	0
88	21	2	1	0	1	0	0	0
74	20	1	1	0	1	0	0	0
78	20	1	1	0	1	0	0	0
88	20	1	1	0	0	1	0	0
108	22	1	1	0	0	0	0	0
76	18	3	1	0	0	1	0	0
89	21	1	0	0	0	1	1	0
74	20	1	1	0	0	1	0	0
86	21	1	1	0	1	0	0	0
93	20	1	1	0	1	1	0	0
74	20	1	1	0	0	0	0	0
87	20	1	1	0	1	1	0	0
90	21	1	1	0	0	0	0	0
90	20	1	1	1	1	0	0	0
80	24	1	1	0	0	1	0	0
113	20	1	1	0	0	0	0	0
98	21	1	1	0	1	0	0	0
80	20	1	1	0	0	0	0	0
96	20	2	1	1	1	1	0	0
88	24	1	1	0	1	0	0	0
90	20	1	1	0	1	0	0	0
85	22	1	1	0	1	1	0	0
78	21	1	1	1	1	0	0	0
82	20	1	1	0	1	0	0	0

89	22	3	1	0	0	0	0	0
83	20	1	1	0	1	1	0	0
118	22	1	1	0	0	1	0	0
82	20	1	1	0	0	1	0	0
96	20	1	1	1	1	0	0	0
86	20	1	1	0	1	1	0	0
70	20	1	1	0	1	0	0	0
86	22	1	1	0	1	0	0	0
74	20	1	1	0	1	1	0	0
88	20	1	1	0	0	0	0	0
85	20	1	1	0	1	1	0	0
105	20	1	1	0	1	0	0	0
90	28	1	1	0	1	0	0	0
90	23	1	1	0	0	0	0	0
96	22	1	1	0	1	0	0	0
94	20	1	1	0	1	0	0	0
81	20	1	1	0	0	0	0	0
89	23	1	0	0	0	0	0	0
78	23	1	0	0	0	1	0	0
94	21	1	0	0	0	0	0	0
92	16	1	1	0	0	0	0	0
96	21	1	1	0	0	1	0	0
80	20	1	1	1	1	1	0	0
116	20	1	0	1	0	1	0	0
119	21	1	0	0	1	1	0	0
100	24	1	1	1	0	0	0	0
84	20	1	1	0	0	0	0	0
88	20	3	1	0	0	0	0	0
89	21	3	1	0	0	0	0	0
86	20	1	1	0	0	0	0	0
84	20	1	1	0	1	1	0	0
105	20	1	1	0	1	1	0	0
90	20	1	1	0	0	0	0	0
73	20	1	0	0	0	0	0	0
95	20	3	1	0	0	1	0	0
90	18	1	1	1	1	0	0	0
90	21	3	0	0	0	0	0	0
89	20	2	1	0	1	0	0	0
82	20	3	1	0	0	0	0	0
85	21	1	0	0	0	0	0	0
88	22	1	1	0	0	0	0	0
86	22	1	1	0	0	0	0	0
74	19	1	1	0	0	0	0	0
91	22	1	1	0	1	0	0	0
76	19	3	0	0	0	0	0	0
95	21	1	1	0	1	1	0	0
68	19	1	1	0	1	0	0	0
68	18	1	0	1	0	0	0	0
98	23	1	1	0	0	0	0	0
62	20	1	1	0	1	0	0	0
90	22	1	1	1	0	0	0	0
83	21	1	1	0	0	0	0	0
81	19	1	0	0	0	0	0	0
97	26	3	1	0	0	1	0	0

95	21	1	1	0	1	0	0	0
90	21	3	1	0	0	0	0	0
121	23	1	1	0	1	1	0	0
94	23	1	1	1	0	1	0	0
93	23	2	1	0	1	0	0	0
103	22	1	1	0	0	1	0	0
88	21	1	1	1	1	0	0	0
93	21	1	1	0	1	1	0	0
82	18	2	1	0	1	0	0	0
118	20	1	1	0	0	0	0	0
105	20	1	0	0	0	1	0	0
91	20	1	1	0	0	0	0	0
90	21	1	1	0	0	0	0	0
78	20	1	0	0	0	0	0	0
100	22	1	1	0	0	0	0	0
107	21	1	1	0	0	0	0	0
70	19	3	1	0	0	0	0	0
84	21	2	1	1	0	0	0	0
88	21	1	0	0	0	0	0	0
76	21	2	1	0	0	0	0	0
100	20	3	1	0	0	0	0	0
92	21	1	1	0	0	0	0	0
86	20	3	1	0	1	0	0	0
76	20	1	1	0	0	0	0	0
102	21	3	1	0	0	1	0	0
100	22	3	1	0	0	0	0	0
102	23	1	1	1	0	0	0	0
100	20	2	1	0	1	0	0	0
106	20	3	1	0	0	0	0	0
73	20	3	1	0	0	1	0	0
95	22	1	1	0	0	1	0	0
84	23	1	1	0	1	0	0	0
141	22	1	1	0	0	1	0	0
89	22	1	1	0	0	0	0	0
90	20	1	1	0	0	1	0	0
92	21	1	1	0	0	1	0	0
110	24	3	1	0	0	0	0	0
85	21	1	1	0	0	0	0	0
153	22	1	1	1	1	0	0	0
112	22	1	1	0	1	1	0	0
101	20	1	1	0	0	1	0	0
71	20	1	1	0	0	0	0	0
88	23	1	1	0	0	0	0	0
92	21	1	1	0	0	0	0	0
122	22	3	1	0	0	0	0	0
89	20	1	1	0	0	1	0	0
66	18	1	1	0	0	0	0	0
85	20	1	1	0	1	0	0	0
85	23	1	1	1	0	0	0	0
82	20	1	1	0	0	0	0	0
78	20	1	1	1	0	1	0	0
80	20	1	1	1	0	0	0	0
84	21	1	0	0	0	1	0	0
109	20	3	0	0	0	0	0	0

104	26	1	0	0	0	0	0	0
86	21	2	1	0	0	0	0	0
105	23	1	1	0	1	1	0	0
85	21	1	1	0	1	1	0	0
88	23	1	1	0	0	1	0	0
91	20	3	1	0	0	1	0	0
114	21	1	0	0	0	0	0	0
96	22	1	1	0	1	0	0	0
97	23	1	1	0	1	0	0	0
92	20	1	1	1	1	1	0	0
98	22	1	1	0	0	1	0	0
83	20	1	0	0	0	0	0	0
110	21	1	1	0	0	0	0	0
106	24	1	0	0	0	0	0	0
87	22	1	1	0	1	1	0	0
83	20	1	1	0	0	0	0	0
91	20	3	1	0	0	0	0	0
82	22	1	1	0	0	0	0	0
102	22	1	1	0	0	0	0	0
98	20	1	1	0	0	1	0	0
90	21	1	1	0	0	1	0	0
92	22	1	1	0	1	0	0	0
108	24	1	1	0	0	1	0	0
85	21	1	1	0	1	1	0	0
103	23	1	1	0	0	1	0	0
90	23	1	1	1	1	0	0	0
86	21	1	1	0	1	1	0	0
96	22	1	1	0	0	1	0	0
83	22	3	1	0	1	1	0	0
71	20	3	1	0	1	1	0	0
82	22	3	1	0	0	1	0	0
79	18	1	1	1	0	0	0	0
80	20	1	1	0	1	1	0	0
89	22	1	1	0	0	0	0	0
98	20	1	1	0	0	0	0	0
91	20	2	1	0	0	0	0	0
77	20	1	1	0	0	0	0	0
87	20	1	1	0	1	0	0	0
90	20	1	1	0	0	1	0	0
78	21	1	1	0	1	1	0	0
83	20	1	1	0	1	0	0	0
96	22	1	1	0	1	1	0	0
75	22	1	1	0	1	0	0	0
104	22	3	1	0	0	1	0	0
80	22	3	1	0	1	0	0	0
83	20	1	1	0	1	0	0	0
72	20	1	1	0	0	1	0	0
84	23	2	1	0	0	1	0	0
93	20	1	0	0	1	0	0	0
103	25	1	1	0	0	0	0	0
100	21	1	1	0	1	0	0	0
84	21	1	1	0	1	0	0	0
84	21	1	1	0	0	1	0	0
106	21	1	1	0	1	0	0	0

74	20	1	1	0	1	0	0	0
78	20	1	1	0	0	0	0	0
105	20	1	1	0	0	0	0	0
70	20	1	1	0	0	0	0	0
80	20	1	1	0	0	0	0	0
64	19	3	1	0	0	0	0	0
84	21	1	1	0	0	0	0	0
88	20	1	1	0	1	0	0	0
85	20	1	1	0	1	0	0	0
92	20	1	1	0	0	1	0	0
98	20	1	1	0	0	1	0	0
84	21	1	1	0	0	1	0	0
93	20	1	1	0	0	1	0	0
70	21	1	1	0	0	0	0	0
85	19	1	1	0	1	1	0	0
93	21	1	1	0	0	0	0	0
68	22	1	1	1	0	0	0	0
73	20	1	1	0	0	0	0	0
82	21	1	1	0	1	1	0	0
58	28	2	1	0	0	0	0	0
87	20	1	1	0	1	1	0	0
74	21	1	1	0	1	0	1	0
84	20	1	1	0	1	0	0	0
82	21	1	1	0	1	1	0	0
83	21	1	1	1	0	0	0	0
85	17	3	1	0	0	0	0	0
68	20	1	1	0	0	0	0	0
88	21	1	1	0	0	1	0	0
105	23	3	1	0	0	0	0	0
75	20	1	0	0	0	0	0	0
100	21	1	1	0	0	0	0	0
90	20	1	1	0	0	0	0	0
86	20	1	1	0	0	0	0	0
87	20	1	1	0	0	0	0	0
78	19	1	0	0	0	1	0	0
95	24	1	1	0	1	0	0	0
87	20	1	1	0	0	1	0	0
78	20	1	1	0	0	1	0	0
90	22	1	1	0	1	0	0	0
105	24	1	1	0	0	1	0	0
92	21	1	1	1	0	1	0	0
90	18	1	1	0	1	1	0	0
105	24	1	1	0	0	1	0	0
85	17	1	0	0	0	0	0	0
90	20	1	1	0	1	0	0	0
88	20	1	1	0	0	0	0	0
78	20	1	1	0	0	1	0	0
100	20	1	1	0	0	0	0	0
110	22	1	0	0	0	1	0	0
80	22	1	1	0	1	0	0	0
86	20	1	1	0	1	0	0	0
72	18	1	1	1	0	1	0	0
94	22	1	1	0	0	0	0	0
87	23	1	1	0	0	1	0	0

91	21	1	1	1	0	0	0	0
80	20	1	1	0	0	0	0	0
82	20	3	1	0	0	0	0	0
88	22	1	1	1	1	1	0	0
100	22	1	1	0	0	1	0	0
92	18	1	1	0	0	1	0	0
88	20	1	1	0	0	0	0	0
88	20	1	1	1	0	1	0	0
90	24	1	1	0	0	0	0	0
88	20	1	1	1	0	0	0	0
80	21	1	1	0	0	1	0	0
86	22	1	1	0	0	0	0	0
90	21	1	1	0	0	1	0	0
94	23	1	1	0	0	0	0	0
78	22	1	1	1	1	1	0	0
93	20	1	1	0	1	1	0	0
116	27	1	0	0	0	0	0	0
80	21	1	1	0	0	1	0	0
92	21	1	1	0	0	0	0	0
93	20	1	1	0	0	0	0	0
88	20	1	1	0	1	1	0	0
95	20	1	1	0	0	0	0	0

腹泻	呕血	呕咖啡渣牛	黑便	血便	贫血症状	发热	水肿	误食异物
0	0	0	0	0	0	0	0	0
0	0	0	0	0	0	0	0	0
0	0	0	0	0	0	0	0	0
0	0	0	0	0	0	0	0	0
0	0	0	0	0	0	0	0	0
0	0	0	0	0	1	0	0	0
0	0	0	0	0	0	0	0	0
0	0	0	0	0	0	0	0	0
0	0	0	0	0	0	1	0	0
0	0	0	0	0	0	0	0	0
0	0	1	0	0	0	0	0	0
1	0	1	0	0	0	0	0	0
0	1	0	0	0	0	0	0	0
0	0	0	0	0	0	0	0	0
0	0	0	0	0	0	0	0	0
0	0	0	0	0	0	0	0	0
0	0	0	0	1	0	0	0	0
0	0	0	1	0	0	0	0	0
0	0	0	0	0	0	0	0	0
0	0	0	0	0	0	0	0	0
0	0	0	0	0	0	0	0	0
0	0	0	0	0	0	0	0	0
0	0	0	0	0	0	0	0	0
0	0	0	0	0	0	0	0	0
0	0	0	0	0	0	0	0	0
0	0	0	0	0	0	0	0	0
0	0	0	0	0	0	0	0	0
0	0	0	0	0	0	0	0	0
0	0	0	0	0	0	0	0	0
0	0	0	0	0	0	0	0	0
0	0	0	0	0	0	0	0	0
0	0	0	0	0	0	0	0	0
0	0	0	0	0	0	0	0	0
0	0	0	0	0	0	0	0	0
0	0	0	0	0	0	0	0	0
0	0	0	0	0	0	0	0	0
0	0	0	0	0	0	1	0	0
0	0	0	0	0	0	0	0	0
0	0	0	0	0	0	0	0	0
0	0	0	1	0	0	0	0	0
0	0	0	0	0	1	0	0	0
0	0	0	0	0	0	0	0	0
0	0	0	0	0	0	0	0	0
0	0	0	0	0	0	0	0	0
0	0	0	0	0	0	0	0	0
0	0	0	0	0	0	0	0	0
0	1	0	0	0	0	0	0	0
0	0	0	0	0	0	0	0	0
0	1	0	0	0	0	0	0	0
0	0	0	0	0	0	0	0	0
0	1	0	0	0	0	0	0	0
0	0	0	0	0	0	0	0	0
0	0	0	0	0	0	0	0	0

0	0	0	0	1	0	0	0	0
0	0	0	0	0	0	0	0	0
0	0	0	0	1	0	0	0	0
0	0	0	0	0	0	0	1	0
0	0	0	0	0	0	0	0	0
0	0	0	0	0	0	0	0	0
0	0	0	0	0	0	0	0	0
0	0	0	0	0	0	0	0	0
0	0	0	0	0	0	0	0	0
0	0	0	0	0	0	0	0	0
0	0	0	0	1	0	0	0	0
0	0	0	0	0	0	0	0	0
0	0	0	0	0	0	0	0	0
0	0	0	0	0	0	0	0	0
0	0	0	0	0	0	0	0	0
0	1	0	0	1	0	0	0	0
0	0	0	0	1	0	0	0	0
0	0	0	0	1	0	0	0	0
0	0	0	0	0	0	0	0	0
0	0	0	0	1	0	0	0	0
0	0	0	0	0	0	0	0	0
0	0	0	0	0	0	0	0	0
0	0	0	0	0	0	0	0	0
1	0	0	0	0	0	0	0	0
0	0	0	0	0	0	0	0	0
0	0	0	0	0	0	0	0	0
0	0	0	0	0	0	0	0	0
0	0	0	0	1	0	0	0	0
0	0	0	1	1	0	0	0	0
1	0	0	0	0	0	0	0	0
0	0	0	0	1	0	0	0	0
0	0	0	0	0	0	0	0	0
1	0	0	0	0	0	0	0	0
0	0	0	0	0	0	0	0	0
0	0	0	0	0	0	0	0	0
0	0	0	0	0	0	0	0	0
0	0	0	0	0	0	0	0	0
0	0	0	0	1	0	0	0	0
0	0	0	0	0	0	0	0	0
0	0	0	0	0	0	0	0	0
0	0	0	0	0	0	0	0	0
0	0	0	0	0	0	0	0	0
1	0	0	0	0	0	0	0	0
0	0	0	0	0	0	0	0	0
0	0	0	0	0	0	0	0	0
0	0	0	0	0	0	0	0	0
0	0	0	0	0	0	0	0	0
0	0	0	0	0	0	0	0	1
0	0	0	0	0	0	0	0	0
0	0	0	0	0	0	0	0	0
0	1	0	0	0	0	0	0	0
0	0	0	0	0	0	0	0	0
0	1	0	0	0	0	0	0	0
0	1	0	0	0	0	0	0	0

0	0	0	0	1	0	0	0	0
0	0	0	0	1	0	0	0	0
0	0	0	0	0	0	0	0	0
0	0	0	0	0	0	0	0	0
0	0	0	0	0	0	0	0	0
0	0	0	0	0	0	0	0	0
0	0	0	0	0	0	0	0	0
0	0	0	0	0	0	0	0	0
0	0	0	0	0	1	0	0	0
0	1	0	0	0	0	0	0	0
0	0	0	0	0	0	0	0	0
0	0	0	0	0	0	0	0	0
0	0	0	0	0	0	0	0	0
0	0	0	0	0	0	0	0	0
0	0	0	0	0	0	0	0	0
0	0	0	0	0	0	0	0	0
0	0	0	0	0	0	0	0	0
0	0	0	0	0	0	0	0	0
0	0	0	0	0	0	0	0	0
0	0	0	0	0	0	0	0	0
0	0	0	0	0	0	0	0	0
0	1	0	1	0	0	0	0	0
0	0	0	0	1	0	0	0	0
0	0	0	0	0	0	0	0	0
0	0	0	0	1	0	0	0	0
0	0	0	0	1	0	0	0	0
0	0	0	0	0	0	0	0	0
0	0	0	0	0	0	0	0	0
0	0	0	0	0	0	0	0	0
0	0	0	0	0	0	0	0	0
0	0	0	0	0	0	0	0	0
0	0	0	0	0	0	1	0	0
0	0	0	0	0	0	0	0	0
0	0	0	0	0	0	0	0	0
0	0	0	0	0	0	0	0	0
0	0	0	0	0	0	0	0	0
0	0	0	0	0	0	0	0	0
0	0	0	0	0	0	0	0	0
0	0	0	0	0	0	0	0	0
0	0	0	0	0	0	0	0	0
0	0	0	1	0	1	0	0	0
0	0	0	0	0	0	0	0	0
0	0	0	0	0	0	0	0	0
0	1	0	1	0	0	0	0	0
0	0	0	0	0	0	0	0	0
0	0	0	0	1	0	0	0	0
0	0	0	0	0	0	0	0	0
0	0	0	0	0	0	0	0	0
0	0	0	0	0	0	0	0	0
0	0	0	0	0	0	0	0	0
0	0	0	0	0	0	0	0	0
0	0	0	0	0	0	0	0	0
0	0	0	0	1	0	0	0	0
1	1	0	0	0	0	0	0	0

0	0	0	0	0	0	0	0	0
0	0	0	1	0	0	0	0	0
0	0	0	1	0	1	0	0	0
0	0	0	0	0	0	0	0	0
0	0	0	0	1	0	0	0	0
0	0	0	0	0	0	0	0	0
0	0	0	0	0	0	0	0	0
0	0	0	0	0	0	0	0	0
0	0	0	0	0	0	0	1	0
0	0	0	0	0	0	0	0	0
0	0	0	0	0	0	0	0	0
0	0	0	0	0	0	0	0	0
0	0	0	0	1	0	0	0	0
0	1	0	1	0	0	0	0	0
0	0	0	0	0	0	0	0	0
0	0	0	0	0	0	0	0	0
0	0	0	0	0	0	0	0	0
0	0	0	0	0	0	0	0	0
0	0	0	0	0	1	0	0	0
0	0	0	0	0	0	0	0	0
0	0	0	0	0	0	0	0	0
0	0	0	0	0	0	0	0	0
0	0	0	0	0	0	0	0	0
0	0	0	0	0	0	0	0	0
0	0	0	0	1	0	0	0	0
0	0	0	0	0	0	0	0	0
0	0	0	0	0	0	0	0	0
0	0	0	0	0	0	1	0	0
0	0	0	0	0	0	1	0	0
0	0	0	1	0	0	0	0	0
0	0	0	0	0	0	0	0	0
0	0	0	0	0	0	0	0	0
0	0	0	0	1	0	0	0	0
0	0	0	0	0	1	0	0	0
0	0	0	0	0	0	0	0	0
0	0	0	0	0	0	0	0	0
0	0	0	0	1	0	0	0	0
0	0	0	0	0	0	0	0	0
0	0	0	0	0	0	0	0	0
0	0	0	0	0	0	0	0	0
0	0	0	0	0	0	0	0	0
0	1	0	0	0	0	0	0	0
0	0	0	0	0	0	0	0	0
1	0	0	0	0	0	0	0	0
0	0	0	0	0	0	0	0	0
0	0	0	0	0	0	0	0	0
0	0	0	1	0	0	1	0	0
0	1	0	0	0	0	0	0	0
0	0	0	0	1	0	0	0	0
0	0	0	0	0	0	0	0	0
0	1	0	0	0	0	0	0	0
0	0	0	0	0	0	0	0	0
0	0	0	0	1	0	0	0	0
0	0	0	0	0	0	0	0	0

0	0	0	0	0	0	0	0	0
0	0	0	0	1	0	0	0	0
0	0	0	0	0	0	0	0	0
0	0	0	1	1	0	0	0	0
0	0	0	0	0	0	0	0	0
0	0	0	0	0	0	0	0	0
0	0	0	0	0	0	0	0	0
0	0	0	0	0	0	1	0	0
0	0	0	0	1	0	0	0	0
0	0	0	0	0	0	0	0	0
0	0	0	0	0	0	0	0	0
0	0	0	0	0	0	1	0	0
0	0	0	0	0	0	0	0	0
0	0	0	0	0	0	0	0	0
0	0	0	0	1	0	0	0	0
0	0	0	1	1	0	0	0	0
0	0	0	0	0	0	0	0	0
0	0	0	0	1	0	0	0	0
0	0	0	0	0	0	0	0	0
0	0	0	0	1	0	0	0	0
0	0	0	0	0	0	0	0	0
0	0	0	0	0	0	0	0	0
0	0	0	0	0	0	0	0	0
0	0	0	0	0	0	0	0	0
0	0	0	0	0	0	0	0	0
0	0	0	0	0	1	0	0	0
0	0	0	0	0	0	0	0	0
0	0	0	0	0	0	0	0	0
0	0	0	0	0	0	0	0	0
0	0	0	1	0	0	0	0	0
0	0	0	1	0	0	0	0	0
0	0	0	1	0	0	0	0	0
0	0	0	0	0	0	0	0	0
0	0	0	0	1	0	0	0	0
0	0	0	0	1	0	0	0	0
0	0	0	0	0	0	0	0	0
0	0	0	1	0	0	0	0	0
0	0	0	0	1	0	0	0	0
1	0	0	0	0	0	0	1	0
0	0	0	0	1	1	0	0	0
0	0	0	0	0	0	1	0	0
0	1	0	0	0	0	0	0	0
0	0	0	1	0	0	0	0	0
0	0	0	1	0	0	0	0	0
0	0	0	0	0	0	0	0	0
0	0	0	0	0	1	0	0	0
0	1	0	1	0	0	0	0	0
0	0	0	0	0	0	0	0	0
0	0	0	0	0	0	0	0	0
0	0	0	0	1	0	0	0	0
0	0	0	0	0	0	0	0	0
0	0	0	0	0	0	0	0	0
0	0	0	0	0	0	0	0	0
0	0	0	0	1	0	0	0	0

0	0	0	0	1	0	0	0	0
0	0	0	0	0	0	1	0	0
0	0	0	0	1	0	0	0	0
0	0	0	0	1	0	0	0	0
0	0	0	0	0	0	0	0	0
0	0	0	0	0	0	0	0	0
0	0	0	0	0	0	0	0	0
0	0	1	0	0	0	0	0	0
1	0	0	0	0	0	0	0	0
0	0	0	0	0	0	0	0	0
0	0	0	0	0	0	0	0	0
0	0	0	0	0	0	0	0	0
0	1	0	0	1	0	0	0	0
0	0	0	0	0	0	0	0	0
0	0	0	0	0	0	0	0	0
0	0	0	0	0	0	0	0	0
0	0	0	0	1	0	0	0	0
0	0	0	0	1	0	0	0	0
0	0	0	0	1	0	0	0	0
0	0	0	0	1	0	0	0	0
0	0	0	0	0	0	0	0	0
0	0	0	0	0	0	0	0	0
0	0	0	0	0	0	0	0	0
0	0	0	0	0	0	0	0	0
0	0	0	0	0	0	0	0	0
0	0	0	0	0	0	0	0	0
0	0	0	0	1	0	0	0	0
0	0	0	0	0	0	0	0	0
0	0	0	0	0	0	0	0	0
0	0	0	0	0	0	0	0	0
0	0	0	0	0	0	0	0	0
0	0	0	0	0	0	0	0	0
0	0	0	0	0	0	0	0	0
0	1	0	0	0	0	0	0	0
0	0	0	1	0	0	0	0	0
0	0	0	0	0	0	0	0	0
0	0	0	0	0	0	0	0	0
0	0	0	0	0	0	0	0	0
0	0	0	0	0	0	0	0	0
0	0	0	0	0	0	0	0	0
0	0	0	0	0	0	0	0	0
0	0	0	0	0	0	0	0	0
0	0	0	0	0	0	1	0	0
0	0	0	0	0	0	0	0	0
0	0	0	0	0	0	0	0	1
0	0	0	0	0	0	0	0	0
0	0	0	0	0	0	0	0	0
0	0	0	0	0	0	0	0	0
0	0	0	0	0	0	0	0	0
0	0	0	0	0	0	0	0	0
0	0	0	0	0	0	0	0	0
1	0	0	0	0	0	0	0	0
0	0	0	0	0	0	0	0	0
0	0	0	0	0	0	0	0	0

0	0	0	0	0	0	0	0	0
0	1	0	0	0	0	0	0	0
0	0	0	0	0	0	0	0	0
0	0	0	0	0	0	0	0	0
0	0	0	0	0	0	0	0	0
0	0	0	0	0	0	0	0	0
0	0	0	0	0	0	0	0	0
0	0	0	0	0	0	0	0	0
0	0	0	0	0	0	0	0	0
0	0	0	0	0	0	0	0	0
0	0	0	0	0	0	0	0	0
0	0	0	0	0	0	0	0	0
0	0	0	0	0	0	0	0	0
0	0	0	0	0	0	0	0	0
0	0	0	0	0	0	0	0	0
0	0	0	0	0	0	0	0	0
0	0	0	0	0	0	0	0	0
0	0	0	0	0	0	0	0	0
0	0	0	0	0	0	0	0	0
0	0	0	0	0	0	1	0	0
0	0	0	0	0	0	0	0	0
0	0	0	0	0	0	0	0	0
0	0	0	0	1	0	0	0	0
1	0	0	0	0	0	0	0	0
0	0	0	0	0	0	0	0	0
0	0	0	0	0	0	0	0	0
0	0	0	0	0	0	0	0	0
0	1	0	0	0	0	0	0	0
0	0	0	0	0	0	0	0	0
0	0	0	0	0	0	0	0	0
0	0	0	0	1	0	0	0	0
0	0	0	0	1	0	0	0	0
0	0	0	0	0	0	0	0	0
0	0	0	0	0	0	0	0	0
0	0	0	0	0	1	0	0	0
0	0	0	0	0	0	0	0	0
0	0	0	0	0	1	1	0	0
0	0	0	0	0	1	0	0	0
0	0	0	0	0	0	0	0	0
0	0	0	0	0	0	0	0	0
0	0	0	0	0	0	0	0	0
0	0	0	0	0	0	0	0	0
0	0	0	0	0	0	0	0	0
0	0	0	0	0	0	0	0	0
0	0	0	1	0	0	0	0	0
0	0	0	0	0	0	0	0	0
0	0	0	0	0	0	0	0	0
0	0	0	0	0	0	0	0	0
1	0	0	0	0	0	0	0	0
0	0	0	0	0	0	0	0	0
0	0	0	0	0	0	0	0	0
0	0	0	0	0	0	0	0	0
0	1	0	0	0	0	0	0	0
0	0	0	0	0	0	0	0	0

0	0	0	0	0	0	0	0	0
0	0	0	0	0	0	0	0	0
0	0	0	0	0	0	1	0	0
0	0	0	0	0	0	0	0	0
0	1	0	1	0	0	0	0	0
0	0	0	0	0	0	1	0	0
0	0	0	0	0	0	0	0	0
0	0	0	0	0	0	0	0	0
0	0	0	0	0	0	0	0	0
0	0	0	0	0	0	0	0	0
0	0	0	0	0	0	1	0	0
0	0	0	0	0	0	0	0	0
0	0	0	0	0	0	0	0	0
0	0	0	0	0	0	1	0	0
0	0	0	0	0	0	0	0	0
0	0	0	0	0	0	0	0	0
0	0	0	0	0	0	0	0	0
0	0	0	0	0	1	0	0	0
0	0	0	0	0	0	0	0	0
0	0	0	0	0	0	0	0	0
0	0	0	0	0	0	0	0	0
0	0	0	0	0	0	0	0	0
0	0	0	0	0	0	0	0	0
0	0	0	0	0	0	0	0	0
0	0	0	0	0	0	0	0	0
0	0	0	1	0	0	0	0	0
0	0	0	0	0	0	0	0	0
0	0	0	0	0	0	0	0	0
0	0	0	0	0	1	0	0	0
0	0	0	0	0	0	0	0	0
0	0	0	0	0	0	0	0	0
0	0	0	0	0	0	0	0	0
0	0	0	0	0	0	0	0	0
0	0	0	0	0	0	0	0	0
0	0	0	0	0	0	1	0	0
0	0	0	0	0	0	0	0	0
0	0	0	1	0	0	0	0	0
0	0	0	0	1	0	0	0	0
0	1	0	0	0	0	0	0	0
1	0	0	0	1	0	0	0	0
0	0	0	0	0	0	0	0	0
0	0	0	0	0	0	0	0	0
0	0	0	0	0	0	0	0	0
0	0	0	0	0	0	1	0	0
0	0	0	1	0	0	0	0	0
0	0	0	0	1	0	0	0	0
0	0	0	0	0	0	0	0	0
0	0	0	0	0	0	0	0	0
0	0	0	0	0	0	0	0	0
1	0	0	0	0	0	0	0	0
0	0	0	0	0	1	0	0	0
0	0	0	0	0	0	0	0	0
0	0	0	0	0	0	0	0	0
0	0	0	0	0	0	0	0	0

0	0	0	0	0	0	0	0	0
0	0	0	0	0	0	0	0	0
0	0	0	0	0	0	0	0	0
0	0	0	0	0	0	0	0	0
0	0	0	0	0	0	0	0	0
0	0	0	0	0	0	0	0	0
0	0	0	0	0	0	0	0	0
0	0	0	0	0	0	0	0	0
0	0	0	0	0	0	0	0	0
0	0	0	0	0	0	0	0	0
0	0	0	0	0	0	0	0	0
0	0	0	0	0	0	0	0	0
0	0	0	0	0	0	0	0	0
0	0	0	0	0	0	0	0	0
0	0	0	0	0	0	0	0	0
0	0	0	0	0	0	0	0	0
0	0	0	1	0	0	0	0	0
0	0	0	0	0	0	0	0	0
0	0	0	0	0	0	0	0	0
0	0	0	0	0	0	0	0	0
0	0	0	0	0	1	0	0	0
0	1	0	0	0	0	0	0	0
0	0	0	0	0	0	0	0	0
0	0	0	0	0	0	0	0	0
0	0	0	0	0	0	0	0	0
0	0	0	0	0	0	0	0	0
0	0	0	0	0	0	0	0	0
0	0	0	0	0	0	0	0	0
0	0	0	0	0	0	0	0	0
0	0	0	0	0	0	0	0	0
0	0	0	0	0	0	0	0	0
0	0	0	0	0	0	0	0	0
0	0	0	0	0	0	0	0	0
0	0	0	0	0	0	0	0	0
0	0	0	0	0	0	0	0	0
0	0	0	0	0	0	0	0	0
0	1	0	0	0	0	0	0	0
0	0	0	0	0	0	0	0	0
0	0	0	0	0	0	0	0	0
0	0	0	0	0	0	0	0	0
0	0	0	0	0	0	0	0	0
0	0	0	0	0	0	0	0	0
0	0	0	0	0	0	0	0	0
0	0	0	0	0	0	0	0	0
0	0	0	0	0	0	0	0	0
0	0	0	0	0	0	0	0	0
0	0	0	0	0	0	0	0	0
0	0	0	0	0	0	0	0	0
1	0	0	0	0	0	0	0	0
0	0	0	0	0	0	0	0	0
0	0	0	0	0	0	0	0	0
0	0	0	0	0	0	0	0	0
0	0	0	0	0	0	0	0	0
0	0	0	0	0	0	0	0	0

0	0	0	0	0	0	0	0	0
0	0	0	0	0	0	0	0	0
0	0	0	0	0	0	0	0	0
0	0	0	0	0	0	0	0	0
0	0	0	0	0	0	1	0	0
0	0	0	0	0	0	0	0	0
0	0	0	1	0	0	0	0	0
0	0	0	0	1	0	0	0	0
0	0	0	0	1	0	0	0	0
0	0	0	1	0	0	0	0	0
0	0	0	0	0	0	0	0	0
0	0	0	0	0	0	0	0	0
0	0	0	0	0	0	0	0	0
0	0	0	0	0	0	0	0	0
1	0	0	0	0	0	1	0	0
0	0	0	0	0	0	0	0	0
0	0	0	1	0	0	0	0	0
0	0	0	0	0	0	0	0	0
0	0	0	0	0	0	0	0	0
0	0	0	0	1	0	0	0	0
0	0	0	0	0	0	0	0	0
0	0	0	0	1	0	0	0	0
1	0	0	0	0	0	0	0	0
0	0	0	0	0	0	0	0	0
0	0	0	0	0	0	0	0	0
0	0	0	0	0	0	0	0	0
0	1	0	0	0	0	0	0	0
0	0	0	0	1	1	0	0	0
0	0	0	0	0	0	0	0	0
0	0	0	0	0	0	0	0	0
0	0	0	0	0	0	1	0	0
0	0	0	0	0	0	1	0	0
0	0	0	0	0	0	1	0	0
0	0	0	0	0	0	0	0	0
0	0	0	0	0	0	0	0	0
0	0	0	0	0	0	0	0	0
0	0	0	0	0	0	0	0	0
0	0	0	0	0	0	0	0	0
0	0	0	0	0	0	0	0	0
0	0	0	0	0	0	0	0	0
0	0	0	0	0	0	0	0	0
0	0	0	0	0	1	0	0	0
0	0	0	0	0	0	0	0	0
0	0	0	0	0	0	0	0	0
0	0	0	0	0	0	0	0	0
0	0	0	0	0	0	0	0	0
0	0	0	0	0	0	0	0	0
0	0	0	0	0	0	0	0	0
0	0	0	0	0	0	0	0	0
0	0	0	0	0	0	0	0	0
0	0	0	1	0	0	0	0	0
0	0	0	0	0	0	0	0	0
0	0	0	1	0	0	0	0	0
0	0	0	0	0	0	0	0	0

0	0	0	0	1	0	0	0	0
0	0	0	0	0	0	0	0	0
0	0	0	0	0	0	0	0	0
0	0	0	0	0	0	0	0	0
0	0	0	0	0	0	0	0	0
0	1	0	1	0	0	0	0	0
0	0	0	0	0	0	0	0	0
0	0	0	0	0	0	1	0	0
0	0	0	0	0	0	0	0	0
0	0	0	0	0	0	0	0	0
0	0	0	0	0	0	0	0	0
0	0	0	0	0	0	0	0	0
0	0	0	0	1	0	0	0	0
0	0	0	0	0	0	0	0	0
0	0	0	0	0	0	0	0	0
0	0	0	0	0	0	0	0	0
0	0	0	0	0	0	0	0	0
0	0	0	0	0	0	0	0	0
0	0	0	0	0	0	0	0	0
0	0	0	0	0	0	0	0	0
0	0	0	0	1	0	0	0	0
0	0	0	0	0	0	0	0	0
0	0	0	0	0	0	0	0	0
0	0	0	0	1	0	0	0	0
0	0	0	0	0	0	0	0	0
0	0	0	0	0	0	0	0	0
0	0	0	0	0	0	0	0	0
0	0	0	0	1	0	0	0	0
0	0	0	0	0	0	0	0	0
0	0	0	0	0	0	0	0	0
0	0	0	0	0	0	0	0	0
0	0	0	0	0	0	0	0	0
0	0	0	0	0	1	0	0	0
0	1	0	1	0	0	0	0	0
0	0	0	0	0	0	0	0	0
0	0	0	0	0	0	1	0	0
1	0	0	0	1	0	0	0	0
0	0	0	0	0	0	0	0	0
0	0	0	0	0	0	0	0	0
0	0	0	0	0	0	0	0	0
0	0	0	0	0	0	0	0	0
0	0	0	0	0	0	1	0	0
0	0	0	0	0	0	0	0	0
0	1	0	1	0	0	0	0	0
0	0	0	0	1	0	0	0	0
0	0	0	1	0	0	0	0	0
0	0	0	0	0	0	0	0	0
0	0	0	0	0	0	0	0	0
0	0	0	0	0	0	0	0	0
0	0	0	0	0	0	0	0	0
0	0	0	0	0	0	0	0	0
0	1	0	0	0	0	0	0	0
0	0	0	0	0	0	0	0	0
0	0	0	0	0	0	0	0	0

1	0	0	0	1	0	0	0	0
0	0	0	0	0	0	0	0	0
0	0	0	0	0	0	0	0	0
0	0	0	0	0	0	0	0	0
0	0	0	0	0	0	0	0	0
0	0	0	0	0	0	0	0	0
0	0	0	0	0	0	0	0	0
0	0	0	0	0	0	0	0	0
0	0	0	0	0	0	0	0	0
0	0	0	0	0	0	0	0	0
0	0	0	0	0	0	0	0	0
0	0	0	0	0	0	0	0	0
0	0	0	0	0	0	0	0	0
0	0	0	0	0	0	0	0	0
0	0	0	0	0	0	0	0	0
0	0	0	0	1	0	0	0	0
0	0	0	1	0	1	0	0	0
0	0	1	0	0	0	0	0	0
0	0	0	0	0	0	0	0	0
0	0	0	0	0	0	0	0	0
0	0	0	0	1	0	0	0	0
0	0	0	0	0	0	0	0	0
0	0	0	0	0	0	0	0	0
0	0	0	0	0	0	0	0	0
0	0	0	0	0	0	0	0	0
1	0	0	0	0	0	0	0	0
0	0	0	1	0	0	0	0	0
1	0	0	0	0	0	0	1	0
0	0	0	0	0	0	0	0	0
0	0	0	0	0	0	0	0	0
0	0	0	1	0	0	0	0	0
0	0	0	0	0	0	0	0	0
0	0	0	0	0	0	0	0	0
0	0	0	0	0	0	1	0	0
0	0	0	0	0	0	0	0	0
0	0	0	0	0	0	0	0	0
0	0	0	0	0	0	0	0	0
0	0	0	0	0	0	0	0	0
0	0	0	0	0	0	0	0	0
0	0	0	1	0	1	0	0	0
0	0	0	0	0	0	0	0	0
0	0	0	0	0	0	0	0	0
0	0	0	0	0	0	0	0	0
0	0	0	0	0	0	0	0	0
0	0	0	0	0	0	0	0	0
1	0	0	0	0	0	0	0	0
0	0	0	0	0	0	0	0	0
0	0	0	0	0	0	0	0	0
0	0	0	0	0	0	0	0	0
0	0	0	0	0	0	0	0	0
0	0	0	0	0	0	0	0	0
0	0	0	1	0	0	0	0	0
0	0	0	0	0	0	0	0	0
0	0	0	0	0	0	0	0	0

0	0	0	0	0	0	0	0	0
0	1	1	1	0	1	0	0	0
0	0	0	0	0	0	0	0	0
0	0	0	1	0	0	0	0	0
0	0	0	0	0	0	0	0	0
0	0	0	0	1	0	0	0	0
0	0	0	0	0	0	0	0	0
0	0	0	0	0	0	0	0	0
1	0	0	0	0	0	0	0	0
0	0	0	0	0	0	0	0	0
0	0	0	0	0	0	0	0	0
0	0	0	0	1	0	0	0	0
0	0	0	0	0	0	0	0	0
0	0	0	0	0	0	0	0	0
0	0	0	0	1	0	0	0	0
0	0	0	0	0	0	0	0	0
0	0	0	0	0	0	0	0	0
0	0	0	0	0	0	0	0	0
0	0	0	0	0	0	0	0	0
0	0	0	0	0	0	0	0	0
0	0	0	0	0	0	0	0	0
0	0	0	0	0	0	0	0	0
0	0	0	0	0	0	0	0	0
0	0	0	0	0	0	0	0	0
0	0	0	0	0	0	0	0	0
0	0	0	0	0	0	0	0	0
0	0	0	0	0	0	0	0	0
0	0	0	0	0	0	0	0	0
1	0	0	0	0	0	0	0	0
0	0	0	0	0	0	0	0	0
0	0	0	0	0	0	0	0	0
0	1	0	1	0	0	1	0	0
0	0	0	0	0	0	0	0	0
0	0	0	0	0	0	0	0	0
0	0	0	0	0	0	0	0	0
0	0	0	0	0	0	0	0	0
0	0	0	1	1	0	1	0	0
0	0	0	0	0	0	0	0	0
0	0	0	0	0	0	0	0	0
1	0	0	1	0	0	0	0	0
0	0	0	0	0	0	0	0	0
0	0	0	0	0	0	0	0	0
0	0	0	0	0	0	0	0	0
0	0	0	0	0	0	0	0	0
0	0	0	0	1	1	0	0	0
0	0	0	0	0	0	0	0	0
0	0	0	0	0	0	1	0	0
0	0	0	0	0	0	0	0	0
0	0	0	0	0	0	0	0	0
0	0	0	0	0	0	0	0	0
0	0	0	0	0	0	0	0	0
0	0	0	0	0	0	0	0	0
0	0	0	0	0	0	0	0	0
0	0	0	0	0	0	0	0	0

0	0	0	0	1	1	0	0	0
0	0	0	0	0	0	1	0	0
0	0	0	0	0	0	0	0	0
0	0	0	0	0	0	0	0	0
0	0	0	0	0	0	0	0	0
0	0	0	0	0	0	0	0	0
0	0	0	0	0	0	0	1	0
0	0	0	0	1	0	0	0	0
0	0	0	0	0	0	0	0	0
1	0	0	0	0	0	0	0	0
0	0	0	0	0	0	0	0	0
0	0	0	0	0	0	0	0	0
0	0	0	0	0	0	0	0	0
0	1	0	1	0	1	0	0	0
0	0	0	0	0	0	0	0	0
0	0	0	0	0	0	0	0	0
1	0	0	0	0	0	0	0	0
0	0	0	0	0	0	0	0	0
0	0	0	0	0	0	0	0	0
0	0	0	0	0	0	0	0	0
1	0	0	0	1	0	0	0	0
0	1	0	0	0	0	0	0	0
0	0	0	0	0	0	0	0	0
0	0	0	0	0	0	0	0	0
0	0	0	0	0	0	0	0	0
0	0	0	0	0	0	0	0	0
0	0	0	0	0	0	0	0	0
0	0	0	0	0	0	0	0	0
0	0	0	0	0	0	0	0	0
0	0	0	0	0	0	0	0	0
0	0	0	0	0	0	0	0	0
0	0	0	0	0	0	0	0	0
0	0	0	0	0	0	0	0	0
0	0	0	0	0	0	0	0	0
0	0	0	0	0	0	0	0	0
0	0	0	0	0	0	0	0	0
0	0	0	0	0	0	0	0	0
0	0	0	0	0	0	0	0	0
0	0	0	0	0	0	0	0	0
0	0	0	0	0	0	0	0	0
0	0	0	0	0	0	0	0	0
1	0	0	1	0	0	0	0	0
0	0	0	0	0	0	0	0	0
0	0	0	0	0	0	0	0	0
0	0	0	0	0	0	1	1	0
0	0	0	0	0	0	0	0	0
0	0	0	0	0	0	0	0	0
0	0	0	0	0	0	1	0	0
0	0	0	0	0	0	1	0	0
1	0	0	0	0	0	0	0	0
0	0	0	0	0	0	0	0	0
0	0	0	0	0	0	0	0	0
0	0	0	1	0	0	0	0	0
0	0	0	0	0	0	0	0	0

0	0	0	0	0	0	0	0	0
0	0	0	0	0	0	0	0	0
0	0	0	0	0	0	1	0	0
1	0	0	0	0	0	1	0	0
0	0	0	0	0	0	0	0	0
0	0	0	0	0	0	1	0	0
0	0	0	0	0	0	0	0	0
0	0	0	0	0	0	1	0	0
0	0	0	0	0	1	1	0	0
0	0	0	0	0	0	0	0	0
0	0	0	0	0	0	0	0	0
0	0	0	1	0	0	0	0	0
0	0	0	0	0	0	0	0	0
0	0	0	0	1	0	0	0	0
0	0	0	0	1	1	0	0	0
0	0	0	0	0	0	0	0	0
0	0	0	0	0	0	0	0	0
0	0	0	0	0	0	0	0	0
0	0	0	0	1	0	0	0	0
0	0	0	0	0	0	0	0	0
0	0	0	0	0	0	0	0	0
0	0	0	1	0	0	0	0	0
0	0	0	1	0	0	0	0	0
0	0	0	0	0	0	0	0	0
0	0	1	1	0	1	1	0	0
0	0	0	0	0	0	0	0	0
0	0	0	0	0	0	0	0	0
0	0	0	0	0	0	0	0	0
1	0	0	0	0	0	0	0	0
0	0	0	0	0	0	0	0	0
0	0	0	0	0	0	0	0	0
0	0	0	0	0	0	0	0	0
0	0	0	0	0	0	0	0	0
0	0	0	0	1	0	0	0	0
0	0	0	0	0	0	0	0	0
0	0	0	0	0	0	0	0	0
0	0	0	0	0	0	0	0	0
0	0	0	0	0	0	0	0	0
0	0	0	0	0	0	0	0	0
0	0	0	0	0	0	0	0	0
0	0	1	0	1	0	0	0	0
0	0	0	0	0	0	0	0	0
0	0	0	0	0	0	0	0	0
0	0	0	0	0	0	0	0	0
0	0	0	0	0	0	0	0	0
0	0	0	0	0	0	0	0	0
0	0	0	0	0	0	0	0	0
0	0	0	1	0	0	0	0	0
0	0	0	0	0	0	0	0	0
1	0	0	0	0	0	0	0	0
0	0	0	0	0	0	0	0	0
0	0	0	0	0	0	0	0	0
0	0	0	0	0	0	0	0	0
0	0	0	0	0	0	0	0	0

0	1	0	1	0	0	0	0	0
0	0	0	0	0	0	0	0	0
0	0	0	0	0	0	0	0	0
0	0	0	0	0	0	0	0	0
0	0	0	0	0	0	1	0	0
0	0	1	0	0	0	0	0	0
0	0	0	0	0	0	0	0	0
1	0	0	0	0	0	1	0	0
0	0	0	0	0	0	0	0	0
0	0	0	0	0	0	0	0	0
0	0	0	0	0	0	0	0	0
0	0	0	0	0	0	0	0	0
0	0	0	0	0	0	0	0	0
0	0	0	0	0	0	0	0	0
0	0	0	0	0	0	0	0	0
0	0	0	0	0	0	0	0	0
0	0	0	0	0	0	0	0	0
0	0	0	1	0	1	0	0	0
1	0	0	0	0	0	0	0	0
0	0	0	0	0	0	0	0	0
0	0	0	0	0	0	0	0	0
0	0	0	0	0	0	0	0	0
0	0	0	0	0	0	0	0	0
0	0	0	0	0	0	0	0	0
0	0	0	0	0	0	0	0	0
0	0	0	0	0	0	0	0	0
0	0	0	0	1	1	1	0	0
0	0	0	0	0	0	0	0	0
0	1	0	0	0	1	0	0	0
0	0	0	0	0	0	0	0	0
0	0	0	0	0	0	0	0	0
1	0	0	0	0	0	0	0	0
1	0	0	0	1	0	1	0	0
0	0	0	0	0	0	0	0	0
0	0	0	0	0	0	0	0	0
0	0	0	0	0	0	1	0	0
0	0	0	0	0	0	0	0	0
0	0	0	0	0	0	0	0	0
1	0	0	0	0	0	0	0	0
0	0	0	0	0	0	0	0	0
0	0	0	0	0	0	0	0	0
1	0	0	0	0	1	0	0	0
0	0	0	0	0	0	0	0	0
0	0	0	0	0	0	0	0	0
0	0	0	0	0	0	0	0	0
0	0	0	0	0	0	0	0	0
0	0	0	0	0	0	0	0	0
0	0	0	0	0	0	0	0	0
0	0	0	0	0	0	0	0	0
0	0	0	0	1	0	0	0	0
0	0	0	0	0	0	0	0	0
0	0	0	0	0	0	0	0	0
0	0	0	0	0	0	0	0	0
0	0	0	0	0	0	0	0	0

0	0	0	0	0	0	0	0	0
1	0	0	0	0	0	0	0	0
0	0	0	0	1	0	0	0	0
0	0	0	1	0	0	0	0	0
0	0	0	0	1	0	0	0	0
0	0	0	0	1	0	0	0	0
0	0	0	0	0	0	0	0	0
0	0	0	0	0	0	0	0	0
0	0	0	0	1	0	0	0	0
0	0	0	0	0	0	0	0	0
0	0	0	0	0	0	0	0	0
0	0	0	0	0	0	0	0	0
0	0	0	0	0	0	0	0	0
0	0	0	0	0	0	0	0	0
0	0	0	0	0	0	0	0	0
0	0	0	0	0	1	1	0	0
0	0	0	0	0	0	0	0	0
0	0	0	0	0	0	0	0	0
1	0	0	0	0	0	0	0	0
0	0	0	0	0	0	1	0	0
0	0	0	0	0	0	0	0	0
0	0	0	0	0	0	0	0	0
0	0	0	0	0	0	0	0	0
0	0	0	0	0	0	0	0	0
0	0	0	0	0	0	0	0	0
0	0	0	0	0	0	0	0	0
0	0	0	1	0	1	0	0	0
0	0	0	0	0	0	0	0	0
0	0	0	0	0	0	0	0	0
0	0	0	0	0	0	0	0	0
0	0	0	0	0	0	0	0	0
0	0	0	0	0	0	0	0	0
0	0	0	0	0	0	0	0	0
0	0	0	0	0	0	0	0	0
0	0	0	1	0	0	0	0	0
0	0	0	0	0	0	0	0	0
0	0	0	0	0	0	0	0	0
0	0	0	0	0	0	0	0	0
0	0	0	0	0	0	0	0	0
0	0	0	0	0	0	0	0	0
0	0	0	0	0	0	0	0	0
0	0	0	0	0	0	0	0	0
0	0	0	0	0	0	0	0	0
0	0	0	1	0	0	0	0	0
0	0	0	0	0	0	0	0	0
0	0	0	0	0	0	0	0	0
0	0	0	0	0	0	0	0	0
0	0	0	0	0	0	0	0	0
0	0	0	0	0	0	0	0	0
0	0	0	0	0	0	1	0	0
0	1	0	0	0	0	0	0	0
0	0	0	0	0	0	0	0	0
0	0	0	1	0	0	0	0	0
0	0	0	0	0	0	0	0	0

0	0	0	0	0	0	0	0	0
0	0	0	0	0	0	0	0	0
0	0	0	0	0	0	0	0	0
1	0	0	0	0	0	0	0	0
1	0	0	0	0	0	0	0	0
0	0	0	0	0	0	0	0	0
0	0	0	0	0	0	0	0	0
0	0	0	0	0	0	0	0	0
0	0	0	0	0	0	0	0	0
0	0	0	1	1	0	0	0	0
0	0	0	0	0	0	0	0	0
0	0	0	0	0	0	0	0	0
0	0	0	0	0	0	0	0	0
0	0	0	0	0	0	0	0	0
0	0	0	0	0	0	0	0	0
0	0	0	0	0	0	0	0	0
0	0	0	0	0	0	0	0	0
0	0	0	0	0	0	0	0	0
0	0	0	0	0	0	0	0	0
0	0	0	0	0	0	0	0	0
0	0	0	0	0	0	0	0	0
0	0	0	0	0	0	0	0	0
1	0	0	0	0	0	0	0	0
0	0	0	0	0	0	0	0	0
0	0	0	0	0	0	0	0	0
0	0	0	0	1	0	0	0	0
0	0	0	0	1	1	0	0	0
0	0	0	0	0	1	0	0	0
0	0	0	0	0	0	0	0	0
0	0	0	0	0	0	0	0	0
0	0	0	1	0	0	0	0	0
0	0	0	0	0	0	0	0	0
0	0	0	0	0	0	0	0	0
0	0	0	0	0	0	0	0	0
0	0	0	0	0	0	0	0	0
1	0	0	0	0	0	0	0	0
0	0	0	0	0	0	0	0	0
0	0	0	0	1	0	0	0	0
0	0	0	0	0	0	0	0	0
1	0	0	0	0	0	0	0	0
0	0	0	0	0	0	1	0	0
0	0	0	0	0	0	0	0	0
0	0	0	0	0	0	0	0	0
0	0	0	0	0	0	0	0	0
0	0	0	0	0	0	0	0	0
1	0	0	0	0	0	0	0	0
0	0	0	0	0	0	0	0	0
0	0	0	0	0	0	0	0	0
0	0	0	0	0	0	0	0	0
0	0	0	0	0	0	0	0	0
0	0	0	0	0	0	0	0	0
0	0	0	0	0	0	0	0	0
0	0	0	0	0	0	0	0	0
0	0	0	0	0	0	0	0	0

0	0	0	0	0	0	0	0	0
0	0	0	0	0	0	0	0	0
0	0	0	0	0	0	0	0	0
0	0	0	1	1	1	0	0	0
0	0	0	0	0	0	0	0	0
0	0	0	0	0	0	0	0	0
0	0	0	0	0	0	0	0	0
0	0	0	0	0	0	0	0	0
0	0	0	0	0	0	0	0	0
0	0	0	0	1	1	0	0	0
0	0	0	0	0	0	0	0	0
0	0	0	0	0	0	1	0	0
0	0	0	0	0	0	0	0	0
1	0	0	0	0	0	0	0	0
0	0	0	0	0	0	0	0	0
0	0	0	0	0	0	0	0	0
0	0	0	0	0	0	0	0	0
0	1	0	0	0	0	0	0	0
0	0	0	0	0	0	0	0	0
0	0	0	0	0	0	0	0	0
1	0	0	0	0	0	0	0	0
0	0	0	0	0	0	0	0	0
0	0	0	0	0	0	0	0	0
0	0	0	0	0	0	0	0	0
0	0	0	0	0	0	0	0	0
0	0	0	0	0	0	0	0	0
0	0	0	0	0	0	0	0	0
0	0	0	0	0	0	0	0	0
0	1	0	0	0	0	0	0	0
0	0	0	0	0	0	0	0	0
0	0	0	0	0	0	0	0	0
0	0	0	0	0	0	0	0	0
0	0	0	0	0	0	0	0	0
0	0	0	0	0	0	0	0	0
0	0	0	0	0	0	0	0	0
0	0	0	0	0	0	0	0	0
0	0	0	0	0	0	0	0	0
0	0	0	0	0	0	0	0	0
0	1	0	0	0	0	0	0	0
1	0	0	0	0	0	0	0	0
0	0	0	0	1	0	0	0	0
0	0	0	0	0	0	0	0	0
0	0	0	0	0	0	0	0	0
1	0	0	0	0	0	0	0	0
0	0	0	0	0	0	0	0	0
0	0	0	0	0	0	0	0	0
0	0	0	0	0	0	0	0	0
0	0	0	0	0	0	0	0	0
0	1	0	0	0	1	0	0	0
0	0	0	0	0	0	0	0	0
1	0	0	0	0	0	0	0	0
0	0	0	0	0	0	0	0	0
0	0	0	0	0	0	0	0	0
0	0	0	0	1	0	0	0	0

1	0	0	0	0	0	0	1	0
0	0	0	0	0	0	0	0	0
0	0	0	0	0	0	0	0	0
0	1	0	0	0	0	0	0	0
0	0	0	0	0	0	0	0	0
0	0	0	0	1	0	0	0	0
0	0	0	0	0	0	0	0	0
0	0	0	0	0	0	0	0	0
0	0	0	0	0	0	0	0	0
0	0	0	0	0	0	0	0	0
0	0	0	0	0	0	0	0	0
0	0	0	0	0	0	0	0	0
0	0	0	0	0	0	0	0	0
0	0	0	0	1	0	0	0	0
0	0	0	0	0	0	0	0	0
0	0	0	0	0	0	0	0	0
0	0	0	0	0	0	0	0	0
0	0	0	0	0	0	0	0	0
0	0	0	0	0	0	0	0	0
0	0	0	0	0	0	0	0	0
0	0	0	0	0	0	0	0	0
0	0	0	0	0	0	0	0	0
0	0	0	0	0	0	0	0	0
0	0	0	0	0	0	0	0	0
0	0	0	0	0	0	0	0	0
0	0	0	0	0	0	0	0	0
0	0	0	0	0	0	0	0	0
0	0	0	0	0	0	0	0	0
0	0	0	0	0	0	0	0	0
0	0	0	0	0	0	0	0	0
0	0	0	0	0	0	0	0	0
0	0	0	0	0	0	0	0	0
0	0	0	0	1	0	0	0	0
0	0	0	0	0	0	0	0	0
0	0	0	0	0	0	0	0	0
0	0	0	0	0	0	0	0	0
0	0	0	0	0	1	0	0	0
0	0	0	0	0	0	0	0	0
0	0	0	0	0	0	0	0	0
0	0	0	0	0	0	1	0	0
0	0	0	0	0	0	0	0	0
0	0	0	0	0	0	0	0	0
0	0	0	0	0	0	0	0	0
0	0	0	0	0	0	0	0	0
0	0	0	0	0	0	0	0	0
0	0	0	0	1	1	0	0	0
0	0	0	0	0	0	1	0	0
1	0	0	0	0	0	0	0	0
0	0	0	0	0	0	0	0	0
0	1	0	0	0	0	0	0	0
0	0	0	0	0	0	0	0	0
0	0	0	0	0	0	0	0	0
0	0	0	0	0	0	0	0	0
0	0	0	0	0	0	0	0	0

0	0	0	0	0	0	1	0	0
0	0	0	0	0	0	0	0	0
0	0	0	0	0	0	0	0	0
0	0	0	0	0	0	1	0	0
0	0	0	0	0	0	0	0	0
1	0	0	0	0	0	0	0	0
0	0	0	0	0	0	0	0	0
0	0	0	0	0	0	0	0	0
0	0	0	0	0	0	0	0	0
0	0	0	0	0	0	0	0	0
0	0	0	0	0	0	0	0	0
0	0	0	0	0	0	0	0	0
0	0	0	0	0	0	0	0	0
0	0	0	0	0	0	0	0	0
0	0	0	0	0	0	0	0	0
0	0	0	0	0	0	0	0	0
0	0	0	0	0	0	0	0	0
0	0	0	0	0	0	0	0	0
0	0	0	0	0	0	0	0	0
0	0	0	0	0	0	0	0	0
0	0	0	0	0	0	0	0	0
0	0	0	0	0	0	0	0	0
0	0	0	0	0	0	0	0	0
0	0	0	0	0	0	0	0	0
0	0	0	0	0	0	0	0	0
0	0	0	0	0	0	0	0	0
0	0	0	0	0	0	0	0	0
0	0	0	0	0	0	0	0	0
0	0	0	0	0	0	0	0	0
0	0	0	0	1	0	0	0	0
0	0	0	0	1	0	1	0	0
0	0	0	0	1	0	0	0	0
0	0	0	0	0	0	0	0	0
0	0	0	0	0	0	1	0	0
0	0	0	0	0	0	0	0	0
0	0	0	0	0	0	0	0	0
0	0	0	0	0	0	0	0	0
1	0	0	0	0	0	0	0	0
0	0	0	0	1	0	0	0	0
0	0	0	0	0	0	1	0	0
0	0	0	0	0	0	0	0	0
0	0	0	0	0	0	0	0	0
0	0	0	0	0	0	0	0	0
0	0	0	0	0	0	0	0	0
0	0	0	0	0	0	0	0	0
0	0	0	0	0	0	0	0	0
0	0	0	0	0	0	0	0	0
0	0	0	0	0	0	0	0	0
0	0	0	0	1	0	0	0	0
0	0	0	0	0	0	0	0	0
0	0	0	0	0	0	0	0	0
0	0	0	0	0	0	0	0	0
0	0	0	0	0	0	0	0	0
0	0	0	0	0	0	0	0	0

1	0	0	0	0	0	0	0	0
0	0	0	0	0	0	0	0	0
0	0	0	0	0	0	0	0	0
0	0	0	0	0	0	1	0	0
0	0	0	0	0	0	0	0	0
0	0	0	0	0	0	0	0	0
0	0	0	0	0	0	0	0	0
0	0	0	0	0	0	1	0	0
0	0	0	0	0	0	0	0	0
0	0	0	0	0	0	0	0	0
0	0	0	0	0	0	0	0	0
0	0	0	0	0	0	0	0	0
1	0	0	0	0	0	0	0	0
1	0	0	0	0	0	0	0	0
0	0	0	0	0	0	0	0	0
0	0	0	0	0	0	0	0	0
1	0	0	0	0	0	0	0	0
0	0	0	0	0	0	0	0	0
0	0	0	0	0	0	0	0	0
0	1	0	0	0	0	0	0	0
0	0	0	0	0	0	0	0	0
0	0	0	0	1	0	0	0	0
0	0	0	0	0	0	0	0	0
0	0	0	0	1	0	0	0	0
1	0	0	0	0	0	0	0	0
0	0	0	0	0	1	0	0	0
0	0	0	0	0	0	0	0	0
0	0	0	0	0	0	0	0	0
0	0	0	0	0	0	0	0	0
0	0	0	0	0	0	0	0	0
0	0	0	0	0	0	0	0	0
0	0	0	0	0	0	0	0	0
0	0	0	0	0	0	0	0	0
0	0	0	0	0	0	0	0	0
0	0	0	0	1	0	0	0	0
0	0	0	0	0	0	0	0	0
0	0	0	0	0	0	0	0	0
0	0	0	0	0	0	0	0	0
0	0	0	0	0	0	0	0	0
0	0	0	0	0	0	0	0	0
0	0	0	0	0	0	0	0	0
0	0	0	0	0	0	0	0	0
0	0	0	0	0	0	0	0	0
0	0	0	0	0	0	0	0	0
0	0	0	1	1	0	0	0	0
0	0	0	0	0	0	0	0	0
0	0	0	0	0	1	1	0	0
0	0	0	0	0	0	0	0	0
0	0	0	0	0	0	0	0	0
0	0	0	0	0	0	0	0	0
1	0	0	0	0	0	0	0	0
0	0	0	0	0	0	0	0	0
0	0	0	0	0	0	0	0	0
0	0	0	0	0	0	0	0	0

0	0	0	0	0	0	0	0	0
0	0	0	0	0	0	0	0	0
0	0	0	0	0	0	0	0	0
0	0	0	0	0	0	0	0	0
0	0	0	0	0	0	0	0	0
0	0	0	0	0	0	0	0	0
0	0	0	0	0	0	0	0	0
0	0	0	0	0	0	0	0	0
0	0	0	1	1	0	1	0	0
0	0	0	0	0	0	0	0	0
0	0	0	0	0	0	0	0	0
0	0	0	0	0	0	0	0	0
0	0	0	0	0	0	0	0	0
0	0	0	0	0	0	0	0	0
0	0	0	0	0	0	0	0	0
0	0	0	0	0	0	0	0	0
0	0	0	0	1	0	0	0	0
0	0	0	0	0	0	0	0	0
0	0	0	0	0	0	0	0	0
0	0	0	0	0	0	0	0	0
1	0	0	0	0	0	0	0	0
1	0	0	0	0	0	0	0	0
0	0	0	0	0	0	0	0	0
1	0	0	0	0	0	1	1	0
0	0	0	1	0	1	0	0	0
0	0	0	0	1	0	0	0	0
0	0	0	0	0	0	0	0	0
0	0	0	0	0	0	0	0	0
0	0	0	0	0	0	0	0	0
0	0	0	0	0	0	0	0	0
0	0	0	0	0	0	0	0	0
1	0	0	0	0	0	0	0	0
0	0	0	0	0	0	0	0	0
0	0	0	0	0	0	0	0	0
0	0	0	0	0	0	0	0	0
1	0	0	0	0	0	0	0	0
0	0	0	0	0	0	0	0	1
0	0	0	0	0	0	0	0	0
0	0	0	0	0	0	0	0	0
0	0	0	0	0	0	0	0	0
1	0	0	0	0	0	0	0	0
0	0	0	0	0	0	0	0	0
0	0	0	0	0	0	0	0	0
0	0	0	0	0	0	0	0	0
0	0	0	0	1	0	0	0	0
0	0	0	0	0	0	0	0	0
0	0	0	1	0	0	0	0	0
0	0	0	0	0	0	0	0	0
0	0	0	0	0	0	0	0	0
0	0	0	0	0	0	0	0	0
0	0	0	0	0	0	0	0	0
1	0	0	1	1	0	0	0	0
0	0	0	0	0	0	0	0	0
0	0	1	0	0	1	0	0	0

0	0	0	0	0	0	0	0	0
0	0	0	0	0	0	0	0	0
0	0	0	0	0	0	0	0	0
0	0	0	0	0	0	0	0	0
0	0	0	0	0	0	0	0	0
0	0	0	0	0	0	0	0	0
0	0	0	0	0	0	0	0	0
0	0	0	0	0	0	0	0	0
0	0	0	0	0	0	1	0	0
0	0	0	0	1	0	0	0	0
0	0	0	0	0	0	0	0	0
0	0	0	0	0	0	0	0	0
1	0	0	1	0	0	1	0	0
0	0	0	0	0	0	0	0	0
0	0	0	0	0	0	0	0	0
0	0	0	0	0	0	0	0	0
0	0	0	0	0	0	0	0	0
0	0	0	0	0	0	0	0	0
0	0	0	0	1	1	0	0	0
0	0	0	0	0	0	0	0	0
0	0	0	0	0	0	0	0	0
0	0	0	0	0	0	1	0	0
0	0	0	0	0	0	0	0	0
0	0	0	0	0	0	0	0	0
0	0	0	0	0	0	0	0	0
1	0	0	0	0	0	0	0	0
0	0	0	1	0	0	0	0	0
0	1	0	0	0	0	0	0	0
0	0	0	0	0	0	0	0	0
0	0	0	0	0	0	1	0	0
1	0	0	0	0	0	0	0	0
0	0	0	0	0	0	0	0	0
0	1	0	0	0	0	0	0	0
0	0	0	0	0	0	0	0	0
0	0	0	0	0	0	0	0	0
0	0	0	0	0	0	0	0	0
0	0	0	0	0	0	0	0	0
0	0	0	0	0	0	0	0	0
0	0	0	0	0	0	0	0	0
0	0	0	0	0	0	1	0	0
0	0	0	0	0	0	1	0	0
0	0	0	0	0	0	0	0	0
0	0	0	0	0	0	0	0	0
0	1	0	0	0	1	0	0	0
0	0	0	1	0	0	0	0	0
0	0	0	0	0	0	0	0	0
0	0	0	1	0	0	0	0	0
0	0	0	0	0	0	0	0	0
0	0	0	0	0	0	0	0	0
0	0	0	0	0	0	0	0	0
0	0	0	1	0	0	1	0	0
0	0	0	0	0	0	0	0	0
0	0	0	0	0	0	0	0	0
0	1	0	1	0	1	0	0	0

0	0	0	0	1	0	0	0	0
0	0	0	1	0	0	1	0	0
0	0	0	0	0	0	1	0	0
0	0	0	0	0	0	0	0	0
0	0	0	0	0	0	0	0	0
0	0	0	0	0	1	1	0	0
0	0	0	0	0	0	0	0	0
0	0	0	0	0	0	0	0	0
0	0	0	0	0	0	0	0	0
0	0	0	0	0	0	0	0	0
0	0	0	0	0	0	0	0	0
0	0	0	0	0	0	0	0	0
0	0	0	0	0	0	0	0	0
0	0	0	0	0	0	0	0	0
0	0	0	0	0	0	0	0	0
0	0	0	0	0	1	0	0	0
0	0	0	0	0	0	0	0	0
1	0	0	0	0	0	0	0	0
0	0	0	0	0	0	0	0	0
0	0	0	0	0	0	0	0	0
0	0	0	0	0	0	0	0	0
0	0	0	0	0	0	0	0	0
1	0	0	0	0	0	0	0	0
0	0	0	0	0	0	0	0	0
0	0	0	0	0	0	0	0	0
0	0	0	0	0	0	0	0	0
1	0	0	0	0	0	1	0	0
0	0	0	0	0	0	0	0	0
0	0	0	0	0	0	1	0	0
1	0	0	0	0	0	0	0	0
0	0	0	0	0	0	0	0	0
0	0	0	0	0	0	0	0	0
1	0	0	0	0	0	1	0	0
0	0	0	0	0	0	0	0	0
0	0	0	0	0	0	0	0	0
0	0	0	0	0	0	0	0	0
0	0	0	0	0	0	0	0	0
0	0	0	0	0	0	0	0	0
0	0	0	0	0	0	1	0	0
1	0	0	0	0	0	1	0	0
0	0	0	0	0	0	0	0	0
0	0	0	0	0	0	1	0	0
0	0	0	0	0	0	0	0	0
0	0	0	0	0	0	0	0	0
0	0	0	0	0	0	0	0	0
0	0	0	0	0	0	0	0	0
0	0	0	0	0	0	0	0	0
0	0	0	0	0	0	1	0	0
0	0	0	0	0	0	0	0	0
0	0	0	0	1	0	0	0	0
0	0	0	0	0	0	0	0	0
0	0	0	0	0	0	1	0	0
0	0	0	0	0	0	1	0	0
0	0	0	0	0	0	0	0	0

0	0	0	0	0	0	1	0	0
0	0	0	0	0	0	0	0	0
1	0	0	0	0	0	1	0	0
0	0	0	0	0	0	0	0	0
0	0	0	0	0	0	0	0	0
0	0	0	0	0	0	0	0	0
0	0	0	0	0	0	0	0	0
0	0	0	0	0	0	0	0	0
0	0	0	0	0	0	0	0	0
0	0	0	0	0	0	0	0	0
1	0	0	0	0	1	1	0	0
0	0	0	0	0	0	0	0	0
0	0	0	0	0	0	0	0	0
0	0	0	0	0	0	0	0	0
1	0	0	0	0	0	0	0	0
0	0	0	0	0	0	0	0	0
0	0	0	0	0	0	0	0	0
0	0	0	0	0	0	0	0	0
0	0	0	0	0	0	0	0	0
0	0	0	0	0	0	0	0	0
0	0	0	0	0	1	0	0	0
0	0	0	0	0	0	0	0	0
0	0	0	0	0	0	0	0	0
0	0	0	0	0	0	0	0	0
0	0	0	0	0	0	0	0	0
0	0	0	0	1	0	0	0	0
0	0	0	0	0	0	0	0	0
1	0	0	0	0	0	0	0	0
0	0	0	0	1	0	0	0	0
0	0	0	0	0	0	0	0	0
0	0	0	0	0	0	0	0	0
0	0	0	0	0	0	0	0	0
0	0	0	0	0	0	0	0	0
0	0	0	0	0	0	0	0	0
0	0	0	0	0	0	0	0	0
0	0	0	0	0	0	1	0	0
0	0	0	0	0	0	0	0	0
0	0	0	0	0	0	0	0	0
0	0	0	0	0	0	0	0	0
0	0	0	0	0	0	1	0	0
0	0	0	0	0	0	0	0	0
0	0	0	0	0	0	0	0	0
0	0	0	0	0	0	1	0	0
0	1	0	0	0	1	0	0	0
0	0	0	0	0	0	1	0	0
0	0	0	0	0	0	0	0	0
0	0	0	0	0	0	0	0	0
0	0	0	0	0	0	1	0	0
1	0	0	0	0	0	0	0	0
0	0	0	0	0	0	0	0	0
0	0	0	0	0	0	0	0	0
0	0	0	0	0	1	0	0	0
0	0	0	0	0	0	0	0	0
0	0	0	0	0	0	0	0	0

0	0	0	0	0	0	1	0	0
0	0	0	0	0	0	0	0	0
1	0	0	0	0	0	0	0	0
1	0	0	0	0	0	0	0	0
0	1	0	0	0	0	0	0	0
0	0	0	0	0	0	1	0	0
0	0	0	0	0	0	0	0	0
0	0	0	0	0	0	0	0	0
0	0	0	0	0	0	0	0	0
0	0	0	0	0	0	0	0	0
0	0	0	0	0	0	0	0	0
0	0	0	0	0	0	0	0	0
0	0	0	0	0	0	0	0	0
0	0	0	0	0	0	0	0	0
0	1	0	1	0	0	0	0	0
0	0	0	0	0	0	0	0	0
0	0	0	0	1	0	0	0	0
0	0	0	0	0	0	0	0	0
0	0	0	0	0	0	0	0	0
0	0	0	0	0	0	0	0	0
0	0	0	0	0	0	0	0	0
0	0	0	0	1	0	0	0	0

呼吸道症	皮肤表现	口腔溃疡	既往史	消化系统	呼吸系统	血液系统	其他既往	家族史
0	0	0	0	0	0	0	0	0
0	0	1	1	1	0	0	0	0
0	0	0	0	0	0	0	0	0
0	0	1	1	1	0	0	0	0
0	0	0	0	0	0	0	0	0
0	0	0	1	0	0	0	1	0
0	0	0	0	0	0	0	0	0
0	0	0	0	0	0	0	0	0
0	0	0	1	1	0	0	0	0
0	0	0	0	0	0	0	0	0
0	0	0	1	1	0	0	0	0
0	0	0	0	0	0	0	0	0
0	0	0	0	0	0	0	0	0
0	0	0	0	0	0	0	0	0
0	0	1	1	1	0	0	0	0
0	0	1	1	1	0	0	0	0
0	0	1	1	1	0	0	0	0
0	0	0	1	1	0	0	1	0
0	1	0	0	0	0	0	0	0
0	1	0	1	0	0	0	1	0
0	0	0	1	0	0	0	1	0
0	1	0	0	0	0	0	0	0
0	0	0	0	0	0	0	0	0
0	0	0	0	0	0	0	0	0
0	0	0	0	0	0	0	0	0
0	0	0	0	0	0	0	0	0
0	0	0	0	0	0	0	0	0
0	0	0	0	0	0	0	0	0
0	0	0	0	0	0	0	0	0
0	0	0	0	0	0	0	0	0
0	0	0	0	0	0	0	0	0
0	0	0	0	0	0	0	0	0
0	0	0	1	1	0	0	1	0
0	0	0	0	0	0	0	0	0
0	1	0	0	0	0	0	0	0
0	0	0	1	0	0	0	1	0
0	0	0	1	0	0	1	0	0
0	0	0	1	1	0	0	1	0
0	0	0	0	0	0	0	0	0
0	1	0	1	0	0	0	1	0
0	0	0	1	0	0	0	1	0
0	0	0	0	0	0	0	0	0
0	0	0	0	0	0	0	0	0
0	0	0	0	0	0	0	0	0
0	0	0	0	0	0	0	0	0
0	0	0	0	0	0	0	0	0
0	1	0	1	0	0	0	1	1
0	0	0	0	0	0	0	0	0
0	0	0	0	0	0	0	0	0
0	0	0	0	0	0	0	0	0
0	0	0	1	1	0	0	0	0
0	0	0	0	0	0	0	0	0
0	0	0	1	0	0	0	1	0

0	0	0	0	0	0	0	0	0
0	0	0	0	0	0	0	0	0
0	0	0	0	0	0	0	0	0
0	0	0	1	1	0	0	0	0
0	0	0	0	0	0	0	0	0
0	0	0	0	0	0	0	0	0
0	0	0	0	0	0	0	0	0
0	0	0	1	1	0	0	0	0
0	0	0	0	0	0	0	0	0
0	1	0	0	0	0	0	0	0
0	0	0	1	0	0	0	1	0
0	0	0	0	0	0	0	0	0
0	0	0	0	0	0	0	0	0
0	0	0	0	0	0	0	0	0
0	0	0	1	1	0	0	0	0
0	0	0	1	1	0	0	0	0
0	0	0	0	0	0	0	0	0
0	0	0	1	0	0	0	1	0
0	0	1	0	0	0	0	0	0
0	0	0	0	0	0	0	0	0
0	0	0	1	0	0	0	1	0
0	0	0	0	0	0	0	0	0
0	0	0	0	0	0	0	0	0
0	0	0	1	1	0	0	0	1
0	0	0	0	0	0	0	0	0
0	0	0	0	0	0	0	0	0
0	0	0	0	0	0	0	0	0
0	0	0	0	0	0	0	0	0
0	0	0	1	1	0	0	0	0
0	0	0	0	0	0	0	0	0
0	0	0	0	0	0	0	0	0
0	0	0	1	1	0	0	0	0
0	0	0	0	0	0	0	0	0
0	0	0	0	0	0	0	0	0
0	0	0	0	0	0	0	0	0
0	0	0	0	0	0	0	0	0
0	1	0	0	0	0	0	0	0
0	0	0	0	0	0	0	0	0
0	0	0	0	0	0	0	0	0
0	0	0	0	0	0	0	0	0
0	1	0	0	0	0	0	0	0
0	0	0	0	0	0	0	0	0
0	0	0	0	0	0	0	0	0
0	0	0	0	0	0	0	0	0
0	0	0	0	0	0	0	0	0
0	0	0	0	0	0	0	0	0
0	0	0	0	0	0	0	0	0
0	0	0	1	0	0	0	1	0
0	0	0	1	1	0	0	0	1
0	0	0	0	0	0	0	0	0
0	0	0	0	0	0	0	0	0
0	1	0	1	0	0	0	1	0
0	0	0	1	1	0	0	0	0
0	0	0	1	1	0	1	0	0

0	0	0	0	0	0	0	0	0
0	0	0	0	0	0	0	0	0
0	0	0	0	0	0	0	0	0
0	0	0	0	0	0	0	0	0
0	0	0	0	0	0	0	0	0
0	0	0	1	0	0	0	1	0
0	0	0	0	0	0	0	0	0
0	0	0	1	1	0	0	1	0
0	0	0	0	0	0	0	0	0
0	0	0	1	1	0	0	0	0
0	0	0	1	0	0	0	1	0
0	1	0	0	0	0	0	0	0
0	0	0	0	0	0	0	0	0
0	0	0	0	0	0	0	0	0
0	0	0	0	0	0	0	0	0
0	0	0	1	1	0	0	1	0
0	0	0	0	0	0	0	0	0
0	0	0	1	1	0	0	0	0
0	0	0	0	0	0	0	0	0
0	0	0	0	0	0	0	0	0
0	0	0	1	1	0	0	0	0
0	0	0	0	0	0	0	0	0
0	0	0	0	0	0	0	0	0
0	0	0	0	0	0	0	0	0
0	0	0	1	1	0	0	0	0
0	0	0	0	0	0	0	0	0
0	0	0	1	0	0	0	1	0
0	0	0	0	0	0	0	0	0
0	0	0	0	0	0	0	0	0
0	0	0	0	0	0	0	0	0
0	0	0	0	0	0	0	0	0
0	0	0	0	0	0	0	0	0
0	0	0	0	0	0	0	0	0
0	0	0	1	1	0	0	0	0
0	0	0	0	0	0	0	0	0
0	0	0	1	1	0	0	0	0
0	0	0	0	0	0	0	0	0
0	0	0	0	0	0	0	0	0
0	0	0	1	1	0	0	0	0
0	0	0	0	0	0	0	0	1
0	0	0	0	0	0	0	0	0
0	0	0	0	0	0	0	0	0
0	0	0	1	1	0	0	0	0
0	0	0	0	0	0	0	0	0
0	0	0	0	0	0	0	0	0
0	0	0	0	0	0	0	0	0
0	0	0	0	0	0	0	0	0
0	0	0	1	1	0	0	0	0
0	0	0	1	1	0	0	1	0
0	0	0	0	0	0	0	0	0
0	0	0	0	0	0	0	0	0
0	0	0	0	0	0	0	0	0
0	0	0	0	0	0	0	0	0
0	0	0	0	0	0	0	0	0

0	0	0	0	0	0	0	0	0
0	0	0	1	1	0	0	0	0
0	1	0	1	0	0	0	1	0
0	0	0	0	0	0	0	0	0
0	0	0	0	0	0	0	0	0
0	0	0	0	0	0	0	0	0
0	0	0	0	0	0	0	0	0
0	0	0	0	0	0	0	0	0
0	0	0	1	1	0	0	0	0
0	0	0	1	1	0	0	0	0
0	0	0	0	0	0	0	0	0
0	0	0	0	0	0	0	0	0
0	0	0	0	0	0	0	0	0
0	0	0	1	0	0	0	1	0
0	1	0	0	0	0	0	0	0
0	0	0	0	0	0	0	0	0
0	0	0	0	0	0	0	0	0
0	0	0	0	0	0	0	0	0
0	0	0	0	0	0	0	0	0
0	0	0	0	0	0	0	0	0
1	0	0	0	0	0	0	0	0
0	1	0	0	0	0	0	0	0
0	0	0	1	0	0	0	1	0
0	0	0	1	0	0	0	1	0
0	0	0	0	0	0	0	0	0
0	0	0	1	0	0	0	1	0
0	0	0	0	0	0	0	0	0
0	0	0	0	0	0	0	0	0
0	0	0	0	0	0	0	0	0
0	0	0	1	0	0	0	1	0
0	0	0	0	0	0	0	0	0
0	0	0	1	1	0	0	0	1
0	0	0	1	1	0	0	1	0
0	0	0	0	0	0	0	0	0
0	0	0	0	0	0	0	0	0
0	1	0	0	0	0	0	0	0
0	0	0	0	0	0	0	0	0
0	0	0	0	0	0	0	0	0
0	0	0	1	1	0	0	0	0
0	0	0	1	1	0	0	0	0
0	0	0	0	0	0	0	0	0
0	0	0	0	0	0	0	0	0
0	0	0	0	0	0	0	0	0
0	0	0	1	1	0	0	0	0
0	0	0	0	0	0	0	0	0
0	0	0	0	0	0	0	0	0
1	0	0	1	0	0	0	1	0
0	0	0	1	0	0	0	1	0
0	0	0	1	1	0	0	0	0
0	0	0	0	0	0	0	0	0
0	0	0	0	0	0	0	0	0
0	0	0	0	0	0	0	0	0
0	0	0	0	0	0	0	0	0
0	0	0	0	0	0	0	0	0

1	0	0	0	0	0	0	0	0
0	0	0	0	0	0	0	0	0
0	0	0	0	0	0	0	0	0
0	0	0	0	0	0	0	0	0
0	0	0	0	0	0	0	0	0
0	0	0	1	0	0	0	1	0
0	0	0	1	0	0	0	1	0
0	0	0	1	1	0	0	0	0
0	0	0	1	1	0	0	0	0
0	1	0	0	0	0	0	0	0
0	0	0	1	0	0	0	1	0
0	0	0	0	0	0	0	0	0
0	0	0	1	0	0	0	1	0
0	0	0	0	0	0	0	0	0
0	0	0	1	1	0	0	0	0
0	0	0	0	0	0	0	0	0
0	0	0	0	0	0	0	0	0
0	0	0	0	0	0	0	0	0
0	0	0	1	0	0	0	1	0
0	0	0	0	0	0	0	0	0
0	0	0	0	0	0	0	0	0
0	0	0	0	0	0	0	0	0
0	0	0	0	0	0	0	0	0
0	0	0	0	0	0	0	0	0
0	0	0	0	0	0	0	0	0
0	0	0	0	0	0	0	0	0
0	0	0	0	0	0	0	0	0
0	0	0	0	0	0	0	0	0
0	0	0	1	0	0	0	1	0
0	0	0	0	0	0	0	0	0
0	0	0	0	0	0	0	0	0
0	0	0	0	0	0	0	0	0
0	1	0	0	0	0	0	0	0
0	0	0	0	0	0	0	0	0
0	0	0	0	0	0	0	0	0
0	0	0	0	0	0	0	0	0
0	1	0	0	0	0	0	0	0
0	0	0	0	0	0	0	0	0
0	0	0	0	0	0	0	0	0
0	0	0	0	0	0	0	0	0
0	0	0	0	0	0	0	0	0
0	0	0	0	0	0	0	0	0
0	1	0	0	0	0	0	0	0
0	1	0	0	0	0	0	0	0
0	0	0	0	0	0	0	0	0
0	0	0	0	0	0	0	0	0
0	0	0	0	0	0	0	0	0
0	0	0	0	0	0	0	0	0
0	0	0	0	0	0	0	0	0
0	0	0	0	0	0	0	0	0
0	1	0	1	0	0	0	1	0
0	0	0	0	0	0	0	0	0
0	0	0	0	0	0	0	0	0
0	0	0	0	0	0	0	0	0

0	0	0	0	0	0	0	0	0
0	0	0	0	0	0	0	0	0
0	0	0	0	0	0	0	0	0
0	0	0	0	0	0	0	0	0
0	0	0	0	0	0	0	0	0
0	0	0	0	0	0	0	0	0
0	0	0	0	0	0	0	0	0
0	0	0	0	0	0	0	0	0
0	0	0	0	0	0	0	0	0
0	0	0	0	0	0	0	0	0
0	0	0	0	0	0	0	0	0
0	0	0	0	0	0	0	0	0
0	0	0	0	0	0	0	0	0
0	0	0	0	0	0	0	0	0
0	0	0	0	0	0	0	0	0
0	0	0	0	0	0	0	0	0
0	0	0	0	0	0	0	0	0
0	0	0	0	0	0	0	0	0
0	0	0	0	0	0	0	0	0
0	0	0	0	0	0	0	0	0
0	0	0	0	0	0	0	0	0
0	0	0	0	0	0	0	0	0
0	0	0	0	0	0	0	0	0
0	0	0	0	0	0	0	0	0
0	0	0	0	0	0	0	0	0
0	0	0	1	0	0	0	1	0
0	0	0	0	0	0	0	0	0
0	0	0	0	0	0	0	0	0
0	1	0	1	1	0	0	0	0
0	0	0	0	0	0	0	0	0
0	0	0	0	0	0	0	0	0
0	0	0	0	0	0	0	0	0
0	0	0	0	0	0	0	0	0
0	0	0	0	0	0	0	0	0
0	0	0	0	0	0	0	0	0
0	0	0	0	0	0	0	0	0
0	0	0	0	0	0	0	0	0
0	0	0	0	0	0	0	0	0
0	0	0	0	0	0	0	0	0
0	0	0	1	1	0	0	0	0
0	0	0	0	0	0	0	0	0
0	0	0	1	1	0	0	0	0
0	0	0	0	0	0	0	0	0
0	0	0	0	0	0	0	0	0
0	0	0	0	0	0	0	0	0
0	0	0	0	0	0	0	0	0
0	0	0	0	0	0	0	0	0
0	0	0	0	0	0	0	0	0
0	0	0	0	0	0	0	0	0
0	0	0	0	0	0	0	0	0
0	0	0	0	0	0	0	0	0
0	0	0	0	0	0	0	0	0
0	0	0	0	0	0	0	0	0
0	0	0	1	0	0	1	0	0

0	0	0	0	0	0	0	0	0
0	0	0	0	0	0	0	0	0
0	0	0	0	0	0	0	0	0
0	0	0	0	0	0	0	0	0
0	1	0	0	0	0	0	0	0
0	0	0	0	0	0	0	0	0
0	0	0	0	0	0	0	0	0
0	0	0	0	0	0	0	0	0
0	0	0	0	0	0	0	0	0
0	0	0	0	0	0	0	0	0
0	0	0	0	0	0	0	0	0
0	0	0	0	0	0	0	0	0
0	0	0	0	0	0	0	0	0
0	0	0	0	0	0	0	0	0
0	0	0	0	0	0	0	0	0
0	0	0	0	0	0	0	0	0
0	0	0	0	0	0	0	0	0
0	0	0	0	0	0	0	0	0
0	0	0	1	1	0	0	1	0
0	0	0	1	0	0	0	1	0
0	1	0	1	1	0	0	1	0
0	0	0	0	0	0	0	0	0
0	0	0	0	0	0	0	0	0
0	0	0	0	0	0	0	0	0
0	0	0	1	1	0	0	1	0
0	1	0	1	0	0	0	1	0
0	0	0	0	0	0	0	0	0
0	0	0	0	0	0	0	0	0
0	0	0	1	1	0	0	0	0
0	0	0	0	0	0	0	0	0
0	0	0	0	0	0	0	0	0
0	0	0	0	0	0	0	0	0
0	0	0	1	1	0	0	0	0
0	0	0	0	0	0	0	0	0
0	0	0	1	0	0	0	1	0
0	0	0	0	0	0	0	0	0
0	0	0	0	0	0	0	0	0
0	0	0	0	0	0	0	0	0
0	0	0	0	0	0	0	0	0
0	0	0	0	0	0	0	0	0
0	0	0	0	0	0	0	0	0
0	0	0	1	1	0	0	0	0
0	0	0	0	0	0	0	0	0
0	0	0	0	0	0	0	0	0
0	0	0	0	0	0	0	0	0
0	0	0	0	0	0	0	0	0
0	0	0	0	0	0	0	0	0
0	0	0	1	0	0	0	1	0
0	0	0	1	1	0	0	0	0
0	0	0	1	1	0	0	0	0
0	0	0	0	0	0	0	0	0
0	0	0	0	0	0	0	0	0
0	0	0	0	0	0	0	0	0
0	0	0	0	0	0	0	0	0

0	0	0	0	0	0	0	0	0
0	0	0	0	0	0	0	0	0
0	0	0	0	0	0	0	0	0
0	0	0	0	0	0	0	0	0
0	0	0	0	0	0	0	0	0
1	0	0	0	0	0	0	0	0
0	0	0	0	0	0	0	0	0
1	0	0	0	0	0	0	0	0
0	0	0	0	0	0	0	0	0
0	0	0	0	0	0	0	0	0
0	0	0	0	0	0	0	0	0
0	0	0	0	0	0	0	0	0
0	0	0	0	0	0	0	0	0
0	0	0	1	1	0	0	0	0
0	0	0	0	0	0	0	0	0
0	0	0	0	0	0	0	0	0
0	0	0	0	0	0	0	0	0
0	0	0	0	0	0	0	0	0
0	0	0	0	0	0	0	0	0
0	0	0	0	0	0	0	0	0
0	0	0	0	0	0	0	0	0
0	0	0	0	0	0	0	0	0
0	0	0	0	0	0	0	0	0
0	0	0	1	1	0	1	0	0
0	0	0	0	0	0	0	0	0
0	0	0	0	0	0	0	0	0
0	0	0	0	0	0	0	0	0
0	0	0	0	0	0	0	0	0
0	0	0	0	0	0	0	0	0
0	0	0	0	0	0	0	0	0
0	0	0	0	0	0	0	0	0
0	0	0	0	0	0	0	0	0
0	0	0	0	0	0	0	0	0
0	0	0	0	0	0	0	0	0
1	0	0	0	0	0	0	0	0
0	0	0	0	0	0	0	0	0
0	0	0	1	1	0	0	0	0
0	0	0	0	0	0	0	0	0
0	0	0	1	0	0	1	0	0
0	0	0	0	0	0	0	0	0
0	0	0	1	1	0	0	0	0
0	0	0	1	1	0	0	1	0
0	0	0	0	0	0	0	0	0
0	0	0	1	1	0	0	1	0
0	0	0	0	0	0	0	0	0
0	0	0	0	0	0	0	0	0
0	0	0	1	0	0	0	1	0
0	1	0	0	0	0	0	0	0
0	0	0	0	0	0	0	0	0
0	0	0	1	1	0	0	1	0
0	0	0	0	0	0	0	0	0
0	0	0	0	0	0	0	0	0
0	0	0	1	0	0	0	1	0
0	0	0	1	1	0	0	0	0

0	0	0	0	0	0	0	0	0
0	0	0	0	0	0	0	0	0
0	0	0	0	0	0	0	0	0
0	0	0	0	0	0	0	0	0
0	0	0	0	0	0	0	0	0
0	0	0	0	0	0	0	0	0
0	0	0	0	0	0	0	0	0
0	0	0	0	0	0	0	0	0
0	0	0	0	0	0	0	0	0
0	0	0	0	0	0	0	0	0
0	0	0	1	1	0	0	0	0
0	0	0	0	0	0	0	0	0
0	0	0	0	0	0	0	0	0
0	0	0	0	0	0	0	0	0
0	0	0	1	0	0	0	1	0
0	0	0	1	1	0	0	0	0
0	1	0	0	0	0	0	0	0
0	0	0	0	0	0	0	0	0
0	0	0	0	0	0	0	0	0
0	0	0	0	0	0	0	0	0
0	0	0	0	0	0	0	0	0
0	0	0	0	0	0	0	0	0
0	0	0	0	0	0	0	0	0
0	0	0	0	0	0	0	0	0
0	1	0	1	1	0	0	0	0
0	0	0	1	0	0	0	1	0
0	0	0	0	0	0	0	0	0
0	0	0	0	0	0	0	0	0
0	0	0	0	0	0	0	0	0
0	0	0	0	0	0	0	0	0
0	0	0	0	0	0	0	0	0
0	0	0	1	0	0	0	1	0
0	0	0	0	0	0	0	0	0
0	0	0	1	1	0	0	0	0
0	0	0	0	0	0	0	0	0
0	0	0	0	0	0	0	0	0
0	0	0	0	0	0	0	0	0
0	0	0	0	0	0	0	0	0
0	0	0	0	0	0	0	0	0
0	0	0	0	0	0	0	0	0
0	0	0	0	0	0	0	0	0
0	0	0	1	0	0	0	1	0
0	0	0	0	0	0	0	0	0
0	0	0	1	0	0	0	1	0
0	0	0	1	1	0	0	0	0
0	0	0	0	0	0	0	0	0
0	0	0	0	0	0	0	0	0
0	0	0	0	0	0	0	0	0
0	0	0	0	0	0	0	0	0
0	0	0	0	0	0	0	0	0
0	1	0	0	0	0	0	0	0
0	0	0	0	0	0	0	0	0
0	0	0	0	0	0	0	0	0
0	0	0	0	0	0	0	0	0

0	0	0	0	0	0	0	0	0
0	0	0	0	0	0	0	0	0
0	0	0	0	0	0	0	0	0
0	0	0	0	0	0	0	0	0
0	0	0	1	1	0	0	0	0
0	0	0	0	0	0	0	0	0
0	0	0	1	1	0	0	0	0
0	0	0	0	0	0	0	0	0
0	0	0	0	0	0	0	0	0
0	0	0	0	0	0	0	0	0
0	0	0	0	0	0	0	0	0
0	0	0	0	0	0	0	0	0
0	0	0	1	1	0	0	0	0
0	0	0	0	0	0	0	0	0
0	0	0	0	0	0	0	0	0
0	0	0	0	0	0	0	0	0
0	0	0	0	0	0	0	0	0
0	0	0	0	0	0	0	0	0
0	0	0	0	0	0	0	0	0
0	0	0	0	0	0	0	0	0
0	0	0	0	0	0	0	0	0
0	0	0	0	0	0	0	0	0
0	0	0	0	0	0	0	0	0
0	0	0	0	0	0	0	0	0
0	0	0	1	0	0	0	1	0
0	0	0	0	0	0	0	0	0
0	0	0	0	0	0	0	0	0
0	0	0	0	0	0	0	0	0
0	0	0	0	0	0	0	0	0
0	0	0	0	0	0	0	0	0
0	0	0	1	1	0	0	0	0
0	0	0	0	0	0	0	0	0
0	0	0	0	0	0	0	0	0
0	0	0	0	0	0	0	0	0
0	1	0	0	0	0	0	0	0
0	0	0	0	0	0	0	0	0
0	0	0	0	0	0	0	0	0
0	0	0	1	1	0	0	0	0
0	0	0	0	0	0	0	0	0
0	0	0	0	0	0	0	0	0
0	0	0	0	0	0	0	0	0
0	0	0	0	0	0	0	0	0
0	0	0	1	1	0	0	1	0
0	0	0	0	0	0	0	0	0
0	0	0	0	0	0	0	0	0
0	0	0	0	0	0	0	0	0
0	0	0	0	0	0	0	0	0
0	0	0	0	0	0	0	0	1
0	0	0	0	0	0	0	0	0
0	0	0	0	0	0	0	0	0
0	0	0	0	0	0	0	0	0
0	0	0	0	0	0	0	0	0
0	0	0	1	1	0	0	0	0
0	0	0	0	0	0	0	0	0

0	0	0	0	0	0	0	0	0
0	0	0	0	0	0	0	0	0
0	0	0	0	0	0	0	0	0
0	0	0	0	0	0	0	0	0
0	0	0	0	0	0	0	0	0
0	0	0	0	0	0	0	0	0
0	0	0	0	0	0	0	0	0
0	0	0	0	0	0	0	0	0
0	0	0	1	0	0	0	1	0
0	0	0	0	0	0	0	0	0
0	0	0	1	1	0	1	0	0
0	0	0	1	1	0	0	0	0
0	0	0	0	0	0	0	0	0
0	0	0	1	1	0	0	1	0
0	0	0	0	0	0	0	0	0
0	0	0	0	0	0	0	0	0
0	0	0	1	1	0	0	1	0
0	0	0	0	0	0	0	0	0
0	0	0	0	0	0	0	0	0
0	0	0	1	1	0	0	0	0
0	0	0	0	0	0	0	0	0
0	0	0	0	0	0	0	0	0
0	0	0	0	0	0	0	0	0
0	0	0	0	0	0	0	0	0
0	0	0	0	0	0	0	0	0
0	0	0	0	0	0	0	0	0
0	0	0	0	0	0	0	0	0
0	0	0	0	0	0	0	0	0
0	0	0	0	0	0	0	0	0
0	0	0	0	0	0	0	0	0
0	0	0	1	1	0	0	0	0
0	0	0	0	0	0	0	0	0
0	0	0	0	0	0	0	0	0
0	0	0	1	1	0	0	0	0
0	0	0	1	0	0	0	1	0
0	0	0	1	1	0	0	0	0
0	0	0	0	0	0	0	0	0
0	0	0	1	1	0	0	1	0
0	0	0	0	0	0	0	0	0
0	0	0	1	0	0	0	1	0
0	0	0	0	0	0	0	0	0
0	0	0	1	1	0	0	0	0
0	0	0	0	0	0	0	0	0
0	0	0	0	0	0	0	0	0
0	0	0	0	0	0	0	0	0
0	0	0	1	1	0	0	0	0
0	0	0	0	0	0	0	0	0
0	0	0	1	1	0	0	1	0
0	0	0	0	0	0	0	0	0
0	0	0	1	0	0	0	1	0
0	0	0	0	0	0	0	0	0
0	0	0	1	1	0	0	0	0
0	0	0	1	1	0	0	0	0
0	0	0	0	0	0	0	0	0

0	0	0	0	0	0	0	0	0
0	0	0	1	1	0	0	0	0
0	0	0	0	0	0	0	0	0
0	0	0	1	1	0	0	0	0
0	0	0	0	0	0	0	0	0
0	0	0	0	0	0	0	0	0
0	0	0	1	0	0	0	1	0
0	0	0	0	0	0	0	0	0
0	0	0	0	0	0	0	0	0
0	0	0	0	0	0	0	0	0
0	0	0	0	0	0	0	0	0
0	0	0	0	0	0	0	0	0
0	0	0	1	0	1	0	0	1
0	0	0	1	1	0	1	0	0
0	0	0	0	0	0	0	0	0
0	0	0	0	0	0	0	0	0
0	0	0	1	1	0	0	0	0
0	0	0	1	1	1	0	0	0
0	1	0	0	0	0	0	0	0
0	0	0	1	1	0	0	0	0
0	0	0	0	0	0	0	0	1
0	0	0	1	1	0	0	0	0
0	0	0	1	1	0	0	0	0
0	0	0	1	1	0	0	0	0
0	0	0	1	0	1	0	0	0
0	0	0	1	1	0	0	0	1
0	1	0	1	0	0	0	0	0
0	0	0	1	1	0	0	0	0
0	0	0	0	0	0	0	0	0
0	0	0	0	0	0	0	0	0
0	1	0	0	0	0	0	0	0
0	0	0	1	1	0	0	0	0
1	0	0	0	0	0	0	0	0
0	0	0	0	0	0	0	0	0
0	0	0	0	0	0	0	0	0
0	0	0	0	0	0	0	0	0
1	0	0	0	0	0	0	0	0
0	0	0	1	1	0	0	0	1
0	0	0	0	0	0	0	0	0
0	0	0	1	1	0	1	1	0
0	0	0	0	0	0	0	0	0
0	0	0	0	0	0	0	0	0
0	0	0	0	0	0	0	0	0
0	0	0	1	0	0	0	1	0
0	1	0	1	1	0	0	0	0
0	0	0	0	0	0	0	0	0
0	0	0	0	0	0	0	0	0
0	0	0	1	1	0	0	0	0
1	0	0	0	0	0	0	0	0
0	0	0	0	0	0	0	0	0
0	0	0	1	0	0	0	1	0
0	1	0	1	1	0	1	0	0
0	0	0	1	0	0	0	1	0
0	0	0	1	0	0	0	1	0

0	0	0	1	1	0	0	0	0
0	0	0	1	1	0	0	1	0
0	0	0	1	0	1	0	1	0
0	0	0	0	0	0	0	0	0
1	0	0	1	0	1	0	0	0
0	0	0	0	0	0	0	0	0
0	0	0	1	0	0	1	0	0
0	0	0	1	1	0	1	0	0
1	0	0	0	0	0	0	0	0
0	0	0	1	0	0	0	1	0
0	0	0	0	0	0	0	0	0
0	0	0	1	1	0	0	0	0
0	0	0	0	0	0	0	0	0
0	0	0	0	0	0	0	0	0
0	0	0	1	1	0	0	0	0
0	0	0	0	0	0	0	0	0
0	0	0	0	0	0	0	0	0
1	0	0	1	1	0	0	0	0
0	0	0	0	0	0	0	0	0
0	0	0	1	1	0	0	0	0
0	0	0	0	0	0	0	0	0
0	0	0	0	0	0	0	0	0
0	0	0	1	1	0	0	0	0
0	0	0	1	1	0	0	1	0
0	0	0	0	0	0	0	0	0
0	0	0	0	0	0	0	0	0
0	0	0	0	0	0	0	0	0
0	0	0	0	0	0	0	0	0
0	0	0	0	0	0	0	0	0
1	0	0	1	0	0	0	1	0
0	0	0	0	0	0	0	0	0
0	0	0	1	1	0	1	1	1
0	0	0	1	1	0	0	0	0
0	0	1	0	0	0	0	0	1
0	0	0	0	0	0	0	0	1
0	0	0	0	0	0	0	0	0
0	0	0	1	1	0	0	0	0
0	0	0	0	0	0	0	0	0
0	0	0	1	1	0	0	0	0
0	0	0	0	0	0	0	0	0
0	0	0	0	0	0	0	0	0
0	0	0	0	0	0	0	0	0
0	0	0	1	1	0	0	1	0
0	0	0	1	0	0	0	1	0
0	0	0	0	0	0	0	0	0
0	0	0	0	0	0	0	0	0
0	0	0	0	0	0	0	0	0
0	1	0	1	1	0	0	0	0
0	0	0	0	0	0	0	0	0
0	0	0	0	0	0	0	0	0
0	0	0	0	0	0	0	0	0
0	0	0	0	0	0	0	0	0
0	0	0	0	0	0	0	0	0
0	0	0	1	0	0	0	1	0

0	0	0	1	0	0	0	1	0
0	0	0	0	0	0	0	0	0
0	0	0	0	0	0	0	0	0
0	0	0	0	0	0	0	0	0
0	0	0	0	0	0	0	0	0
0	0	0	1	1	0	0	0	0
0	0	0	0	0	0	0	0	1
0	0	0	0	0	0	0	0	0
0	0	0	1	1	0	0	0	0
0	0	0	0	0	0	0	0	1
0	0	0	1	0	1	0	0	0
0	0	0	0	0	0	0	0	0
0	0	0	0	0	0	0	0	0
0	1	0	1	1	0	1	0	0
1	0	0	0	0	0	0	0	0
0	0	0	0	0	0	0	0	0
0	0	0	0	0	0	0	0	0
0	0	0	1	1	0	0	1	0
1	0	0	1	1	0	0	0	0
0	0	0	0	0	0	0	0	0
1	0	1	1	1	0	0	0	0
0	0	0	0	0	0	0	0	0
0	0	0	0	0	0	0	0	0
0	0	0	0	0	0	0	0	0
0	0	0	0	0	0	0	0	0
0	0	0	0	0	0	0	0	0
0	0	0	0	0	0	0	0	0
0	0	0	0	0	0	0	0	0
0	0	0	1	0	0	0	1	0
0	0	0	1	1	0	0	0	0
0	0	0	1	0	1	0	0	0
0	0	0	0	0	0	0	0	0
1	0	0	0	0	0	0	0	0
1	0	0	0	0	0	0	0	0
0	0	0	0	0	0	0	0	0
0	0	0	0	0	0	0	0	0
0	0	0	1	0	1	0	0	1
0	0	0	0	0	0	0	0	1
0	0	0	0	0	0	0	0	0
0	0	0	0	0	0	0	0	0
0	0	0	0	0	0	0	0	0
1	0	0	0	0	0	0	0	0
1	0	0	0	0	0	0	0	0
0	0	0	1	0	0	0	1	0
0	0	0	0	0	0	0	0	1
0	0	0	1	1	0	0	1	0
0	0	0	0	0	0	0	0	0
0	0	0	0	0	0	0	0	0
0	0	0	0	0	0	0	0	0
0	0	0	0	0	0	0	0	0
1	0	0	1	0	1	0	1	0
0	0	0	0	0	0	0	0	0
0	0	0	0	0	0	0	0	0
0	0	0	1	1	0	1	0	0

0	0	0	0	0	0	0	0	0
0	0	0	1	0	0	0	1	0
0	0	0	1	0	0	0	1	0
0	0	0	1	0	0	0	1	0
0	1	0	0	0	0	0	0	0
0	0	0	1	0	0	0	1	0
0	0	0	1	1	0	0	0	0
0	0	0	0	0	0	0	0	0
0	0	0	1	0	0	1	0	0
0	0	0	1	1	0	0	0	0
0	0	0	0	0	0	0	0	0
0	0	0	1	0	0	0	1	0
0	0	0	0	0	0	0	0	0
0	0	0	0	0	0	0	0	1
0	0	0	1	1	1	0	0	0
0	0	0	0	0	0	0	0	0
0	0	0	1	1	0	0	0	0
0	0	0	1	0	0	0	1	0
0	0	0	0	0	0	0	0	0
0	0	0	1	0	0	0	1	0
0	0	0	0	0	0	0	0	0
0	0	0	1	1	1	0	0	0
0	0	0	0	0	0	0	0	0
0	0	0	1	0	0	0	1	0
0	0	0	0	0	0	0	0	1
0	0	0	1	1	0	0	0	0
0	0	0	0	0	0	0	0	0
0	0	0	0	0	0	0	0	0
0	0	0	0	0	0	0	0	0
0	0	0	1	0	0	0	1	0
0	1	0	1	0	0	0	1	0
0	0	0	1	0	0	0	1	0
0	0	0	0	0	0	0	0	0
0	0	0	0	0	0	0	0	0
0	0	0	0	0	0	0	0	0
0	0	0	1	0	0	0	1	0
0	0	0	0	0	0	0	0	0
0	0	0	0	0	0	0	0	0
0	0	0	1	1	1	0	1	0
0	0	0	0	0	0	0	0	0
0	0	0	1	0	0	1	0	0
0	0	0	0	0	0	0	0	0
0	0	0	1	1	0	0	0	0
0	0	0	0	0	0	0	0	0
0	0	0	0	0	0	0	0	0
0	1	0	1	1	0	0	1	0
0	0	0	0	0	0	0	0	0
0	0	0	1	0	0	0	1	0
0	0	0	1	0	1	0	1	0
0	0	0	1	0	1	0	0	0
0	0	0	0	0	0	0	0	0
0	0	0	0	0	0	0	0	0
0	0	0	1	1	1	0	0	0
0	0	0	1	0	0	0	1	0

0	0	0	0	0	0	0	0	0
0	0	1	1	1	0	0	0	0
0	0	0	1	0	0	0	1	0
0	0	0	0	0	0	0	0	0
1	0	0	1	0	0	0	1	0
0	0	0	1	1	1	0	0	0
0	0	0	0	0	0	0	0	0
0	0	0	0	0	0	0	0	0
0	0	0	0	0	0	0	0	0
0	0	0	0	0	0	0	0	0
0	0	0	0	0	0	0	0	0
0	0	0	1	1	0	0	1	0
0	0	0	0	0	0	0	0	0
1	0	0	1	0	1	0	1	0
1	0	0	0	0	0	0	0	0
0	0	0	1	1	0	0	0	0
0	0	0	0	0	0	0	0	0
0	1	0	1	0	0	1	1	1
0	0	0	1	1	0	0	0	0
0	0	0	0	0	0	0	0	0
0	0	0	1	0	1	0	0	0
0	0	0	1	1	0	0	1	0
0	0	0	1	1	0	0	0	0
0	1	0	0	0	0	0	0	0
0	0	0	1	0	0	0	1	0
0	0	0	0	0	0	0	0	0
0	0	0	1	0	0	0	1	0
0	0	0	1	0	0	0	1	0
0	0	0	1	0	0	0	1	0
0	0	0	0	0	0	0	0	0
0	0	0	0	0	0	0	0	0
0	0	0	0	0	0	0	0	0
0	0	0	1	0	0	1	0	0
0	0	0	0	0	0	0	0	0
0	0	0	1	0	0	0	1	0
0	0	0	0	0	0	0	0	1
0	0	0	1	0	0	1	0	0
1	0	0	0	0	0	0	0	0
0	0	0	0	0	0	0	0	0
0	0	0	1	0	0	0	1	0
0	0	0	0	0	0	0	0	0
0	0	0	1	0	0	1	0	0
0	0	0	0	0	0	0	0	0
0	0	0	1	0	1	0	1	0
0	0	0	1	1	0	0	0	0
0	0	0	1	0	0	0	1	0
0	0	0	1	1	0	0	1	0
0	0	0	0	0	0	0	0	0
0	0	0	0	0	0	0	0	0
0	0	0	1	1	0	1	0	0
0	0	0	0	0	0	0	0	0
0	0	0	0	0	0	0	0	0
0	0	0	1	1	0	1	0	0
0	0	0	1	1	0	0	0	0

0	0	0	1	0	0	0	1	0
0	0	0	1	0	0	0	1	0
0	0	0	0	0	0	0	0	1
0	0	0	1	1	0	0	1	0
0	0	0	0	0	0	0	0	0
0	0	0	1	0	0	0	1	0
0	0	0	0	0	0	0	0	0
0	0	0	1	0	0	0	1	0
0	0	0	1	0	0	0	1	0
0	0	0	0	0	0	0	0	0
0	0	0	1	0	0	0	1	0
0	0	0	0	0	0	0	0	0
0	0	0	1	0	1	0	1	0
0	0	0	1	1	0	0	1	0
0	0	0	0	0	0	0	0	0
0	0	0	1	0	0	1	1	0
0	0	0	0	0	0	0	0	0
0	0	0	0	0	0	0	0	0
0	0	0	1	1	0	0	1	0
0	0	0	1	0	0	1	0	0
0	0	0	0	0	0	0	0	0
0	0	0	0	0	0	0	0	0
0	0	0	0	0	0	0	0	0
0	0	0	0	0	0	0	0	0
0	0	0	1	0	1	1	0	0
0	0	0	0	0	0	0	0	0
0	0	0	0	0	0	0	0	0
0	0	0	0	0	0	0	0	0
0	0	0	0	0	0	0	0	0
0	0	0	0	0	0	0	0	0
0	0	0	0	0	0	0	0	0
0	0	0	0	0	0	0	0	0
0	0	0	1	0	0	0	1	0
0	0	0	1	1	0	0	0	0
0	0	0	1	0	0	0	1	0
0	0	0	1	0	1	0	1	0
0	0	0	0	0	0	0	0	0
0	0	0	1	0	0	1	0	0
0	0	0	0	0	0	0	0	0
0	0	0	1	0	0	0	1	0
0	0	0	0	0	0	0	0	0
0	0	0	1	0	0	0	1	0
0	0	0	0	0	0	0	0	0
0	0	0	0	0	0	0	0	0
0	0	0	1	0	0	0	1	0
0	0	0	0	0	0	0	0	0
0	0	0	0	0	0	0	0	0
0	0	0	1	0	0	0	1	0
0	0	0	1	0	1	0	0	0
1	0	1	0	0	0	0	0	0
0	0	0	1	1	0	0	0	0
0	0	0	1	0	0	0	1	0
0	1	0	0	0	0	0	0	0
0	0	0	0	0	0	0	0	0

0	0	0	1	0	1	1	0	0
0	0	0	1	0	0	0	1	0
0	0	0	1	0	0	0	1	0
0	0	0	0	0	0	0	0	0
0	0	1	1	0	1	0	0	0
0	0	0	0	0	0	0	0	0
0	0	0	1	0	0	0	1	0
0	0	0	0	0	0	0	0	0
0	0	0	0	0	0	0	0	0
0	0	0	1	0	1	0	0	0
0	0	0	1	0	0	0	1	0
0	0	0	0	0	0	0	0	0
1	0	0	1	1	0	0	0	0
0	1	0	1	1	0	1	0	0
0	0	0	0	0	0	0	0	0
1	0	0	1	0	1	0	1	0
0	0	0	0	0	0	0	0	0
0	1	0	1	0	0	0	1	0
0	0	0	1	0	0	0	1	0
0	0	0	1	0	0	0	1	0
0	0	0	0	0	0	0	0	0
0	0	0	0	0	0	0	0	0
0	0	0	0	0	0	0	0	0
0	0	0	1	1	0	1	1	0
0	0	0	0	0	0	0	0	0
0	0	0	1	0	0	0	1	0
0	0	0	1	1	0	0	0	0
0	0	0	0	0	0	0	0	0
0	0	0	0	0	0	0	0	0
0	0	0	0	0	0	0	0	0
0	0	0	0	0	0	0	0	0
0	0	0	0	0	0	0	0	0
0	0	0	0	0	0	0	0	0
0	0	0	0	0	0	0	0	0
0	0	0	1	0	0	0	1	0
0	0	0	1	0	0	0	1	0
0	0	0	1	0	0	0	1	0
0	0	0	1	1	0	0	1	0
0	0	1	1	1	0	0	1	0
0	0	0	1	0	0	0	1	0
0	0	0	1	0	0	0	1	0
0	0	0	0	0	0	0	0	0
0	0	0	1	1	0	0	0	0
0	0	0	0	0	0	0	0	0
0	0	0	1	0	0	0	1	0
0	0	0	1	1	0	0	0	0
0	0	0	0	0	0	0	0	0
0	0	0	0	0	0	0	0	0
0	0	0	0	0	0	0	0	0
0	0	0	1	0	0	0	1	0
0	0	0	1	0	0	0	1	0
1	0	0	0	0	0	0	0	0
0	0	0	0	0	0	0	0	0
0	0	0	0	0	0	0	0	0

0	0	0	0	0	0	0	0	0
0	0	0	1	1	0	0	0	0
0	0	0	0	0	0	0	0	0
0	0	0	0	0	0	0	0	0
0	0	0	1	0	0	0	1	0
0	0	0	0	0	0	0	0	0
0	0	0	1	0	0	1	0	0
0	0	0	1	0	0	0	1	0
0	0	0	0	0	0	0	0	0
0	0	0	1	1	0	0	0	0
0	0	0	0	0	0	0	0	0
0	0	0	0	0	0	0	0	0
0	0	0	0	0	0	0	0	0
1	0	0	1	1	0	0	1	0
0	0	0	0	0	0	0	0	0
0	0	0	1	1	0	0	1	0
0	0	0	1	1	0	0	0	0
0	0	0	0	0	0	0	0	0
0	0	0	0	0	0	0	0	0
0	0	0	0	0	0	0	0	0
0	0	0	0	0	0	0	0	0
0	0	0	1	1	0	0	1	0
0	0	0	0	0	0	0	0	0
0	0	0	0	0	0	0	0	0
0	0	0	1	0	0	0	1	0
0	0	0	0	0	0	0	0	0
0	1	0	0	0	0	0	0	0
0	0	0	1	0	1	0	1	0
0	0	0	1	0	0	0	1	0
0	0	0	1	0	0	1	0	0
0	0	0	0	0	0	0	0	0
0	0	0	1	0	0	0	1	0
0	0	0	0	0	0	0	0	0
0	0	0	1	0	0	0	1	0
0	0	0	1	0	0	0	1	0
0	1	0	0	0	0	0	0	0
0	0	0	1	0	0	0	1	0
0	0	0	0	0	0	0	0	0
0	0	0	0	0	0	0	0	0
0	0	0	1	1	0	0	1	0
0	0	0	0	0	0	0	0	0
0	0	1	0	0	0	0	0	0
0	0	0	0	0	0	0	0	0
0	0	0	0	0	0	0	0	0
0	0	0	0	0	0	0	0	0
0	0	0	1	0	0	0	1	0
0	0	0	0	0	0	0	0	0
0	0	0	0	0	0	0	0	0
0	0	0	0	0	0	0	0	0
0	0	0	1	0	0	0	1	0
0	0	0	0	0	0	0	0	0
0	0	0	0	0	0	0	0	0
0	0	0	0	0	0	0	0	0
0	0	0	0	0	0	0	0	0

0	0	0	1	0	0	0	1	0
1	0	0	0	0	0	0	0	0
0	0	0	1	1	0	0	0	0
0	0	0	0	0	0	0	0	0
1	0	0	0	0	0	0	0	0
0	0	1	0	0	0	0	0	0
0	0	0	0	0	0	0	0	0
1	0	0	1	1	0	0	0	0
0	0	0	1	0	0	0	1	0
0	0	0	1	0	0	0	1	0
0	0	0	1	1	0	0	0	0
0	0	0	1	1	0	0	1	0
0	0	0	1	0	0	0	1	0
0	0	0	1	1	0	0	0	0
0	0	0	0	0	0	0	0	0
0	0	0	0	0	0	0	0	0
0	0	0	0	0	0	0	0	0
0	0	0	1	0	0	1	0	0
0	1	0	0	0	0	0	0	0
0	1	0	0	0	0	0	0	0
0	0	0	0	0	0	0	0	0
0	0	0	1	1	0	1	1	0
0	0	0	0	0	0	0	0	0
0	0	0	0	0	0	0	0	0
1	0	0	1	1	0	0	0	0
0	0	0	0	0	0	0	0	0
0	0	0	0	0	0	0	0	0
0	0	0	0	0	0	0	0	0
0	0	0	0	0	0	0	0	0
0	0	0	1	1	0	0	0	0
0	0	0	0	0	0	0	0	0
0	0	0	0	0	0	0	0	0
0	0	0	0	0	0	0	0	0
0	0	0	1	1	0	0	0	0
0	0	0	0	0	0	0	0	0
0	0	0	0	0	0	0	0	0
0	0	0	0	0	0	0	0	0
0	0	0	0	0	0	0	0	0
0	0	0	0	0	0	0	0	0
0	0	0	0	0	0	0	0	0
0	1	0	1	0	0	1	0	0
0	1	0	1	1	0	1	0	0
1	0	0	0	0	0	0	0	0
0	0	0	0	0	0	0	0	0
0	0	0	0	0	0	0	0	0
0	0	0	1	1	0	0	0	0
0	0	0	0	0	0	0	0	0
0	0	0	0	0	0	0	0	0
0	0	0	0	0	0	0	0	0
0	1	0	0	0	0	0	0	0
0	0	0	1	0	1	0	0	0
0	0	0	1	1	0	0	0	0
0	0	0	1	1	0	0	0	0
1	0	0	1	1	1	0	1	0

0	0	0	1	1	1	0	0	0
0	0	0	1	0	1	0	0	0
0	0	0	1	0	0	1	0	0
0	0	0	0	0	0	0	0	0
0	0	0	0	0	0	0	0	0
0	0	0	1	0	0	0	1	0
0	0	0	0	0	0	0	0	0
1	0	0	0	0	0	0	0	0
0	0	0	1	1	0	0	0	0
0	0	0	1	1	0	1	1	0
0	0	0	1	1	1	0	0	0
0	0	0	1	1	0	0	0	0
0	0	0	0	0	0	0	0	0
1	0	0	1	0	1	0	1	0
0	0	0	1	1	1	0	0	0
0	0	0	0	0	0	0	0	0
0	0	0	1	0	0	0	1	0
0	0	0	1	0	0	0	1	0
1	0	0	1	1	1	1	1	1
0	0	0	1	1	0	1	0	0
0	0	0	1	0	0	0	1	0
0	0	0	1	1	0	0	0	0
0	0	0	0	0	0	0	0	0
0	0	0	1	0	0	1	0	0
0	0	0	0	0	0	0	0	0
0	0	0	1	1	0	0	0	0
0	0	0	1	0	1	0	0	0
0	0	0	0	0	0	0	0	0
0	0	0	0	0	0	0	0	0
0	0	0	1	1	0	0	0	0
0	0	0	0	0	0	0	0	0
0	0	0	1	0	0	1	0	0
0	0	0	1	1	0	0	0	0
0	0	0	0	0	0	0	0	0
0	0	0	0	0	0	0	0	0
0	0	0	0	0	0	0	0	0
1	0	0	1	0	0	0	1	0
0	0	0	1	1	0	0	1	0
0	0	0	0	0	0	0	0	0
0	0	1	1	1	0	0	0	0
0	0	0	0	0	0	0	0	0
0	0	0	1	0	0	0	1	0
0	0	0	1	0	1	0	0	0
0	0	0	1	0	1	0	1	0
0	0	0	0	0	0	0	0	0
0	0	1	1	0	1	0	0	0
0	0	0	0	0	0	0	0	0
0	0	0	1	0	0	0	1	0
0	0	0	0	0	0	0	0	0
0	0	0	1	0	0	0	1	0
0	0	0	1	0	0	0	1	0
0	0	0	1	1	0	0	0	0
0	0	0	0	0	0	0	0	0
0	0	0	0	0	0	0	0	0

0	0	0	0	0	0	0	0	0
0	0	0	0	0	0	0	0	0
0	0	0	0	0	0	0	0	0
1	0	0	1	1	0	0	1	0
0	0	0	1	0	1	0	0	0
0	0	0	0	0	0	0	0	0
0	0	0	0	0	0	0	0	0
0	0	0	1	0	0	0	1	0
0	0	0	0	0	0	0	0	0
0	0	0	0	0	0	0	0	0
0	0	0	0	0	0	0	0	0
1	0	0	0	0	0	0	0	0
0	0	0	0	0	0	0	0	0
0	0	0	1	0	0	0	1	0
0	0	0	1	1	0	0	0	0
0	0	0	0	0	0	0	0	0
0	0	0	1	1	1	0	0	0
0	0	0	0	0	0	0	0	0
0	0	0	0	0	0	0	0	0
0	0	0	1	0	1	0	0	0
0	0	0	1	1	0	0	0	0
0	0	0	1	0	0	0	1	0
0	0	0	0	0	0	0	0	0
0	0	0	1	1	1	0	0	0
0	0	0	0	0	0	0	0	0
0	0	0	1	0	1	0	1	0
0	0	0	1	0	0	0	1	0
0	0	0	1	1	0	0	0	0
0	0	0	1	1	1	0	0	0
0	0	0	1	0	0	0	1	0
0	0	0	0	0	0	0	0	0
0	0	0	1	0	1	0	0	0
0	0	0	1	0	0	0	0	0
0	0	0	1	0	0	0	1	0
0	0	0	1	0	0	0	1	0
0	0	0	0	0	0	0	0	0
0	0	0	1	0	0	0	1	0
0	0	0	0	0	0	0	0	0
0	0	0	0	0	0	0	0	0
1	0	0	1	1	0	0	0	0
0	0	0	0	0	0	0	0	0
0	0	0	1	1	0	0	0	0
0	0	0	1	0	1	0	0	0
0	0	0	0	0	0	0	0	0
0	0	1	0	0	0	0	0	0
0	0	0	1	1	0	0	0	0
0	0	0	1	0	0	0	1	0
0	0	0	1	0	1	0	0	0
0	0	0	1	1	0	0	0	0
0	0	0	1	0	0	0	1	0
0	0	0	1	0	0	0	1	0
0	0	0	1	1	0	0	1	0
0	0	0	1	0	1	0	0	0
0	0	0	0	0	0	0	0	0

0	0	0	0	0	0	0	0	0
0	0	0	1	0	0	0	1	0
0	0	0	1	1	0	0	0	0
0	0	0	1	0	0	0	1	0
0	0	0	1	1	1	0	1	0
0	0	0	1	1	1	0	0	0
0	0	0	1	1	0	0	0	0
1	0	0	0	0	0	0	0	0
0	0	0	1	1	0	0	0	0
0	0	0	0	0	0	0	0	0
0	0	0	0	0	0	0	0	0
0	0	0	0	0	0	0	0	0
0	0	0	0	0	0	0	0	0
1	0	0	0	0	0	0	0	0
0	0	0	1	1	0	0	0	0
0	0	0	1	1	1	0	0	0
0	0	0	1	1	0	0	0	0
0	0	0	1	1	1	1	0	0
0	0	0	0	0	0	0	0	0
1	0	0	1	1	0	0	0	0
0	0	0	1	0	1	0	1	0
0	0	0	1	1	0	0	0	0
0	0	0	0	0	0	0	0	0
0	0	0	0	0	0	0	0	0
0	0	0	0	0	0	0	0	0
0	0	0	1	0	0	0	1	0
0	0	0	0	0	0	0	0	0
0	0	0	1	1	0	0	0	0
0	0	0	0	0	0	0	0	0
0	0	0	0	0	0	0	0	0
0	0	0	0	0	0	0	0	0
0	1	0	0	0	0	0	0	0
0	0	0	1	1	0	0	0	0
0	0	0	1	1	0	0	0	0
0	0	0	0	0	0	0	0	0
0	0	0	1	1	1	0	0	0
0	0	0	0	0	0	0	0	0
0	1	0	0	0	0	0	0	0
0	0	0	0	0	0	0	0	0
0	0	0	0	0	0	0	0	0
0	0	0	1	0	1	0	0	0
0	0	0	0	0	0	0	0	0
0	0	0	0	0	0	0	0	0
0	0	0	1	1	0	0	1	0
0	0	0	0	0	0	0	0	0
0	0	0	1	0	0	0	1	0
0	0	0	1	1	0	0	0	0
0	0	0	0	0	0	0	0	0
0	0	0	0	0	0	0	0	0
1	0	0	0	0	0	0	0	0
0	0	0	1	1	0	0	1	0
0	0	0	1	1	0	0	1	0
0	0	0	0	0	0	0	0	0
0	0	0	0	0	0	0	0	0

0	0	0	1	1	0	0	0	0
0	0	0	0	0	0	0	0	1
1	0	0	1	1	0	0	0	0
0	0	0	0	0	0	0	0	0
0	0	0	0	0	0	0	0	0
0	0	0	0	0	0	0	0	0
0	0	0	1	0	1	0	1	0
0	0	0	1	1	0	0	0	0
0	0	0	0	0	0	0	0	0
0	0	0	0	0	0	0	0	0
0	0	0	0	0	0	0	0	0
0	0	0	1	1	0	0	0	0
1	0	0	0	0	0	0	0	0
0	0	0	1	1	0	0	0	0
0	0	0	0	0	0	0	0	0
0	0	0	0	0	0	0	0	0
0	0	0	0	0	0	0	0	0
0	0	0	0	0	0	0	0	0
0	0	0	0	0	0	0	0	0
0	0	0	0	0	0	0	0	0
0	0	0	0	0	0	0	0	0
0	0	0	1	0	0	0	1	1
0	0	0	0	0	0	0	0	0
0	0	1	0	0	0	0	0	0
0	0	0	0	0	0	0	0	0
0	0	1	0	0	0	0	0	0
0	0	0	1	1	0	0	0	0
0	0	0	1	1	0	0	1	0
0	0	0	0	0	0	0	0	0
0	0	0	1	0	0	0	1	0
1	0	0	0	0	0	0	0	0
0	0	0	0	0	0	0	0	0
0	0	0	1	1	0	0	0	0
0	0	0	0	0	0	0	0	0
0	0	0	0	0	0	0	0	0
0	0	1	1	0	0	0	1	0
0	0	0	0	0	0	0	0	0
0	0	0	0	0	0	0	0	0
0	0	0	1	0	0	0	1	0
0	0	0	0	0	0	0	0	0
0	0	0	0	0	0	0	0	0
0	0	0	0	0	0	0	0	0
0	0	0	0	0	0	0	0	0
0	0	0	1	0	0	0	1	0
0	0	0	1	1	0	0	0	0
0	0	0	0	0	0	0	0	0
0	0	0	0	0	0	0	0	0
0	0	0	1	0	1	0	0	0
0	0	0	1	0	0	0	1	0
0	0	0	0	0	0	0	0	0
1	0	0	0	0	0	0	0	0
0	0	0	1	1	0	0	0	0
1	0	0	0	0	0	0	0	0
0	0	0	1	1	0	0	1	0

1	0	0	1	0	0	1	0	0
1	0	0	1	1	0	1	0	0
1	0	0	0	0	0	0	0	0
0	0	0	0	0	0	0	0	0
0	0	0	0	0	0	0	0	0
1	0	0	0	0	0	0	0	0
0	0	0	1	0	0	0	1	0
0	0	0	0	0	0	0	0	0
0	0	0	0	0	0	0	0	0
0	0	0	1	0	0	1	0	0
0	0	0	0	0	0	0	0	0
0	0	1	0	0	0	0	0	0
0	0	0	0	0	0	0	0	0
0	0	0	0	0	0	0	0	0
0	0	0	1	1	0	0	0	0
0	0	0	0	0	0	0	0	0
0	0	1	0	0	0	0	0	0
0	0	0	1	0	0	1	1	0
0	0	0	1	0	1	0	0	0
0	0	0	0	0	0	0	0	0
0	0	0	1	1	0	0	0	0
0	0	0	0	0	0	0	0	0
0	0	0	1	0	1	1	0	0
0	0	1	0	0	0	0	0	0
0	0	0	0	0	0	0	0	0
0	0	0	0	0	0	0	0	0
1	0	0	1	1	0	1	0	0
0	0	0	1	0	0	0	1	0
1	0	0	1	0	0	0	1	0
0	0	0	1	1	0	0	0	0
1	0	0	0	0	0	0	0	0
0	0	0	0	0	0	0	0	0
0	0	0	0	0	0	0	0	0
0	1	0	1	0	0	0	1	0
0	0	0	1	1	0	0	0	0
0	0	0	0	0	0	0	0	0
0	0	0	0	0	0	0	0	0
0	0	0	0	0	0	0	0	0
0	0	0	1	0	0	0	1	0
0	0	0	0	0	0	0	0	0
0	0	0	1	0	0	0	1	0
0	0	0	0	0	0	0	0	0
0	0	0	0	0	0	0	0	0
0	0	0	0	0	0	0	0	0
0	0	0	0	0	0	0	0	0
0	0	0	0	0	0	0	0	0
0	0	0	0	0	0	0	0	0
0	0	0	1	0	0	0	1	0
0	0	0	1	0	0	0	1	0
0	0	0	0	0	0	0	0	0
0	0	0	1	1	0	0	0	0
0	0	0	0	0	0	0	0	0
1	0	0	0	0	0	0	0	0
1	0	0	0	0	0	0	0	0

1	0	0	1	0	1	0	0	0
0	0	0	0	0	0	0	0	0
0	0	0	1	0	1	0	0	0
0	0	0	0	0	0	0	0	0
0	0	0	1	0	0	0	1	0
0	0	0	0	0	0	0	0	0
0	0	0	1	0	0	0	1	0
0	0	0	0	0	0	0	0	0
0	0	0	0	0	0	0	0	0
0	0	0	1	0	0	1	0	0
0	0	0	0	0	0	0	0	0
0	0	0	0	0	0	0	0	0
0	0	0	0	0	0	0	0	0
0	0	0	0	0	0	0	0	0
0	0	0	1	1	0	0	0	0
0	0	0	0	0	0	0	0	0
0	0	0	1	1	0	0	0	0
0	0	0	0	0	0	0	0	0
0	0	0	0	0	0	0	0	0
1	0	0	1	1	0	0	0	0
1	0	0	0	0	0	0	0	0
0	0	0	1	1	0	0	0	0
0	0	0	0	0	0	0	0	0
0	0	0	0	0	0	0	0	0
0	0	0	1	1	1	0	0	0
0	0	0	0	0	0	0	0	0
0	0	0	1	1	1	0	1	0
0	0	0	0	0	0	0	0	0
0	0	0	1	0	1	0	1	0
0	0	0	1	0	1	0	1	0
0	0	0	0	0	0	0	0	0
0	0	1	1	1	1	0	0	0
0	0	0	0	0	0	0	0	0
0	0	0	1	0	0	0	1	0
0	0	0	1	0	0	0	1	0
1	0	0	0	0	0	0	0	0
0	0	0	0	0	0	0	0	0
0	0	0	0	0	0	0	0	0
1	0	0	0	0	0	0	0	0
0	0	0	0	0	0	0	0	0
0	0	0	1	0	1	0	0	0
0	0	0	0	0	0	0	0	0
0	0	0	1	0	1	0	1	0
0	0	0	1	1	0	0	0	0
1	0	0	1	1	1	0	1	0
0	0	0	1	1	1	0	0	0
0	0	0	1	1	0	0	0	0
0	0	0	0	0	0	0	0	0
1	0	0	1	0	1	0	0	0
0	0	0	0	0	0	0	0	0
0	0	0	0	0	0	0	0	0
0	0	0	0	0	0	0	0	0
1	0	0	1	0	0	0	1	0
1	0	0	0	0	0	0	0	0

0	0	0	0	0	0	0	0	0
0	0	0	0	0	0	0	0	0
0	1	1	0	0	0	0	0	0
0	0	0	1	1	0	0	0	0
0	0	0	1	1	0	0	0	0
1	0	0	1	0	1	0	1	0
0	0	0	1	1	0	0	0	0
0	0	0	0	0	0	0	0	0
0	0	0	0	0	0	0	0	0
0	0	0	0	0	0	0	0	0
0	0	0	0	0	0	0	0	0
0	0	0	1	1	0	0	0	0
0	0	0	1	0	1	0	0	0
0	0	0	0	0	0	0	0	0
0	0	0	0	0	0	0	0	0
0	0	0	0	0	0	0	0	0
0	0	0	0	0	0	0	0	0
0	0	0	0	0	0	0	0	0
0	0	0	1	1	0	0	0	0
0	0	0	0	0	0	0	0	0
0	0	0	1	1	0	0	1	0
0	0	0	1	0	0	0	1	0

阳性体征	腹部压痛	口腔溃疡、贫血貌		腹水	长期用药史	激素、非甾	抗结核药物	氨基水杨酸
1	1	0	0	0	0	0	0	0
1	0	1	0	0	0	0	0	0
1	1	0	0	0	0	0	0	0
0	0	0	0	0	1	0	0	1
1	1	0	0	0	0	0	0	0
1	1	0	1	0	0	0	0	0
1	1	0	0	0	0	0	0	0
1	1	0	0	0	0	0	0	0
1	1	0	0	0	0	0	0	0
1	1	0	0	0	0	0	0	0
1	0	1	0	0	0	0	0	0
1	1	0	0	0	0	0	0	0
1	0	1	0	0	0	0	0	0
1	1	0	0	0	0	0	0	0
1	1	0	0	0	0	0	0	0
1	1	1	0	0	0	0	0	0
1	0	1	0	0	0	0	0	0
1	0	0	1	0	0	0	0	0
1	1	1	0	0	0	0	0	0
1	1	1	0	0	0	0	0	0
0	0	0	0	0	0	0	0	0
1	1	1	0	0	0	0	0	0
1	1	0	0	0	0	0	0	0
1	1	0	0	0	0	0	0	0
1	1	0	0	0	0	0	0	0
1	1	0	0	0	0	0	0	0
1	1	0	0	0	0	0	0	0
1	1	0	0	0	0	0	0	0
1	1	0	0	0	0	0	0	0
1	1	0	0	0	0	0	0	0
1	1	0	0	0	0	0	0	0
1	1	0	0	0	0	0	0	0
0	0	0	0	0	0	0	0	0
1	1	0	0	0	0	0	0	0
1	1	1	0	0	0	0	0	0
1	1	0	0	0	0	0	0	0
0	0	0	0	0	0	1	0	0
0	0	0	0	0	0	0	0	0
1	1	0	0	0	0	0	0	0
1	1	1	0	0	0	0	0	0
1	1	0	1	0	0	0	0	0
1	1	0	0	0	0	0	0	0
1	1	0	0	0	0	0	0	0
1	1	0	0	0	0	0	0	0
1	1	0	0	0	0	0	0	0
1	1	0	0	0	0	0	0	0
1	1	1	0	0	0	0	0	0
0	0	0	0	0	0	0	0	0
1	1	0	0	0	0	0	0	0
1	1	0	0	0	0	0	0	0
1	1	0	0	0	0	0	0	0
1	1	0	0	0	0	0	0	0
1	1	1	0	0	0	0	0	0

1	1	0	0	0	0	0	0	0
1	1	0	0	0	0	0	0	0
1	1	0	0	0	0	0	0	0
1	1	1	0	1	0	0	0	0
1	1	0	0	0	0	0	0	0
0	0	0	0	0	0	0	0	0
1	1	0	0	0	0	0	0	0
1	1	0	0	0	0	0	0	0
1	1	0	0	0	0	0	0	0
1	1	0	0	0	0	0	0	0
1	1	1	1	0	0	0	0	0
0	0	0	0	0	0	0	0	0
1	1	0	0	0	0	0	0	0
1	1	0	0	0	0	0	0	0
1	1	0	0	0	0	0	0	0
1	1	0	0	0	0	0	0	0
1	0	0	1	0	0	0	0	0
0	0	0	0	0	0	0	0	0
1	0	1	0	0	0	0	0	0
0	0	0	0	0	0	0	0	0
1	1	0	0	0	0	0	0	0
1	1	0	0	0	0	0	0	0
0	0	0	0	0	0	0	0	0
1	1	0	0	0	0	0	0	0
1	1	1	0	0	0	0	0	0
1	1	0	0	0	0	0	0	0
1	1	0	0	0	0	0	0	0
0	0	0	0	0	0	0	0	0
1	1	0	1	0	0	0	0	0
1	1	0	0	0	0	0	0	0
1	1	0	1	0	0	0	0	0
1	1	0	0	0	0	0	0	0
1	0	1	0	0	0	0	0	0
1	1	0	0	0	0	0	0	0
0	0	0	0	0	0	0	0	0
1	1	0	0	0	0	0	0	0
1	0	1	0	1	0	0	0	0
1	1	0	0	0	0	0	0	0
1	1	0	0	0	0	0	0	0
1	1	0	0	0	0	0	0	0
1	0	1	0	0	0	0	0	0
1	1	0	0	0	0	0	0	0
1	1	0	0	0	0	0	0	0
1	1	0	0	0	0	0	0	0
1	1	0	0	0	0	0	0	0
1	1	0	0	0	0	0	0	0
0	0	0	0	0	0	0	0	0
0	0	0	0	0	0	0	0	0
0	0	0	0	0	0	0	0	0
0	0	0	0	0	0	0	0	0
1	1	0	0	0	0	0	0	0
1	1	0	0	0	0	0	0	0
0	0	0	0	0	0	0	0	0
1	0	0	1	0	0	0	0	0

0	0	0	0	0	0	0	0	0
0	0	0	0	0	0	0	0	0
0	0	0	0	0	0	0	0	0
1	1	0	0	0	0	0	0	0
1	1	0	0	0	0	0	0	0
1	1	0	0	0	0	0	0	0
1	1	0	0	0	0	0	0	0
1	1	0	0	0	0	0	0	0
1	0	0	1	0	0	0	0	0
0	0	0	0	0	0	0	0	0
0	0	0	0	0	0	0	0	0
1	1	1	0	0	0	0	0	0
1	1	1	0	0	0	0	0	0
1	1	0	0	0	0	0	0	0
1	0	0	1	0	0	0	0	0
1	1	0	0	0	0	0	0	0
1	1	0	0	0	0	0	0	0
1	1	0	0	0	0	0	0	0
1	1	0	0	0	0	0	0	0
1	1	0	0	0	0	0	0	0
1	1	0	0	0	0	0	0	0
1	0	0	1	0	0	0	0	0
0	0	0	0	0	0	0	0	0
1	1	0	0	0	0	0	0	0
1	0	1	0	0	0	0	0	0
0	0	0	0	0	0	0	0	0
1	1	0	0	0	0	0	0	0
1	1	0	0	0	0	0	0	0
0	0	0	0	0	0	0	0	0
1	1	0	0	0	0	0	0	0
1	1	0	0	0	0	0	0	0
1	1	0	0	0	0	0	0	0
1	1	0	0	0	0	0	0	0
0	0	0	0	0	0	0	0	0
1	1	0	0	0	0	0	0	0
1	1	0	0	0	0	0	0	0
1	1	0	1	0	0	0	0	0
1	1	0	0	0	0	0	0	0
1	1	0	0	0	0	0	0	0
1	1	0	0	0	0	0	0	0
1	0	0	1	0	0	0	0	0
1	1	0	0	0	0	0	0	0
1	1	0	0	0	0	0	0	0
1	0	0	1	0	0	0	0	0
1	1	0	0	0	0	0	0	0
1	0	1	0	0	0	0	0	0
1	0	1	0	0	0	0	0	0
1	1	0	0	0	0	0	0	0
1	1	0	0	0	0	0	0	0
1	1	0	0	0	0	0	0	0
1	1	0	0	0	0	0	0	0
1	1	0	0	0	0	0	0	0
0	0	0	0	0	0	0	0	0
0	0	0	0	0	0	0	0	0

0	0	0	0	0	0	0	0	0
1	1	0	0	0	0	0	0	0
1	0	1	1	0	0	1	0	0
1	1	0	0	0	0	0	0	0
0	0	0	0	0	0	0	0	0
1	1	0	0	0	0	0	0	0
1	1	0	0	0	0	0	0	0
1	1	0	0	0	0	0	0	0
1	0	0	0	1	0	0	0	0
1	1	0	0	0	0	0	0	0
1	1	0	0	0	0	0	0	0
1	1	0	0	0	0	0	0	0
0	0	0	0	0	0	0	0	0
1	0	1	1	0	0	0	0	0
1	1	1	0	0	0	0	0	0
1	1	0	0	0	0	0	0	0
0	0	0	0	0	0	0	0	0
1	1	0	0	0	0	0	0	0
1	1	0	0	0	0	0	0	0
0	0	0	0	0	0	0	0	0
0	0	0	0	0	0	0	0	0
1	0	1	0	0	0	0	0	0
1	1	0	0	0	0	0	0	0
0	0	0	0	0	0	0	0	0
1	1	0	0	0	0	0	0	0
1	1	0	0	0	0	0	0	0
1	1	0	0	0	0	0	0	0
1	1	0	0	0	0	0	0	0
1	1	0	0	0	0	0	0	0
1	1	0	1	0	0	0	0	0
1	1	0	0	0	0	0	0	0
1	0	0	1	0	0	0	0	0
1	1	0	0	0	0	0	0	0
1	0	0	1	0	0	0	0	0
1	1	0	0	0	0	0	0	0
1	1	1	0	0	0	0	0	0
0	0	0	0	0	0	0	0	0
1	1	0	0	0	0	0	0	0
1	1	0	0	0	0	0	0	0
1	1	0	0	0	0	0	0	0
0	0	0	0	0	0	0	0	0
0	0	0	0	0	0	0	0	0
1	1	0	0	0	0	0	0	0
1	1	1	0	0	0	0	0	0
1	1	0	0	0	0	0	0	0
1	1	0	0	0	0	0	0	0
1	0	1	0	0	0	0	0	0
0	0	0	0	0	0	0	0	0
0	0	0	0	0	0	0	0	0
1	1	0	0	0	0	0	0	0
0	0	0	0	0	0	0	0	0
0	0	0	0	0	0	0	0	0
0	0	0	0	0	0	0	0	0
0	0	0	0	0	0	0	0	0

0	0	0	0	0	0	0	0	0
0	0	0	0	0	0	0	0	0
1	1	0	0	0	0	0	0	0
1	1	0	0	0	0	0	0	0
1	1	0	0	0	0	0	0	0
1	1	0	0	0	0	0	0	0
1	1	0	0	0	0	0	0	0
1	1	0	0	0	0	0	0	0
0	0	0	0	0	0	0	0	0
1	1	1	0	0	0	0	0	0
1	1	0	0	0	0	0	0	0
1	1	0	0	0	0	0	0	0
1	1	0	0	0	0	0	0	0
1	1	0	0	0	0	0	0	0
0	0	0	0	0	0	0	0	0
0	0	0	0	0	0	0	0	0
0	0	0	0	0	0	0	0	0
0	0	0	0	0	0	0	0	0
0	0	0	0	0	0	0	0	0
1	0	0	1	0	0	0	0	0
1	0	0	0	1	0	0	0	0
1	1	0	0	0	0	0	0	0
1	1	0	0	0	0	0	0	0
1	1	0	0	0	0	0	0	0
1	1	0	0	0	0	0	0	0
1	1	0	0	0	0	0	0	0
0	0	0	0	0	0	0	0	0
1	1	0	0	0	0	0	0	0
0	0	0	0	0	0	0	0	0
1	1	0	1	0	0	0	0	0
1	1	0	0	0	0	0	0	0
0	0	0	0	0	0	0	0	0
1	0	1	0	0	0	0	0	0
0	0	0	0	0	0	0	0	0
1	0	0	1	0	0	0	0	0
1	0	1	0	0	0	0	0	0
1	1	0	0	0	0	0	0	0
0	0	0	0	0	0	0	0	0
1	0	0	0	1	0	0	0	0
1	0	0	1	0	0	0	0	0
1	1	0	0	0	0	0	0	0
1	1	0	0	0	0	0	0	0
1	0	1	0	0	0	0	0	0
1	0	1	0	0	0	0	0	0
0	0	0	0	0	0	0	0	0
0	0	0	0	0	0	0	0	0
1	1	0	0	0	0	0	0	0
0	0	0	0	0	0	0	0	0
1	1	0	0	0	0	0	0	0
1	1	0	0	0	0	0	0	0
1	0	1	0	0	0	0	0	0
0	0	0	0	0	0	0	0	0
1	1	0	0	0	0	0	0	0
0	0	0	0	0	0	0	0	0

0	0	0	0	0	0	0	0	0
1	1	0	0	0	0	0	0	0
0	0	0	0	0	0	0	0	0
0	0	0	0	0	0	0	0	0
0	0	0	0	0	0	0	0	0
0	0	0	0	0	0	0	0	0
1	1	0	0	0	0	0	0	0
0	0	0	0	0	0	0	0	0
1	1	0	0	0	0	0	0	0
1	1	0	0	0	0	0	0	0
0	0	0	0	0	0	0	0	0
1	1	0	0	0	0	0	0	0
1	1	0	0	0	0	0	0	0
0	0	0	0	0	0	0	0	0
0	0	0	0	0	0	0	0	0
1	1	0	0	0	0	0	0	0
0	0	0	0	0	0	0	0	0
0	0	0	0	0	0	0	0	0
0	0	0	0	0	0	0	0	0
0	0	0	0	0	0	0	0	0
0	0	0	0	0	0	0	0	0
1	1	0	0	0	0	0	0	0
0	0	0	0	0	0	0	0	0
1	1	0	0	0	0	0	0	0
1	1	0	0	0	0	0	0	0
0	0	0	0	0	0	0	0	0
0	0	0	0	0	0	0	0	0
1	1	0	0	0	0	0	0	0
1	1	1	0	0	0	0	0	0
1	1	0	0	0	0	0	0	0
1	1	0	0	0	0	0	0	0
1	1	0	0	0	0	0	0	0
1	1	0	0	0	0	0	0	0
1	1	0	0	0	0	0	0	0
1	0	0	1	0	0	0	0	0
0	0	0	0	0	0	0	0	0
1	1	0	0	0	0	0	0	0
1	1	0	0	0	0	0	0	0
1	1	0	0	0	0	0	0	0
1	1	0	0	0	0	0	0	0
1	1	0	0	0	0	0	0	0
1	1	0	0	0	0	0	0	0
0	0	0	0	0	0	0	0	0
0	0	0	0	0	0	0	0	0
0	0	0	0	0	0	0	0	0
1	1	0	0	0	0	0	0	0
1	1	0	0	0	0	0	0	0
1	1	0	0	0	0	0	0	0
1	1	0	0	0	0	0	0	0
1	1	0	0	0	0	0	0	0
1	1	0	0	0	0	0	0	0
0	0	0	0	0	0	0	0	0
1	1	0	0	0	0	0	0	0
1	1	0	0	0	0	0	0	0

1	1	0	0	0	0	0	0	0
1	1	0	0	0	0	0	0	0
1	1	0	0	0	0	0	0	0
1	1	0	0	0	0	0	0	0
1	0	1	0	0	0	0	0	0
1	1	0	0	0	0	0	0	0
1	1	0	0	0	0	0	0	0
1	1	0	0	0	0	0	0	0
1	1	0	0	0	0	0	0	0
0	0	0	0	0	0	0	0	0
0	0	0	0	0	0	0	0	0
1	1	0	0	0	0	0	0	0
1	1	0	0	0	0	0	0	0
1	1	0	0	0	0	0	0	0
1	1	0	0	0	0	0	0	0
0	0	0	0	0	0	0	0	0
1	1	0	0	0	0	0	0	0
1	1	0	0	0	0	0	0	0
1	1	0	0	0	0	0	0	0
0	0	0	0	0	0	0	0	0
1	1	1	0	0	0	0	0	0
1	1	0	0	0	0	0	0	0
0	0	0	0	0	0	0	0	0
0	0	0	0	0	0	0	0	0
0	0	0	0	0	0	0	0	0
1	0	1	0	0	0	0	0	0
1	1	0	0	0	0	0	0	0
1	1	0	0	0	0	0	0	0
1	1	0	0	0	0	1	0	0
1	1	0	0	0	0	0	0	0
1	1	0	0	0	0	0	0	0
1	1	0	0	0	0	0	0	0
1	1	0	0	0	0	0	0	0
1	1	0	0	0	0	0	0	0
0	0	0	0	0	0	0	0	0
0	0	0	0	0	0	0	0	0
1	0	0	1	0	0	0	0	0
1	0	0	1	0	0	0	0	0
1	1	0	0	0	0	0	0	0
1	1	0	0	0	0	0	0	0
0	0	0	0	0	0	0	0	0
1	1	0	0	0	0	0	0	0
1	1	0	0	0	0	0	0	0
0	0	0	0	0	0	0	0	0
1	0	0	1	0	0	0	0	0
0	0	0	0	0	0	0	0	0
1	1	0	0	0	0	0	0	0
1	1	0	0	0	0	0	0	0
1	0	0	1	0	0	0	0	0
1	1	0	0	0	0	0	0	0
1	1	0	0	0	0	0	0	0
1	1	0	0	0	0	0	0	0
1	1	0	0	0	0	0	0	0
1	1	0	0	0	0	0	0	0

1	1	0	0	0	0	0	0	0
1	1	0	0	0	0	0	0	0
0	0	0	0	0	0	0	0	0
0	0	0	0	0	0	0	0	0
1	0	0	1	0	0	0	0	0
1	1	0	0	0	0	0	0	0
1	1	0	0	0	0	0	0	0
1	1	0	0	0	0	0	0	0
1	1	0	0	0	0	0	0	0
1	1	0	0	0	0	0	0	0
1	1	0	0	0	0	0	0	0
1	1	0	0	0	0	0	0	0
1	1	0	0	0	0	0	0	0
1	1	0	0	0	0	0	0	0
1	1	0	0	0	0	0	0	0
0	0	0	0	0	0	0	0	0
1	1	0	0	0	0	0	0	0
0	0	0	0	0	0	0	0	0
0	0	0	0	0	0	0	0	0
1	1	0	0	0	0	0	0	0
1	1	0	0	0	0	0	0	0
1	1	0	0	0	0	0	0	0
1	1	0	0	0	0	0	0	0
1	1	0	0	0	0	0	0	0
0	0	0	0	0	0	0	0	0
1	1	0	0	0	0	0	0	0
1	1	0	0	0	0	0	0	0
1	1	0	0	0	0	0	0	0
1	0	0	1	0	0	0	0	0
1	1	0	0	0	0	0	0	0
1	1	0	0	0	0	0	0	0
1	1	0	0	0	0	0	0	0
1	1	0	0	0	0	0	0	0
1	1	0	0	0	0	0	0	0
1	1	0	0	0	0	0	0	0
0	0	0	0	0	0	0	0	0
1	0	0	1	0	0	0	0	0
1	0	0	1	0	0	0	0	0
0	0	0	0	0	0	0	0	0
1	1	0	0	0	0	0	0	0
1	1	0	0	0	0	0	0	0
0	0	0	0	0	0	0	0	0
1	1	0	0	0	0	0	0	0
1	1	0	0	0	0	0	0	0
1	1	0	1	0	0	0	0	0
1	0	0	1	0	0	0	0	0
0	0	0	0	0	0	0	0	0
0	0	0	0	0	0	0	0	0
1	1	0	0	0	0	0	0	0
0	0	0	0	0	0	0	0	0
1	0	0	1	0	0	0	0	0
0	0	0	0	0	0	0	0	0
1	1	0	0	0	0	0	0	0
1	1	0	0	0	0	0	0	0

1	1	0	0	0	0	0	0	0
0	0	0	0	0	0	0	0	0
1	1	0	0	0	0	0	0	0
1	1	0	0	0	0	0	0	0
0	0	0	0	0	0	0	0	0
1	1	0	0	0	0	0	0	0
1	1	0	0	0	0	0	0	0
1	1	0	0	0	0	0	0	0
1	1	0	0	0	0	0	0	0
1	1	0	0	0	0	0	0	0
1	1	0	0	0	0	0	0	0
1	1	0	0	0	0	0	0	0
1	1	0	0	0	0	0	0	0
1	1	0	0	0	0	0	0	0
1	1	0	0	0	0	0	0	0
1	1	1	0	0	0	0	0	0
1	1	1	0	0	0	0	0	0
1	1	0	0	0	0	0	0	0
1	1	0	0	0	0	0	0	0
1	1	0	0	0	0	0	0	0
0	0	0	0	0	0	0	0	0
1	1	0	0	0	0	0	0	0
1	1	0	0	0	0	0	0	0
1	1	0	0	0	0	0	0	0
1	1	0	0	0	0	0	0	0
1	1	0	0	0	0	0	0	0
1	0	0	1	0	0	0	0	0
1	1	0	0	0	0	0	0	0
1	1	0	0	0	0	0	0	0
1	1	0	0	0	0	0	0	0
1	1	0	0	0	0	0	0	0
1	1	0	0	0	0	0	0	0
1	1	0	0	0	0	0	0	0
1	1	0	0	0	0	0	0	0
1	1	0	0	0	0	0	0	0
1	1	0	0	0	0	0	0	0
1	1	0	0	0	0	0	0	0
1	1	0	0	0	0	0	0	0
1	1	0	0	0	0	0	0	0
1	1	0	0	0	0	0	0	0
1	1	1	0	0	0	0	0	0
1	1	0	0	0	1	0	1	0
1	1	0	0	0	0	0	0	0
1	1	0	0	0	1	0	0	0
1	1	0	0	0	0	0	0	0
1	1	0	0	0	0	0	0	0
1	1	0	0	0	0	0	0	0
1	1	0	0	0	0	0	0	0
1	1	0	0	0	0	0	0	0
1	1	0	0	0	0	0	0	0
1	1	1	0	0	0	0	0	0
1	1	0	0	0	0	0	0	0
1	1	0	0	0	0	0	0	0
1	1	0	0	0	0	0	0	0

1	1	0	0	0	0	0	0	0
1	1	0	0	0	0	0	0	0
1	1	0	0	0	0	0	0	0
1	1	0	0	0	0	0	0	0
1	1	0	0	0	1	0	0	1
1	1	0	0	0	0	0	0	0
1	0	0	1	0	0	0	0	0
0	0	0	0	0	0	0	0	0
0	0	0	0	0	0	0	0	0
1	1	0	1	0	0	0	0	0
1	1	0	0	0	0	0	0	0
1	1	0	0	0	0	0	0	0
1	0	1	0	0	0	0	0	0
1	1	0	0	0	0	0	0	0
1	1	0	0	0	0	0	0	0
1	1	0	0	0	0	0	0	0
1	1	0	1	0	0	0	0	0
1	0	1	0	0	0	0	0	0
1	1	0	0	0	0	0	0	0
0	0	0	0	0	0	0	0	0
1	1	0	0	0	0	0	0	0
1	1	0	0	0	0	0	0	0
1	1	0	0	0	0	0	0	0
1	1	0	0	0	0	0	0	0
1	1	0	0	0	1	0	0	0
1	1	0	0	0	0	0	0	0
1	1	0	0	0	0	0	0	0
1	1	0	1	0	0	0	0	0
1	1	0	0	0	0	0	0	0
1	0	0	1	0	0	0	0	0
1	1	0	0	0	1	0	0	1
1	1	0	0	0	0	0	0	0
1	0	0	1	0	0	0	0	0
0	0	0	0	0	0	0	0	0
1	1	1	0	0	0	0	0	0
1	1	0	0	0	0	0	0	0
1	1	0	0	0	0	0	0	0
1	1	0	0	0	0	0	0	0
1	1	0	0	0	0	0	0	0
1	1	0	0	0	0	0	0	0
1	1	0	0	0	0	0	0	0
0	0	0	0	0	0	0	0	0
0	0	0	0	0	1	0	0	1
1	1	0	0	0	0	0	0	0
1	1	0	0	0	0	0	0	0
1	1	0	0	0	0	0	0	0
1	1	0	0	0	0	0	0	0
1	1	0	0	0	0	0	0	0
1	1	0	0	0	0	0	0	0
1	1	0	0	0	0	0	0	0
0	0	0	0	0	0	0	0	0
1	1	0	0	0	0	0	0	0
1	1	0	1	0	0	0	0	0
1	1	0	0	0	0	0	0	0

1	1	0	0	0	0	0	0	0
1	1	0	0	0	0	0	0	0
1	1	0	0	0	0	0	0	0
1	1	0	0	0	0	0	0	0
1	1	0	0	0	0	0	0	0
1	1	0	0	0	0	0	0	0
1	0	1	0	0	0	0	0	0
1	1	0	0	0	0	0	0	0
1	1	0	0	0	0	0	0	0
1	1	0	0	0	0	0	0	0
1	1	0	0	0	0	0	0	0
1	1	0	0	0	0	0	0	0
0	0	0	0	0	0	0	0	0
1	1	0	0	0	0	0	0	0
1	1	1	0	0	0	0	0	0
1	1	0	0	0	0	0	0	0
1	1	1	0	0	0	0	0	0
1	1	0	0	0	0	0	0	0
1	1	0	0	0	0	0	0	0
1	1	0	0	0	0	0	0	0
1	1	0	0	0	0	0	0	0
1	1	0	0	0	0	0	0	0
1	1	0	0	0	0	0	0	0
0	0	0	0	0	0	0	0	0
1	1	0	0	0	0	0	0	0
1	1	0	0	0	0	0	0	0
1	1	0	0	0	0	0	0	0
1	1	0	0	0	0	0	0	0
1	1	0	0	0	0	0	0	0
1	1	0	0	0	0	0	0	0
1	1	0	0	0	0	0	0	0
0	0	0	0	0	0	0	0	0
0	0	0	0	0	0	0	0	0
1	1	0	0	0	0	0	0	0
1	1	0	0	0	0	0	0	0
0	0	0	0	0	0	0	0	0
1	1	0	0	0	0	0	0	0
1	1	0	0	0	0	0	0	0
0	0	0	0	0	0	0	0	0
1	1	0	0	0	0	0	0	0
1	1	0	0	0	0	0	0	0
1	1	0	0	0	0	0	0	0
1	1	0	0	0	0	0	0	0
0	0	0	0	0	0	0	0	0
0	0	0	0	0	0	0	0	0
1	1	0	1	0	0	0	0	0
1	1	0	0	0	0	0	0	0
1	1	0	0	0	0	0	0	0
1	1	0	0	0	0	0	0	0
1	1	0	0	0	0	0	0	0
1	1	0	0	0	0	0	0	0
1	1	0	0	0	0	0	0	0
1	1	0	1	0	0	0	0	0
1	1	0	0	0	0	0	0	0

1	0	1	0	0	0	0	0	0
1	1	0	0	0	0	0	0	0
0	0	0	0	0	0	0	0	0
1	1	0	0	0	0	0	0	0
1	1	0	0	0	0	0	0	0
1	1	0	0	0	0	0	0	0
1	1	0	0	0	0	0	0	0
1	1	0	0	0	0	0	0	0
1	0	0	0	0	0	0	0	0
1	1	0	0	0	0	0	0	0
1	1	0	0	0	0	0	0	0
1	1	0	0	0	0	0	0	0
1	1	0	0	0	0	0	0	0
1	1	0	0	0	0	0	0	0
1	1	0	0	0	0	0	0	0
0	0	0	0	0	0	0	0	0
1	0	0	1	0	0	0	0	0
1	0	0	0	0	0	0	0	0
1	1	0	0	0	0	0	0	0
1	1	0	0	0	0	0	0	0
0	0	0	0	0	0	0	0	0
1	1	0	0	0	0	0	0	0
1	1	0	0	0	0	0	0	0
1	1	0	0	0	0	0	0	0
1	0	0	0	0	0	0	0	0
1	1	0	0	0	0	0	0	0
1	1	0	0	0	0	0	0	0
1	1	0	0	0	1	1	0	0
1	0	0	0	0	0	0	0	0
1	1	0	0	0	0	0	0	0
1	0	0	0	0	0	0	0	0
1	1	0	0	0	0	0	0	0
1	1	0	0	0	0	0	0	0
1	1	0	0	0	0	0	0	0
1	1	0	0	0	0	0	0	0
1	1	0	0	0	0	0	0	0
1	1	0	0	0	0	0	0	0
1	1	0	0	0	0	0	0	0
1	1	0	0	0	0	0	0	0
1	1	0	1	0	0	0	0	0
1	1	0	0	0	0	0	0	0
1	1	0	0	0	0	0	0	0
1	1	0	0	0	0	0	0	0
1	0	0	0	0	0	0	0	0
1	1	0	0	0	0	0	0	0
1	1	0	0	0	0	0	0	0
0	0	0	0	0	0	0	0	0
1	1	0	0	0	0	0	0	0
1	1	0	0	0	0	0	0	0
1	1	0	0	0	0	0	0	0
0	0	0	0	0	0	0	0	0
1	1	0	0	0	0	0	0	0
1	1	0	0	0	0	0	0	0
1	1	0	0	0	0	0	0	0

1	1	0	0	0	0	0	0	0
1	0	0	1	0	0	0	0	0
1	1	0	0	0	0	0	0	0
1	1	0	0	0	0	0	0	0
1	1	0	0	0	0	0	0	0
0	0	0	0	0	0	0	0	0
1	1	0	0	0	0	0	0	0
0	0	0	0	0	1	1	0	1
1	1	0	0	0	1	0	0	0
1	1	0	0	0	0	0	0	0
1	1	0	0	0	0	0	0	0
0	0	0	0	0	0	0	0	0
1	1	0	0	0	0	0	0	0
1	1	0	0	0	0	0	0	0
0	0	0	0	0	0	0	0	0
1	1	0	0	0	0	0	0	0
1	1	0	0	0	0	0	0	0
1	1	0	0	0	0	0	0	0
1	1	0	0	0	0	0	0	0
1	1	0	0	0	0	0	0	0
1	1	0	0	0	0	0	0	0
0	0	0	0	0	0	0	0	0
1	1	0	0	0	0	0	0	0
1	1	0	0	0	0	0	0	0
1	1	0	0	0	0	0	0	0
1	1	0	0	0	0	0	0	0
1	1	0	0	0	0	0	0	0
1	1	0	0	0	0	0	0	0
1	1	0	0	0	0	0	0	0
1	0	0	0	0	0	0	0	0
1	1	0	0	0	0	0	0	0
1	0	0	1	0	0	0	0	0
1	1	0	0	0	1	1	0	0
1	0	1	0	0	0	0	0	0
1	1	0	0	0	0	0	0	0
1	1	0	0	0	0	0	0	0
1	1	0	1	0	0	0	0	0
1	1	0	0	0	0	0	0	0
1	1	0	0	0	0	0	0	0
1	1	0	0	0	0	0	0	0
0	0	0	0	0	0	0	0	0
1	1	0	0	0	0	0	0	0
0	0	0	0	0	0	0	0	0
1	1	0	0	0	0	0	0	0
0	0	0	0	0	0	0	0	0
1	1	0	0	0	0	0	0	0
1	1	0	0	0	0	0	0	0
1	0	0	0	0	0	0	0	0
1	1	0	0	0	0	0	0	0
0	0	0	0	0	0	0	0	0
1	1	0	0	0	0	0	0	0
1	0	0	0	0	0	0	0	0
1	1	0	0	0	0	0	0	0
1	1	0	0	0	0	0	0	0

0	0	0	0	0	1	0	0	0
0	0	0	0	0	0	0	0	0
1	0	0	0	0	0	0	0	0
1	1	0	0	0	0	0	0	0
0	0	0	0	0	0	0	0	0
1	1	0	0	0	0	0	0	0
1	0	0	0	0	1	1	0	0
0	0	0	0	0	0	0	0	0
1	1	0	0	0	0	0	0	0
0	0	0	0	0	0	0	0	0
1	0	0	0	0	0	0	0	0
0	0	0	0	0	0	0	0	0
1	1	0	0	0	0	0	0	0
1	0	0	1	0	0	0	0	0
1	1	0	0	0	0	0	0	0
1	1	0	0	0	0	0	0	0
1	1	0	0	0	0	0	0	0
1	0	0	0	0	0	0	0	0
1	1	0	0	0	0	0	0	0
1	1	0	0	0	0	0	0	0
1	1	0	0	0	1	1	0	0
1	1	0	0	0	0	0	0	0
1	1	0	0	0	0	0	0	0
1	1	0	0	0	0	0	0	0
1	1	0	0	0	0	0	0	0
1	1	0	0	0	0	0	0	0
1	1	0	0	0	0	0	0	0
1	1	0	0	0	0	0	0	0
1	1	0	0	0	0	0	0	0
1	1	0	0	0	0	0	0	0
1	1	0	0	0	0	0	0	0
1	1	0	0	0	0	0	0	0
1	1	0	0	0	0	0	0	0
1	1	0	0	1	1	0	1	0
0	0	0	0	0	0	0	0	0
1	1	0	0	0	0	0	0	0
1	1	0	0	0	0	0	0	0
1	1	0	0	0	0	0	0	0
1	1	0	0	0	0	0	0	0
1	1	0	0	0	0	0	0	0
1	1	0	0	0	0	0	0	0
1	1	0	0	0	0	0	0	0
1	1	0	0	0	0	0	0	0
1	0	0	0	0	0	0	0	0
1	1	0	0	1	1	0	1	0
0	0	0	0	0	0	0	0	0
1	1	0	0	0	0	0	0	0
1	1	0	0	0	0	0	0	0
1	1	0	0	0	0	0	0	0
1	1	0	0	0	0	0	0	0
1	0	0	0	0	0	0	0	0
1	1	0	0	0	0	0	0	0
1	1	0	1	0	0	0	0	0
1	1	0	0	0	1	1	0	1

1	1	0	0	0	0	0	0	0
1	1	0	0	0	0	0	0	0
1	0	0	0	0	0	0	0	0
1	1	0	0	0	0	0	0	0
1	1	0	0	0	0	0	0	0
1	0	0	0	0	0	0	0	0
1	1	0	0	0	0	0	0	0
0	0	0	0	0	0	0	0	0
1	0	0	0	0	1	1	0	0
1	1	0	0	0	0	0	0	0
1	1	0	0	0	0	0	0	0
1	1	0	0	0	0	0	0	0
1	1	0	0	0	0	0	0	0
0	0	0	0	0	0	0	0	0
1	1	0	1	0	0	0	0	0
1	0	0	0	0	0	0	0	0
1	1	0	0	0	0	0	0	0
1	1	0	0	0	0	0	0	0
1	0	0	0	0	0	0	0	0
1	0	0	0	0	0	0	0	0
0	0	0	0	0	0	0	0	0
1	1	0	0	0	0	0	0	0
1	1	0	0	0	0	0	0	0
1	1	0	0	0	0	0	0	0
1	1	0	1	0	0	0	0	0
1	1	0	0	0	0	0	0	0
1	1	0	0	0	0	0	0	0
0	0	0	0	0	0	0	0	0
1	1	0	0	0	0	0	0	0
1	1	0	0	0	0	0	0	0
1	0	0	0	0	0	0	0	0
1	1	0	0	0	0	0	0	0
1	1	0	0	0	0	0	0	0
0	0	0	0	0	0	0	0	0
1	1	0	0	0	0	0	0	0
0	0	0	0	0	0	0	0	0
1	1	0	0	0	0	0	0	0
1	1	0	0	0	0	0	0	0
1	1	0	0	0	0	0	0	0
1	1	0	0	0	0	0	0	0
1	1	0	0	0	1	0	0	0
1	1	0	0	0	0	0	0	0
1	1	0	0	0	0	0	0	0
1	1	0	0	0	0	0	0	0
1	1	0	0	0	0	0	0	0
1	0	0	0	0	0	0	0	0
1	1	0	0	0	0	0	0	0
1	1	0	0	0	0	0	0	0
1	1	0	0	0	0	0	0	0
1	1	0	0	0	0	0	0	0
0	0	0	0	0	0	0	0	0
1	1	0	0	0	0	0	0	0
1	1	0	0	0	0	0	0	0
1	1	0	0	0	0	0	0	0

1	1	0	0	0	0	0	0	0
1	0	0	0	0	0	0	0	0
1	1	0	0	0	0	0	0	0
1	1	0	0	0	0	0	0	0
1	0	0	0	0	0	0	0	0
1	1	0	0	0	0	0	0	0
1	1	0	0	0	0	0	0	0
0	0	0	0	0	0	0	0	0
1	1	0	0	0	0	0	0	0
1	1	0	0	0	0	0	0	0
1	1	0	0	0	0	0	0	0
1	1	0	0	0	0	0	0	0
1	1	0	0	0	0	0	0	0
1	0	0	0	0	0	0	0	0
1	1	0	0	0	0	0	0	0
1	0	0	0	0	0	0	0	0
1	1	0	0	0	0	0	0	0
1	0	0	1	0	0	0	0	0
1	1	0	0	0	0	0	0	0
1	1	0	0	0	0	0	0	0
1	0	0	0	0	0	0	0	0
1	1	0	0	0	0	0	0	0
1	1	0	0	0	0	0	0	0
1	1	0	0	0	0	0	0	0
0	0	0	0	0	0	0	0	0
1	1	0	0	0	0	0	0	0
1	0	0	1	0	0	0	0	0
1	1	0	0	0	0	0	0	0
1	1	0	0	0	0	0	0	0
1	1	0	0	0	0	0	0	0
1	1	0	0	0	0	0	0	0
1	1	0	0	0	0	0	0	0
1	1	0	0	0	0	0	0	0
1	1	0	0	0	0	0	0	0
1	1	0	0	0	0	0	0	0
1	1	0	0	0	0	0	0	0
0	0	0	0	0	0	0	0	0
1	1	0	0	0	0	0	0	0
1	1	0	0	0	0	0	0	0
1	1	0	0	0	0	0	0	0
1	1	0	0	0	0	0	0	0
1	0	0	1	0	0	0	0	0
0	0	0	0	0	0	0	0	0
1	1	0	0	0	0	0	0	0
1	1	0	0	0	0	0	0	0
1	1	0	0	0	0	0	0	0
1	0	0	0	0	0	0	0	0
1	1	0	0	0	0	0	0	0
1	1	0	0	0	0	0	0	0
1	1	0	0	0	1	1	0	0
1	1	0	0	0	0	0	0	0
1	1	0	0	0	0	0	0	0
0	0	0	0	0	0	0	0	0
1	1	0	0	0	0	0	0	0

0	0	0	0	0	1	0	0	0
1	1	0	0	0	0	0	0	0
1	1	0	0	0	0	0	0	0
1	1	0	0	1	0	0	0	0
1	1	0	0	0	0	0	0	0
0	0	0	0	0	0	0	0	0
1	1	0	0	0	0	0	0	0
1	1	0	0	0	0	0	0	0
1	1	0	0	0	1	0	0	1
1	1	0	0	0	0	0	0	0
1	1	0	0	0	0	0	0	0
1	1	0	0	0	0	0	0	0
1	1	0	0	0	0	0	0	0
1	1	0	0	0	0	0	0	0
1	1	0	0	0	0	0	0	0
1	0	0	1	0	0	0	0	0
1	1	0	0	0	0	0	0	0
1	1	0	0	0	0	0	0	0
1	1	0	0	0	1	1	0	0
1	1	0	0	0	0	0	0	0
1	1	0	1	0	0	0	0	0
1	1	0	0	0	0	0	0	0
1	1	0	0	0	0	0	0	0
1	1	0	0	0	0	0	0	0
1	1	0	0	0	1	0	0	0
1	1	0	0	0	0	0	0	0
1	0	0	1	0	0	0	0	0
1	1	0	0	0	0	0	0	0
1	1	0	0	0	0	0	0	0
1	1	0	0	0	0	0	0	0
1	1	0	0	0	0	0	0	0
1	1	0	0	0	0	0	0	0
1	1	0	0	0	0	0	0	0
1	1	0	0	0	0	0	0	0
1	1	0	0	0	0	0	0	0
1	0	0	0	0	0	0	0	0
1	1	0	0	0	0	0	0	0
1	0	0	0	0	1	1	0	0
1	1	0	0	0	0	0	0	0
1	1	0	0	0	0	0	0	0
1	1	0	0	0	0	0	0	0
1	1	0	0	0	0	0	0	0
1	1	0	0	0	0	0	0	0
1	1	0	0	0	0	0	0	0
1	1	0	0	0	0	0	0	0
1	1	0	0	0	0	0	0	0
1	1	0	0	0	0	0	0	0
1	1	0	0	0	0	0	0	0
1	1	0	0	0	0	0	0	0
1	1	1	1	0	0	0	0	0
1	1	0	0	0	0	0	0	0
1	1	0	0	0	0	0	0	0
1	1	0	0	0	0	0	0	0
1	1	0	0	0	0	0	0	0

1	1	0	0	0	0	0	0	0
1	1	0	0	0	0	0	0	0
1	1	0	0	0	0	0	0	0
1	1	0	0	0	0	0	0	0
1	1	0	0	0	0	0	0	0
1	1	0	0	0	0	0	0	0
1	1	0	0	0	0	0	0	0
1	1	0	0	0	0	0	0	0
1	1	0	0	0	0	0	0	0
0	0	0	0	0	0	0	0	0
0	0	0	0	0	0	0	0	0
1	1	0	0	0	0	0	0	0
1	1	0	0	0	0	0	0	0
1	1	0	0	0	0	0	0	0
1	1	0	0	0	0	0	0	0
1	1	0	0	0	0	0	0	0
0	0	0	0	0	0	0	0	0
0	0	0	0	0	0	0	0	0
1	1	0	0	0	0	0	0	0
1	1	0	0	0	0	0	0	0
1	1	0	0	0	0	0	0	0
1	1	0	0	0	0	0	0	0
1	1	0	0	0	0	0	0	0
1	1	0	0	0	0	0	0	0
1	1	0	0	0	0	0	0	0
0	0	0	0	0	0	0	0	0
1	1	0	1	0	0	0	0	0
1	0	0	1	0	0	0	0	0
1	1	0	0	0	0	0	0	0
1	1	0	0	0	0	0	0	0
1	1	0	0	0	0	0	0	0
1	1	0	0	0	0	0	0	0
1	1	0	0	0	0	0	0	0
0	0	0	0	0	0	0	0	0
1	1	0	0	0	0	0	0	0
1	0	0	0	0	0	0	0	0
1	1	0	0	0	0	0	0	0
1	0	0	0	0	0	0	0	0
1	0	1	0	0	0	0	0	0
1	1	0	0	0	0	0	0	0
1	1	0	0	0	0	0	0	0
1	1	0	0	0	0	0	0	0
0	0	0	0	0	0	0	0	0
0	0	0	0	0	0	0	0	0
0	0	0	0	0	0	0	0	0
1	1	0	0	0	0	0	0	0
1	1	0	0	0	0	0	0	0
1	1	0	0	0	0	0	0	0
0	0	0	0	0	0	0	0	0
1	1	0	0	0	0	0	0	0
0	0	0	0	0	0	0	0	0
1	1	0	0	0	0	0	0	0
1	1	0	0	0	0	0	0	0
1	1	0	0	0	0	0	0	0

1	1	0	0	0	0	0	0	0
1	1	0	0	0	0	0	0	0
1	1	0	0	0	0	0	0	0
1	1	0	0	0	0	0	0	0
1	1	0	0	0	0	0	0	0
1	1	0	0	0	0	0	0	0
1	1	0	0	0	0	0	0	0
1	1	0	0	0	0	0	0	0
0	0	0	0	0	0	0	0	0
1	1	0	1	0	0	0	0	0
1	1	0	0	0	0	0	0	0
1	1	0	0	0	0	0	0	0
1	1	0	0	0	0	0	0	0
1	1	0	0	0	0	0	0	0
1	1	0	0	0	0	0	0	0
1	1	0	0	0	0	0	0	0
0	0	0	0	0	0	0	0	0
1	1	0	0	0	0	0	0	0
1	1	0	0	0	0	0	0	0
1	1	0	0	0	0	0	0	0
1	1	0	0	0	0	0	0	0
1	1	0	0	0	0	0	0	0
1	1	0	0	0	0	0	0	0
1	1	0	0	0	0	0	0	0
1	1	0	0	0	0	0	0	0
1	1	0	0	0	0	0	0	0
1	1	0	0	0	0	0	0	0
0	0	0	0	0	0	0	0	0
1	1	0	0	0	0	0	0	0
1	1	0	0	0	0	0	0	0
0	0	0	0	0	0	0	0	0
1	1	0	0	0	0	0	0	0
1	1	0	0	0	0	0	0	0
1	1	0	0	0	0	0	0	0
1	1	0	0	0	0	0	0	0
1	0	0	0	0	0	0	0	0
1	1	0	0	0	0	0	0	0
1	1	0	0	0	0	0	0	0
1	1	1	0	0	0	0	0	0
1	1	0	0	0	0	0	0	0
1	1	0	0	0	0	0	0	0
0	0	0	0	0	0	0	0	0
1	1	0	0	0	0	0	0	0
1	1	0	0	0	0	0	0	0
1	1	0	0	0	0	0	0	0
1	1	0	0	0	0	0	0	0
1	1	0	0	0	0	0	0	0
0	0	0	0	0	0	0	0	0
1	1	0	0	0	0	0	0	0
0	1	0	0	0	0	0	0	0
1	1	0	0	0	0	0	0	0
1	1	0	0	0	0	0	0	0
1	1	0	0	0	0	0	0	0
1	1	0	0	0	0	0	0	0

1	1	0	1	1	1	1	0	0
1	1	0	0	0	0	0	0	0
0	0	0	0	0	0	0	0	0
1	1	0	0	0	1	0	0	1
1	1	0	0	0	0	0	0	0
1	0	1	0	0	0	0	0	0
1	1	0	0	0	0	0	0	0
1	1	0	0	0	0	0	0	0
1	0	0	0	0	0	0	0	0
0	0	0	0	0	0	0	0	0
1	1	0	0	0	1	0	0	0
1	1	0	0	0	0	0	0	0
1	1	0	0	0	0	0	0	0
0	0	0	0	0	1	1	0	0
1	1	0	0	0	0	0	0	0
1	1	0	0	0	0	0	0	0
0	1	0	0	0	0	0	0	0
0	0	0	0	0	1	1	0	0
1	1	0	0	0	0	0	0	0
1	1	1	0	0	0	0	0	0
1	1	0	0	0	0	0	0	0
1	1	0	0	0	0	0	0	0
1	1	0	0	0	0	0	0	0
1	1	0	0	0	0	0	0	0
1	1	0	0	0	0	0	0	0
1	1	0	0	0	0	0	0	0
1	1	0	0	0	0	0	0	0
1	1	0	0	0	0	0	0	0
1	1	0	0	0	0	0	0	0
0	0	0	0	0	0	0	0	0
1	1	0	0	0	0	0	0	0
1	1	0	0	0	0	0	0	0
1	1	0	0	0	0	0	0	0
1	1	0	0	0	0	0	0	0
1	1	0	0	0	0	0	0	0
1	1	0	0	0	0	0	0	0
1	1	0	0	0	0	0	0	0
1	1	0	0	0	0	0	0	0
1	1	0	0	0	0	0	0	0
1	1	0	0	0	0	0	0	0
1	0	1	0	0	1	1	0	0
1	0	1	0	0	0	0	0	0
1	1	0	0	0	0	0	0	0
1	1	0	0	0	0	0	0	0
1	1	0	0	0	0	0	0	0
1	1	0	1	0	0	0	0	0
1	1	0	0	0	0	0	0	0
1	1	0	0	0	0	0	0	0
1	1	0	0	0	0	0	0	0
1	1	1	0	0	0	0	0	0
1	1	0	0	0	0	0	0	0
1	1	0	0	0	0	0	0	0
1	1	0	0	0	0	0	0	0
1	1	0	0	0	0	0	0	0

1	1	0	0	0	0	0	0	0
1	1	0	0	0	0	0	0	0
1	1	0	0	0	0	0	0	0
1	1	0	0	0	0	0	0	0
1	1	0	0	0	0	0	0	0
1	1	0	0	0	0	0	0	0
0	0	0	0	0	0	0	0	0
1	1	0	0	0	0	0	0	0
1	1	0	0	0	0	0	0	0
1	1	0	0	0	0	0	0	0
1	1	0	0	0	0	0	0	0
1	1	0	0	0	0	0	0	0
1	1	0	0	0	0	0	0	0
1	1	0	0	0	1	1	0	0
1	1	0	0	0	0	0	0	0
1	1	0	0	0	0	0	0	0
1	1	1	0	0	0	0	0	0
1	1	0	0	0	0	0	0	0
1	1	0	0	0	0	0	0	0
0	0	0	0	0	0	0	0	0
1	1	0	0	0	1	0	0	0
1	1	0	0	0	0	0	0	0
1	1	0	0	0	0	0	0	0
1	1	0	0	0	0	0	0	0
0	0	0	0	0	0	0	0	0
1	1	0	0	0	0	0	0	0
0	0	0	0	0	0	0	0	0
1	1	0	0	0	0	0	0	0
0	0	0	0	1	0	0	0	0
0	0	0	0	0	0	0	0	0
1	0	0	1	0	0	0	0	0
1	0	1	0	0	0	0	0	0
1	0	0	0	0	1	1	0	1
1	1	0	0	0	0	0	0	0
1	0	0	0	0	1	1	0	0
0	0	0	0	0	0	0	0	0
1	1	0	0	0	0	0	0	0
1	0	1	0	0	1	0	0	0
1	1	0	0	0	0	0	0	0
0	0	0	0	0	1	1	0	1
0	0	0	0	0	0	0	0	0
1	1	0	0	0	0	0	0	0
1	1	0	0	0	0	0	0	0
0	0	0	0	0	0	0	0	0
0	0	0	0	0	0	0	0	0
0	0	0	0	0	0	0	0	0
1	1	0	0	0	0	0	0	0
1	0	1	0	0	1	0	0	1
1	1	0	0	0	0	0	0	0
1	1	0	0	0	0	0	0	0
1	1	0	0	0	0	0	0	0
1	1	0	0	0	0	0	0	0
0	0	0	0	0	0	0	0	0
1	1	0	0	0	0	0	0	0

1	1	0	0	0	0	0	0	0
1	1	0	0	0	0	0	0	0
0	0	0	0	0	0	0	0	0
1	1	0	0	0	0	0	0	0
1	1	0	0	0	0	0	0	0
1	1	0	0	0	0	0	0	0
1	1	0	0	0	0	0	0	0
1	1	0	0	0	0	0	0	0
1	1	0	0	0	0	0	0	0
1	1	0	0	0	0	0	0	0
1	1	1	0	0	0	0	0	0
1	1	0	0	0	0	0	0	0
1	1	0	0	0	0	0	0	0
1	1	0	0	0	0	0	0	0
1	1	0	0	0	0	0	0	0
1	1	0	0	0	0	0	0	0
1	1	0	0	0	0	0	0	0
1	1	0	0	0	0	0	0	0
1	0	1	0	0	0	0	0	0
1	1	1	0	0	0	0	0	0
1	1	0	0	0	0	0	0	0
1	1	0	0	0	0	0	0	0
1	1	0	0	0	0	0	0	0
1	1	0	0	0	0	0	0	0
1	1	0	0	0	0	0	0	0
1	1	0	0	0	0	0	0	0
1	1	0	0	0	0	0	0	0
1	0	0	0	0	0	0	0	0
1	1	0	0	0	0	0	0	0
1	1	0	0	0	0	0	0	0
1	1	0	0	0	0	0	0	0
1	1	0	0	0	0	0	0	0
1	1	0	0	0	0	0	0	0
1	1	0	0	0	0	0	0	0
1	1	0	0	0	0	0	0	0
1	0	0	0	0	0	0	0	0
0	0	0	0	0	0	0	0	0
1	1	0	0	0	0	0	0	0
1	1	0	0	0	0	0	0	0
1	1	0	0	0	0	0	0	0
1	1	0	0	0	0	0	0	0
1	1	0	0	0	0	0	0	0
0	0	0	0	0	0	0	0	0
1	1	0	0	0	0	0	0	0
1	1	0	0	0	0	0	0	0
1	1	0	0	0	0	0	0	0
1	1	0	0	0	0	0	0	0
1	1	0	0	0	0	0	0	0
1	1	0	0	0	0	0	0	0
1	1	0	0	0	0	0	0	0
1	1	1	0	0	0	0	0	0
1	1	0	0	0	0	0	0	0
1	1	0	0	0	0	0	0	0
1	1	0	0	0	0	0	0	0

1	1	0	0	0	0	0	0	0
1	1	0	0	0	0	0	0	0
1	1	0	0	0	1	0	1	0
1	1	0	0	0	0	0	0	0
1	1	0	0	0	0	0	0	0
1	1	0	0	0	0	0	0	0
1	1	0	0	0	0	0	0	0
1	1	0	0	0	0	0	0	0
1	1	0	0	0	0	0	0	0
1	1	0	0	0	0	0	0	0
1	1	1	0	0	0	0	0	0
1	1	0	0	0	0	0	0	0
0	0	0	0	0	0	0	0	0
1	1	0	0	0	0	0	0	0
1	1	0	0	0	0	0	0	0
1	1	0	0	0	0	0	0	0
1	1	0	0	0	0	0	0	0
0	0	0	0	0	0	0	0	0
1	1	0	0	0	0	0	0	0
1	0	1	0	0	1	1	0	1
0	0	0	0	0	0	0	0	0
1	1	0	0	0	0	0	0	0
1	1	0	0	0	0	0	0	0
1	0	0	0	1	0	0	0	0
1	0	0	1	0	0	0	0	0
0	0	0	0	0	1	0	0	0
1	1	0	0	0	0	0	0	0
0	0	0	0	0	1	1	0	0
0	0	0	0	0	0	0	0	0
1	1	0	0	0	0	0	0	0
1	1	0	0	0	1	0	0	1
1	1	1	0	0	0	0	0	0
1	1	0	0	0	1	1	0	0
0	0	0	0	0	1	1	0	1
1	1	0	0	0	0	0	0	0
1	1	0	0	0	0	0	0	0
0	0	0	0	0	0	0	0	0
1	1	1	0	0	0	0	0	0
1	1	0	0	0	0	0	0	0
1	1	0	0	0	0	0	0	0
1	1	0	0	0	0	0	0	0
1	1	0	0	0	0	0	0	0
1	1	0	0	0	0	0	0	0
1	1	0	0	0	0	0	0	0
0	0	0	0	0	0	0	0	0
0	0	0	0	0	0	0	0	0
1	1	0	0	0	0	0	0	0
1	1	0	0	0	0	0	0	0
1	1	0	0	0	0	0	0	0
1	1	0	0	0	0	0	0	0
1	1	0	0	0	0	0	0	0
1	1	0	0	0	0	0	0	0
0	0	0	0	0	0	0	0	0
1	0	0	1	0	0	0	0	0

1	1	0	0	0	0	0	0	0
1	1	0	0	0	0	0	0	0
1	1	0	0	0	0	0	0	0
0	0	0	0	0	0	0	0	0
1	1	0	0	0	0	0	0	0
1	1	0	0	0	0	0	0	0
1	1	0	0	0	0	0	0	0
1	1	0	0	0	0	0	0	0
1	1	0	0	0	0	0	0	0
0	0	0	0	0	0	0	0	0
0	0	0	0	0	0	0	0	0
1	1	0	0	0	0	0	0	0
1	1	0	0	0	0	0	0	0
0	0	0	0	0	0	0	0	0
0	0	0	0	0	0	0	0	0
1	1	0	0	0	0	0	0	0
1	1	0	0	0	0	0	0	0
1	1	0	0	0	0	0	0	0
0	0	0	0	0	0	0	0	0
1	1	0	0	0	0	0	0	0
1	1	0	0	0	0	0	0	0
1	1	0	0	0	0	0	0	0
1	1	0	0	0	0	0	0	0
1	1	0	0	0	0	0	0	0
1	1	0	0	0	0	0	0	0
0	0	0	0	0	0	0	0	0
1	1	0	0	0	0	0	0	0
1	1	0	0	0	0	0	0	0
1	1	0	0	0	0	0	0	0
0	0	0	0	0	0	0	0	0
0	0	0	0	0	0	0	0	0
1	1	0	0	0	0	0	0	0
1	1	0	0	0	0	0	0	0
1	1	0	0	0	0	0	0	0
1	1	0	0	0	0	0	0	0
1	1	0	0	0	0	0	0	0
1	1	0	0	0	0	0	0	0
1	1	0	0	0	0	0	0	0
1	1	0	0	0	0	0	0	0
1	1	0	0	0	0	0	0	0
1	1	0	0	0	0	0	0	0
0	0	0	0	0	0	0	0	0
1	1	0	0	0	0	0	0	0
1	1	0	0	0	1	0	0	0
1	0	0	1	0	0	0	0	0
1	1	0	0	0	0	0	0	0
0	0	0	0	0	0	0	0	0
1	1	0	0	0	0	0	0	0
1	1	0	0	0	0	0	0	0
1	1	0	0	0	0	0	0	0
1	1	0	0	0	0	0	0	0
1	1	0	0	0	0	0	0	0
0	0	0	0	0	0	0	0	0
1	0	1	1	0	0	0	0	0

0	0	0	0	0	0	0	0	0
1	1	0	0	0	1	1	0	0
1	1	0	0	0	1	0	0	0
1	1	0	0	0	0	0	0	0
1	1	0	0	0	0	0	0	0
1	1	0	0	0	0	0	0	0
0	0	0	0	0	0	0	0	0
1	1	0	0	0	0	0	0	0
1	1	0	0	0	0	0	0	0
1	1	0	0	0	0	0	0	0
0	0	0	0	0	0	0	0	0
0	0	0	0	0	0	0	0	0
1	1	0	0	0	0	0	0	0
0	0	0	0	0	0	0	0	0
1	1	0	0	0	1	1	0	1
1	1	0	0	0	0	0	0	0
1	1	1	0	0	0	0	0	0
1	1	0	0	0	0	0	0	0
1	1	0	0	0	0	0	0	0
1	1	0	0	0	0	0	0	0
1	1	0	0	0	0	0	0	0
1	1	0	0	0	0	0	0	0
1	1	0	0	0	0	0	0	0
1	1	0	0	0	0	0	0	0
1	1	0	0	0	0	0	0	0
1	1	0	0	0	0	0	0	0
1	1	0	0	0	0	0	0	0
0	0	0	0	0	0	0	0	0
1	1	0	0	0	0	0	0	0
1	1	0	0	0	0	0	0	0
1	1	0	0	0	0	0	0	0
1	1	0	0	0	0	0	0	0
1	1	0	0	0	0	0	0	0
1	0	1	0	0	0	0	0	0
1	1	0	0	0	0	0	0	0
0	0	0	0	0	0	0	0	0
1	1	0	0	0	0	0	0	0
1	1	0	0	0	0	0	0	0
1	1	0	0	0	0	0	0	0
1	1	0	0	0	0	0	0	0
1	1	0	0	0	0	0	0	0
1	1	0	0	0	0	0	0	0
1	1	0	0	0	0	0	0	0
1	1	0	0	0	0	0	0	0
1	1	0	0	0	0	0	0	0
1	1	0	0	0	0	0	0	0
1	1	0	0	0	0	0	0	0
0	0	0	0	0	0	0	0	0
0	0	0	0	0	0	0	0	0
0	0	0	0	0	0	0	0	0
0	0	0	0	0	0	0	0	0
1	1	0	0	0	0	0	0	0
0	0	0	0	0	0	0	0	0
1	1	0	0	0	0	0	0	0

1	1	0	0	0	0	0	0	0
1	1	0	0	0	0	0	0	0
1	1	0	0	0	0	0	0	0
1	1	0	0	0	0	0	0	0
1	1	0	0	0	0	0	0	0
1	1	0	0	0	0	0	0	0
1	1	0	0	0	0	0	0	0
1	1	0	0	0	0	0	0	0
1	1	0	0	0	0	0	0	0
1	1	0	0	0	0	0	0	0
1	1	0	0	0	0	0	0	0
0	0	0	0	0	0	0	0	0
1	1	0	0	0	0	0	0	0
1	1	0	0	0	0	0	0	0
1	1	0	0	0	0	0	0	0
1	1	0	0	0	0	0	0	0
1	1	0	0	0	0	0	0	0
1	1	0	0	0	0	0	0	0
1	1	0	0	0	0	0	0	0
1	1	0	0	0	0	0	0	0
1	1	0	0	0	0	0	0	0
1	1	0	0	0	0	0	0	0
1	1	0	0	0	0	0	0	0
1	1	1	0	0	0	0	0	0
1	1	0	0	0	0	0	0	0
0	0	0	0	0	0	0	0	0
1	1	0	0	0	0	0	0	0
1	1	0	0	0	0	0	0	0
0	0	0	0	0	0	0	0	0
0	0	0	0	0	0	0	0	0
1	1	0	0	0	0	0	0	0
1	1	0	0	0	0	0	0	0
0	0	0	0	0	0	0	0	0
1	1	0	0	0	0	0	0	0
0	0	0	0	0	0	0	0	0
1	1	0	0	0	0	0	0	0
1	1	0	0	0	0	0	0	0
1	1	0	0	0	0	0	0	0
1	1	0	0	0	0	0	0	0
1	1	0	0	0	0	0	0	0
1	1	0	0	0	0	0	0	0
1	1	0	0	0	0	0	0	0
1	1	0	0	0	0	0	0	0
1	0	0	0	0	0	0	0	0
1	1	0	0	0	0	0	0	0
1	1	0	0	0	0	0	0	0
1	1	0	0	0	0	0	0	0
1	1	0	0	0	0	0	0	0
1	1	0	0	0	0	0	0	0
1	1	0	0	0	0	0	0	0
1	1	0	0	0	0	0	0	0
1	1	0	0	0	0	0	0	0
1	1	0	0	0	0	0	0	0
1	1	0	0	0	0	0	0	0

1	1	0	0	0	0	0	0	0
1	1	0	0	0	0	0	0	0
0	0	0	0	0	0	0	0	0
0	0	0	0	0	0	0	0	0
0	0	0	0	0	0	0	0	0
1	1	0	0	0	0	0	0	0
1	1	0	0	0	0	0	0	0
1	1	0	0	0	0	0	0	0
1	1	0	0	0	0	0	0	0
1	1	0	0	0	0	0	0	0
1	1	0	0	0	0	0	0	0
1	1	0	0	0	0	0	0	0
0	0	0	0	0	0	0	0	0
1	0	1	0	0	0	0	0	0
1	0	1	0	0	0	0	0	0
1	1	0	0	0	0	0	0	0
0	0	0	0	0	0	0	0	0
1	1	0	0	0	0	0	0	0
1	1	0	0	0	0	0	0	0
1	1	0	0	0	0	0	0	0
1	1	0	0	0	0	0	0	0
1	1	0	0	0	0	0	0	0

其他药物	是否顺产	出生体重	是否母乳	WBC	N	N百分比	L	L百分比
0	0	3.30	1	4.70	2.54	54.00	1.65	35.10
0	1	3.00	1	3.44	0.98	28.60	2.08	60.40
0	1	3.60	1	5.31	2.33	43.90	2.61	49.20
0	1	2.95	1	9.07	6.29	69.40	2.04	22.50
0	0	3.30	1	5.46	3.11	56.90	1.87	34.20
0	0	3.90	1	10.46	8.25	78.90	1.70	16.30
0	1	3.80	1	8.65	2.53	29.30	5.62	65.00
0	1	3.15	1	5.35	2.58	48.30	2.33	43.60
0	1	3.00	1	5.04	2.99	59.30	1.76	34.90
0	1	3.10	1	5.24	2.15	41.00	2.53	48.30
0	1	3.58	1	7.88	5.22	66.20	1.90	24.10
0	1	3.60	1	8.01	3.87	48.30	3.58	44.70
0	1	3.20	1	8.70	5.09	58.50	2.66	30.60
0	0	3.85	1	5.31	2.04	38.40	2.88	54.20
0	1	2.92	1	13.72	10.40	75.80	2.17	15.80
0	1	3.00	1	5.31	2.84	53.50	1.92	36.20
0	0	2.70	1	13.03	10.20	78.30	1.88	14.40
0	0	3.40	1	3.86	1.54	39.90	1.91	49.40
0	1	3.00	1	10.60	7.92	74.70	2.06	19.40
0	1	3.30	1	15.26	8.90	58.30	5.81	38.10
0	1	3.20	1	8.23	5.47	66.50	1.91	23.20
0	1	3.30	1	9.65	6.71	69.50	2.53	26.20
0	1	3.00	1	4.36	2.28	52.30	1.79	41.10
0	1	2.75	1	7.28	3.95	54.30	2.76	37.90
0	1	2.50	1	10.26	5.95	58.00	3.72	36.30
0	1	3.00	1	2.97	1.56	52.50	1.10	37.00
0	1	2.70	1	5.84	2.91	49.80	2.51	43.00
0	1	3.10	1	12.70	9.75	76.80	1.91	15.00
0	1	3.36	1	5.10	3.12	61.10	1.79	35.00
0	1	3.00	1	6.48	3.16	48.70	2.94	45.40
0	1	3.00	1	4.50	2.34	52.00	1.94	43.20
0	1	3.10	1	5.92	2.46	41.50	3.10	52.30
0	1	3.00	1	13.31	9.92	74.50	2.40	18.00
0	1	2.80	1	5.39	2.71	50.30	2.27	42.10
0	1	3.00	1	16.44	13.09	79.60	2.35	14.30
0	0	6.30	1	5.08	1.88	37.00	2.98	58.70
0	1	3.02	1	10.17	7.65	75.20	2.02	19.90
1	0	3.00	1	5.00	1.94	38.80	2.24	44.80
0	1	3.00	1	5.48	2.71	49.40	2.48	45.30
0	1	3.00	1	17.44	14.67	84.10	1.50	8.60
1	0	3.00	1	3.75	1.48	39.50	1.77	47.20
0	1	3.20	1	6.10	4.18	68.60	1.55	25.40
0	1	3.00	1	7.04	3.87	55.00	2.45	34.80
0	1	2.50	1	5.20	2.91	56.00	1.88	36.20
0	1	3.00	1	3.20	1.38	43.00	1.66	52.00
0	0	3.30	1	5.01	2.72	54.30	1.44	28.70
0	1	3.00	1	4.94	1.60	32.40	2.93	59.30
0	1	2.70	1	7.65	2.98	38.90	4.06	53.10
0	0	3.80	1	5.84	2.56	43.90	2.87	49.10
0	0	3.30	1	7.01	4.35	62.00	2.17	30.90
0	1	3.00	1	10.52	7.65	72.70	2.37	22.50
0	1	1.50	1	7.33	4.05	55.20	2.37	32.30
0	1	3.00	1	3.00	1.56	52.00	1.31	43.70

0	0	3.50	1	4.80	2.81	58.50	1.55	32.20
0	1	3.00	1	5.70	3.91	68.60	1.50	26.30
0	0	4.00	1	5.75	4.14	72.00	1.31	22.80
0	1	3.20	1	14.74	10.98	74.50	2.23	15.10
0	1	3.30	1	5.81	2.99	51.50	2.33	40.10
0	1	2.80	1	4.12	2.21	53.70	1.39	33.70
0	1	3.00	1	4.14	1.94	46.80	1.79	43.20
0	0	3.40	1	6.29	2.15	34.20	3.21	51.00
0	1	3.00	1	4.50	2.79	62.00	1.49	33.00
0	0	3.20	1	11.37	8.35	73.40	2.62	23.00
0	1	4.80	1	6.91	3.09	44.70	3.37	48.80
0	1	3.10	1	6.67	3.93	58.90	2.19	32.80
0	1	4.00	1	4.38	1.59	36.30	2.27	51.80
0	0	2.90	1	5.80	2.69	46.30	2.36	40.70
0	0	3.30	1	6.07	2.39	39.30	2.93	48.30
0	1	2.60	1	6.82	3.95	57.90	2.43	35.60
0	0	2.95	1	7.44	3.39	45.60	3.48	46.80
0	1	2.90	1	6.68	3.69	55.20	2.28	34.10
0	1	3.20	1	6.50	3.26	50.20	2.74	42.20
0	1	2.85	1	5.72	2.53	44.30	2.17	37.90
0	1	2.96	1	4.95	2.39	48.20	2.19	44.20
0	1	3.00	1	7.88	4.22	53.50	3.38	42.90
0	1	2.68	1	6.86	2.56	37.30	3.58	52.20
0	1	2.90	1	8.73	5.06	58.00	3.04	34.80
0	1	3.00	1	4.76	1.89	39.70	2.62	55.00
0	0	3.00	1	3.41	1.73	50.70	1.17	34.30
0	1	3.50	1	12.10	8.99	74.30	2.46	20.30
0	1	3.00	1	8.10	4.09	50.50	3.25	40.10
0	0	2.95	1	6.37	3.78	59.30	2.17	34.10
0	1	3.40	1	5.13	2.31	45.00	2.41	47.00
0	1	3.30	1	11.63	10.57	90.90	0.53	4.60
0	1	3.00	1	8.27	4.57	55.30	2.99	36.20
0	1	2.85	1	4.54	2.51	55.30	1.57	34.60
0	1	3.40	1	12.20	7.23	59.30	4.23	34.70
0	1	3.20	1	5.83	3.75	64.30	1.72	29.50
0	1	2.80	1	11.39	8.68	76.20	2.12	18.60
0	1	3.00	1	7.98	3.31	41.50	4.19	52.50
0	0	2.30	1	4.92	2.31	46.90	2.16	43.90
0	0	6.30	1	5.64	1.81	32.10	3.29	58.30
0	1	3.00	1	11.93	10.28	86.20	1.26	10.60
0	1	3.30	1	11.15	7.59	68.10	2.77	24.80
0	1	2.00	1	4.37	1.65	37.70	2.31	52.90
0	1	2.85	1	3.76	1.86	49.50	1.69	44.90
0	1	3.10	1	5.24	3.12	59.50	1.55	29.60
0	1	2.85	1	10.46	7.71	73.70	2.02	19.30
0	1	2.90	1	6.98	6.12	87.70	0.71	10.20
0	1	3.60	1	4.84	2.51	51.90	1.96	40.50
0	0	2.80	1	9.42	6.09	64.70	2.71	28.80
0	1	3.25	1	10.62	8.97	84.50	1.16	10.90
0	0	3.40	1	6.68	4.68	70.00	1.62	24.20
0	1	4.00	1	6.96	4.29	61.60	1.80	25.80
0	1	3.30	1	5.74	3.29	57.40	1.74	30.30
0	1	3.20	1	9.68	7.31	75.50	1.83	18.90
0	1	2.98	1	14.99	10.79	72.00	3.49	23.30

0	0	3.30	1	5.28	2.46	46.60	2.42	45.80
0	1	2.85	1	5.19	3.35	64.50	1.56	30.00
0	1	2.65	1	6.70	3.24	48.30	2.96	44.20
0	0	2.80	1	5.90	2.67	45.30	2.82	47.80
0	1	3.50	1	5.39	2.47	45.80	2.33	43.20
0	1	3.20	1	5.22	3.40	65.20	1.37	26.20
0	1	3.00	1	5.18	1.85	35.80	2.93	56.60
0	1	2.85	1	6.76	3.60	53.20	2.77	41.00
0	1	3.00	1	3.39	1.99	58.70	0.96	28.30
0	1	3.30	1	7.69	3.08	40.10	4.00	52.00
0	1	3.00	1	4.58	2.15	47.00	1.79	39.10
0	0	2.85	1	6.78	3.48	51.40	2.91	42.90
0	1	3.00	1	4.52	2.92	64.70	1.27	28.10
0	1	3.00	1	7.72	4.84	62.70	2.27	29.40
0	1	3.20	1	5.52	2.91	52.80	2.05	37.10
0	1	3.50	1	7.83	4.96	63.37	2.38	30.40
0	1	3.00	1	7.23	4.22	58.40	2.53	35.00
0	1	3.20	1	8.85	4.66	52.60	3.18	35.90
0	1	3.20	1	7.06	3.33	47.10	3.42	48.50
0	1	2.80	1	3.83	1.29	33.60	2.17	56.70
0	0	3.00	1	5.36	2.97	55.40	1.91	35.60
0	1	3.10	1	6.40	4.73	73.90	1.39	21.70
0	1	3.00	1	6.52	3.15	48.30	2.76	42.40
0	1	3.30	1	4.05	2.17	53.60	1.45	35.80
0	0	3.90	1	6.08	2.33	38.40	2.92	48.00
0	1	2.80	1	8.49	4.64	54.60	3.12	36.70
0	1	3.20	1	4.84	2.27	46.90	1.69	34.90
0	1	2.50	1	4.37	2.68	61.30	1.24	28.40
0	1	2.85	1	3.46	1.69	48.90	1.34	38.70
0	0	3.20	1	4.74	3.06	64.50	1.18	24.90
0	1	3.50	1	19.74	16.92	85.70	1.90	9.60
0	0	3.60	1	13.10	10.38	79.20	1.39	10.60
0	1	3.20	1	10.36	8.14	78.60	1.32	12.70
0	1	3.30	1	3.75	1.89	50.40	1.60	42.70
0	1	3.60	1	6.15	3.70	60.17	1.96	31.90
0	0	4.20	1	7.88	3.45	43.80	3.67	46.60
0	0	3.40	1	13.24	10.17	76.80	2.04	15.40
0	1	3.00	1	6.07	3.22	53.00	2.23	36.70
0	1	3.10	1	4.23	2.01	47.50	1.78	42.10
0	1	3.50	1	7.03	4.20	59.80	2.29	32.60
0	1	3.27	0	5.58	3.54	63.40	1.61	28.90
0	1	3.00	1	6.14	2.39	38.90	3.27	53.30
0	1	2.50	1	4.48	1.63	36.40	2.50	55.80
0	1	3.50	1	4.85	2.68	55.30	1.93	39.80
0	1	6.80	1	5.65	2.15	38.00	3.02	53.50
0	1	3.50	1	5.79	3.97	68.50	1.38	23.80
0	1	3.45	1	6.08	2.76	45.40	2.50	41.10
0	1	3.60	1	9.26	8.43	91.00	0.64	6.90
0	1	3.00	1	5.07	2.21	43.50	1.85	36.50
0	1	3.10	1	3.30	1.82	55.00	1.02	31.00
0	1	3.30	1	5.52	2.78	50.40	2.19	39.70
0	1	3.60	1	5.15	2.05	39.80	2.70	52.40
0	1	3.20	1	4.45	2.50	56.20	1.58	35.50
0	0	2.80	1	4.86	3.10	63.70	1.30	26.70

0	1	3.20	1	6.56	3.60	54.90	2.38	36.30
0	1	3.00	1	6.10	3.08	50.50	2.22	36.40
1	1	3.00	1	7.33	4.77	65.10	1.71	23.30
0	1	3.50	1	5.66	3.36	59.40	2.04	36.00
0	0	2.30	1	5.10	2.81	55.00	1.99	39.00
0	1	3.60	1	4.99	4.07	81.60	0.65	13.00
0	1	3.00	1	10.91	5.07	46.50	5.21	47.80
0	1	2.70	1	4.25	1.50	35.20	2.53	59.50
0	1	3.40	1	6.46	4.39	67.90	1.50	23.20
0	1	3.20	1	7.61	6.00	78.80	1.38	18.10
0	1	3.40	1	8.15	4.73	58.00	2.67	32.80
0	0	3.20	0	4.53	2.08	46.00	1.86	41.00
0	1	3.00	1	5.23	2.56	48.90	2.17	41.50
0	1	2.50	1	4.44	2.35	53.00	1.81	40.80
0	1	3.10	1	6.66	2.72	40.80	2.78	41.70
0	1	3.40	0	6.68	2.87	42.90	3.08	46.10
0	1	3.50	1	5.42	3.04	56.10	2.04	37.60
0	1	2.80	1	4.67	2.05	43.80	2.23	47.80
0	1	3.30	1	6.81	4.46	65.50	1.83	26.90
0	1	2.90	1	5.46	3.19	58.40	1.50	27.50
0	1	3.20	1	11.48	6.65	57.90	4.26	37.10
0	1	3.50	1	8.73	3.76	43.10	4.44	50.90
0	1	3.20	1	4.13	1.96	47.40	1.84	44.60
0	1	2.80	1	6.16	2.32	37.70	2.14	34.70
0	1	3.10	1	3.50	1.64	46.90	1.58	45.00
0	0	3.35	1	13.57	11.83	87.20	1.38	10.20
0	1	3.20	1	5.02	3.07	61.10	1.57	31.30
0	1	3.60	1	5.47	3.50	64.00	1.58	28.90
0	1	3.25	1	13.99	11.14	79.60	1.54	11.00
0	1	3.50	1	5.52	2.91	52.70	2.07	37.50
0	1	3.20	1	4.44	2.01	45.20	2.13	48.00
0	1	2.70	1	5.85	3.53	60.40	1.80	30.70
0	1	2.80	1	3.01	1.47	48.80	1.33	44.20
0	1	3.00	1	4.04	1.90	47.10	1.65	40.80
0	1	3.15	1	3.43	1.95	56.90	1.14	33.20
0	1	5.40	1	12.91	8.59	66.50	3.21	24.90
0	1	3.20	1	4.06	1.94	47.80	1.78	43.80
0	1	3.30	1	7.02	3.58	51.00	2.90	41.30
0	0	2.85	1	12.62	8.61	68.20	3.12	24.70
0	0	3.20	1	3.77	1.98	52.50	1.45	38.50
0	1	3.30	1	10.28	8.20	79.80	1.83	17.80
0	1	3.10	1	3.15	1.54	48.80	1.32	41.80
0	1	3.20	1	4.94	3.08	62.40	1.20	24.30
0	1	3.20	1	10.52	5.63	53.50	3.85	36.60
0	1	3.00	1	3.89	1.38	35.40	2.11	54.20
0	1	2.70	1	4.89	2.61	53.40	1.97	40.30
0	1	3.00	1	5.85	2.12	36.20	3.30	56.40
0	1	2.90	1	4.17	2.61	62.60	1.26	30.20
0	1	2.85	1	7.24	4.63	64.00	1.77	24.40
0	1	2.70	1	7.13	5.52	77.40	1.34	18.80
0	1	2.80	1	11.88	2.93	24.70	4.75	40.00
0	1	2.60	1	3.43	0.80	23.30	2.17	63.30
0	1	3.20	1	8.21	4.97	60.50	2.24	27.30
0	1	3.20	1	3.50	1.90	54.20	1.30	37.20

0	1	3.40	1	4.26	1.96	45.90	1.85	43.40
0	1	3.00	1	13.10	9.85	75.20	2.82	21.50
0	1	3.15	1	5.80	2.52	43.40	2.83	48.80
0	0	3.30	1	10.37	8.81	85.00	1.03	9.90
0	0	3.40	1	7.50	4.53	60.40	2.55	34.00
0	1	3.25	1	4.43	1.73	39.00	2.30	51.90
0	1	3.20	1	14.02	10.75	76.70	2.22	15.80
0	0	2.90	1	13.36	10.65	79.70	1.74	13.00
0	1	3.80	1	3.96	1.84	46.40	1.60	40.40
0	1	2.60	1	10.22	7.06	69.10	2.58	25.20
0	1	3.50	1	27.94	22.10	79.10	3.60	12.90
0	1	3.20	1	4.44	1.31	29.50	2.90	65.30
0	1	3.00	1	6.51	2.78	42.70	3.12	48.00
0	1	3.00	1	3.50	1.88	53.60	0.89	25.40
0	1	4.00	1	5.80	2.17	37.40	2.86	49.30
0	1	3.10	1	2.94	1.46	49.70	1.07	36.40
0	1	2.40	1	5.55	1.57	28.30	3.38	60.90
0	1	3.10	1	7.52	4.90	65.20	2.04	27.10
0	0	2.95	1	5.47	3.11	56.90	1.71	31.30
0	1	3.30	1	6.35	3.45	54.30	1.63	25.70
0	1	2.90	1	5.30	2.83	53.40	1.98	37.40
0	1	3.00	1	5.70	2.93	51.40	2.18	38.20
0	1	3.10	1	4.80	1.80	37.60	2.52	52.40
0	1	2.85	1	5.90	2.59	43.90	2.88	48.80
0	1	3.20	1	6.23	2.69	43.10	2.75	44.10
0	1	3.00	1	3.10	1.51	48.80	1.30	42.00
0	1	3.30	1	4.92	3.38	68.70	1.09	22.20
0	1	2.80	1	3.40	1.09	32.00	2.01	59.00
0	1	3.10	1	6.51	2.03	31.20	3.56	54.70
0	1	2.75	1	3.20	1.12	35.00	1.73	54.00
0	1	3.15	1	18.65	15.46	82.90	1.90	10.20
0	1	3.60	1	5.68	1.95	34.30	3.06	53.90
0	1	3.00	1	7.34	2.64	36.00	3.43	46.70
0	1	2.90	1	6.81	3.53	51.90	2.59	38.00
0	1	3.20	1	6.95	3.38	48.70	2.74	39.40
0	1	2.95	1	14.59	11.69	80.10	1.74	11.90
0	1	3.30	1	13.25	9.79	73.90	2.90	21.90
0	1	3.00	1	5.52	2.13	38.60	2.71	49.10
0	1	2.90	1	14.03	7.73	55.10	4.84	34.50
0	1	3.80	1	3.89	2.15	55.20	1.45	37.30
0	1	3.00	1	5.91	2.45	41.50	3.17	53.70
0	0	3.20	1	16.04	13.25	82.60	1.70	10.60
0	1	2.60	1	6.37	4.07	63.90	1.32	20.80
0	1	3.20	1	10.49	8.59	81.90	1.63	15.50
0	1	3.10	1	9.72	5.74	59.10	3.07	31.60
0	1	3.30	1	5.77	2.56	44.40	2.27	39.30
0	1	3.30	1	7.01	4.44	63.40	1.88	26.80
0	1	3.20	1	6.32	3.24	51.20	2.33	36.90
0	1	3.50	1	10.27	7.75	75.50	1.67	16.30
0	1	3.40	1	7.03	3.04	43.30	3.33	47.30
0	1	3.00	1	4.70	3.36	71.50	0.95	20.20
0	0	2.80	1	4.18	2.01	48.10	1.79	42.90
0	1	2.90	1	7.98	4.64	58.20	2.77	34.70
0	1	3.20	1	5.44	2.65	48.70	2.06	37.90

0	1	3.00	1	4.32	1.07	24.80	2.52	58.30
0	1	2.50	1	5.82	2.02	34.70	2.91	50.00
0	1	3.10	1	5.81	2.45	42.20	2.92	50.30
0	1	3.20	1	11.19	7.14	63.80	2.74	24.50
0	1	3.20	1	3.97	0.95	23.90	2.52	63.50
0	1	3.00	1	4.02	1.20	29.90	2.24	55.70
0	1	3.10	1	3.30	1.25	38.00	1.58	48.00
0	0	2.60	1	8.80	4.36	49.50	3.78	43.00
0	1	3.10	1	7.41	3.67	49.50	2.92	39.40
0	1	3.00	1	7.09	1.62	22.90	4.47	63.00
0	1	3.30	1	3.97	1.22	30.70	2.47	62.20
0	1	3.30	1	6.79	4.05	59.60	2.11	31.10
0	1	2.50	1	9.06	6.54	72.20	2.04	22.50
0	1	3.20	1	6.46	3.29	51.00	2.71	42.00
0	1	3.10	1	8.30	5.88	70.80	1.50	18.10
0	1	3.00	1	5.14	1.88	36.50	2.67	51.90
0	1	3.20	1	6.24	3.08	49.30	2.61	41.90
0	1	2.80	1	4.41	2.73	61.80	1.30	29.40
0	1	2.80	1	3.60	1.87	51.90	1.52	42.30
0	1	2.90	1	9.52	5.78	60.70	3.15	33.10
0	1	3.10	1	10.26	5.71	55.70	3.67	35.80
0	1	3.10	1	7.13	4.31	60.50	2.26	31.70
0	1	2.90	1	7.95	4.91	61.80	2.53	31.80
0	1	3.50	1	6.23	3.26	52.30	2.65	42.50
0	1	3.10	1	4.98	2.43	48.80	2.03	40.80
0	1	2.95	1	6.30	4.40	69.90	1.61	25.60
0	1	3.60	1	6.20	2.00	32.20	3.40	54.80
0	1	4.00	1	6.30	2.12	33.70	3.81	60.50
0	1	3.40	1	17.64	12.79	72.50	3.21	18.20
0	1	3.00	1	3.99	2.11	52.80	1.49	37.30
0	1	3.40	1	3.50	1.23	35.00	1.82	52.00
0	1	3.10	1	4.02	2.25	56.00	1.61	40.00
0	1	3.20	1	5.20	1.61	31.00	3.26	62.60
0	1	3.30	1	4.03	2.10	52.00	1.81	45.00
0	0	3.00	1	4.40	1.44	32.70	2.67	60.70
0	1	3.80	1	6.20	2.62	42.30	3.19	51.50
0	1	3.00	1	6.10	3.18	52.20	2.48	40.70
0	1	2.90	1	4.20	1.43	34.00	2.35	56.00
0	1	3.60	1	4.74	2.30	48.50	1.95	41.10
0	1	3.20	1	7.05	4.46	63.30	1.96	27.80
0	0	3.20	1	5.60	2.11	37.60	3.07	54.80
0	1	3.30	1	7.31	3.32	45.40	3.17	43.40
0	1	3.15	1	3.94	2.26	57.40	1.44	36.50
0	1	3.20	1	4.82	2.72	56.50	1.80	37.30
0	0	2.80	1	8.18	3.46	42.30	4.22	51.60
0	1	3.50	1	7.84	2.58	32.90	4.64	59.20
0	1	3.15	1	5.63	2.43	43.10	2.79	49.60
0	1	3.00	1	4.32	1.87	43.30	1.90	44.00
0	0	3.45	1	5.93	2.46	41.50	2.54	42.80
0	1	3.25	1	4.29	1.74	40.60	2.06	48.00
0	1	3.60	1	5.32	1.89	35.50	3.15	59.20
0	1	3.50	1	7.14	4.06	56.90	2.53	35.40
0	0	3.70	1	5.45	2.02	37.10	2.81	51.60
0	1	3.00	1	6.40	2.84	44.40	2.98	46.60

0	1	3.00	1	6.96	3.59	51.60	2.74	39.40
0	1	3.20	1	9.05	4.80	53.00	3.17	35.00
0	1	3.00	1	4.88	1.99	40.80	2.44	50.00
0	1	4.00	1	5.48	2.90	52.90	2.07	37.80
0	0	2.80	1	7.81	4.80	61.50	2.38	30.50
0	0	4.00	1	5.03	3.10	61.70	1.47	29.20
0	1	3.20	1	5.73	1.93	33.60	3.28	57.30
0	1	3.40	1	5.01	2.29	45.70	2.12	42.30
0	1	2.80	1	8.75	2.76	31.50	4.82	55.10
0	1	3.30	1	5.08	3.03	59.70	1.68	33.00
0	1	3.45	1	5.90	3.45	58.40	1.82	30.80
0	1	3.00	1	5.43	3.40	62.60	1.48	27.30
0	1	3.50	1	8.80	6.27	71.20	1.58	18.00
0	1	3.00	1	4.22	1.22	28.90	2.58	61.10
0	1	3.00	1	6.33	2.49	39.30	3.42	54.00
0	1	3.00	1	5.00	2.46	49.20	2.25	45.00
0	1	3.10	1	6.97	2.93	42.00	3.44	49.40
0	1	3.50	1	5.67	3.24	57.10	1.85	32.60
0	0	4.41	1	7.43	5.80	78.00	1.33	17.90
0	1	3.30	1	9.01	4.41	48.90	3.86	42.80
0	0	3.35	1	14.24	11.43	80.30	1.77	12.40
0	1	3.20	1	5.20	2.08	40.00	2.66	51.20
0	1	3.20	1	8.44	3.06	36.30	4.59	54.40
0	1	3.50	1	4.61	2.11	45.70	2.19	47.60
0	0	3.35	1	3.61	1.42	39.20	1.89	52.40
0	1	3.50	1	11.72	8.94	76.27	2.06	17.60
0	1	3.20	1	4.88	2.39	49.00	1.96	40.20
0	1	3.20	1	7.04	3.67	52.20	2.62	37.20
0	1	3.60	1	4.10	2.14	52.30	1.62	39.50
0	1	3.10	1	5.76	1.78	30.90	3.64	63.20
0	1	4.00	1	7.37	5.20	70.60	1.76	23.90
0	1	2.85	1	13.79	9.18	66.60	3.86	28.00
0	1	3.20	1	4.17	2.15	51.60	1.61	38.60
0	1	3.30	1	3.66	1.78	48.70	1.22	33.30
0	1	2.70	1	6.99	3.40	48.70	2.94	42.10
0	1	3.00	1	5.21	2.43	46.70	1.77	33.90
0	1	3.20	1	18.33	16.08	87.70	1.23	6.70
0	1	2.80	1	7.09	3.85	54.30	2.35	33.10
0	1	3.00	1	4.50	1.89	42.00	2.30	51.20
0	1	3.45	1	4.44	1.35	30.30	2.69	60.60
0	1	3.50	1	4.71	1.42	30.20	2.91	61.80
0	1	3.50	1	8.10	5.69	70.20	1.70	21.00
0	1	3.15	1	5.36	2.00	37.30	3.07	57.20
0	0	3.40	1	4.90	2.21	45.00	2.23	45.50
0	1	3.20	1	5.03	2.08	41.30	2.37	47.10
0	1	4.20	1	4.72	1.93	40.90	2.27	48.10
0	1	3.05	1	2.41	0.51	21.20	1.58	65.60
0	1	3.00	1	6.96	2.51	36.10	3.55	51.00
0	1	3.60	1	5.48	3.42	62.40	1.14	20.80
0	0	3.50	1	5.40	2.21	40.90	2.81	52.10
0	1	3.20	1	5.58	2.28	40.90	2.91	52.20
0	1	3.20	1	5.65	2.17	38.40	3.09	54.70
0	1	3.80	1	5.84	1.96	33.50	3.47	59.40
0	0	3.00	1	7.20	3.86	53.60	2.51	34.90

0	1	3.60	1	6.01	2.69	44.70	2.52	41.90
0	0	2.80	1	5.81	2.43	41.80	2.75	47.30
0	1	3.60	1	7.98	5.47	68.60	1.83	22.90
0	1	3.20	1	6.30	3.82	60.70	2.06	32.70
0	1	3.30	1	2.28	1.65	72.40	0.41	18.00
0	0	2.90	1	6.10	2.62	42.90	2.55	41.80
0	1	3.00	1	3.84	1.42	37.00	1.91	49.70
0	1	2.80	1	4.54	1.96	43.20	2.08	45.80
0	0	2.90	1	6.66	4.17	62.60	1.76	26.40
0	1	3.20	1	4.09	1.46	35.60	2.21	54.00
0	1	3.10	1	6.34	2.56	40.30	3.36	53.00
0	0	3.08	1	5.49	1.72	31.40	3.25	59.20
0	1	3.00	1	4.60	2.11	45.80	1.98	43.00
0	1	3.60	1	4.01	1.38	34.40	2.03	50.60
0	1	3.00	1	4.78	2.61	54.60	1.62	33.90
0	1	3.00	1	6.29	3.25	51.70	2.39	38.00
0	0	3.20	1	4.73	1.84	39.00	2.48	52.40
0	1	3.30	1	6.34	3.70	58.30	1.98	31.30
0	1	2.89	1	5.18	2.92	56.30	1.74	33.60
0	1	3.30	1	3.73	1.60	42.90	1.66	44.50
0	1	4.40	1	7.53	4.16	55.30	2.48	32.90
0	1	2.90	1	5.30	2.29	43.20	2.60	49.00
0	0	3.90	1	6.80	4.52	66.50	1.47	21.60
0	1	3.45	1	5.01	2.18	43.50	2.12	42.30
0	1	3.20	1	4.22	1.45	34.30	2.40	56.90
0	1	3.10	1	7.49	2.82	37.70	3.66	48.90
0	1	4.85	1	4.72	1.77	37.50	2.42	51.30
0	1	2.80	1	4.93	1.58	32.10	2.94	59.60
0	1	3.00	1	5.55	3.05	54.90	1.91	34.40
0	1	2.90	1	4.90	2.17	44.30	2.23	45.50
0	1	3.25	1	8.62	4.07	47.20	3.39	39.30
0	1	3.60	1	3.40	1.60	47.00	1.45	42.50
0	1	3.20	1	7.80	3.23	41.40	3.60	46.20
0	0	2.80	1	5.82	2.69	46.20	2.85	49.00
0	1	3.30	1	5.40	3.01	55.70	1.80	33.30
0	1	3.20	1	7.45	3.78	50.80	3.02	40.50
0	1	3.50	1	10.59	8.66	81.80	1.13	10.70
0	1	3.60	1	4.60	3.13	68.10	1.14	24.80
0	0	3.00	1	5.05	1.99	39.40	2.48	49.10
0	0	3.60	1	4.59	2.65	57.70	0.72	15.70
0	1	3.50	1	3.88	1.49	38.40	1.78	45.90
0	0	3.85	1	8.50	2.89	34.00	4.73	55.70
0	1	3.40	1	9.43	6.47	68.60	2.03	21.50
0	1	3.20	1	4.93	2.17	44.10	2.24	45.40
0	1	3.10	1	3.40	1.90	56.00	1.23	36.30
0	1	3.40	1	6.37	3.45	54.20	2.07	32.50
0	1	3.10	1	6.25	4.05	64.80	1.28	20.50
0	1	3.40	1	7.18	4.14	57.70	2.46	34.30
0	0	3.30	1	6.18	2.16	34.90	3.57	57.80
0	0	3.40	1	8.27	4.41	53.30	2.98	36.00
0	1	3.50	1	4.60	3.08	67.00	1.15	25.00
0	1	2.85	1	8.25	5.29	64.10	1.86	22.50
0	0	3.85	1	7.99	3.89	48.70	3.08	38.50
0	1	3.60	1	3.87	2.17	56.20	1.29	33.30

0	0	3.16	1	5.12	2.66	52.00	1.92	37.50
0	0	3.00	1	6.45	2.98	46.20	2.74	42.50
0	1	2.90	1	6.91	3.64	52.70	2.45	35.50
0	0	3.40	1	6.00	2.75	45.80	2.74	45.70
0	1	3.20	1	7.81	4.59	58.80	2.34	30.00
0	1	3.02	1	7.64	2.44	32.00	4.18	54.70
0	0	3.35	1	10.08	6.19	61.40	3.16	31.30
0	1	3.40	1	5.27	2.22	42.20	2.07	39.20
0	0	3.40	1	3.52	1.51	43.00	1.37	38.80
0	1	3.30	1	10.75	6.15	57.20	3.79	35.30
0	0	2.50	1	5.87	3.22	54.80	2.05	34.90
0	1	3.30	1	5.18	1.93	37.20	2.77	53.50
0	1	3.10	1	4.49	2.40	53.40	1.44	32.10
0	0	3.50	1	6.30	3.51	55.70	1.90	30.20
0	1	3.00	1	4.48	1.64	36.70	2.20	49.10
0	0	4.05	1	9.91	7.53	76.00	1.44	14.50
0	1	3.00	1	13.70	10.66	77.80	1.86	13.60
0	0	3.10	1	5.35	3.17	59.30	1.45	27.10
0	1	2.85	1	6.06	3.71	61.30	1.71	28.30
0	1	2.90	1	6.31	3.41	54.00	2.28	36.10
0	1	3.00	1	7.63	3.23	42.30	3.74	49.00
0	1	2.90	1	3.32	1.24	37.30	1.61	48.50
0	1	3.20	1	5.63	3.27	58.10	1.71	30.40
0	1	3.30	1	5.32	2.26	42.50	2.65	49.80
0	1	3.00	1	7.30	3.77	51.70	2.73	37.40
0	1	3.10	1	8.22	4.57	55.60	2.83	34.40
0	1	2.75	1	7.39	4.02	54.40	2.46	33.30
0	1	3.00	1	3.90	1.28	32.80	2.20	56.40
0	1	3.40	1	9.44	6.55	69.40	2.31	24.50
0	1	2.50	1	5.92	2.86	48.30	2.23	37.70
0	1	3.10	1	12.62	9.38	74.30	2.22	17.60
0	1	3.50	1	6.91	3.57	51.60	2.75	39.80
0	0	3.10	1	3.80	2.05	54.00	1.37	36.10
0	1	2.60	1	7.55	3.90	51.60	2.84	37.60
0	1	2.80	1	4.55	1.04	22.80	2.72	59.80
0	0	3.00	1	5.62	1.59	28.30	3.38	60.10
0	1	2.65	1	5.98	3.38	56.60	2.22	37.10
0	0	2.50	1	3.78	1.36	35.90	2.05	54.30
0	1	3.20	1	7.37	3.92	53.20	2.28	30.90
0	1	3.20	1	3.65	1.16	31.80	2.13	58.40
0	1	2.90	1	5.98	2.36	39.40	2.98	49.80
0	1	3.00	1	6.10	4.12	67.50	1.57	25.80
0	1	3.60	1	4.80	1.99	41.50	2.44	50.80
1	0	2.00	1	4.53	1.28	28.20	2.87	63.40
0	1	3.90	1	6.42	3.45	53.70	2.64	41.10
0	1	3.00	1	4.41	1.79	40.60	2.05	46.50
0	1	3.00	1	4.66	1.85	39.70	2.30	49.40
0	1	2.85	1	4.73	2.06	43.60	2.42	51.20
0	1	2.88	1	6.87	4.44	64.60	1.98	28.80
0	1	3.00	1	9.00	4.64	51.60	3.70	41.10
0	1	2.95	1	6.05	3.75	62.00	1.66	27.40
0	1	3.10	1	5.42	2.38	43.90	2.54	46.80
0	1	3.00	1	6.13	2.31	37.70	3.39	55.30
0	0	3.00	1	6.57	3.26	49.60	2.54	38.60

0	1	3.15	1	8.60	4.18	48.60	3.49	40.60
0	1	3.25	1	7.05	5.24	74.30	1.12	15.90
0	1	3.30	1	5.02	2.13	42.40	2.44	48.60
0	1	2.80	1	3.50	2.25	64.20	0.95	27.10
0	1	2.80	1	10.19	7.31	71.70	1.93	18.90
0	1	2.50	1	18.68	12.29	65.80	4.97	26.60
0	1	3.20	1	4.54	2.87	63.20	1.31	28.90
0	1	3.58	1	6.71	2.80	41.80	3.27	48.70
0	1	3.75	1	5.21	2.92	56.00	1.75	33.50
0	1	2.65	1	5.77	3.60	62.40	1.61	27.90
0	1	2.90	1	4.50	2.52	56.00	1.44	32.00
0	1	3.00	1	11.64	10.21	87.70	0.86	7.40
0	1	3.30	1	20.49	2.19	10.70	4.92	24.00
0	1	3.20	1	4.86	1.73	35.60	2.62	53.90
0	1	2.95	1	20.49	17.62	86.00	1.48	7.20
0	1	2.85	1	5.88	3.41	58.00	1.80	30.60
0	1	3.10	1	4.20	2.48	59.00	1.22	29.00
0	1	3.40	1	6.67	3.80	57.00	2.33	35.00
0	1	3.10	1	6.52	3.40	52.10	2.49	38.20
0	1	3.00	1	5.13	2.36	46.00	2.12	41.30
0	1	3.00	1	3.50	2.14	61.00	0.84	24.00
0	1	3.50	1	14.40	7.78	54.00	4.46	31.00
0	1	2.85	1	6.20	3.41	55.00	2.24	36.20
0	1	2.69	1	19.67	18.02	91.60	0.85	4.30
1	1	3.30	1	4.29	2.22	51.80	1.40	32.60
0	1	3.30	1	8.00	4.09	51.10	3.19	39.90
0	1	2.90	1	5.40	2.44	45.10	2.23	41.30
0	1	2.75	1	7.39	4.43	60.00	2.12	28.70
0	1	2.10	1	5.24	2.52	48.10	2.26	43.10
0	1	2.50	1	2.91	1.81	62.20	0.78	26.80
0	1	3.00	1	8.48	6.35	74.90	1.50	17.70
0	0	3.30	1	12.30	9.10	74.00	1.59	12.90
0	1	3.20	1	5.93	3.23	54.40	2.20	37.10
0	1	2.90	1	2.54	1.38	54.50	0.95	37.50
0	1	2.69	1	12.39	9.23	74.50	1.73	14.00
0	1	2.90	1	4.97	3.03	61.00	1.38	27.80
0	1	2.80	1	3.97	1.50	37.80	2.03	51.10
0	1	3.50	1	10.50	9.37	89.20	0.66	6.30
0	0	3.60	1	10.63	9.12	85.80	1.00	9.40
0	1	3.60	1	7.18	4.67	65.10	1.36	18.90
0	1	3.20	1	4.04	1.66	41.20	2.00	49.50
0	1	3.70	1	5.90	2.97	50.40	2.52	42.70
0	1	3.00	1	5.88	3.16	53.80	2.01	34.20
0	1	2.90	1	7.14	3.78	53.00	2.46	34.50
0	1	3.00	1	9.12	6.13	67.20	2.40	26.30
0	0	2.70	1	6.41	4.35	67.80	1.29	20.20
0	1	3.00	1	5.22	2.05	39.30	2.60	49.80
0	1	3.50	1	5.94	2.16	36.40	3.10	52.20
0	1	3.50	1	5.39	2.44	45.20	2.46	45.60
0	1	3.30	1	6.38	3.03	47.50	2.86	44.80
0	1	3.00	1	5.22	2.80	53.60	1.94	37.20
0	1	2.75	1	4.19	2.32	55.30	1.62	38.70
0	1	3.50	1	2.73	1.19	43.50	1.29	47.30
0	1	3.20	1	6.26	3.12	49.90	2.68	42.80

0	1	3.00	1	5.73	1.68	29.30	3.45	60.20
0	1	3.30	1	7.86	5.09	64.70	1.97	25.10
0	1	3.00	1	6.85	2.65	38.70	3.57	52.10
0	1	3.11	1	5.20	2.98	57.40	1.66	31.90
0	1	2.40	1	4.74	2.41	50.90	1.76	37.10
0	1	2.50	1	9.26	5.37	58.00	2.76	29.80
0	0	3.35	1	7.97	5.84	73.30	1.92	24.10
0	1	3.00	1	7.06	4.09	58.00	2.22	31.40
0	1	3.70	1	3.62	1.65	45.60	1.67	46.10
0	1	3.20	1	4.32	2.12	49.10	1.74	40.30
0	1	3.10	1	4.10	2.17	53.00	1.44	35.00
0	1	3.00	1	3.61	1.94	53.70	1.33	36.80
0	0	3.65	1	4.20	2.18	52.00	1.51	36.00
0	0	3.60	1	4.60	1.96	42.60	2.31	50.20
0	1	3.42	1	6.82	4.61	67.60	1.36	19.90
0	1	5.00	1	4.35	1.92	44.10	2.09	48.00
0	1	3.10	1	7.12	3.65	51.30	3.07	43.10
0	1	2.80	1	9.04	5.79	64.00	2.55	28.20
0	0	3.20	1	7.31	3.88	53.10	2.87	39.30
0	1	2.85	1	5.15	2.30	44.70	2.49	48.30
0	1	2.90	1	9.19	5.93	64.50	2.46	26.80
0	1	2.70	1	6.02	3.43	57.00	2.11	35.00
0	1	3.30	1	4.37	2.10	48.00	2.03	46.50
0	1	3.20	1	5.15	2.85	55.40	1.74	33.80
0	1	2.60	1	6.44	3.63	56.30	2.13	33.10
0	1	3.90	1	6.69	4.02	60.10	1.81	27.10
0	1	3.30	1	6.20	3.52	56.70	2.00	32.30
0	1	3.00	1	5.02	1.86	37.00	2.76	55.00
0	1	3.01	1	4.22	1.25	29.70	2.61	61.80
0	1	3.50	1	10.83	8.62	79.60	1.56	14.40
0	0	2.90	1	10.05	8.59	85.50	0.73	7.30
0	1	2.95	1	5.37	1.80	33.50	3.13	58.30
0	1	3.15	1	6.35	3.44	54.10	2.42	38.10
0	1	3.50	1	3.90	1.44	36.80	2.05	52.60
0	1	3.51	1	5.05	1.93	38.20	2.03	40.20
0	0	2.80	1	4.82	2.73	56.60	1.71	35.50
0	0	2.70	1	8.49	2.73	32.10	5.02	59.10
0	1	2.80	1	4.45	2.03	45.60	1.93	43.40
0	1	3.50	1	4.25	1.78	41.90	2.08	48.90
0	1	3.60	1	6.28	2.75	43.80	2.89	46.00
0	0	3.05	1	8.57	4.51	52.60	3.49	40.70
0	1	3.00	1	9.47	7.10	75.00	1.41	14.90
0	0	3.15	1	6.41	2.90	45.20	2.33	36.30
0	1	3.20	1	2.61	1.57	60.20	0.83	31.80
0	1	3.10	1	13.79	9.49	68.80	3.34	24.20
0	1	2.90	1	7.55	4.14	54.90	2.69	35.60
0	1	2.60	1	4.59	1.78	38.80	2.27	49.50
0	1	4.00	1	5.39	2.72	50.40	1.96	36.40
0	1	3.45	1	5.52	3.12	56.50	1.81	32.80
0	1	3.50	1	4.01	1.72	42.90	1.78	44.40
0	1	2.90	1	4.46	1.75	39.20	2.35	52.70
0	0	3.00	1	6.23	2.80	44.90	2.92	46.90
0	1	5.00	1	8.22	4.19	51.00	3.54	43.10
0	0	2.00	0	7.20	2.47	34.30	3.51	48.80

0	0	2.70	1	7.68	4.25	55.40	3.13	40.80
0	0	5.40	1	8.54	6.77	79.30	1.04	12.20
0	1	3.40	1	3.84	1.54	40.10	1.88	49.00
0	1	3.70	1	4.72	1.98	42.00	2.27	48.10
0	0	3.00	1	7.43	4.67	62.80	2.31	31.10
0	1	3.50	1	5.73	2.48	43.30	2.90	50.60
0	0	4.00	1	4.70	2.41	51.30	1.66	35.30
0	1	3.40	1	6.58	3.63	55.20	2.18	33.10
0	0	3.10	0	4.82	1.99	41.30	2.25	46.70
0	1	2.40	1	6.98	5.42	77.70	1.35	19.30
0	1	99.00	1	4.57	2.14	46.90	1.99	43.50
0	1	99.00	1	5.00	2.67	53.40	1.92	38.40
0	1	2.70	1	6.61	2.93	44.30	3.10	46.90
1	1	3.20	1	9.06	6.29	69.40	2.21	24.40
0	1	3.95	1	6.61	3.12	47.20	3.00	45.40
0	0	3.50	0	4.76	2.37	49.70	1.95	41.00
0	3	99.00	3	7.20	3.97	55.00	2.61	36.30
0	1	3.10	1	13.14	11.80	88.10	0.70	5.20
0	1	3.05	1	7.25	3.66	50.50	2.86	39.40
0	3	99.00	3	10.09	8.57	84.90	1.00	9.90
0	1	2.60	1	7.01	2.51	35.70	4.05	57.80
0	1	3.80	1	5.85	2.73	46.60	2.42	41.40
0	1	2.60	1	8.87	3.40	37.80	5.00	56.50
0	1	3.25	1	6.38	3.00	47.40	2.90	45.00
0	1	3.10	0	4.58	2.10	45.00	2.10	46.70
0	1	3.30	1	5.70	1.80	31.80	3.30	31.80
0	3	99.00	3	18.88	15.60	82.30	2.10	11.00
1	1	2.70	1	6.34	3.00	47.20	2.60	41.30
0	0	3.30	1	7.51	2.70	36.00	3.80	50.50
0	1	99.00	1	5.62	2.70	48.50	2.40	42.20
0	1	99.00	1	7.05	5.50	78.60	1.30	18.20
0	1	3.10	0	11.83	10.54	89.10	0.89	7.50
0	1	3.00	1	4.71	2.68	56.90	1.48	31.40
0	1	3.00	1	19.15	15.87	82.80	1.87	9.80
0	1	2.50	0	6.60	3.01	45.60	2.77	42.00
0	0	3.40	0	#NULL!	#NULL!	#NULL!	#NULL!	#NULL!
0	1	3.00	1	4.61	2.38	51.60	1.84	39.90
0	1	2.90	1	5.30	3.10	58.50	1.73	32.60
0	1	3.25	0	5.09	2.05	40.20	2.52	49.50
0	1	3.20	1	5.96	3.35	56.10	2.00	33.60
0	1	3.30	1	9.20	6.21	67.50	2.21	24.00
0	0	2.50	0	6.96	3.37	48.50	2.62	37.60
0	1	3.10	1	4.74	2.59	54.70	1.38	29.10
0	1	2.75	1	4.83	2.03	42.10	2.29	47.40
0	1	3.20	0	4.79	2.01	42.10	2.33	48.60
0	1	3.80	1	4.44	2.11	47.50	1.83	41.20
0	1	3.10	1	5.01	2.00	39.90	2.61	52.10
0	0	2.23	1	4.54	1.83	40.30	2.12	46.70
0	1	2.80	1	7.44	3.65	49.10	2.60	34.90
0	1	3.50	1	5.68	2.85	50.20	2.28	40.10
0	1	99.00	1	7.05	3.20	45.40	3.25	46.10
0	0	2.30	0	23.35	20.75	88.90	1.36	5.80
0	1	3.20	1	3.47	1.30	37.50	1.78	51.30
0	0	3.50	1	10.00	3.10	31.00	5.73	57.30

0	3	99.00	3	5.89	3.15	53.40	2.23	37.90
0	0	3.50	0	8.57	7.39	86.30	0.86	10.00
0	3	99.00	3	6.65	2.48	37.20	3.41	51.30
0	0	3.80	1	4.93	2.19	44.50	2.25	45.60
0	1	3.50	1	3.93	1.83	46.50	1.80	45.80
0	1	3.30	1	5.86	2.99	51.00	2.00	34.10
0	1	3.50	1	5.21	2.55	49.00	1.97	37.80
0	1	3.20	1	6.57	2.89	43.90	2.93	44.60
1	1	3.50	1	7.26	3.76	51.80	2.95	40.60
0	1	99.00	1	4.33	2.19	50.50	1.51	34.90
0	1	99.00	1	5.40	3.35	62.00	1.49	27.60
0	0	2.90	1	6.78	3.84	56.60	2.26	33.30
0	1	2.40	1	5.46	3.02	55.30	1.76	32.20
0	1	3.00	1	5.86	2.11	36.00	2.99	51.00
0	1	3.20	1	5.79	2.33	40.30	2.45	42.30
0	1	3.90	1	6.02	3.22	53.50	2.27	37.70
0	1	2.80	1	4.95	2.16	43.70	2.19	44.20
0	1	99.00	1	4.48	2.67	59.50	1.29	28.80
0	1	3.10	1	5.25	3.03	57.70	1.46	27.80
0	1	2.70	1	6.05	2.42	40.00	2.92	48.30
0	1	3.12	1	6.08	1.99	32.60	3.21	52.80
0	1	3.00	1	6.60	4.17	63.20	1.71	25.90
0	0	3.00	1	6.54	1.71	26.20	4.37	66.80
0	1	99.00	1	18.70	15.67	83.80	1.85	9.90
0	1	3.00	1	4.01	1.34	33.50	2.32	57.90
0	3	99.00	3	6.24	3.85	61.70	1.90	30.40
0	1	99.00	1	7.05	2.83	40.20	3.57	50.60
0	1	3.90	1	5.26	2.52	47.90	2.07	39.40
0	3	99.00	3	12.19	8.34	68.50	2.65	21.70
0	0	3.20	1	11.85	6.16	52.00	4.94	41.70
0	1	2.90	1	8.37	3.44	41.00	3.98	47.60
0	1	3.10	1	3.74	2.09	55.90	1.25	33.40
0	1	3.50	1	4.91	2.09	42.60	2.22	45.20
0	1	3.01	1	7.61	4.90	64.50	2.07	27.20
0	1	2.50	1	5.34	1.94	36.30	2.98	55.80
0	0	3.00	1	5.94	2.67	45.00	2.28	38.40
0	0	3.60	1	4.39	2.30	52.50	1.60	36.40
0	1	3.30	1	4.68	2.02	43.20	2.30	49.10
0	1	2.60	0	4.21	1.81	42.90	2.16	51.30
0	3	99.00	3	17.68	14.43	81.60	2.19	12.40
0	1	2.50	1	5.42	1.96	36.20	2.75	50.70
0	1	3.50	1	4.26	1.67	39.20	2.12	49.80
0	1	3.00	1	5.14	2.88	55.90	1.88	36.60
0	1	3.50	1	6.92	2.31	33.40	3.92	56.60
0	0	99.00	3	8.09	5.76	71.30	1.92	23.70
0	1	3.80	1	7.94	5.44	68.40	1.84	23.20
0	1	3.21	1	22.89	20.66	90.20	1.12	4.90
0	1	3.75	1	1.20	8.15	68.10	3.19	26.60
0	1	99.00	1	5.65	2.31	40.90	2.83	50.10
0	1	3.10	1	7.71	4.60	59.60	2.50	32.40
0	1	99.00	1	5.54	3.23	58.20	1.76	31.80
0	1	3.00	1	6.77	3.32	49.10	2.84	41.90
0	1	3.20	1	3.18	1.21	38.10	1.64	51.80
0	1	3.00	1	5.32	2.69	50.50	2.07	38.90

1	0	3.30	1	4.31	2.61	60.60	1.54	35.70
0	3	99.00	3	4.36	1.79	41.10	2.23	51.10
0	1	99.00	1	6.28	2.80	44.60	2.91	46.30
0	1	3.50	1	7.74	2.80	36.10	4.06	52.50
0	0	2.60	0	6.77	2.66	39.30	3.29	48.60
0	3	99.00	3	4.72	2.37	50.30	1.81	38.30
1	1	3.50	1	9.06	6.87	75.80	1.63	18.00
0	1	3.80	1	10.19	6.38	62.60	3.10	30.40
0	3	99.00	3	11.72	9.35	79.70	1.38	11.80
0	3	99.00	3	4.33	1.82	42.00	2.09	48.30
0	1	3.60	1	3.77	1.67	44.30	1.49	39.50
0	1	99.00	1	8.93	5.78	64.80	2.64	29.60
0	1	99.00	1	4.76	2.44	51.20	1.68	35.30
0	0	4.30	0	10.57	7.81	73.90	2.44	23.10
0	1	99.00	1	8.09	2.99	37.00	4.50	55.60
0	1	3.30	1	7.67	5.57	72.50	1.28	16.70
0	1	3.50	1	6.45	2.98	46.20	2.66	41.20
0	1	3.90	1	5.20	1.81	34.80	2.92	56.20
0	1	2.95	1	8.01	5.15	64.40	2.15	26.80
0	1	2.85	1	5.45	2.08	38.20	2.59	47.50
0	1	3.00	0	5.57	3.12	56.10	2.09	37.50
0	0	3.00	1	8.51	6.17	72.50	1.92	22.60
0	1	3.10	1	7.12	2.90	40.70	3.58	50.30
0	0	3.80	1	6.28	3.06	48.80	2.81	44.70
0	0	3.05	1	4.87	2.23	45.90	2.33	47.80
0	1	3.00	1	7.08	2.96	41.90	3.38	47.70
0	1	3.50	1	6.49	3.06	47.20	2.59	39.90
0	1	99.00	1	5.94	2.45	41.20	2.81	47.30
0	1	3.00	1	5.28	2.42	45.80	2.06	39.00
0	1	3.00	1	6.08	3.55	58.40	2.07	34.00
0	1	3.20	1	6.19	2.86	46.10	2.92	47.20
0	1	3.50	0	9.50	2.72	28.70	5.79	60.90
0	1	3.40	1	4.01	1.90	47.40	1.74	43.40
0	3	99.00	3	5.39	1.63	30.30	2.55	47.30
0	1	99.00	1	9.20	4.89	53.10	3.11	33.80
0	3	99.00	3	7.43	3.72	50.00	3.06	41.20
0	1	2.80	1	6.76	3.78	55.90	2.31	34.20
0	0	3.00	1	9.47	5.46	57.60	2.45	25.90
0	1	99.00	1	6.99	3.95	56.50	2.36	33.80
0	1	99.00	1	5.89	2.74	46.50	2.53	43.00
0	0	6.60	1	7.99	5.12	64.00	2.21	27.70
0	0	2.50	1	5.46	1.33	24.30	3.62	66.30
0	1	2.60	1	4.34	0.94	21.60	2.78	64.10
0	1	3.70	1	6.78	3.25	47.90	2.94	43.40
0	1	99.00	1	6.33	3.58	56.60	1.99	31.40
0	3	99.00	3	6.52	3.80	58.20	2.39	36.70
0	1	3.00	1	6.16	2.49	40.50	2.83	45.90
0	3	99.00	3	4.75	2.53	53.30	1.60	33.70
0	1	2.80	1	4.69	2.45	52.20	1.66	35.40
0	1	3.00	1	5.01	2.22	44.30	2.46	49.10
0	1	3.10	1	5.45	2.68	49.20	2.01	36.90
0	1	99.00	1	5.26	1.92	36.40	2.65	50.40
0	1	99.00	1	5.10	3.53	69.20	1.19	23.30
1	1	3.20	1	7.63	5.05	66.10	2.05	26.90

0	1	3.00	1	#NULL!	#NULL!	#NULL!	#NULL!	#NULL!
0	0	3.50	1	6.64	2.75	41.40	3.32	50.00
0	0	2.75	1	5.22	1.70	32.50	2.88	55.20
0	1	3.35	1	7.28	3.85	52.90	2.87	39.40
0	1	2.55	1	6.59	3.38	51.20	2.66	40.40
0	1	3.73	1	6.44	4.10	63.60	1.70	26.40
0	1	3.00	1	6.11	4.21	69.00	1.45	23.70
0	1	2.60	1	7.67	2.98	38.90	2.92	38.10
1	3	99.00	3	2.14	1.10	51.30	0.61	28.50
0	3	99.00	3	9.90	6.62	66.90	2.35	23.70
0	1	3.15	1	4.12	2.10	51.00	1.66	40.30
0	0	3.30	1	7.49	4.05	54.10	2.86	38.20
0	0	2.50	1	4.25	1.38	32.50	2.47	58.10
0	0	3.50	1	7.89	2.73	34.60	4.48	56.80
0	0	2.80	1	9.59	5.34	55.60	3.40	35.50
0	1	2.80	1	3.45	0.95	27.50	1.91	55.40
0	1	2.75	1	7.72	2.98	38.70	4.04	52.30
0	1	3.50	1	6.19	3.22	52.00	2.45	39.60
0	1	2.80	1	7.80	3.08	39.50	4.09	52.40
0	1	3.20	1	7.37	4.41	59.90	2.18	29.60
0	1	2.00	1	7.95	4.19	52.70	3.21	40.40
0	3	99.00	3	5.90	2.81	47.60	2.36	40.00
0	1	2.90	1	6.25	3.16	50.60	2.14	34.20
0	1	2.95	1	12.19	7.96	65.40	3.49	28.60
0	1	3.30	1	11.36	8.42	74.10	2.33	20.50
0	1	99.00	1	8.42	5.60	66.60	1.93	22.90
0	0	3.50	1	7.40	3.30	44.60	3.14	42.40
0	0	3.20	1	8.13	2.74	33.70	4.49	55.20
0	1	3.40	0	11.60	3.44	29.60	4.16	35.90
0	1	99.00	1	3.76	1.44	38.20	2.01	53.50
0	3	99.00	3	4.61	2.48	53.80	1.80	39.00
0	3	99.00	3	4.96	2.75	55.40	1.58	31.90
0	1	3.20	1	9.28	5.19	55.90	3.44	37.10
0	1	99.00	1	9.40	4.76	50.60	3.42	36.40
0	3	99.00	3	7.02	4.36	62.10	2.19	31.20
0	1	99.00	1	6.58	2.90	44.10	2.93	44.50
0	1	99.00	1	5.32	2.73	51.20	2.17	40.80
0	1	3.20	1	4.65	2.41	51.80	1.71	36.80
0	1	3.40	1	4.96	1.93	38.90	2.43	49.00
0	0	3.04	1	5.27	1.55	29.50	3.39	64.30
1	1	3.00	1	13.75	9.37	68.20	3.11	22.60
0	1	3.50	0	6.06	2.83	44.70	2.71	44.70
0	1	3.00	1	5.62	2.54	45.20	2.41	42.90
0	1	2.60	1	6.39	3.84	60.10	2.06	32.20
0	1	3.10	1	5.00	2.17	43.40	2.23	44.60
0	1	3.20	1	5.83	2.64	45.40	2.41	41.30
0	0	3.30	0	4.97	1.30	26.20	3.23	65.00
0	1	3.05	1	3.98	1.96	49.20	1.27	31.90
0	1	3.75	1	6.50	3.52	54.00	2.37	36.50
0	1	3.00	1	9.39	6.39	68.10	2.47	26.30
0	1	3.00	1	8.84	6.13	69.30	1.89	21.40
0	1	2.90	1	5.32	2.26	42.40	2.69	50.60
0	0	3.80	1	10.00	6.28	62.80	2.89	28.90
0	1	3.00	1	10.20	4.66	45.70	4.97	48.70

0	0	3.40	1	5.45	2.88	52.80	2.05	37.60
0	3	99.00	3	4.28	1.80	42.00	2.10	49.10
0	1	3.40	1	6.81	2.05	30.10	3.86	56.70
0	1	99.00	1	6.61	2.97	44.90	3.09	46.70
0	0	3.80	1	4.70	1.68	35.70	2.39	50.90
0	1	3.00	1	3.99	1.71	42.80	1.82	45.60
0	1	3.30	1	8.87	4.56	51.30	3.34	37.70
0	3	99.00	0	5.13	2.54	49.50	2.10	40.90
0	1	3.30	1	4.72	1.99	42.10	2.24	47.50
0	1	99.00	1	5.43	2.49	45.80	2.61	48.10
0	1	99.00	1	6.85	3.00	43.80	3.19	46.60
0	0	3.50	1	6.28	3.07	48.90	2.79	44.40
0	0	3.60	1	5.24	2.15	41.00	2.61	49.80
0	1	3.10	1	5.59	2.73	37.20	2.08	37.20
0	1	3.40	1	5.35	2.44	45.60	1.96	36.60
0	1	99.00	1	6.30	4.39	69.60	1.32	21.00
0	1	99.00	1	4.19	1.86	44.30	1.82	43.40
0	1	3.10	1	6.38	4.41	69.10	1.49	69.10
0	1	99.00	1	7.87	3.00	38.10	3.66	46.50
0	1	3.00	1	5.09	#NULL!	38.90	#NULL!	51.70
0	0	3.10	0	7.24	2.99	41.40	3.30	45.60
0	1	2.60	1	7.22	3.62	50.10	2.77	38.40
0	1	3.35	0	6.09	2.11	34.70	3.13	51.40
0	0	3.60	1	6.32	3.10	49.10	2.25	35.60
0	1	2.40	0	3.73	1.49	40.00	1.67	44.80
0	1	3.00	1	13.36	9.06	67.80	3.19	23.90
0	1	2.70	1	2.96	1.49	50.30	1.18	39.90
0	1	2.70	1	6.71	3.30	49.20	2.83	42.20
0	0	2.00	1	6.85	4.52	66.00	1.89	27.60
1	1	3.20	1	7.47	4.61	61.80	2.14	28.50
0	1	99.00	1	6.97	3.17	45.40	3.19	45.80
0	1	3.05	0	4.17	2.08	49.90	1.72	41.20
0	0	3.20	1	6.45	3.20	49.60	2.21	34.30
0	1	3.30	1	8.33	3.09	37.10	4.43	53.20
0	1	99.00	1	8.47	4.48	52.90	3.45	40.70
0	0	2.75	1	12.45	8.43	67.70	2.93	23.50
0	1	2.55	1	7.85	3.08	39.10	4.22	54.80
0	1	3.20	1	5.68	3.09	54.40	2.08	36.60
0	1	3.38	0	8.58	3.74	43.70	4.33	50.50
0	1	2.60	1	5.34	2.75	51.40	2.02	37.80
0	1	4.00	0	6.52	2.87	44.00	3.11	47.70
0	1	99.00	0	2.31	1.30	56.20	0.85	36.80
0	3	99.00	3	4.27	1.96	45.80	1.63	38.20
0	1	99.00	1	7.67	3.70	48.30	2.78	36.20
0	1	99.00	0	9.42	7.17	76.20	1.38	14.60
0	0	3.50	1	6.74	3.21	47.60	2.91	43.20
0	1	3.50	1	6.51	2.72	41.70	2.84	43.60
0	3	99.00	3	6.82	3.07	45.00	2.95	43.30
0	3	99.00	3	6.32	3.88	61.40	1.90	30.10
0	1	3.60	1	29.00	24.99	86.10	2.23	7.70
0	1	99.00	1	10.27	8.91	86.70	0.95	9.30
0	1	99.00	1	6.46	2.37	36.70	3.54	54.80
0	0	3.10	1	7.08	4.55	64.30	2.00	28.20
0	1	3.30	1	9.33	5.45	58.40	3.37	36.10

1	3	99.00	3	7.66	3.36	43.80	3.79	49.50
0	1	99.00	1	5.65	3.15	55.70	2.04	36.10
0	0	2.60	1	11.09	7.23	65.30	3.03	27.30
0	1	3.35	1	33.30	9.24	27.70	2.20	6.60
0	1	3.05	1	9.73	2.76	28.40	5.57	57.20
0	1	3.40	1	8.44	2.32	27.40	5.24	62.10
0	1	3.70	1	10.65	4.59	43.20	4.85	45.50
0	1	99.00	1	8.84	5.55	62.70	2.58	29.20
0	3	99.00	3	7.47	3.38	45.20	3.21	43.00
0	0	3.25	1	7.03	3.45	49.00	2.60	37.00
0	1	99.00	1	5.84	2.39	40.90	2.76	47.30
0	1	99.00	0	5.16	2.06	39.80	2.30	44.60
0	0	3.00	1	6.67	#NULL!	45.90	#NULL!	40.20
0	1	3.20	1	7.51	3.61	48.20	3.24	43.10
0	1	3.30	1	10.52	5.50	52.20	4.23	40.20
0	1	99.00	1	7.58	5.24	69.10	1.82	24.00
0	1	99.00	1	10.88	7.63	70.10	2.52	23.20
0	1	99.00	1	9.98	2.74	27.50	6.83	68.40
0	1	3.60	1	9.04	4.56	50.50	3.72	41.20
0	1	99.00	1	6.95	5.52	79.50	1.12	16.10
0	1	99.00	1	5.72	2.42	42.30	2.68	46.90
0	1	4.00	1	8.82	4.65	52.60	3.56	40.40
0	3	99.00	3	8.81	6.79	77.10	1.76	20.00
0	3	99.00	3	8.36	5.53	66.00	2.27	27.20
1	1	3.60	1	6.65	3.92	58.90	2.01	30.20
0	1	3.00	1	3.77	1.27	33.60	2.17	57.60
0	0	2.90	1	4.18	2.33	55.80	1.33	31.80
0	0	3.70	0	7.46	2.78	37.30	3.74	50.10
0	1	99.00	1	7.91	4.93	62.30	2.31	29.20
0	1	3.00	1	6.78	2.24	33.00	3.89	57.40
0	3	99.00	3	5.42	2.81	51.80	2.18	40.20
0	0	99.00	1	6.17	1.72	27.90	3.89	63.00
0	3	99.00	3	5.13	2.27	44.20	2.34	45.60
0	1	3.45	1	6.60	4.17	63.10	1.66	25.20
0	1	3.45	1	5.91	2.75	46.60	2.60	44.00
0	1	99.00	1	11.18	4.70	42.00	5.84	52.20
0	0	2.80	1	3.84	1.99	51.80	1.52	39.60
0	1	2.90	0	3.23	1.26	39.00	0.93	28.80
0	0	99.00	1	4.66	2.04	43.80	2.25	48.30
0	3	99.00	3	6.55	3.27	49.90	2.81	42.90
0	1	2.80	1	6.18	2.42	39.20	3.16	51.10
0	3	99.00	3	25.08	22.60	90.10	1.31	5.20
0	1	3.50	0	5.07	2.24	44.20	2.49	49.10
0	1	3.00	1	10.50	6.19	59.00	3.26	31.00
0	1	3.10	1	5.16	2.17	42.00	2.65	51.40
0	1	99.00	1	8.17	2.65	32.40	4.64	56.80
0	1	2.74	1	9.50	4.83	50.80	3.92	41.30
0	1	3.20	1	6.25	2.59	41.40	3.15	50.40
0	1	99.00	1	4.49	1.48	33.00	2.48	55.20
0	3	99.00	3	0.40	0.01	2.70	0.36	97.30
0	1	99.00	1	5.25	2.82	53.60	2.15	41.00
0	1	3.10	1	7.29	3.76	51.60	3.02	41.40
0	1	3.10	1	5.63	1.95	34.60	3.30	58.60
0	1	3.50	1	5.30	1.85	34.90	2.89	54.50

0	1	99.00	1	6.00	2.57	42.90	2.84	47.30
1	1	3.20	1	7.83	4.49	57.40	2.70	34.50
0	1	99.00	1	8.39	4.06	48.40	3.76	44.80
0	1	3.00	1	6.57	3.70	56.30	2.35	35.80
0	1	3.50	1	5.46	2.03	37.20	2.50	45.80
0	0	3.80	1	6.20	2.66	43.00	2.70	43.50
0	1	3.10	1	9.90	4.05	41.00	5.24	52.90
0	1	3.50	0	7.26	3.63	50.00	3.20	44.10
0	0	2.55	1	6.24	2.53	40.50	2.87	46.00
0	0	3.20	1	7.78	4.03	51.70	2.97	38.20
0	1	3.45	1	7.65	5.46	71.30	1.65	21.60
0	0	3.80	1	5.75	2.99	52.00	2.26	39.30
0	1	3.00	1	7.12	4.24	59.60	1.75	24.60
0	1	3.30	1	6.02	2.76	45.90	2.73	45.30
0	0	3.50	1	7.00	3.90	55.70	2.56	36.60
0	0	3.55	1	7.38	3.07	41.60	3.53	47.80
0	1	4.60	1	8.62	4.86	56.40	2.88	33.40
0	0	2.40	1	10.00	4.60	46.00	4.77	47.70
0	0	3.50	0	14.51	10.20	70.30	3.26	22.50
0	1	99.00	1	6.20	2.56	41.30	2.81	45.30
0	1	99.00	1	5.52	2.51	45.50	2.61	47.30
0	1	3.80	1	7.05	2.24	31.70	4.39	62.30
0	1	2.86	1	8.15	4.59	56.30	2.59	31.80
0	1	3.20	0	6.30	3.05	47.90	2.92	45.80
0	1	99.00	1	5.76	3.26	56.70	2.12	36.80
0	3	99.00	3	5.46	2.48	45.40	2.51	46.00
0	1	2.60	1	8.55	3.98	46.50	3.65	42.70
0	1	99.00	1	9.41	6.61	70.30	1.96	20.80
0	1	3.20	0	8.28	3.53	42.70	3.29	39.70
0	0	3.00	1	7.63	3.82	50.00	3.12	40.90
0	1	2.80	1	4.81	2.69	55.90	1.60	33.30
0	0	4.20	1	8.67	3.32	38.40	4.59	52.90
0	0	3.30	1	6.43	3.13	48.70	2.97	46.20
0	1	3.40	1	#NULL!	#NULL!	#NULL!	#NULL!	#NULL!
0	1	2.70	1	4.66	2.22	47.70	1.95	41.80
0	0	99.00	1	8.05	4.15	51.50	3.08	38.30
0	1	2.20	1	14.28	10.53	73.70	2.74	19.20
0	1	3.30	1	9.77	5.83	59.70	3.23	33.10
0	0	4.40	1	7.18	#NULL!	57.40	#NULL!	36.60
0	3	99.00	3	8.23	4.72	57.40	2.70	32.80
0	1	2.90	1	10.61	6.23	58.70	3.30	31.10
0	0	99.00	1	6.71	3.24	48.30	2.73	40.70
0	0	3.20	1	6.47	3.08	47.60	2.75	42.50
0	3	99.00	3	5.57	3.14	56.40	1.98	35.50
0	1	6.00	0	6.66	2.59	38.80	3.54	53.20
0	1	99.00	1	7.83	4.08	52.10	3.00	38.30
0	0	3.40	1	12.88	5.36	41.60	6.47	50.20
0	1	3.50	1	12.47	8.20	65.80	3.56	28.50
0	3	99.00	3	7.48	4.64	62.00	2.34	31.30
0	0	2.80	1	5.45	2.35	43.10	2.55	46.80
0	1	3.35	1	4.49	2.04	45.50	2.07	46.10
0	1	2.80	1	10.89	7.53	69.10	2.87	26.40
0	1	99.00	1	5.95	3.22	54.10	2.30	38.70
0	1	3.50	1	6.88	3.86	56.10	2.53	36.80

0	1	3.00	1	5.93	2.02	48.30	2.67	48.30
0	1	2.80	1	#NULL!	#NULL!	#NULL!	#NULL!	#NULL!
0	1	3.50	1	8.52	3.45	40.50	4.39	51.50
0	1	3.10	1	7.08	4.25	60.10	2.49	35.20
0	1	2.80	1	5.64	2.60	46.10	2.70	47.90
0	1	3.40	1	4.36	2.29	52.60	1.67	38.30
0	1	2.30	1	6.81	3.21	47.10	3.11	45.70
0	3	99.00	3	7.86	4.62	58.70	2.70	34.40
0	1	2.95	0	5.90	2.35	39.80	2.91	49.30
0	1	99.00	1	3.50	0.82	23.40	1.19	34.00
0	1	99.00	1	6.24	2.78	44.60	2.79	44.70
0	0	99.00	1	5.82	2.22	38.10	2.82	48.50
0	1	2.60	0	5.83	3.30	56.70	2.02	34.60
0	0	4.50	1	6.25	2.64	42.20	2.35	37.60
0	1	99.00	1	7.62	3.19	41.90	3.47	45.50
0	0	2.70	1	5.15	2.55	49.60	2.02	39.20
0	3	99.00	3	6.53	3.82	58.40	2.08	31.90
0	0	3.20	1	5.34	2.59	48.50	2.21	41.40
0	1	3.00	1	5.61	2.89	51.50	2.14	38.10
0	1	4.25	1	5.35	2.31	43.20	2.64	49.30
0	1	99.00	1	3.98	1.88	47.20	1.68	42.20
0	0	3.25	1	9.63	5.82	60.50	2.94	30.50
0	1	3.20	1	3.51	1.36	38.80	1.81	51.60
0	1	3.00	1	3.40	1.10	31.40	2.00	58.20
0	1	99.00	1	4.77	2.06	43.10	1.63	34.20
0	0	3.50	1	7.09	4.50	63.50	2.13	30.00
0	1	99.00	1	#NULL!	#NULL!	#NULL!	#NULL!	#NULL!
0	1	3.10	1	6.77	2.18	32.20	4.31	63.70
0	1	99.00	0	8.16	4.27	52.30	2.87	35.20
0	1	3.07	1	4.36	2.18	50.00	1.71	39.20
0	1	3.00	1	6.45	2.14	33.20	3.64	56.40
0	1	99.00	0	4.60	2.13	46.20	2.00	43.50
0	1	2.80	1	6.58	2.56	38.90	3.03	46.00
0	1	2.60	1	9.28	5.81	62.60	2.68	28.90
0	1	3.00	1	5.18	2.37	45.70	2.36	45.60
0	0	2.97	1	9.92	4.57	46.00	4.54	45.80
0	1	3.20	0	11.10	7.37	66.30	2.74	24.70
0	1	99.00	1	9.96	4.54	45.60	4.55	45.70
0	3	99.00	0	4.11	2.08	50.60	1.66	40.40
0	0	4.10	1	4.45	2.09	46.90	1.81	40.70
0	3	99.00	0	5.89	2.70	45.90	2.81	47.70
0	1	99.00	1	9.61	5.40	56.20	3.45	35.90
0	0	4.30	1	5.18	2.25	43.40	2.42	46.70
0	1	3.20	1	7.00	2.79	39.80	3.52	50.30
0	0	3.50	1	8.17	4.31	52.70	3.10	37.90
0	0	3.50	0	10.64	5.53	52.00	3.75	35.20
0	1	2.40	1	8.91	4.08	45.70	4.30	48.30
0	1	99.00	1	4.95	1.70	34.40	2.60	52.50
0	0	3.00	1	5.94	2.61	44.00	2.84	47.80
0	1	2.80	0	5.58	2.57	46.10	2.64	47.30
0	1	2.20	0	6.04	3.13	51.80	2.22	36.80
0	0	99.00	1	9.13	4.59	50.30	3.80	41.60
0	1	3.00	0	7.18	3.35	46.60	3.28	45.70
0	0	2.90	1	9.33	5.12	54.90	3.53	37.80

0	1	3.00	1	20.51	18.68	91.00	1.23	6.00
0	1	99.00	0	6.79	3.22	47.60	2.98	43.90
0	0	3.60	0	11.53	9.88	85.70	1.08	9.40
0	0	3.80	1	6.81	2.95	43.30	3.27	48.00
0	0	3.20	1	6.07	2.69	44.30	2.74	45.10
0	1	3.30	1	6.83	2.52	36.90	3.76	55.10
0	0	2.60	1	8.00	3.33	41.50	3.58	44.80
0	0	3.65	1	4.07	2.07	50.90	1.56	38.30
0	1	2.60	1	7.13	3.82	53.70	2.78	39.00
0	3	99.00	3	10.16	6.94	68.30	2.61	25.70
1	1	3.30	1	4.90	2.72	55.60	1.67	34.10
0	1	99.00	0	3.82	1.34	35.00	2.05	53.70
0	0	2.65	0	6.97	1.88	26.90	4.62	66.30
0	0	2.70	1	5.54	3.57	64.40	1.47	26.50
0	0	3.15	1	5.81	2.40	41.30	2.94	50.60
0	0	99.00	1	3.41	1.24	36.30	1.84	54.00
0	1	99.00	1	7.17	3.37	47.00	3.30	46.00
0	1	3.40	1	4.19	1.29	30.70	2.30	54.90
0	1	99.00	1	5.92	2.76	46.50	0.40	6.80
0	1	2.95	1	13.08	11.60	88.70	1.43	10.90
0	1	2.05	1	6.12	1.78	29.20	3.84	62.70
0	1	3.00	1	11.14	9.02	80.90	1.58	14.20
0	3	99.00	3	6.16	3.08	50.00	2.57	41.70
0	1	2.40	1	4.60	1.59	34.60	2.66	57.80
0	0	2.90	1	9.28	4.13	44.50	4.23	45.60
0	0	3.70	1	6.96	2.42	34.80	3.76	54.00
0	0	3.40	1	13.21	9.62	72.80	2.85	21.60
0	1	3.00	1	4.77	2.29	48.00	1.95	40.90
0	0	99.00	1	4.11	1.91	46.40	1.68	40.90
0	0	2.70	1	7.22	3.53	48.80	2.90	40.20
0	1	3.00	1	5.01	1.79	35.70	2.78	55.50
0	1	3.00	1	5.42	3.09	57.10	1.72	31.70
0	1	3.10	1	3.61	1.47	40.80	1.64	45.40
0	1	3.30	1	5.79	2.38	41.10	2.83	48.90
0	1	3.60	1	5.71	4.02	70.30	1.17	20.50
0	1	3.10	1	4.97	1.98	39.90	2.47	49.70
0	1	2.30	0	6.62	2.71	40.90	2.73	41.20
0	1	2.60	1	4.15	1.30	31.30	2.26	54.40
0	0	2.80	1	6.16	2.42	39.30	3.22	52.30
0	1	2.90	0	4.91	2.06	42.00	2.25	45.80
0	1	2.90	0	7.60	3.33	43.80	3.60	47.40
0	1	99.00	1	6.03	2.55	42.40	2.78	46.10
0	1	3.20	1	6.57	2.56	39.00	3.29	50.10
0	0	3.40	1	11.80	8.12	68.80	3.12	26.40
0	1	3.60	1	6.60	2.85	43.20	3.04	46.10
0	1	3.15	1	6.74	4.36	64.80	1.58	23.40
0	1	99.00	1	5.69	3.00	52.60	2.19	38.50
0	1	3.00	1	5.39	2.73	50.60	2.14	39.70
0	0	99.00	1	4.82	2.04	42.40	2.02	41.90
0	1	3.00	1	17.96	15.43	85.90	1.08	6.00
0	0	3.10	1	8.06	3.58	44.40	3.26	40.40
0	0	3.80	1	3.55	2.02	56.90	1.23	34.50
0	1	99.00	1	7.68	4.53	59.00	2.29	29.80
0	1	3.05	1	7.31	3.49	47.80	3.15	43.10

0	1	3.72	1	4.32	2.01	46.60	1.92	44.40
0	1	3.30	1	5.71	2.07	36.20	3.17	55.50
0	1	2.70	1	5.07	2.32	45.70	2.29	45.20
0	1	2.80	1	8.07	32.10	2.59	4.87	60.30
0	0	2.65	1	5.33	1.96	36.80	2.87	53.80
0	1	2.52	1	6.01	3.03	50.40	2.30	38.30
0	0	3.20	1	3.55	1.52	42.80	1.64	46.20
0	0	3.50	1	4.93	1.68	34.10	2.72	55.20
0	1	2.24	1	8.38	2.37	28.20	4.84	57.80
0	0	2.50	0	7.55	2.88	38.10	4.01	53.10
0	1	2.40	0	7.62	4.27	56.00	2.87	37.70
0	0	3.30	1	3.82	1.36	35.60	1.89	49.50
0	0	99.00	1	7.67	3.63	47.30	3.02	39.40
0	0	3.65	0	3.95	1.43	36.20	2.14	54.20
0	1	3.70	1	5.26	2.44	46.40	2.29	43.50
0	0	3.65	1	8.18	3.92	47.90	3.59	43.90
0	1	4.00	1	5.68	1.91	33.50	3.36	59.20
0	0	2.50	0	7.03	3.19	45.40	2.97	42.20
0	0	2.98	0	9.58	5.50	57.40	3.41	35.60
0	0	3.40	1	9.09	5.76	63.40	3.12	34.30
1	1	99.00	1	5.94	3.51	59.20	1.93	32.50
0	1	99.00	1	23.65	20.54	86.80	1.89	8.00
0	1	2.90	1	5.66	2.95	52.10	2.17	38.30
0	0	3.30	1	5.99	3.48	58.20	2.02	33.70
0	1	3.90	1	4.54	1.96	43.20	2.18	48.00
0	1	2.82	1	5.89	2.83	48.10	2.58	43.80
0	1	1.90	1	6.27	2.21	35.20	3.43	54.70
0	1	99.00	1	4.44	2.23	50.20	1.76	39.60
0	0	3.60	1	4.50	1.66	37.00	2.49	55.30
0	1	3.50	1	5.77	2.74	47.50	2.67	46.30
0	1	1.80	1	8.12	3.05	37.60	3.80	46.80
0	1	2.99	1	11.64	6.30	54.10	4.31	37.00
0	1	99.00	1	4.86	3.53	72.70	0.74	15.20
0	0	3.20	0	8.55	2.82	33.00	4.93	57.70
1	1	99.00	1	5.34	1.97	36.80	2.77	51.90
0	0	2.70	0	9.31	5.06	54.40	3.41	36.60
0	0	2.40	1	8.04	3.56	44.30	3.42	42.50
1	1	3.20	1	10.36	2.27	21.90	7.17	69.20
0	1	2.90	1	4.89	1.71	35.00	2.77	56.60
1	1	3.15	0	7.23	1.68	23.20	4.51	62.40
0	1	3.20	1	6.12	2.45	40.00	2.90	47.40
0	1	3.15	0	4.32	1.36	31.50	2.48	57.40
0	0	2.65	1	#NULL!	#NULL!	#NULL!	#NULL!	#NULL!
0	1	3.20	0	7.46	2.56	34.30	3.78	50.70
0	1	3.25	1	#NULL!	#NULL!	#NULL!	#NULL!	#NULL!
0	1	3.25	1	6.90	2.39	34.70	3.74	54.20
0	1	99.00	0	5.48	2.21	40.20	2.76	50.40
0	1	2.90	1	6.99	2.28	32.60	3.44	49.20
0	1	99.00	1	5.05	2.21	43.70	2.07	41.00
0	1	99.00	1	4.33	2.16	49.80	1.64	37.90
0	1	5.09	1	6.34	2.37	37.30	3.51	55.40
0	1	3.00	1	18.97	16.82	88.70	0.89	4.70
0	1	3.00	1	3.22	1.46	45.30	1.51	46.90
0	1	4.00	1	6.08	3.30	54.30	2.36	38.80

0	1	3.50	1	7.09	3.38	47.70	3.17	44.70
0	1	2.70	1	5.39	1.02	18.90	3.85	71.40
0	0	2.30	1	8.94	4.71	52.70	3.44	38.50
0	0	3.10	1	4.66	3.29	70.00	0.82	17.60
0	1	3.25	1	6.79	2.54	37.40	3.76	55.20
0	0	5.20	1	7.45	3.07	41.20	3.38	45.40
0	1	99.00	1	9.04	4.43	49.00	3.88	42.90
0	1	3.05	0	6.34	3.58	56.50	2.41	38.00
0	1	3.30	1	5.60	2.89	51.60	2.25	40.20
0	1	3.10	1	7.15	#NULL!	45.50	#NULL!	42.50
0	1	3.20	1	5.55	2.71	48.80	2.25	40.50
0	1	3.60	1	7.14	2.96	41.40	3.74	52.40
0	0	3.40	1	6.87	3.31	48.30	2.81	40.90
0	1	99.00	1	6.23	3.21	51.60	2.24	36.00
0	0	3.20	1	4.09	1.70	41.60	2.10	51.30
0	1	2.80	1	4.68	2.55	54.50	1.73	37.00
0	0	99.00	0	12.37	9.03	73.00	#NULL!	21.70
0	0	2.65	0	6.17	2.67	43.30	2.89	46.80
0	1	3.50	0	9.56	1.14	11.90	2.86	29.90
0	1	2.80	1	6.38	3.52	55.10	1.86	29.20
0	1	3.60	1	6.80	2.06	30.30	4.05	59.60
0	0	2.40	0	4.96	2.42	48.80	2.04	41.10
0	1	2.80	1	6.04	3.24	53.70	2.14	35.40
0	1	3.50	1	14.37	11.43	79.50	2.11	14.70
0	0	3.40	1	5.17	2.10	40.60	2.59	50.10
0	1	99.00	1	6.52	3.91	60.00	1.98	30.40
0	1	3.00	1	4.15	1.22	29.30	2.51	60.50
0	1	3.30	1	6.27	3.49	55.70	2.25	35.90
0	0	3.20	1	3.94	0.76	19.30	2.49	63.20
0	1	99.00	1	5.39	2.76	51.20	2.06	38.20
0	1	3.80	1	8.24	5.81	70.50	1.87	22.70
0	0	2.80	1	4.75	2.09	44.00	2.19	46.10
0	1	99.00	1	4.75	2.39	50.30	1.94	40.80
0	0	3.50	1	6.05	3.78	62.50	1.42	23.50
0	0	4.20	1	13.02	9.78	75.20	2.05	15.70
0	1	3.20	1	4.34	1.88	43.30	1.97	45.40
0	0	3.90	1	4.66	2.42	51.90	1.74	37.30
0	1	3.00	1	28.74	24.04	87.20	1.56	5.40
0	1	99.00	1	4.94	2.17	43.90	2.27	46.00
0	1	3.30	1	5.23	2.88	55.10	1.79	34.20
0	1	2.80	1	7.92	4.66	58.70	2.80	35.40
0	0	99.00	1	9.35	5.78	61.80	2.95	31.60
0	0	3.10	1	5.48	2.14	39.00	2.85	52.00
0	0	3.25	1	6.28	2.48	39.50	2.45	39.00
0	1	99.00	1	4.39	1.65	37.50	2.23	50.80
0	0	3.73	1	8.55	3.80	44.40	3.11	36.40
0	0	3.20	1	4.70	1.82	38.70	2.36	50.10
0	1	2.60	1	5.36	2.14	39.90	2.58	48.20
0	1	4.00	1	7.60	5.20	68.40	1.85	24.30
0	1	3.50	0	5.66	2.43	43.00	2.24	39.50
0	0	99.00	1	5.27	2.06	39.10	2.64	50.10
0	0	3.00	1	5.92	2.68	45.20	2.22	37.40
0	1	99.00	1	6.52	3.59	55.00	2.54	38.90
0	1	3.00	1	4.30	1.55	36.20	2.19	51.00

0	1	2.70	1	5.18	1.56	30.10	3.22	62.00
0	0	3.00	1	4.90	1.74	35.60	2.73	55.70
0	1	2.99	1	3.33	1.41	42.60	1.43	42.80
0	1	2.75	1	4.51	1.97	43.70	1.94	43.00
0	1	2.90	1	7.36	3.86	52.40	2.82	38.30
0	1	3.50	1	5.73	2.23	38.90	2.95	51.60
0	0	3.20	1	3.60	1.61	44.80	1.60	44.40
0	1	3.50	1	2.97	1.06	35.60	1.61	54.30
0	0	2.45	1	3.61	1.68	46.50	1.58	43.80
0	1	3.20	0	8.16	5.11	62.70	2.25	27.60
0	0	3.60	0	5.48	2.22	40.40	2.66	48.50
0	1	99.00	1	5.54	1.40	25.20	3.85	69.50
0	1	99.00	1	3.86	1.24	32.20	2.42	62.50
0	1	2.40	1	8.42	6.54	77.80	1.12	13.30
0	1	3.55	0	4.58	2.96	64.30	1.20	26.20
0	1	3.00	1	4.84	2.27	46.90	2.21	45.70
0	0	2.90	1	4.13	1.68	40.80	2.13	51.30
0	1	3.00	1	5.96	2.66	44.70	2.76	46.20
0	1	3.00	1	12.84	10.88	84.70	1.42	11.10
0	1	3.15	0	8.81	3.21	36.40	4.46	50.60
0	0	3.50	1	6.97	3.68	52.90	2.81	40.20
0	0	3.66	0	4.92	3.96	80.40	0.57	11.70
0	0	3.10	1	7.05	2.46	34.80	3.81	54.00
0	3	99.00	3	7.93	4.85	61.10	2.10	26.50
0	3	99.00	3	12.47	6.68	53.70	4.87	39.00
1	0	3.50	1	5.68	1.33	23.40	3.93	69.30
0	1	3.00	1	4.15	2.11	51.00	1.67	40.20
0	1	3.00	1	4.67	1.65	35.40	2.31	49.40
0	1	99.00	1	3.82	1.35	35.50	2.09	54.70
0	1	99.00	1	7.32	4.44	60.70	2.42	32.90
0	1	3.80	1	7.72	4.31	55.80	2.90	37.60
0	3	99.00	3	28.96	26.36	91.00	1.91	6.60
0	1	99.00	1	8.10	6.86	84.80	1.02	12.60
0	1	3.30	1	11.44	8.03	70.20	2.77	24.20
0	1	3.60	1	6.25	3.86	61.70	2.02	32.30
0	0	99.00	1	5.44	2.29	42.20	2.40	44.20
0	1	3.20	1	7.68	4.02	52.30	3.14	40.80
0	1	3.20	1	10.33	7.35	71.20	2.42	23.40
0	1	2.80	1	6.21	2.49	40.10	3.44	55.40
0	1	3.20	1	5.73	3.28	57.10	1.86	32.50
0	1	3.30	1	5.06	1.68	33.20	2.79	55.10
0	1	99.00	1	4.66	1.83	29.20	2.46	52.70
0	1	3.55	1	3.73	1.08	28.90	2.26	60.70
0	0	3.00	1	4.12	1.60	38.80	2.05	49.70
0	1	99.00	1	4.51	2.29	50.70	1.88	41.70
0	1	3.00	1	5.64	4.31	76.50	0.92	16.30
0	0	3.79	1	6.19	2.24	43.10	2.56	49.50
0	1	3.10	1	4.93	2.07	41.90	2.41	48.90
0	1	2.90	1	5.71	1.64	28.60	3.15	55.20
0	1	2.90	1	4.89	2.31	47.10	2.24	46.00
0	1	99.00	1	5.79	2.23	38.50	3.04	52.50
0	0	3.60	1	4.62	1.59	34.50	2.49	53.80
0	1	99.00	1	5.42	2.67	49.20	2.13	39.30
0	1	3.10	1	21.29	19.04	89.40	1.60	7.50

0	1	3.60	0	8.69	4.74	54.60	3.11	35.70
0	1	99.00	1	3.92	1.28	32.70	2.31	58.90
0	1	99.00	1	6.52	4.02	61.70	1.56	24.00
0	0	3.25	1	7.99	2.73	34.20	4.54	56.90
0	1	99.00	1	5.18	2.40	46.30	2.18	42.20
0	1	3.00	1	5.82	2.13	36.60	3.38	58.10
0	1	2.90	1	11.03	8.74	79.20	1.77	16.10
0	1	3.00	1	5.65	2.37	42.00	2.74	48.50
0	3	99.00	3	6.91	4.52	65.50	1.93	27.90
0	1	4.10	1	4.66	2.00	42.90	2.18	46.80
0	1	1.70	1	4.03	1.64	40.70	2.04	50.60
0	1	99.00	1	5.62	2.33	41.40	2.70	48.20
0	1	3.00	1	10.65	7.69	72.20	2.08	19.50
0	1	3.00	1	5.48	2.79	50.90	2.09	38.10
0	0	99.00	1	5.69	2.05	36.20	3.20	56.20
0	1	3.50	1	2.83	#NULL!	52.50	#NULL!	35.10
0	1	3.00	1	5.23	2.55	48.70	2.04	39.10
0	1	99.00	0	3.32	1.29	39.10	1.64	49.40
0	0	3.80	1	3.75	1.81	48.30	1.50	40.10
0	0	3.50	1	8.02	4.56	56.90	2.90	36.20
0	0	2.70	1	7.29	4.39	60.30	2.27	31.20
0	1	2.50	1	5.40	2.18	40.30	2.67	49.50
0	1	3.60	1	7.96	2.41	30.30	4.66	58.50
0	0	3.40	1	3.90	1.48	38.00	2.05	52.50
0	1	3.10	1	4.85	1.60	33.00	2.95	60.70
0	1	3.40	1	7.31	2.37	32.40	4.16	57.00
0	0	2.45	1	24.78	18.69	75.50	4.46	18.00
0	1	4.00	0	6.98	3.43	49.20	3.05	43.70
0	0	2.50	1	7.52	4.30	57.20	2.40	31.90
0	1	2.48	1	7.99	2.03	25.50	5.26	65.90
0	0	3.90	0	6.21	2.85	45.70	2.64	42.60
0	1	99.00	1	4.10	0.98	23.90	2.72	66.30
0	1	3.40	1	13.21	9.61	72.60	2.91	22.10
0	1	2.80	1	3.63	0.92	25.50	2.40	65.70
0	1	3.00	1	5.36	2.82	52.60	2.12	39.70
0	1	3.00	0	6.24	2.26	36.20	3.53	56.60
0	1	3.00	1	6.55	3.34	51.00	2.64	40.30
0	1	99.00	1	5.60	3.44	61.50	1.79	32.00
0	1	2.35	1	4.80	1.89	39.30	2.50	52.10
0	1	3.20	1	22.89	20.66	90.20	1.12	4.90
0	1	99.00	1	5.51	2.04	37.10	3.06	55.40
0	1	99.00	1	4.32	1.86	43.30	1.88	43.40
0	1	3.65	1	5.71	2.10	36.80	3.28	57.50
1	1	1.40	1	9.11	5.30	58.20	3.28	36.00
0	0	3.30	1	9.70	6.39	66.00	2.72	28.00
0	1	3.00	1	4.40	2.42	55.20	1.37	31.00
0	3	99.00	3	6.81	3.59	52.60	2.61	38.30
0	1	3.10	1	5.85	3.00	51.30	2.12	36.40
0	1	2.40	1	7.47	4.68	62.60	2.27	30.40
0	1	2.80	1	5.99	2.58	43.00	2.55	42.50
0	0	1.90	0	5.00	2.58	51.60	1.98	39.50
0	1	3.75	1	6.81	3.88	57.00	2.47	36.40
0	1	3.20	1	3.66	1.54	42.00	1.39	38.30
0	0	2.20	0	6.10	4.61	76.80	0.65	10.80

0	1	3.30	1	5.93	2.26	38.20	3.18	53.50
0	0	3.60	1	14.06	11.34	80.70	1.69	12.00
1	1	99.00	1	5.64	2.82	50.10	2.14	38.00
0	1	3.00	0	5.35	2.31	43.10	2.48	46.40
0	1	99.00	1	5.58	1.80	32.20	3.37	60.40
0	0	3.80	0	2.87	1.04	36.10	1.55	54.00
0	3	99.00	3	5.48	2.08	37.90	2.59	47.30
0	1	3.60	1	5.66	2.05	36.10	3.24	57.30
0	1	3.20	1	4.91	2.01	40.90	2.60	53.10
0	1	3.50	1	9.33	7.11	76.20	1.75	18.80
0	1	2.80	0	9.28	3.83	41.20	4.50	48.50
0	0	3.10	1	6.06	2.51	41.30	2.85	47.10
0	1	99.00	1	3.55	1.50	42.40	1.77	50.00
0	1	3.20	0	6.30	2.02	32.10	3.40	54.00
0	1	2.30	1	9.60	7.73	80.50	1.33	13.90
0	1	3.00	1	5.82	2.13	36.70	2.91	50.00
0	1	3.50	1	3.69	1.85	50.20	1.41	38.20
0	1	3.50	1	8.29	5.11	61.70	2.49	30.00
0	0	3.21	1	5.59	1.44	25.70	3.72	66.50
0	1	3.70	0	9.30	5.71	61.50	3.00	32.20
0	1	99.00	1	4.29	1.72	40.00	2.26	52.70
0	1	2.60	1	8.51	4.29	50.40	3.51	41.20
0	0	3.25	1	8.59	5.73	66.60	2.13	24.80
0	0	3.60	1	6.43	2.60	40.50	2.89	45.00
0	0	3.35	1	6.77	2.83	41.80	3.34	49.30
0	0	2.75	1	5.80	2.33	40.10	3.07	52.90
0	1	4.00	1	8.00	4.40	55.10	2.63	32.90
0	1	3.30	1	7.15	2.77	38.70	3.59	50.30
0	1	3.10	1	9.77	6.41	65.60	2.69	27.50
0	0	2.95	1	6.21	3.21	51.70	2.29	37.00
0	0	3.65	1	7.96	2.29	28.80	5.20	65.30
0	1	2.00	1	8.39	4.25	50.60	3.62	43.10
0	0	3.60	1	5.59	2.35	41.90	2.53	45.30
0	0	3.00	1	10.93	9.03	82.70	1.75	16.00
0	1	3.00	0	5.24	2.33	44.40	2.36	45.10
0	3	99.00	3	4.13	2.08	50.30	1.70	41.20
0	1	99.00	1	6.05	2.93	48.30	2.72	44.90
0	1	2.40	1	6.25	2.84	45.50	2.64	42.20
0	1	3.30	1	6.42	3.75	58.40	2.31	36.10
0	1	3.30	1	7.12	4.24	59.70	2.02	28.30
0	0	2.52	1	9.64	7.23	75.00	1.44	15.00
0	1	3.70	1	18.17	13.15	72.50	2.93	16.10
0	0	99.00	1	#NULL!	#NULL!	#NULL!	#NULL!	#NULL!
0	1	2.65	1	4.69	2.16	46.00	2.18	46.40
0	1	3.20	1	5.49	1.63	29.60	3.16	57.80
0	1	3.00	1	8.82	4.09	46.40	4.17	47.30
0	1	3.55	1	6.02	3.54	58.90	1.91	31.70
0	1	3.00	1	6.00	3.98	66.40	1.48	24.60
0	0	3.20	1	6.35	3.91	61.60	1.84	29.00
0	1	3.85	0	7.43	2.71	36.50	4.03	54.10
0	0	99.00	0	4.48	1.66	37.10	2.38	53.00
0	1	3.10	1	3.56	1.90	50.60	1.43	40.30
0	0	3.35	1	5.87	2.76	47.00	2.57	43.70
0	1	99.00	1	4.65	2.07	44.50	2.11	45.30

0	1	3.40	1	3.65	1.10	30.20	2.06	56.10
0	1	2.50	1	13.72	9.53	69.50	3.38	24.60
0	1	2.50	1	5.26	1.88	35.70	2.79	53.00
0	0	3.40	1	10.05	7.32	72.80	1.93	19.20
0	1	2.55	1	7.87	3.52	44.70	3.78	48.00
0	0	3.40	1	7.01	4.31	61.40	2.06	29.40
0	1	99.00	1	4.42	1.95	44.20	1.98	44.60
0	0	3.20	1	9.50	2.91	30.60	5.91	62.30
0	1	3.00	1	3.89	1.92	49.40	1.68	43.30
0	0	2.00	1	4.60	1.18	25.70	2.80	60.90
0	1	3.00	1	7.62	2.44	32.00	4.50	59.10
0	1	2.70	1	5.92	2.70	45.60	2.72	45.90
0	1	3.75	1	5.51	3.05	55.30	2.04	37.20
0	1	3.20	1	4.06	0.98	24.20	2.52	61.90
0	0	3.80	1	5.34	2.22	41.60	2.58	48.40
0	1	3.05	1	7.31	4.64	63.50	2.24	30.60
0	1	3.10	1	4.93	2.31	46.80	2.29	46.40
0	1	3.20	1	#NULL!	#NULL!	#NULL!	#NULL!	#NULL!
0	0	3.15	1	7.63	4.79	62.80	2.42	31.70
0	1	2.45	1	8.69	5.38	61.90	2.82	32.40
0	1	3.00	1	6.71	3.03	45.10	2.72	40.60
0	0	3.00	1	4.30	2.20	51.20	1.72	40.00
0	1	3.00	1	9.02	4.75	52.60	3.55	39.40
0	1	3.60	1	5.52	2.65	48.00	2.59	46.90
0	1	2.90	1	5.53	2.36	42.70	2.90	52.50
0	1	3.25	1	4.99	2.27	45.40	2.23	44.60
0	1	3.30	0	4.45	1.97	44.30	1.98	44.40
0	1	3.00	1	5.90	1.97	33.40	2.81	47.50
0	0	3.70	0	5.43	1.50	27.70	3.36	61.80
0	1	3.00	1	5.41	2.92	53.90	1.91	35.40
0	1	2.70	1	9.67	5.13	53.00	3.79	39.20
0	1	99.00	1	6.75	2.08	30.90	3.75	55.50
0	0	2.80	1	9.47	5.70	60.20	3.23	34.10
0	1	99.00	1	7.35	3.77	51.30	2.67	36.30
0	1	3.40	1	6.71	2.72	40.50	3.26	48.60
0	1	99.00	1	10.51	5.73	54.50	3.91	37.20
0	1	3.00	1	3.96	0.97	24.50	2.68	67.60
0	1	3.40	1	5.86	2.83	48.20	2.54	43.40
0	0	3.75	1	3.91	1.29	33.00	2.37	60.50
0	1	2.60	1	7.32	4.18	57.10	2.68	36.60
0	1	2.50	1	3.94	2.23	56.60	1.35	34.30
0	1	99.00	1	5.94	2.50	42.00	3.00	50.40
0	1	3.11	1	16.56	9.44	57.00	5.51	33.30
0	1	2.80	1	2.72	1.55	57.00	0.94	34.50
0	0	3.20	1	4.65	1.73	37.10	2.65	56.90
0	1	3.40	1	6.04	3.31	54.70	2.25	37.30
0	1	3.60	1	7.64	4.61	60.30	2.33	30.50
0	1	3.10	1	8.59	3.24	37.80	4.60	53.50
0	0	2.50	1	8.49	3.49	41.10	4.23	49.80
0	0	3.00	1	4.24	2.03	47.80	1.86	44.00
0	1	1.60	1	7.35	4.08	55.50	2.70	36.70
0	1	2.80	1	5.24	3.20	61.10	1.67	31.90
0	0	2.60	1	6.83	3.71	54.20	2.53	37.10
0	1	3.20	1	4.19	2.12	50.40	1.73	41.40

0	0	99.00	3	7.46	1.89	25.30	4.83	64.80
0	1	3.20	1	6.20	2.77	44.70	2.91	46.90
0	1	3.00	1	5.75	2.41	41.90	2.79	48.60
0	0	3.20	0	8.86	5.26	59.40	2.71	30.60
0	1	99.00	1	4.29	1.49	34.80	2.44	56.80
0	1	2.70	1	9.50	4.50	47.40	4.00	42.10
0	1	3.75	1	5.38	2.02	37.50	2.35	43.70
0	1	3.00	1	8.09	5.95	73.60	1.11	13.70
0	1	99.00	1	9.37	5.48	58.50	3.00	32.00
0	1	99.00	1	6.99	3.56	51.00	2.66	38.00
0	1	3.30	1	5.34	1.54	28.80	2.96	55.40
0	1	3.35	1	3.67	1.62	44.20	1.69	45.80
0	0	2.50	1	5.83	3.01	51.70	2.07	35.50
0	0	3.20	1	2.49	1.47	59.30	0.67	26.80
0	1	99.00	1	4.98	2.20	44.20	2.22	44.50
0	0	3.10	1	5.65	1.87	33.10	3.05	54.00
0	0	3.10	0	8.89	3.41	38.40	4.39	49.30
0	1	3.20	1	5.11	2.01	39.40	2.67	52.30
0	1	3.50	1	5.62	1.95	34.70	2.97	52.80
0	0	3.60	1	7.43	4.42	59.50	2.07	27.80
0	1	99.00	1	7.03	4.09	58.20	1.95	27.70
0	0	3.20	1	4.67	1.96	41.90	1.84	39.60

单核	单核百分比	嗜酸	嗜酸百分比	RBC	HCT百分比	HB	MCV	MCH
0.27	5.80	0.18	3.90	4.66	39.8	130.0	85.40	27.9
0.25	7.20	0.12	3.60	4.90	40.7	139.0	83.10	28.4
0.21	3.90	0.11	2.10	5.07	46.4	157.0	91.50	31.0
0.41	4.50	0.08	0.90	4.79	40.0	127.0	83.50	26.5
0.28	5.20	0.11	2.10	4.82	44.9	147.0	93.20	30.5
0.38	3.60	0.10	1.00	4.76	39.1	130.0	82.10	27.3
0.25	2.90	0.07	0.80	5.13	42.3	145.0	82.50	28.3
0.28	5.20	0.03	0.60	4.93	41.4	131.0	84.00	26.6
0.26	5.10	0.02	0.30	4.94	39.2	128.0	79.40	25.9
0.41	7.80	0.11	2.10	4.48	41.0	133.0	78.00	26.2
0.44	5.60	0.25	3.20	5.56	44.1	148.0	79.30	26.6
0.31	3.90	0.12	1.50	4.55	39.7	129.0	87.30	28.4
0.57	6.60	0.29	3.30	5.32	43.0	145.0	80.80	27.3
0.24	4.50	0.03	0.50	4.91	42.3	141.0	86.20	28.7
0.85	6.20	0.19	1.40	4.79	36.5	117.0	76.20	24.4
0.45	8.40	0.04	0.80	4.38	35.4	122.0	80.80	27.9
0.46	3.50	0.43	3.30	4.91	37.1	121.0	75.60	24.6
0.26	6.80	0.13	3.30	3.45	28.9	93.0	83.80	27.0
0.48	4.50	0.10	0.90	7.55	50.8	155.0	67.30	20.5
0.49	3.20	0.00	0.00	4.80	38.5	129.0	80.20	26.9
0.53	6.40	0.26	3.20	5.19	44.7	146.0	86.10	28.1
0.36	3.70	0.05	0.50	4.97	42.7	147.0	85.90	29.6
0.15	3.50	0.06	1.30	4.96	39.7	130.0	80.00	26.2
0.25	3.40	0.13	1.80	5.26	44.2	142.0	84.00	27.0
0.29	2.80	0.24	2.30	5.09	39.0	138.0	83.00	28.0
0.18	6.20	0.10	3.50	4.23	36.6	119.0	86.50	28.1
0.25	4.20	0.15	2.50	4.86	40.0	143.0	82.30	29.4
0.58	4.60	0.20	1.60	5.34	42.0	155.0	85.20	29.3
0.13	2.50	0.06	1.20	4.80	39.9	133.0	83.10	27.7
0.33	5.10	0.03	0.50	4.46	45.0	130.0	83.00	27.5
0.16	3.60	0.03	0.70	4.20	38.0	125.0	82.00	27.8
0.22	3.80	0.07	1.20	4.73	40.4	139.0	85.40	29.4
0.60	4.50	0.12	0.90	4.94	39.2	135.0	79.40	27.3
0.23	4.20	0.18	3.30	5.04	42.5	140.0	84.30	27.8
0.46	2.80	0.46	2.80	4.36	41.5	131.0	95.20	30.0
0.10	1.90	0.08	1.60	4.75	40.5	134.0	85.30	28.2
0.33	3.20	0.12	1.20	4.66	40.4	132.0	86.70	28.3
0.49	9.80	0.20	3.90	4.50	34.4	111.0	76.40	24.7
0.18	3.30	0.10	1.80	3.81	31.6	102.8	82.90	26.8
0.72	4.10	0.14	0.80	4.20	41.0	120.0	81.00	27.2
0.43	11.50	0.02	0.50	3.15	24.8	72.0	78.70	72.9
0.30	4.90	0.04	0.70	5.11	42.0	143.0	85.00	27.0
0.45	6.40	0.27	3.80	5.43	46.7	159.0	86.00	29.3
0.21	4.10	0.04	0.70	4.50	39.0	123.0	85.00	28.5
0.14	4.50	0.02	0.50	4.20	40.0	123.0	84.30	29.0
0.39	7.84	0.44	8.80	4.51	39.4	128.0	87.00	28.4
0.19	3.90	0.09	1.90	4.53	40.0	131.0	83.00	29.2
0.51	6.70	0.06	0.80	4.42	42.7	141.0	56.60	31.9
0.30	5.10	0.10	1.70	4.41	41.5	137.0	93.00	30.9
0.45	6.40	0.04	0.60	5.42	56.6	157.0	85.80	29.0
0.48	4.60	0.00	0.00	3.93	34.0	109.0	66.50	27.7
0.56	7.64	0.35	4.80	5.52	44.7	147.0	81.00	26.0
0.12	4.00	0.01	0.30	4.63	40.2	138.0	86.00	29.8

0.42	8.70	0.01	0.20	4.60	40.2	128.0	57.40	27.8
0.23	4.00	0.05	0.90	4.78	40.6	129.0	84.00	27.0
0.27	4.70	0.02	0.30	2.70	24.2	77.0	89.00	28.5
1.50	10.20	0.01	0.10	3.74	31.1	98.0	83.20	26.2
0.33	5.70	0.14	2.40	4.41	38.7	127.0	87.80	28.8
0.28	6.80	0.23	5.60	5.06	44.5	156.0	87.90	30.8
0.35	8.50	0.04	1.00	4.67	45.0	148.0	96.40	31.7
0.41	6.50	0.49	7.80	5.12	40.9	135.0	79.00	26.4
0.11	2.50	0.06	1.30	5.10	42.0	122.0	82.00	28.0
0.38	3.30	0.01	0.10	4.72	39.4	132.0	83.00	28.0
0.42	6.10	0.01	0.10	5.26	37.6	119.0	71.00	22.6
0.50	7.50	0.05	0.70	3.58	28.4	90.0	79.30	26.1
0.27	6.20	0.12	2.70	4.87	43.0	141.0	88.30	29.0
0.38	6.60	0.34	5.90	5.35	42.9	141.0	80.20	26.4
0.40	6.60	0.31	5.10	4.76	37.7	125.0	79.00	26.0
0.40	5.90	0.04	0.60	2.34	19.9	63.0	85.00	26.9
0.45	6.00	0.05	0.70	2.84	22.0	74.0	79.20	26.0
0.52	7.80	0.13	1.90	4.82	38.5	121.0	79.90	26.0
0.34	5.20	0.14	2.10	4.55	39.0	125.0	80.00	28.0
0.53	9.30	0.46	8.00	5.35	42.1	142.0	78.70	26.5
0.23	4.60	0.14	2.80	4.95	41.1	136.0	83.00	27.5
0.23	2.90	0.05	0.60	4.02	36.5	121.0	90.80	30.1
0.47	6.90	0.24	3.50	4.60	38.1	127.0	82.80	27.6
0.54	6.20	0.08	0.90	4.99	43.5	143.0	87.20	28.7
0.19	4.00	0.05	1.10	5.14	42.3	146.0	82.30	28.4
0.48	14.11	0.02	0.60	4.89	42.6	148.0	87.10	30.3
0.44	3.60	0.19	1.60	5.42	42.0	154.0	83.00	32.0
0.58	7.20	0.09	1.10	5.33	46.9	150.0	86.10	28.1
0.40	6.30	0.02	0.30	3.27	25.6	82.0	78.50	36.1
0.25	4.90	0.10	1.90	4.71	40.9	136.0	86.80	28.9
0.52	4.50	0.00	0.00	3.19	25.5	88.0	79.90	27.5
0.55	6.70	0.13	1.60	5.22	42.4	142.0	81.00	27.2
0.35	7.70	0.10	2.20	4.11	35.0	117.0	85.20	28.5
0.56	4.60	0.13	1.10	2.71	21.3	74.0	78.60	27.3
0.31	5.30	0.04	0.70	5.36	49.1	162.0	91.60	30.2
0.52	4.60	0.05	0.40	4.85	39.8	139.0	82.10	28.7
0.34	4.30	0.13	1.60	4.59	39.6	128.0	85.30	27.9
0.27	5.54	0.17	3.50	4.67	37.8	126.0	80.90	27.0
0.26	4.60	0.27	4.80	4.62	39.5	125.0	85.50	27.3
0.36	3.00	0.00	0.00	4.70	38.0	134.0	79.00	28.0
0.64	5.70	0.14	1.30	5.03	45.2	147.0	59.00	29.2
0.30	6.90	0.10	2.30	4.18	36.3	112.0	86.80	26.8
0.17	4.50	0.03	0.80	5.14	43.4	144.0	84.40	28.0
0.48	9.20	0.09	1.70	4.43	38.6	130.0	87.00	29.0
0.47	4.50	0.24	2.30	5.83	38.0	121.0	61.00	20.0
0.13	1.90	0.01	0.10	4.80	40.0	132.0	83.00	27.6
0.21	4.30	0.13	2.70	4.51	40.7	137.0	90.00	30.0
0.51	5.40	0.08	0.80	5.14	39.4	139.0	76.70	27.0
0.40	3.80	0.06	0.60	4.85	39.8	138.0	82.00	28.0
0.30	4.50	0.06	0.90	4.25	42.0	128.0	84.00	29.2
0.33	4.70	0.51	7.30	4.90	44.4	145.0	89.00	29.0
0.33	5.70	0.37	6.40	5.30	43.7	143.0	82.00	27.0
0.27	2.80	0.11	1.10	4.33	36.6	126.0	84.00	29.0
0.54	3.60	0.15	1.00	3.27	25.9	86.0	79.00	26.3

0.26	4.90	0.13	2.50	6.99	56.4	191.0	80.00	27.0
0.22	4.20	0.06	1.20	4.21	38.0	122.0	81.20	28.3
0.28	4.20	0.20	3.00	5.13	44.5	147.0	86.00	28.7
0.28	4.70	0.10	1.70	4.75	43.9	143.0	92.40	30.1
0.43	8.00	0.13	2.40	4.73	42.0	134.0	88.00	28.3
0.35	6.70	0.09	1.70	4.37	37.1	110.0	84.90	25.2
0.20	3.90	0.18	3.50	4.88	40.7	129.0	83.40	26.4
0.23	3.40	0.12	1.80	5.41	45.7	151.0	84.00	27.9
0.40	11.80	0.02	0.60	2.31	20.2	57.0	86.30	24.4
0.40	5.20	0.16	2.10	4.69	39.8	129.0	84.90	27.6
0.46	10.00	0.17	3.70	4.66	41.5	135.0	89.10	29.0
0.36	5.30	0.02	0.30	4.69	42.9	136.0	91.60	29.0
0.20	4.40	0.11	2.40	5.06	42.7	135.0	84.40	26.7
0.39	5.10	0.05	0.60	5.27	44.4	141.0	84.30	26.8
0.41	7.40	0.12	2.20	5.00	41.6	134.0	83.20	26.8
0.33	4.20	0.06	0.80	4.58	37.0	122.0	82.30	27.8
0.41	5.70	0.06	0.80	4.53	40.6	128.0	89.00	28.0
0.40	4.50	0.57	6.40	4.49	38.5	123.0	85.70	27.4
0.21	3.00	0.08	1.10	4.88	44.7	146.0	91.60	29.9
0.20	5.20	0.16	4.20	4.56	40.0	127.0	85.80	27.3
0.38	7.10	0.08	1.50	4.79	41.9	137.0	87.50	28.6
0.25	3.90	0.02	0.30	2.01	17.9	52.0	59.10	25.8
0.31	4.70	0.28	4.30	4.92	39.4	126.0	80.00	25.6
0.36	8.90	0.04	1.00	4.70	39.4	128.0	83.80	27.2
0.43	7.10	0.37	6.10	4.76	41.5	132.0	87.20	27.0
0.43	5.10	0.29	3.40	5.07	40.1	139.0	79.00	27.0
0.25	5.20	0.62	12.80	4.57	39.0	126.0	85.30	27.6
0.31	7.10	0.10	2.30	4.99	42.0	134.0	84.20	26.9
0.42	12.00	0.00	0.00	3.62	34.7	100.0	90.10	28.0
0.33	7.04	0.16	3.40	3.25	29.5	91.0	90.80	29.0
0.69	3.50	0.22	1.10	4.05	35.4	108.0	87.00	26.7
1.22	9.30	0.07	0.50	5.32	43.9	154.0	82.50	28.9
0.45	4.30	0.44	4.20	4.73	40.8	144.0	86.30	30.4
0.20	5.30	0.05	1.30	4.85	40.8	138.0	87.70	29.7
0.44	7.20	0.01	0.20	5.28	41.5	146.0	78.60	27.7
0.50	6.30	0.24	3.00	5.52	44.7	157.0	81.00	28.4
0.97	7.30	0.01	0.10	5.81	38.0	120.0	65.40	20.7
0.30	5.00	0.27	4.40	4.63	37.8	131.0	81.60	28.3
0.27	6.40	0.14	3.30	4.72	38.1	127.0	80.70	36.9
0.39	5.50	0.13	1.80	5.06	39.5	132.0	78.10	26.1
0.32	5.70	0.04	0.70	2.94	24.8	84.0	84.40	28.6
0.32	5.20	0.15	2.40	4.94	39.8	139.0	80.60	28.1
0.21	4.74	0.13	2.90	5.07	43.6	151.0	86.00	29.8
0.15	3.10	0.08	1.60	3.72	29.9	95.0	80.40	25.5
0.37	6.50	0.09	1.60	4.77	39.7	135.0	83.20	28.3
0.20	3.54	0.23	4.00	4.65	29.1	91.0	62.60	19.6
0.35	5.80	0.41	6.70	4.75	39.1	137.0	82.30	28.8
0.18	1.90	0.00	0.00	4.49	40.2	132.0	89.50	29.4
0.81	16.01	0.18	3.60	4.67	41.7	142.0	89.30	30.4
0.29	8.80	0.17	5.00	4.00	41.0	130.0	85.30	31.2
0.37	6.70	0.15	2.70	4.79	39.2	133.0	81.90	27.8
0.19	3.70	0.20	3.90	4.92	38.9	129.0	79.10	26.2
0.28	6.30	0.07	1.60	3.84	34.1	108.0	88.80	28.1
0.43	8.80	0.02	0.40	4.74	39.5	135.0	83.30	28.5

0.44	6.70	0.12	1.80	4.70	38.9	128.0	82.80	27.2
0.43	7.00	0.37	6.10	4.71	39.7	135.0	84.30	28.7
0.74	10.04	0.11	1.50	4.26	31.8	90.0	74.60	21.1
0.22	3.90	0.04	0.70	4.86	43.3	150.0	89.10	30.9
0.21	4.10	0.05	0.90	5.10	40.0	130.0	84.00	30.2
0.22	4.50	0.04	0.80	4.70	41.2	138.0	87.70	29.0
0.50	4.60	0.10	0.90	4.83	40.8	141.0	84.50	29.0
0.16	3.80	0.05	1.10	4.60	40.4	140.0	87.80	30.4
0.33	5.10	0.08	1.20	3.41	31.5	102.0	91.60	29.7
0.22	2.90	0.01	0.10	4.99	41.8	148.0	83.80	29.7
0.50	6.10	0.16	2.00	4.77	40.8	135.0	85.50	28.3
0.26	5.70	0.24	5.30	4.65	40.7	136.0	87.50	29.2
0.39	7.50	0.11	2.10	5.42	43.1	141.0	79.50	26.0
0.10	2.30	0.15	3.40	2.48	20.1	59.0	81.00	23.8
0.50	7.54	0.59	8.90	4.46	38.1	127.0	85.60	28.5
0.58	8.70	0.15	2.20	4.84	39.6	137.0	81.80	28.3
0.23	4.20	0.09	1.70	4.64	39.3	130.0	84.70	28.0
0.34	7.30	0.04	0.90	4.58	37.0	129.0	80.80	28.2
0.39	5.74	0.10	1.50	5.32	43.2	150.0	81.20	28.2
0.31	5.70	0.46	8.40	4.93	41.9	143.0	85.00	29.0
0.54	4.70	0.03	0.30	4.24	36.5	119.0	86.10	28.1
0.43	4.90	0.07	0.80	5.06	43.9	151.0	86.80	29.8
0.19	4.60	0.12	2.90	4.42	40.3	135.0	91.20	30.5
0.45	7.30	1.12	18.20	5.76	45.3	152.0	78.60	26.4
0.18	5.20	0.08	2.30	4.30	41.0	134.0	83.00	28.5
0.35	2.60	0.00	0.00	3.37	31.7	109.0	85.00	29.2
0.28	5.60	0.09	1.80	4.23	38.8	130.0	91.70	30.7
0.35	6.40	0.01	0.20	4.16	33.0	112.0	79.30	36.9
1.14	8.14	0.17	1.20	3.96	32.1	101.0	81.10	25.5
0.39	7.10	0.11	2.00	3.81	32.2	102.0	84.50	20.8
0.23	5.20	0.05	1.10	4.51	38.0	126.0	84.30	27.9
0.32	5.50	0.13	2.20	4.54	38.7	134.0	85.20	29.5
0.16	5.30	0.05	1.70	2.79	23.7	76.0	84.90	27.2
0.16	4.00	0.30	7.40	3.82	26.4	72.0	69.10	18.8
0.23	6.70	0.09	2.60	4.50	38.0	131.0	85.80	29.1
1.07	8.30	0.04	0.30	5.21	43.8	146.0	84.10	28.0
0.26	6.40	0.08	2.00	4.62	40.3	129.0	87.20	27.9
0.47	6.70	0.04	0.60	4.50	38.1	130.0	84.70	28.9
0.61	4.80	0.27	2.10	4.56	39.0	129.0	85.50	28.3
0.16	4.24	0.15	4.00	4.47	40.0	138.0	89.90	30.9
0.21	2.04	0.00	0.00	4.22	33.0	130.0	78.00	30.8
0.22	6.90	0.05	1.60	4.95	40.7	134.0	82.20	27.1
0.53	10.70	0.12	2.40	4.92	39.7	137.0	80.70	27.8
0.54	5.10	0.22	2.10	4.39	33.6	106.0	76.60	24.0
0.28	7.20	0.09	2.20	3.61	30.6	100.0	84.00	27.7
0.18	3.60	0.05	1.10	4.38	37.0	129.0	84.70	29.5
0.24	4.10	0.05	0.80	2.77	23.0	78.0	83.80	28.2
0.19	4.60	0.10	2.40	5.38	40.9	140.0	76.00	26.0
0.62	8.50	0.22	3.00	4.98	41.3	134.0	82.90	26.9
0.27	3.80	0.00	0.00	4.96	40.0	147.0	80.60	29.6
0.78	6.60	3.28	27.60	3.58	40.0	105.0	86.90	28.8
0.37	10.80	0.08	2.30	4.76	40.0	140.0	84.50	29.4
0.49	6.00	0.49	6.00	4.65	52.0	102.0	71.00	22.5
0.25	7.20	0.04	1.10	4.50	39.3	136.0	87.30	30.2

0.27	6.30	0.18	4.20	4.86	44.4	144.0	91.40	29.6
0.39	3.00	0.03	0.20	3.92	35.8	132.0	91.30	33.7
0.27	4.70	0.17	2.90	4.56	39.7	138.0	87.10	30.3
0.50	4.80	0.01	0.10	3.25	30.0	101.0	92.30	31.1
0.34	4.50	0.08	1.00	4.20	32.0	141.0	90.00	32.0
0.24	5.40	0.13	2.90	4.27	40.0	133.0	93.70	31.1
0.66	4.70	0.34	2.40	3.95	39.0	114.0	85.30	28.9
0.67	5.00	0.25	1.90	4.09	37.0	126.0	91.00	30.8
0.32	8.10	0.13	3.30	3.06	29.3	93.0	89.30	28.4
0.58	5.70	0.00	0.00	4.33	37.3	129.0	86.10	29.8
2.11	7.54	0.11	0.40	5.85	32.0	109.0	80.00	18.6
0.17	3.80	0.06	1.40	4.60	39.9	136.0	86.70	39.6
0.38	5.80	0.19	2.90	4.39	38.0	130.0	86.80	29.6
0.20	5.60	0.51	14.60	5.37	40.5	137.0	76.00	25.5
0.34	5.80	0.41	7.00	4.60	39.0	132.0	84.90	28.0
0.33	11.20	0.07	2.40	4.00	36.0	127.0	91.00	31.8
0.50	9.00	0.07	1.30	5.35	42.7	157.0	79.80	29.3
0.45	6.00	0.12	1.60	4.43	36.7	126.0	82.80	28.4
0.30	5.50	0.32	5.90	4.45	37.4	133.0	84.00	29.9
0.57	9.00	0.68	10.70	3.91	28.4	97.0	72.60	24.8
0.41	7.80	0.05	0.90	2.09	23.4	78.0	111.00	37.3
0.33	5.80	0.25	4.30	4.64	40.9	137.0	88.00	29.6
0.36	7.40	0.09	1.90	4.30	39.0	129.0	91.20	30.0
0.33	5.60	0.09	1.50	4.23	36.0	127.0	85.30	30.0
0.52	8.30	0.23	3.70	4.43	35.6	125.0	80.40	28.2
0.16	5.10	0.11	3.70	3.98	36.5	124.0	91.90	31.3
0.31	6.30	0.13	2.60	2.92	27.7	89.0	94.90	30.5
0.15	4.30	0.12	3.50	4.00	41.0	132.0	85.00	32.0
0.42	6.50	0.46	7.10	5.30	43.5	147.0	82.10	27.7
0.14	4.30	0.18	5.60	2.90	36.0	98.0	75.00	25.4
1.25	6.70	0.02	0.10	4.86	38.7	137.0	79.60	28.2
0.24	4.20	0.38	6.70	3.79	33.3	104.0	87.90	27.4
0.66	9.00	0.59	8.00	4.71	36.0	127.0	78.30	27.0
0.51	7.50	0.16	2.30	4.79	40.6	131.0	84.80	27.3
0.62	8.90	0.19	2.80	3.80	24.4	72.0	64.46	18.9
0.88	6.00	0.26	1.80	3.75	29.4	105.0	78.40	28.0
0.46	3.50	0.09	0.70	4.77	40.9	139.0	85.70	29.1
0.27	4.90	0.38	6.90	4.72	39.7	139.0	84.00	29.4
1.37	9.80	0.07	0.50	4.55	37.6	129.0	83.10	28.4
0.21	5.40	0.07	1.80	2.15	18.4	63.0	85.60	29.3
0.21	3.54	0.01	0.20	4.51	38.0	132.0	84.00	29.3
1.00	6.24	0.02	0.10	4.45	27.6	92.0	62.00	20.7
0.92	14.44	0.04	0.60	4.73	43.6	149.0	92.30	31.6
0.11	1.01	0.13	1.20	4.85	42.0	144.0	86.99	29.8
0.73	7.56	0.13	1.30	4.55	38.8	131.0	85.29	26.8
0.54	9.38	0.35	6.10	4.19	39.0	136.0	93.15	32.5
0.59	8.44	0.09	1.30	3.03	27.8	91.0	91.70	30.0
0.39	6.20	0.33	5.20	4.36	36.5	125.0	83.70	28.7
0.80	7.80	0.03	0.30	5.39	45.0	160.0	83.00	27.8
0.44	6.30	0.19	2.70	4.65	39.0	130.0	84.70	28.0
0.33	7.00	0.05	1.10	4.63	39.3	135.0	84.90	29.0
0.30	7.14	0.06	1.40	5.65	35.5	114.0	62.87	20.2
0.44	5.50	0.10	1.30	4.45	39.0	137.0	86.70	30.8
0.44	8.10	0.25	4.60	3.03	25.7	82.0	84.80	27.1

0.20	4.60	0.53	12.30	3.20	22.8	68.0	71.30	21.3
0.44	7.60	0.39	6.70	6.25	39.6	134.0	63.40	21.4
0.31	5.30	0.11	1.90	4.71	37.6	125.0	79.80	26.5
1.12	10.00	0.01	0.10	2.70	22.6	76.0	83.70	28.9
0.34	8.60	0.14	3.50	4.23	35.0	123.0	84.40	29.1
0.44	10.90	0.10	2.50	5.41	42.0	155.0	77.30	28.7
0.18	5.60	0.20	6.20	4.50	48.0	123.0	81.00	28.0
0.56	6.40	0.09	1.00	4.81	40.0	142.0	83.80	29.3
0.64	8.68	0.16	2.20	3.72	32.5	110.0	87.40	29.5
0.41	5.80	0.54	7.60	5.22	42.0	144.0	81.40	27.6
0.23	5.80	0.04	1.00	2.92	22.6	76.0	77.40	26.0
0.39	5.70	0.24	3.50	4.48	38.8	133.0	86.60	29.7
0.38	4.20	0.07	0.80	3.96	33.0	115.0	83.60	29.0
0.30	4.59	0.14	2.20	4.65	43.7	148.0	94.00	31.8
0.65	7.78	0.20	2.40	4.05	35.0	115.0	88.29	28.7
0.40	7.71	0.17	3.30	4.89	41.0	148.0	85.00	30.0
0.39	6.30	0.14	2.20	4.78	40.0	137.0	83.00	28.7
0.23	5.30	0.12	2.80	3.29	29.0	102.0	59.00	31.0
0.15	4.20	0.05	1.30	3.05	27.0	92.0	89.00	30.0
0.47	4.90	0.10	1.10	3.08	25.0	85.0	81.50	27.6
0.56	5.43	0.26	2.50	3.51	35.0	125.0	100.00	35.5
0.36	5.00	0.12	1.70	4.05	34.0	123.0	85.00	30.1
0.41	5.10	0.10	1.30	4.19	36.5	126.0	87.00	30.0
0.20	3.20	0.11	1.80	5.00	42.0	139.0	84.20	27.0
0.32	6.40	0.16	3.20	4.71	39.0	136.0	82.00	28.9
0.26	4.10	0.01	0.20	5.22	40.0	125.0	77.80	23.9
0.46	7.46	0.31	5.00	4.01	35.6	120.0	55.60	29.9
0.20	3.20	0.04	0.60	4.20	35.6	118.0	84.70	28.0
1.29	7.30	0.16	0.90	4.94	40.0	135.0	80.90	27.3
0.31	7.77	0.06	1.60	5.02	42.2	156.0	84.00	31.1
0.27	7.60	0.11	3.20	4.60	40.0	122.0	81.20	32.0
0.12	3.10	0.02	0.60	4.30	40.0	135.0	88.00	32.0
0.20	3.80	0.03	0.50	5.22	44.0	148.0	84.70	28.4
0.05	1.20	0.04	0.90	4.02	41.0	125.0	89.00	31.0
0.13	2.90	0.07	1.60	3.37	27.0	96.0	80.60	28.5
0.22	3.60	0.11	1.80	4.09	38.0	124.0	81.40	28.4
0.26	4.30	0.12	1.90	4.37	35.0	124.0	82.20	28.4
0.30	7.20	0.11	2.50	4.30	42.0	125.0	81.50	29.0
0.25	5.20	0.19	4.10	4.32	37.0	124.0	84.70	28.7
0.56	7.99	0.03	0.40	4.79	41.0	142.0	84.60	29.6
0.31	5.50	0.10	1.80	4.71	42.0	146.0	88.00	31.0
0.44	6.00	0.33	4.50	4.27	38.0	126.0	88.80	29.5
0.08	2.00	0.15	3.80	4.08	35.0	98.0	85.50	24.0
0.21	4.40	0.05	1.00	4.73	41.0	140.0	86.90	29.6
0.41	5.00	0.06	0.70	4.77	40.0	134.0	83.60	28.1
0.23	2.90	0.35	4.50	4.62	39.0	135.0	85.30	29.2
0.31	5.50	0.08	1.40	5.08	44.0	146.0	86.50	28.5
0.36	8.30	0.13	3.00	4.99	43.0	150.0	87.00	30.1
0.32	5.40	0.57	9.60	4.85	42.0	139.0	85.40	28.6
0.30	7.00	0.15	3.50	4.85	40.0	139.0	82.10	28.7
0.18	3.40	0.08	1.50	4.28	40.0	124.0	93.00	29.0
0.37	5.20	0.08	1.10	5.61	47.0	173.0	83.80	30.8
0.48	8.80	0.09	1.70	4.88	41.0	139.0	84.40	28.6
0.43	6.70	0.11	1.70	3.90	27.0	81.0	66.90	20.3

0.38	5.50	0.18	2.60	5.31	47.0	158.0	89.30	29.8
0.64	7.10	0.38	4.20	5.20	43.0	143.0	81.00	27.5
0.34	7.00	0.08	1.60	4.57	39.0	136.0	86.00	29.8
0.37	6.80	0.11	2.00	4.52	42.0	137.0	91.80	30.3
0.42	5.40	0.20	2.60	5.09	42.0	139.0	82.00	27.3
0.34	6.70	0.10	1.90	5.01	41.0	142.0	82.00	28.3
0.27	4.70	0.24	4.20	5.00	44.0	152.0	87.00	30.4
0.36	7.20	0.21	4.20	5.11	41.0	146.0	81.00	28.6
0.43	4.90	0.67	7.70	4.58	38.0	131.0	83.80	28.6
0.32	6.30	0.03	0.60	5.29	42.0	134.0	79.60	25.3
0.48	8.10	0.13	2.20	4.20	39.0	131.0	93.00	31.0
0.43	7.90	0.11	2.00	4.19	37.0	139.0	89.00	30.8
0.81	9.20	0.07	0.80	4.58	39.0	129.0	85.20	28.2
0.16	3.80	0.23	5.50	5.35	46.0	159.0	85.40	29.7
0.24	3.80	0.15	2.40	5.04	42.0	145.0	83.70	28.8
0.17	3.40	0.09	1.80	4.78	41.0	139.0	86.20	29.1
0.54	7.70	0.02	0.30	4.86	42.0	146.0	86.40	30.0
0.34	6.00	0.21	3.70	4.63	40.0	137.0	88.30	30.2
0.20	2.70	0.08	1.10	4.82	40.0	135.0	83.00	28.0
0.50	5.50	0.12	1.30	5.21	43.0	143.0	82.00	27.4
0.91	6.40	0.01	0.10	5.17	41.0	136.0	79.50	26.3
0.27	5.10	0.12	2.30	4.20	43.0	132.0	82.00	28.5
0.40	4.70	0.35	4.10	5.06	40.0	135.0	78.20	26.7
0.21	4.60	0.09	2.00	4.08	37.0	125.0	91.40	30.6
0.19	5.30	0.09	2.50	4.86	41.0	140.0	84.80	28.8
0.64	5.50	0.01	0.10	4.81	40.0	130.0	82.30	27.0
0.37	7.60	0.14	2.90	4.61	40.0	136.0	85.30	29.3
0.70	9.90	0.03	0.40	4.96	40.0	136.0	83.30	27.4
0.28	6.80	0.05	1.20	5.68	45.0	146.0	80.00	26.2
0.27	4.70	0.05	0.90	4.86	41.0	139.0	84.40	28.6
0.39	5.30	0.01	0.10	2.01	17.0	58.6	86.10	28.9
0.54	3.90	0.17	1.20	3.66	33.0	90.0	80.90	21.6
0.30	7.20	0.10	2.40	4.79	41.0	138.0	85.40	28.8
0.30	8.20	0.33	9.00	4.51	38.0	125.0	84.00	27.7
0.54	7.70	0.06	0.90	5.62	49.0	170.0	86.00	30.2
0.95	18.20	0.00	0.00	4.73	37.0	110.0	78.90	23.3
0.93	5.10	0.02	0.10	2.36	22.0	66.0	91.10	28.0
0.43	6.10	0.43	6.10	4.23	26.0	68.0	62.20	16.1
0.19	4.20	0.07	1.60	4.50	45.0	133.0	81.20	32.5
0.30	6.80	0.08	1.80	4.80	41.0	144.0	84.80	30.0
0.20	4.20	0.15	3.20	5.06	44.0	153.0	86.20	30.2
0.50	6.20	0.18	2.20	4.67	40.0	130.0	84.60	27.8
0.19	3.60	0.08	1.50	5.19	46.0	154.0	88.20	29.7
0.33	6.70	0.12	2.40	4.53	41.0	136.0	90.90	30.0
0.27	5.40	0.27	5.40	4.23	35.0	111.0	83.20	26.3
0.40	8.50	0.09	1.90	4.62	42.0	142.0	91.10	30.7
0.22	9.10	0.08	3.30	3.41	30.0	93.0	88.40	27.0
0.49	7.00	0.38	5.50	5.72	48.0	166.0	84.00	29.0
0.58	10.60	0.30	5.50	5.53	38.0	109.0	68.90	19.7
0.26	4.80	0.07	1.30	5.63	49.0	167.0	57.70	29.7
0.26	4.70	0.11	2.00	4.02	33.0	102.0	81.00	25.4
0.30	5.30	0.06	1.10	4.33	40.0	138.0	92.00	32.0
0.30	5.10	0.10	1.70	4.09	41.0	133.0	99.00	32.5
0.62	8.60	0.19	2.60	5.41	46.0	147.0	85.80	27.0

0.59	9.80	0.20	3.30	4.45	41.0	141.0	93.00	31.7
0.41	7.10	0.17	2.90	5.25	46.0	152.0	86.70	29.0
0.59	7.40	0.06	0.80	3.86	31.0	97.0	79.00	25.0
0.31	4.90	0.07	1.10	5.31	46.0	168.0	85.70	31.6
0.20	8.80	0.01	0.40	2.43	22.0	71.0	89.70	29.2
0.54	8.90	0.33	5.40	4.78	43.0	144.0	89.00	30.1
0.36	9.40	0.13	3.40	4.76	41.0	133.0	85.60	27.9
0.46	10.19	0.03	0.70	4.36	39.0	134.0	88.80	30.7
0.35	5.30	0.36	5.40	4.66	35.0	117.0	75.50	24.0
0.27	6.60	0.14	3.40	4.55	37.0	128.0	81.30	28.1
0.38	6.00	0.03	0.50	4.97	40.0	132.0	81.30	26.6
0.24	4.40	0.25	4.60	4.93	42.0	146.0	84.00	29.3
0.43	9.30	0.08	1.70	4.87	42.0	142.0	86.90	29.2
0.34	8.50	0.20	5.00	4.14	39.0	132.0	94.00	31.9
0.40	8.40	0.12	2.50	5.35	45.0	158.0	83.00	29.0
0.46	7.30	0.15	2.40	4.23	37.0	118.0	87.50	27.9
0.28	5.90	0.11	2.30	4.75	41.0	139.0	86.30	28.3
0.50	7.90	0.14	2.20	5.40	48.0	158.0	88.00	29.3
0.33	6.40	0.17	3.30	4.48	38.0	128.0	85.30	28.6
0.32	8.60	0.10	2.70	4.79	42.0	139.0	88.30	29.0
0.52	6.90	0.32	4.20	4.79	41.0	134.0	85.60	28.0
0.22	4.20	0.16	3.10	4.66	38.0	138.0	88.00	32.0
0.72	10.60	0.07	1.00	4.94	41.0	130.0	83.20	26.3
0.52	10.40	0.18	3.60	5.48	38.0	107.0	95.00	19.5
0.27	6.40	0.08	1.90	4.71	41.0	138.0	86.00	29.3
0.61	8.10	0.33	4.40	4.59	42.0	145.0	91.90	31.6
0.44	9.30	0.06	1.30	5.41	43.0	148.0	80.00	27.4
0.34	6.90	0.05	1.00	4.19	38.0	128.0	90.60	30.5
0.46	8.30	0.07	1.30	3.67	30.0	99.0	82.60	27.0
0.37	7.60	0.11	2.20	5.09	44.0	151.0	86.60	29.7
0.49	5.70	0.66	7.60	5.23	34.0	107.0	88.00	26.0
0.18	5.40	0.15	4.50	4.00	38.0	128.0	83.00	27.0
0.49	6.30	0.46	5.90	5.33	37.0	119.0	88.00	22.4
0.20	3.40	0.07	1.20	4.27	40.0	134.0	93.70	31.4
0.56	10.40	0.02	0.40	4.65	38.0	129.0	81.90	27.7
0.53	7.10	0.10	1.30	4.45	38.0	132.0	86.00	29.7
0.52	4.90	0.21	2.00	6.46	37.0	114.0	68.00	20.0
0.29	6.30	0.02	0.40	3.64	34.0	116.0	92.60	31.9
0.40	7.90	0.14	2.80	4.29	39.0	122.0	91.10	28.5
1.05	22.90	0.16	3.50	4.13	25.0	69.0	59.00	16.7
0.26	6.70	0.32	8.20	4.63	40.0	139.0	86.80	30.0
0.64	7.50	0.19	2.20	4.50	40.0	129.0	89.60	29.7
0.63	6.70	0.28	3.00	4.44	40.0	135.0	89.40	30.4
0.41	8.30	0.10	2.00	4.71	42.0	144.0	89.00	30.6
0.17	5.10	0.07	2.20	3.00	32.0	99.0	76.00	27.6
0.70	11.00	0.13	2.00	2.73	22.0	73.0	81.00	26.7
0.85	13.60	0.03	0.50	5.91	48.0	172.0	81.60	29.1
0.45	6.30	0.10	1.40	5.62	52.0	173.0	92.70	30.8
0.29	4.70	0.14	2.30	6.08	35.0	109.0	57.60	47.0
0.56	6.80	0.29	3.50	5.12	45.0	150.0	87.00	29.3
0.24	5.30	0.12	2.50	4.50	37.0	127.0	83.00	29.0
0.81	9.80	0.26	3.20	5.13	44.0	151.0	84.80	29.4
0.79	9.90	0.21	2.60	6.45	51.0	173.0	79.00	26.8
0.26	6.70	0.11	2.80	4.89	44.0	148.0	89.00	30.0

0.40	7.80	0.11	2.10	4.65	39.0	132.0	84.70	28.4
0.59	9.10	0.12	1.90	4.56	42.0	139.0	91.20	30.0
0.58	8.40	0.21	3.00	5.44	42.0	137.0	78.00	28.0
0.37	6.20	0.11	1.80	4.21	38.0	126.0	90.00	29.9
0.59	7.60	0.22	2.80	5.05	44.0	151.0	87.30	29.9
0.50	6.50	0.48	6.30	4.90	42.0	143.0	85.70	29.2
0.59	5.90	0.12	1.20	5.00	42.0	144.0	84.00	28.8
0.45	8.50	0.51	9.70	4.28	37.0	111.0	85.30	26.0
0.31	8.80	0.32	9.20	4.13	38.0	128.0	92.30	31.0
0.51	4.70	0.27	2.50	5.15	41.0	148.0	85.00	28.7
0.37	6.30	0.21	3.60	5.04	42.0	149.0	84.00	29.6
0.27	5.20	0.18	3.50	4.95	41.0	136.0	82.80	27.6
0.39	8.70	0.23	5.10	4.72	37.0	123.0	79.00	26.3
0.50	7.90	0.35	5.60	4.93	43.0	142.0	87.40	28.8
0.32	7.10	0.26	5.80	4.47	38.0	131.0	84.00	29.3
0.72	7.30	0.19	1.90	5.64	44.0	145.0	78.20	25.7
1.15	8.40	0.01	0.10	5.55	48.0	163.0	85.60	29.4
0.51	9.50	0.20	3.70	4.57	43.0	145.0	93.70	31.7
0.50	8.20	0.12	1.90	4.55	41.0	134.0	83.50	27.6
0.48	7.60	0.11	1.70	5.50	47.0	116.0	84.70	28.4
0.40	5.20	0.25	3.30	4.51	40.0	137.0	89.00	30.4
0.44	13.30	0.03	0.90	4.52	42.0	140.0	92.00	31.0
0.51	9.10	0.11	2.00	4.30	40.0	138.0	93.00	32.1
0.30	5.60	0.09	1.70	4.55	40.0	137.0	88.60	30.1
0.50	6.80	0.27	3.70	4.93	39.0	129.0	79.00	26.2
0.63	7.70	0.18	2.20	5.03	41.0	139.0	82.30	27.6
0.61	8.30	0.25	3.40	4.69	39.0	128.0	82.30	25.9
0.24	6.20	0.13	3.30	5.42	47.0	157.0	86.00	29.0
0.48	5.10	0.06	0.60	5.36	43.0	147.0	82.00	27.5
0.57	9.60	0.21	3.60	4.73	40.0	123.0	85.40	28.0
0.76	6.00	0.24	1.90	4.88	41.0	141.0	83.30	29.0
0.48	6.90	0.08	1.20	5.53	45.0	145.0	81.70	26.9
0.25	6.50	0.12	3.20	4.20	39.0	131.0	82.00	28.0
0.64	8.50	0.14	1.90	4.93	42.0	141.0	84.00	28.0
0.25	5.50	0.51	11.20	4.70	41.0	137.0	87.00	29.1
0.30	5.40	0.31	5.60	4.90	40.0	138.0	82.40	28.2
0.26	4.30	0.09	1.50	4.64	39.0	130.0	83.80	28.0
0.23	6.10	0.13	3.40	4.48	38.0	132.0	85.30	29.6
0.87	11.80	0.28	3.80	5.13	44.0	143.0	85.40	28.0
0.28	7.70	0.06	1.60	4.85	42.0	137.0	86.00	28.2
0.48	8.00	0.13	2.20	4.69	41.0	138.0	85.00	29.0
0.30	4.90	0.08	1.30	5.66	42.0	142.0	75.00	28.0
0.22	4.60	0.11	2.30	4.87	42.0	142.0	85.00	29.2
0.22	4.90	0.15	3.30	4.94	40.0	141.0	80.60	28.5
0.31	4.80	0.01	0.20	3.98	38.0	111.0	83.00	27.9
0.46	10.40	0.08	1.80	4.62	41.0	139.0	87.00	30.1
0.37	7.90	0.12	2.60	5.16	44.0	150.0	85.00	29.1
0.20	4.20	0.04	0.80	4.52	40.0	135.0	89.40	29.9
0.38	5.50	0.07	1.00	4.98	45.0	146.0	89.40	29.3
0.50	5.60	0.14	1.60	4.77	41.0	142.0	86.00	29.8
0.43	7.10	0.17	2.80	4.59	42.0	139.0	90.80	30.3
0.41	7.50	0.07	1.30	3.91	35.0	116.0	90.30	29.7
0.32	5.20	0.10	1.60	5.01	45.0	150.0	90.60	29.9
0.53	8.10	0.23	3.50	5.43	47.0	153.0	85.80	28.2

0.62	7.20	0.30	3.50	5.02	46.0	148.0	88.00	29.3
0.44	6.20	0.25	3.50	5.06	47.0	151.0	92.50	29.8
0.31	6.20	0.13	2.60	4.91	39.0	132.0	79.00	26.9
0.28	8.00	0.02	0.60	4.30	45.0	120.0	81.00	27.0
0.92	9.00	0.03	0.30	4.63	40.0	136.0	85.60	29.4
1.06	5.70	0.34	1.80	5.45	34.0	103.0	62.40	18.9
0.29	6.40	0.05	1.10	2.82	20.0	55.0	69.00	19.5
0.46	6.90	0.17	2.50	4.60	37.0	122.0	80.00	26.6
0.38	7.20	0.17	3.20	4.50	38.0	125.0	86.00	28.0
0.49	8.50	0.05	0.90	3.00	23.0	64.0	77.70	31.3
0.39	8.60	0.09	2.10	4.50	46.0	126.0	82.00	32.1
0.54	4.60	0.02	0.20	5.00	41.0	141.0	82.00	28.2
0.84	4.10	12.36	60.30	5.57	49.0	155.0	88.00	27.8
0.38	7.80	0.11	2.30	4.53	42.0	139.0	92.10	30.7
1.31	6.40	0.04	0.20	4.64	42.0	139.0	89.40	30.0
0.47	8.00	0.19	3.30	4.39	40.0	127.0	90.70	28.8
0.34	8.10	0.16	3.70	3.50	36.0	102.0	83.40	29.2
0.48	7.20	0.04	0.60	4.95	36.0	113.0	73.50	22.8
0.55	8.40	0.07	1.10	4.28	38.0	119.0	87.60	27.8
0.54	10.50	0.07	1.40	5.10	46.0	155.0	91.00	30.4
0.28	8.00	0.19	5.40	4.10	43.0	121.0	83.00	29.0
1.97	13.70	0.14	1.00	4.55	38.0	127.0	83.50	27.9
0.38	6.20	0.15	2.40	4.50	39.2	135.0	89.00	28.2
0.77	3.90	0.00	0.00	4.23	42.0	138.0	99.80	32.0
0.26	6.10	0.40	9.30	4.41	37.0	124.0	84.40	28.1
0.47	5.90	0.24	3.00	5.21	45.0	151.0	86.00	29.0
0.50	9.30	0.22	4.10	4.74	40.0	136.0	81.60	28.7
0.67	9.10	0.00	0.00	3.39	30.0	102.0	88.50	30.1
0.32	6.10	0.13	2.50	4.52	39.0	134.0	85.80	29.6
0.25	8.60	0.06	2.10	4.23	37.0	121.0	87.90	28.6
0.60	7.10	0.02	0.20	4.47	39.0	128.0	87.90	28.6
0.92	7.50	0.63	5.10	4.56	42.0	152.0	89.20	29.0
0.44	7.34	0.06	1.00	2.66	22.0	64.0	81.20	24.1
0.20	8.00	0.00	0.00	4.65	39.0	131.0	84.30	28.2
1.37	11.04	0.05	0.40	4.90	47.0	158.0	95.50	32.2
0.34	6.80	0.21	4.20	4.70	38.0	129.0	87.00	27.4
0.31	7.80	0.11	2.80	4.91	42.0	145.0	84.60	29.5
0.46	4.40	0.00	0.00	4.73	31.0	90.0	66.50	19.0
0.49	4.60	0.01	0.10	4.81	41.0	136.0	84.40	28.3
0.75	10.40	0.23	3.20	5.79	47.0	151.0	81.20	26.1
0.34	8.40	0.03	0.70	4.63	42.0	143.0	90.50	30.9
0.32	5.40	0.07	1.20	4.89	42.0	138.0	85.30	28.2
0.55	9.40	0.14	2.40	5.07	43.0	140.0	84.00	27.0
0.69	9.70	0.15	2.10	4.77	42.0	140.0	88.90	29.1
0.49	5.40	0.09	1.00	5.64	36.0	113.0	63.60	20.0
0.46	7.20	0.23	3.60	4.78	41.0	135.0	84.90	28.2
0.50	9.60	0.06	1.10	4.82	41.0	136.0	85.50	28.2
0.43	7.20	0.24	4.00	4.55	41.0	142.0	90.30	31.2
0.29	5.40	0.14	2.60	5.04	44.0	142.0	85.60	28.2
0.36	5.60	0.12	1.90	4.56	40.0	128.0	87.90	28.1
0.31	5.90	0.15	2.90	4.76	40.0	123.0	84.00	25.8
0.20	4.80	0.04	1.00	5.06	42.0	136.0	84.00	26.9
0.19	7.00	0.06	2.20	3.18	24.0	71.0	74.20	22.3
0.34	5.40	0.04	0.60	4.79	41.0	135.0	85.80	28.2

0.35	6.10	0.24	4.20	4.92	40.0	136.0	81.30	27.6
0.60	7.60	0.17	2.20	4.98	40.0	132.0	79.90	26.5
0.51	7.50	0.10	1.50	4.34	38.0	125.0	87.00	29.0
0.42	8.00	0.14	2.70	4.97	42.0	138.0	94.40	30.9
0.39	8.20	0.17	3.60	4.97	41.0	144.0	82.00	29.0
0.68	7.30	0.42	4.50	2.85	25.0	78.0	88.40	27.4
0.20	2.50	0.00	0.00	4.96	41.0	140.0	82.70	28.2
0.49	6.90	0.19	2.70	5.48	42.0	138.0	76.50	25.2
0.21	5.80	0.08	2.20	4.38	40.0	136.0	91.30	31.1
0.29	6.70	0.16	3.70	5.26	43.0	143.0	82.10	27.2
0.26	6.30	0.21	5.10	4.10	40.0	135.0	82.50	32.1
0.19	5.30	0.14	3.90	4.79	40.0	135.0	82.70	28.2
0.37	8.80	0.10	2.40	4.00	41.0	125.0	82.00	29.0
0.28	6.10	0.04	0.90	4.57	40.0	134.0	87.10	29.3
0.60	8.80	0.24	3.50	4.22	35.0	114.0	82.70	27.0
0.26	6.00	0.06	1.40	4.85	40.0	135.0	82.70	27.8
0.23	3.30	0.11	1.60	6.30	40.0	150.0	88.00	29.3
0.58	6.40	0.11	1.20	5.04	42.0	139.0	83.70	27.6
0.43	5.90	0.12	1.60	5.17	44.0	151.0	85.10	29.2
0.28	5.40	0.07	1.40	4.93	42.0	139.0	84.20	28.2
0.70	7.60	0.06	0.70	4.76	39.0	130.0	82.40	27.3
0.30	5.00	0.16	2.70	4.99	44.0	147.0	87.60	29.5
0.20	4.60	0.04	0.90	4.92	40.0	118.0	81.50	24.0
0.28	5.40	0.21	4.00	5.10	43.8	141.0	85.90	27.6
0.45	7.00	0.22	3.40	5.27	44.9	147.0	85.20	27.9
0.54	8.10	0.31	4.60	4.73	39.7	137.0	83.90	27.9
0.53	8.50	0.09	1.50	5.05	44.0	145.0	87.10	28.7
0.37	7.40	0.03	0.50	4.43	40.5	134.0	91.40	30.2
0.25	5.90	0.10	2.40	4.41	37.9	119.0	85.90	27.0
0.32	3.00	0.31	2.90	4.75	41.1	139.0	86.50	29.3
0.46	4.60	0.25	2.50	4.76	40.5	137.0	85.10	28.8
0.33	6.10	0.09	1.70	4.94	40.8	131.0	82.60	25.5
0.41	6.50	0.07	1.10	4.42	34.5	110.0	78.10	24.9
0.31	8.00	0.09	2.30	3.43	28.5	89.0	83.10	25.9
0.35	6.90	0.71	14.10	4.53	39.7	130.0	87.60	28.7
0.26	5.40	0.11	2.30	5.07	45.2	151.0	89.20	29.8
0.51	6.00	0.16	1.90	5.02	42.8	144.0	85.30	28.7
0.39	8.80	0.09	2.00	4.37	40.5	133.0	92.70	30.4
0.27	6.40	0.09	2.10	5.27	45.8	154.0	86.90	29.2
0.49	7.80	0.13	2.10	4.68	39.8	136.0	85.00	29.1
0.39	4.60	0.17	2.00	4.83	41.6	141.0	86.70	29.2
0.93	9.80	0.02	0.20	4.74	40.8	133.0	86.10	28.1
0.53	8.30	0.59	9.20	5.97	40.0	121.0	67.00	20.3
0.15	5.70	0.06	2.30	4.82	38.2	129.0	79.30	26.8
0.79	5.70	0.14	1.00	3.32	25.7	77.0	77.40	23.2
0.43	5.70	0.20	2.60	4.72	33.0	92.0	69.90	19.5
0.28	6.10	0.25	5.40	4.80	41.1	142.0	85.60	29.6
0.51	9.50	0.18	3.30	5.00	40.8	142.0	81.60	28.4
0.38	6.90	0.19	3.40	4.83	40.7	132.0	84.30	27.3
0.28	7.04	0.18	4.50	4.84	41.5	137.0	85.70	28.3
0.24	5.40	0.12	2.70	4.61	38.3	128.0	83.10	27.8
0.33	5.30	0.17	2.70	5.07	41.8	144.0	82.40	28.4
0.35	4.30	0.08	1.00	4.30	38.0	126.0	88.40	29.3
0.48	6.70	0.68	9.40	4.54	40.5	139.0	89.20	30.6

0.13	1.70	0.09	1.21	3.72	34.2	104.0	91.90	28.0
0.59	6.90	0.08	0.90	5.05	43.1	146.0	85.90	28.9
0.32	8.30	0.08	2.10	4.04	35.7	114.0	88.40	28.2
0.35	7.40	0.11	2.30	4.12	36.5	119.0	88.60	28.9
0.32	4.30	0.13	1.70	5.28	43.8	146.0	83.00	27.7
0.23	4.00	0.11	1.90	4.77	40.8	137.0	85.50	28.7
0.44	9.40	0.17	3.60	4.55	38.7	134.0	85.10	29.5
0.37	5.60	0.35	5.30	4.19	38.0	128.0	91.60	30.5
0.30	6.20	0.26	0.40	4.53	35.0	125.0	77.30	27.6
0.16	2.30	0.01	0.10	4.48	37.0	126.0	81.90	28.1
0.28	6.10	0.10	2.20	4.34	33.0	104.0	75.60	24.0
0.26	5.20	0.10	2.00	4.94	40.0	136.0	81.00	27.5
0.33	5.00	0.22	3.30	4.84	37.0	136.0	76.70	28.1
0.44	4.90	0.07	0.80	4.71	38.0	127.0	79.60	27.0
0.36	5.40	0.10	1.50	4.93	42.0	139.0	85.20	28.2
0.35	7.40	0.06	1.30	4.99	40.0	144.0	81.00	28.9
0.48	6.70	0.10	1.40	5.12	32.0	85.0	61.70	16.6
0.89	6.60	0.00	0.00	5.42	46.0	157.0	84.10	29.0
0.49	6.80	0.19	2.60	4.82	42.0	146.0	87.60	30.3
0.47	4.70	0.01	0.10	5.03	39.0	133.0	76.70	26.4
0.27	3.90	0.14	2.00	4.85	40.0	135.0	82.30	27.8
0.36	6.20	0.27	4.60	5.06	43.0	153.0	85.00	30.2
0.40	4.40	0.07	0.50	5.04	42.0	142.0	83.00	28.0
0.30	4.20	0.18	2.80	5.06	42.0	147.0	83.00	29.0
0.30	6.30	0.06	1.30	4.91	42.0	143.0	86.00	29.0
0.40	7.70	0.11	1.90	4.47	39.0	138.0	88.00	31.0
0.80	4.30	0.37	2.00	4.28	37.0	127.0	86.00	30.0
0.40	7.10	0.21	3.30	5.00	42.0	144.0	84.00	28.8
0.50	6.50	0.44	5.90	4.88	41.0	142.0	84.60	29.1
0.40	6.80	0.08	1.40	4.80	41.0	140.0	85.60	29.2
0.20	3.00	0.01	0.01	3.45	32.0	105.0	93.00	30.0
0.39	3.30	0.00	0.00	4.68	41.0	134.0	86.80	28.6
0.22	4.70	0.31	6.60	4.70	39.0	128.0	82.10	27.2
1.26	6.60	0.12	0.60	4.97	37.0	120.0	74.80	24.1
0.55	8.30	0.21	3.20	5.08	43.0	142.0	84.60	28.0
#NULL!	#NULL!	#NULL!	#NULL!	#NULL!	#NULL!	#NULL!	#NULL!	#NULL!
0.22	4.80	0.14	3.00	5.16	47.0	155.0	91.50	30.0
0.33	6.20	0.11	2.10	4.50	38.0	126.0	85.10	28.0
0.39	7.70	0.10	2.00	4.72	40.0	132.0	83.70	28.0
0.41	6.90	0.13	2.20	3.04	26.1	85.0	85.90	28.0
0.54	5.90	0.21	2.30	4.43	37.0	125.0	84.20	28.2
0.50	7.20	0.41	5.90	4.78	40.0	141.0	83.50	29.5
0.61	12.90	0.13	2.70	5.96	43.0	145.0	71.50	24.3
0.21	4.30	0.24	5.00	4.74	40.0	139.0	85.20	29.3
0.28	5.80	0.15	3.10	4.27	39.0	127.0	90.20	29.7
0.32	7.20	0.16	3.60	4.96	43.0	140.0	85.90	28.2
0.28	5.60	0.08	1.60	4.35	40.0	131.0	90.80	30.1
0.35	7.70	0.21	4.60	4.97	41.0	135.0	82.70	27.2
0.57	7.70	0.50	6.70	5.05	42.0	145.0	83.40	28.7
0.35	6.20	0.16	2.80	4.75	41.0	139.0	85.90	29.3
0.39	5.50	0.16	2.30	5.19	42.0	148.0	80.20	28.5
1.08	4.60	0.11	0.50	4.23	34.7	117.0	82.00	27.7
0.31	8.90	0.06	1.70	4.40	39.0	130.0	89.30	29.5
0.84	8.40	0.29	2.90	4.95	42.0	136.0	84.20	27.5

0.40	6.80	0.08	1.40	4.52	40.0	132.0	88.50	29.2
0.25	2.90	0.01	0.10	4.15	35.0	120.0	83.10	28.9
0.31	4.70	0.35	5.30	4.38	41.0	138.0	92.50	31.5
0.31	6.30	0.12	2.40	4.70	40.0	135.0	84.90	28.7
0.19	4.80	0.09	2.30	4.24	38.0	123.0	89.20	29.0
0.49	8.40	0.36	6.10	4.22	35.0	118.0	82.90	28.0
0.44	8.40	0.18	3.50	4.84	44.0	142.0	90.30	29.3
0.55	8.40	0.13	2.00	4.83	43.0	139.0	88.80	28.8
0.39	5.40	0.13	1.80	5.35	46.0	150.0	86.50	28.0
0.18	4.20	0.41	9.50	4.84	43.0	147.0	89.70	30.4
0.37	6.90	0.15	2.80	4.94	41.0	130.0	82.40	26.3
0.44	6.50	0.18	2.70	4.86	38.0	126.0	78.60	25.9
0.54	9.90	0.06	1.10	6.04	37.0	114.0	60.80	18.9
0.47	8.00	0.22	3.80	4.97	42.0	137.0	84.50	27.6
0.58	10.00	0.38	6.60	5.54	36.0	105.0	64.80	19.0
0.41	37.70	0.10	1.70	5.10	42.7	145.0	83.70	28.4
0.44	8.90	0.11	2.20	4.80	42.0	134.0	86.90	27.9
0.41	9.20	0.08	1.80	4.98	43.0	144.0	87.10	28.9
0.47	9.00	0.27	5.10	4.36	39.0	129.0	89.40	29.6
0.51	8.40	0.14	2.30	4.35	41.0	131.0	93.60	30.1
0.46	7.60	0.38	6.30	4.61	40.0	137.0	87.00	29.7
0.43	6.50	0.27	4.10	4.30	41.0	138.0	94.20	32.1
0.33	5.00	0.11	1.70	5.30	46.0	144.0	86.40	27.2
1.12	6.00	0.02	#NULL!	4.95	42.6	147.0	86.10	29.7
0.27	6.70	0.07	1.70	4.76	43.0	138.0	89.50	29.0
0.41	6.60	0.05	0.80	5.10	45.0	151.0	89.00	29.6
0.40	5.70	0.22	3.10	5.77	38.0	113.0	65.30	19.6
0.36	6.80	0.28	5.30	4.98	44.0	145.0	88.80	29.1
0.88	7.20	0.27	2.20	4.69	38.0	119.0	80.00	25.4
0.59	5.00	0.10	0.80	5.26	44.0	149.0	84.00	28.3
0.56	6.70	0.34	4.10	5.02	42.0	141.0	84.50	28.1
0.37	9.90	0.01	0.30	3.19	27.0	91.0	85.60	28.5
0.44	9.00	0.12	2.40	4.60	37.0	127.0	80.00	27.6
0.61	8.00	0.01	0.10	4.09	36.0	121.0	87.50	29.6
6.20	6.20	0.07	1.30	4.89	45.0	152.0	92.20	31.1
0.79	13.30	0.15	2.50	4.86	43.0	142.0	87.90	29.2
0.48	10.90	0.00	0.00	2.87	23.4	78.0	81.50	27.2
0.22	4.70	0.11	2.40	5.03	45.0	144.0	88.70	28.6
0.15	3.60	0.07	1.70	4.45	39.0	129.0	88.50	29.0
0.86	4.90	0.16	0.90	4.67	37.0	129.0	79.20	27.6
0.39	7.20	0.30	5.50	4.00	36.0	116.0	89.80	29.0
0.24	5.60	0.19	4.50	4.93	42.0	146.0	85.20	29.6
0.25	4.90	0.09	1.80	4.48	40.9	143.0	91.30	31.9
0.51	7.40	0.15	2.20	4.99	43.9	144.0	88.00	28.9
0.39	4.80	0.01	0.10	3.67	32.3	113.0	88.00	30.8
0.53	6.70	0.11	1.40	5.04	40.3	139.0	80.00	27.6
1.09	4.80	0.00	0.00	5.44	44.3	146.0	81.40	26.8
0.55	4.60	0.02	0.20	4.72	40.3	142.0	85.40	30.1
0.22	3.90	0.26	4.60	5.04	42.5	140.0	84.30	27.8
0.49	6.40	0.09	1.20	5.53	46.2	165.0	83.50	29.8
0.43	7.80	0.10	1.80	5.21	47.0	167.0	90.20	32.1
0.49	7.20	0.10	1.50	4.69	37.1	126.0	79.10	26.9
0.24	7.50	0.08	2.50	4.14	34.8	122.0	84.10	29.5
0.28	5.30	0.24	4.50	5.34	45.8	151.0	85.80	28.3

0.16	3.70	0.00	0.00	4.08	35.6	123.0	87.30	30.1
0.25	5.70	0.07	1.60	4.70	41.1	140.0	87.40	29.8
0.41	6.50	0.15	2.40	5.16	41.3	135.0	80.00	26.2
0.66	8.50	0.20	2.60	4.90	42.5	136.0	86.70	27.8
0.29	4.30	0.51	7.50	4.86	41.2	135.0	84.80	27.8
0.39	8.30	0.12	2.50	4.40	37.8	128.0	85.90	29.1
0.56	6.20	0.00	0.00	5.92	53.0	175.0	89.00	29.6
0.60	5.90	0.06	0.60	5.49	47.6	162.0	86.70	29.5
0.96	8.20	0.00	0.00	3.96	35.0	125.0	89.10	31.6
0.26	6.00	0.12	2.80	5.46	37.0	115.0	67.60	21.1
0.47	12.50	0.12	3.20	4.57	37.7	127.0	82.50	27.8
0.35	3.90	0.12	1.30	4.89	42.0	148.0	86.70	30.3
0.37	7.80	0.22	4.60	4.90	40.3	139.0	82.20	28.4
0.29	2.70	0.01	0.10	2.48	18.7	58.0	75.40	23.4
0.40	4.90	0.15	1.90	5.41	43.0	143.0	79.50	26.4
0.45	5.90	0.32	4.20	4.81	40.1	131.0	83.40	27.2
0.58	9.00	0.20	3.10	5.48	47.1	163.0	85.90	29.7
0.36	6.90	0.08	1.50	4.64	39.6	130.0	85.30	28.0
0.58	7.20	0.07	0.90	5.07	45.2	148.0	89.20	29.2
0.31	5.70	0.42	7.70	4.46	39.2	129.0	87.90	28.9
0.25	4.50	0.08	1.40	5.81	38.5	123.0	66.30	21.2
0.33	3.90	0.02	0.20	4.79	43.3	141.0	90.40	29.4
0.28	3.90	0.32	4.50	5.24	42.2	143.0	80.50	27.3
0.34	5.40	0.05	0.80	4.69	39.0	129.0	83.20	27.5
0.26	5.30	0.04	0.80	5.30	44.7	152.0	84.30	28.7
0.42	5.90	0.24	3.40	5.75	48.3	164.0	84.00	28.5
0.40	6.20	0.34	5.20	5.37	44.1	146.0	82.10	27.2
0.51	8.60	0.14	2.40	5.17	44.4	148.0	85.90	28.6
0.55	10.40	0.21	4.00	4.59	40.2	136.0	87.60	29.6
0.33	5.40	0.12	2.00	4.58	41.0	137.0	89.50	29.9
0.32	5.20	0.06	1.00	5.15	43.4	157.0	84.30	30.5
0.74	7.80	0.23	2.40	4.90	40.7	144.0	83.10	29.4
0.26	6.50	0.09	2.20	4.74	40.8	135.0	86.10	28.5
0.45	8.30	0.71	13.20	5.19	43.0	135.0	82.90	26.0
0.47	5.10	0.65	7.10	4.73	39.5	139.0	83.50	29.4
0.43	5.80	0.20	2.70	4.60	43.0	141.0	93.50	30.7
0.43	6.40	0.21	3.10	5.02	39.9	134.0	79.50	26.7
0.57	6.00	0.89	9.40	5.51	42.7	139.0	77.50	25.2
0.38	5.40	0.28	4.00	5.18	42.6	143.0	82.20	27.6
0.39	6.60	0.19	3.20	4.69	41.1	137.0	87.60	29.2
0.42	5.30	0.19	2.40	4.47	42.3	142.0	94.60	31.8
0.24	4.40	0.25	4.60	4.86	39.9	139.0	82.10	28.6
0.32	7.40	0.26	6.00	4.43	39.6	132.0	89.40	29.8
0.52	7.70	0.04	0.60	4.66	39.8	137.0	85.40	29.4
0.53	8.40	0.19	3.00	5.08	40.7	126.0	80.10	24.8
0.23	3.50	0.07	1.10	4.67	40.0	141.0	86.30	30.2
0.55	8.90	0.26	4.20	5.40	46.8	159.0	86.70	29.4
0.38	8.00	0.23	4.80	5.18	46.1	160.0	89.00	30.9
0.37	7.90	0.19	4.10	4.44	39.4	135.0	88.70	30.4
0.20	4.00	0.11	2.20	4.45	39.9	131.0	89.70	29.4
0.42	7.70	0.30	5.50	4.46	38.0	124.0	85.90	27.8
0.34	6.50	0.31	5.90	4.60	40.9	138.0	88.90	30.0
0.28	5.50	0.03	0.60	5.24	31.6	82.0	60.30	15.6
0.45	5.90	0.06	0.80	4.95	41.7	143.0	84.20	28.9

#NULL!	#NULL!	#NULL!	#NULL!	#NULL!	#NULL!	#NULL!	#NULL!	#NULL!
0.39	5.90	0.14	2.10	4.85	38.5	140.0	79.40	28.9
0.26	5.00	0.35	6.70	4.71	40.2	137.0	85.40	29.1
0.37	5.10	0.16	2.20	5.34	42.9	137.0	80.30	25.7
0.42	6.40	0.09	1.40	4.19	40.1	135.0	95.70	32.2
7.30	0.47	0.14	2.20	5.29	44.1	154.0	83.40	29.1
0.29	4.70	0.13	2.10	4.75	44.7	143.0	94.10	30.1
0.70	9.10	1.03	13.40	4.57	40.0	135.0	87.50	29.5
0.41	19.20	0.01	0.50	3.07	34.7	114.0	113.00	37.1
0.78	7.90	0.11	1.10	4.72	39.1	126.0	82.80	26.7
0.26	6.30	0.05	1.20	5.26	45.4	154.0	86.30	29.3
0.43	5.70	0.13	1.70	5.12	44.0	151.0	85.90	29.5
0.34	8.00	0.04	0.90	4.85	40.3	135.0	83.10	27.8
0.33	4.20	0.30	3.80	4.87	39.0	136.0	80.10	27.9
0.62	6.50	0.20	2.10	2.86	23.2	82.0	81.10	28.7
0.28	8.10	0.29	8.40	3.92	27.7	82.0	70.70	20.9
0.44	5.70	0.21	2.70	4.88	42.4	144.0	86.90	29.5
0.31	5.00	0.16	2.60	5.17	42.5	152.0	82.20	29.4
0.53	6.80	0.08	1.00	5.29	43.4	145.0	82.00	27.4
0.42	5.70	0.29	3.90	4.11	36.1	119.0	87.80	29.0
0.38	4.80	0.13	1.60	5.50	42.2	139.0	76.70	25.3
0.54	9.20	0.15	2.50	5.27	48.1	163.0	91.30	30.9
0.27	4.30	0.66	10.60	4.57	40.4	139.0	88.40	30.4
0.60	4.90	0.10	0.80	5.15	42.1	135.0	81.70	26.2
0.54	4.80	0.05	0.40	2.30	20.3	62.0	88.30	27.0
0.65	7.70	0.22	2.60	3.85	35.4	109.0	91.90	28.3
0.66	8.90	0.28	3.80	4.68	41.5	137.0	88.70	29.3
0.68	8.40	0.18	2.20	5.26	40.5	134.0	77.00	25.5
0.51	4.40	3.40	29.30	5.03	41.7	141.0	82.90	28.0
0.24	6.40	0.04	1.10	4.27	35.7	131.0	83.60	30.7
0.27	5.90	0.05	1.10	4.60	41.5	132.0	90.20	28.7
0.19	3.80	0.38	7.70	4.99	43.0	145.0	87.00	29.1
0.54	5.80	0.09	1.00	5.33	43.6	145.0	81.80	27.2
0.89	9.50	0.28	3.00	4.53	38.1	126.0	84.10	27.8
0.41	5.80	0.04	0.60	4.65	38.7	137.0	83.20	29.5
0.43	6.50	0.29	4.40	4.88	39.5	134.0	80.90	27.5
0.37	7.00	0.04	0.80	5.02	38.4	142.0	76.50	28.3
0.42	9.00	0.10	2.20	4.87	40.5	142.0	83.20	29.2
0.46	9.30	0.11	2.20	5.45	43.7	152.0	80.20	27.9
0.26	4.90	0.05	0.90	4.42	36.2	126.0	81.90	28.5
1.01	7.30	0.20	1.50	3.64	28.7	92.0	78.80	25.3
0.31	5.10	0.16	2.60	4.74	39.0	135.0	82.30	28.5
0.39	6.90	0.26	4.60	5.24	43.9	144.0	83.80	27.5
0.37	5.80	0.08	1.30	4.40	39.3	132.0	89.30	30.0
0.50	10.00	0.06	1.20	4.36	38.1	132.0	87.40	30.3
0.60	10.30	0.16	2.70	4.24	37.5	131.0	88.40	30.9
0.36	7.20	0.05	1.00	4.65	38.5	126.0	82.80	27.1
0.44	11.10	0.28	7.00	5.21	43.5	151.0	83.50	29.0
0.40	6.20	0.18	2.80	5.45	47.7	153.0	87.50	28.1
0.45	4.80	0.07	0.70	3.99	37.3	124.0	93.50	31.1
0.67	7.60	0.10	1.10	5.45	43.1	146.0	79.10	26.8
0.26	4.90	0.09	1.70	4.82	40.8	145.0	84.60	30.1
0.59	5.90	0.17	1.70	4.58	38.6	139.0	84.30	30.3
0.39	3.80	0.14	1.40	4.73	39.9	137.0	84.40	29.0

0.31	5.70	0.18	3.30	5.07	45.1	153.0	89.00	30.2
0.28	6.50	0.08	1.90	4.42	39.9	130.0	90.30	29.4
0.38	5.60	0.48	7.00	5.11	42.7	149.0	83.60	29.2
0.42	6.40	0.09	1.40	5.17	43.9	152.0	84.90	29.4
0.34	7.20	0.24	5.10	4.65	42.2	145.0	90.80	31.2
0.33	8.30	0.11	2.80	4.72	40.3	139.0	85.40	29.4
0.72	8.10	0.20	2.30	5.16	44.3	158.0	85.90	30.6
0.40	7.80	0.06	1.20	4.37	38.9	129.0	89.00	29.5
0.38	8.10	0.09	1.90	4.87	41.3	147.0	84.80	30.2
0.27	5.00	0.05	0.90	4.25	37.2	126.0	87.50	29.6
0.51	7.40	0.12	1.80	4.78	42.7	139.0	89.30	29.1
0.34	5.40	0.06	1.00	4.33	39.1	130.0	90.30	30.0
0.31	5.90	0.14	2.70	5.23	43.8	149.0	83.70	28.5
0.32	5.70	0.39	7.00	5.47	47.6	151.0	87.00	27.6
0.63	11.80	0.29	5.40	4.83	39.2	137.0	81.20	28.4
0.37	5.90	0.18	2.90	4.73	41.9	150.0	88.60	31.7
0.35	8.40	0.12	2.90	5.38	42.9	152.0	79.70	28.3
0.43	6.70	0.03	0.50	2.52	18.3	44.0	72.60	17.5
0.97	12.30	0.21	2.70	5.59	48.6	166.0	86.90	29.7
#NULL!	6.10	#NULL!	#NULL!	5.05	#NULL!	148.0	#NULL!	#NULL!
0.59	8.10	0.30	4.10	4.85	45.2	150.0	93.20	30.9
0.70	9.70	0.12	1.70	4.81	40.2	144.0	83.60	29.9
0.55	9.00	0.28	4.60	4.98	40.1	131.0	80.50	26.3
0.60	9.50	0.33	5.20	4.12	37.2	133.0	90.30	32.3
0.24	6.40	0.31	8.30	5.87	36.2	117.0	61.70	19.9
0.88	6.60	0.16	1.20	4.71	40.4	137.0	86.80	29.1
0.24	8.10	0.04	1.40	2.96	23.3	79.0	78.70	26.7
0.47	7.00	0.09	1.30	4.35	36.1	127.0	83.00	29.2
0.31	4.50	0.10	1.50	4.83	41.1	136.0	85.10	28.2
0.57	7.60	0.11	1.50	4.56	40.5	139.0	88.80	30.5
0.42	6.00	0.15	2.20	4.57	38.9	128.0	85.10	28.0
0.29	7.00	0.07	1.70	4.49	38.4	130.0	85.50	29.0
0.97	15.00	0.05	0.80	4.20	35.3	122.0	84.00	29.0
0.47	5.60	0.31	3.70	5.22	43.3	159.0	83.00	30.5
0.38	4.50	0.13	1.50	4.76	39.7	134.0	83.40	28.2
0.87	7.00	0.20	1.60	4.37	38.7	125.0	88.60	28.6
0.39	5.00	0.10	1.30	4.86	40.5	140.0	83.30	28.8
0.32	5.60	0.14	2.50	5.25	45.6	156.0	86.90	29.7
0.45	5.20	0.03	0.30	5.56	46.0	156.0	82.70	28.1
0.51	9.60	0.03	0.60	5.07	42.7	145.0	84.20	28.6
0.41	6.30	0.10	1.50	4.17	37.0	130.0	88.70	31.2
0.14	6.10	0.02	0.90	1.19	12.0	38.0	100.80	31.9
0.40	9.40	0.23	5.40	4.99	34.9	99.0	69.90	19.8
0.57	7.40	0.60	7.80	5.04	40.2	140.0	79.80	27.8
0.83	8.80	0.01	0.10	5.30	44.3	155.0	83.60	29.2
0.42	6.20	0.18	2.70	4.71	41.9	147.0	89.00	31.2
0.72	11.10	0.20	3.10	4.21	36.4	120.0	86.50	28.5
0.57	8.40	0.18	2.60	4.09	36.3	121.0	88.80	29.6
0.48	7.60	0.02	0.30	4.72	44.3	148.0	93.90	31.4
1.50	5.20	0.22	0.80	4.83	43.0	148.0	89.00	30.6
0.16	1.60	0.21	2.00	4.25	38.9	127.0	91.50	29.9
0.46	7.10	0.08	1.20	5.08	38.4	129.0	75.60	25.4
0.43	6.10	0.06	0.80	5.23	41.8	137.0	79.90	26.2
0.37	4.00	0.08	0.90	4.89	41.2	138.0	84.30	28.2

0.33	4.30	0.13	1.70	4.93	39.9	135.0	80.90	27.4
0.39	6.90	0.06	1.10	4.00	34.1	117.0	85.30	29.3
0.61	5.50	0.16	1.40	4.74	40.3	129.0	85.00	27.2
0.49	1.50	21.28	63.90	5.37	44.6	153.0	83.10	28.5
0.72	7.40	0.65	6.70	5.55	40.5	130.0	73.00	23.4
0.54	6.40	0.30	3.60	4.45	37.0	122.0	83.10	27.4
0.68	6.40	0.44	4.10	5.08	43.2	147.0	85.00	28.9
0.52	5.90	0.13	1.50	4.96	44.5	150.0	89.70	30.2
0.52	7.00	0.33	4.40	5.25	45.8	160.0	87.20	30.5
0.49	7.00	0.40	5.70	6.01	48.1	159.0	80.00	26.5
0.37	6.30	0.29	5.00	4.97	42.7	147.0	85.90	29.6
0.36	7.00	0.39	7.60	5.01	40.9	137.0	81.60	27.3
#NULL!	#NULL!	#NULL!	#NULL!	4.88	#NULL!	150.0	#NULL!	#NULL!
0.52	6.90	0.13	1.70	4.63	39.7	131.0	85.70	28.3
0.66	6.30	0.09	0.90	4.77	39.5	134.0	82.80	28.1
0.38	5.00	0.12	1.60	4.19	32.9	97.0	78.50	23.2
0.51	4.70	0.19	1.70	5.47	46.3	161.0	84.60	29.4
0.31	3.10	0.08	0.80	4.48	38.0	127.0	84.80	28.3
0.70	7.70	0.04	0.40	5.02	43.7	149.0	87.40	28.2
0.26	3.70	0.03	0.40	4.87	39.7	139.0	81.50	28.5
0.46	8.00	0.11	1.90	3.85	27.6	88.0	71.70	22.9
0.51	5.80	0.04	0.50	4.92	41.4	140.0	84.10	28.5
0.23	2.60	0.01	0.10	5.01	44.9	157.0	89.60	31.3
0.49	5.90	0.04	0.50	4.52	42.0	138.0	92.90	30.5
0.55	8.30	0.13	2.00	5.07	38.0	132.0	75.00	26.0
0.18	4.80	0.11	2.90	4.97	42.5	142.0	85.50	28.6
0.46	11.00	0.05	1.20	2.64	24.4	80.0	92.40	30.3
0.49	6.60	0.39	5.20	4.53	39.3	131.0	86.80	28.9
0.49	6.20	0.15	1.90	4.19	37.2	126.0	88.80	30.1
0.42	6.20	0.21	3.10	6.01	39.2	122.0	65.20	20.3
0.26	4.80	0.16	3.00	5.24	48.1	154.0	91.80	29.4
0.46	7.50	0.06	1.00	4.73	40.3	139.0	85.20	29.4
0.42	8.20	0.08	1.60	5.06	44.7	151.0	88.30	29.8
0.46	7.00	0.27	4.10	4.74	37.5	122.0	79.10	25.7
0.48	8.10	0.06	1.00	5.15	45.9	161.0	89.10	31.3
0.52	4.70	0.08	0.70	5.22	41.5	136.0	79.50	26.1
0.27	7.00	0.05	1.30	4.85	41.2	136.0	84.90	28.0
1.01	31.30	0.01	0.30	5.12	41.3	140.0	80.70	27.3
0.30	6.40	0.06	1.30	4.53	38.6	131.0	85.20	28.9
0.30	4.60	0.13	2.00	5.06	42.4	148.0	83.80	29.2
0.47	7.60	0.10	1.60	5.12	44.7	146.0	87.30	28.5
1.10	4.40	0.03	0.10	5.64	45.8	162.0	81.20	28.7
0.25	4.90	0.08	1.60	4.90	41.7	141.0	85.10	28.8
0.75	7.10	0.24	2.30	5.18	45.2	150.0	87.30	29.0
0.25	4.80	0.07	1.40	5.40	43.3	138.0	80.20	25.6
0.41	5.00	0.44	5.40	5.40	40.1	139.0	82.50	28.6
0.48	5.10	0.23	2.40	4.55	41.3	133.0	90.80	29.2
0.38	6.10	0.11	1.80	4.54	38.8	133.0	85.50	29.3
0.44	9.80	0.07	1.60	4.53	39.1	134.0	86.30	29.6
0.00	0.00	0.00	0.00	2.94	23.9	88.0	81.30	29.9
0.22	4.20	0.03	0.60	4.86	39.6	136.0	81.50	28.0
0.33	4.50	0.15	2.10	4.54	42.4	138.0	93.40	30.4
0.24	4.30	0.11	2.00	4.97	41.6	142.0	83.70	28.6
0.47	8.90	0.05	0.90	4.29	38.3	127.0	89.30	29.6

0.48	8.00	0.08	1.30	4.81	41.1	139.0	85.40	28.9
0.52	6.60	0.09	1.10	4.72	41.1	142.0	87.10	30.1
0.28	3.30	0.24	2.90	4.61	40.0	139.0	86.80	30.2
0.42	6.40	0.06	0.90	5.42	46.2	157.0	85.20	29.0
0.48	8.80	0.42	7.70	4.52	38.3	130.0	84.70	28.8
0.43	6.90	0.37	6.00	4.62	41.0	140.0	88.70	30.3
0.49	4.90	0.09	0.90	4.75	41.6	138.0	87.60	29.1
0.31	4.30	0.09	1.20	4.58	41.6	135.0	90.80	29.5
0.51	8.20	0.27	4.30	5.43	46.8	163.0	86.20	30.0
0.65	8.40	0.09	1.20	4.43	35.4	118.0	79.90	26.6
0.48	6.30	0.05	0.70	4.58	41.9	142.0	91.50	31.0
0.40	7.00	0.06	1.00	4.70	40.8	139.0	86.80	29.6
0.89	12.50	0.21	2.90	4.61	42.3	141.0	91.80	30.6
0.33	5.50	0.14	2.30	4.46	36.6	122.0	82.10	27.4
0.32	4.60	0.17	2.40	5.97	48.4	162.0	81.10	27.1
0.31	4.20	0.44	6.00	4.90	38.6	133.0	78.80	27.1
0.51	5.90	0.35	4.10	5.00	45.8	151.0	91.60	30.2
0.47	4.70	0.12	1.20	6.02	39.7	126.0	65.90	20.9
0.83	5.70	0.15	1.00	5.15	43.7	143.0	84.90	27.8
0.42	6.80	0.36	5.80	4.93	40.1	130.0	81.30	26.4
0.34	6.20	0.03	0.50	4.75	38.8	134.0	81.70	28.2
0.31	4.40	0.07	1.00	4.85	41.7	144.0	86.00	29.7
0.76	9.30	0.18	2.20	5.09	40.2	144.0	79.00	28.3
0.29	4.60	0.07	1.10	4.40	39.2	131.0	89.10	29.8
0.26	4.50	0.10	1.70	5.37	46.9	153.0	87.30	28.5
0.36	6.60	0.08	1.50	5.48	46.4	162.0	84.70	29.6
0.77	9.00	0.12	1.40	3.52	28.9	94.0	82.10	26.7
0.78	8.30	0.04	0.40	4.07	26.7	77.0	65.60	18.9
0.54	6.50	0.87	10.50	5.00	43.1	143.0	86.20	28.6
0.49	6.40	0.18	2.40	4.25	36.0	125.0	84.70	29.4
0.41	8.50	0.10	2.10	3.88	31.9	101.0	82.20	26.0
0.42	4.80	0.32	3.70	5.16	43.2	146.0	83.70	28.3
0.22	3.40	0.08	1.20	4.23	36.7	125.0	86.80	29.6
#NULL!	#NULL!	#NULL!	#NULL!	#NULL!	#NULL!	#NULL!	#NULL!	#NULL!
0.34	7.30	0.14	3.00	5.09	41.5	136.0	81.50	26.7
0.61	7.60	0.19	2.40	4.94	41.8	141.0	84.60	28.5
0.86	6.00	0.11	0.80	4.57	37.2	131.0	81.40	28.7
0.50	5.10	0.20	2.00	3.28	26.1	82.0	79.60	25.0
#NULL!	#NULL!	#NULL!	#NULL!	5.35	#NULL!	141.0	#NULL!	#NULL!
0.70	8.50	0.09	1.10	5.15	44.5	150.0	86.40	29.1
0.49	4.60	0.56	5.30	5.36	41.2	133.0	76.90	24.8
0.33	4.90	0.37	5.50	4.69	37.8	135.0	80.60	28.8
0.55	8.50	0.07	1.10	4.59	39.5	128.0	86.10	27.9
0.29	5.20	0.15	2.70	4.73	44.6	145.0	94.30	30.7
0.44	6.60	0.05	0.80	3.22	29.1	100.0	90.40	31.1
0.39	5.00	0.33	4.20	5.63	45.8	147.0	81.30	26.1
0.81	6.30	0.19	1.50	5.69	45.6	155.0	80.10	27.2
0.51	4.10	0.12	1.00	4.91	40.5	132.0	82.50	26.9
0.35	4.70	0.10	1.30	4.41	41.0	133.0	93.00	30.2
0.42	7.70	0.11	2.00	5.30	45.5	155.0	85.80	29.2
0.18	4.00	0.18	4.00	4.80	40.4	132.0	84.20	27.5
0.37	3.40	0.07	0.60	4.80	37.9	128.0	79.00	26.7
0.38	6.40	0.03	0.50	5.29	45.0	157.0	85.10	29.7
0.27	3.90	0.20	2.90	4.43	36.2	123.0	81.70	27.8

0.35	6.30	0.42	7.60	4.65	38.7	133.0	83.20	28.6
#NULL!	#NULL!	#NULL!	#NULL!	#NULL!	#NULL!	#NULL!	#NULL!	#NULL!
0.51	6.00	0.15	1.80	4.81	40.3	143.0	83.80	29.7
0.30	4.20	0.01	0.10	2.35	18.3	60.0	77.90	25.5
0.26	4.60	0.07	1.20	4.31	37.7	126.0	87.50	29.2
0.32	7.30	0.07	1.60	5.24	43.4	153.0	82.80	29.2
0.40	5.90	0.06	0.90	4.44	35.7	122.0	80.40	27.5
0.40	5.10	0.11	1.40	5.16	44.3	150.0	85.90	29.1
0.37	6.30	0.24	4.10	4.16	34.3	119.0	82.50	28.6
1.28	36.60	0.21	6.00	1.89	17.5	58.0	92.60	30.7
0.52	8.30	0.12	1.90	5.96	45.0	152.0	88.90	30.0
0.56	9.60	0.19	3.30	5.14	42.5	150.0	82.70	29.2
0.39	6.70	0.09	1.50	4.88	42.8	145.0	87.70	29.7
0.58	9.30	0.65	10.40	4.57	38.5	131.0	84.20	28.7
0.70	9.20	0.22	2.90	5.54	46.0	151.0	83.00	27.3
0.45	8.70	0.12	2.30	4.65	38.7	129.0	83.20	27.7
0.50	7.70	0.09	1.40	5.26	44.2	154.0	84.00	29.3
0.37	6.90	0.16	3.00	5.22	41.2	139.0	78.90	26.6
0.48	8.60	0.08	1.40	4.39	38.5	128.0	87.70	29.2
0.32	6.00	0.05	0.90	4.09	37.8	130.0	92.40	31.8
0.31	7.80	0.09	2.30	4.94	38.8	129.0	78.50	26.1
0.52	5.40	0.32	3.30	5.50	46.2	146.0	84.00	26.5
0.26	7.40	0.04	1.10	5.22	42.6	151.0	81.60	28.9
#NULL!	#NULL!	#NULL!	#NULL!	5.07	#NULL!	152.0	#NULL!	#NULL!
0.58	12.20	8.60	0.41	4.49	38.6	125.0	86.00	27.8
0.38	5.40	0.06	0.80	5.05	42.9	135.0	85.00	26.7
#NULL!	#NULL!	#NULL!	#NULL!	#NULL!	#NULL!	#NULL!	#NULL!	#NULL!
0.16	2.40	0.09	1.30	4.29	37.4	126.0	87.20	29.4
0.73	8.90	0.26	3.20	4.88	42.2	136.0	86.50	27.9
0.39	8.90	0.06	1.40	4.59	38.8	140.0	84.50	30.5
0.47	7.30	0.19	2.90	4.66	38.4	128.0	82.40	27.5
0.27	5.90	0.17	3.70	4.89	41.4	140.0	84.70	28.6
0.48	7.30	0.03	7.30	4.49	36.9	129.0	82.20	28.7
0.41	4.40	0.35	3.80	5.04	43.3	144.0	85.90	28.6
0.23	4.40	0.19	3.70	5.33	42.3	152.0	79.40	28.5
0.64	6.50	0.14	1.40	4.81	39.3	130.0	81.70	27.0
0.83	7.50	0.11	1.00	5.39	43.6	154.0	80.90	28.6
0.68	6.80	0.12	1.20	4.76	41.3	139.0	86.80	29.2
0.30	7.30	0.05	1.20	4.09	34.2	121.0	83.60	29.6
0.35	7.90	0.17	3.80	5.15	39.9	128.0	77.50	24.9
0.33	5.60	0.03	0.50	4.54	39.2	127.0	86.30	28.0
0.58	6.00	0.15	1.60	4.55	39.4	126.0	86.60	27.7
0.41	7.90	0.07	1.40	4.86	41.6	141.0	85.60	29.0
0.47	6.70	0.18	2.60	5.73	50.1	176.0	87.40	30.7
0.56	6.90	0.17	2.10	5.04	38.2	129.0	75.80	25.6
0.75	7.00	0.58	5.50	5.68	45.2	149.0	79.60	26.2
0.30	3.40	0.18	2.00	4.78	40.0	133.0	83.70	27.8
0.51	10.30	0.11	2.20	4.53	40.5	134.0	89.40	29.6
0.37	6.20	0.09	1.50	4.46	37.6	126.0	84.30	28.3
0.27	4.80	0.08	1.40	4.80	38.2	129.0	79.60	26.9
0.62	10.30	0.05	0.80	4.95	42.2	153.0	85.30	30.9
0.60	6.60	0.11	1.20	4.32	36.1	121.0	83.60	28.0
0.49	6.80	0.04	0.60	4.64	41.4	143.0	89.20	30.8
0.49	5.30	0.13	1.40	5.35	44.8	152.0	83.70	28.4

0.36	1.80	0.20	1.00	3.41	27.9	95.0	81.80	27.9
0.41	6.00	0.15	2.20	4.45	37.6	125.0	84.50	28.1
0.52	4.50	0.04	0.30	5.08	43.5	142.0	85.60	28.0
0.40	5.90	0.15	2.20	5.18	44.4	161.0	85.70	31.1
0.43	7.10	0.18	3.00	4.48	39.6	128.0	88.40	28.6
0.35	5.10	0.18	2.60	4.93	39.8	131.0	80.70	26.6
0.95	11.90	0.08	1.00	4.54	37.6	130.0	82.80	28.6
0.35	8.60	0.07	1.70	5.09	45.8	150.0	90.00	29.5
0.36	5.00	0.16	2.20	4.80	41.4	134.0	86.30	27.9
0.39	3.80	0.20	2.00	4.93	39.9	127.0	80.90	25.8
0.36	7.30	0.12	2.40	5.58	45.1	151.0	80.80	27.1
0.41	10.70	0.01	0.30	4.59	38.8	131.0	84.50	28.5
0.32	4.60	0.11	1.60	5.59	43.1	142.0	77.10	25.4
0.47	8.50	0.01	0.20	5.16	43.0	145.0	83.30	28.1
0.37	6.40	0.07	1.20	4.75	40.5	135.0	85.30	28.4
0.12	3.50	0.19	5.60	4.74	39.6	129.0	83.50	27.2
0.43	6.00	0.03	0.40	5.17	45.0	145.0	87.00	28.0
0.36	8.60	0.22	5.30	4.70	38.8	135.0	82.60	28.7
0.17	2.90	0.17	2.90	4.95	40.5	136.0	81.80	27.5
0.04	0.30	0.00	0.00	4.90	38.8	137.0	79.20	28.0
0.26	4.20	0.19	3.10	4.36	35.5	113.0	81.40	25.9
0.52	14.20	0.01	0.10	4.99	40.1	141.0	80.40	28.3
0.40	6.50	0.09	1.50	4.81	40.7	138.0	84.60	28.7
0.22	4.80	0.12	2.60	4.23	35.6	114.0	84.20	27.0
#NULL!	#NULL!	#NULL!	#NULL!	5.31	#NULL!	142.0	#NULL!	#NULL!
0.57	8.20	0.13	1.90	5.38	43.9	144.0	81.60	26.8
0.51	3.90	0.20	1.50	5.13	42.1	134.0	82.10	26.1
0.25	5.20	0.27	5.70	4.94	43.2	145.0	87.40	29.4
0.36	8.80	0.14	3.40	4.30	34.5	116.0	80.20	27.0
0.62	40.20	0.15	2.10	5.23	42.0	142.0	80.30	27.2
0.23	4.60	0.19	3.80	4.92	39.8	134.0	80.90	27.2
0.38	7.00	0.17	3.10	5.47	43.7	139.0	79.90	25.4
0.39	10.80	0.08	2.20	5.21	43.1	143.0	82.70	27.4
0.37	6.40	0.15	2.60	4.57	39.5	130.0	86.40	28.4
0.46	8.10	0.04	0.70	5.11	42.2	142.0	82.60	27.8
0.31	6.20	0.17	3.40	4.76	40.7	132.0	85.50	27.7
0.43	6.50	0.68	10.20	4.57	42.5	142.0	93.00	31.1
0.35	8.40	0.21	5.10	4.53	38.5	130.0	85.00	28.7
0.37	6.00	0.12	1.90	4.82	43.2	141.0	89.60	29.3
0.40	8.10	0.18	3.70	4.58	37.4	124.0	81.70	27.1
0.61	8.00	0.03	0.40	5.55	44.1	145.0	79.50	26.1
0.43	7.10	0.25	4.10	4.10	36.4	111.0	88.80	27.1
0.41	6.20	0.27	4.10	5.40	42.0	147.0	77.80	27.2
0.54	4.60	0.00	0.00	3.93	37.6	123.0	95.70	31.3
0.41	6.20	0.28	4.20	5.03	41.2	143.0	81.90	28.4
0.63	9.30	0.15	2.20	2.71	23.7	76.0	87.50	28.0
0.35	6.20	0.14	2.50	5.16	44.7	152.0	86.60	29.5
0.44	8.20	0.07	1.30	4.61	35.5	121.0	77.00	26.2
0.68	14.10	0.06	1.20	4.04	37.9	120.0	93.80	29.7
1.44	8.00	0.00	0.00	5.57	45.8	147.0	82.20	26.4
0.98	12.20	0.19	2.40	4.70	40.6	134.0	86.40	28.5
0.27	7.60	0.02	0.60	4.73	41.4	132.0	87.50	27.9
0.57	7.40	0.26	3.40	5.09	42.8	141.0	84.10	27.7
0.55	7.50	0.08	1.10	4.70	38.0	131.0	80.90	27.9

0.29	6.80	0.09	2.10	4.96	40.8	138.0	82.30	27.8
0.33	5.80	0.10	1.80	5.07	42.3	151.0	83.40	29.8
0.34	6.70	0.09	1.80	5.80	35.6	110.0	61.40	19.0
0.33	4.10	0.24	3.00	4.57	39.5	130.0	86.40	28.4
0.40	7.50	0.09	1.70	5.19	44.3	144.0	85.40	27.7
0.48	8.00	0.18	3.00	4.89	41.2	141.0	84.30	28.8
0.33	9.30	0.05	1.40	4.60	38.2	131.0	83.00	28.5
0.37	7.50	0.14	2.80	4.29	39.1	128.0	91.10	29.8
0.42	5.00	0.72	8.60	4.64	39.4	128.0	84.90	27.6
0.54	7.20	0.08	1.10	5.17	44.5	140.0	86.10	27.1
#NULL!	#NULL!	#NULL!	#NULL!	#NULL!	#NULL!	124.0	#NULL!	#NULL!
0.40	10.50	0.15	3.90	4.11	38.3	128.0	93.20	31.1
0.46	6.00	0.54	7.00	4.69	42.7	136.0	91.00	29.0
0.23	5.80	0.13	3.30	5.09	46.6	157.0	91.60	30.8
0.43	8.20	0.07	1.30	4.46	39.0	127.0	87.40	28.5
0.43	5.30	0.19	2.30	5.00	41.0	140.0	82.00	28.0
0.31	5.50	0.08	1.40	5.59	46.6	149.0	83.40	26.7
0.44	6.30	0.39	5.50	5.23	46.3	150.0	88.50	28.7
0.66	6.90	0.00	0.00	4.95	39.3	128.0	79.40	25.9
0.20	2.20	0.00	0.00	4.14	40.2	138.0	97.10	33.3
0.40	6.70	0.08	1.20	4.87	41.2	129.0	84.60	26.5
1.10	4.70	0.08	0.30	4.92	42.2	143.0	85.80	29.1
0.45	8.00	0.07	1.20	4.75	42.9	132.0	90.30	27.8
0.39	6.50	0.08	1.30	4.81	40.5	142.0	84.20	29.5
0.36	7.90	0.04	0.90	4.64	41.5	135.0	89.40	29.1
0.32	5.40	0.14	2.40	4.64	41.8	135.0	90.10	29.1
0.44	7.00	0.16	2.60	4.56	43.0	136.0	94.30	29.8
0.27	6.10	0.16	3.60	4.56	38.8	132.0	85.10	28.9
0.22	4.90	0.11	2.40	4.14	37.8	126.0	91.30	30.4
0.30	5.20	0.04	0.70	4.50	38.4	129.0	85.30	28.7
1.13	13.90	0.12	1.50	4.86	41.4	133.0	85.20	27.4
0.99	8.50	0.03	0.30	3.70	34.4	106.0	93.00	28.6
0.55	11.30	0.02	0.40	3.91	42.9	132.0	109.70	33.8
0.47	5.50	0.31	3.60	5.00	41.3	136.0	82.60	27.2
0.50	9.40	0.08	1.50	4.89	40.9	134.0	83.60	27.4
0.60	6.40	0.22	2.40	5.46	45.7	146.0	83.70	26.7
0.63	7.80	0.39	4.90	5.28	46.5	145.0	88.10	27.5
0.66	6.40	0.22	2.10	4.72	41.3	130.0	87.50	27.6
0.23	4.70	0.16	3.30	4.29	38.0	125.0	88.60	29.1
0.59	8.20	0.32	4.40	4.68	35.3	113.0	75.40	24.1
0.53	8.70	0.21	3.40	4.78	41.5	131.0	86.80	27.4
0.35	8.10	0.12	2.80	4.43	39.1	126.0	88.30	28.4
#NULL!	#NULL!	#NULL!	#NULL!	#NULL!	#NULL!	#NULL!	#NULL!	#NULL!
0.42	5.60	0.65	8.70	4.43	40.2	132.0	90.70	29.8
#NULL!	#NULL!	#NULL!	#NULL!	#NULL!	#NULL!	#NULL!	#NULL!	#NULL!
0.27	3.90	0.47	6.80	4.28	40.4	134.0	94.40	31.3
0.42	7.70	0.08	1.50	4.38	36.8	127.0	84.00	29.0
0.58	8.30	0.65	9.30	4.20	38.0	129.0	90.50	30.7
0.45	8.90	0.25	5.00	4.31	37.7	128.0	87.50	29.7
0.44	10.20	0.07	1.60	5.06	42.2	141.0	83.40	27.9
0.31	4.90	0.13	2.10	4.44	37.4	121.0	84.20	27.3
1.24	6.50	0.00	0.00	5.02	43.3	142.0	86.30	28.3
0.19	5.90	0.05	2.60	3.89	42.3	149.0	86.50	30.5
0.28	4.60	0.11	1.80	4.87	42.9	141.0	88.10	29.0

0.42	5.90	0.10	1.40	3.93	36.1	118.0	91.90	30.0
0.34	6.30	0.16	3.00	5.00	40.5	134.0	81.00	26.8
0.56	6.30	0.19	2.10	4.53	38.4	129.0	84.80	28.5
0.47	10.10	0.07	1.50	4.20	35.5	120.0	84.50	28.6
0.40	5.90	0.08	1.20	5.10	44.8	149.0	87.80	29.2
0.56	7.50	0.40	5.40	4.79	42.0	142.0	87.70	29.6
0.53	5.90	0.16	1.80	5.03	41.8	136.0	83.10	27.0
0.22	3.50	0.11	1.70	4.66	40.5	131.0	86.90	28.1
0.29	5.20	0.14	2.50	4.28	40.0	129.0	93.50	30.1
#NULL!	8.30	#NULL!	#NULL!	5.31	#NULL!	133.0	#NULL!	#NULL!
0.47	8.50	0.10	1.80	4.85	39.0	128.0	80.40	26.4
0.33	4.60	0.11	1.50	5.09	40.8	138.0	80.20	27.1
0.60	8.70	0.12	1.70	4.74	38.8	127.0	81.90	26.8
0.55	8.80	0.19	3.00	4.98	42.8	143.0	85.90	28.7
0.22	5.40	0.05	1.20	4.84	42.4	142.0	97.60	29.3
0.28	6.00	0.10	2.10	4.21	36.6	130.0	86.90	30.9
#NULL!	#NULL!	#NULL!	#NULL!	4.89	#NULL!	132.0	#NULL!	#NULL!
0.42	6.80	0.17	2.80	4.65	42.1	138.0	90.50	29.7
0.39	4.10	5.12	53.60	4.85	39.8	133.0	82.10	27.4
0.65	10.20	0.33	5.20	4.36	37.2	126.0	85.30	28.9
0.58	8.50	0.08	1.20	5.37	46.3	160.0	86.20	29.8
0.41	8.30	0.07	1.40	5.20	43.5	140.0	83.70	26.9
0.52	8.60	0.12	2.00	4.78	39.9	129.0	83.50	27.0
0.72	5.00	0.08	0.60	5.32	40.1	133.0	75.40	25.0
0.32	6.20	0.14	2.70	4.80	38.5	128.0	80.20	26.7
0.55	8.40	0.06	0.90	2.89	25.4	82.0	87.90	28.4
0.29	7.00	0.09	2.20	4.89	41.6	139.0	85.10	28.4
0.32	5.10	0.14	2.20	4.32	34.8	112.0	80.60	25.9
0.29	7.40	0.38	9.60	3.98	33.2	109.0	83.40	27.4
0.42	7.80	0.12	2.20	5.24	39.0	123.0	74.40	23.5
0.47	5.70	0.06	0.70	5.09	43.5	142.0	85.50	27.9
0.29	6.10	0.16	3.40	4.12	35.5	120.0	86.20	29.1
0.36	7.60	0.05	1.10	5.02	34.1	106.0	67.90	21.1
0.55	9.10	0.28	4.60	4.68	40.1	135.0	85.70	28.8
0.98	7.50	0.18	1.40	4.54	39.0	130.0	85.90	28.6
0.26	6.00	0.22	5.10	4.34	38.8	132.0	89.40	30.4
0.33	7.10	0.13	2.80	4.58	40.9	130.0	89.30	28.4
2.05	7.10	0.02	0.10	4.99	39.8	138.0	79.80	27.7
0.35	7.10	0.11	2.20	5.02	44.0	143.0	87.60	28.5
0.40	7.60	0.13	2.50	5.08	39.3	123.0	77.40	24.2
0.40	5.10	0.50	0.04	4.79	42.0	142.0	87.70	29.6
0.49	5.20	0.11	1.20	4.17	41.3	138.0	91.10	30.5
0.34	6.20	0.13	2.40	5.02	37.4	125.0	74.50	24.9
0.63	10.00	0.69	11.00	4.76	40.5	137.0	85.10	28.8
0.35	8.00	0.14	3.20	4.85	41.1	141.0	84.70	29.1
0.93	10.90	0.67	7.80	5.11	42.9	142.0	84.00	27.8
0.38	8.10	0.12	2.60	4.50	38.9	129.0	86.40	28.7
0.44	8.20	0.18	3.40	4.72	41.8	138.0	88.60	29.2
0.34	4.50	0.20	2.70	5.62	46.1	152.0	82.10	27.0
0.37	6.60	0.57	10.10	5.17	42.8	147.0	82.80	28.3
0.46	8.80	0.08	1.50	4.83	42.6	143.0	88.30	29.7
0.68	11.50	0.31	5.30	5.56	47.6	159.0	85.50	28.5
0.25	3.90	0.07	1.10	4.93	40.4	134.0	82.00	27.3
0.38	8.80	0.13	2.90	4.67	40.6	141.0	86.90	30.1

0.23	4.50	0.16	3.10	5.04	41.9	140.0	83.20	27.7
0.30	6.10	0.11	2.20	5.39	46.3	157.0	85.90	29.1
0.38	11.30	0.08	2.40	4.76	40.1	134.0	84.40	28.3
0.42	9.40	0.13	2.80	4.72	39.4	127.0	83.40	26.8
0.51	7.00	0.13	1.80	5.50	43.2	144.0	78.60	26.3
0.32	5.50	0.17	2.90	5.26	44.6	150.0	84.90	28.5
0.22	6.20	0.15	4.10	4.62	40.1	136.0	86.90	29.4
0.16	5.40	0.13	4.50	5.45	47.9	162.0	87.80	29.7
0.22	3.20	0.12	3.20	4.66	40.4	142.0	86.80	30.5
0.70	8.50	0.09	1.10	5.14	45.2	152.0	87.80	29.5
0.42	7.80	0.14	2.60	4.96	41.0	133.0	82.70	26.8
0.21	3.90	0.06	1.10	4.76	40.1	132.0	84.20	27.8
0.18	4.70	0.02	0.50	5.00	43.1	147.0	86.20	29.4
0.74	8.70	0.00	0.00	4.78	40.8	135.0	85.30	28.3
0.42	9.30	0.00	0.10	5.31	42.5	140.0	80.10	26.3
0.28	5.80	0.07	1.40	4.55	39.6	130.0	86.90	28.6
0.27	6.60	0.05	1.20	4.83	43.4	147.0	89.90	30.4
0.26	4.40	0.25	4.20	4.30	37.6	124.0	87.60	28.8
0.45	3.50	0.03	0.20	4.37	37.4	124.0	85.40	28.5
0.82	9.30	0.19	2.20	5.43	42.2	139.0	77.80	25.6
0.38	5.40	0.08	1.20	4.36	37.5	125.0	86.10	28.7
0.38	7.60	0.01	0.20	4.57	40.0	134.0	87.40	29.3
0.38	5.40	0.37	5.30	5.04	40.4	135.0	80.10	26.9
0.47	5.90	0.48	6.10	5.41	47.6	161.0	88.00	29.8
0.84	6.70	0.04	0.30	2.23	19.7	66.0	88.20	29.4
0.32	5.60	0.09	1.50	3.78	32.4	108.0	85.70	28.7
0.21	5.00	0.14	3.30	5.02	42.9	144.0	85.50	28.7
0.45	9.50	0.24	5.20	4.47	36.8	121.0	82.40	27.2
0.25	6.60	0.12	3.00	4.63	39.3	127.0	85.00	27.6
0.33	4.60	0.09	1.20	4.40	37.3	126.0	84.80	28.5
0.41	5.40	0.07	0.90	4.89	40.6	133.0	83.10	27.3
0.67	2.30	0.02	0.10	4.58	37.6	128.0	82.10	28.0
0.18	2.20	0.02	0.20	4.66	40.7	140.0	87.20	20.1
0.54	4.70	0.07	0.60	5.19	45.3	148.0	87.30	28.4
0.34	5.50	0.01	0.20	5.33	40.4	144.0	75.80	27.0
0.48	8.80	0.24	4.30	5.13	42.4	144.0	82.60	28.1
0.41	5.40	0.09	1.20	4.62	42.5	145.0	92.00	31.3
0.54	5.20	0.01	0.10	4.03	34.3	124.0	85.20	30.7
0.20	3.30	0.06	0.90	5.08	43.6	148.0	85.80	29.2
0.38	6.70	0.17	3.00	4.60	41.0	138.0	89.00	30.0
0.22	4.30	0.34	6.80	4.70	41.8	148.0	88.80	31.4
0.20	4.40	0.15	3.20	4.95	45.9	155.0	92.70	31.3
0.24	6.50	0.13	3.40	4.31	37.4	128.0	86.90	29.8
0.19	4.70	0.27	6.50	4.52	39.8	132.0	88.10	29.3
0.21	4.70	0.08	1.80	4.83	44.0	144.0	90.70	29.8
0.38	6.70	0.02	0.30	4.64	39.9	141.0	86.10	30.3
0.29	5.60	0.09	1.60	4.38	39.0	130.0	89.20	29.7
0.25	5.10	0.15	3.10	4.42	39.1	128.0	88.50	29.0
0.35	6.20	0.52	9.10	4.72	38.8	132.0	82.20	28.0
0.26	5.30	0.07	1.40	4.48	39.8	135.0	88.80	30.2
0.35	6.00	0.16	2.80	4.60	39.6	134.0	86.00	29.2
0.25	5.30	0.24	5.30	4.73	40.5	138.0	85.60	29.2
2.13	7.60	0.18	3.40	4.82	39.2	131.0	81.20	27.2
0.63	3.00	0.00	0.00	3.78	32.6	110.0	86.20	29.1

0.55	6.30	0.25	2.90	5.29	39.1	131.0	74.00	24.7
0.21	5.30	0.10	2.60	4.48	38.3	129.0	85.50	28.8
0.93	14.20	0.00	0.00	4.18	39.6	133.0	94.70	31.8
0.42	5.30	0.27	3.30	4.68	39.2	134.0	83.70	28.7
0.32	6.10	0.25	4.80	4.14	36.1	123.0	87.10	29.8
0.24	4.10	0.05	0.80	4.45	38.8	131.0	87.10	29.4
0.50	4.50	0.00	0.00	5.44	45.5	152.0	83.50	27.9
0.40	7.00	0.11	2.00	4.67	41.2	138.0	88.10	28.4
0.38	27.90	0.07	1.00	5.69	49.0	167.0	86.10	29.3
0.27	5.90	0.13	2.80	4.54	38.1	130.0	83.80	28.5
0.27	6.70	0.07	1.70	4.51	39.9	136.0	88.50	30.1
0.43	7.60	0.14	2.40	4.67	38.7	128.0	82.70	27.4
0.62	5.90	0.24	2.20	4.73	43.0	146.0	90.90	30.9
0.47	8.60	0.11	2.00	5.13	41.8	145.0	81.50	28.3
0.30	5.20	0.12	2.10	4.95	42.1	148.0	85.10	29.8
#NULL!	#NULL!	#NULL!	#NULL!	4.61	39.1	134.0	#NULL!	#NULL!
0.32	6.10	0.29	5.50	4.07	36.3	125.0	89.20	30.7
0.27	8.10	0.11	3.20	4.49	39.2	133.0	87.20	29.6
0.18	4.80	0.21	5.50	4.36	38.8	133.0	89.10	30.5
0.43	5.40	0.10	1.20	5.28	45.8	155.0	86.80	29.3
0.43	5.90	0.16	2.10	4.34	39.0	131.0	90.00	30.3
0.36	6.70	0.17	3.10	4.82	37.8	128.0	78.40	26.5
0.33	4.20	0.53	6.60	4.21	36.1	121.0	85.60	28.6
0.21	5.30	0.13	3.40	4.60	39.5	135.0	85.90	29.3
0.23	4.80	0.06	1.20	4.68	39.3	133.0	83.90	28.5
0.42	5.70	0.33	4.50	5.00	39.6	134.0	79.10	26.8
1.59	6.40	0.03	0.10	3.64	33.7	111.0	92.70	30.4
0.42	6.00	0.05	0.70	4.80	44.2	150.0	92.10	31.3
0.64	8.60	0.16	2.10	4.13	36.7	126.0	89.00	30.4
0.55	6.80	0.14	1.70	4.40	38.2	131.0	86.80	29.8
0.44	7.10	0.25	4.10	4.40	38.7	132.0	87.90	30.0
0.34	8.40	0.05	1.10	4.72	39.5	136.0	83.60	28.9
0.68	5.20	0.00	0.00	4.04	33.7	114.0	83.40	28.3
0.22	6.10	0.07	2.00	4.96	40.4	138.0	81.50	27.8
0.24	4.50	0.15	2.70	5.27	44.1	143.0	83.60	27.2
0.31	5.00	0.11	1.80	4.40	39.7	137.0	90.30	31.2
0.50	7.70	0.04	0.50	4.61	38.2	130.0	82.90	28.1
0.32	5.60	0.04	0.80	4.44	42.1	142.0	94.90	31.9
0.19	3.90	0.20	4.20	4.45	38.4	129.0	86.30	28.9
1.09	4.80	0.00	0.00	5.44	44.3	146.0	81.40	26.8
0.35	6.30	0.05	0.90	3.86	36.5	125.0	94.50	32.4
0.41	9.40	0.15	3.50	4.66	39.9	137.0	85.60	29.4
0.23	4.00	0.06	1.00	4.28	37.9	131.0	88.60	30.5
0.43	4.70	0.08	0.90	4.25	38.5	131.0	90.50	30.8
0.48	4.90	0.08	0.80	3.33	28.8	95.0	86.70	28.6
0.52	11.80	0.07	1.50	4.37	39.5	134.0	90.30	30.6
0.32	4.70	0.26	3.90	4.62	43.4	149.0	93.90	32.3
0.40	6.80	0.30	5.10	4.33	39.0	130.0	90.10	30.1
0.44	30.40	0.05	0.70	5.59	48.6	164.0	87.00	29.3
0.54	9.10	0.29	4.80	5.04	43.5	152.0	86.40	30.2
0.29	5.90	0.14	2.80	5.35	47.6	161.0	88.90	30.2
0.30	4.30	0.12	1.70	5.07	43.2	150.0	85.10	29.5
0.55	14.90	0.16	4.40	4.81	39.6	135.0	82.40	28.1
0.73	12.20	0.01	0.20	1.03	8.6	25.0	83.50	24.3

0.42	7.10	0.06	1.00	4.58	38.4	131.0	84.00	28.6
0.83	5.90	0.18	1.30	5.11	42.8	150.0	83.70	29.4
0.43	7.50	0.21	3.70	4.78	41.3	142.0	86.40	29.8
0.29	5.40	0.23	4.30	4.46	38.0	129.0	85.20	29.0
0.31	5.60	0.04	0.70	4.70	41.0	140.0	87.40	29.8
0.23	8.10	0.04	1.50	4.84	39.3	132.0	81.10	27.3
0.61	11.10	0.18	3.30	4.23	34.8	114.0	82.30	27.0
0.25	4.40	0.09	1.70	4.25	39.7	136.0	93.40	31.9
0.20	4.10	0.08	1.60	4.39	38.0	131.0	86.60	29.9
0.35	3.80	0.03	0.30	4.80	42.3	145.0	88.20	30.3
0.45	4.90	0.45	4.80	4.88	42.3	143.0	86.70	29.4
0.52	8.60	0.16	2.70	4.25	36.0	121.0	84.80	28.5
0.21	5.80	0.05	1.30	4.60	39.7	134.0	86.30	29.0
0.45	7.10	0.42	6.60	4.64	35.8	115.0	77.00	24.9
0.47	4.90	0.05	0.50	4.82	41.9	143.0	86.90	29.7
0.39	6.70	0.37	6.30	4.98	39.0	128.0	78.40	25.6
0.30	8.00	0.11	2.90	4.75	40.9	137.0	86.10	28.8
0.48	5.80	0.18	2.20	4.86	40.2	137.0	82.60	28.1
0.26	4.70	0.14	2.60	4.73	38.7	133.0	81.90	28.2
0.43	4.60	0.14	1.50	4.76	39.8	129.0	83.50	27.2
0.20	4.70	0.08	1.90	4.45	40.0	136.0	90.00	30.5
0.56	6.60	0.09	1.10	4.74	41.0	139.0	87.10	29.3
0.36	4.20	0.34	4.00	4.75	40.1	137.0	84.40	28.9
0.52	8.00	0.39	6.00	4.54	40.4	137.0	89.00	30.2
0.44	6.50	0.13	2.00	4.98	41.7	140.0	83.80	28.2
0.31	5.40	0.07	1.20	4.54	39.4	132.0	86.70	29.0
0.55	6.90	0.39	4.80	4.89	41.5	140.0	84.90	28.5
0.37	5.20	0.38	5.30	4.73	38.0	126.0	80.30	26.6
0.60	6.20	0.03	0.30	5.81	50.5	170.0	87.00	19.3
0.36	5.80	0.32	5.10	4.24	36.1	119.0	85.10	28.0
0.30	3.80	0.13	1.60	4.72	40.0	133.0	84.70	28.2
0.37	4.50	0.11	1.30	5.14	43.8	148.0	85.40	28.7
0.25	4.50	0.42	7.50	5.02	42.3	139.0	84.30	27.7
0.12	1.10	0.01	0.10	5.18	43.6	149.0	84.20	28.7
0.39	7.40	0.13	2.50	5.04	40.3	131.0	79.90	25.9
0.28	6.80	0.04	1.00	4.19	38.0	124.0	89.70	29.6
0.29	4.80	0.08	1.40	5.43	44.5	149.0	82.00	27.5
0.32	5.10	0.41	6.60	5.54	47.1	160.0	85.10	28.9
0.29	4.50	0.05	0.70	4.87	41.3	141.0	84.70	28.8
0.46	6.40	0.35	4.90	5.21	45.1	152.0	86.50	29.1
0.36	3.70	0.57	5.90	4.93	40.4	137.0	81.90	27.7
1.15	6.30	0.90	4.90	5.31	42.4	145.0	79.80	27.4
#NULL!	#NULL!	#NULL!	#NULL!	#NULL!	#NULL!	#NULL!	#NULL!	#NULL!
0.18	3.90	0.14	3.10	4.96	42.4	143.0	85.40	28.7
0.44	7.90	0.24	4.40	4.77	40.1	135.0	84.10	28.3
0.39	4.40	0.13	1.50	4.70	42.3	141.0	89.80	29.9
0.37	6.10	0.18	3.00	5.54	44.0	149.0	79.50	26.8
0.43	7.10	0.09	1.50	5.05	40.9	137.0	81.00	27.1
0.34	5.40	0.22	3.40	5.12	39.5	125.0	77.10	24.5
0.54	7.30	0.14	1.90	5.48	44.1	150.0	80.40	27.3
0.25	5.70	0.17	3.70	4.86	36.9	119.0	76.10	24.5
0.14	3.80	0.17	4.70	4.51	41.5	138.0	92.00	30.6
0.41	7.10	0.10	1.70	4.74	38.8	128.0	81.70	27.0
0.25	5.40	0.20	4.40	4.85	40.6	134.0	83.80	27.7

0.40	11.00	0.01	2.30	5.14	44.7	153.0	86.90	29.7
0.55	4.00	0.20	1.50	4.91	40.3	140.0	82.10	28.6
0.39	7.40	0.14	2.70	5.00	44.0	153.0	88.40	30.6
0.68	6.80	0.09	0.90	5.15	40.6	132.0	78.80	25.7
0.36	4.60	0.18	2.30	5.04	43.3	151.0	85.90	30.0
0.39	5.60	0.19	2.70	4.68	37.4	123.0	79.90	26.3
0.31	7.00	0.16	3.70	3.97	34.8	114.0	87.70	28.8
0.45	4.70	0.20	2.10	5.38	44.1	148.0	81.90	27.5
0.19	4.90	0.07	1.70	4.88	40.6	138.0	83.10	28.4
0.30	6.50	0.28	6.00	4.43	37.9	128.0	85.50	29.0
0.38	5.00	0.26	5.00	4.49	40.3	134.0	89.80	29.8
0.36	6.00	0.12	2.10	4.72	39.9	135.0	84.60	28.6
0.34	6.10	0.06	1.10	4.38	36.5	122.0	83.40	27.9
0.31	7.60	0.23	5.70	4.07	32.9	105.0	80.90	25.9
0.25	4.60	0.22	4.10	4.82	41.6	146.0	86.40	30.3
0.33	4.50	0.08	1.10	4.74	41.5	139.0	87.50	29.2
0.21	4.30	0.08	1.70	4.38	39.3	130.0	89.80	29.7
#NULL!	#NULL!	#NULL!	#NULL!	#NULL!	#NULL!	#NULL!	#NULL!	#NULL!
0.27	3.50	0.10	1.30	4.79	42.9	145.0	89.60	30.3
0.32	3.70	0.07	0.80	5.65	47.5	163.0	84.00	28.8
0.48	7.20	0.43	6.40	4.61	41.6	141.0	90.20	30.6
0.30	7.00	0.07	1.60	5.21	44.1	153.0	84.60	29.4
0.44	4.90	0.26	2.90	5.16	43.9	150.0	85.10	29.1
0.23	4.10	0.04	0.80	4.81	42.9	147.0	89.10	30.5
0.17	3.10	0.07	1.20	4.49	39.1	130.0	87.10	29.1
0.21	4.30	0.25	5.00	4.41	35.3	117.0	80.00	26.4
0.24	5.30	0.24	5.50	4.91	44.2	150.0	90.00	30.6
0.42	7.20	0.64	10.90	5.32	42.9	145.0	80.60	27.2
0.39	7.20	0.16	3.00	4.68	38.2	128.0	81.60	27.4
0.28	5.20	0.28	5.10	4.67	38.9	127.0	83.40	27.3
0.53	5.50	0.18	1.90	5.45	44.5	146.0	81.70	26.8
0.43	6.30	0.43	6.40	4.71	40.6	134.0	86.10	28.6
0.38	4.00	0.13	1.40	4.69	43.0	146.0	91.60	31.2
0.66	9.00	0.23	3.10	4.92	41.9	141.0	85.20	28.7
0.53	7.90	0.18	2.70	5.00	40.3	133.0	80.60	26.6
0.54	5.10	0.28	2.70	4.93	43.0	145.0	87.60	29.4
0.23	5.80	0.06	1.60	4.53	39.7	132.0	87.70	29.1
0.28	4.70	0.19	3.30	4.66	37.3	127.0	80.10	27.3
0.15	3.90	0.08	2.10	4.22	38.2	128.0	90.50	30.2
0.29	4.00	0.15	2.00	5.16	42.0	142.0	81.40	27.5
0.27	6.90	0.07	1.90	4.67	40.3	133.0	86.40	28.5
0.31	5.30	0.11	1.90	4.99	40.5	131.0	81.20	26.2
1.57	9.50	0.02	0.10	4.27	36.9	125.0	86.30	29.2
0.20	7.30	0.03	1.10	2.75	20.4	61.0	74.10	22.0
0.19	4.10	0.07	1.60	4.92	40.1	132.0	81.50	26.9
0.28	4.70	0.18	2.90	5.62	48.1	164.0	85.50	29.2
0.55	7.20	0.13	1.70	4.13	36.2	126.0	87.70	30.5
0.39	4.50	0.31	3.60	5.17	43.3	148.0	83.70	28.6
0.56	6.60	0.13	1.50	5.46	46.1	153.0	84.40	28.0
0.28	6.60	0.06	1.40	5.06	42.5	144.0	84.00	28.5
0.42	5.70	0.12	1.70	4.98	39.8	134.0	79.90	27.0
0.21	4.00	0.13	2.50	4.74	40.0	132.0	84.40	27.9
0.43	6.30	0.13	1.90	4.54	38.1	128.0	83.90	28.3
0.23	5.60	0.09	2.10	4.60	40.1	132.0	87.10	28.7

0.34	4.50	0.32	4.30	4.35	35.3	117.0	81.20	27.0
0.29	4.70	0.15	2.40	5.42	40.0	137.0	73.80	25.3
0.40	6.90	0.12	2.10	4.65	42.5	144.0	91.50	31.0
0.61	6.90	0.25	2.80	4.87	42.6	142.0	87.50	29.1
0.20	4.70	0.13	3.10	4.73	40.0	134.0	84.60	28.3
0.52	5.50	0.45	4.70	5.05	41.7	141.0	82.60	27.8
0.33	6.10	0.61	11.40	4.85	42.9	150.0	88.50	30.8
0.63	7.80	0.38	4.70	4.98	41.4	140.0	83.10	28.0
0.47	5.00	0.39	4.20	4.83	38.5	133.0	79.70	27.5
0.43	6.20	0.31	4.40	4.58	37.7	128.0	82.30	28.0
0.35	6.50	0.44	8.30	4.43	37.4	126.0	84.50	28.6
0.22	6.00	0.12	3.40	4.91	39.9	135.0	81.30	27.5
0.43	7.30	0.27	4.60	4.80	41.9	146.0	87.20	30.3
0.21	8.50	0.09	3.50	4.50	38.6	128.0	85.70	28.5
0.37	7.40	0.17	3.50	4.88	41.8	143.0	85.60	29.3
0.31	5.50	0.38	6.70	4.49	39.9	133.0	88.80	29.7
0.74	8.30	0.31	3.50	4.56	37.6	122.0	82.50	26.7
0.25	4.90	0.15	2.90	5.09	38.7	127.0	76.10	25.0
0.36	6.40	0.31	5.50	5.06	41.8	139.0	82.60	27.5
0.62	8.30	0.31	4.20	4.23	35.3	120.0	83.40	28.4
0.44	6.30	0.53	7.50	5.01	41.9	139.0	83.60	27.8
0.41	8.80	0.42	8.90	5.04	41.1	139.0	81.50	27.5

MCHC	PLT	TP	ALB	GLB	ALT	AST	γGGT	TB
327.0	347	66.70	42.50	24.20	6.90	17.90	10.50	7.3
342.0	213	67.20	44.50	22.70	8.50	18.40	10.90	10.9
338.0	237	69.90	44.90	25.00	8.40	12.80	17.40	13.1
318.0	255	66.20	40.50	25.70	28.20	20.50	21.80	7.2
327.0	237	69.40	42.90	26.50	53.00	22.60	80.70	40.4
332.0	288	62.70	37.80	24.90	13.00	31.00	#NULL!	10.1
343.0	286	66.40	42.30	24.10	14.00	15.90	13.60	4.1
316.0	312	64.70	41.70	23.00	7.50	16.10	15.60	21.2
327.0	384	79.70	46.70	33.00	14.50	23.00	13.10	13.7
332.0	326	63.90	43.20	20.70	12.50	25.90	7.90	5.4
336.0	297	59.00	42.60	16.40	14.60	17.60	11.50	8.6
325.0	255	64.40	37.70	26.70	29.90	23.60	15.10	2.4
337.0	269	66.60	39.80	26.80	14.60	17.40	11.20	4.2
333.0	286	67.20	43.70	23.50	5.40	17.70	14.90	3.1
321.0	409	72.10	40.70	31.40	4.80	9.10	15.20	9.0
345.0	152	66.50	40.60	25.90	10.80	16.20	11.20	12.4
326.0	485	78.10	32.70	45.40	5.30	11.50	16.10	3.5
322.0	273	62.50	38.70	23.80	14.20	10.20	14.00	12.0
305.0	340	73.70	48.50	25.20	36.20	15.70	32.40	11.4
335.0	439	66.40	39.20	27.20	14.80	18.00	17.00	3.4
327.0	226	70.60	43.30	27.30	5.50	10.40	9.30	23.2
344.0	322	75.20	42.50	32.70	16.00	12.30	39.50	18.5
327.0	327	69.60	41.60	28.00	6.60	15.40	14.60	4.8
321.0	344	71.50	44.90	26.60	20.10	36.80	9.90	15.5
336.0	424	73.40	37.80	35.60	7.20	20.10	7.10	6.3
325.0	197	62.00	36.00	26.00	27.00	34.00	#NULL!	4.0
358.0	254	64.50	39.20	25.30	11.30	17.10	16.00	4.5
335.0	351	69.20	42.30	26.90	21.10	17.30	15.10	13.7
333.0	240	63.00	39.70	23.30	5.00	22.70	7.90	10.0
329.0	360	68.40	44.80	23.60	14.00	22.70	17.80	19.7
360.0	230	67.90	42.20	25.70	14.70	20.50	12.00	4.5
344.0	332	67.40	44.90	22.50	12.00	14.10	14.80	7.5
344.0	303	67.00	39.70	27.30	34.50	24.60	22.10	4.1
329.0	190	64.00	37.00	27.00	10.00	9.00	#NULL!	5.0
316.0	371	55.70	34.40	21.30	19.40	6.50	11.40	11.7
331.0	296	63.00	36.00	27.00	8.00	7.00	#NULL!	4.0
327.0	467	62.40	39.90	22.50	73.80	30.60	20.80	10.3
323.0	70	61.80	34.10	27.70	38.90	54.40	67.50	14.0
323.0	321	73.30	46.10	27.20	17.60	28.60	11.60	8.9
352.0	484	69.90	41.00	28.90	8.20	14.50	14.70	8.5
290.0	352	57.50	35.30	22.20	9.70	23.60	6.50	4.2
327.0	315	76.70	45.10	31.60	6.30	16.00	15.40	8.6
340.0	375	68.80	40.60	28.20	11.80	15.10	11.90	11.2
335.0	265	67.50	39.60	27.90	7.80	14.40	10.80	3.6
329.0	230	63.00	40.00	23.00	4.00	5.00	#NULL!	12.0
325.0	293	79.00	42.10	36.90	9.70	18.60	15.60	5.9
328.0	177	66.20	39.30	26.90	8.70	18.10	9.90	9.3
330.0	310	67.20	37.70	29.50	6.10	13.10	12.40	7.3
330.0	228	66.20	40.80	25.40	11.60	28.60	11.40	9.8
338.0	393	86.50	50.00	36.50	13.80	24.30	11.90	10.4
323.0	316	66.00	39.30	26.70	10.40	21.10	8.20	11.9
329.0	258	75.30	44.00	31.30	29.50	22.00	27.00	10.0
343.0	186	65.40	41.90	23.50	22.00	21.30	15.80	16.1

318.0	280	63.20	35.50	27.70	4.50	14.00	18.00	4.2
318.0	65	61.30	32.40	28.90	413.00	1078.00	65.60	10.7
318.0	334	58.80	37.40	21.40	5.60	11.90	13.80	5.0
315.0	477	28.98	14.19	14.79	17.00	26.00	#NULL!	5.0
328.0	265	63.90	41.80	22.10	17.90	28.40	12.60	8.9
351.0	228	69.90	43.50	26.40	12.30	16.20	16.20	10.4
329.0	100	73.70	43.80	29.90	8.60	13.60	11.80	9.3
330.0	302	72.80	46.40	26.40	12.70	21.90	18.20	14.6
328.0	418	69.00	41.10	27.90	28.30	38.50	14.30	2.9
335.0	297	70.30	43.50	26.80	13.00	19.30	21.40	7.8
316.0	351	67.70	44.70	23.00	12.20	25.70	8.80	24.7
317.0	403	70.40	40.70	29.70	11.90	19.20	10.90	5.6
328.0	176	62.00	40.40	21.60	10.60	14.60	15.00	11.9
329.0	295	67.10	42.80	24.30	12.20	20.20	12.70	4.0
334.0	359	61.40	41.60	19.80	38.00	33.00	8.10	7.2
317.0	270	61.00	34.90	26.10	7.90	11.80	21.40	9.2
229.0	281	66.00	35.00	31.00	10.00	23.00	#NULL!	3.6
314.0	308	68.70	40.70	28.00	8.50	17.00	13.30	16.6
322.0	205	67.70	36.10	31.60	8.50	11.00	8.90	6.4
337.0	315	70.80	42.50	28.30	59.00	40.00	19.90	7.2
331.0	239	69.60	41.90	27.70	99.00	37.60	275.00	18.8
332.0	432	61.30	37.60	23.70	11.10	33.90	15.20	7.5
333.0	300	70.80	43.90	26.90	13.20	41.40	11.10	7.4
329.0	421	70.90	41.00	29.90	13.60	14.70	23.80	4.6
345.0	279	71.10	42.10	29.00	5.60	12.40	16.00	20.5
347.0	209	72.00	47.20	24.80	14.10	35.00	9.90	11.5
352.0	334	65.40	40.90	24.50	14.70	22.40	13.80	11.0
327.0	241	69.80	40.60	29.20	7.10	13.70	17.70	3.2
320.0	267	62.60	39.60	23.00	7.40	23.60	11.90	2.2
333.0	218	61.40	40.00	21.40	26.00	20.00	17.10	8.1
345.0	95	62.00	36.00	26.00	15.00	20.00	#NULL!	10.0
335.0	406	66.30	39.80	26.50	13.50	22.10	41.40	4.9
334.0	453	68.00	40.50	27.50	9.70	12.30	8.00	11.2
347.0	308	51.40	33.80	17.60	9.50	17.60	10.10	13.0
330.0	189	64.70	43.40	21.30	12.10	12.10	14.50	8.4
349.0	223	67.50	41.10	26.40	26.40	36.30	10.20	10.1
323.0	353	63.50	34.30	29.20	15.80	18.30	16.30	5.6
333.0	270	64.20	36.70	27.50	10.90	22.90	9.10	7.4
319.0	349	67.80	40.00	27.80	12.20	21.40	11.60	8.9
363.0	211	76.40	40.90	35.50	15.20	28.30	11.60	6.9
325.0	298	66.80	40.20	26.60	13.80	15.60	14.80	4.2
309.0	197	78.10	43.30	34.80	12.50	22.60	12.30	10.0
332.0	198	68.30	39.40	28.90	21.30	25.20	18.50	6.5
337.0	200	70.00	43.00	27.00	13.10	18.40	10.90	11.9
318.0	350	75.50	45.60	29.90	13.00	17.00	17.90	4.6
330.0	263	72.30	49.20	23.10	9.80	17.50	17.10	13.4
337.0	283	76.30	46.90	29.40	11.70	17.80	21.10	13.2
353.0	267	65.50	38.10	27.40	14.00	25.00	#NULL!	6.0
347.0	318	66.40	40.70	25.70	15.30	20.90	12.50	20.6
344.0	230	70.50	44.80	25.70	18.00	27.40	12.10	14.6
327.0	306	69.30	42.40	26.90	22.40	21.50	30.40	4.0
327.0	290	71.30	43.60	27.70	8.90	21.40	12.60	9.2
344.0	221	65.20	39.50	25.70	5.80	34.80	13.10	7.4
332.0	260	66.00	34.00	32.00	19.00	16.00	#NULL!	3.2

339.0	185	68.80	43.50	25.30	9.50	25.20	15.20	9.1
331.0	215	65.00	35.00	30.00	9.00	9.00	#NULL!	7.0
330.0	301	64.00	34.00	30.00	7.00	5.00	#NULL!	8.0
326.0	298	67.30	42.00	25.30	16.90	16.50	15.80	13.1
319.0	400	70.70	48.90	21.80	18.80	23.10	13.20	10.2
296.0	168	59.50	24.80	34.70	24.80	50.30	50.50	11.5
317.0	232	69.20	43.90	25.30	24.70	25.10	11.50	4.1
330.0	258	72.30	43.80	28.50	33.20	32.80	17.80	13.6
282.0	334	56.40	35.10	21.30	16.20	17.60	9.60	8.0
324.0	270	65.80	42.20	23.60	16.50	25.70	9.10	4.7
325.0	207	58.80	37.60	21.20	25.90	33.60	12.10	7.9
317.0	228	62.80	33.80	29.00	13.30	25.70	9.00	31.9
316.0	338	69.70	45.90	23.80	13.70	19.50	11.10	24.3
318.0	341	64.70	42.20	22.50	9.10	15.50	12.50	6.2
322.0	313	72.20	45.50	26.70	9.00	19.50	13.60	8.2
352.0	268	74.10	43.20	30.90	11.50	19.30	10.30	8.4
315.6	420	70.20	41.80	28.40	25.20	23.00	27.40	5.6
319.0	241	69.10	39.20	29.90	16.00	23.70	14.60	7.1
327.0	242	71.30	42.30	29.00	11.30	18.00	14.40	8.3
318.0	194	61.30	40.20	21.10	13.80	19.80	10.60	5.7
327.0	426	67.50	41.50	26.00	15.20	17.20	12.70	8.9
291.0	417	56.60	35.60	21.00	9.40	13.40	10.00	22.2
320.0	365	63.20	40.80	22.40	13.50	26.30	13.70	4.2
325.0	264	68.90	42.60	26.30	13.20	17.60	9.20	8.9
318.0	323	61.90	38.30	23.60	16.60	21.60	11.10	5.9
347.0	257	62.00	35.00	27.00	5.00	6.00	#NULL!	5.0
323.0	333	75.80	40.90	34.90	6.90	16.00	12.80	7.9
319.0	273	62.30	41.50	20.80	9.90	19.80	14.80	6.4
316.0	240	64.10	40.30	23.80	19.20	25.20	8.90	5.8
309.0	355	74.50	50.30	24.20	15.70	29.40	29.00	14.6
306.0	590	78.00	38.30	39.70	17.40	33.20	20.50	3.4
351.0	252	68.00	35.00	33.00	12.00	9.00	#NULL!	19.1
353.0	288	68.20	39.10	29.10	155.10	217.50	178.00	10.3
338.0	125	65.90	43.40	22.50	12.00	20.10	10.00	10.3
352.0	354	75.40	44.70	30.70	16.50	19.20	12.50	9.0
351.0	334	69.30	44.60	24.70	#NULL!	34.60	27.00	8.1
315.0	439	71.00	44.00	27.00	11.70	26.30	11.60	9.6
347.0	298	65.20	41.00	24.20	32.00	30.20	15.50	4.1
333.0	212	68.90	44.70	24.20	8.70	15.50	15.50	16.0
334.0	292	73.60	45.80	27.80	13.20	25.10	14.10	7.8
339.0	294	58.80	39.40	19.40	11.10	18.00	8.00	3.8
349.0	212	63.20	39.50	23.70	18.30	20.50	17.30	4.6
346.0	328	63.30	41.80	21.50	24.60	29.00	12.20	8.9
318.0	178	63.60	40.80	22.80	48.80	44.60	39.70	9.7
340.0	349	72.30	43.00	29.30	39.30	35.70	2.40	6.7
313.0	351	63.40	42.50	20.90	25.00	17.50	23.90	23.7
350.0	185	64.90	44.30	20.60	17.70	22.40	10.10	15.8
328.0	384	64.10	38.20	25.90	491.10	654.30	725.20	15.1
341.0	220	67.50	41.90	25.60	22.50	28.30	9.00	7.4
352.0	210	66.00	42.00	24.00	12.00	10.00	#NULL!	10.0
339.0	302	75.80	46.80	29.00	44.70	31.40	28.00	4.8
332.0	287	72.70	46.70	26.00	14.50	24.90	13.40	9.2
317.0	201	66.70	42.00	24.70	14.30	28.20	11.10	6.7
342.0	252	57.80	36.90	20.90	22.20	36.70	10.10	4.4

229.0	272	65.30	40.30	25.00	20.20	32.30	9.20	8.7
340.0	317	69.10	42.30	26.80	12.00	26.60	10.50	8.2
283.0	385	65.10	40.10	25.00	31.30	52.80	25.90	4.8
346.0	231	67.20	46.40	20.80	18.10	24.50	9.50	15.3
355.0	254	71.70	43.90	27.80	19.50	27.40	8.90	11.8
335.0	189	64.60	39.20	25.40	15.80	28.60	8.50	7.5
346.0	270	75.00	45.00	30.00	333.00	30.00	15.60	7.1
347.0	249	71.90	45.40	26.50	19.30	28.50	11.30	31.6
324.0	593	43.30	21.20	22.10	36.10	30.50	34.20	3.8
354.0	273	76.00	44.90	31.10	14.90	19.90	13.80	9.8
331.0	282	69.20	44.00	25.20	11.40	24.20	17.30	6.8
334.0	241	66.20	44.00	22.20	14.60	28.10	10.80	5.2
327.0	303	74.90	45.20	29.70	30.00	28.00	#NULL!	12.8
294.0	87	56.30	31.90	24.40	26.80	40.10	13.60	21.6
333.0	308	69.20	40.70	28.50	18.50	33.20	10.90	10.5
346.0	320	67.70	42.20	25.50	26.20	38.70	17.70	6.6
331.0	277	62.90	42.70	20.20	24.00	36.30	10.90	15.8
349.0	321	77.50	45.50	32.00	20.10	29.20	14.20	15.1
347.0	280	66.30	43.60	22.70	30.50	25.50	26.00	7.6
341.0	212	74.00	43.00	31.00	16.60	29.10	16.30	14.0
326.0	513	69.30	39.10	30.20	26.00	21.00	#NULL!	6.0
344.0	339	67.00	41.80	25.20	12.70	25.30	#NULL!	8.7
335.0	244	67.80	45.40	22.40	29.20	27.10	15.80	15.0
336.0	215	70.30	39.60	30.70	430.30	349.80	89.30	21.3
354.0	278	62.00	36.00	26.00	8.00	7.00	#NULL!	4.0
344.0	340	57.80	37.70	20.10	27.00	26.90	9.70	7.9
335.0	222	74.00	46.20	27.80	11.40	25.50	8.00	19.2
339.0	214	67.00	38.60	28.40	31.90	48.50	21.30	10.0
315.0	251	73.20	35.90	37.30	12.00	12.40	20.50	9.2
317.0	139	58.90	30.30	28.60	51.30	70.90	99.60	22.6
332.0	216	67.30	40.70	26.60	22.40	34.80	13.80	7.6
346.0	280	72.10	48.70	23.40	16.00	25.60	10.20	15.1
321.0	162	56.90	37.10	19.80	16.80	33.90	13.50	14.8
273.0	408	73.80	43.10	30.70	16.90	22.10	11.00	13.6
339.0	256	71.10	45.70	25.40	15.60	21.80	10.50	14.0
333.0	247	63.80	37.30	26.50	70.10	29.10	82.30	13.0
320.0	269	64.10	38.90	25.20	12.80	20.30	8.70	9.0
341.0	281	61.50	40.00	21.50	22.30	25.10	16.00	12.5
331.0	532	54.60	30.40	24.20	11.80	22.10	9.70	5.8
343.0	317	56.50	41.90	14.60	17.50	27.30	14.80	12.2
394.0	263	59.40	36.40	23.00	16.00	25.60	10.80	6.2
329.0	241	63.00	34.00	29.00	12.00	10.00	#NULL!	5.0
345.0	240	64.70	41.80	22.90	24.50	26.00	11.40	10.4
315.0	405	83.20	29.90	53.30	22.30	44.90	9.50	6.3
327.0	139	60.70	23.00	37.70	45.00	123.00	183.00	35.0
348.0	297	65.60	42.40	23.20	17.20	23.20	15.20	11.1
336.0	394	60.50	34.60	25.90	27.20	44.90	30.70	10.2
342.0	137	64.70	39.70	25.00	6.90	21.30	15.40	8.7
324.0	332	68.40	38.70	29.70	17.00	23.80	15.00	8.6
368.0	269	72.30	44.40	27.90	26.00	18.00	#NULL!	14.0
334.0	407	63.80	37.00	26.80	21.60	34.90	18.60	6.1
348.0	288	70.30	43.70	26.60	22.20	37.10	10.20	27.5
316.0	472	58.10	32.30	25.80	13.90	11.70	13.10	10.1
346.0	171	71.70	43.20	28.50	22.80	29.80	15.20	66.0

324.0	227	69.30	40.90	28.40	16.80	23.40	13.70	13.6
369.0	290	66.00	35.00	31.00	8.00	8.00	#NULL!	6.0
348.0	367	84.70	41.90	42.80	15.50	25.20	12.00	7.5
337.0	276	67.40	43.60	23.80	14.10	13.10	16.70	12.5
325.0	220	68.50	40.50	28.00	11.50	30.30	10.00	7.5
333.0	176	67.60	45.30	22.30	19.50	30.80	9.30	9.6
338.0	335	58.30	29.80	28.50	21.90	27.00	13.60	8.4
339.0	192	59.50	35.20	24.30	12.50	28.60	9.10	3.8
317.0	272	57.00	35.70	21.30	14.40	20.50	7.60	15.4
346.0	208	63.40	34.10	29.30	15.00	34.70	10.70	9.6
332.0	555	67.70	38.90	28.80	9.10	22.40	10.60	7.9
341.0	257	69.70	42.70	27.00	27.60	25.30	13.10	8.0
341.0	267	70.20	43.30	26.90	22.30	30.90	10.00	11.5
338.0	255	66.30	41.70	24.60	14.30	18.00	11.80	12.4
337.0	193	60.00	32.00	28.00	7.00	5.00	#NULL!	8.0
347.0	241	50.50	32.90	17.60	29.00	58.30	10.40	12.7
368.0	283	70.40	45.80	24.60	13.40	26.20	12.30	11.2
343.0	289	68.50	41.50	27.00	27.80	30.00	20.90	11.0
366.0	230	72.50	44.80	27.70	16.10	28.50	13.00	10.3
342.0	174	53.00	32.00	21.00	17.20	20.00	16.20	20.3
334.0	62	67.30	17.60	49.70	86.60	131.90	20.00	72.4
336.0	239	70.10	41.30	28.80	23.00	33.10	8.80	8.2
330.0	191	73.50	44.10	29.40	9.80	30.60	13.50	20.4
352.0	248	67.40	42.30	25.10	18.10	61.20	10.30	6.7
351.0	292	75.20	44.10	31.10	21.90	34.70	10.10	17.8
340.0	199	62.70	38.70	24.00	15.00	20.70	17.10	19.1
321.0	180	66.20	45.50	20.70	20.10	33.20	19.70	5.7
345.0	239	67.00	43.10	23.90	13.00	12.00	#NULL!	9.0
338.0	290	64.00	34.00	30.00	9.00	7.00	#NULL!	7.0
315.0	231	68.00	37.00	31.00	11.00	11.00	#NULL!	8.0
354.0	554	53.80	29.40	24.40	10.70	19.90	8.60	9.2
312.0	364	69.60	45.10	24.50	17.70	27.70	15.40	6.9
344.0	563	74.70	41.20	33.50	11.50	24.60	18.20	10.9
323.0	333	68.40	42.10	26.30	17.10	28.20	9.70	7.8
292.0	514	68.30	33.00	35.30	8.20	22.10	9.20	9.5
358.0	305	74.50	40.60	33.90	13.00	25.10	10.30	9.8
340.0	557	56.60	31.00	25.60	77.20	46.00	32.40	9.4
350.0	252	64.20	41.20	23.00	5.40	18.80	#NULL!	7.1
341.0	219	41.70	25.40	16.30	20.50	38.60	19.80	8.2
342.0	248	51.50	36.10	15.40	15.70	27.40	7.40	10.0
347.0	259	61.70	32.70	29.00	277.00	102.00	174.90	8.5
333.0	260	62.00	32.00	30.00	12.00	10.00	#NULL!	5.0
341.0	275	58.10	37.50	20.60	38.70	22.40	#NULL!	12.2
342.0	316	71.70	44.00	27.70	18.20	26.60	12.70	12.0
337.0	323	66.60	43.10	23.50	28.50	20.30	16.20	5.7
348.0	121	59.30	33.20	26.10	113.00	132.00	304.00	33.2
327.0	540	54.90	31.80	23.10	179.00	55.00	122.00	90.7
342.0	255	68.80	44.50	24.30	26.00	23.10	#NULL!	11.8
332.0	327	84.00	50.70	33.30	20.30	27.00	#NULL!	16.8
330.0	373	66.70	42.60	24.10	19.70	27.70	#NULL!	13.1
344.0	298	62.00	32.00	30.00	10.00	8.00	#NULL!	9.0
320.0	251	74.50	48.00	26.50	45.40	52.60	12.80	10.5
355.0	296	68.60	45.70	22.90	20.60	26.70	9.40	11.8
319.0	402	68.00	44.00	24.00	28.30	34.70	16.20	7.6

28.0	443	64.00	41.70	22.30	17.40	27.60	8.30	8.0
338.0	267	67.40	44.40	23.00	11.10	22.50	15.10	9.7
332.0	390	63.00	33.00	30.00	8.00	7.00	#NULL!	10.0
345.0	227	38.90	26.10	12.80	9.40	25.40	3.80	5.4
345.0	242	61.00	32.00	29.00	26.10	58.70	#NULL!	12.0
371.0	237	63.30	42.20	21.10	12.30	20.50	11.50	14.9
354.0	234	66.00	44.70	21.30	12.00	14.00	#NULL!	10.0
350.0	209	66.00	35.00	31.00	9.00	8.00	#NULL!	5.0
337.0	297	62.70	39.60	23.10	16.20	23.70	10.60	5.4
339.0	258	75.50	48.20	27.30	12.60	31.10	8.50	5.5
336.0	305	58.30	37.30	21.00	17.90	28.30	17.80	4.4
343.0	202	73.40	45.90	27.50	12.80	26.70	13.80	20.7
347.0	119	69.20	40.70	28.50	24.80	29.90	19.00	26.5
337.0	259	69.00	45.60	23.40	13.70	24.00	22.20	9.2
324.0	293	71.20	38.60	32.60	14.70	26.60	65.90	13.1
364.0	137	66.70	43.70	23.00	24.30	20.20	14.70	22.2
341.0	299	67.10	44.60	22.50	8.30	32.70	8.00	8.0
349.0	194	68.10	43.20	24.90	13.70	22.30	9.40	8.9
337.0	211	76.50	48.30	28.20	18.20	26.50	#NULL!	9.9
339.0	270	63.10	42.60	20.50	12.00	28.60	7.80	7.2
352.0	196	61.30	24.60	36.70	191.00	239.00	201.00	311.0
353.0	232	68.00	44.10	23.90	11.10	12.50	9.20	5.9
344.0	223	62.00	31.00	31.00	7.00	6.00	#NULL!	8.0
330.0	204	72.70	47.70	25.00	19.50	25.70	#NULL!	17.1
330.0	282	61.00	31.00	30.00	8.00	7.00	#NULL!	14.0
308.0	168	60.00	32.00	28.00	6.00	5.00	#NULL!	5.0
337.0	209	64.00	42.70	21.30	10.10	31.00	#NULL!	14.2
331.0	197	64.70	42.70	22.00	34.20	40.30	9.90	19.3
337.0	230	57.80	36.20	21.60	42.20	24.10	49.90	8.6
369.0	296	69.10	9.10	60.00	25.70	20.10	#NULL!	9.1
345.0	236	65.00	44.10	20.90	12.00	20.00	#NULL!	11.0
352.0	156	62.60	41.90	20.70	22.70	28.70	15.80	9.5
335.0	266	64.00	34.00	30.00	7.00	6.00	#NULL!	8.0
325.0	210	63.50	44.70	18.80	11.90	22.70	9.20	16.8
353.0	393	62.00	33.00	29.00	9.00	8.00	#NULL!	9.0
325.0	209	67.90	43.10	24.80	15.20	28.80	#NULL!	10.3
345.0	219	77.70	49.40	28.30	13.90	36.10	9.60	13.8
342.0	253	65.00	47.20	17.80	13.00	17.00	#NULL!	12.0
339.0	250	72.10	41.40	30.70	22.00	26.00	11.00	2.5
351.0	271	77.00	43.60	33.40	53.30	53.00	50.00	7.2
351.0	169	57.00	35.20	21.80	12.70	19.20	17.00	7.6
332.0	280	62.50	34.90	27.60	30.50	23.40	19.00	6.7
281.0	383	54.60	27.60	27.00	174.70	292.00	1197.00	10.5
341.0	264	78.00	46.70	31.30	13.00	15.10	14.00	13.1
336.0	437	61.00	34.00	27.00	5.00	7.00	#NULL!	8.0
343.0	322	72.00	45.70	26.30	8.70	21.50	11.00	6.4
330.0	310	72.30	42.20	30.10	13.00	16.30	13.00	14.0
345.0	206	73.10	45.70	27.40	10.00	20.10	12.00	7.2
335.0	270	74.10	44.70	29.40	10.90	21.00	10.00	7.9
349.0	257	70.30	42.50	27.80	12.20	25.70	12.00	8.3
312.0	352	67.90	40.20	27.70	4.50	10.60	8.00	5.6
368.0	301	72.30	36.60	35.70	23.00	20.00	#NULL!	19.6
337.0	386	69.10	40.10	29.00	15.40	21.90	10.00	7.9
303.0	580	62.10	31.60	30.50	6.00	17.60	9.00	4.7

333.0	257	63.60	41.60	22.00	25.90	19.50	23.00	19.9
336.0	431	84.20	49.70	34.50	67.60	37.50	56.00	7.1
346.0	310	67.00	44.20	22.80	10.50	19.30	10.00	5.3
330.0	311	63.80	40.30	23.50	8.60	11.60	10.00	27.3
332.0	363	74.20	42.10	32.10	10.60	20.00	14.00	11.8
345.0	199	70.60	43.30	27.30	10.30	16.70	9.30	6.4
346.0	159	66.40	44.10	22.30	16.40	22.10	18.00	12.3
353.0	222	68.00	46.20	21.80	13.20	17.40	10.00	10.2
341.0	314	66.90	38.20	28.70	81.60	27.90	200.00	5.3
318.0	246	80.30	50.60	29.70	7.20	16.10	12.30	11.8
335.0	243	62.40	40.80	21.60	9.70	16.50	11.00	5.9
346.0	236	70.50	43.90	26.60	9.70	19.40	13.00	14.0
331.0	263	75.20	42.20	33.00	9.00	12.90	13.00	7.6
348.0	249	75.40	47.20	28.20	44.60	39.80	17.00	4.9
344.0	263	73.20	44.70	28.50	8.60	22.00	9.40	4.9
337.0	327	70.90	44.80	26.10	15.40	21.90	13.00	11.6
346.0	316	70.90	44.60	26.30	17.00	14.00	#NULL!	9.1
343.0	279	66.20	39.80	26.40	8.20	17.10	9.00	5.0
338.0	369	72.40	47.20	25.20	11.30	16.80	18.00	12.8
335.0	326	76.90	47.80	29.10	80.80	49.40	40.00	10.1
331.0	164	72.20	36.80	35.40	10.30	21.90	11.00	7.3
342.0	263	67.00	48.90	18.10	14.00	18.00	#NULL!	13.0
342.0	271	68.40	41.40	27.00	13.50	24.60	9.00	8.4
335.0	206	61.50	43.60	17.90	7.20	15.10	8.00	9.1
340.0	205	69.20	44.50	24.70	7.80	18.70	10.00	8.7
328.0	413	64.90	38.80	26.10	11.10	21.20	11.00	4.2
343.0	144	62.10	41.90	20.20	10.40	19.10	8.00	10.7
337.0	291	71.00	47.60	23.40	9.10	20.40	12.00	7.9
224.0	218	71.20	45.40	25.80	8.66	11.60	11.00	6.2
239.0	445	70.90	45.30	25.60	12.60	23.20	12.00	4.6
335.0	418	63.80	38.10	25.70	14.00	15.00	#NULL!	2.4
304.0	261	79.70	31.80	47.90	15.00	21.00	#NULL!	5.4
337.0	281	68.20	44.90	23.30	19.90	21.80	15.00	7.1
329.0	300	63.50	39.70	23.80	23.00	17.00	#NULL!	13.7
351.0	184	78.50	50.40	28.10	68.80	30.70	39.00	9.8
295.0	590	52.30	26.80	25.50	11.30	20.10	20.00	7.1
307.0	261	60.20	34.30	25.90	14.00	12.00	147.00	1.3
269.0	632	50.30	28.60	21.70	13.40	20.70	22.00	3.4
336.0	293	68.00	41.80	26.20	12.00	16.00	#NULL!	10.0
364.0	304	65.40	42.90	22.50	37.10	35.20	12.00	9.8
351.0	234	73.50	46.20	27.30	10.20	19.20	14.00	12.1
329.0	338	68.60	43.80	24.80	9.00	25.00	9.00	9.1
336.0	394	70.20	43.80	26.40	10.00	14.50	19.00	11.9
330.0	255	77.50	42.20	35.30	1.60	21.70	13.00	3.1
315.0	269	69.20	41.80	27.40	6.90	19.10	8.00	5.5
337.0	220	76.60	49.50	27.10	13.80	25.20	14.00	6.4
306.0	539	66.70	42.30	24.40	41.70	46.30	20.00	6.0
345.0	304	75.00	48.00	27.00	7.00	23.30	10.00	5.7
286.0	384	77.30	40.70	36.60	10.00	17.40	7.00	4.9
338.0	287	78.60	50.90	27.70	10.50	12.30	27.00	18.6
310.0	304	65.20	36.90	28.30	21.20	47.50	15.00	5.1
348.0	287	66.10	43.00	23.10	9.90	19.00	12.00	8.7
328.0	225	75.90	45.40	30.50	10.00	14.60	11.00	14.6
317.0	331	80.50	48.90	31.60	19.00	19.00	220.00	4.7

341.0	189	74.90	43.20	31.70	10.00	17.50	10.00	7.2
334.0	221	70.80	29.90	40.90	6.60	17.00	11.00	4.4
318.0	285	72.50	34.70	37.80	13.80	13.30	23.00	7.2
369.0	260	79.10	51.30	27.80	20.00	20.00	#NULL!	10.8
326.0	71	56.00	35.70	20.30	17.00	20.10	17.00	12.8
336.0	316	68.80	42.60	26.20	25.70	23.60	36.00	14.4
327.0	216	66.30	41.00	25.30	39.80	8.20	7.00	6.1
346.0	260	67.30	39.80	27.50	8.90	16.40	10.60	8.9
318.0	348	74.50	37.90	36.60	57.10	55.20	19.00	5.3
346.0	249	63.80	42.40	21.40	29.40	34.70	10.00	6.7
327.0	296	68.40	41.50	26.90	7.70	8.60	8.00	4.5
348.0	501	72.80	44.50	28.30	14.30	15.70	11.00	3.0
336.0	367	72.00	43.50	28.50	12.00	19.30	7.00	7.8
339.0	228	75.60	42.80	32.80	6.30	13.40	9.00	16.5
352.0	243	72.50	46.00	26.50	11.40	15.90	16.00	9.8
319.0	482	74.60	42.30	32.30	11.80	17.90	6.00	9.0
343.0	387	69.40	41.80	27.60	9.30	17.30	5.00	20.1
333.0	204	66.60	40.70	25.90	32.30	18.70	28.00	18.7
335.0	142	66.50	41.00	25.50	13.00	23.00	8.00	6.2
329.0	109	66.20	40.30	25.90	8.30	16.20	8.00	11.6
327.0	413	75.70	43.70	32.00	15.10	18.50	21.00	6.5
365.0	252	66.80	45.60	21.20	18.40	19.70	11.00	7.6
316.0	355	73.30	47.80	25.50	9.60	19.90	10.00	5.1
300.0	291	72.40	43.20	29.20	31.00	29.40	12.00	6.2
341.0	283	75.30	47.20	28.10	10.90	20.60	11.00	9.8
344.0	432	73.50	45.30	28.20	6.50	14.60	12.00	3.1
342.0	332	74.20	46.80	27.40	11.20	21.00	10.00	10.6
338.0	235	68.90	44.20	24.70	10.60	13.40	12.00	13.3
327.0	664	56.70	37.30	19.40	12.00	19.50	10.00	3.0
342.0	226	65.70	41.80	23.90	7.10	14.80	9.00	8.6
312.0	268	29.10	15.90	13.20	43.00	50.00	#NULL!	3.3
320.0	298	62.00	32.00	30.00	8.00	9.00	#NULL!	7.0
324.0	255	66.10	40.20	25.90	11.30	18.30	10.00	6.5
335.0	246	67.00	40.40	26.60	8.90	18.90	9.00	5.5
339.0	80	64.00	41.50	22.50	8.90	30.80	9.00	6.7
348.0	271	60.80	40.50	20.30	8.80	19.80	7.00	3.7
206.0	496	82.40	39.60	42.80	8.30	14.40	19.00	4.8
344.0	210	59.80	38.10	21.70	10.36	16.90	18.00	20.8
313.0	168	67.50	45.50	22.00	13.70	21.70	12.00	6.2
282.0	489	70.30	33.90	36.40	4.70	9.60	9.00	5.8
346.0	178	65.70	43.20	22.50	5.60	15.30	10.00	10.3
320.0	273	69.40	43.80	25.60	10.30	24.50	11.00	5.0
340.0	227	66.90	43.50	23.40	5.80	11.10	8.00	5.4
344.0	250	78.60	46.80	31.80	13.60	21.40	9.00	18.0
310.0	240	66.00	41.50	24.50	12.00	19.00	#NULL!	9.0
329.0	184	67.30	38.30	29.00	6.20	15.80	6.00	10.1
357.0	183	59.40	37.10	22.30	9.20	22.80	9.00	12.8
332.0	263	79.40	46.50	32.90	13.30	15.30	14.00	17.1
311.0	349	69.70	43.20	26.50	6.00	15.60	6.00	11.3
236.0	425	78.60	48.70	29.90	19.40	15.80	20.00	10.0
325.0	255	63.00	32.00	31.00	7.00	9.00	#NULL!	8.0
347.0	354	78.80	46.30	32.50	23.00	16.00	#NULL!	12.1
339.0	331	81.90	47.30	34.60	71.70	37.60	87.00	8.2
340.0	248	78.80	47.10	31.70	17.30	19.30	22.00	19.4

335.0	295	67.50	36.80	30.70	12.50	17.00	11.00	9.3
334.0	231	60.40	38.40	22.00	11.80	23.10	13.00	6.8
328.0	241	76.00	42.60	33.40	60.90	46.20	143.00	11.4
332.0	329	72.40	40.90	31.50	7.10	23.90	11.00	3.6
342.0	369	69.90	41.10	28.80	19.00	17.00	21.00	13.6
340.0	130	66.70	39.20	27.50	26.20	23.70	10.00	12.2
342.0	393	76.00	45.80	30.20	30.00	19.60	10.99	5.2
312.0	415	67.70	37.30	30.40	15.50	16.30	8.00	4.1
336.0	207	71.10	43.30	27.80	9.70	22.80	10.00	8.4
338.0	452	77.50	41.30	36.20	3.60	14.60	11.00	4.5
351.0	222	67.30	44.00	23.30	11.60	21.70	11.00	4.9
332.0	237	65.40	40.70	24.70	6.70	24.90	8.00	6.7
332.0	326	66.20	42.00	24.20	10.00	22.80	9.00	6.1
329.0	408	72.60	43.80	28.80	7.30	18.10	14.00	6.5
346.0	260	68.10	43.00	25.10	7.40	21.40	10.00	3.1
329.0	235	79.60	46.30	33.30	8.20	14.90	13.00	8.4
343.0	385	60.90	35.80	25.10	7.90	2.30	15.00	13.2
339.0	325	70.30	41.50	28.80	18.20	18.80	19.00	5.5
331.0	180	69.70	42.70	27.00	12.40	19.40	10.00	16.7
335.0	386	83.40	47.50	35.90	16.70	16.40	18.00	6.5
339.0	180	73.30	44.10	29.20	7.80	16.30	9.00	5.8
336.0	199	68.70	30.40	38.30	8.40	7.90	8.00	6.8
345.0	249	73.30	42.00	31.30	16.50	17.90	22.90	3.8
340.0	293	69.60	41.40	28.20	13.40	20.50	12.00	4.5
327.0	317	57.40	27.30	30.10	19.20	19.60	15.00	1.8
336.0	239	72.30	41.50	30.80	8.70	17.50	14.00	8.6
323.0	361	73.30	37.60	35.70	10.30	25.40	9.00	3.1
337.0	257	72.20	42.90	29.30	8.60	18.90	10.00	16.2
343.0	332	73.80	49.40	24.40	9.40	18.90	14.00	7.1
329.0	255	65.50	40.30	25.20	20.80	31.20	11.00	5.2
348.0	394	70.90	42.10	28.80	8.70	16.70	9.00	9.4
322.0	305	75.80	48.50	27.30	9.80	12.20	11.00	5.0
325.0	233	71.90	43.80	28.10	10.20	22.70	13.00	10.8
339.0	300	62.00	39.60	22.40	35.00	26.90	8.00	8.1
333.0	301	67.20	44.30	22.90	9.50	17.30	11.00	8.7
342.0	251	72.70	43.70	29.00	23.00	21.50	12.00	13.8
334.0	273	70.80	44.70	26.10	8.70	20.30	9.60	13.3
346.0	263	66.70	44.00	22.70	10.20	20.10	8.00	16.1
331.0	409	71.50	45.00	26.50	14.00	20.00	13.00	2.3
329.0	254	72.20	44.10	28.10	10.10	20.60	12.00	8.6
341.0	346	70.40	43.20	27.20	30.80	28.00	35.00	7.4
338.0	331	66.50	40.80	25.70	10.20	18.80	12.00	9.7
342.0	370	72.50	46.00	26.50	9.50	19.60	7.00	4.5
354.0	227	68.20	49.20	19.00	8.10	20.20	12.00	8.2
335.0	561	66.60	43.20	23.40	15.40	28.40	13.00	4.6
343.0	404	72.60	41.80	30.80	5.00	14.00	11.00	8.1
340.0	314	69.70	41.70	28.00	7.20	16.50	11.00	10.2
334.0	231	65.60	41.70	23.90	7.00	16.30	12.00	3.9
328.0	376	66.80	40.30	26.50	7.00	12.50	14.00	8.4
346.0	342	61.50	36.70	24.80	9.10	19.30	8.00	6.2
333.0	300	65.70	34.20	31.50	19.00	19.00	#NULL!	11.3
329.0	217	59.00	39.90	19.10	19.00	14.00	#NULL!	5.9
330.0	257	74.40	46.10	28.30	11.30	16.70	10.00	13.9
328.0	257	74.30	45.60	28.70	12.40	21.30	9.00	7.8

235.0	308	72.50	42.50	30.00	15.60	19.60	14.00	3.8
323.0	163	75.70	46.80	28.90	25.70	18.60	22.00	9.0
338.0	377	65.80	44.00	21.80	16.10	17.80	9.00	15.2
326.0	250	64.00	38.00	26.00	13.00	20.00	#NULL!	8.0
339.0	216	72.70	41.80	30.90	28.60	24.00	17.00	6.5
303.0	789	63.40	35.60	27.80	6.00	13.90	9.00	1.9
282.0	245	67.00	40.70	26.30	11.90	14.16	10.00	8.3
331.0	282	65.10	48.00	17.10	20.50	22.30	10.00	3.1
325.0	256	62.00	31.00	31.00	5.00	8.00	#NULL!	5.0
275.0	360	61.40	39.70	21.70	13.60	21.80	14.00	8.4
327.0	233	66.00	36.00	30.00	14.00	23.00	#NULL!	7.0
344.0	187	70.30	35.10	35.20	13.00	21.00	25.00	10.1
316.0	441	85.40	36.00	49.40	39.30	33.40	32.20	2.7
333.0	284	70.50	40.50	30.00	4.95	15.90	11.00	5.0
336.0	579	56.90	27.90	29.00	21.00	15.80	37.00	6.4
319.0	295	69.90	40.90	29.00	8.70	11.60	9.00	9.2
355.0	267	65.00	37.00	28.00	15.00	21.00	#NULL!	9.0
310.0	391	69.00	38.60	30.40	8.70	19.30	18.00	7.2
317.0	210	68.80	44.80	24.00	12.30	13.90	12.00	2.0
334.0	250	71.20	47.10	24.10	14.60	17.80	16.00	28.3
347.0	245	67.00	36.00	31.00	12.00	16.00	#NULL!	9.0
334.0	211	57.10	26.50	30.60	4.20	10.90	13.00	7.4
325.0	256	68.20	47.10	21.10	18.20	24.70	11.00	3.6
327.0	280	69.90	40.90	29.00	11.20	14.50	125.00	22.8
333.0	250	71.90	44.00	27.90	10.40	17.30	13.00	8.2
337.0	349	73.70	45.40	28.30	13.70	17.50	25.00	3.8
339.0	207	66.00	41.70	24.30	8.60	22.10	9.00	8.2
340.0	152	57.50	35.60	21.90	6.80	17.90	11.00	12.2
345.0	256	58.90	30.50	28.40	7.70	18.00	12.00	10.9
325.0	212	61.30	42.00	19.30	4.00	16.00	8.00	5.7
326.0	322	66.60	36.60	30.00	11.60	14.20	15.00	5.4
336.0	298	67.40	39.00	28.40	17.50	20.80	11.00	14.8
296.0	366	65.00	33.00	32.00	6.00	7.00	#NULL!	7.0
334.0	219	63.20	37.10	26.10	64.70	70.70	16.00	4.8
338.0	83	48.40	28.80	19.60	45.00	61.10	50.00	28.5
337.0	279	64.60	43.70	20.90	8.20	16.20	11.00	6.8
349.0	278	65.20	43.80	21.40	11.10	22.25	9.00	8.1
290.0	375	70.00	39.00	31.00	4.40	10.00	6.00	12.8
336.0	342	76.00	40.30	35.70	10.10	19.20	10.00	6.2
321.0	378	71.10	44.80	26.30	6.50	14.30	10.00	12.1
341.0	241	71.70	46.40	25.30	11.10	21.80	7.00	18.1
331.0	241	67.80	45.40	22.40	20.90	21.20	10.00	9.1
329.0	293	66.40	42.10	24.30	11.80	25.10	15.00	7.4
330.0	285	78.80	42.50	36.30	9.00	19.30	18.00	11.5
316.0	499	66.00	43.10	22.90	12.20	21.60	8.00	10.9
333.0	253	67.10	40.90	26.20	7.50	19.30	8.00	17.0
330.0	239	64.80	43.40	21.40	9.40	19.90	12.00	13.8
345.0	248	67.90	40.30	27.60	20.90	34.70	30.00	6.9
326.0	272	68.60	46.70	21.90	16.70	24.50	13.00	9.2
319.0	231	72.20	44.60	27.60	8.10	16.20	9.00	10.5
306.0	277	62.00	32.00	30.00	5.00	8.00	#NULL!	6.0
321.0	227	76.30	48.20	28.10	19.00	21.70	22.00	4.6
301.0	290	56.30	33.50	22.80	20.00	23.00	#NULL!	4.8
378.0	335	69.30	44.20	25.10	19.40	16.00	27.00	7.2

340.0	261	65.10	43.70	21.40	6.50	18.50	12.00	6.7
332.0	271	71.70	49.50	22.20	8.10	17.70	11.00	7.9
332.0	201	67.00	48.60	18.40	32.60	33.70	20.00	5.6
327.0	213	63.80	39.60	24.20	10.00	16.60	9.00	8.9
350.0	212	65.20	46.10	19.10	13.10	23.80	11.00	9.8
310.0	363	54.30	27.10	27.20	24.70	18.30	29.00	3.0
341.0	383	66.80	41.10	25.70	9.00	15.90	14.00	8.9
329.0	244	68.10	44.90	23.20	51.00	23.90	27.00	3.8
340.0	234	67.60	45.60	22.00	9.40	17.30	10.00	7.8
331.0	263	65.70	41.60	24.10	13.90	21.30	10.00	9.3
338.0	251	64.00	34.00	30.00	7.00	9.00	#NULL!	4.0
341.0	213	65.70	41.90	23.80	8.90	20.40	12.00	9.2
351.0	241	68.60	46.90	21.70	13.40	26.80	6.90	11.8
337.0	222	68.80	44.00	24.80	8.00	19.20	11.00	12.5
327.0	296	70.00	33.10	36.90	16.40	19.40	36.00	4.1
336.0	282	68.30	47.20	21.10	6.00	17.70	11.60	20.3
365.0	378	68.20	46.30	21.90	6.40	13.70	14.80	5.3
329.0	330	64.60	42.10	22.50	17.90	26.40	9.50	12.6
343.0	242	68.80	44.60	24.20	32.60	25.10	13.40	9.9
336.0	381	65.50	42.20	23.30	8.30	17.60	13.10	4.6
332.0	395	70.00	42.30	27.70	7.10	20.20	11.20	8.0
336.0	205	73.20	48.00	25.20	11.90	23.50	12.50	7.3
294.0	295	68.10	43.60	24.50	6.30	14.30	11.30	7.1
322.0	229	66.70	42.20	24.50	8.00	19.70	10.80	10.6
327.0	316	67.70	43.90	23.80	7.20	17.70	15.80	5.0
332.0	308	60.60	42.10	18.50	10.40	16.30	12.30	4.6
330.0	283	76.90	44.60	32.30	12.00	22.00	18.00	8.1
331.0	232	70.90	45.70	25.20	8.20	16.10	14.30	6.9
314.0	286	61.80	43.10	18.70	12.40	18.50	14.40	9.6
338.0	270	65.60	41.60	24.00	13.90	22.80	7.90	12.3
338.0	207	71.30	42.30	29.00	19.70	27.30	10.70	11.5
321.0	331	66.00	39.20	26.80	15.80	20.80	12.20	8.0
319.0	215	66.50	48.70	17.80	4.80	19.80	116.90	14.6
312.0	290	64.40	39.30	25.10	4.40	9.00	7.10	4.6
327.0	297	63.40	40.80	22.60	6.30	16.80	9.90	9.7
334.0	223	62.00	32.00	30.00	8.00	8.00	#NULL!	8.0
336.0	325	75.20	47.70	27.50	10.40	25.00	6.40	6.5
328.0	295	74.20	45.00	29.20	9.20	13.10	12.60	11.7
336.0	265	63.00	32.00	31.00	9.00	5.00	#NULL!	5.0
342.0	287	64.20	45.10	19.10	19.10	36.50	34.40	9.9
337.0	315	68.70	41.50	27.20	18.50	16.30	30.70	3.7
326.0	324	72.20	38.80	33.40	33.40	12.90	15.10	8.8
303.0	311	72.10	43.20	28.90	7.10	21.80	11.70	2.8
338.0	51	63.40	40.50	22.90	22.50	31.00	38.70	24.1
300.0	252	67.50	39.10	28.40	26.00	30.00	#NULL!	4.2
279.0	392	65.00	41.10	23.90	28.00	24.00	10.80	4.4
345.0	239	66.50	42.40	24.10	29.40	31.40	10.70	10.9
348.0	216	62.80	42.40	20.40	16.60	25.50	12.30	6.9
324.0	248	70.70	46.70	24.00	24.00	20.30	14.30	9.2
330.0	276	68.70	45.70	23.00	12.50	19.10	13.50	8.8
334.0	251	70.30	42.90	27.40	27.40	20.10	14.40	7.1
344.0	327	60.90	40.00	20.90	9.10	15.80	7.70	14.0
332.0	236	63.70	41.10	22.60	13.40	16.20	10.70	6.4
343.0	191	67.20	39.40	27.80	8.50	20.60	12.30	6.6

304.0	479	75.10	37.70	37.40	35.40	40.40	5.20	5.6
335.0	125	61.10	42.20	18.90	16.20	19.50	10.70	8.2
319.0	271	66.60	42.80	23.80	29.80	37.60	22.00	1.7
326.0	279	57.70	36.60	21.10	7.70	15.90	7.20	8.4
333.0	279	63.30	43.20	20.10	10.20	14.50	9.40	19.6
336.0	209	75.50	49.40	26.10	9.70	22.50	13.60	9.2
346.0	207	66.40	41.80	24.60	19.70	24.80	14.20	7.7
333.0	186	69.90	46.50	23.40	10.30	16.50	12.00	23.4
357.0	260	64.80	41.00	23.80	10.00	20.70	10.00	13.3
343.0	182	78.50	47.80	30.70	32.90	34.00	17.00	15.4
317.0	333	65.20	40.70	24.50	7.10	17.00	11.50	8.5
340.0	343	69.10	41.80	27.30	4.90	13.00	6.80	9.8
367.0	282	70.80	41.90	28.90	10.20	21.60	10.00	3.4
339.0	345	76.10	42.80	33.30	7.40	18.70	10.00	9.4
331.0	303	80.60	47.80	32.80	12.30	26.60	12.00	5.2
356.0	187	76.20	46.80	29.40	10.10	19.90	16.00	19.7
269.0	775	69.40	42.60	26.80	4.50	10.50	22.00	12.3
344.0	208	70.00	42.60	27.40	11.00	14.80	9.00	11.6
346.0	217	68.90	38.50	30.40	6.00	17.30	9.00	17.3
345.0	284	84.10	50.10	34.00	24.00	14.00	34.00	14.3
338.0	316	76.70	46.10	30.60	11.80	30.90	10.00	8.2
356.0	203	69.00	42.70	26.30	8.40	14.00	14.00	8.6
338.0	313	66.50	41.00	25.50	11.40	21.70	9.00	10.4
349.0	347	70.80	44.00	26.80	12.50	14.30	13.00	21.4
340.0	281	72.60	44.00	28.60	15.50	22.50	55.30	2.8
350.0	256	68.80	40.30	28.50	8.40	19.20	9.00	7.9
345.0	484	59.20	24.90	34.30	11.70	11.90	60.00	10.9
343.0	304	80.00	44.10	35.90	9.90	20.60	4.00	4.7
344.0	367	65.80	43.70	22.10	10.70	20.60	11.00	6.2
341.0	234	71.90	42.80	29.10	7.00	13.90	8.00	16.9
328.0	337	58.50	26.10	32.40	21.80	19.60	56.00	6.6
330.0	250	60.20	32.70	27.50	0.70	13.00	8.90	8.5
332.0	141	64.20	41.40	22.80	12.20	21.30	11.00	11.9
323.0	469	55.20	31.90	23.30	4.20	10.60	9.00	6.3
330.0	402	78.30	47.20	31.10	12.80	20.40	15.00	5.3
#NULL!	#NULL!	75.00	45.10	29.90	19.90	22.40	16.00	6.6
328.0	273	66.00	42.50	23.50	10.00	16.60	10.90	13.3
329.0	289	69.50	43.30	26.20	5.00	8.50	14.00	11.9
334.0	281	67.10	40.80	26.30	14.20	25.50	11.00	5.1
326.0	551	67.80	43.60	24.20	29.00	25.70	28.00	10.2
335.0	237	64.60	39.80	24.80	6.20	18.90	9.40	7.5
353.0	213	69.20	42.90	26.30	14.30	23.70	8.00	8.3
340.0	183	70.20	44.00	26.20	6.40	18.00	13.00	13.5
344.0	221	63.70	40.50	23.20	11.00	15.30	12.00	4.6
330.0	366	74.30	44.20	30.10	5.80	15.40	9.30	6.1
329.0	201	63.40	39.70	23.70	7.10	11.40	13.00	7.3
332.0	240	63.10	42.50	20.60	2.70	13.30	8.00	6.2
328.0	214	64.80	37.10	27.70	17.90	21.50	8.00	10.3
344.0	280	67.90	41.20	26.70	11.60	15.50	9.00	7.0
341.0	202	63.90	42.10	21.80	12.00	15.10	11.00	3.9
356.0	402	67.70	42.00	25.70	8.70	18.90	7.00	4.7
337.0	356	52.70	22.30	30.40	4.10	9.20	11.00	2.6
331.0	214	63.50	42.20	21.30	7.10	16.40	7.00	6.4
326.0	300	67.30	41.20	26.10	7.70	22.30	11.00	12.5

330.0	268	76.90	46.80	30.10	18.00	15.00	9.00	10.0
348.0	271	69.30	42.90	26.40	16.00	18.00	#NULL!	6.6
341.0	282	72.30	44.60	27.70	6.60	9.80	9.00	14.0
338.0	227	76.80	45.00	31.80	22.00	27.00	#NULL!	6.5
325.0	105	70.30	45.10	25.20	10.70	21.60	9.00	9.2
337.0	330	71.60	41.80	29.80	8.40	25.60	13.00	5.6
325.0	292	76.50	46.00	30.50	21.90	23.10	19.00	11.8
324.0	279	68.40	44.10	24.30	11.80	14.70	3.00	9.3
324.0	363	72.10	45.10	27.00	13.30	18.40	15.50	4.2
339.0	283	70.70	39.90	30.80	10.90	18.60	10.00	9.7
319.0	269	75.20	43.40	31.80	12.00	24.00	10.90	5.2
330.0	369	67.50	39.10	28.40	12.90	21.40	9.00	1.2
311.0	306	79.70	46.20	33.50	8.50	20.80	11.00	6.8
326.0	333	74.20	46.80	27.40	13.70	22.80	12.00	2.5
292.0	417	78.50	48.30	30.20	9.60	18.10	12.00	5.7
340.0	263	73.60	43.70	29.90	16.60	17.20	17.00	4.4
321.0	226	64.20	40.50	23.70	19.90	27.50	12.00	12.7
332.0	254	71.50	42.80	28.70	27.10	26.60	13.00	4.9
331.0	308	67.00	38.70	28.30	8.30	16.20	8.00	11.0
322.0	285	67.20	41.30	25.90	11.20	16.00	9.00	5.9
342.0	413	65.50	41.30	24.20	9.80	21.10	7.00	2.9
341.0	267	67.20	40.50	26.70	14.50	16.40	12.00	5.6
314.0	354	73.30	45.30	28.00	11.70	25.30	9.00	9.5
345.0	469	62.40	32.30	30.10	11.90	11.00	16.00	4.1
324.0	239	69.10	43.40	25.70	7.40	14.60	8.00	8.9
333.0	246	64.00	41.80	22.20	4.50	9.40	12.00	11.5
300.0	279	67.30	42.60	24.70	11.20	16.10	9.00	9.0
328.0	189	73.90	43.10	30.80	11.80	21.10	12.00	4.8
317.0	553	81.20	38.40	42.80	7.30	9.50	12.00	6.8
337.0	306	87.90	49.70	38.20	39.00	20.50	44.00	4.8
333.0	345	75.70	45.10	30.60	10.40	21.60	13.00	5.2
333.0	47	50.60	26.60	24.00	76.70	118.90	370.00	10.0
345.0	320	64.00	38.60	25.40	13.10	19.30	12.00	5.3
338.0	186	73.90	42.50	31.40	10.00	16.90	11.00	6.1
337.0	300	63.70	39.80	23.90	10.50	15.50	19.00	12.6
333.0	369	72.00	46.70	25.30	8.50	13.70	15.80	7.7
333.0	280	65.00	38.40	26.60	13.00	20.00	#NULL!	3.6
323.0	249	73.60	49.00	24.60	10.50	18.50	12.90	6.4
327.0	197	66.80	41.00	25.80	8.90	17.40	9.00	11.3
349.0	294	50.60	24.40	26.20	24.00	16.00	#NULL!	5.5
323.0	309	64.40	37.80	26.60	14.20	31.30	12.60	3.1
348.0	272	68.50	41.70	26.80	11.90	14.50	9.00	7.5
350.0	303	62.40	39.40	23.00	8.60	13.40	11.00	10.7
328.0	256	65.50	41.90	23.60	17.70	29.50	11.10	6.3
350.0	281	61.30	37.70	19.80	18.00	9.00	14.00	7.7
345.0	335	68.60	41.10	27.50	8.50	17.90	8.60	4.0
330.0	353	57.00	36.80	20.20	77.10	45.20	90.30	13.1
352.0	294	61.90	40.00	21.90	8.50	19.70	10.90	4.9
329.0	355	76.10	49.60	26.50	12.90	18.50	12.00	6.1
357.0	256	74.10	46.20	27.90	12.60	19.00	12.00	14.8
355.0	329	71.80	47.20	24.60	13.70	19.90	15.50	10.0
340.0	286	69.70	40.70	29.00	3.50	21.40	8.60	1.9
351.0	249	65.30	41.00	24.30	7.30	25.70	8.50	13.6
330.0	361	70.20	42.40	27.80	8.40	14.20	9.80	5.0

346.0	231	61.90	39.50	22.40	12.10	18.80	13.00	9.0
341.0	333	75.40	45.40	30.00	27.00	19.00	18.10	8.0
327.0	218	74.10	48.20	25.90	6.30	26.20	10.00	17.5
320.0	217	70.10	39.80	30.30	4.50	21.80	13.80	6.2
328.0	249	62.10	38.00	24.10	21.10	28.40	10.00	4.1
339.0	139	61.60	35.60	26.00	12.10	15.90	6.00	2.4
332.0	277	40.10	14.00	29.90	26.00	17.40	82.00	3.6
340.0	310	70.60	43.20	27.40	14.90	20.50	8.00	11.7
354.0	380	93.40	46.50	46.90	24.00	22.00	#NULL!	31.3
312.0	307	73.30	43.20	30.10	5.80	16.40	6.30	5.2
337.0	350	67.30	41.60	25.70	16.00	30.70	7.00	16.0
349.0	329	65.20	41.80	23.40	27.40	32.10	15.70	11.3
345.0	356	70.90	43.20	27.70	22.00	23.90	13.20	8.5
310.0	8	45.20	23.70	21.50	17.00	8.00	6.00	20.2
333.0	358	66.10	40.80	25.30	14.30	23.10	9.00	6.3
327.0	233	70.60	44.80	25.80	6.60	19.00	11.00	15.0
346.0	221	60.10	36.60	23.50	10.10	16.40	11.00	7.6
328.0	324	67.30	44.10	23.20	14.00	20.90	9.00	6.2
327.0	298	68.90	40.30	28.60	9.70	12.30	9.00	13.4
329.0	209	71.00	43.30	27.70	7.10	20.10	10.00	2.8
319.0	286	72.20	44.80	27.40	7.90	16.90	7.00	7.1
326.0	277	68.10	42.80	25.30	9.30	15.40	15.00	8.9
339.0	574	66.30	39.70	26.60	8.50	23.70	9.00	2.8
331.0	338	65.50	42.30	23.20	7.10	16.10	11.00	17.2
340.0	248	68.70	44.70	24.00	7.70	16.20	12.00	9.3
340.0	295	68.70	43.50	25.20	11.50	14.50	13.00	14.8
331.0	403	79.10	44.90	34.20	9.50	19.90	15.00	6.5
333.0	310	69.20	45.20	24.00	11.70	18.00	14.00	23.1
338.0	464	72.60	42.20	30.40	36.40	29.50	21.00	4.7
334.0	185	70.80	42.60	28.20	1.70	12.50	14.00	11.9
362.0	314	68.50	41.60	26.90	10.20	24.40	8.00	14.7
354.0	302	73.20	46.90	26.30	8.90	26.90	14.00	11.7
331.0	303	70.80	41.80	29.00	10.40	16.90	10.00	6.8
314.0	346	63.90	35.90	28.00	12.30	19.20	12.00	8.5
352.0	368	74.40	46.80	27.60	8.90	19.80	11.00	6.6
328.0	242	71.60	41.20	30.40	7.90	15.80	14.00	12.4
336.0	391	74.60	45.10	29.20	6.70	22.40	12.00	8.6
326.0	316	70.70	43.10	27.60	4.40	13.50	10.00	14.3
336.0	286	73.30	45.00	28.30	11.70	17.90	14.40	5.8
333.0	311	69.80	45.50	24.30	12.60	17.00	21.00	9.6
336.0	323	71.20	41.10	30.10	11.00	13.80	13.00	9.3
348.0	258	67.80	44.50	23.30	16.60	23.50	13.10	5.5
333.0	242	65.30	44.00	21.30	12.80	24.90	11.00	12.9
344.0	328	71.20	41.90	29.30	16.10	19.00	11.00	5.9
310.0	361	87.30	39.60	53.50	48.60	58.90	20.00	4.9
350.0	270	64.70	40.20	24.50	8.00	11.80	13.00	11.8
340.0	279	68.90	41.00	27.90	3.90	12.50	13.00	9.6
347.0	290	74.70	47.50	27.20	20.00	16.00	12.00	9.3
343.0	309	73.00	45.10	27.90	8.90	23.10	14.00	8.3
328.0	253	60.30	39.10	21.20	12.70	20.70	11.00	1.8
324.0	240	67.70	39.80	27.90	4.40	17.50	11.00	4.4
337.0	172	70.80	44.70	26.10	22.50	23.90	12.00	5.1
259.0	607	73.10	42.00	31.10	10.70	20.50	10.00	5.4
343.0	297	76.20	45.10	31.10	9.00	17.00	10.00	11.6

#NULL!	#NULL!	74.30	47.30	27.00	10.30	22.90	19.00	8.4
364.0	289	74.60	48.90	25.70	9.90	27.70	10.00	10.0
341.0	293	63.60	38.10	25.50	8.40	17.60	8.00	6.5
319.0	309	74.30	44.60	29.70	30.10	17.40	20.00	4.9
337.0	262	61.50	40.10	21.40	16.80	15.90	13.00	10.9
349.0	328	71.50	46.10	25.40	45.40	26.50	27.00	19.5
320.0	259	85.80	50.30	35.50	12.20	16.40	12.00	12.1
338.0	259	61.70	39.20	22.50	2.30	9.10	12.00	17.2
329.0	118	64.40	43.70	20.70	29.00	25.80	48.00	16.4
322.0	293	80.00	40.30	39.70	13.00	19.00	8.00	10.7
339.0	306	64.40	41.20	23.20	13.00	17.50	11.00	8.8
343.0	237	69.80	45.10	24.70	14.30	17.10	10.00	7.5
335.0	313	70.40	42.10	28.30	10.40	19.80	10.00	5.4
349.0	297	72.10	46.60	25.50	13.20	30.50	13.00	7.3
253.0	248	55.00	35.00	20.00	19.00	20.00	8.00	4.1
296.0	511	67.70	42.70	25.00	7.90	19.50	10.00	2.0
340.0	316	68.20	45.10	23.10	22.20	28.00	14.00	10.6
358.0	267	71.40	43.40	28.00	11.40	24.50	15.00	6.3
334.0	344	66.80	40.60	26.20	5.60	15.40	13.30	3.1
330.0	334	67.50	42.70	24.80	6.80	19.60	12.00	2.0
329.0	204	75.40	43.80	31.60	7.80	14.70	13.50	27.8
339.0	290	73.80	43.70	30.10	8.20	18.30	11.00	12.7
344.0	240	71.80	46.40	25.40	4.50	12.10	8.00	11.3
321.0	371	79.70	41.30	38.40	4.10	12.40	13.00	9.1
305.0	107	48.70	26.60	22.10	59.00	56.00	90.40	22.2
308.0	176	69.50	37.00	32.50	8.70	10.20	13.00	7.4
330.0	199	68.80	40.80	28.00	8.90	23.50	11.00	11.6
331.0	279	68.50	41.20	27.30	6.00	13.60	10.00	13.8
338.0	375	66.00	39.00	27.00	13.30	22.90	9.00	2.0
367.0	297	64.90	42.80	22.10	10.80	19.20	9.00	11.5
318.0	236	69.50	44.70	24.80	8.10	17.70	9.00	10.6
334.0	375	74.40	46.40	28.00	518.10	159.90	292.00	58.3
333.0	367	68.60	42.20	26.40	13.60	18.00	13.00	6.4
331.0	275	72.80	45.00	27.80	15.80	24.50	17.00	11.6
354.0	212	69.40	40.60	28.80	22.00	15.00	20.00	5.7
339.0	295	73.40	43.70	29.70	51.10	43.40	18.00	21.0
370.0	391	62.30	41.20	21.10	13.40	18.80	9.90	13.3
351.0	268	68.20	44.20	24.00	15.70	16.20	23.00	5.2
348.0	252	65.80	40.80	25.00	16.20	16.90	14.00	10.2
348.0	349	75.60	48.10	27.50	10.10	29.80	9.00	11.0
321.0	351	66.30	38.20	28.10	37.00	27.00	#NULL!	1.2
346.0	202	75.00	49.00	26.00	38.10	44.90	6.00	11.4
328.0	300	74.60	44.90	29.70	9.90	23.20	14.00	6.4
336.0	261	76.20	47.50	28.70	12.10	22.90	14.00	10.0
346.0	459	67.70	40.60	27.10	7.60	32.50	11.50	4.7
349.0	339	71.70	47.70	24.00	20.50	21.20	17.00	7.3
327.0	245	70.10	43.30	26.80	58.40	51.10	19.00	8.6
347.0	154	73.70	43.30	30.40	14.70	24.80	10.00	9.1
321.0	188	73.00	41.50	31.50	64.80	34.20	44.00	8.3
332.0	271	65.60	35.90	29.70	4.00	15.50	11.00	9.0
339.0	325	73.20	48.00	24.30	9.30	24.60	11.00	5.9
355.0	271	67.90	46.60	21.30	17.40	26.60	14.00	4.4
360.0	318	80.20	48.00	32.20	8.90	19.20	14.00	14.5
343.0	316	76.20	46.70	29.50	11.50	18.80	14.00	7.2

339.0	218	68.50	43.10	25.40	7.00	19.50	17.00	11.5
326.0	274	80.70	49.40	31.30	10.50	16.60	17.00	17.3
349.0	251	64.90	41.80	23.10	7.50	19.20	10.00	8.9
346.0	255	78.80	47.90	30.90	14.10	23.00	18.00	15.4
344.0	299	67.70	41.80	25.90	12.30	16.70	25.00	14.6
345.0	245	66.80	43.00	23.80	8.10	17.30	9.60	11.8
357.0	320	80.10	48.00	32.10	60.80	36.20	45.20	7.4
332.0	238	79.70	44.30	35.40	18.00	14.00	#NULL!	5.9
356.0	244	64.90	43.40	21.50	11.20	18.40	10.10	8.6
339.0	321	67.00	45.20	21.80	8.50	18.50	8.50	4.0
326.0	294	65.40	37.70	27.70	8.90	20.30	12.00	8.5
332.0	189	76.40	46.30	30.10	12.10	21.40	13.00	8.6
340.0	300	74.30	47.00	27.30	12.00	19.90	15.00	10.7
317.0	311	72.50	42.60	29.90	7.70	14.60	13.00	7.7
349.0	298	72.40	41.70	30.70	10.60	22.40	17.00	6.3
358.0	340	70.10	42.80	27.30	9.50	16.40	13.00	15.5
354.0	256	74.40	44.30	30.10	25.90	25.20	16.00	18.7
240.0	393	62.00	35.90	26.10	1.70	24.00	69.00	12.5
342.0	361	77.60	44.00	33.60	56.50	29.10	45.00	5.8
#NULL!	295	66.20	41.90	24.30	12.40	16.40	12.00	5.8
332.0	323	69.60	45.60	24.00	7.30	13.40	17.00	7.2
358.0	335	72.90	49.50	23.40	19.40	20.20	35.00	3.2
327.0	205	68.00	40.30	27.70	40.00	38.90	229.00	8.3
358.0	324	65.00	40.40	24.60	13.80	16.00	15.00	4.4
323.0	275	68.90	42.10	26.80	10.00	21.70	12.00	3.8
339.0	442	71.10	43.00	28.10	7.30	15.90	10.00	4.7
339.0	402	62.50	36.40	26.10	24.00	25.00	13.30	9.9
352.0	347	67.30	42.00	25.30	17.70	25.50	16.00	7.4
331.0	211	74.50	42.10	32.40	25.00	17.00	#NULL!	6.6
343.0	247	63.70	39.90	23.80	7.60	15.40	12.00	8.9
329.0	316	71.80	48.10	23.70	11.90	27.70	13.00	6.7
339.0	254	60.90	40.20	20.70	24.00	28.30	15.00	2.4
346.0	251	61.90	35.10	26.80	10.10	16.10	16.00	4.0
367.0	277	64.70	44.90	19.80	4.40	16.40	8.90	14.4
338.0	248	63.20	40.50	22.70	6.40	16.40	11.00	4.6
323.0	378	71.20	40.70	30.50	13.90	26.40	10.00	5.5
346.0	277	66.50	39.80	26.70	8.20	23.60	10.00	9.3
342.0	237	69.80	44.90	24.90	8.10	17.40	10.00	5.0
339.0	325	76.30	46.40	29.90	14.80	19.80	17.00	5.8
340.0	326	76.40	44.50	31.90	6.00	13.40	16.00	9.4
351.0	248	66.40	40.60	25.80	7.90	22.30	9.00	3.5
317.0	28	66.20	45.90	20.30	20.50	34.40	10.00	17.7
284.0	423	75.80	48.10	27.70	12.70	19.50	28.00	6.2
348.0	388	66.60	41.30	25.30	14.30	19.30	7.00	7.0
350.0	258	71.50	46.20	25.30	8.80	18.90	10.00	4.6
351.0	348	76.00	47.60	28.40	15.80	21.90	18.00	5.8
330.0	480	64.80	43.00	21.80	27.40	28.50	13.00	4.5
333.0	319	68.30	42.30	26.00	6.40	11.50	9.00	10.0
334.0	229	63.60	41.40	22.20	56.50	45.60	14.00	14.1
344.0	350	68.00	39.90	28.10	5.90	9.10	9.00	9.6
326.0	274	80.20	49.00	31.20	22.30	22.60	13.00	24.7
336.0	279	71.60	48.00	23.60	10.10	23.90	12.00	5.7
328.0	337	68.30	42.00	26.30	11.20	20.70	10.00	7.2
335.0	320	71.20	44.60	26.60	9.60	15.80	11.00	6.7

338.0	415	78.60	45.30	33.30	8.30	19.30	13.00	3.2
343.0	167	75.00	49.00	26.00	21.10	34.50	9.00	5.5
320.0	377	65.60	40.20	25.40	9.50	24.10	9.00	6.5
343.0	422	61.40	39.30	22.10	7.60	17.40	7.90	8.3
321.0	379	70.90	47.00	23.90	13.00	29.60	10.00	9.1
330.0	314	67.90	44.90	23.00	9.30	24.20	6.00	8.5
340.0	358	77.50	46.10	31.40	57.10	46.20	46.00	10.2
337.0	432	73.30	46.30	27.00	19.30	15.90	23.00	7.7
349.0	222	84.30	48.40	35.90	20.00	16.00	14.00	8.7
331.0	318	71.30	44.10	27.20	5.60	9.50	15.00	6.6
344.0	201	72.00	48.10	23.90	11.00	23.30	8.00	5.4
335.0	289	67.80	40.10	27.70	10.90	24.40	7.00	5.1
#NULL!	259	67.10	42.80	24.30	6.00	14.50	11.00	9.3
330.0	316	74.60	48.60	26.00	28.20	25.40	16.00	5.6
339.0	403	69.70	44.00	25.70	11.70	23.40	11.50	13.0
295.0	278	61.30	35.70	25.60	16.70	23.30	9.40	6.1
348.0	286	69.50	46.30	23.20	13.70	23.40	15.00	13.7
334.0	308	75.20	47.80	27.40	6.20	16.20	9.00	12.2
341.0	290	72.00	46.60	25.40	8.90	19.00	11.00	10.9
350.0	298	64.50	42.00	22.50	9.50	17.60	12.00	4.1
319.0	277	63.00	40.10	22.90	9.70	27.50	9.00	1.3
338.0	244	72.30	44.30	28.00	10.30	16.40	8.00	20.2
350.0	232	73.50	47.80	25.70	8.70	15.70	11.00	9.9
329.0	373	81.20	44.90	42.10	23.00	17.00	#NULL!	12.3
347.0	230	74.50	44.70	29.80	17.20	20.70	16.00	18.5
334.0	190	67.50	41.50	26.00	15.50	27.00	10.00	6.9
328.0	186	52.70	37.90	14.80	35.60	28.40	36.00	15.5
333.0	274	74.10	45.70	28.40	12.40	17.70	10.00	11.7
339.0	237	70.20	44.10	26.10	8.80	18.70	9.00	11.2
311.0	466	67.70	40.00	27.70	10.90	19.20	17.60	5.0
320.0	294	74.90	41.10	33.80	24.00	23.00	#NULL!	10.2
345.0	404	66.80	40.50	26.30	12.40	21.60	10.30	4.1
338.0	252	76.30	41.00	35.30	18.00	19.00	12.00	9.2
325.0	337	66.70	39.40	27.30	13.70	20.00	9.00	5.5
351.0	260	64.20	42.20	22.00	17.70	18.50	28.00	6.2
328.0	395	74.50	41.70	32.80	15.50	24.00	9.00	3.1
330.0	314	66.60	42.90	23.70	10.20	12.20	11.00	3.4
339.0	75	71.40	42.00	40.40	8.40	16.40	17.00	6.6
339.0	201	69.40	47.10	22.30	14.50	19.90	12.00	4.4
349.0	216	65.80	38.60	27.20	23.00	20.00	14.00	9.5
327.0	330	68.90	44.60	24.30	9.50	15.60	9.00	8.9
354.0	431	75.70	33.90	41.80	12.00	15.00	13.00	13.7
338.0	333	70.40	42.30	28.10	5.70	15.70	16.00	3.3
332.0	388	71.20	42.80	28.40	16.40	18.90	23.00	11.3
319.0	269	68.50	39.90	28.60	6.90	13.00	15.00	5.6
347.0	320	71.40	47.10	24.30	18.50	29.00	17.00	7.8
322.0	366	76.60	44.60	32.00	8.70	15.70	12.00	10.8
343.0	283	70.70	47.20	23.50	8.50	20.90	12.00	5.0
343.0	366	64.70	41.70	23.00	5.30	14.40	11.00	7.7
368.0	27	84.60	36.10	36.00	80.00	108.00	124.00	42.3
343.0	257	67.80	43.00	24.80	11.70	24.50	11.00	7.7
325.0	197	70.80	43.20	27.60	8.20	15.80	7.00	7.7
341.0	287	65.20	42.30	22.90	10.50	22.60	8.00	5.3
332.0	353	74.00	44.20	29.80	11.80	26.10	12.00	8.0

338.0	272	74.00	48.20	25.80	7.10	17.70	15.00	9.3
345.0	338	71.60	41.50	30.10	13.80	21.60	10.00	3.3
348.0	297	67.60	40.70	26.90	6.50	18.30	8.00	5.5
340.0	221	71.40	44.80	26.60	7.00	11.60	11.00	10.7
339.0	262	70.40	46.30	24.10	10.90	17.60	9.00	12.1
341.0	278	71.60	44.60	27.00	9.30	24.20	7.00	9.8
332.0	217	73.20	45.20	28.00	37.50	28.80	20.00	13.3
325.0	179	71.00	46.20	24.80	10.90	21.10	11.00	5.6
348.0	312	66.20	42.40	23.80	15.40	19.50	12.10	7.3
333.0	375	71.50	43.70	27.80	17.10	32.90	10.00	4.1
339.0	294	66.90	40.30	26.60	6.70	11.00	11.00	4.5
341.0	297	65.70	45.60	20.10	12.30	21.40	10.00	8.9
333.0	323	72.00	45.20	26.80	14.70	19.90	11.00	9.6
333.0	233	70.80	44.60	26.20	7.90	20.30	8.00	12.5
335.0	161	80.90	50.30	30.60	60.10	46.30	36.00	19.2
345.0	267	63.40	42.00	21.40	13.20	32.20	7.00	7.8
330.0	343	69.80	45.70	24.10	11.30	12.10	15.00	10.9
317.0	344	75.00	42.50	32.50	17.90	26.80	13.00	5.8
327.0	431	72.20	40.70	31.50	5.00	12.50	11.00	16.9
324.0	223	78.00	47.40	30.60	8.10	17.00	13.00	11.8
345.0	272	68.90	42.90	26.00	13.60	21.50	12.00	10.8
345.0	268	69.10	45.20	23.90	7.70	16.30	12.00	8.4
358.0	330	83.90	48.50	35.40	10.30	23.90	14.00	11.5
334.0	185	68.80	44.40	24.40	9.50	20.40	14.00	13.8
326.0	222	69.00	42.00	27.00	10.70	15.50	11.00	23.9
349.0	368	#NULL!	#NULL!	#NULL!	17.00	23.00	#NULL!	15.1
325.0	377	64.00	39.50	24.50	13.00	16.40	9.00	20.9
288.0	853	73.60	35.60	38.00	6.70	10.90	17.00	5.7
332.0	319	65.80	41.40	24.40	11.80	21.90	13.00	7.5
347.0	349	63.80	40.90	22.90	10.20	22.30	10.00	7.5
317.0	551	61.00	19.70	41.30	2.60	8.40	11.00	4.7
338.0	312	63.70	42.50	21.20	7.60	17.10	10.00	16.0
341.0	305	67.20	43.50	23.70	8.50	19.10	7.00	8.2
#NULL!	#NULL!	68.20	43.10	25.10	9.70	24.50	10.00	4.9
328.0	330	72.20	44.40	27.80	17.80	18.70	19.00	6.1
337.0	266	75.10	44.90	30.20	19.10	19.30	14.00	7.4
352.0	316	67.80	44.00	23.80	12.20	21.80	8.00	10.8
314.0	307	66.10	41.90	24.20	19.80	23.30	14.00	6.5
#NULL!	343	#NULL!	#NULL!	28.50	#NULL!	#NULL!	#NULL!	#NULL!
337.0	346	82.80	50.40	32.40	13.10	17.80	#NULL!	19.9
323.0	465	76.30	44.30	32.00	13.80	17.00	17.00	5.6
357.0	403	67.10	43.30	23.80	19.60	25.90	11.50	9.9
324.0	284	72.60	42.10	30.50	8.80	16.20	8.00	4.6
325.0	252	71.10	43.50	27.60	10.20	12.20	9.00	9.0
344.0	190	61.40	38.60	22.80	6.20	31.40	10.00	6.9
321.0	316	80.80	45.10	35.70	15.00	18.90	22.00	9.5
340.0	492	75.00	44.50	30.50	113.60	57.40	63.00	10.3
326.0	370	70.60	44.10	26.50	14.70	13.70	14.00	8.7
324.0	411	75.00	47.60	27.40	154.60	80.10	100.00	17.8
341.0	323	78.10	49.50	28.60	11.00	17.20	10.00	16.3
327.0	215	70.20	45.30	24.90	13.60	16.30	11.00	7.2
338.0	377	68.50	40.90	27.60	9.10	13.10	16.00	6.0
349.0	367	73.30	44.90	28.40	13.50	19.90	13.00	6.8
340.0	300	66.90	38.10	28.80	12.80	19.60	10.00	3.4

344.0	276	82.50	48.90	33.60	13.70	21.80	17.00	6.6
#NULL!	#NULL!	#NULL!	#NULL!	27.70	#NULL!	#NULL!	13.00	#NULL!
355.0	283	76.50	47.40	29.10	13.80	28.80	5.00	7.8
328.0	542	59.10	35.10	24.00	21.00	43.00	#NULL!	6.3
334.0	266	67.90	43.80	24.10	11.70	15.10	11.00	4.6
353.0	223	70.00	46.20	23.80	15.80	19.60	23.80	29.5
342.0	291	76.00	46.20	29.80	5.70	20.60	10.00	6.3
339.0	279	83.80	53.00	30.80	53.00	27.00	20.00	7.7
347.0	330	61.20	40.10	21.10	10.80	26.40	9.00	11.9
331.0	151	69.70	45.10	24.60	24.50	16.40	24.00	6.9
338.0	280	73.80	44.90	28.90	10.40	19.60	12.00	5.2
353.0	300	70.70	43.50	27.20	11.90	20.60	17.00	4.6
339.0	263	66.80	41.90	24.90	15.30	25.70	13.00	5.6
340.0	176	69.40	44.30	25.10	15.10	20.60	12.00	4.7
328.0	367	77.50	48.30	29.20	15.80	25.50	31.00	7.9
333.0	263	62.20	42.10	20.10	10.70	17.50	9.00	3.1
348.0	272	75.70	43.60	32.10	22.00	18.00	14.00	10.7
337.0	285	75.40	41.90	33.50	10.50	15.80	17.00	4.7
332.0	249	55.30	34.00	21.30	7.60	20.00	11.00	5.5
344.0	182	77.00	47.40	29.60	16.50	29.60	5.00	6.0
332.0	231	69.00	46.80	22.20	13.10	18.90	17.00	11.3
316.0	321	69.40	43.40	26.00	29.00	19.60	42.00	11.1
354.0	264	70.80	46.30	24.50	11.10	21.10	12.00	7.3
#NULL!	236	60.70	37.30	23.40	17.40	35.50	12.90	3.4
324.0	191	72.20	42.20	30.00	17.50	19.80	14.00	5.7
315.0	236	78.90	44.40	34.50	17.50	17.00	16.00	11.0
#NULL!	#NULL!	75.90	47.50	28.40	11.00	21.20	13.00	3.9
337.0	234	68.20	41.00	27.20	7.10	22.70	10.00	8.1
322.0	357	77.90	45.60	32.30	13.20	19.60	12.00	6.0
361.0	343	79.70	48.90	30.80	19.50	26.60	11.00	13.0
333.0	259	69.40	43.30	26.10	14.70	22.30	10.00	6.9
338.0	182	73.90	49.70	24.20	10.90	21.30	11.00	12.0
350.0	390	75.50	48.90	26.60	11.00	24.90	16.00	14.5
333.0	390	75.30	43.60	31.70	10.50	17.60	10.80	3.6
359.0	271	69.30	44.50	24.80	10.00	20.70	15.00	11.5
331.0	357	68.80	39.70	29.10	9.70	22.40	12.00	5.9
353.0	344	62.80	35.20	27.60	17.70	23.20	11.00	10.2
337.0	316	70.70	39.30	31.40	11.60	23.90	12.00	3.3
354.0	228	64.70	42.00	22.70	10.70	25.00	12.00	11.4
321.0	268	69.70	41.80	27.90	8.40	15.30	11.00	4.2
324.0	248	75.70	40.10	35.60	18.00	22.00	#NULL!	14.2
320.0	358	68.30	40.60	27.70	7.90	21.50	12.00	10.8
339.0	194	70.20	42.50	27.70	11.70	18.70	13.00	5.2
351.0	278	79.90	45.80	34.10	15.00	17.10	16.00	8.1
338.0	327	70.80	43.50	27.30	29.80	20.10	25.00	5.9
330.0	375	76.80	47.00	29.80	13.90	23.70	11.00	19.3
333.0	310	68.60	42.30	26.30	20.10	30.00	11.00	2.2
331.0	220	67.90	40.90	27.00	9.60	16.20	8.00	6.2
335.0	232	70.60	39.20	31.40	8.80	26.00	9.00	2.7
338.0	271	70.90	45.00	25.90	11.10	26.20	11.00	4.1
363.0	288	63.80	42.70	21.10	10.10	17.20	15.00	4.6
335.0	435	67.70	41.20	26.50	7.40	24.80	9.00	12.9
345.0	224	60.90	37.00	23.90	8.40	18.30	12.00	4.7
339.0	404	76.20	43.50	32.70	14.20	14.90	15.00	7.1

341.0	480	34.90	4.60	30.30	13.00	24.00	35.00	6.0
332.0	384	74.60	43.10	31.50	9.20	24.50	11.00	6.6
326.0	424	69.60	46.30	23.30	10.10	27.00	7.00	5.1
363.0	335	65.90	40.20	25.70	9.00	15.90	10.00	17.7
323.0	271	71.80	47.60	24.20	11.60	21.90	19.00	6.2
329.0	324	65.70	44.00	21.70	10.70	26.10	10.00	4.1
346.0	342	72.40	42.60	29.80	23.10	19.70	26.00	4.1
328.0	260	72.20	47.60	24.60	5.90	21.90	13.00	29.7
324.0	280	69.30	45.10	24.80	10.30	16.40	13.00	14.2
318.0	333	74.10	43.30	30.80	5.80	11.40	9.70	13.7
335.0	301	73.20	45.50	27.70	15.10	26.30	12.00	10.2
338.0	155	74.70	50.70	24.00	10.40	19.00	9.00	8.8
329.0	318	71.80	46.50	25.30	15.40	30.50	10.00	16.6
337.0	308	82.30	46.20	36.10	12.10	18.70	7.00	4.6
333.0	306	76.00	45.60	30.40	14.40	29.10	15.00	2.5
326.0	199	68.10	46.30	21.80	19.30	36.00	11.10	5.3
322.0	336	68.60	44.10	24.50	15.20	13.40	30.00	5.3
348.0	310	68.70	43.20	25.50	9.30	17.40	18.00	5.2
336.0	266	72.70	48.10	24.60	11.00	17.40	15.00	3.0
353.0	318	71.70	42.70	29.00	6.30	17.90	15.00	5.9
318.0	311	65.40	40.10	25.30	11.40	24.10	9.00	3.2
352.0	322	47.10	29.10	18.00	7.20	15.70	11.00	12.5
339.0	263	62.10	39.00	23.10	224.30	141.00	296.00	128.7
320.0	188	66.00	45.70	20.30	10.90	20.00	10.00	6.5
#NULL!	211	76.30	47.50	28.80	22.40	23.70	17.00	8.3
328.0	424	71.40	46.20	25.20	21.50	26.00	18.00	6.0
318.0	241	76.00	40.80	35.20	14.90	11.20	26.00	7.4
336.0	186	66.20	44.40	21.80	6.50	14.70	11.00	25.4
336.0	274	65.00	38.80	26.20	8.10	22.00	9.00	3.4
338.0	344	75.30	44.90	30.40	6.30	22.30	14.00	10.6
337.0	226	70.70	47.20	23.50	17.20	29.20	9.00	5.4
318.0	246	77.30	48.40	28.90	11.10	23.00	15.00	7.8
332.0	302	69.20	44.90	24.30	11.20	25.10	13.30	19.0
329.0	295	69.00	40.90	28.10	8.90	17.60	12.30	8.7
336.0	433	76.20	46.50	29.70	653.00	1253.70	445.00	38.8
324.0	352	67.30	42.80	24.50	13.50	30.70	8.00	5.9
334.0	326	77.50	47.60	29.90	9.60	25.70	14.00	11.9
338.0	327	75.10	43.80	31.30	7.70	20.10	12.00	6.1
326.0	321	69.30	44.70	24.60	7.40	23.00	12.00	9.1
332.0	352	68.90	39.70	28.90	9.10	21.60	12.00	8.3
329.0	10	83.40	43.00	40.40	11.50	16.50	17.00	6.7
305.0	345	60.30	35.10	25.20	7.20	11.20	8.00	4.6
350.0	284	73.80	44.30	29.50	10.60	24.50	15.00	15.1
327.0	455	76.40	42.20	34.20	9.00	26.00	#NULL!	10.6
347.0	346	69.20	47.40	21.80	9.80	19.90	15.00	7.7
321.0	292	69.50	44.70	24.80	11.40	20.30	14.00	3.2
340.0	244	63.20	44.00	19.20	8.90	15.70	12.00	8.9
341.0	404	71.60	43.30	28.30	10.00	28.40	9.00	7.2
317.0	305	63.00	42.30	20.70	10.90	18.30	11.00	7.8
321.0	609	64.40	33.40	31.00	17.10	11.90	63.00	16.1
330.0	371	76.20	44.70	31.50	4.20	22.00	15.00	12.0
319.0	227	70.10	41.40	28.70	9.70	20.00	13.60	13.5
329.0	383	67.90	44.10	23.80	12.40	19.60	13.00	5.0
345.0	272	68.90	45.70	23.20	9.00	21.20	13.00	21.1

338.0	258	71.50	49.50	22.00	11.40	22.90	14.00	25.5
357.0	278	66.00	43.60	22.40	12.80	18.00	11.00	24.3
309.0	323	65.40	43.80	21.60	8.00	20.60	12.00	14.1
329.0	393	67.20	41.70	25.50	11.50	25.40	12.00	5.4
325.0	290	72.90	45.50	27.40	7.80	16.90	12.00	8.6
342.0	327	64.80	42.40	22.40	9.80	21.70	9.00	7.9
343.0	223	61.10	41.50	19.60	9.20	19.40	15.00	9.9
327.0	227	65.20	40.20	25.00	10.00	17.60	16.00	2.4
325.0	352	65.20	40.50	24.70	6.90	19.00	10.00	9.4
315.0	305	70.70	42.60	28.10	7.30	25.60	17.00	9.0
#NULL!	350	72.50	40.40	32.10	34.70	32.90	17.10	5.3
334.0	239	66.00	40.00	26.00	3.50	21.30	10.00	7.8
319.0	268	68.70	40.30	28.40	16.40	15.50	21.00	14.7
337.0	236	73.50	45.30	28.20	6.20	11.70	11.00	16.7
326.0	270	68.10	43.20	24.90	25.00	28.20	22.00	10.1
341.0	410	70.30	46.00	24.30	9.40	16.60	14.00	22.6
320.0	330	68.90	39.90	29.00	9.30	16.30	10.00	9.5
324.0	261	66.60	43.30	23.30	12.30	18.00	13.00	4.0
326.0	489	58.90	38.10	20.80	9.50	19.30	12.00	7.3
343.0	392	64.50	45.40	19.10	17.10	22.90	23.00	5.5
313.0	320	77.70	48.20	29.50	11.30	18.80	39.00	7.7
339.0	347	68.60	43.60	25.00	10.70	19.00	12.00	10.5
308.0	296	69.70	41.80	27.90	12.20	19.30	11.00	3.3
351.0	253	72.70	45.60	27.10	10.50	15.50	15.00	6.4
325.0	191	66.40	42.70	23.70	10.40	17.40	13.00	10.7
323.0	355	64.40	40.10	24.30	12.30	21.80	16.00	9.0
316.0	289	72.20	44.70	27.50	15.80	31.00	14.00	4.0
340.0	302	68.80	45.10	23.70	14.70	22.30	11.00	9.1
333.0	258	67.20	43.30	23.90	14.40	25.40	9.00	4.8
336.0	271	69.90	43.50	26.40	15.00	17.10	16.00	21.6
321.0	413	63.70	39.90	23.80	9.40	23.40	11.00	5.2
308.0	297	58.20	37.50	20.70	20.00	16.30	19.00	2.5
308.0	109	67.70	40.10	27.60	12.40	15.00	8.00	7.9
329.0	466	74.30	46.50	27.80	30.30	46.20	18.00	4.1
328.0	401	75.60	46.20	39.40	11.50	15.90	14.00	13.0
319.0	284	62.70	42.50	20.20	12.60	28.40	12.00	8.7
312.0	321	76.30	43.80	32.50	9.80	17.90	15.00	5.0
315.0	263	67.90	44.90	23.00	20.40	35.50	13.00	3.3
329.0	359	67.30	40.90	26.40	10.40	21.00	17.00	7.9
320.0	442	65.20	40.20	25.00	18.10	32.60	6.00	5.6
316.0	285	72.20	42.40	29.80	12.10	21.00	14.00	2.5
322.0	248	61.30	39.90	21.40	17.50	29.00	9.00	11.0
#NULL!	#NULL!	69.10	40.20	28.90	18.00	24.50	14.00	6.1
328.0	327	73.90	41.30	32.60	9.40	21.20	12.00	1.8
#NULL!	#NULL!	66.40	42.80	23.60	12.10	24.80	12.00	4.5
332.0	320	64.50	43.60	20.90	15.90	29.70	9.00	6.7
345.0	296	63.80	40.60	23.20	19.50	23.90	12.00	7.1
339.0	239	70.00	42.60	27.40	10.70	20.00	12.00	9.2
340.0	276	69.00	43.00	26.00	10.90	20.60	13.00	7.4
334.0	322	70.30	43.20	27.10	12.00	19.60	22.00	4.1
324.0	244	71.40	42.60	28.80	9.90	53.90	13.00	7.1
328.0	340	72.50	42.60	29.90	11.50	16.30	43.00	7.7
352.0	258	70.50	46.90	23.60	36.50	28.00	21.00	13.0
329.0	254	61.60	43.40	18.20	7.30	13.80	12.00	3.7

327.0	350	68.50	40.20	28.30	17.60	13.70	12.00	4.2
331.0	219	63.50	41.00	22.50	11.60	22.70	10.00	7.2
336.0	237	70.30	47.40	22.90	15.00	26.00	12.00	6.5
338.0	166	64.10	39.20	24.90	14.40	27.10	8.00	7.0
333.0	320	71.40	42.70	28.70	11.10	16.80	16.00	4.8
338.0	278	74.10	46.30	27.80	14.00	22.60	20.00	5.9
325.0	401	69.80	44.10	25.70	14.90	15.50	16.00	7.7
323.0	300	75.50	49.70	25.80	13.80	20.60	11.00	5.4
323.0	228	68.80	42.40	26.40	11.50	16.50	8.00	19.1
#NULL!	297	79.20	51.50	27.70	22.50	18.70	19.00	11.3
328.0	403	78.90	44.60	34.30	24.60	23.80	16.00	7.8
338.0	331	74.90	44.50	30.40	15.20	26.60	17.00	6.5
327.0	321	74.60	47.90	26.70	7.80	15.00	10.00	7.7
334.0	295	71.10	44.50	26.60	13.60	18.10	17.00	6.0
335.0	317	67.80	42.00	25.80	10.00	16.90	11.00	14.7
355.0	206	69.50	43.20	26.40	11.00	17.30	13.00	5.3
#NULL!	453	66.90	41.80	25.10	12.60	11.80	16.00	9.0
328.0	273	73.60	44.80	28.80	8.40	16.30	15.00	8.2
334.0	297	86.30	45.70	40.60	10.60	18.00	14.00	5.4
339.0	290	73.00	44.70	28.30	19.70	28.90	11.20	2.4
346.0	401	76.60	48.60	28.00	12.90	18.30	23.00	9.3
322.0	238	66.20	42.80	23.40	10.80	22.60	14.00	6.0
323.0	282	78.60	41.60	37.00	15.40	20.50	17.00	4.1
332.0	532	50.90	31.20	19.70	6.60	15.30	9.00	5.7
332.0	385	70.40	44.20	26.20	8.00	23.00	12.00	6.7
323.0	432	65.00	38.30	26.70	16.00	21.00	11.00	2.9
334.0	334	75.50	45.10	30.40	11.70	23.60	11.00	14.2
322.0	455	62.20	41.00	21.20	32.40	44.70	9.70	3.6
328.0	276	70.80	45.10	25.70	15.10	21.60	12.00	8.7
315.0	285	73.60	41.60	32.00	20.00	16.00	#NULL!	7.9
326.0	292	72.50	44.90	27.60	44.30	31.20	14.00	11.8
338.0	196	70.10	44.10	26.00	8.30	20.80	10.00	7.0
311.0	257	76.00	45.50	30.50	6.90	15.50	12.20	14.0
337.0	245	74.40	44.80	29.60	10.40	23.60	11.00	4.5
333.0	371	71.10	41.00	30.10	13.20	15.50	9.00	4.8
340.0	322	72.70	46.10	26.60	13.30	16.10	22.00	13.7
318.0	383	69.80	45.70	24.10	6.00	15.40	9.00	11.8
347.0	292	67.80	35.40	32.40	5.60	14.90	#NULL!	9.2
325.0	380	75.70	47.40	28.30	9.00	24.90	10.00	10.9
313.0	286	76.90	47.30	29.60	63.80	40.50	43.70	6.0
338.0	291	72.60	42.60	30.00	5.60	15.40	10.00	10.7
334.0	301	74.40	40.90	33.50	21.00	15.00	#NULL!	7.8
334.0	206	65.20	39.80	25.40	17.10	21.70	14.00	3.9
338.0	240	67.80	43.00	24.80	8.10	9.70	9.00	7.0
343.0	265	71.30	45.80	25.50	11.00	15.30	13.00	13.3
331.0	403	73.70	43.40	30.30	40.50	31.00	26.00	6.2
332.0	350	73.50	43.90	29.60	7.60	17.20	9.00	5.5
330.0	246	66.60	40.60	26.00	4.40	15.00	9.00	6.2
329.0	274	73.00	45.00	28.00	98.50	41.10	60.00	8.6
342.0	362	69.90	46.10	23.80	11.20	17.10	12.00	8.2
336.0	328	68.10	41.20	26.90	7.00	13.20	7.70	9.9
333.0	330	73.20	47.40	25.80	12.00	17.00	16.00	8.9
333.0	338	76.20	47.20	29.00	16.20	29.80	14.00	4.3
346.0	294	66.70	43.90	22.80	11.20	17.70	12.00	24.2

333.0	267	62.60	41.10	21.50	12.00	20.60	9.00	17.0
339.0	269	74.50	47.60	26.90	9.40	21.80	10.00	7.1
335.0	308	68.90	44.90	24.00	26.20	23.30	23.00	9.7
321.0	276	64.20	40.20	24.00	17.70	26.10	10.00	3.4
334.0	406	74.40	45.10	29.30	16.70	18.30	11.00	5.0
336.0	310	77.40	47.70	29.70	11.10	13.10	11.00	12.0
339.0	234	69.90	43.90	26.00	11.20	15.60	8.00	5.4
339.0	166	77.30	49.40	27.90	12.20	20.80	11.00	10.6
352.0	268	75.60	48.60	27.00	14.10	17.80	14.00	13.4
336.0	233	79.20	51.10	28.10	72.80	58.40	78.00	8.4
324.0	225	73.60	43.40	30.20	24.80	26.90	19.00	7.8
330.0	358	67.50	44.10	23.40	15.20	20.50	12.00	15.3
341.0	255	80.00	48.20	31.80	26.00	30.90	17.00	5.3
332.0	279	85.90	51.00	34.90	11.80	24.90	9.00	5.0
328.0	316	66.30	40.60	25.70	10.90	18.90	11.00	18.0
329.0	298	71.10	42.50	28.60	6.30	13.10	12.00	8.0
339.0	261	63.10	35.60	27.50	26.30	20.20	10.00	8.8
329.0	247	72.20	47.00	25.20	12.50	17.10	13.00	6.7
333.0	472	72.40	43.70	28.70	11.20	19.90	22.00	9.5
329.0	461	80.50	44.90	35.60	8.20	20.80	8.00	11.1
333.0	292	72.20	44.30	27.90	16.00	24.80	16.00	5.3
335.0	220	69.50	44.90	24.60	14.70	24.40	8.00	8.9
335.0	375	67.30	42.70	24.60	17.40	24.40	12.00	2.4
338.0	160	38.40	15.80	22.60	18.70	14.00	28.00	3.0
333.0	350	58.50	39.10	19.40	7.77	17.02	10.00	2.3
334.0	108	57.50	37.20	20.30	37.40	47.70	56.00	5.3
335.0	328	66.50	42.90	23.60	17.10	21.00	12.00	7.9
330.0	324	68.00	41.00	27.00	9.90	20.60	9.00	6.6
#NULL!	325	63.70	43.40	20.30	14.30	20.50	10.00	7.9
337.0	286	69.70	42.50	27.20	10.10	13.60	16.00	9.8
328.0	307	72.20	41.90	30.30	17.10	14.20	29.00	4.6
341.0	473	51.70	27.50	24.20	10.40	29.70	25.00	12.5
345.0	324	70.10	44.30	25.80	16.60	20.90	11.00	4.3
325.0	363	80.30	49.20	31.10	12.90	19.50	12.00	8.1
357.0	332	69.40	45.40	24.00	8.00	21.30	11.00	14.2
340.0	27	67.00	40.20	26.80	9.90	16.40	19.00	5.3
341.0	339	78.40	49.50	28.90	59.31	30.91	#NULL!	4.0
360.0	433	72.90	43.60	29.30	13.91	21.61	#NULL!	8.1
340.0	340	68.80	43.80	25.00	13.90	24.20	10.00	5.5
337.0	297	69.90	43.20	26.70	14.80	17.10	10.00	9.8
354.0	316	67.50	43.40	24.10	8.80	21.80	10.00	8.4
337.0	206	63.40	39.10	24.30	21.20	27.90	17.00	15.6
343.0	181	66.90	45.30	21.60	10.10	22.50	10.00	10.6
333.0	181	70.70	47.40	23.30	24.10	30.30	11.00	4.2
329.0	333	76.90	48.20	28.70	7.40	19.10	11.00	7.5
352.0	385	78.10	53.20	24.90	18.11	28.30	#NULL!	13.6
333.0	268	63.30	39.50	23.80	5.60	12.30	7.00	8.0
328.0	226	68.10	44.30	23.80	12.80	20.70	8.00	9.9
341.0	338	67.40	45.00	22.40	8.10	24.00	12.00	5.3
340.0	224	67.60	44.40	23.20	18.30	20.80	16.00	8.2
340.0	280	67.70	43.20	24.50	8.20	21.50	10.00	8.8
341.0	246	62.90	45.10	17.80	8.50	16.90	14.00	7.1
335.0	280	73.10	47.00	26.10	18.30	17.60	17.00	6.7
337.0	327	62.70	39.40	23.30	8.80	16.39	#NULL!	6.1

334.0	395	73.10	49.70	23.40	34.60	27.40	29.00	5.2
337.0	287	67.80	46.10	21.70	23.10	23.80	19.00	7.0
336.0	124	74.50	43.30	31.20	5.50	10.10	7.00	15.4
343.0	304	60.00	43.50	16.50	8.00	26.90	10.00	8.4
342.0	234	67.60	45.20	22.40	23.40	32.90	17.00	3.3
337.0	235	69.70	47.30	22.40	10.20	23.40	7.00	4.3
334.0	435	77.00	51.90	25.10	8.60	11.50	13.00	6.6
334.0	323	74.70	45.70	29.00	9.10	17.40	11.00	2.9
341.0	242	77.60	48.80	28.80	9.70	20.30	9.00	12.2
341.0	194	65.10	45.70	19.40	13.50	25.50	9.00	5.1
340.0	166	61.80	43.70	18.10	9.40	20.50	14.00	9.4
332.0	243	69.30	44.00	25.30	9.70	21.90	8.00	1.5
340.0	196	73.00	45.20	27.80	16.80	20.60	19.00	4.2
347.0	249	70.10	43.20	26.90	18.30	15.90	15.00	10.0
351.0	170	66.20	43.20	23.00	16.00	26.50	13.00	4.8
#NULL!	240	70.00	48.30	21.70	14.50	23.40	15.00	7.4
345.0	284	70.00	46.50	23.50	17.00	20.80	12.00	7.4
339.0	233	67.40	45.00	22.40	8.80	17.50	9.00	5.7
342.0	258	64.80	40.50	24.30	11.90	15.20	12.00	5.5
337.0	305	73.60	47.20	26.40	36.90	25.40	24.00	13.2
336.0	197	71.30	46.20	25.10	17.90	17.30	18.00	10.7
338.0	278	66.20	41.70	24.50	12.30	29.60	11.00	2.0
334.0	288	73.30	46.40	26.90	9.28	23.76	#NULL!	8.9
342.0	230	63.10	42.10	21.00	11.20	24.70	11.00	7.9
339.0	211	70.00	47.50	22.50	16.70	26.80	11.00	10.8
338.0	325	72.00	44.30	27.70	20.10	43.30	11.00	1.6
328.0	524	49.00	25.70	23.30	7.20	14.80	9.00	1.0
340.0	196	63.60	41.40	22.20	13.60	17.20	8.00	3.9
342.0	259	66.90	40.40	26.50	12.00	22.80	8.00	9.9
343.0	362	64.30	43.50	20.80	14.40	17.60	12.00	6.8
341.0	266	69.00	46.80	22.20	19.30	18.80	19.00	2.8
345.0	285	64.60	40.90	23.70	67.50	41.90	43.30	4.2
339.0	419	66.50	45.80	20.70	15.38	26.08	#NULL!	12.8
341.0	203	70.00	48.40	21.60	11.10	23.60	12.00	5.2
325.0	447	72.20	45.60	26.60	20.30	18.00	19.00	4.0
346.0	308	69.40	47.10	22.30	21.40	33.10	10.00	3.8
339.0	407	67.00	42.20	24.80	24.34	36.94	#NULL!	5.3
336.0	237	63.90	42.30	21.60	10.50	14.50	13.00	12.0
335.0	342	62.20	42.90	19.30	19.20	20.30	12.00	3.4
330.0	353	57.00	36.80	20.20	77.10	45.20	90.30	13.1
343.0	277	73.60	42.90	30.70	5.00	13.10	9.00	6.2
342.0	237	75.00	48.20	26.80	11.10	22.90	11.00	5.1
345.0	311	73.60	46.30	27.30	7.60	23.40	14.00	6.0
340.0	300	79.30	49.00	30.30	15.76	27.35	#NULL!	4.5
330.0	248	65.50	41.70	23.90	12.33	25.96	#NULL!	6.2
339.0	165	71.40	43.90	27.50	4.20	18.30	5.00	14.7
344.0	248	74.20	44.40	29.80	14.80	18.70	22.00	11.0
334.0	220	73.20	43.40	29.80	7.10	16.30	11.00	10.7
337.0	424	81.90	53.20	28.70	21.10	16.90	52.00	9.7
349.0	301	67.50	39.50	28.00	18.00	27.70	15.00	8.8
339.0	209	72.70	46.00	26.70	9.30	15.90	13.00	11.7
347.0	320	73.00	45.40	27.60	9.20	14.00	14.00	9.7
341.0	278	74.90	44.00	30.90	10.20	20.90	13.00	3.8
291.0	149	38.60	25.10	13.50	14.10	13.20	11.00	34.7

341.0	323	70.40	41.30	29.10	6.60	30.30	13.00	4.5
351.0	315	65.10	40.00	25.10	8.50	16.50	12.00	7.5
344.0	339	68.70	41.60	27.10	20.10	25.00	15.00	15.4
340.0	250	66.60	40.90	25.70	10.10	20.70	13.00	2.8
341.0	309	77.50	47.90	29.60	9.20	17.30	10.00	8.6
336.0	164	74.20	43.60	30.60	7.10	27.70	10.00	8.6
328.0	305	72.10	43.60	28.50	20.30	26.80	16.00	8.6
342.0	211	69.70	46.10	23.60	8.70	22.30	11.00	7.2
345.0	306	65.90	40.40	25.50	9.60	15.80	14.00	9.8
343.0	296	83.20	47.80	35.40	15.63	29.82	13.00	11.8
339.0	411	73.20	42.50	30.70	21.40	19.20	25.00	6.0
336.0	360	60.80	39.10	21.70	15.10	25.10	12.00	6.5
336.0	183	65.20	40.40	24.80	10.40	16.20	13.00	13.7
323.0	278	68.50	42.80	25.70	11.50	40.30	13.00	8.6
342.0	371	81.80	45.80	36.00	10.10	17.60	11.00	9.3
327.0	267	69.80	40.50	29.30	13.20	18.10	12.00	4.4
335.0	255	64.10	39.60	24.50	7.00	16.60	11.00	3.5
340.0	290	81.20	45.70	35.50	21.60	31.10	16.00	8.1
344.0	283	63.70	43.20	20.50	13.00	31.20	9.00	10.2
325.0	304	73.30	44.20	29.10	23.20	14.30	36.00	7.9
339.0	188	76.90	46.50	30.40	27.10	21.20	23.00	19.1
337.0	384	77.90	46.90	31.00	15.30	21.80	17.00	5.6
342.0	195	65.70	42.50	23.20	9.20	17.90	14.00	6.9
339.0	233	76.40	46.90	29.50	11.20	22.90	15.00	3.9
336.0	325	68.30	39.10	29.20	129.80	108.70	268.00	14.0
334.0	243	74.00	47.00	27.00	10.70	18.20	14.00	16.0
336.0	323	73.70	43.40	30.30	17.00	17.40	43.00	6.2
332.0	312	64.50	42.30	22.20	12.10	27.60	12.00	3.6
337.0	623	94.00	49.30	44.70	20.10	32.50	20.00	10.6
329.0	287	63.00	41.60	21.40	8.30	15.00	9.00	5.4
333.0	401	62.60	40.10	22.50	29.40	20.50	16.00	3.3
337.0	385	83.10	51.20	31.90	7.80	13.30	19.00	8.4
329.0	316	71.10	44.10	27.00	7.00	18.00	15.40	5.6
341.0	405	72.80	44.60	28.20	9.10	19.20	10.00	10.1
324.0	272	70.10	44.40	25.70	8.00	22.20	15.00	4.2
330.0	273	77.30	48.50	28.80	8.90	17.90	9.00	8.6
335.0	309	69.00	41.70	27.30	15.60	21.70	18.00	10.5
340.0	258	76.00	45.50	30.50	27.80	24.20	33.00	13.6
340.0	241	68.50	43.50	25.00	11.47	24.11	#NULL!	15.8
336.0	335	69.30	45.00	24.30	9.40	18.30	13.00	12.9
338.0	138	69.20	42.40	26.80	11.50	21.20	23.00	9.1
343.0	316	71.50	38.60	32.90	8.20	18.40	14.00	3.9
#NULL!	#NULL!	66.50	43.50	23.00	10.60	27.10	12.00	9.1
336.0	194	66.80	42.80	24.00	6.40	12.40	10.00	10.3
337.0	276	65.50	44.00	21.50	8.90	23.50	12.00	8.7
333.0	320	71.40	42.50	28.90	9.50	16.60	17.00	9.9
337.0	357	68.70	43.80	24.90	20.50	23.40	20.00	9.7
334.0	288	74.00	45.10	28.90	7.80	11.80	23.00	11.0
317.0	320	69.40	42.30	27.10	18.10	15.90	21.00	7.2
340.0	391	72.70	45.50	27.20	14.80	33.30	15.00	21.6
322.0	231	67.30	43.80	23.50	91.90	71.80	16.00	3.0
333.0	258	68.00	44.60	23.40	10.30	20.40	15.00	22.1
330.0	310	73.00	46.20	26.80	11.50	22.10	14.00	4.7
330.0	300	67.50	41.80	25.70	17.40	20.40	21.00	4.9

342.0	288	71.90	43.40	28.50	12.10	18.30	11.00	5.1
348.0	522	61.10	38.90	22.20	16.20	25.30	10.00	2.5
346.0	367	71.60	44.40	27.20	18.80	21.50	16.00	4.0
325.0	386	73.60	41.70	31.90	5.80	20.20	15.00	22.1
349.0	177	71.10	41.20	29.90	16.00	20.00	11.00	11.7
329.0	413	76.90	45.00	31.90	16.60	23.20	14.00	5.3
328.0	279	56.20	36.40	19.80	15.60	22.20	10.00	6.6
336.0	391	79.00	42.30	36.70	11.00	20.30	8.00	6.6
340.0	283	63.70	41.40	22.30	8.20	13.10	21.00	5.3
338.0	306	72.30	42.20	30.10	13.00	27.40	14.00	5.7
333.0	282	67.40	41.20	26.20	32.70	30.40	14.00	5.4
338.0	261	79.70	46.40	33.30	11.70	17.30	13.00	12.3
334.0	344	71.00	44.10	26.90	9.90	15.30	13.00	5.2
319.0	189	64.00	38.50	25.50	9.00	17.20	11.00	7.6
351.0	8	69.20	41.50	27.70	11.10	13.50	13.00	14.7
335.0	244	72.80	45.20	27.60	26.40	19.80	27.00	40.7
331.0	230	64.00	40.40	23.60	14.80	18.10	8.00	4.1
#NULL!	#NULL!	67.90	44.00	23.90	6.20	17.50	14.00	6.2
338.0	300	71.20	42.40	28.80	7.70	14.70	13.00	19.5
343.0	242	72.70	46.00	26.70	6.20	17.40	26.00	23.2
339.0	297	65.20	42.70	22.50	10.70	22.10	11.00	7.4
347.0	280	70.90	46.00	24.90	20.70	17.60	17.50	12.5
342.0	390	71.40	44.90	26.50	9.90	23.20	12.00	7.8
343.0	251	70.80	47.20	23.60	9.80	17.60	15.00	19.7
332.0	205	68.10	40.90	27.20	12.10	19.20	18.00	6.4
331.0	325	80.40	49.00	31.40	23.37	33.54	12.00	7.8
339.0	265	69.80	43.00	26.80	11.10	19.50	12.00	12.4
338.0	433	69.70	46.60	23.10	23.20	23.30	11.00	6.8
335.0	349	62.60	40.40	22.20	16.00	30.60	14.00	5.8
326.0	272	70.50	43.90	26.60	13.70	23.40	14.00	4.3
328.0	337	76.10	42.00	34.10	11.30	18.60	29.00	6.3
330.0	322	67.70	44.90	22.80	11.40	18.90	13.00	9.1
340.0	329	69.70	40.10	29.60	11.80	14.60	17.00	3.5
337.0	414	62.30	39.00	23.30	8.00	16.30	9.30	8.6
330.0	292	66.30	40.60	25.70	11.30	17.10	19.00	6.8
336.0	320	68.80	44.60	24.20	18.80	20.40	15.00	6.7
332.0	297	73.60	45.30	28.30	18.80	27.50	17.00	11.2
340.0	298	74.40	46.70	27.70	10.50	21.70	14.60	7.0
335.0	264	72.10	43.80	28.30	7.30	22.50	10.00	11.0
338.0	473	73.60	47.50	26.10	13.70	29.60	16.00	4.9
330.0	485	63.50	38.70	24.80	6.50	18.00	12.00	4.3
323.0	348	64.90	42.80	22.10	11.90	21.10	12.00	5.1
339.0	386	70.80	42.30	28.50	15.00	32.70	9.50	8.0
299.0	36	68.70	39.20	29.50	44.50	45.70	60.00	12.5
329.0	326	66.70	41.20	25.50	8.20	15.90	25.00	5.4
341.0	323	77.70	49.60	28.10	15.90	15.50	25.00	9.6
348.0	268	68.30	41.80	26.50	7.20	13.80	9.00	4.3
342.0	328	70.30	43.20	27.10	12.70	20.00	17.00	13.6
332.0	451	72.40	46.10	26.30	10.10	21.70	16.00	7.5
339.0	301	67.90	45.80	22.10	12.10	18.20	11.70	18.0
337.0	244	74.80	44.60	30.20	7.60	11.90	12.00	5.7
330.0	325	74.90	44.70	30.20	15.10	16.10	17.00	5.9
336.0	278	63.80	41.10	22.70	10.90	20.10	16.00	8.5
329.0	405	70.40	45.00	25.40	12.60	18.60	17.00	5.0

331.0	452	62.00	39.70	22.30	22.90	35.10	12.00	3.0
343.0	215	73.80	46.80	27.00	7.50	25.70	10.00	12.4
339.0	403	68.00	42.10	25.90	11.10	19.10	10.00	6.3
333.0	297	71.70	43.90	27.80	12.10	19.50	11.00	2.8
335.0	251	68.40	41.70	26.70	10.70	21.70	10.00	7.7
338.0	287	80.60	47.20	33.40	103.80	54.60	48.00	7.7
350.0	303	71.50	44.20	27.30	10.60	22.10	13.00	12.3
338.0	252	69.90	44.10	25.80	18.20	25.60	15.00	14.2
345.0	465	79.00	47.00	32.00	16.90	21.00	18.00	5.0
340.0	332	61.70	40.40	21.30	13.00	25.20	9.00	3.1
337.0	289	67.20	43.60	23.60	10.60	18.70	12.00	4.6
338.0	313	66.10	44.00	22.10	11.30	23.50	12.00	11.5
348.0	483	79.00	48.80	30.20	22.40	26.60	20.20	5.0
332.0	175	72.40	45.80	26.60	251.00	249.40	380.00	54.4
342.0	263	67.30	46.60	20.70	6.00	12.70	13.00	11.8
333.0	274	69.30	44.50	24.80	8.30	17.00	10.00	10.1
324.0	376	62.00	43.80	18.20	13.20	29.90	9.00	4.9
328.0	350	68.30	44.50	23.80	13.40	22.10	17.00	8.5
333.0	283	68.60	45.60	23.00	16.20	24.20	12.00	5.1
340.0	255	66.10	40.70	25.40	7.00	19.50	10.00	5.7
332.0	311	67.10	44.70	22.40	11.00	22.60	12.00	14.8
338.0	268	69.80	44.90	24.90	6.00	20.50	10.00	9.9

DB	IDB	CRE	BUN	hsCRP	钾	钠	氯	钙
3.6	3.70	46.00	3.97	0.500	4.040	138.50	102.60	2.39
6.0	4.90	46.60	3.80	0.500	#NULL!	#NULL!	#NULL!	#NULL!
5.3	7.80	55.50	5.99	0.500	#NULL!	#NULL!	#NULL!	#NULL!
3.6	3.60	39.40	2.01	19.660	#NULL!	#NULL!	#NULL!	#NULL!
17.6	22.80	60.90	4.48	0.500	4.220	136.60	102.50	2.51
2.6	7.50	58.00	4.74	0.500	4.270	139.50	107.40	2.40
2.1	2.00	53.40	4.67	0.500	3.650	144.50	103.00	2.49
10.4	10.80	62.80	2.97	0.500	3.090	140.00	103.00	2.40
6.5	7.20	37.80	2.51	0.500	3.820	141.30	104.00	#NULL!
1.4	4.00	37.40	4.76	0.500	4.230	141.80	104.70	2.54
5.2	3.40	41.90	4.84	0.500	3.860	136.40	100.00	2.51
1.1	1.30	59.00	5.26	0.500	3.790	140.00	106.00	#NULL!
2.5	1.70	43.80	4.19	0.500	4.020	139.70	104.00	2.32
1.3	1.80	39.10	4.08	0.500	3.600	141.80	100.70	2.35
4.5	4.50	39.00	2.36	43.270	3.620	141.00	102.00	2.27
6.7	5.70	52.50	3.39	0.500	3.600	140.00	102.00	2.33
1.8	1.70	37.50	1.92	44.480	3.870	137.00	100.00	2.25
5.6	6.40	56.00	3.78	0.500	3.990	138.00	103.00	2.32
4.9	6.50	71.90	5.10	0.500	3.910	142.00	102.00	#NULL!
2.0	1.40	50.20	6.30	0.500	3.900	141.00	107.00	2.25
10.8	12.40	51.90	1.92	0.500	3.460	137.00	99.80	#NULL!
8.2	10.30	70.00	4.57	8.300	3.610	136.00	100.00	#NULL!
2.7	2.10	52.80	6.87	0.580	4.650	136.00	100.00	2.48
5.0	10.50	42.20	3.18	0.500	4.470	141.00	105.00	2.41
2.6	3.70	38.50	2.62	12.420	3.540	133.00	99.00	2.37
2.3	1.70	67.00	4.78	0.500	3.890	137.00	103.00	2.11
2.7	1.80	53.40	3.59	0.500	3.630	131.20	96.60	2.32
6.4	7.30	45.20	4.95	5.860	3.770	131.90	93.50	2.33
3.3	6.70	32.00	3.00	0.530	3.580	141.00	108.00	2.48
7.9	11.80	42.00	2.80	0.500	3.690	138.00	102.00	2.40
2.6	1.90	40.80	6.56	0.500	3.830	141.00	105.00	2.46
3.8	3.70	36.30	2.68	4.190	3.820	136.90	100.40	2.43
2.3	1.80	44.90	4.63	0.500	3.730	141.40	102.30	2.28
3.0	2.00	41.00	3.50	1.150	#NULL!	#NULL!	#NULL!	#NULL!
5.9	5.80	48.10	3.94	0.500	4.200	140.00	103.00	#NULL!
2.0	2.00	44.10	2.88	0.500	4.430	140.00	106.00	2.43
2.8	7.50	35.80	4.25	0.500	4.400	138.00	103.00	2.46
9.1	4.90	36.10	3.82	0.500	4.030	138.00	109.00	2.23
1.7	7.20	15.60	5.21	0.550	4.510	134.40	105.40	2.55
4.3	4.20	62.60	4.78	0.500	3.340	140.00	101.00	2.17
2.2	2.00	58.20	4.46	1.040	4.440	139.00	106.00	2.27
4.1	4.50	47.00	3.08	0.500	4.260	139.00	104.00	2.43
4.3	6.90	60.70	3.66	0.500	4.170	140.00	103.00	#NULL!
1.1	2.50	45.00	5.73	0.500	3.680	138.00	103.00	2.38
7.0	5.00	40.00	3.70	0.500	#NULL!	#NULL!	#NULL!	#NULL!
2.5	3.40	47.80	2.92	2.120	4.380	140.00	101.00	2.38
4.5	4.80	36.50	3.02	0.500	3.910	141.00	105.00	2.37
3.3	4.00	45.30	2.56	0.450	3.760	142.00	106.00	#NULL!
3.0	6.80	52.70	3.05	0.540	4.520	141.00	106.00	2.46
4.6	5.80	40.90	4.88	0.500	4.050	140.00	101.00	2.76
6.0	5.90	40.60	3.13	0.500	3.900	139.00	103.00	2.32
3.5	6.50	46.00	3.40	3.440	3.910	135.00	98.70	2.44
6.8	9.30	62.00	4.42	0.500	3.660	140.00	104.00	2.29

2.5	1.70	67.00	4.65	23.950	4.320	140.00	107.00	2.30
4.9	5.80	48.10	4.01	0.500	3.970	140.00	110.00	#NULL!
2.5	2.50	40.30	2.08	0.500	4.020	138.00	106.00	2.32
2.0	3.00	90.00	13.95	0.500	3.270	141.00	108.00	#NULL!
3.3	5.60	25.80	4.93	0.500	3.590	135.00	106.00	2.57
4.6	5.80	84.00	5.90	0.500	4.390	142.00	104.00	#NULL!
4.4	4.90	66.90	4.41	0.500	4.380	142.00	104.00	2.45
5.9	8.70	53.60	5.55	0.500	4.180	139.00	105.00	2.47
1.7	1.20	56.20	3.90	0.500	4.160	138.00	106.00	2.33
3.4	4.40	36.10	4.20	0.500	3.500	145.00	106.00	2.44
9.9	14.80	27.10	3.64	0.500	4.210	140.00	105.00	2.39
3.3	2.30	75.90	3.81	0.500	4.360	140.00	108.00	#NULL!
5.3	6.60	62.00	3.30	3.300	3.800	142.00	107.00	2.28
2.3	1.70	46.40	6.00	0.500	4.410	139.00	103.00	2.38
1.9	5.30	49.40	5.40	0.500	#NULL!	#NULL!	#NULL!	#NULL!
4.5	4.70	56.00	8.06	1.580	4.210	142.00	110.00	2.26
0.8	2.80	45.00	4.00	1.800	#NULL!	#NULL!	#NULL!	#NULL!
6.4	10.20	82.00	3.47	0.500	3.450	140.00	103.00	2.27
3.7	2.70	57.00	4.59	0.500	4.100	141.00	105.00	2.32
1.9	5.30	81.00	6.02	0.500	3.370	140.00	101.00	2.45
7.0	11.80	55.00	4.57	0.500	3.590	143.00	103.00	2.35
2.7	4.80	44.60	2.76	9.800	4.670	140.00	100.00	2.51
2.2	5.20	45.60	2.89	0.500	3.730	142.00	106.00	2.60
1.8	2.80	70.80	3.59	33.000	4.090	144.00	105.00	#NULL!
7.4	13.10	58.60	2.90	12.100	3.750	142.00	104.00	#NULL!
4.2	7.30	44.10	3.51	0.760	3.550	139.00	105.00	2.53
4.8	6.20	63.00	5.22	0.500	4.070	140.00	106.00	2.48
1.9	1.30	60.40	3.23	0.840	3.700	143.00	106.00	2.35
1.0	1.20	40.80	3.90	0.900	4.060	142.00	106.00	2.27
1.9	6.20	72.00	3.54	0.900	3.600	141.00	106.00	#NULL!
1.8	8.20	65.00	5.88	2.100	3.770	134.00	102.00	2.46
2.7	2.20	47.60	2.59	1.650	3.180	140.00	106.00	2.40
5.4	5.80	49.30	5.56	0.850	2.600	141.00	107.00	2.37
4.7	8.30	44.50	4.95	0.500	4.240	139.00	107.00	2.18
2.7	5.70	77.80	5.01	3.300	3.460	144.00	103.00	#NULL!
4.0	6.10	45.20	4.53	3.550	4.220	140.00	103.00	2.32
2.2	3.40	55.10	2.57	0.500	4.170	143.00	105.00	2.35
2.8	4.60	30.00	2.88	1.050	3.930	143.00	106.00	2.34
3.3	5.60	36.30	4.60	0.560	4.230	143.00	106.00	2.31
2.7	4.20	40.60	1.85	72.020	4.950	145.00	106.00	2.47
2.4	1.80	41.40	2.09	0.690	4.180	138.00	100.00	2.27
4.1	5.90	57.00	5.24	0.500	3.600	142.00	103.00	2.39
3.1	3.40	58.00	3.38	0.560	3.870	143.00	107.00	2.32
4.0	7.90	37.80	3.14	0.500	4.020	141.00	103.00	2.30
2.8	1.80	69.50	8.16	0.500	3.680	144.00	104.00	2.47
5.9	7.50	33.20	4.49	0.500	4.110	138.00	98.00	2.52
5.3	7.90	55.00	3.73	0.500	3.660	144.00	105.00	2.46
3.0	3.00	37.00	3.87	4.060	3.840	142.00	106.00	#NULL!
8.6	12.00	41.00	2.81	16.760	3.450	143.00	107.00	2.30
5.7	8.90	62.00	5.00	0.660	4.080	142.00	102.00	2.57
2.2	1.80	63.70	5.82	2.360	4.190	142.00	102.00	2.51
3.9	5.30	55.70	7.06	0.590	4.230	142.00	103.00	2.50
3.6	3.80	45.20	2.67	1.090	3.960	141.00	107.00	2.40
0.8	2.40	57.00	9.00	0.210	3.590	140.00	108.00	2.10

3.5	5.60	31.30	5.09	3.220	4.280	143.00	104.00	2.52
5.0	2.00	25.00	2.20	0.500	#NULL!	#NULL!	#NULL!	#NULL!
5.0	3.00	33.00	2.80	0.500	#NULL!	#NULL!	#NULL!	#NULL!
4.8	8.30	53.80	3.72	1.070	4.040	139.00	106.00	2.42
2.6	7.60	40.00	3.85	0.610	4.770	138.00	102.00	#NULL!
5.9	5.60	49.90	3.83	10.310	3.490	141.00	107.00	#NULL!
1.8	2.30	28.80	4.01	0.990	3.920	140.00	100.00	2.34
5.1	8.50	66.40	5.08	0.500	4.000	140.00	105.00	2.40
2.8	5.20	34.50	3.95	0.550	3.750	137.00	104.00	2.30
2.4	2.30	40.50	3.50	0.500	4.190	140.00	105.00	2.46
3.1	4.80	47.00	3.08	0.500	3.730	144.00	107.00	2.34
8.8	23.10	59.90	6.37	1.200	3.970	141.00	105.00	2.48
8.8	15.50	46.60	3.45	0.290	4.200	143.00	105.00	2.53
2.7	3.50	49.50	2.80	0.500	4.390	144.00	108.00	2.49
3.7	4.50	39.20	4.24	0.500	3.180	144.00	104.00	2.55
4.1	4.30	38.00	1.59	12.600	4.000	142.00	104.00	2.47
2.7	2.90	41.40	4.52	0.500	3.510	145.00	108.00	2.36
3.2	3.90	44.00	3.11	25.450	3.570	141.00	104.00	#NULL!
4.4	3.90	55.70	3.93	0.500	4.050	142.00	103.00	2.46
2.7	3.00	37.90	2.93	0.500	3.990	143.00	108.00	2.27
3.5	5.40	32.80	4.29	0.500	4.190	143.00	105.00	2.40
9.2	13.00	63.80	5.06	4.000	4.010	147.00	111.00	2.14
1.6	2.60	33.70	2.77	0.500	3.640	136.00	101.00	2.36
4.0	4.90	59.00	6.70	0.500	3.490	139.00	102.00	2.34
2.8	3.10	48.60	2.72	0.500	3.760	143.00	107.00	2.27
3.0	2.00	39.00	3.50	0.500	#NULL!	#NULL!	#NULL!	#NULL!
3.7	4.20	44.40	4.19	48.170	3.470	142.00	103.00	2.49
2.9	3.50	46.60	1.95	1.870	3.860	143.00	107.00	2.44
2.7	3.10	48.00	2.64	50.290	3.910	141.00	106.00	2.22
6.2	8.40	37.20	4.49	10.500	4.690	137.00	96.10	#NULL!
0.4	3.00	32.00	2.80	56.550	4.040	139.00	100.00	2.27
5.1	14.00	75.00	4.53	126.410	#NULL!	#NULL!	#NULL!	#NULL!
4.1	6.20	44.70	1.27	0.500	3.340	139.90	99.20	2.28
3.9	6.40	58.60	6.62	0.500	4.500	141.00	105.00	2.45
4.0	5.00	45.00	2.77	0.500	3.870	136.10	100.30	2.36
2.7	5.40	37.20	3.26	0.850	4.260	142.10	106.00	2.82
3.6	6.00	38.90	4.36	5.540	#NULL!	#NULL!	#NULL!	#NULL!
1.9	2.20	43.40	4.74	0.500	3.860	135.40	107.40	2.48
6.7	9.30	49.50	4.02	0.500	3.700	141.30	107.70	#NULL!
3.3	4.50	43.70	5.47	0.500	3.660	144.10	106.40	2.45
1.6	2.20	43.10	6.49	0.500	4.160	140.50	106.90	2.27
2.1	2.50	69.00	4.42	0.500	3.720	141.10	103.80	2.32
2.6	6.30	50.40	5.75	0.500	4.420	142.10	108.80	2.51
3.5	6.20	37.70	5.22	0.500	4.300	141.40	105.50	#NULL!
2.4	4.30	48.00	3.20	2.290	4.320	143.10	104.10	2.55
6.9	16.80	33.10	3.41	0.500	5.590	136.20	102.00	2.49
5.7	10.10	53.60	4.03	0.500	4.220	142.40	104.90	2.51
5.2	9.90	40.10	1.62	0.500	3.840	139.40	105.80	2.39
4.1	3.30	59.90	2.10	1.530	4.270	136.60	106.30	2.52
6.0	4.00	38.00	3.20	0.500	#NULL!	#NULL!	#NULL!	#NULL!
1.3	3.50	51.60	4.59	0.500	3.870	141.20	105.20	2.55
2.8	6.40	52.60	3.78	0.500	4.260	141.70	102.50	2.55
1.9	4.80	55.90	4.11	0.500	3.780	141.60	103.60	#NULL!
1.5	2.90	33.00	1.37	5.540	4.390	139.50	102.70	2.45

3.5	5.20	57.00	4.25	0.500	4.380	142.90	103.60	#NULL!
2.4	5.80	49.00	2.58	0.500	4.170	139.00	106.10	2.52
1.5	3.30	27.20	2.05	147.410	3.990	143.90	100.10	2.38
6.8	8.50	62.20	3.03	0.500	5.150	138.30	104.00	2.52
3.7	8.10	45.60	2.86	0.500	4.090	141.30	103.80	2.44
2.7	4.80	42.10	2.54	10.660	3.400	139.00	102.00	2.31
2.9	4.20	70.80	3.38	0.500	4.340	140.00	104.00	#NULL!
11.1	20.50	48.70	4.15	0.500	3.940	136.00	103.00	2.54
1.3	2.50	44.50	3.53	24.930	4.150	138.00	101.00	1.74
4.1	5.70	74.80	2.89	0.500	3.860	140.70	100.50	2.50
2.8	4.00	52.00	4.10	1.250	#NULL!	#NULL!	#NULL!	#NULL!
1.6	3.60	37.70	2.72	0.500	4.050	141.60	104.40	2.42
1.7	11.10	51.00	4.10	0.500	3.900	139.00	100.00	#NULL!
9.2	12.40	39.00	5.69	0.510	3.850	141.00	107.30	2.21
3.7	6.80	36.80	3.55	0.500	3.700	141.60	105.30	2.49
1.6	5.00	37.40	4.38	0.500	4.470	137.20	104.90	2.53
5.7	10.10	31.10	5.88	0.500	4.090	141.80	104.70	2.46
6.3	8.80	55.70	2.93	0.500	3.680	137.30	103.00	2.55
2.6	5.00	36.80	3.83	0.500	3.830	142.50	101.30	2.45
3.5	10.50	33.90	6.70	0.500	#NULL!	#NULL!	#NULL!	#NULL!
2.0	4.00	69.00	5.96	0.500	3.470	135.90	101.30	#NULL!
2.7	6.00	50.20	2.21	0.550	3.950	141.30	101.30	2.41
4.7	10.30	44.40	4.98	0.500	#NULL!	#NULL!	#NULL!	#NULL!
9.6	11.70	63.40	1.74	0.500	4.270	146.90	106.80	#NULL!
2.0	2.00	28.00	2.20	0.500	#NULL!	#NULL!	#NULL!	#NULL!
2.5	5.40	29.00	2.50	1.020	4.070	132.90	105.50	2.26
6.9	12.30	51.10	3.82	0.500	3.730	138.80	105.40	2.47
2.7	7.30	43.80	3.71	27.440	3.720	134.90	99.30	#NULL!
3.5	5.70	64.40	3.21	0.500	4.140	138.80	102.70	#NULL!
8.8	13.80	31.90	2.50	0.500	3.570	139.40	106.80	2.16
2.4	5.20	42.40	4.29	0.500	3.550	141.90	104.30	2.40
5.2	9.90	47.90	6.96	0.500	3.500	137.70	101.40	2.44
6.4	8.40	47.80	3.46	0.500	4.510	137.00	102.70	2.31
4.4	9.20	45.00	3.55	0.500	4.040	142.40	#NULL!	#NULL!
5.4	8.60	49.30	5.31	0.500	3.880	136.90	104.10	2.40
3.2	9.80	64.80	5.51	0.500	4.210	142.50	101.20	2.24
1.9	7.10	44.80	3.50	0.500	4.480	139.50	103.40	#NULL!
4.0	8.50	32.70	4.17	0.870	3.830	135.00	104.00	2.35
3.2	2.60	36.00	3.76	22.720	3.390	138.00	100.00	2.03
3.9	8.30	38.00	4.15	1.700	3.720	141.00	105.00	#NULL!
3.9	2.30	90.10	21.30	21.600	2.750	133.00	95.00	2.14
2.0	3.00	32.00	3.10	0.870	#NULL!	#NULL!	#NULL!	#NULL!
3.6	6.80	58.00	4.72	0.500	4.120	138.00	105.00	2.51
2.6	3.70	38.60	1.55	7.500	3.900	136.00	100.00	#NULL!
18.0	17.00	48.00	2.62	0.500	3.700	136.00	108.00	#NULL!
3.3	7.80	44.20	2.74	0.860	3.840	140.00	107.00	2.30
3.2	7.00	24.70	3.49	1.040	4.020	136.00	105.00	2.16
2.7	6.00	49.80	3.92	1.000	4.730	142.00	104.00	2.44
2.0	6.60	40.80	3.20	0.500	4.380	139.00	101.00	2.23
3.9	10.10	46.00	4.37	0.630	3.420	129.00	92.60	#NULL!
2.2	3.90	35.50	2.02	0.610	4.070	140.00	104.00	2.33
8.4	19.10	50.00	2.69	0.500	4.190	136.50	103.10	2.24
2.1	8.00	45.70	3.49	0.500	4.300	138.80	103.00	2.35
9.1	56.90	39.20	3.55	0.980	#NULL!	#NULL!	#NULL!	#NULL!

4.8	8.80	53.10	5.37	0.500	4.020	138.00	103.00	2.30
3.0	3.00	27.00	2.60	1.740	#NULL!	#NULL!	#NULL!	#NULL!
2.1	5.40	35.40	4.48	0.500	3.570	135.00	108.00	2.22
4.3	8.20	51.00	3.01	0.500	3.910	140.00	104.00	#NULL!
2.5	5.00	37.90	3.11	3.900	4.410	138.00	104.00	2.38
3.0	6.60	38.60	4.56	0.500	#NULL!	#NULL!	#NULL!	#NULL!
2.2	6.20	34.80	3.23	0.500	3.740	138.00	107.00	2.26
1.4	2.40	26.70	1.52	0.500	3.820	140.00	107.00	2.20
4.7	10.70	64.10	2.13	0.800	4.220	144.00	109.00	2.22
2.6	7.00	40.50	5.92	55.900	#NULL!	#NULL!	#NULL!	#NULL!
2.1	5.80	32.50	2.99	17.300	4.150	135.00	100.00	2.24
2.6	5.40	51.60	3.06	0.500	4.520	141.00	99.90	2.36
4.1	7.40	42.70	5.55	0.500	4.320	141.00	99.90	2.31
3.5	8.90	45.40	3.59	0.500	3.950	140.00	104.00	2.32
3.0	5.00	35.00	3.10	0.500	#NULL!	#NULL!	#NULL!	#NULL!
4.0	8.70	48.80	2.41	0.500	4.950	141.00	104.00	2.25
3.5	7.70	37.70	4.71	0.700	#NULL!	#NULL!	#NULL!	#NULL!
3.7	7.30	40.30	2.71	0.500	3.820	140.00	105.00	2.32
3.3	7.00	16.30	4.55	0.500	3.650	138.00	106.00	2.35
8.1	12.20	55.60	6.63	0.500	#NULL!	#NULL!	#NULL!	#NULL!
37.5	34.90	55.20	1.95	0.500	3.980	135.00	107.00	1.04
2.4	5.80	41.20	3.40	0.800	3.860	137.00	105.00	2.24
7.2	13.20	47.70	3.18	0.700	3.850	140.00	106.00	2.36
3.5	3.20	37.50	4.94	1.100	3.550	144.00	105.00	2.23
7.2	10.60	31.90	2.94	0.500	4.290	137.00	101.00	2.59
7.5	11.60	37.60	3.31	0.900	3.750	139.00	107.00	2.27
2.3	3.40	68.10	4.82	0.500	3.790	144.00	106.00	2.57
7.0	2.00	38.00	3.40	0.500	#NULL!	#NULL!	#NULL!	#NULL!
4.0	3.00	36.00	3.20	0.500	#NULL!	#NULL!	#NULL!	#NULL!
7.0	1.00	35.00	3.00	0.500	#NULL!	#NULL!	#NULL!	#NULL!
2.0	7.20	26.90	2.50	32.400	4.190	140.00	102.00	2.40
2.8	4.10	36.70	4.82	0.500	4.040	139.00	104.00	2.41
6.3	4.60	34.50	3.33	158.500	5.240	132.00	94.00	2.44
4.9	2.90	32.60	4.46	0.500	4.510	145.00	105.00	2.31
2.1	7.40	54.30	2.69	0.500	3.920	141.00	105.00	2.31
1.7	8.10	471.00	2.70	0.500	4.160	136.00	107.00	2.43
2.5	6.90	49.30	2.62	0.500	3.770	140.00	104.00	2.23
3.5	3.60	57.80	5.68	0.500	3.760	141.00	102.00	2.41
3.7	4.50	27.60	2.38	2.200	3.600	141.00	105.00	1.98
1.8	8.20	54.20	3.37	0.500	3.670	140.00	105.00	2.17
2.3	6.20	42.60	2.91	0.500	4.280	142.00	106.00	2.19
3.0	2.00	34.00	3.10	17.000	#NULL!	#NULL!	#NULL!	#NULL!
1.7	10.50	65.40	6.04	0.500	3.760	138.00	99.00	2.32
4.0	8.00	69.20	4.94	3.200	4.190	130.00	101.00	2.42
1.8	3.90	42.90	6.59	0.500	3.870	139.00	107.00	2.31
14.3	18.90	33.50	2.33	0.500	3.980	140.00	105.00	2.38
53.9	36.80	30.80	2.44	0.500	3.140	137.00	103.00	2.21
2.3	9.50	54.80	5.30	0.500	#NULL!	#NULL!	#NULL!	#NULL!
6.6	10.20	47.30	4.78	0.500	4.260	136.00	98.00	2.36
4.2	8.90	37.30	3.51	0.500	3.850	141.00	102.00	#NULL!
5.0	4.00	42.00	4.00	0.500	#NULL!	#NULL!	#NULL!	#NULL!
3.3	7.20	34.00	5.33	0.500	4.040	136.00	99.00	#NULL!
4.3	7.50	36.80	2.84	0.500	4.590	138.00	104.00	2.31
2.5	5.10	40.30	6.67	0.500	5.540	137.00	99.00	2.45

2.6	5.40	39.30	5.79	0.500	#NULL!	#NULL!	#NULL!	#NULL!
2.4	7.30	51.00	3.80	0.500	#NULL!	#NULL!	#NULL!	#NULL!
6.0	4.00	62.00	4.20	0.500	#NULL!	#NULL!	#NULL!	#NULL!
2.3	3.10	75.20	6.11	0.500	#NULL!	#NULL!	#NULL!	#NULL!
7.0	5.00	28.00	2.20	2.000	#NULL!	#NULL!	#NULL!	#NULL!
4.9	10.00	50.00	3.80	136.000	#NULL!	#NULL!	#NULL!	#NULL!
6.0	4.00	34.00	3.00	0.500	#NULL!	#NULL!	#NULL!	#NULL!
3.0	2.00	41.00	2.80	0.500	#NULL!	#NULL!	#NULL!	#NULL!
1.2	4.20	42.50	5.48	0.500	4.180	143.00	107.00	2.37
2.0	3.50	32.00	2.70	0.500	#NULL!	#NULL!	#NULL!	#NULL!
2.0	2.40	35.00	3.00	0.500	3.950	143.00	104.00	2.16
7.4	13.30	47.00	3.50	0.500	4.000	140.00	100.00	2.41
7.3	19.20	35.90	10.89	0.500	3.800	135.00	103.00	2.32
2.2	7.00	54.50	1.73	0.500	4.280	144.00	105.00	#NULL!
6.0	7.10	56.70	5.26	0.500	4.730	134.00	94.00	2.21
7.2	15.00	60.60	3.53	0.500	3.990	140.00	99.90	#NULL!
3.0	5.00	32.80	5.92	0.500	4.000	139.00	101.00	#NULL!
2.5	6.40	64.20	5.89	0.500	4.220	141.00	101.00	2.46
2.5	7.40	51.20	3.83	0.500	5.310	139.00	101.00	2.49
3.2	4.00	39.90	3.98	0.500	4.520	143.00	105.00	2.43
160.0	151.00	27.10	1.96	0.500	3.350	142.00	104.00	#NULL!
2.5	3.40	35.30	2.65	0.500	3.800	143.00	102.00	2.45
5.0	3.00	35.00	3.10	0.500	#NULL!	#NULL!	#NULL!	#NULL!
6.0	11.10	47.90	6.03	0.500	4.070	143.00	104.00	2.44
7.0	7.00	36.00	3.20	0.500	#NULL!	#NULL!	#NULL!	#NULL!
2.0	3.00	35.00	3.20	0.500	#NULL!	#NULL!	#NULL!	#NULL!
4.2	10.00	35.00	3.10	0.500	4.180	135.00	102.00	2.32
7.3	12.00	64.70	1.84	0.500	4.100	138.00	101.00	#NULL!
2.0	6.60	79.90	4.23	0.500	4.600	137.00	101.00	#NULL!
1.7	7.40	67.30	4.54	0.500	4.400	137.00	101.00	2.40
8.0	3.00	33.00	2.90	0.500	#NULL!	#NULL!	#NULL!	#NULL!
2.4	7.10	32.00	3.00	5.000	3.800	137.00	103.00	2.37
5.0	3.00	34.00	3.10	0.500	#NULL!	#NULL!	#NULL!	#NULL!
6.5	10.30	46.80	5.21	0.500	4.100	142.00	103.00	2.46
6.0	3.00	36.00	2.80	0.500	#NULL!	#NULL!	#NULL!	#NULL!
2.1	8.20	57.60	2.88	0.500	4.480	138.00	105.00	2.39
3.9	9.90	43.20	3.39	0.500	3.500	142.00	107.00	2.54
8.0	4.00	34.00	3.10	0.500	#NULL!	#NULL!	#NULL!	#NULL!
1.6	0.90	56.80	5.88	1.200	3.980	140.00	108.00	2.39
4.2	3.00	42.90	2.57	72.530	3.890	140.00	103.00	2.40
4.4	3.20	57.10	3.65	0.500	3.620	140.00	106.00	2.21
3.4	3.30	54.80	2.53	0.500	3.550	141.00	108.00	2.32
9.2	1.30	14.50	2.23	150.540	3.540	136.00	95.50	2.11
5.2	7.90	59.10	3.46	0.500	3.950	145.00	107.00	2.47
5.0	3.00	28.00	2.40	0.500	#NULL!	#NULL!	#NULL!	#NULL!
3.0	3.40	33.70	5.27	0.030	3.930	140.00	103.00	2.17
5.2	8.80	45.20	2.85	0.500	3.680	142.00	106.00	2.45
3.4	3.80	51.50	2.54	3.020	3.890	142.00	107.00	#NULL!
3.0	4.90	18.80	2.91	0.500	3.680	144.00	108.00	2.53
3.7	4.60	60.70	6.38	0.500	3.690	141.00	106.00	2.57
2.9	2.70	55.00	3.67	0.500	3.640	142.00	103.00	2.23
5.5	14.10	56.20	3.67	0.500	4.130	138.00	103.00	2.21
4.1	3.80	71.90	5.67	0.500	3.670	143.00	107.00	2.44
2.7	2.00	37.60	2.25	8.560	3.670	139.00	106.00	2.21

8.0	11.90	72.30	7.36	0.500	3.400	141.00	106.00	2.41
3.4	3.70	38.00	4.60	5.710	3.430	140.00	103.00	2.06
2.7	2.60	44.00	2.63	0.500	3.510	144.00	103.00	2.31
9.2	18.10	54.00	2.75	0.500	3.790	143.00	109.00	2.33
3.7	8.10	42.90	5.16	1.880	3.770	140.00	105.00	2.50
3.0	3.40	39.30	3.08	0.500	4.060	141.00	106.00	2.47
5.4	6.90	56.80	3.26	0.500	3.420	143.00	107.00	2.33
3.9	6.30	74.90	3.34	0.500	3.060	147.00	108.00	2.41
3.2	2.10	40.90	2.90	1.180	3.490	142.00	105.00	2.38
5.2	6.60	55.00	2.60	0.500	3.980	142.00	100.00	2.56
3.0	2.90	42.00	1.76	0.500	3.820	138.00	106.00	2.22
5.0	9.00	66.70	5.38	1.640	3.030	142.00	106.00	2.26
3.6	4.00	68.00	3.63	10.650	3.490	139.00	104.00	2.38
2.4	2.50	44.60	7.47	0.500	4.230	137.00	99.50	2.55
2.8	2.10	46.90	3.76	0.500	3.810	139.00	105.00	2.35
4.5	7.10	44.50	3.18	0.200	3.580	138.00	103.00	2.38
2.2	6.90	77.80	4.26	0.570	3.410	140.00	102.00	2.10
2.5	2.50	43.80	4.44	0.500	3.420	142.00	102.00	2.39
5.6	7.20	51.60	2.83	1.020	3.390	140.00	103.00	2.41
4.4	5.70	562.00	7.06	2.230	3.680	143.00	106.00	2.43
3.7	3.60	48.60	2.86	61.280	3.740	138.00	101.00	2.33
9.0	4.00	34.00	3.10	0.500	#NULL!	#NULL!	#NULL!	#NULL!
3.6	4.80	31.00	3.90	0.990	4.100	140.00	103.00	2.45
3.9	5.20	54.60	3.37	0.500	3.080	145.00	106.00	#NULL!
4.3	4.40	64.70	3.87	0.500	4.030	142.00	105.00	2.35
2.0	2.20	43.50	4.74	16.470	4.500	141.00	103.00	2.29
5.4	5.30	44.80	4.13	0.500	3.550	142.00	106.00	2.35
4.1	3.80	57.00	3.60	1.660	4.090	141.00	103.00	2.44
2.7	3.50	51.40	3.39	0.500	4.190	136.00	100.00	2.36
2.1	2.50	52.00	5.49	0.500	4.350	139.00	102.00	2.42
0.8	1.60	43.30	3.90	0.500	3.880	139.00	100.00	2.16
1.5	3.90	38.30	4.30	64.820	3.180	133.00	100.00	2.20
3.1	4.00	53.00	4.06	0.500	3.830	140.00	104.00	2.40
4.1	9.60	43.00	4.15	1.400	3.940	135.00	101.00	2.23
3.5	6.30	57.00	8.22	0.500	3.820	141.00	103.00	2.63
3.7	3.40	29.00	2.50	167.040	#NULL!	#NULL!	#NULL!	#NULL!
0.6	0.70	52.80	5.95	14.100	3.300	147.00	113.00	2.27
1.4	2.00	31.10	3.07	26.920	3.720	143.00	109.00	2.10
7.0	3.00	36.00	3.40	0.500	#NULL!	#NULL!	#NULL!	#NULL!
3.5	6.30	48.40	4.18	0.500	3.790	143.00	108.00	2.32
4.7	7.40	64.00	4.15	0.500	4.200	141.00	102.00	2.44
3.2	5.90	31.70	4.47	7.160	3.760	138.00	100.00	2.80
4.6	7.30	46.70	4.25	0.500	3.790	138.00	102.00	2.46
1.6	1.50	56.30	2.74	0.500	4.290	139.00	100.00	2.41
2.5	3.00	42.70	2.61	19.750	3.820	138.00	101.00	2.34
2.5	3.90	69.00	2.88	0.500	3.710	149.00	111.00	2.55
3.3	2.70	51.00	2.52	0.500	4.370	146.00	113.00	2.38
2.2	3.50	36.70	3.70	0.500	4.440	138.00	101.00	2.36
2.5	2.40	33.00	4.35	15.510	4.500	137.00	103.00	2.44
6.4	12.20	59.00	4.66	0.500	3.940	141.00	104.00	2.34
2.7	2.40	32.70	3.50	1.860	4.140	145.00	108.00	2.24
2.4	6.30	44.70	3.56	0.500	4.340	143.00	108.00	2.46
4.8	9.80	59.70	6.26	0.500	4.100	141.00	106.00	2.41
2.5	2.20	47.00	3.69	4.020	3.840	146.00	104.00	2.52

3.4	3.80	64.00	4.04	17.000	3.910	144.00	106.00	#NULL!
1.9	2.50	66.70	7.84	0.840	4.180	139.00	107.00	2.37
3.3	3.90	51.00	2.69	111.960	3.780	138.00	103.00	2.24
2.7	8.10	63.00	2.96	1.370	3.460	144.00	101.00	2.28
5.2	7.60	70.00	3.74	3.130	4.780	144.00	112.00	#NULL!
5.9	8.50	43.60	3.61	0.500	4.320	140.00	106.00	2.44
2.5	3.60	44.00	4.48	0.500	3.760	144.00	108.00	2.46
3.0	5.90	50.00	2.47	2.020	3.830	142.00	108.00	2.35
2.4	2.90	29.00	1.95	93.670	4.030	142.00	101.00	2.40
2.9	3.80	46.60	8.46	0.500	3.690	141.00	104.00	2.39
2.1	2.40	49.00	5.17	0.840	3.870	141.00	107.00	2.32
1.5	1.50	37.00	3.10	0.500	4.010	142.00	106.00	2.40
3.0	4.80	46.70	4.25	0.930	4.130	141.10	102.70	2.49
6.1	10.40	49.00	3.32	3.130	3.620	141.00	104.00	2.36
3.9	5.90	47.00	3.80	0.500	4.800	143.00	104.00	2.03
3.2	5.80	34.20	6.78	11.140	3.970	138.00	103.00	2.47
5.8	14.30	60.30	5.37	0.500	4.060	143.00	106.00	2.41
11.4	7.30	33.00	2.54	1.920	3.690	144.50	107.00	2.30
2.3	3.90	50.90	5.22	0.950	4.000	140.00	105.00	2.03
4.6	7.00	43.20	2.81	0.500	4.230	143.00	107.00	2.38
2.2	4.30	41.00	4.18	2.400	4.230	140.00	103.00	2.04
3.0	4.60	51.10	3.62	0.500	3.820	144.00	109.00	2.42
2.2	2.90	41.00	4.09	0.500	3.530	142.00	104.00	2.44
2.8	3.40	41.00	3.84	0.590	4.400	139.00	104.00	2.40
3.9	5.90	39.20	3.07	0.500	3.900	142.00	106.00	2.42
1.9	1.20	51.20	3.50	0.500	3.790	139.00	104.00	2.40
4.2	6.40	45.00	3.63	0.500	4.030	138.00	103.00	2.43
5.9	7.40	55.30	3.12	0.500	3.440	142.00	107.00	#NULL!
1.6	1.40	41.00	5.22	3.230	4.080	140.00	108.00	2.27
4.3	4.30	51.80	3.20	11.170	4.280	145.00	102.00	2.36
0.7	2.60	37.60	3.99	0.500	3.110	140.00	107.00	1.69
4.0	3.00	32.00	2.90	0.500	3.750	139.00	103.00	2.60
2.9	3.60	50.00	3.54	0.500	3.810	142.00	108.00	2.46
2.2	3.30	46.00	5.16	0.500	4.180	142.00	105.00	2.32
0.4	6.30	29.50	3.16	13.370	4.660	137.00	103.00	2.29
1.8	1.90	33.00	2.50	0.500	3.930	139.00	103.00	2.33
2.5	2.30	36.40	4.07	0.500	3.680	139.00	102.00	#NULL!
10.6	10.20	58.30	3.97	29.600	3.980	138.00	100.00	2.24
3.2	3.00	46.30	6.31	0.500	3.850	144.00	107.00	2.35
2.6	3.20	34.70	1.84	60.090	3.560	145.00	108.00	2.19
5.1	5.20	52.00	5.53	0.500	3.790	140.00	106.00	2.30
2.3	2.70	51.00	5.59	19.670	4.020	137.00	102.00	2.33
2.7	2.70	51.80	2.21	0.500	3.230	142.00	100.00	2.29
7.7	10.30	61.60	2.76	0.500	3.960	139.00	104.00	2.39
5.0	4.00	37.00	3.30	0.500	#NULL!	#NULL!	#NULL!	#NULL!
4.4	5.70	42.00	3.33	0.500	3.830	138.00	104.00	2.21
8.7	4.10	61.90	4.39	0.500	3.490	135.00	101.00	2.09
7.2	9.90	77.00	4.72	3.230	4.090	139.00	103.00	2.44
5.8	5.50	38.70	5.25	0.500	3.670	140.00	104.00	2.52
4.7	5.30	85.30	3.97	1.350	3.990	143.00	105.00	2.41
5.0	3.00	25.00	2.10	0.500	#NULL!	#NULL!	#NULL!	#NULL!
1.8	10.30	68.00	2.37	0.500	3.210	142.00	104.00	2.29
3.5	4.70	54.00	3.48	2.940	3.750	142.00	101.00	2.52
8.1	11.30	63.50	3.90	3.230	3.250	141.00	102.00	2.44

4.5	4.80	42.00	5.00	0.500	3.710	139.00	105.00	2.26
3.7	3.10	49.00	3.63	0.500	3.200	144.00	106.00	2.38
5.5	5.90	53.00	4.67	5.600	4.420	138.00	102.00	2.57
1.9	1.70	51.00	4.31	1.360	3.630	139.00	103.00	2.26
6.3	7.30	65.00	3.12	3.230	3.660	140.00	101.00	#NULL!
4.8	7.40	58.00	4.13	0.500	3.080	142.00	104.00	2.30
2.8	2.40	39.40	6.07	0.500	3.450	140.00	100.00	2.49
2.4	1.70	50.00	2.27	0.500	3.640	142.00	102.00	2.28
4.4	4.00	61.20	7.06	0.500	3.880	137.00	103.00	2.35
2.6	1.90	58.00	3.23	26.770	3.800	141.00	106.00	2.41
2.5	2.40	61.90	4.68	0.500	3.900	137.00	100.00	2.40
3.2	3.50	40.50	5.22	0.500	3.760	137.00	104.00	2.47
3.1	3.00	40.30	3.62	0.500	4.210	138.00	103.00	2.42
3.1	3.40	44.00	3.93	1.030	3.980	138.00	105.00	2.43
2.3	0.80	52.50	4.12	0.500	5.350	139.10	104.40	2.43
3.8	4.60	65.30	4.63	3.920	3.960	140.80	101.10	2.55
6.4	6.80	62.50	9.53	3.230	2.870	138.90	98.80	2.20
2.4	3.10	76.50	4.31	1.240	3.740	140.40	103.00	2.32
6.2	10.50	52.90	2.47	0.500	3.650	143.00	103.00	2.12
3.0	3.50	43.30	5.09	1.170	4.240	138.00	99.60	2.45
2.4	3.40	85.00	4.02	0.500	4.110	143.00	106.00	2.25
2.8	4.00	78.00	5.67	0.500	3.610	142.00	106.00	2.49
2.3	1.50	74.00	3.41	0.500	3.700	138.00	101.00	2.51
2.8	1.70	88.00	7.60	0.500	4.240	141.00	103.00	2.32
1.3	0.50	47.80	2.97	9.480	3.730	138.00	104.00	2.52
3.4	5.20	50.60	3.18	1.880	3.540	139.00	103.00	3.03
2.1	1.00	31.00	4.93	7.280	4.130	138.00	102.00	2.20
7.1	9.10	51.00	4.88	0.500	3.730	142.00	103.00	2.33
3.5	3.60	36.70	4.28	0.500	3.820	140.00	102.00	2.62
2.7	2.50	51.00	4.56	0.500	3.710	135.00	103.00	2.44
4.0	5.40	55.00	4.45	0.500	3.710	139.00	101.00	2.41
2.4	2.60	48.00	3.37	0.600	4.520	141.00	103.00	2.52
4.8	6.00	53.00	4.64	0.500	3.640	141.00	103.00	2.49
3.7	4.40	44.00	2.67	0.500	3.640	153.00	91.60	2.10
3.0	5.70	62.60	3.75	0.500	3.510	141.00	103.00	2.44
5.9	7.90	52.00	3.52	0.500	3.750	140.00	102.00	2.52
4.9	8.40	39.80	3.07	0.500	3.610	142.00	105.00	2.40
6.3	9.80	46.10	3.75	0.500	3.660	141.00	103.00	2.38
1.8	0.50	43.00	4.42	0.670	3.690	129.00	101.00	2.43
3.0	5.60	52.50	4.90	0.500	3.930	142.00	105.00	2.46
4.6	2.80	59.00	5.06	0.500	4.120	140.00	103.00	2.46
4.4	5.30	50.60	3.31	0.500	3.890	141.00	105.00	2.29
2.7	1.80	60.20	5.92	0.500	4.310	142.00	102.00	2.57
3.3	4.90	45.00	4.12	0.500	3.700	142.00	102.00	2.45
2.2	2.40	25.00	3.11	0.500	4.510	136.00	99.20	2.38
3.8	4.30	55.50	5.00	6.020	3.880	138.00	100.00	2.41
3.5	6.70	51.00	5.50	0.500	3.890	139.00	102.00	2.46
1.7	2.20	48.00	5.00	0.500	4.870	140.00	105.00	2.26
4.8	3.60	62.80	2.42	1.810	3.660	143.00	103.00	2.33
3.6	2.60	35.70	3.14	3.000	3.790	142.00	108.00	2.36
3.1	8.20	51.80	4.68	12.990	3.710	141.00	102.00	2.18
1.4	4.50	58.90	2.53	1.010	3.610	143.00	108.00	2.09
6.5	7.40	51.90	2.30	0.650	3.630	143.00	104.00	2.48
4.1	3.70	57.00	4.44	0.500	4.500	145.00	104.00	2.56

2.2	1.60	41.00	4.96	1.560	3.730	139.00	101.00	2.49
4.4	4.60	51.90	2.65	2.570	4.150	147.00	108.00	#NULL!
6.7	8.50	42.60	5.44	0.520	3.540	141.00	107.00	2.43
4.0	4.00	36.00	3.20	0.500	#NULL!	#NULL!	#NULL!	#NULL!
3.3	3.20	42.00	3.29	13.850	4.370	141.00	102.00	2.37
0.8	1.10	37.00	3.96	45.740	3.770	138.00	100.00	2.27
4.5	3.80	44.80	4.48	0.500	3.860	138.00	106.00	2.18
1.9	1.20	38.00	4.59	0.500	3.490	139.00	106.00	2.28
3.0	2.00	26.00	2.00	0.500	#NULL!	#NULL!	#NULL!	#NULL!
4.7	3.70	75.60	5.39	6.960	4.140	138.00	103.00	2.17
5.0	2.00	38.00	3.50	0.500	#NULL!	#NULL!	#NULL!	#NULL!
2.4	7.70	51.00	3.95	92.330	4.010	141.00	105.00	2.13
1.4	1.30	57.10	7.60	0.500	4.120	138.00	101.00	2.34
2.6	2.40	59.30	3.21	0.500	4.390	139.00	105.00	#NULL!
3.4	3.00	24.00	2.20	0.500	3.640	135.00	96.60	2.02
3.9	5.30	54.80	4.45	1.130	3.700	141.00	104.00	#NULL!
4.0	5.00	32.00	3.00	0.500	#NULL!	#NULL!	#NULL!	#NULL!
2.9	4.30	41.90	2.60	0.500	3.690	138.00	102.00	#NULL!
1.4	0.60	57.30	6.06	0.500	4.040	138.00	103.70	2.65
10.1	18.20	88.70	7.02	0.500	4.110	138.80	100.60	#NULL!
4.0	5.00	30.00	2.70	0.500	#NULL!	#NULL!	#NULL!	#NULL!
4.4	3.00	69.10	7.03	0.500	4.160	130.90	101.00	#NULL!
2.0	1.60	36.00	4.20	0.500	4.670	142.00	101.00	2.52
19.6	3.20	49.50	6.84	152.000	3.950	141.00	102.30	2.30
3.3	4.90	48.10	5.20	0.500	4.240	140.00	103.80	2.42
2.3	1.50	55.20	5.66	0.500	3.700	144.00	103.00	2.37
4.7	3.50	41.60	5.06	0.500	3.780	141.40	102.70	2.42
6.3	5.90	63.60	7.78	6.700	3.790	140.00	101.00	2.22
4.0	6.90	45.90	2.71	0.500	3.580	139.00	102.90	2.32
3.0	2.70	47.00	4.93	0.500	3.780	138.00	95.00	2.38
2.7	2.70	47.00	4.20	0.500	3.730	143.00	103.90	2.30
6.5	8.30	63.90	3.59	18.220	3.660	142.00	99.90	2.27
4.0	3.00	27.00	2.30	59.500	#NULL!	#NULL!	#NULL!	#NULL!
2.5	2.30	55.90	3.13	2.930	3.800	138.60	100.70	2.19
21.4	7.10	69.70	4.64	11.200	3.790	137.70	102.30	2.02
3.2	3.60	51.10	3.58	0.500	3.370	141.20	104.90	2.36
2.0	6.10	48.30	2.64	0.500	3.930	142.00	102.50	2.47
6.7	6.10	45.40	3.36	104.000	3.860	136.00	101.00	2.14
2.9	3.30	42.80	5.15	4.090	3.650	142.10	104.10	2.60
4.7	7.40	56.00	4.68	0.500	4.080	140.30	102.70	2.34
5.2	12.90	64.70	4.20	0.500	3.730	130.00	101.00	2.41
4.3	4.80	44.70	5.42	0.500	3.560	140.00	103.00	2.29
3.7	3.70	52.70	2.86	0.500	3.590	142.70	101.70	2.38
5.4	6.10	67.10	3.64	3.410	3.790	141.00	104.50	2.72
5.7	5.20	39.30	4.64	0.500	3.980	138.00	100.00	2.51
7.4	9.60	57.50	3.64	0.500	4.150	143.00	105.00	2.28
5.5	8.30	46.00	4.14	0.500	3.900	144.00	104.90	2.51
3.2	3.70	43.00	2.19	0.500	3.850	146.00	107.20	2.48
4.5	4.70	44.20	2.51	0.500	3.160	143.00	100.00	2.57
4.5	6.00	64.00	4.08	3.000	4.340	140.00	102.30	2.44
3.0	3.00	63.00	4.33	0.500	3.620	141.00	105.00	#NULL!
2.6	2.00	59.70	4.35	0.750	4.350	141.70	104.00	2.42
2.2	2.60	51.00	4.50	1.290	3.430	139.00	101.00	1.89
3.2	4.00	48.00	4.58	1.250	3.770	143.00	103.00	2.33

3.3	3.40	38.60	4.70	2.340	3.690	139.00	103.20	2.34
3.3	4.60	50.60	3.11	0.500	3.940	141.00	101.70	2.48
2.9	2.70	48.00	6.00	0.500	4.100	142.00	103.00	2.38
4.0	4.90	57.80	4.10	0.500	3.730	146.00	108.00	2.25
4.2	5.60	56.50	4.75	0.500	3.900	143.00	105.00	2.38
1.9	1.10	27.00	2.56	32.600	3.450	146.00	111.00	2.10
4.0	4.90	39.60	3.69	0.500	3.720	142.00	105.00	2.31
1.9	1.90	45.40	3.47	0.500	3.810	143.10	103.90	2.43
3.8	4.00	61.30	5.12	0.500	3.850	140.30	103.80	2.42
5.0	4.30	45.10	3.85	0.500	3.810	142.80	102.50	2.40
2.0	2.00	28.00	2.10	0.500	#NULL!	#NULL!	#NULL!	#NULL!
3.9	5.30	52.20	3.13	0.500	4.140	143.50	109.30	2.34
5.5	6.30	35.10	3.47	0.500	4.240	143.40	100.90	2.45
5.8	6.70	63.30	5.57	0.500	4.010	145.10	109.20	2.39
1.6	2.50	52.90	2.66	0.500	5.470	141.90	96.20	2.35
6.4	13.90	61.00	6.06	0.500	4.330	143.30	105.70	2.46
2.5	2.80	54.50	6.87	0.500	4.220	142.70	106.00	2.47
6.1	6.50	43.80	3.95	0.500	4.010	142.90	105.20	2.38
4.2	5.70	44.10	3.99	0.500	4.060	141.00	108.20	2.33
1.9	2.70	55.60	3.36	0.500	4.210	141.00	106.30	#NULL!
3.3	4.70	49.30	4.09	3.000	3.810	144.00	107.00	2.41
3.1	4.20	50.90	4.58	0.500	4.160	142.80	106.70	2.51
3.3	3.80	51.60	3.40	3.000	4.810	141.00	105.00	2.34
4.7	5.90	48.40	3.01	0.500	3.900	140.00	103.80	2.33
2.4	2.60	51.60	3.89	2.490	4.130	141.60	103.10	2.40
2.3	2.30	52.00	4.32	4.040	3.790	140.00	105.00	2.32
3.8	4.30	64.90	4.43	7.800	4.010	144.00	105.00	2.56
3.7	3.20	64.60	4.33	0.500	3.740	141.00	105.00	2.35
4.3	5.30	34.70	2.02	0.500	4.260	139.90	103.80	2.32
5.8	6.50	39.40	1.98	1.680	3.910	139.30	105.40	2.38
5.1	6.40	45.70	2.69	2.120	3.450	136.80	101.50	2.28
3.7	4.30	39.20	1.39	0.500	3.930	146.70	107.60	2.38
6.1	8.50	52.40	2.68	0.500	3.600	140.00	103.60	2.16
2.2	2.40	46.00	4.55	3.820	4.300	135.40	101.90	2.28
4.4	5.30	46.60	3.26	0.500	3.970	142.40	108.80	2.41
5.0	3.00	29.00	2.50	0.500	#NULL!	#NULL!	#NULL!	#NULL!
2.4	4.10	24.80	2.82	0.500	4.360	139.70	104.50	2.54
4.4	7.30	69.00	5.39	0.500	3.980	139.60	104.50	2.55
3.0	2.00	67.00	2.20	0.460	3.340	142.90	106.30	2.27
3.7	6.20	60.40	4.35	0.500	4.110	142.20	100.90	2.41
2.3	1.40	44.40	5.95	1.300	4.010	140.70	103.40	2.46
4.2	4.60	51.60	3.63	0.500	#NULL!	#NULL!	#NULL!	#NULL!
1.7	1.10	58.10	3.42	0.500	3.730	142.10	102.90	2.41
11.5	12.60	46.70	3.60	0.500	3.720	142.40	103.60	2.24
1.3	2.90	36.00	7.20	0.500	3.850	140.00	104.60	#NULL!
2.3	2.10	45.00	6.14	0.410	4.220	143.50	106.50	#NULL!
5.3	5.60	56.50	4.15	0.500	3.460	141.60	100.50	2.38
3.7	3.20	54.80	2.35	4.870	#NULL!	#NULL!	#NULL!	#NULL!
4.3	4.90	52.50	2.90	0.500	#NULL!	#NULL!	#NULL!	#NULL!
3.4	5.40	49.00	5.14	0.500	3.850	140.50	102.10	2.53
3.5	3.60	39.80	3.67	0.500	4.020	137.40	99.60	2.44
3.7	10.30	43.10	1.60	0.500	4.010	141.60	104.50	2.28
3.1	3.30	58.10	3.31	0.500	4.110	142.50	103.70	2.41
3.4	3.20	62.30	6.08	1.100	3.690	139.50	102.70	#NULL!

1.5	4.10	35.80	3.48	0.500	3.590	139.40	105.90	2.48
3.6	4.60	51.90	5.68	0.550	3.640	143.30	108.00	2.31
0.4	1.30	43.00	4.40	0.500	3.750	142.00	105.90	2.40
4.1	4.30	56.80	2.81	0.500	3.470	139.10	104.40	2.16
7.2	12.40	49.30	1.90	0.500	3.120	142.40	104.30	2.32
4.4	4.80	48.00	5.54	0.500	4.060	139.70	102.20	2.53
4.3	3.40	57.90	5.52	0.500	4.170	139.60	101.10	2.36
9.4	14.00	76.90	7.42	7.850	#NULL!	#NULL!	#NULL!	#NULL!
6.1	7.20	45.20	3.86	0.500	3.670	135.70	106.50	2.43
6.5	8.90	42.60	3.12	93.180	3.270	136.60	100.50	2.39
4.0	4.50	57.20	3.00	0.500	4.110	144.40	102.50	2.32
4.7	5.10	47.30	3.50	0.500	3.480	142.80	100.30	2.32
2.2	1.20	39.60	4.31	0.500	3.740	144.90	108.70	2.46
4.6	4.80	40.90	3.04	27.450	4.000	139.80	102.10	2.47
2.5	2.70	39.00	6.49	0.520	3.860	142.50	104.90	2.61
8.5	11.20	54.50	4.16	0.500	3.770	141.20	102.50	2.51
5.8	6.50	81.10	4.08	0.500	3.710	142.80	104.90	2.23
5.1	6.50	50.30	4.63	0.440	3.410	136.40	98.60	2.28
7.1	10.20	69.80	4.20	0.500	3.680	141.80	103.60	2.35
3.9	10.40	56.10	4.53	0.500	3.640	142.20	104.80	2.54
4.1	4.10	38.60	6.03	0.500	4.020	143.80	108.10	2.60
4.7	3.90	58.40	5.09	0.500	3.980	145.40	107.70	2.43
5.0	5.40	51.70	6.01	0.500	#NULL!	#NULL!	#NULL!	#NULL!
9.0	12.40	55.80	3.69	0.500	3.540	144.00	107.20	2.59
1.7	1.10	43.00	5.60	0.500	3.720	141.70	99.00	2.41
3.9	4.00	51.70	3.75	0.500	3.360	146.00	107.00	2.42
5.7	5.20	55.50	2.50	74.030	3.720	139.60	102.00	2.21
2.2	2.50	27.30	4.60	0.500	4.080	143.90	110.30	2.47
3.6	2.60	52.30	3.77	0.500	3.920	144.60	108.10	2.47
6.9	10.00	52.30	3.91	0.500	3.840	144.90	105.60	2.39
3.4	3.20	40.10	4.79	12.890	3.740	140.90	104.10	2.23
4.0	4.50	36.70	4.40	1.030	4.080	134.60	97.30	2.19
5.4	6.50	52.00	4.45	0.500	3.230	143.90	107.20	2.38
3.6	2.70	35.90	2.22	82.580	3.220	137.20	98.00	2.22
2.9	2.40	55.50	4.58	0.510	4.230	139.20	99.20	2.59
3.0	3.60	52.60	6.06	1.590	4.190	139.70	102.40	2.60
5.8	7.50	64.80	3.40	0.500	3.940	142.90	101.00	2.37
4.3	7.60	65.50	4.79	0.500	3.630	141.60	106.20	2.35
3.1	2.00	41.70	4.73	1.780	4.060	141.10	105.70	2.34
5.5	4.70	31.90	4.16	0.080	4.340	140.20	103.40	2.36
3.1	4.40	53.60	4.10	0.550	3.710	140.40	106.20	2.27
4.2	4.10	44.90	3.04	4.080	3.650	140.80	104.40	2.36
6.0	7.50	54.20	4.81	0.250	3.450	140.70	102.30	2.38
2.8	1.80	47.20	5.61	0.500	3.520	142.50	106.00	2.31
2.7	3.40	60.00	4.60	2.720	4.310	143.40	107.50	2.45
3.5	3.80	67.10	3.78	0.500	4.250	144.30	108.50	2.26
3.1	3.10	33.00	2.37	0.500	3.620	143.90	106.60	2.50
5.0	5.30	60.30	3.53	0.500	3.450	141.00	103.50	2.36
3.6	3.40	51.00	4.40	3.290	3.560	143.10	106.80	2.28
2.5	1.40	56.50	6.02	0.500	3.830	142.90	106.10	2.36
2.7	2.00	49.40	3.62	0.190	3.430	139.80	103.50	2.34
2.0	0.60	26.00	2.06	38.110	#NULL!	#NULL!	#NULL!	#NULL!
3.5	2.90	55.20	3.33	0.500	3.420	142.20	104.30	2.37
4.5	8.00	34.30	4.04	0.500	3.610	142.60	105.90	2.36

2.7	7.30	51.40	4.37	0.520	4.250	142.50	103.70	2.33
2.0	4.60	32.50	8.86	0.800	3.950	137.14	105.42	2.27
6.0	8.00	53.90	3.42	3.110	3.770	143.70	104.50	2.45
1.7	4.80	48.50	4.49	0.500	3.630	138.40	105.08	2.37
4.9	4.30	53.30	6.30	0.500	3.960	140.60	103.20	2.40
2.7	2.90	32.70	3.10	0.500	4.120	142.30	106.90	2.38
4.8	7.00	35.90	5.05	0.500	4.720	144.50	106.80	2.68
4.3	5.00	38.50	4.12	0.500	3.530	143.80	105.30	2.40
2.2	2.00	51.50	3.20	0.500	3.650	139.20	104.70	2.48
4.9	4.80	44.90	3.46	0.500	3.920	140.60	104.50	2.40
2.9	2.30	31.10	3.00	7.600	3.680	137.40	100.30	2.34
1.1	0.10	41.30	5.53	0.500	4.360	138.40	102.90	2.38
3.9	2.90	46.50	6.35	0.500	4.450	144.20	104.10	2.42
2.0	0.50	47.20	2.92	0.500	4.180	141.90	103.50	2.58
3.8	1.90	48.80	3.70	0.500	3.850	141.30	104.00	2.41
2.8	1.60	51.40	5.58	1.400	4.200	142.70	104.80	2.51
5.6	7.10	42.20	4.14	0.500	4.350	143.20	103.40	2.44
2.6	2.30	44.50	5.08	0.500	4.240	141.60	105.00	2.39
5.1	5.90	51.00	4.15	1.700	3.670	142.90	105.40	2.39
3.0	2.90	37.50	3.88	0.120	3.760	140.50	101.60	2.38
2.4	0.50	52.10	2.80	0.420	3.970	144.10	106.80	2.37
3.8	1.80	56.30	4.56	0.500	4.090	140.20	103.40	2.39
3.5	6.00	32.90	3.91	0.500	4.590	145.10	104.70	2.43
2.3	1.80	36.20	2.73	10.800	3.970	138.20	100.80	2.07
3.7	5.20	45.10	2.89	0.500	4.120	143.50	105.70	2.42
4.4	7.10	66.50	2.94	0.500	3.840	143.20	106.10	#NULL!
4.1	4.90	47.70	3.10	0.500	4.320	142.90	106.30	2.36
3.0	1.80	55.90	4.13	0.500	4.260	144.90	105.00	2.37
3.0	3.80	67.80	3.78	27.620	4.070	142.20	104.40	2.32
3.4	1.40	40.10	5.72	6.280	3.740	132.60	93.40	2.58
2.7	2.50	50.00	3.60	0.500	4.760	141.90	103.20	2.54
7.7	2.30	46.40	2.38	20.670	3.330	130.92	97.23	1.84
2.2	3.10	51.70	2.93	1.160	3.620	142.20	105.90	2.34
3.2	2.90	51.30	3.74	43.300	3.530	139.40	100.80	2.33
4.7	7.90	58.60	3.53	0.500	3.860	144.40	106.70	2.47
3.3	4.40	62.00	5.60	0.560	3.900	140.20	104.60	2.38
1.5	2.10	65.90	4.61	1.920	3.520	137.43	102.20	2.10
2.9	3.50	56.70	5.70	0.120	4.130	138.40	100.60	2.38
4.9	6.40	48.00	3.87	0.360	4.040	142.10	104.50	2.26
1.7	3.80	32.90	1.26	43.200	3.080	137.78	102.85	1.91
2.2	0.90	39.60	2.80	0.500	3.710	142.50	108.30	2.39
3.9	3.60	47.30	2.70	0.500	3.910	142.10	109.30	2.40
5.2	5.50	72.80	5.31	0.500	4.080	144.90	111.50	2.35
3.0	3.30	44.60	5.60	0.500	3.690	139.00	104.30	2.54
2.1	6.20	66.30	5.37	0.670	3.480	141.80	105.60	2.20
2.2	1.80	45.80	4.20	0.500	3.550	139.50	103.20	2.49
7.0	6.10	39.30	4.70	94.100	4.860	134.60	101.20	2.33
2.4	2.50	39.90	3.70	#NULL!	3.550	141.20	105.10	2.46
2.3	3.80	44.70	5.48	0.500	4.030	142.00	104.20	2.50
5.1	9.70	57.80	4.68	0.500	3.580	145.90	105.50	#NULL!
3.9	6.10	50.80	3.90	0.500	3.510	140.80	103.90	2.99
1.5	0.40	33.00	3.10	0.500	3.750	138.90	102.30	2.54
5.2	8.40	43.00	2.30	0.500	3.540	140.60	104.90	2.44
2.2	2.80	46.40	4.40	0.500	4.090	139.50	101.10	2.52

4.0	5.00	50.60	5.36	1.400	3.800	142.00	106.20	2.31
2.3	2.30	42.80	1.70	0.210	3.490	140.30	103.50	2.42
5.9	11.60	29.40	5.13	0.500	3.630	142.20	105.00	2.65
3.0	3.20	61.70	6.80	0.500	4.280	140.00	102.90	2.49
2.2	1.90	38.90	5.82	0.050	3.880	141.60	108.70	2.50
1.6	0.80	54.20	2.51	#NULL!	3.480	143.70	109.70	#NULL!
2.0	0.90	101.30	8.80	0.500	3.700	139.50	108.90	2.14
4.7	7.00	55.80	3.98	1.100	4.260	141.30	106.70	2.48
7.2	24.10	172.00	6.36	2.020	1.900	126.68	85.78	2.53
2.7	2.50	57.30	6.60	20.400	4.380	140.80	104.90	#NULL!
7.4	8.60	31.70	5.35	0.500	4.300	140.70	107.70	2.53
4.5	6.80	45.20	2.80	0.500	3.840	143.40	105.60	2.47
4.1	4.40	44.20	5.80	0.300	3.930	142.00	104.30	2.54
4.5	46.80	83.60	12.69	0.500	3.020	142.30	114.00	2.09
2.7	3.60	38.20	3.61	0.500	3.810	141.60	108.30	2.52
6.3	8.70	51.90	4.87	0.500	3.690	140.80	103.70	2.52
4.1	3.50	67.60	3.42	0.500	3.560	145.00	111.40	2.30
3.0	3.20	34.50	3.09	#NULL!	3.190	147.60	113.00	2.33
5.4	8.00	74.30	1.69	0.500	3.090	142.70	108.00	2.30
1.4	1.40	38.90	6.66	0.500	3.860	140.10	106.60	2.49
3.4	3.70	51.00	5.40	#NULL!	4.120	141.40	106.70	2.41
4.4	4.50	64.30	2.12	#NULL!	3.230	139.11	104.60	2.30
1.9	0.90	46.60	5.20	#NULL!	3.790	141.10	105.30	2.33
8.0	9.20	61.10	3.54	#NULL!	3.880	144.50	108.90	2.29
4.7	4.60	50.10	3.68	#NULL!	4.110	142.70	103.60	2.45
6.3	8.50	58.30	2.51	#NULL!	3.610	146.00	108.20	2.36
3.7	2.80	39.50	4.46	#NULL!	4.470	138.70	99.50	2.63
7.9	15.20	43.00	6.77	#NULL!	3.580	146.80	110.20	2.41
3.4	1.30	47.20	5.86	#NULL!	3.850	142.80	105.40	2.43
5.5	6.40	54.90	2.38	#NULL!	3.710	144.90	108.80	2.40
7.5	7.20	50.70	3.98	#NULL!	3.760	142.30	106.10	2.30
5.2	6.50	61.20	3.42	0.280	3.850	143.90	107.00	2.50
3.7	3.10	54.90	3.61	#NULL!	4.030	143.00	108.70	2.33
4.1	4.40	57.70	4.76	3.140	4.270	144.90	110.30	#NULL!
3.4	3.20	56.30	3.91	#NULL!	4.040	142.00	105.80	2.60
5.7	6.70	71.00	4.96	0.530	3.640	144.60	107.00	2.30
4.1	4.50	50.40	3.66	#NULL!	3.920	144.40	108.40	2.63
6.2	8.10	59.10	5.64	#NULL!	3.870	143.10	107.80	2.37
3.0	2.80	49.90	4.10	#NULL!	3.710	141.50	100.70	2.47
3.4	6.20	45.80	3.12	0.500	3.970	144.00	105.50	2.52
4.2	5.10	58.80	4.04	#NULL!	4.100	143.30	107.40	2.38
3.3	2.20	47.60	3.30	0.700	3.930	142.00	106.50	2.41
5.8	7.10	45.50	4.08	#NULL!	3.670	142.10	106.20	2.42
2.6	3.30	52.10	2.84	#NULL!	3.730	143.30	106.90	2.37
2.1	2.80	60.50	3.39	46.200	3.830	141.30	105.70	2.35
5.2	6.60	73.60	5.12	0.280	3.630	143.24	107.27	#NULL!
4.4	5.20	62.90	4.52	#NULL!	3.550	143.40	105.70	2.38
1.8	7.50	82.20	4.10	0.490	3.900	143.10	106.70	2.13
3.5	4.80	48.80	5.18	#NULL!	4.170	142.60	106.50	2.54
1.4	0.40	44.10	2.30	#NULL!	3.160	146.20	112.40	2.28
2.5	1.90	40.20	2.67	1.390	3.730	141.30	106.70	2.35
2.7	2.40	45.40	5.81	#NULL!	4.420	143.40	106.00	2.52
2.4	3.00	51.60	5.15	#NULL!	4.270	140.40	104.40	2.43
5.3	6.30	47.10	5.13	12.000	4.590	145.60	105.10	2.57

4.0	4.40	44.70	3.96	#NULL!	3.940	143.00	105.10	2.51
4.5	5.50	49.50	3.49	#NULL!	4.150	146.20	104.90	2.54
3.1	3.40	42.70	3.43	#NULL!	3.950	143.70	109.30	2.30
2.9	2.00	44.40	5.52	0.070	4.070	142.20	105.70	2.45
5.6	5.30	46.10	2.04	#NULL!	3.180	144.90	109.00	2.31
7.9	11.60	49.90	4.95	#NULL!	3.790	142.20	104.60	2.54
5.0	7.10	53.00	3.66	#NULL!	3.510	141.20	100.00	2.39
7.1	10.10	51.80	3.43	#NULL!	3.710	146.30	110.80	2.45
8.8	7.60	83.60	5.91	110.100	4.190	145.00	108.00	2.44
2.9	7.80	52.60	2.57	77.540	3.200	138.51	103.10	2.22
3.4	5.40	54.30	4.08	#NULL!	4.010	142.50	105.10	2.43
3.2	4.30	56.80	6.41	#NULL!	4.200	144.60	106.20	2.56
3.0	2.40	39.20	4.65	#NULL!	3.610	144.90	106.70	2.52
2.9	4.40	34.10	2.73	2.980	3.950	142.50	105.10	2.56
0.8	3.30	64.70	8.05	#NULL!	3.980	142.10	105.90	2.37
1.1	0.90	30.60	3.92	#NULL!	4.010	141.50	106.10	2.47
5.1	5.50	53.70	4.61	#NULL!	3.970	143.40	106.00	2.48
3.0	3.30	45.10	5.77	#NULL!	3.820	142.10	104.70	2.52
1.8	1.30	33.90	5.30	0.500	3.870	139.50	102.20	2.26
0.9	1.10	33.20	8.00	#NULL!	3.940	140.10	105.10	2.38
9.9	17.90	57.90	4.00	#NULL!	3.820	138.80	103.00	2.31
5.7	7.00	72.20	5.37	#NULL!	3.800	143.70	108.60	2.36
5.0	6.30	67.10	4.77	#NULL!	4.230	145.60	107.70	2.46
3.8	5.30	44.40	3.91	2.600	3.870	141.20	104.00	2.47
13.9	2.60	43.20	6.92	1.000	3.620	142.88	112.37	1.98
3.0	4.40	32.30	4.36	16.200	3.690	144.60	107.60	2.22
4.7	6.90	61.90	4.83	0.500	4.040	143.10	105.40	2.50
6.2	7.60	52.60	2.11	7.420	3.420	142.60	107.60	2.33
1.4	0.60	39.60	3.88	0.500	4.140	145.20	108.80	2.36
4.0	7.50	43.00	3.95	#NULL!	3.580	145.50	109.40	2.37
4.5	6.10	46.80	4.05	#NULL!	4.020	144.40	105.70	#NULL!
26.1	32.20	57.10	3.05	#NULL!	3.730	144.00	104.80	#NULL!
3.4	3.00	43.70	5.12	0.600	3.630	142.40	105.30	2.45
5.2	6.40	54.70	3.36	#NULL!	4.000	142.50	105.60	2.56
1.8	3.90	50.60	4.66	0.420	3.590	140.91	104.26	2.10
7.8	13.20	41.60	3.41	#NULL!	4.190	142.20	106.00	2.39
5.3	8.00	45.70	4.70	#NULL!	3.210	140.10	102.90	2.24
2.6	2.60	52.30	4.77	1.900	3.880	144.30	106.90	2.45
5.3	4.90	59.10	4.85	0.500	3.770	144.40	108.40	2.41
5.1	5.90	31.80	3.23	#NULL!	4.170	142.30	106.20	2.61
0.1	1.10	35.80	9.50	1.700	3.800	139.55	104.85	#NULL!
5.2	6.20	57.60	3.02	#NULL!	3.990	142.80	107.00	2.57
3.7	2.70	48.60	3.95	2.100	#NULL!	#NULL!	#NULL!	#NULL!
4.1	5.90	52.10	4.01	0.560	4.270	140.70	105.90	2.61
2.4	2.30	40.70	2.60	1.000	3.890	139.00	105.90	2.31
4.1	3.20	45.70	3.19	1.200	3.450	145.90	107.50	2.52
4.6	4.00	43.70	3.38	1.500	3.920	147.00	109.90	2.38
4.5	4.60	47.10	2.48	1.900	4.030	144.50	106.00	2.33
3.6	4.70	55.70	4.10	#NULL!	3.700	148.10	109.00	2.39
3.1	5.90	51.60	3.96	0.800	3.920	148.20	111.60	2.26
2.4	0.20	37.60	5.30	#NULL!	4.110	143.10	105.90	2.46
2.9	1.50	37.80	2.57	#NULL!	3.600	144.60	106.10	2.44
6.1	8.40	46.10	5.30	#NULL!	3.890	143.70	105.00	2.49
3.2	4.00	54.70	5.21	#NULL!	3.980	144.10	104.80	2.43

5.6	5.90	66.70	3.37	#NULL!	4.250	146.90	109.70	2.37
7.6	9.70	74.60	4.40	#NULL!	3.830	145.40	107.00	#NULL!
3.4	5.50	67.80	5.55	#NULL!	4.160	143.70	108.00	2.32
6.2	9.20	50.20	2.85	#NULL!	3.510	143.70	103.90	2.49
5.2	9.40	49.90	2.01	#NULL!	3.730	142.50	105.70	2.36
6.4	5.40	55.00	4.50	#NULL!	3.790	141.40	103.10	2.40
3.3	4.10	61.90	3.80	#NULL!	3.810	139.50	100.20	2.47
1.2	4.70	54.00	5.51	0.950	4.000	137.45	103.22	2.43
3.7	4.90	55.00	3.90	#NULL!	3.610	141.20	104.00	2.33
2.0	2.00	42.50	3.30	#NULL!	3.990	139.00	103.60	2.38
3.4	5.10	47.50	3.53	#NULL!	4.270	144.10	108.80	2.29
4.0	4.60	34.10	3.40	#NULL!	3.800	146.10	107.00	2.45
4.8	5.90	51.70	3.49	#NULL!	4.450	144.90	106.60	2.43
3.3	4.40	58.90	4.45	#NULL!	4.320	144.10	105.60	2.41
3.1	3.20	44.10	2.72	#NULL!	4.220	141.70	106.30	2.38
6.3	9.20	52.00	1.71	0.500	3.890	144.10	106.90	2.25
6.9	11.80	52.40	6.15	#NULL!	3.890	143.90	106.80	2.39
6.5	6.00	54.00	5.40	#NULL!	4.380	143.20	109.50	2.19
3.0	2.80	71.30	5.56	7.000	4.230	141.70	105.20	2.44
3.1	2.70	67.90	3.28	0.500	4.110	142.90	107.50	2.38
3.6	3.60	56.50	2.43	#NULL!	4.380	140.60	106.90	2.40
2.3	0.90	45.80	2.04	#NULL!	3.860	139.30	102.70	2.53
4.9	6.50	40.30	2.02	#NULL!	4.070	136.60	98.40	2.22
2.5	1.90	41.40	2.00	#NULL!	3.980	140.80	108.20	2.29
2.2	1.60	42.40	3.64	0.200	4.180	142.80	107.00	2.41
2.4	2.30	37.30	3.45	3.300	3.810	141.60	107.10	2.43
2.0	7.90	43.80	6.72	#NULL!	4.280	140.10	101.80	2.30
3.0	4.40	46.10	4.86	#NULL!	4.010	141.60	107.80	2.43
1.5	5.10	34.90	3.32	#NULL!	3.690	138.90	105.70	2.42
4.3	4.60	35.30	2.43	#NULL!	4.060	144.50	109.40	2.27
3.2	3.50	47.80	4.12	#NULL!	3.870	141.50	106.20	2.44
1.6	0.80	46.10	4.94	0.500	4.180	143.50	113.00	2.35
2.4	1.60	42.70	3.32	27.200	3.990	142.20	106.50	2.31
5.2	9.20	51.60	4.30	0.500	3.600	140.80	102.90	2.41
2.5	2.10	35.90	3.76	#NULL!	3.930	140.50	107.10	2.35
3.1	2.40	30.90	3.62	4.900	4.130	140.60	108.90	2.46
3.8	5.50	39.30	4.39	#NULL!	4.060	141.90	106.80	2.44
2.5	2.50	49.50	3.40	#NULL!	#NULL!	#NULL!	#NULL!	#NULL!
2.9	2.90	43.40	6.59	#NULL!	3.950	142.10	105.90	2.53
4.3	5.10	42.70	3.58	#NULL!	3.620	142.00	107.60	2.37
2.1	1.40	45.80	3.30	#NULL!	3.980	143.20	109.80	2.34
7.0	10.70	29.50	2.40	4.200	4.000	142.30	108.80	2.29
2.8	3.40	78.30	7.01	#NULL!	5.250	141.90	106.90	2.42
2.7	4.30	48.50	4.42	0.500	4.290	140.80	104.90	2.31
1.6	3.00	42.40	2.93	4.100	3.840	138.70	105.20	2.44
2.4	3.40	37.00	3.73	#NULL!	4.040	143.20	109.50	2.42
2.0	2.50	43.00	6.17	#NULL!	4.180	142.30	108.60	2.37
5.5	4.50	47.90	5.25	#NULL!	3.860	142.51	105.81	#NULL!
5.8	8.30	77.00	2.60	0.980	4.050	142.20	109.10	#NULL!
3.7	5.90	72.20	5.50	47.500	3.890	137.10	100.40	2.27
9.1	15.60	43.40	7.09	1.270	4.320	136.40	101.70	2.49
2.5	3.20	40.70	2.84	#NULL!	4.460	139.90	103.40	2.54
2.9	4.30	53.60	5.81	0.280	4.060	140.80	107.70	2.35
3.2	3.50	44.80	4.24	0.500	3.860	139.80	105.30	2.37

1.5	1.70	63.40	4.68	#NULL!	4.260	139.90	104.80	2.33
2.9	2.60	50.90	2.93	1.200	3.930	141.40	103.30	2.40
2.9	3.60	36.30	2.57	#NULL!	3.670	141.40	106.60	2.34
4.0	4.30	41.90	1.50	#NULL!	3.920	140.10	102.10	2.24
3.4	5.70	38.70	5.05	#NULL!	4.790	140.00	105.80	2.57
3.7	4.80	43.50	4.33	#NULL!	3.410	141.10	107.70	2.40
4.3	5.90	54.90	3.14	#NULL!	4.430	140.50	105.10	2.52
3.2	4.50	61.70	5.69	#NULL!	4.340	141.80	105.40	2.41
2.9	5.80	114.90	6.20	#NULL!	3.800	140.52	104.54	2.46
2.8	3.80	52.90	4.70	1.370	3.660	144.40	109.30	2.37
2.7	2.70	47.50	5.26	#NULL!	4.700	141.70	104.40	2.53
2.4	2.70	50.20	4.27	#NULL!	4.080	144.30	107.00	2.30
3.9	5.40	58.70	5.04	0.480	4.020	144.10	110.60	2.33
2.8	2.80	54.60	3.15	#NULL!	4.330	147.90	111.00	2.45
5.0	8.00	40.40	2.70	#NULL!	3.820	136.70	105.10	2.48
2.6	3.50	50.10	4.10	#NULL!	3.620	137.40	106.20	2.23
5.7	8.00	54.80	4.06	#NULL!	4.260	144.80	111.60	2.37
5.2	7.00	46.70	4.87	0.760	4.230	145.20	106.20	2.44
5.7	5.20	47.00	3.81	1.030	3.780	144.90	105.00	2.50
2.4	1.70	44.70	6.35	#NULL!	3.590	147.00	110.90	2.34
1.2	0.10	57.40	3.52	#NULL!	3.770	143.70	110.20	2.26
9.3	10.90	55.80	4.11	#NULL!	4.100	145.90	108.30	2.47
4.6	5.30	60.00	4.37	#NULL!	3.820	140.14	102.50	2.49
3.7	8.60	69.60	3.44	9.780	3.520	136.97	102.65	2.52
6.9	11.60	60.30	4.27	#NULL!	3.960	147.40	107.40	2.50
3.8	3.10	58.80	6.01	#NULL!	4.130	144.10	107.10	2.44
8.9	6.60	64.40	3.66	#NULL!	3.640	143.50	108.40	2.23
5.6	6.10	54.10	4.50	#NULL!	4.020	141.80	108.90	2.49
5.7	5.50	44.90	4.90	#NULL!	4.310	144.60	110.80	2.48
2.6	2.40	39.90	4.80	#NULL!	4.000	140.00	106.20	2.40
3.1	7.10	64.80	4.87	0.820	#NULL!	#NULL!	#NULL!	2.54
2.6	1.50	50.00	4.30	#NULL!	3.650	141.70	109.00	2.36
3.4	5.80	64.20	4.51	0.780	4.220	146.40	111.00	#NULL!
2.8	2.70	50.20	4.77	#NULL!	3.830	144.30	109.00	2.30
2.9	2.90	76.90	4.01	#NULL!	3.650	146.70	109.20	2.31
1.7	1.40	43.20	5.31	0.520	4.110	143.30	107.40	2.46
2.0	1.40	55.10	3.97	1.000	3.870	144.50	108.60	2.36
3.4	4.10	42.50	3.22	50.000	4.160	140.90	102.90	2.45
2.3	2.10	46.00	3.95	#NULL!	3.930	146.50	112.30	2.43
3.4	6.10	63.00	5.30	0.360	3.690	144.60	107.70	#NULL!
3.7	5.20	59.00	2.78	#NULL!	4.010	146.50	107.80	2.36
4.8	8.90	70.30	4.65	105.710	4.370	134.80	95.32	2.50
1.8	1.50	43.30	1.89	4.800	4.110	146.60	107.80	2.50
4.5	6.80	67.80	2.52	#NULL!	3.540	145.70	107.70	2.36
3.3	2.30	67.10	6.38	3.800	4.260	147.70	109.60	2.36
3.5	4.30	49.70	2.67	#NULL!	3.830	146.30	106.30	2.48
4.2	6.60	50.70	3.75	#NULL!	3.960	145.70	108.20	2.34
2.8	2.20	45.20	3.68	#NULL!	4.260	146.00	107.80	2.55
3.5	4.20	62.30	3.22	#NULL!	3.270	146.80	108.60	2.35
24.4	3.50	98.30	6.05	258.120	4.020	135.90	102.00	2.51
3.9	3.80	51.30	4.98	#NULL!	4.440	147.90	108.50	2.42
3.3	4.40	72.70	3.97	#NULL!	4.370	146.80	110.50	2.41
2.9	2.40	37.60	2.33	#NULL!	3.620	145.70	108.00	2.48
3.8	4.20	39.30	3.24	#NULL!	3.520	145.50	108.80	2.46

4.4	4.90	49.60	3.35	0.910	3.910	147.70	106.80	2.47
1.9	1.40	42.90	3.71	#NULL!	4.340	144.80	106.70	2.38
2.4	3.10	56.60	3.86	#NULL!	3.680	145.40	109.10	2.38
4.2	6.50	69.30	4.18	#NULL!	4.550	146.90	109.20	2.42
5.0	7.10	42.40	2.60	#NULL!	3.660	146.80	111.70	2.44
3.1	6.70	51.60	3.77	0.160	3.630	144.30	107.70	2.42
4.7	8.60	50.60	3.97	#NULL!	3.840	146.10	104.50	2.50
3.0	2.60	65.80	4.62	#NULL!	4.040	150.60	109.60	2.47
3.4	3.90	48.40	4.30	#NULL!	3.910	142.00	107.20	2.31
2.1	2.00	31.60	4.06	#NULL!	4.050	145.90	110.40	2.33
2.1	2.40	55.30	3.67	#NULL!	3.800	146.40	109.80	2.32
3.7	5.20	40.70	5.86	#NULL!	4.170	144.40	106.30	2.44
4.5	5.10	55.00	3.52	#NULL!	3.860	145.30	108.60	2.40
6.2	6.30	53.70	4.42	#NULL!	4.210	148.00	110.00	2.46
7.3	11.90	50.30	6.14	2.540	3.680	146.10	104.90	2.60
3.6	4.20	38.00	3.32	#NULL!	3.650	145.20	107.80	2.25
4.0	6.90	48.90	3.85	0.410	3.770	147.10	107.70	2.42
2.3	3.50	38.30	3.30	0.200	4.170	144.80	104.50	2.38
6.1	10.80	59.20	3.50	#NULL!	3.800	144.20	106.60	2.40
4.1	7.70	50.20	2.81	#NULL!	3.570	144.60	103.90	2.48
4.0	6.80	42.50	4.53	#NULL!	3.740	144.30	107.60	2.39
3.9	4.50	46.50	4.17	#NULL!	4.000	142.60	106.00	2.45
5.2	6.30	55.50	3.93	24.400	3.930	144.80	105.40	2.52
4.7	9.10	50.50	5.20	0.180	3.900	142.00	106.40	2.44
9.4	14.50	58.60	5.80	0.310	3.430	146.60	110.50	2.53
3.7	11.40	64.00	4.31	0.620	3.440	140.82	99.22	2.52
7.7	13.20	54.90	3.27	#NULL!	3.930	145.30	109.70	2.25
2.3	3.40	59.20	3.91	30.700	3.310	145.90	108.80	2.24
2.9	4.60	52.20	3.43	#NULL!	3.750	144.00	107.90	2.36
3.1	4.40	43.50	4.14	#NULL!	3.930	142.70	107.50	2.35
2.5	2.20	44.50	2.12	60.100	3.500	143.40	108.10	1.91
6.9	9.10	54.20	4.42	0.300	3.700	144.70	108.20	2.37
3.6	4.60	41.80	3.00	2.400	4.230	143.40	110.00	2.43
2.7	2.20	49.40	3.87	#NULL!	3.950	146.60	109.20	2.46
2.6	3.50	46.70	4.73	0.690	4.250	145.40	108.80	2.45
3.9	3.50	47.40	3.93	4.250	3.750	144.70	107.60	2.35
4.9	5.90	38.50	3.53	0.600	3.820	145.50	107.40	2.40
3.2	3.30	40.00	3.97	0.500	3.640	145.10	108.30	2.40
#NULL!	4.10	#NULL!	#NULL!	0.010	3.420	142.20	108.20	2.35
3.9	16.00	43.00	4.00	0.220	4.310	140.70	104.60	2.37
2.8	2.80	55.80	5.41	2.600	3.790	143.50	106.00	2.46
4.9	5.00	51.50	6.00	#NULL!	4.150	143.40	108.60	2.26
2.2	2.40	41.00	3.06	#NULL!	3.970	144.00	109.10	#NULL!
4.0	5.00	#NULL!	#NULL!	#NULL!	4.230	147.00	107.70	#NULL!
3.7	3.20	42.40	6.98	#NULL!	3.570	144.90	110.50	2.30
4.4	5.10	49.40	4.68	3.300	4.010	142.60	104.00	2.57
4.9	5.40	49.20	5.09	6.600	4.090	144.90	107.40	2.42
3.7	5.00	55.90	2.47	#NULL!	3.870	144.40	108.40	2.32
8.0	9.80	49.70	4.32	3.300	3.860	145.80	106.30	#NULL!
5.9	10.40	53.40	3.00	#NULL!	3.570	144.60	106.90	2.40
3.3	3.90	49.40	3.70	#NULL!	4.040	144.30	106.60	2.53
2.7	3.30	54.40	5.30	0.360	3.780	142.80	109.20	2.20
3.5	3.30	47.70	7.62	#NULL!	3.860	142.20	104.10	2.51
1.5	1.90	39.60	1.91	#NULL!	3.630	148.00	109.10	2.35

2.5	2.50	56.10	2.14	#NULL!	3.920	143.90	105.00	2.40
#NULL!	13.20	#NULL!	#NULL!	#NULL!	3.930	143.90	106.70	2.44
3.7	4.10	45.00	6.93	#NULL!	4.020	144.10	106.10	2.60
1.5	4.80	61.30	9.28	#NULL!	4.310	136.39	100.77	2.42
2.3	2.20	46.70	3.98	0.040	3.430	146.10	108.20	2.30
9.0	20.50	62.90	4.80	0.600	3.890	142.80	103.40	2.37
2.7	3.60	35.60	4.65	#NULL!	#NULL!	#NULL!	#NULL!	#NULL!
2.3	5.50	77.00	6.41	#NULL!	4.020	148.70	109.40	2.51
4.9	7.00	40.30	3.67	#NULL!	3.560	146.80	109.90	2.35
3.2	3.70	60.80	2.74	10.800	4.360	144.90	110.00	2.31
2.9	2.30	56.80	5.13	#NULL!	3.820	144.40	110.20	2.42
1.8	2.80	41.30	3.94	#NULL!	3.730	145.60	107.40	2.49
2.4	3.20	39.30	2.12	#NULL!	3.750	147.50	109.30	2.37
2.0	2.70	44.00	5.06	#NULL!	3.870	145.60	107.60	2.45
3.2	4.70	41.30	6.88	1.700	4.320	146.10	107.80	2.59
2.2	0.90	51.70	5.89	#NULL!	3.970	147.50	111.80	2.40
3.6	10.30	64.70	4.07	0.060	3.910	146.50	106.70	2.45
2.4	2.30	61.10	8.12	17.100	4.320	144.60	107.70	2.45
2.9	2.60	40.30	2.17	#NULL!	3.850	145.20	112.30	2.21
2.9	3.10	54.40	2.93	#NULL!	4.280	145.40	104.60	2.52
4.9	6.40	64.50	5.84	#NULL!	3.950	146.70	109.50	2.39
6.0	5.10	55.10	3.67	#NULL!	4.210	140.60	106.90	2.45
3.6	3.70	52.70	3.44	#NULL!	3.790	142.20	107.90	2.33
1.7	1.70	54.30	7.10	#NULL!	3.730	138.10	104.40	2.29
2.8	2.90	51.70	4.22	2.400	4.360	138.30	107.20	2.36
4.4	6.60	67.50	3.39	#NULL!	4.200	140.10	104.80	2.46
2.2	1.70	52.10	6.72	#NULL!	4.340	139.60	103.00	2.50
3.8	4.30	33.90	3.48	#NULL!	3.930	141.70	111.10	2.35
2.6	3.40	52.10	5.63	0.700	4.150	146.10	107.60	2.48
6.6	6.40	46.70	3.51	#NULL!	4.390	144.20	107.00	2.46
3.6	3.30	41.30	4.21	#NULL!	4.710	143.20	107.00	2.39
6.7	5.30	38.00	3.59	#NULL!	3.890	145.70	109.90	2.49
6.2	8.30	53.40	5.11	#NULL!	4.100	143.90	108.00	2.57
1.8	1.80	39.80	4.30	1.800	4.110	135.40	100.70	2.37
5.2	6.30	48.40	3.02	#NULL!	4.130	142.70	107.60	2.43
3.1	2.80	37.00	3.05	0.500	4.490	140.30	106.90	2.41
5.4	4.80	50.70	3.52	#NULL!	4.150	142.40	108.90	2.33
1.9	1.40	37.60	3.70	#NULL!	4.970	141.60	107.10	2.40
4.2	7.20	47.60	5.17	0.500	4.080	142.00	106.20	2.34
2.2	2.00	54.40	3.34	#NULL!	#NULL!	#NULL!	#NULL!	#NULL!
2.9	11.30	49.50	2.67	0.500	3.940	138.55	104.67	#NULL!
5.6	5.20	41.90	2.38	0.500	3.830	143.40	108.10	2.45
3.0	2.20	46.30	3.04	0.500	#NULL!	#NULL!	#NULL!	#NULL!
3.9	4.20	71.10	3.29	0.500	3.910	142.80	105.70	2.56
3.0	2.90	42.60	4.57	0.500	3.990	140.40	104.20	2.53
6.9	12.40	43.60	4.64	0.500	3.740	140.60	103.40	2.46
1.4	0.80	34.30	2.62	0.500	4.220	139.80	107.50	2.44
2.8	3.40	57.00	2.64	0.500	3.710	141.10	107.40	2.24
1.5	1.20	42.60	4.01	0.500	3.860	142.40	108.10	2.52
2.5	1.60	40.00	4.20	0.500	4.070	140.00	106.80	2.44
2.6	2.00	53.40	5.36	0.500	3.760	141.80	107.50	2.30
6.2	6.70	31.70	4.05	0.500	3.840	141.90	110.20	2.36
2.5	2.20	56.00	6.74	0.500	4.030	143.00	111.00	2.12
3.3	3.80	66.10	3.05	0.500	4.380	142.10	105.70	2.38

1.4	4.60	49.60	5.37	0.500	3.820	138.84	110.87	1.72
3.0	3.60	38.70	4.14	0.500	3.920	142.60	107.20	2.40
2.5	2.60	32.30	4.85	0.500	#NULL!	#NULL!	#NULL!	#NULL!
7.9	9.80	62.50	4.56	0.500	3.640	140.60	106.10	2.25
3.1	3.10	44.60	3.81	0.500	3.940	141.70	107.80	2.35
2.4	1.70	35.10	5.37	0.500	3.970	139.20	106.30	2.36
2.8	1.30	55.90	3.98	0.500	3.810	143.10	107.30	2.38
10.6	19.10	53.80	4.50	0.500	3.770	140.40	105.40	2.53
6.0	8.20	55.20	5.32	0.500	3.500	142.80	104.70	2.42
6.2	7.50	58.90	4.90	6.110	3.960	142.21	102.68	2.37
5.2	5.00	50.30	5.65	0.500	3.800	140.80	106.30	2.42
5.0	3.90	36.00	2.95	4.100	4.050	137.80	102.90	2.41
6.8	9.80	48.60	3.50	0.500	3.690	141.20	106.50	2.51
2.2	2.40	32.50	3.46	0.500	3.930	141.30	107.10	2.52
1.6	0.90	51.40	5.21	0.500	4.100	142.00	107.30	2.61
2.3	3.00	38.00	1.70	0.500	3.730	143.30	106.60	2.36
2.6	3.70	45.10	3.58	0.500	3.890	142.90	108.60	2.49
2.8	2.40	48.40	5.11	0.500	3.970	141.70	106.00	2.43
1.5	1.50	48.30	6.28	0.500	4.340	141.80	104.50	2.50
3.0	2.90	52.10	3.59	9.900	4.000	141.00	101.90	2.44
1.5	1.70	47.60	7.76	0.500	4.080	142.50	110.00	2.33
5.2	7.30	25.70	3.10	5.100	3.950	133.19	95.39	2.38
109.3	19.40	55.20	1.83	0.500	3.800	143.00	108.20	#NULL!
3.4	3.10	55.50	3.80	0.500	4.310	145.00	109.10	2.35
4.0	4.30	46.00	4.81	0.500	4.230	140.20	104.00	2.52
2.2	3.80	48.40	6.00	0.500	3.960	145.00	106.90	2.34
3.0	4.40	59.90	5.00	15.500	3.890	138.40	106.00	2.27
8.7	16.70	64.70	4.39	0.500	3.840	145.90	108.70	2.39
2.3	1.10	40.90	4.88	0.900	4.020	143.00	111.60	2.35
5.4	5.20	48.70	4.25	0.500	4.180	142.60	105.40	2.48
2.8	2.60	42.60	6.07	0.500	4.450	147.60	110.60	2.41
3.7	4.10	44.60	3.55	0.500	4.420	143.30	106.40	2.57
6.4	12.60	45.70	3.50	0.500	3.750	136.70	101.80	2.65
3.8	4.90	42.10	3.60	0.500	3.630	137.90	103.50	2.58
30.3	8.50	49.10	3.03	1.000	3.910	141.90	105.90	#NULL!
2.7	3.20	44.80	4.30	0.500	3.710	143.40	108.50	2.44
5.6	6.30	46.60	3.82	0.500	4.150	143.80	105.20	2.53
3.1	3.00	60.30	5.03	0.500	3.990	142.10	105.30	2.54
3.9	5.20	46.60	4.95	0.500	3.830	142.50	106.40	2.51
4.9	3.40	41.20	2.05	1.100	3.910	141.90	106.60	2.33
2.6	4.10	38.30	3.56	0.500	4.160	140.90	102.90	2.46
2.2	2.40	41.50	3.90	0.500	3.760	144.90	109.60	2.42
4.9	10.20	48.70	3.30	0.500	3.950	139.40	105.90	2.50
2.1	8.50	39.80	6.00	16.100	3.790	136.26	101.36	2.38
3.4	4.30	48.90	4.79	0.500	4.030	143.90	109.60	2.58
2.1	1.10	43.70	7.12	1.400	3.830	140.80	107.50	2.51
4.6	4.30	52.90	3.64	0.500	3.720	144.80	107.20	2.45
2.9	4.30	48.50	4.26	0.200	3.370	145.10	109.20	2.40
3.3	4.50	40.20	3.86	0.500	3.440	144.10	107.90	2.27
5.8	10.30	37.30	4.52	58.300	4.470	134.30	98.60	2.17
4.6	7.40	43.10	4.35	0.500	4.870	140.70	105.70	2.56
5.5	8.50	41.90	1.70	0.500	3.940	133.70	101.30	2.44
2.5	2.50	39.50	5.71	0.500	3.680	142.40	106.90	2.45
6.4	14.70	40.90	5.41	0.500	3.830	142.10	107.40	2.42

10.2	15.30	40.20	3.11	0.500	4.030	140.90	106.10	2.61
7.9	16.40	49.60	4.51	0.500	3.980	144.00	107.30	2.48
6.0	8.10	49.50	3.37	0.500	3.900	143.10	106.40	2.56
2.4	3.00	45.60	4.38	0.500	3.870	142.20	106.50	2.41
3.2	5.40	52.00	4.70	0.500	3.620	145.50	106.50	2.55
3.0	4.90	60.40	4.45	84.800	3.950	141.60	106.50	2.52
3.8	6.10	42.90	4.75	0.500	3.980	141.00	106.00	2.42
1.7	0.70	58.20	5.34	0.500	3.930	144.00	107.80	2.38
4.2	5.20	51.70	4.05	0.500	3.840	143.30	107.50	2.48
4.8	4.20	62.10	4.55	0.500	4.240	143.10	109.40	2.54
2.3	3.00	42.20	3.30	0.500	4.080	140.60	106.70	2.45
4.1	3.70	58.70	3.71	0.500	3.500	143.50	108.50	2.37
5.4	9.30	61.70	4.50	0.500	4.040	139.90	106.60	2.40
6.5	10.20	72.90	5.48	0.500	4.200	143.60	108.20	2.47
4.8	5.30	59.50	4.45	0.500	3.920	144.30	107.70	2.42
9.9	12.70	51.80	4.21	0.500	3.870	143.70	107.20	2.47
4.2	5.30	65.70	4.37	0.500	3.700	143.00	107.40	2.41
2.9	1.10	66.80	6.67	0.500	4.110	143.60	104.80	2.45
3.0	4.30	21.00	1.99	0.700	3.750	135.00	104.50	2.16
2.3	3.20	47.60	3.91	1.540	4.070	142.60	108.50	2.42
3.2	4.50	47.20	5.54	0.600	3.720	143.50	109.80	2.51
5.3	5.20	42.70	3.96	34.300	3.530	139.60	97.50	2.47
1.9	1.40	49.30	6.57	0.500	4.460	143.80	111.00	2.54
2.8	3.60	52.80	3.34	0.500	3.680	143.40	107.70	2.53
4.9	5.80	58.10	3.68	0.500	3.800	144.50	108.00	2.52
3.7	5.30	56.70	5.40	0.500	3.720	144.60	110.40	2.47
1.6	2.40	36.70	4.48	0.500	4.360	143.80	106.60	2.69
4.7	4.40	46.90	7.86	0.500	3.665	142.00	106.00	2.56
3.1	1.70	54.20	4.54	0.500	3.910	143.20	109.20	2.58
7.7	13.90	60.50	4.61	2.600	3.670	142.30	109.20	2.49
2.4	2.80	32.20	3.18	1.500	4.290	142.50	108.70	2.43
1.8	0.70	31.50	4.08	5.600	3.790	143.70	104.30	2.42
3.9	4.00	36.00	6.91	4.000	4.050	145.40	108.30	2.39
2.1	2.00	42.30	3.88	0.500	4.120	144.70	108.10	2.63
5.2	7.80	58.20	3.55	0.500	3.780	142.40	107.40	2.65
4.1	4.60	38.20	4.30	0.500	4.240	138.70	104.60	2.64
2.1	2.90	48.30	5.89	0.500	4.130	142.40	107.30	2.51
2.0	1.30	18.50	3.54	0.500	3.650	139.90	107.60	2.48
2.8	5.10	49.90	2.83	0.500	3.730	142.90	109.00	2.44
3.2	2.40	36.30	4.11	0.500	4.020	142.40	109.50	2.37
1.6	0.90	44.00	3.70	1.000	3.760	143.70	108.10	2.43
5.7	5.30	43.20	3.41	0.500	3.620	144.10	110.70	2.32
2.9	3.20	44.30	4.09	#NULL!	3.520	141.70	107.50	2.39
1.7	0.10	44.00	4.23	0.500	3.660	143.80	108.90	2.37
2.3	2.20	41.00	2.99	#NULL!	3.850	142.00	106.30	2.37
3.5	3.20	43.20	4.30	0.500	3.990	143.70	108.70	2.42
2.9	4.20	43.00	4.52	0.500	3.710	142.50	107.10	2.37
4.1	5.10	43.20	3.24	0.500	3.790	143.20	107.60	2.43
3.3	4.10	49.30	6.81	0.500	4.080	140.30	107.20	2.42
2.0	2.10	45.00	4.46	0.700	4.110	142.00	106.00	2.45
3.6	3.50	38.20	5.91	0.500	3.570	144.00	106.80	2.51
3.7	4.00	53.10	3.80	1.500	4.200	138.80	102.60	2.33
5.4	7.60	56.50	5.23	0.500	4.310	141.80	109.40	2.66
1.9	1.80	46.40	2.94	0.500	3.540	138.42	100.34	2.24

2.8	1.40	55.00	6.69	0.500	3.720	140.40	106.10	2.34
3.4	3.80	38.90	5.62	0.500	3.640	144.30	108.30	2.26
3.1	3.40	40.30	4.71	0.500	3.880	145.70	108.90	2.40
4.1	2.90	38.80	3.72	11.600	3.510	141.70	103.60	2.27
3.0	1.80	64.60	5.10	0.500	3.390	145.20	106.30	2.34
3.0	2.90	57.60	5.85	0.700	4.230	142.40	108.10	2.58
3.3	4.40	55.80	3.53	1.000	4.030	141.60	106.80	2.52
2.9	2.50	49.00	4.41	2.500	4.100	141.40	108.70	2.34
9.0	10.10	55.00	3.65	0.500	3.500	142.10	108.40	2.36
5.2	6.10	60.80	4.14	0.778	4.220	141.70	106.80	2.56
3.2	4.60	46.40	4.00	0.600	4.360	140.80	105.80	2.49
3.4	3.10	43.80	4.52	0.500	4.330	142.80	108.50	2.50
4.0	3.70	62.70	4.08	0.600	3.930	142.80	110.80	2.34
2.7	3.30	60.20	3.17	0.500	3.800	141.60	106.40	2.42
6.2	8.50	54.00	3.89	0.500	3.740	139.90	108.60	2.40
2.7	2.60	53.30	5.88	0.500	4.230	141.60	110.90	2.41
4.2	4.80	59.80	4.77	0.800	4.100	140.10	111.10	2.30
3.9	4.30	56.20	4.78	0.500	4.240	144.10	111.30	2.44
2.3	3.10	35.50	2.96	0.500	3.720	136.71	100.10	#NULL!
1.8	0.60	43.40	5.30	0.500	3.970	136.90	103.00	2.54
4.9	4.40	61.20	3.98	0.600	4.630	142.10	109.60	2.55
3.6	2.40	63.80	4.54	0.700	4.980	142.10	106.80	2.35
2.6	1.50	47.50	4.07	15.000	4.250	138.20	106.00	2.26
3.2	2.50	35.20	2.08	1.700	3.850	130.60	101.10	1.96
3.6	3.10	42.80	4.74	0.500	3.860	142.60	110.70	2.44
2.0	0.90	48.20	5.02	0.700	3.960	140.40	110.50	2.14
5.4	8.80	53.60	3.65	0.500	4.050	140.10	106.80	2.42
2.0	1.60	38.80	2.20	0.500	4.070	141.40	107.30	2.41
4.6	4.10	45.70	3.60	0.500	3.500	143.70	112.10	2.25
2.3	5.60	52.20	4.90	0.500	3.920	135.56	102.03	#NULL!
5.8	6.00	60.50	3.26	4.100	3.810	139.20	107.70	2.27
3.9	2.60	46.70	3.98	0.500	3.930	141.00	107.90	2.29
7.8	6.20	48.40	3.60	1.500	4.120	141.40	106.10	2.49
2.9	1.60	43.80	3.62	0.500	4.040	143.20	112.00	2.58
3.8	1.00	44.80	4.75	8.500	3.620	141.10	108.50	2.32
6.4	7.30	62.60	3.43	0.500	4.260	139.90	111.40	2.36
5.0	6.80	55.30	2.55	0.500	3.570	141.50	109.50	2.44
5.0	4.20	29.90	2.20	28.900	3.980	134.60	103.20	2.05
4.7	6.20	59.80	6.04	0.500	4.120	141.90	108.60	2.41
4.2	1.80	39.50	4.30	2.800	3.960	138.90	102.60	2.43
4.3	6.40	56.20	2.25	0.500	4.170	138.00	108.00	2.57
2.0	5.80	56.30	7.84	0.500	3.480	136.92	100.03	2.16
2.2	1.70	42.80	5.09	0.500	4.080	141.50	109.80	2.46
4.1	2.90	55.90	8.70	0.500	3.550	142.00	108.90	2.47
7.3	6.00	59.60	3.11	0.500	4.410	141.00	108.40	2.49
3.4	2.80	48.80	4.28	4.400	4.220	141.20	106.40	2.52
3.1	2.40	53.10	3.97	0.500	3.670	141.10	109.40	2.54
3.7	2.50	48.40	1.61	0.500	3.500	141.20	110.80	2.38
3.8	4.80	55.00	5.06	3.400	3.940	142.70	109.00	2.59
4.4	3.80	57.60	4.16	0.500	4.660	144.60	111.40	2.51
4.3	5.60	59.70	2.10	0.500	3.420	141.30	104.00	2.35
4.4	4.50	70.30	5.60	0.600	3.980	140.40	107.10	2.52
2.6	1.70	55.30	6.44	0.500	4.200	141.00	107.70	2.63
10.4	13.80	51.70	3.92	0.500	3.900	143.30	112.00	2.37

7.4	9.60	46.20	2.65	0.500	3.860	142.70	111.40	2.42
3.5	3.60	68.60	4.00	0.500	4.310	144.40	109.30	2.58
5.4	4.30	45.60	3.99	0.500	4.050	142.70	109.60	2.51
2.8	0.60	38.50	5.12	0.500	3.520	142.20	111.50	2.28
2.8	2.20	43.80	5.76	0.500	4.120	142.00	108.10	2.51
4.9	7.10	54.50	3.64	0.500	4.280	142.70	107.20	2.67
2.7	2.70	47.70	3.50	0.500	3.950	142.40	108.00	2.41
4.6	6.00	45.60	3.76	0.500	3.860	141.70	105.20	2.59
6.5	6.90	66.50	4.87	1.900	3.580	143.90	107.20	2.56
3.6	4.80	44.90	2.94	1.400	4.860	143.60	106.00	2.74
3.6	4.20	43.10	5.89	0.500	4.480	142.40	107.70	2.45
6.6	8.70	57.40	4.66	0.500	3.880	140.30	105.80	2.34
2.2	3.10	52.90	5.12	0.500	4.060	142.50	104.50	2.24
2.9	2.10	43.80	3.32	17.500	3.580	137.70	100.80	2.51
5.8	12.20	44.70	3.32	2.800	4.650	138.70	104.60	2.24
3.9	4.10	48.80	3.14	0.500	4.040	140.90	107.00	2.39
3.9	4.90	46.10	4.12	0.500	4.840	143.60	107.40	2.24
3.0	3.70	36.80	8.35	0.500	4.390	138.70	105.60	2.42
5.1	4.40	46.10	4.54	9.400	4.210	140.70	105.80	2.24
4.5	6.60	36.80	5.11	8.500	4.380	139.80	102.70	2.55
3.1	2.20	46.40	7.29	0.500	4.350	140.10	106.90	2.38
3.1	5.80	45.80	4.10	27.500	4.450	133.10	98.60	2.32
1.8	0.60	60.90	3.85	0.500	4.570	142.60	107.90	2.28
1.7	1.30	71.50	2.96	20.260	3.340	140.76	106.58	1.86
1.0	1.30	59.60	7.41	1.300	3.480	136.87	106.53	2.16
3.1	2.20	32.70	1.97	0.500	3.840	140.60	107.80	2.17
3.7	4.20	48.30	6.16	0.500	4.530	140.40	106.80	2.31
3.4	3.20	38.80	3.73	0.500	4.530	139.40	106.60	2.43
3.8	4.10	43.40	4.67	0.500	4.580	144.80	112.80	2.38
4.4	5.40	50.80	3.80	2.700	3.680	142.40	108.40	2.19
2.1	2.50	49.70	4.51	0.600	4.230	142.60	107.00	2.35
5.9	6.60	58.10	5.72	25.600	3.920	135.80	99.50	1.86
2.1	2.20	32.60	4.01	1.300	4.500	139.10	105.40	2.40
3.5	4.60	46.80	4.43	5.600	3.860	143.90	104.60	2.50
5.8	8.40	36.70	4.20	0.500	4.070	134.70	101.10	2.41
2.6	2.70	61.40	6.78	1.500	3.900	144.90	106.10	2.29
1.1	2.90	50.90	4.72	7.240	4.470	137.82	108.26	2.44
2.7	5.40	47.79	4.01	8.200	3.610	138.33	108.60	2.43
2.2	3.30	40.50	5.12	0.500	4.570	139.30	107.20	2.49
4.4	5.40	50.90	5.26	0.500	3.980	141.40	105.80	2.37
3.7	4.70	47.40	5.24	0.500	4.220	141.90	110.10	2.43
5.3	10.30	69.80	3.98	0.500	4.550	140.70	104.40	2.33
5.3	5.30	57.50	4.06	0.500	4.190	140.50	107.80	2.34
2.3	1.90	43.90	5.71	0.500	3.850	141.50	107.70	2.34
3.6	3.90	50.00	2.40	0.500	3.960	140.00	105.00	2.52
4.9	8.70	41.66	5.00	7.300	4.170	140.87	105.96	2.44
3.6	4.40	53.00	2.82	0.500	3.870	141.40	108.10	2.32
5.3	4.60	69.10	5.33	0.500	3.410	144.60	107.40	2.30
2.9	2.40	49.20	3.70	0.500	4.380	142.20	108.50	2.34
4.4	3.80	63.80	4.73	0.500	4.010	144.50	110.00	2.42
3.6	5.20	64.20	6.96	0.500	4.110	141.50	105.90	2.35
3.6	3.50	49.90	4.33	0.500	4.180	143.60	109.00	2.31
3.0	3.70	55.50	3.81	0.500	4.370	142.20	105.90	2.41
1.6	4.50	36.77	11.10	1.200	3.830	141.11	110.82	2.20

2.9	2.30	46.90	4.50	1.000	4.060	140.30	104.00	2.47
3.1	3.90	51.00	4.32	0.500	3.970	142.30	109.10	2.44
7.0	8.40	61.10	2.58	82.800	3.590	139.60	108.20	2.27
4.2	4.20	38.10	3.26	0.700	3.940	140.80	109.80	2.33
1.9	1.40	44.70	3.78	0.500	4.190	139.50	107.20	2.36
1.7	2.60	45.40	4.25	0.500	4.530	140.10	108.80	2.43
3.4	3.20	49.60	2.98	0.500	4.720	139.10	105.00	2.57
0.4	2.50	51.30	3.88	0.500	4.510	139.60	106.10	2.38
5.3	6.90	57.90	3.87	0.500	4.530	140.40	102.90	#NULL!
2.7	2.40	41.00	3.24	0.500	4.110	141.80	109.80	2.57
4.5	4.90	65.60	6.66	0.500	3.790	141.90	108.50	2.35
1.2	0.30	39.60	7.16	0.500	4.270	138.50	107.20	2.44
2.5	1.70	51.60	5.16	0.600	4.320	141.00	106.30	2.33
4.9	5.10	50.00	5.92	6.000	3.570	142.00	107.30	2.42
2.4	2.40	51.00	4.48	0.500	4.160	140.40	108.10	2.43
3.3	4.10	42.00	5.86	0.500	4.270	141.00	106.60	2.50
3.3	4.10	41.00	4.30	0.500	3.750	140.10	107.80	2.50
3.8	1.90	38.00	2.41	0.500	3.700	143.30	109.70	2.47
3.7	1.80	54.20	2.56	0.500	3.730	140.30	108.50	2.37
6.4	6.80	73.00	3.94	0.500	4.370	142.40	105.80	2.47
4.9	5.80	45.20	4.55	0.500	4.340	141.70	106.40	2.35
1.4	0.60	41.60	4.22	1.400	4.400	141.70	110.30	2.33
2.5	6.40	44.35	7.48	0.500	4.010	139.89	107.68	2.45
3.9	4.00	44.60	5.75	0.500	4.020	141.60	108.40	2.43
5.1	5.70	43.60	4.03	0.500	3.960	143.50	107.50	2.53
0.8	0.80	32.30	5.93	2.900	4.420	141.50	106.60	2.46
0.6	0.40	24.20	2.84	1.800	3.440	140.50	107.90	2.07
2.4	1.50	36.50	3.43	0.500	3.770	140.10	106.90	2.36
5.1	4.80	52.80	2.67	5.400	3.780	140.10	105.60	2.29
5.7	1.10	40.40	6.38	4.100	3.810	142.70	108.80	2.48
2.6	0.20	45.60	3.74	0.500	3.780	142.30	106.80	2.48
1.9	2.30	43.00	6.00	0.500	3.660	142.90	104.20	2.30
4.4	8.40	29.73	11.10	81.000	3.110	137.86	104.88	2.30
2.6	2.60	47.10	5.00	0.500	4.050	141.70	104.20	2.48
1.8	2.20	51.90	4.95	0.500	4.200	141.00	104.20	2.33
2.5	1.30	48.80	6.41	0.500	4.110	141.20	106.70	2.35
1.1	4.20	32.93	2.49	0.500	3.200	141.27	106.44	2.29
5.2	6.80	47.40	2.11	0.900	3.880	145.30	109.80	2.27
2.8	0.60	65.40	6.13	0.500	3.930	142.00	106.20	2.45
7.0	6.10	39.30	4.70	94.100	4.860	134.60	101.20	2.33
3.0	3.20	57.90	4.11	0.500	3.860	146.90	111.10	2.34
2.6	2.50	42.60	4.00	0.500	3.780	144.00	104.70	2.39
3.3	2.70	48.40	7.14	0.500	3.610	144.50	108.90	2.44
1.0	3.50	48.33	6.28	0.500	3.570	139.71	105.29	2.57
1.3	4.90	39.24	3.89	0.500	4.140	140.93	108.49	2.37
6.6	8.10	62.90	3.50	0.500	4.500	147.80	108.30	2.44
5.4	5.60	80.90	3.90	0.920	4.180	149.00	110.80	2.35
3.9	6.80	43.60	2.75	0.500	3.560	146.60	108.20	2.45
4.9	4.80	68.30	3.08	0.500	4.130	145.90	105.10	2.61
3.5	5.30	49.40	4.03	0.500	3.820	145.00	109.90	2.36
5.2	6.50	63.90	6.00	0.500	4.480	143.30	105.90	2.44
4.7	5.00	47.40	4.97	0.500	3.760	141.70	106.60	2.25
2.5	1.30	46.70	4.95	7.100	4.400	141.50	104.60	2.40
13.1	21.60	54.40	6.03	0.500	4.360	127.50	101.30	1.84

2.4	2.10	34.50	6.00	0.500	4.540	143.50	109.10	2.44
3.2	4.30	30.40	2.34	20.400	3.740	137.70	99.60	2.36
7.5	7.90	45.70	3.61	0.500	4.280	143.50	108.60	2.49
1.9	0.90	39.60	4.28	0.500	3.960	144.60	108.00	2.47
3.8	4.80	48.00	5.70	0.500	3.830	138.00	104.00	2.53
4.3	4.30	42.00	3.97	0.500	3.780	141.70	107.60	2.49
3.9	4.70	57.50	3.24	0.500	3.700	146.10	109.30	2.36
4.1	3.10	41.60	5.06	0.500	3.600	144.80	108.30	2.36
5.2	4.60	40.80	3.17	0.500	3.780	143.70	108.90	2.34
3.7	8.10	39.75	4.36	17.800	3.690	140.83	105.12	2.29
2.9	3.10	35.50	6.24	0.700	3.780	148.30	107.00	2.40
3.9	2.60	45.00	3.18	0.500	3.450	144.90	107.90	2.32
6.2	7.50	54.80	5.29	0.500	3.990	143.60	107.30	2.31
5.0	3.60	30.40	4.49	0.500	4.000	145.50	109.20	2.42
3.9	5.40	38.90	3.70	14.600	3.820	139.90	100.90	2.43
2.8	1.60	39.90	4.65	0.500	4.230	144.90	107.40	2.35
2.3	1.20	39.60	2.59	0.500	3.300	148.40	109.90	2.32
1.2	6.90	54.40	5.08	4.400	4.480	144.10	102.80	2.45
3.8	6.40	48.10	3.80	0.500	4.200	145.00	108.20	2.39
4.3	3.60	62.80	4.79	1.000	3.550	148.10	106.10	2.35
5.7	13.40	68.80	2.69	0.500	4.490	143.20	103.60	2.45
3.2	2.40	47.40	4.10	0.500	3.960	149.40	106.60	2.61
3.8	3.10	48.80	4.61	0.500	3.660	143.90	106.20	2.28
2.3	1.60	38.60	4.44	0.500	3.570	144.60	105.80	2.51
8.1	5.90	55.40	3.17	1.500	3.670	145.70	108.90	2.37
7.2	8.80	47.40	5.72	0.500	3.740	147.30	108.20	2.54
3.9	2.30	56.70	4.96	5.100	3.630	145.60	106.90	2.31
2.6	1.00	40.80	4.80	0.500	4.250	147.40	110.10	2.29
5.0	5.60	47.30	8.62	0.900	2.790	139.00	89.90	2.53
3.0	2.40	44.90	4.82	0.500	3.660	144.90	107.80	2.30
2.0	1.30	54.00	4.58	0.500	3.860	144.30	108.10	2.28
4.1	4.30	63.20	4.36	0.500	3.670	147.00	105.30	2.64
3.1	2.50	55.20	4.00	0.500	3.870	141.40	104.80	2.38
5.1	5.00	42.60	3.27	0.500	3.540	145.10	106.60	2.39
2.5	1.70	44.90	3.60	0.500	4.050	144.90	105.40	2.47
3.8	4.80	51.00	2.40	0.860	3.550	137.60	104.50	2.38
5.2	5.30	60.80	5.26	0.500	3.460	145.90	105.30	2.47
5.9	7.70	67.70	5.44	0.500	3.350	145.20	103.20	2.45
4.5	11.30	46.70	2.87	0.500	3.610	141.67	104.69	2.41
5.8	7.10	61.50	3.64	0.500	3.960	144.60	106.10	2.37
4.7	4.40	52.50	3.75	0.500	3.780	143.90	103.80	2.37
2.7	1.20	59.50	3.73	18.300	3.670	142.50	104.60	2.33
4.8	4.30	48.00	4.40	#NULL!	4.330	142.50	104.90	2.47
5.7	4.60	79.50	4.60	0.500	3.790	144.50	106.60	2.32
3.8	4.90	39.00	3.33	0.500	3.430	144.40	107.10	2.47
5.0	4.90	68.80	6.91	0.700	3.630	144.70	101.70	2.65
3.9	5.80	42.90	2.75	0.800	4.070	142.00	105.90	2.42
5.5	5.50	68.10	2.39	19.500	3.990	142.10	107.00	2.55
4.1	3.10	49.10	5.45	4.900	4.020	141.10	103.20	2.39
9.7	11.90	46.40	4.73	0.500	4.010	149.60	111.40	2.53
2.2	0.80	52.90	4.80	0.500	4.060	143.60	109.50	2.58
10.0	12.00	59.20	3.90	0.500	4.100	146.10	108.20	2.40
2.8	1.90	52.20	3.69	1.400	3.580	146.20	107.50	2.44
2.7	2.20	45.10	3.04	0.500	3.670	145.00	108.40	2.45

3.4	1.70	62.90	7.02	4.100	3.700	145.00	106.20	2.45
1.7	0.80	44.10	3.14	0.500	3.600	144.50	107.20	2.32
2.4	1.60	59.00	4.40	0.500	3.980	139.00	104.00	2.45
8.6	13.50	48.40	2.30	19.200	4.000	143.40	105.10	2.41
4.7	7.00	57.90	4.83	0.500	3.560	143.60	105.70	2.51
3.3	2.00	44.80	3.55	0.500	3.350	144.50	104.80	2.38
3.8	2.80	41.10	5.06	0.500	3.480	143.80	109.10	2.13
3.4	3.20	38.50	5.47	1.900	3.960	140.00	103.90	2.47
3.6	1.70	50.70	6.98	0.500	4.150	144.40	109.00	2.43
3.3	2.40	55.30	7.11	0.500	3.790	147.10	111.80	2.45
3.5	1.90	43.80	6.36	0.500	4.440	144.90	109.60	2.50
6.0	6.30	68.80	9.15	1.900	4.200	145.40	110.50	2.51
2.9	2.30	40.50	3.61	0.500	3.600	144.70	109.70	2.42
4.3	3.30	68.10	2.50	3.100	3.570	145.60	109.70	2.25
6.1	8.60	82.90	5.03	0.500	3.430	143.30	106.40	2.26
14.9	25.80	46.70	3.80	0.500	#NULL!	#NULL!	#NULL!	#NULL!
2.7	1.40	62.90	3.05	0.500	3.660	142.20	108.50	2.36
3.2	3.00	62.20	7.78	#NULL!	4.140	143.40	109.50	2.49
9.6	9.90	71.10	6.33	0.500	4.090	145.10	109.40	2.33
8.8	14.40	59.90	6.64	0.500	3.400	143.40	104.50	2.49
3.4	4.00	50.00	3.87	0.500	3.940	144.10	108.80	2.47
5.8	6.70	73.20	3.80	13.210	3.320	142.02	110.28	2.28
4.2	3.60	63.90	4.69	0.900	3.940	144.20	107.90	2.46
8.2	11.50	61.00	4.26	0.500	4.070	143.70	107.50	2.35
3.6	2.80	59.70	3.95	0.500	4.690	144.50	110.20	2.50
2.3	5.50	48.48	5.58	0.500	3.700	140.62	105.82	2.46
7.3	5.10	60.40	3.43	0.500	3.960	147.80	110.70	2.37
3.5	3.30	41.60	5.47	0.500	4.090	144.08	107.10	2.43
3.1	2.70	37.40	3.87	0.500	3.800	139.30	106.70	2.53
2.6	1.70	46.80	9.44	0.500	4.050	141.70	109.10	2.44
3.6	2.70	42.30	4.41	2.500	3.520	143.40	106.10	2.46
4.2	4.90	46.20	4.96	0.500	3.780	143.30	108.20	2.44
2.3	1.20	47.80	2.95	0.500	3.740	143.10	109.10	2.34
4.7	3.90	47.60	2.80	0.500	3.860	140.50	105.50	2.41
3.6	3.20	52.00	4.51	0.500	3.680	145.00	108.60	2.39
3.0	3.70	42.00	2.00	10.510	4.060	136.00	103.00	2.42
5.9	5.30	47.00	5.83	0.500	3.940	143.50	106.80	2.41
4.2	2.80	49.20	4.60	0.500	3.850	138.00	105.00	2.38
5.5	5.50	36.50	5.06	0.500	3.750	140.40	105.30	2.41
3.0	1.90	35.80	6.61	0.500	4.140	143.10	106.20	2.65
2.3	2.00	39.40	4.57	0.900	3.890	142.40	109.40	2.43
3.4	1.70	49.60	3.93	0.500	3.390	143.50	109.60	2.30
4.3	3.70	43.60	2.20	21.200	4.150	137.30	105.90	2.39
7.5	5.00	55.80	3.22	0.500	3.890	141.70	108.90	2.23
2.9	2.50	49.30	3.36	0.500	4.290	144.40	108.70	2.37
4.0	5.60	66.00	5.20	0.500	3.900	142.00	100.40	2.65
2.6	1.70	60.60	5.31	0.960	3.640	145.30	109.00	2.33
6.9	6.70	60.10	4.70	0.500	3.780	142.70	106.00	2.46
3.9	3.60	48.00	4.34	0.500	4.550	145.00	108.40	2.27
8.5	9.50	46.10	7.00	0.500	3.750	139.70	102.80	2.46
2.6	3.10	72.50	5.14	0.500	3.380	143.80	106.80	2.46
3.6	2.30	65.50	4.03	1.100	3.560	143.30	106.80	2.37
4.6	3.90	48.70	5.08	0.500	3.470	143.10	109.10	1.67
2.8	2.20	56.60	2.56	2.900	3.650	144.50	107.30	2.38

1.6	1.40	41.40	3.56	0.500	3.950	142.20	108.50	2.35
5.5	6.90	50.40	4.38	0.500	3.980	143.60	109.00	2.49
3.2	3.10	47.60	3.80	0.500	3.870	142.50	106.60	2.44
2.4	0.40	55.00	4.32	3.500	3.700	144.70	107.70	2.40
4.5	3.20	45.00	3.66	1.500	3.960	144.60	111.00	2.49
3.3	4.40	52.60	5.09	0.600	4.340	143.00	102.40	2.62
6.0	6.30	58.60	4.39	0.500	#NULL!	#NULL!	#NULL!	#NULL!
6.4	7.80	48.90	5.38	2.000	3.880	141.40	106.40	2.43
2.6	2.40	51.90	7.80	1.700	4.040	141.20	106.00	2.52
2.3	0.80	40.60	5.65	0.500	3.330	146.80	111.00	2.41
2.8	1.80	41.60	2.70	0.500	3.680	144.50	110.50	2.44
5.8	5.70	55.20	3.62	0.500	3.590	144.40	108.20	2.47
3.4	1.60	44.70	4.60	0.500	3.800	140.00	102.20	2.62
44.8	9.60	57.20	4.03	1.200	4.230	141.00	103.70	2.58
6.7	5.10	78.90	4.62	0.500	3.770	146.00	107.90	2.46
5.3	4.80	46.60	3.63	0.500	3.390	143.60	107.50	2.46
2.8	2.10	33.20	3.85	0.500	4.240	142.50	105.30	2.42
4.6	3.90	51.80	5.50	0.500	3.470	145.70	106.80	2.43
2.8	2.30	62.60	4.11	0.500	3.370	144.80	109.50	2.46
3.3	2.40	34.60	2.37	1.600	4.010	142.50	107.20	2.52
6.4	8.40	57.80	4.10	0.500	3.980	142.20	106.50	2.31
5.3	4.60	47.80	4.52	0.500	3.910	142.20	109.30	2.47

碱性磷酸	乳酸脱氢	尿酸	CK	CKMB	血清铁	非铁结合	总铁结合	铁蛋白
8.3	155.0	321.5	68.9	16.80	#NULL!	#NULL!	#NULL!	#NULL!
217.6	146.0	247.7	71.5	14.60	#NULL!	#NULL!	#NULL!	#NULL!
121.5	178.0	618.0	50.7	13.20	#NULL!	#NULL!	#NULL!	#NULL!
125.0	53.0	#NULL!	36.4	16.70	#NULL!	#NULL!	#NULL!	#NULL!
71.8	133.0	384.3	33.5	14.00	#NULL!	#NULL!	#NULL!	#NULL!
155.0	211.0	#NULL!	#NULL!	#NULL!	25.0	29.3	54.30	58.5
172.0	#NULL!	331.0	#NULL!	#NULL!	#NULL!	#NULL!	#NULL!	#NULL!
226.2	155.0	357.0	93.5	13.90	#NULL!	#NULL!	#NULL!	#NULL!
165.0	#NULL!	371.0	#NULL!	#NULL!	#NULL!	#NULL!	#NULL!	#NULL!
171.8	157.0	214.4	49.8	11.00	#NULL!	#NULL!	#NULL!	#NULL!
158.4	130.0	292.0	37.6	12.40	#NULL!	#NULL!	#NULL!	#NULL!
122.3	#NULL!	#NULL!	#NULL!	#NULL!	#NULL!	#NULL!	#NULL!	#NULL!
224.6	#NULL!	252.8	92.8	38.60	11.5	69.6	81.10	37.2
344.0	206.0	282.0	75.8	24.90	#NULL!	#NULL!	#NULL!	#NULL!
88.5	126.0	189.0	22.7	8.40	#NULL!	#NULL!	#NULL!	#NULL!
179.0	#NULL!	242.0	#NULL!	#NULL!	#NULL!	#NULL!	#NULL!	#NULL!
74.9	115.0	190.0	24.9	6.10	3.3	47.9	51.10	11.5
233.0	#NULL!	527.0	41.2	10.40	2.6	99.8	102.40	8.9
266.0	#NULL!	324.0	#NULL!	#NULL!	#NULL!	#NULL!	#NULL!	#NULL!
205.0	#NULL!	264.0	36.1	18.00	#NULL!	#NULL!	#NULL!	#NULL!
89.0	#NULL!	256.0	#NULL!	#NULL!	#NULL!	#NULL!	#NULL!	#NULL!
127.0	#NULL!	521.0	47.0	0.34	#NULL!	#NULL!	#NULL!	#NULL!
135.0	169.0	250.0	47.3	14.40	#NULL!	#NULL!	#NULL!	#NULL!
184.0	126.0	201.0	41.9	13.20	#NULL!	#NULL!	#NULL!	#NULL!
132.0	173.0	277.0	50.8	21.70	#NULL!	#NULL!	#NULL!	#NULL!
124.0	183.0	#NULL!	#NULL!	3.91	#NULL!	#NULL!	#NULL!	#NULL!
114.0	155.0	283.5	71.3	14.10	#NULL!	#NULL!	#NULL!	#NULL!
185.0	158.0	409.7	20.6	9.10	#NULL!	#NULL!	#NULL!	#NULL!
265.3	166.0	#NULL!	54.1	11.20	#NULL!	#NULL!	#NULL!	#NULL!
161.0	149.0	#NULL!	64.4	21.80	#NULL!	#NULL!	#NULL!	#NULL!
260.0	189.0	402.0	109.0	25.90	#NULL!	#NULL!	#NULL!	#NULL!
179.0	138.0	238.6	29.9	23.40	#NULL!	#NULL!	#NULL!	#NULL!
89.0	156.0	321.4	35.4	15.10	#NULL!	#NULL!	#NULL!	#NULL!
87.0	#NULL!	#NULL!	#NULL!	#NULL!	#NULL!	#NULL!	#NULL!	#NULL!
56.0	#NULL!	208.0	#NULL!	#NULL!	#NULL!	#NULL!	#NULL!	#NULL!
37.0	251.0	#NULL!	115.0	36.30	#NULL!	#NULL!	#NULL!	#NULL!
215.0	133.0	173.0	21.8	7.20	#NULL!	#NULL!	#NULL!	#NULL!
300.8	189.0	229.0	83.0	20.40	#NULL!	#NULL!	#NULL!	#NULL!
78.0	165.0	195.8	52.0	2.20	#NULL!	#NULL!	#NULL!	#NULL!
120.0	#NULL!	#NULL!	#NULL!	#NULL!	#NULL!	#NULL!	#NULL!	#NULL!
206.0	191.0	254.0	222.0	13.20	1.4	99.2	100.60	8.8
199.0	156.0	319.0	69.6	18.30	#NULL!	#NULL!	#NULL!	#NULL!
274.0	#NULL!	280.0	#NULL!	#NULL!	#NULL!	#NULL!	#NULL!	#NULL!
247.0	160.0	251.0	61.0	17.00	#NULL!	#NULL!	#NULL!	#NULL!
58.0	120.0	#NULL!	#NULL!	#NULL!	#NULL!	#NULL!	#NULL!	#NULL!
217.0	199.0	#NULL!	71.0	14.70	#NULL!	#NULL!	#NULL!	#NULL!
184.0	244.0	201.0	101.0	93.00	#NULL!	#NULL!	#NULL!	#NULL!
52.6	#NULL!	154.0	#NULL!	#NULL!	#NULL!	#NULL!	#NULL!	#NULL!
90.2	120.0	406.0	49.8	4.40	#NULL!	#NULL!	#NULL!	#NULL!
348.0	205.0	#NULL!	105.8	19.80	#NULL!	#NULL!	#NULL!	#NULL!
188.0	175.0	205.0	222.0	19.90	6.3	51.0	57.20	43.7
259.0	241.0	#NULL!	72.2	25.00	#NULL!	#NULL!	#NULL!	#NULL!
55.1	236.0	107.0	81.6	10.40	#NULL!	#NULL!	#NULL!	#NULL!

171.0	154.0	430.0	40.5	13.70	#NULL!	#NULL!	#NULL!	#NULL!
158.0	826.0	185.0	6358.0	87.40	7.3	39.4	46.70	146.0
138.0	162.0	424.0	153.0	9.00	#NULL!	#NULL!	#NULL!	#NULL!
126.0	303.0	#NULL!	126.0	#NULL!	#NULL!	#NULL!	#NULL!	#NULL!
185.0	162.0	105.0	64.5	3.60	#NULL!	#NULL!	#NULL!	#NULL!
197.0	#NULL!	320.0	#NULL!	#NULL!	#NULL!	#NULL!	#NULL!	#NULL!
72.6	163.0	355.0	69.2	9.50	#NULL!	#NULL!	#NULL!	#NULL!
380.0	224.0	462.0	116.0	19.00	#NULL!	#NULL!	#NULL!	#NULL!
204.0	160.0	227.0	74.0	10.50	#NULL!	#NULL!	#NULL!	#NULL!
176.0	149.0	151.0	20.9	6.70	#NULL!	#NULL!	#NULL!	#NULL!
184.0	206.0	362.0	68.0	20.20	#NULL!	#NULL!	#NULL!	#NULL!
99.7	#NULL!	#NULL!	#NULL!	#NULL!	#NULL!	#NULL!	#NULL!	#NULL!
324.0	133.0	427.0	55.7	9.30	17.1	28.0	46.10	66.2
237.0	183.0	348.0	109.0	14.90	#NULL!	#NULL!	#NULL!	#NULL!
195.0	182.0	278.0	91.0	13.60	#NULL!	#NULL!	#NULL!	#NULL!
152.0	67.4	462.0	67.4	14.00	#NULL!	#NULL!	#NULL!	#NULL!
47.0	#NULL!	#NULL!	#NULL!	#NULL!	#NULL!	#NULL!	#NULL!	#NULL!
73.7	#NULL!	331.0	#NULL!	#NULL!	#NULL!	#NULL!	#NULL!	#NULL!
72.3	#NULL!	207.0	#NULL!	#NULL!	#NULL!	#NULL!	#NULL!	#NULL!
190.0	#NULL!	348.0	#NULL!	#NULL!	#NULL!	#NULL!	#NULL!	#NULL!
395.0	#NULL!	#NULL!	#NULL!	#NULL!	#NULL!	#NULL!	#NULL!	#NULL!
150.0	164.0	161.0	12.1	6.20	#NULL!	#NULL!	#NULL!	#NULL!
224.0	#NULL!	400.0	#NULL!	#NULL!	#NULL!	#NULL!	#NULL!	#NULL!
181.0	#NULL!	283.0	#NULL!	#NULL!	#NULL!	#NULL!	#NULL!	#NULL!
226.0	#NULL!	354.0	#NULL!	#NULL!	#NULL!	#NULL!	#NULL!	#NULL!
201.0	143.0	353.0	57.1	15.10	10.9	71.8	82.70	73.5
344.0	179.0	374.0	78.2	12.80	#NULL!	#NULL!	#NULL!	#NULL!
190.5	136.0	339.0	58.4	24.10	#NULL!	#NULL!	#NULL!	#NULL!
150.0	295.0	216.0	169.3	15.90	3.9	86.3	89.20	10.2
174.0	#NULL!	368.0	#NULL!	#NULL!	#NULL!	#NULL!	#NULL!	#NULL!
98.0	220.0	#NULL!	93.0	0.91	#NULL!	#NULL!	#NULL!	#NULL!
161.0	163.0	299.0	42.6	14.70	#NULL!	#NULL!	#NULL!	#NULL!
146.0	#NULL!	153.0	#NULL!	#NULL!	#NULL!	#NULL!	#NULL!	#NULL!
193.0	164.0	244.0	61.7	11.90	#NULL!	#NULL!	#NULL!	#NULL!
117.0	#NULL!	#NULL!	#NULL!	#NULL!	#NULL!	#NULL!	#NULL!	#NULL!
178.0	266.0	306.0	243.0	69.60	#NULL!	#NULL!	#NULL!	#NULL!
155.0	175.0	261.0	44.7	16.70	#NULL!	#NULL!	#NULL!	#NULL!
203.0	186.0	229.0	95.7	13.80	#NULL!	#NULL!	#NULL!	#NULL!
192.0	194.0	215.0	116.0	22.00	#NULL!	#NULL!	#NULL!	#NULL!
148.0	#NULL!	312.0	43.7	5.60	#NULL!	#NULL!	#NULL!	#NULL!
179.0	179.0	259.0	46.6	22.30	#NULL!	#NULL!	#NULL!	#NULL!
122.0	162.0	389.0	#NULL!	#NULL!	#NULL!	#NULL!	#NULL!	#NULL!
259.0	#NULL!	409.0	#NULL!	#NULL!	#NULL!	#NULL!	#NULL!	#NULL!
215.0	210.0	275.0	73.6	24.10	#NULL!	#NULL!	#NULL!	#NULL!
378.0	#NULL!	350.0	#NULL!	#NULL!	#NULL!	#NULL!	#NULL!	#NULL!
182.0	#NULL!	180.0	54.0	12.10	#NULL!	#NULL!	#NULL!	#NULL!
134.0	#NULL!	377.0	#NULL!	#NULL!	#NULL!	#NULL!	#NULL!	#NULL!
125.0	289.0	#NULL!	#NULL!	#NULL!	#NULL!	#NULL!	#NULL!	#NULL!
190.0	166.0	267.0	65.7	16.30	#NULL!	#NULL!	#NULL!	#NULL!
165.0	210.0	435.0	47.6	16.10	#NULL!	#NULL!	#NULL!	#NULL!
184.0	186.0	430.0	56.3	12.20	#NULL!	#NULL!	#NULL!	#NULL!
233.0	207.0	322.0	116.0	14.80	#NULL!	#NULL!	#NULL!	#NULL!
197.0	215.0	266.0	110.0	18.50	18.4	49.2	67.60	97.0
73.0	#NULL!	180.0	#NULL!	1.22	1.9	76.0	78.00	7.4

194.0	160.0	390.0	60.0	11.40	#NULL!	#NULL!	#NULL!	#NULL!
36.0	#NULL!	#NULL!	#NULL!	#NULL!	#NULL!	#NULL!	#NULL!	#NULL!
85.0	#NULL!	#NULL!	#NULL!	#NULL!	#NULL!	#NULL!	#NULL!	#NULL!
331.0	167.0	297.0	77.0	30.80	#NULL!	#NULL!	#NULL!	#NULL!
195.0	180.0	#NULL!	50.2	7.90	#NULL!	#NULL!	#NULL!	#NULL!
297.0	187.0	#NULL!	104.0	0.67	#NULL!	#NULL!	#NULL!	#NULL!
250.3	172.0	284.0	79.3	24.60	20.2	64.6	84.80	37.2
165.5	179.0	387.0	164.0	23.30	#NULL!	#NULL!	#NULL!	#NULL!
84.7	118.0	413.0	28.0	6.70	2.8	63.0	65.80	47.8
250.0	185.0	292.0	59.0	18.20	13.7	59.0	72.70	62.7
231.0	201.0	363.0	49.6	32.00	#NULL!	#NULL!	#NULL!	#NULL!
112.0	#NULL!	307.0	#NULL!	#NULL!	#NULL!	#NULL!	#NULL!	#NULL!
218.0	157.0	290.0	72.0	19.20	23.0	44.0	67.60	146.0
241.0	172.0	427.0	67.0	15.00	15.7	62.0	78.00	116.0
140.0	159.0	314.0	45.4	15.40	#NULL!	#NULL!	#NULL!	#NULL!
214.0	200.0	313.0	73.5	18.10	#NULL!	#NULL!	#NULL!	#NULL!
182.0	172.0	202.0	36.4	15.90	#NULL!	#NULL!	#NULL!	#NULL!
291.0	#NULL!	#NULL!	#NULL!	#NULL!	#NULL!	#NULL!	#NULL!	#NULL!
259.0	136.0	490.0	67.8	13.70	#NULL!	#NULL!	#NULL!	#NULL!
201.0	165.0	217.0	74.9	14.40	#NULL!	#NULL!	#NULL!	#NULL!
170.0	149.0	266.0	61.7	15.40	#NULL!	#NULL!	#NULL!	#NULL!
150.0	147.0	353.0	87.3	17.30	#NULL!	#NULL!	#NULL!	#NULL!
143.0	174.0	172.0	85.6	17.10	#NULL!	#NULL!	#NULL!	#NULL!
252.0	136.0	291.0	41.4	15.60	#NULL!	#NULL!	#NULL!	#NULL!
130.0	201.0	334.0	135.0	18.80	#NULL!	#NULL!	#NULL!	#NULL!
128.0	#NULL!	#NULL!	#NULL!	#NULL!	#NULL!	#NULL!	#NULL!	#NULL!
134.0	141.0	332.0	49.5	12.10	#NULL!	#NULL!	#NULL!	#NULL!
182.0	131.0	228.0	59.7	14.90	13.2	37.2	50.40	69.0
110.0	#NULL!	282.0	#NULL!	#NULL!	#NULL!	#NULL!	#NULL!	#NULL!
57.9	#NULL!	177.0	#NULL!	0.10	#NULL!	#NULL!	#NULL!	#NULL!
21.2	103.0	#NULL!	21.2	8.40	3.5	59.4	62.90	59.0
156.0	#NULL!	#NULL!	#NULL!	#NULL!	#NULL!	#NULL!	#NULL!	#NULL!
148.8	#NULL!	237.0	#NULL!	#NULL!	#NULL!	#NULL!	#NULL!	#NULL!
240.7	113.0	333.5	37.8	11.80	#NULL!	#NULL!	#NULL!	#NULL!
186.3	206.0	176.5	83.8	15.30	#NULL!	#NULL!	#NULL!	#NULL!
325.4	164.0	605.5	79.8	10.00	#NULL!	#NULL!	#NULL!	#NULL!
147.9	159.0	584.5	24.6	14.30	#NULL!	#NULL!	#NULL!	#NULL!
165.2	186.0	233.2	64.4	17.30	#NULL!	#NULL!	#NULL!	#NULL!
62.6	#NULL!	402.1	#NULL!	#NULL!	#NULL!	#NULL!	#NULL!	#NULL!
130.9	169.0	305.4	47.4	26.60	#NULL!	#NULL!	#NULL!	#NULL!
131.8	157.0	291.0	78.3	25.20	31.0	15.4	46.40	72.7
203.1	145.0	273.8	34.8	16.80	#NULL!	#NULL!	#NULL!	#NULL!
222.3	113.0	358.9	42.3	5.80	#NULL!	#NULL!	#NULL!	#NULL!
150.6	#NULL!	237.4	#NULL!	#NULL!	#NULL!	#NULL!	#NULL!	#NULL!
266.3	191.0	324.9	119.6	8.40	#NULL!	#NULL!	#NULL!	#NULL!
133.1	108.0	316.3	44.1	10.70	4.6	38.9	43.50	56.7
208.5	149.0	369.5	122.1	17.10	#NULL!	#NULL!	#NULL!	#NULL!
632.0	#NULL!	323.9	#NULL!	#NULL!	#NULL!	#NULL!	#NULL!	#NULL!
204.3	117.0	314.3	53.1	22.20	#NULL!	#NULL!	#NULL!	#NULL!
189.0	78.0	#NULL!	#NULL!	#NULL!	#NULL!	#NULL!	#NULL!	#NULL!
238.4	142.0	306.7	73.2	9.40	#NULL!	#NULL!	#NULL!	#NULL!
113.7	158.0	174.6	59.6	18.20	#NULL!	#NULL!	#NULL!	#NULL!
83.9	#NULL!	398.1	#NULL!	#NULL!	#NULL!	#NULL!	#NULL!	#NULL!
139.7	169.0	348.0	95.7	35.80	#NULL!	#NULL!	#NULL!	#NULL!

223.3	#NULL!	435.1	#NULL!	#NULL!	#NULL!	#NULL!	#NULL!	#NULL!
193.2	#NULL!	345.3	#NULL!	#NULL!	#NULL!	#NULL!	#NULL!	#NULL!
81.0	300.0	305.8	393.9	33.40	2.0	79.3	81.30	9.4
171.5	147.0	338.2	104.8	15.30	#NULL!	#NULL!	#NULL!	#NULL!
380.8	#NULL!	#NULL!	#NULL!	#NULL!	#NULL!	#NULL!	#NULL!	#NULL!
159.0	188.0	119.0	47.7	6.70	#NULL!	#NULL!	#NULL!	#NULL!
234.0	#NULL!	#NULL!	#NULL!	#NULL!	#NULL!	#NULL!	#NULL!	#NULL!
201.0	153.0	#NULL!	59.3	1.00	#NULL!	#NULL!	#NULL!	#NULL!
27.7	187.0	215.0	18.5	6.80	8.3	17.0	26.00	145.0
171.7	156.0	298.2	67.3	8.50	#NULL!	#NULL!	#NULL!	#NULL!
188.0	#NULL!	#NULL!	#NULL!	#NULL!	#NULL!	#NULL!	#NULL!	#NULL!
158.3	#NULL!	275.2	#NULL!	#NULL!	#NULL!	#NULL!	#NULL!	#NULL!
147.0	#NULL!	#NULL!	#NULL!	#NULL!	#NULL!	#NULL!	#NULL!	#NULL!
114.3	223.0	233.6	76.3	12.60	#NULL!	#NULL!	#NULL!	#NULL!
162.9	182.0	270.5	134.8	19.60	#NULL!	#NULL!	#NULL!	#NULL!
159.4	177.0	282.1	75.7	20.80	#NULL!	#NULL!	#NULL!	#NULL!
185.6	192.0	369.8	108.3	22.30	#NULL!	#NULL!	#NULL!	#NULL!
105.2	143.0	270.0	62.9	22.00	#NULL!	#NULL!	#NULL!	#NULL!
202.3	166.0	280.9	19.0	16.30	#NULL!	#NULL!	#NULL!	#NULL!
207.5	#NULL!	472.1	#NULL!	#NULL!	#NULL!	#NULL!	#NULL!	#NULL!
125.0	#NULL!	#NULL!	#NULL!	#NULL!	#NULL!	#NULL!	#NULL!	#NULL!
149.4	115.0	250.0	37.4	8.20	#NULL!	#NULL!	#NULL!	#NULL!
216.6	#NULL!	245.1	#NULL!	#NULL!	#NULL!	#NULL!	#NULL!	#NULL!
181.7	#NULL!	378.0	#NULL!	#NULL!	18.1	70.2	88.30	45.2
136.0	#NULL!	#NULL!	#NULL!	#NULL!	#NULL!	#NULL!	#NULL!	#NULL!
160.6	94.0	#NULL!	189.1	18.70	#NULL!	#NULL!	#NULL!	#NULL!
129.0	#NULL!	#NULL!	#NULL!	#NULL!	#NULL!	#NULL!	#NULL!	#NULL!
153.4	#NULL!	152.2	#NULL!	#NULL!	7.2	43.9	51.10	164.0
77.5	#NULL!	64.4	#NULL!	#NULL!	2.6	43.4	46.00	179.0
128.0	186.0	306.0	92.9	53.10	2.3	65.9	68.40	16.6
129.6	113.0	390.4	24.8	9.10	#NULL!	#NULL!	#NULL!	#NULL!
252.0	159.0	510.6	61.6	14.20	#NULL!	#NULL!	#NULL!	#NULL!
445.5	137.0	350.7	63.8	17.20	#NULL!	#NULL!	#NULL!	#NULL!
111.0	#NULL!	260.8	#NULL!	#NULL!	#NULL!	#NULL!	#NULL!	#NULL!
205.2	122.0	269.9	83.0	11.40	#NULL!	#NULL!	#NULL!	#NULL!
176.0	113.0	401.0	54.6	3.80	#NULL!	#NULL!	#NULL!	#NULL!
219.0	#NULL!	249.0	#NULL!	#NULL!	#NULL!	#NULL!	#NULL!	#NULL!
172.0	#NULL!	#NULL!	#NULL!	#NULL!	#NULL!	#NULL!	#NULL!	#NULL!
51.0	#NULL!	#NULL!	#NULL!	#NULL!	3.2	26.2	29.49	114.0
385.0	#NULL!	#NULL!	#NULL!	#NULL!	#NULL!	#NULL!	#NULL!	#NULL!
82.0	128.0	345.0	#NULL!	#NULL!	#NULL!	#NULL!	#NULL!	#NULL!
158.0	#NULL!	#NULL!	#NULL!	#NULL!	#NULL!	#NULL!	#NULL!	#NULL!
205.0	217.0	344.0	#NULL!	#NULL!	#NULL!	#NULL!	#NULL!	#NULL!
95.0	#NULL!	202.0	#NULL!	#NULL!	#NULL!	#NULL!	#NULL!	#NULL!
384.0	#NULL!	89.0	#NULL!	#NULL!	9.0	21.8	30.80	149.0
259.0	139.0	384.0	#NULL!	#NULL!	#NULL!	#NULL!	#NULL!	#NULL!
115.0	240.0	183.0	74.2	24.30	2.5	38.0	40.50	19.6
434.0	#NULL!	431.0	#NULL!	#NULL!	#NULL!	#NULL!	#NULL!	#NULL!
168.0	#NULL!	#NULL!	#NULL!	#NULL!	#NULL!	#NULL!	#NULL!	#NULL!
198.0	183.0	#NULL!	#NULL!	#NULL!	#NULL!	#NULL!	#NULL!	#NULL!
121.0	153.0	316.0	#NULL!	16.00	21.2	24.4	45.60	135.0
126.0	162.0	376.0	#NULL!	25.00	#NULL!	#NULL!	#NULL!	#NULL!
80.0	#NULL!	356.0	#NULL!	#NULL!	#NULL!	#NULL!	#NULL!	#NULL!
128.0	127.0	289.0	#NULL!	13.20	#NULL!	#NULL!	#NULL!	#NULL!

324.0	119.0	309.0	55.2	19.10	#NULL!	#NULL!	#NULL!	#NULL!
156.0	#NULL!	#NULL!	#NULL!	#NULL!	#NULL!	#NULL!	#NULL!	#NULL!
186.0	170.0	297.0	97.6	16.60	#NULL!	#NULL!	#NULL!	#NULL!
451.0	#NULL!	477.0	#NULL!	#NULL!	#NULL!	#NULL!	#NULL!	#NULL!
114.0	#NULL!	245.0	#NULL!	#NULL!	#NULL!	#NULL!	#NULL!	#NULL!
145.0	#NULL!	#NULL!	#NULL!	#NULL!	#NULL!	#NULL!	#NULL!	#NULL!
210.0	#NULL!	311.0	26.5	9.20	14.7	37.6	52.30	356.0
104.0	#NULL!	207.0	45.7	4.90	#NULL!	#NULL!	#NULL!	#NULL!
266.0	#NULL!	#NULL!	#NULL!	#NULL!	3.5	30.0	33.50	9.7
175.0	#NULL!	266.0	#NULL!	#NULL!	#NULL!	#NULL!	#NULL!	#NULL!
126.0	142.0	218.0	19.3	5.80	#NULL!	#NULL!	#NULL!	#NULL!
257.0	#NULL!	296.0	#NULL!	#NULL!	#NULL!	#NULL!	#NULL!	#NULL!
243.0	#NULL!	360.0	#NULL!	#NULL!	#NULL!	#NULL!	#NULL!	#NULL!
215.0	#NULL!	178.0	#NULL!	#NULL!	#NULL!	#NULL!	#NULL!	#NULL!
158.0	#NULL!	#NULL!	#NULL!	#NULL!	#NULL!	#NULL!	#NULL!	#NULL!
240.0	218.0	371.0	748.0	13.30	#NULL!	#NULL!	#NULL!	#NULL!
213.0	138.0	479.0	20.9	9.90	20.8	30.0	50.80	112.0
156.0	#NULL!	#NULL!	#NULL!	#NULL!	#NULL!	#NULL!	#NULL!	#NULL!
153.0	138.0	250.0	62.8	7.10	14.3	37.3	51.60	54.5
123.0	85.0	391.0	24.3	9.60	7.4	60.9	68.30	48.3
176.0	#NULL!	220.0	33.1	19.50	10.9	9.2	20.10	161.0
154.0	142.0	#NULL!	108.0	79.50	#NULL!	#NULL!	#NULL!	#NULL!
147.0	#NULL!	#NULL!	61.8	12.70	#NULL!	#NULL!	#NULL!	#NULL!
223.0	#NULL!	#NULL!	#NULL!	#NULL!	#NULL!	#NULL!	#NULL!	#NULL!
233.0	#NULL!	#NULL!	#NULL!	#NULL!	#NULL!	#NULL!	#NULL!	#NULL!
218.0	212.0	#NULL!	120.0	20.30	#NULL!	#NULL!	#NULL!	#NULL!
353.0	133.0	90.0	59.6	11.20	15.6	30.8	46.40	243.0
124.0	87.0	#NULL!	#NULL!	#NULL!	#NULL!	#NULL!	#NULL!	#NULL!
135.0	#NULL!	#NULL!	#NULL!	#NULL!	#NULL!	#NULL!	#NULL!	#NULL!
164.0	90.0	#NULL!	#NULL!	#NULL!	#NULL!	#NULL!	#NULL!	#NULL!
83.0	#NULL!	#NULL!	#NULL!	#NULL!	17.2	10.9	28.10	68.7
189.0	#NULL!	230.0	#NULL!	#NULL!	6.5	20.5	27.00	9.3
158.0	#NULL!	#NULL!	#NULL!	#NULL!	#NULL!	#NULL!	#NULL!	#NULL!
188.0	163.0	#NULL!	105.0	16.80	#NULL!	#NULL!	#NULL!	#NULL!
121.0	#NULL!	#NULL!	#NULL!	#NULL!	#NULL!	#NULL!	#NULL!	#NULL!
122.0	#NULL!	#NULL!	#NULL!	#NULL!	#NULL!	#NULL!	#NULL!	#NULL!
112.0	#NULL!	#NULL!	#NULL!	#NULL!	#NULL!	#NULL!	#NULL!	#NULL!
287.0	#NULL!	#NULL!	#NULL!	#NULL!	#NULL!	#NULL!	#NULL!	#NULL!
108.0	249.0	160.0	28.6	6.70	#NULL!	#NULL!	#NULL!	#NULL!
206.0	162.0	256.0	197.0	3.00	4.2	20.4	24.60	20.6
309.0	#NULL!	326.0	#NULL!	#NULL!	#NULL!	#NULL!	#NULL!	#NULL!
144.0	#NULL!	#NULL!	#NULL!	#NULL!	#NULL!	#NULL!	#NULL!	#NULL!
158.0	#NULL!	#NULL!	#NULL!	#NULL!	#NULL!	#NULL!	#NULL!	#NULL!
21.0	#NULL!	430.0	#NULL!	#NULL!	#NULL!	#NULL!	#NULL!	#NULL!
246.0	#NULL!	#NULL!	#NULL!	#NULL!	#NULL!	#NULL!	#NULL!	#NULL!
305.0	#NULL!	211.0	#NULL!	#NULL!	36.7	11.8	48.30	363.0
156.0	#NULL!	#NULL!	#NULL!	#NULL!	#NULL!	#NULL!	#NULL!	#NULL!
178.0	#NULL!	#NULL!	#NULL!	#NULL!	34.4	9.5	43.90	70.7
189.0	#NULL!	#NULL!	#NULL!	#NULL!	#NULL!	#NULL!	#NULL!	#NULL!
156.0	#NULL!	#NULL!	#NULL!	#NULL!	#NULL!	#NULL!	#NULL!	#NULL!
122.0	#NULL!	#NULL!	#NULL!	#NULL!	#NULL!	#NULL!	#NULL!	#NULL!
282.0	#NULL!	#NULL!	#NULL!	#NULL!	#NULL!	#NULL!	#NULL!	#NULL!
362.0	#NULL!	#NULL!	#NULL!	#NULL!	#NULL!	#NULL!	#NULL!	#NULL!
135.0	#NULL!	#NULL!	#NULL!	#NULL!	#NULL!	#NULL!	#NULL!	#NULL!

188.0	#NULL!	#NULL!	#NULL!	#NULL!	3.8	29.4	33.20	10.2
175.0	#NULL!	#NULL!	#NULL!	#NULL!	#NULL!	#NULL!	#NULL!	#NULL!
147.0	#NULL!	#NULL!	#NULL!	#NULL!	#NULL!	#NULL!	#NULL!	#NULL!
121.0	203.0	#NULL!	#NULL!	#NULL!	12.2	6.0	18.20	39.3
89.0	#NULL!	#NULL!	58.7	11.80	#NULL!	#NULL!	#NULL!	#NULL!
57.0	#NULL!	#NULL!	#NULL!	#NULL!	#NULL!	#NULL!	#NULL!	#NULL!
48.0	93.0	#NULL!	#NULL!	#NULL!	#NULL!	#NULL!	#NULL!	#NULL!
89.0	#NULL!	#NULL!	#NULL!	#NULL!	#NULL!	#NULL!	#NULL!	#NULL!
75.0	#NULL!	304.0	#NULL!	#NULL!	#NULL!	#NULL!	#NULL!	#NULL!
98.0	#NULL!	#NULL!	#NULL!	#NULL!	#NULL!	#NULL!	#NULL!	#NULL!
85.0	#NULL!	#NULL!	#NULL!	#NULL!	11.9	17.7	29.00	#NULL!
158.0	#NULL!	#NULL!	#NULL!	#NULL!	#NULL!	#NULL!	#NULL!	#NULL!
116.0	#NULL!	#NULL!	#NULL!	#NULL!	#NULL!	#NULL!	#NULL!	#NULL!
104.0	#NULL!	#NULL!	#NULL!	#NULL!	#NULL!	#NULL!	#NULL!	#NULL!
94.0	#NULL!	#NULL!	#NULL!	#NULL!	#NULL!	#NULL!	#NULL!	#NULL!
270.0	#NULL!	316.0	#NULL!	#NULL!	#NULL!	#NULL!	#NULL!	#NULL!
181.0	#NULL!	#NULL!	#NULL!	#NULL!	#NULL!	#NULL!	#NULL!	#NULL!
196.0	#NULL!	#NULL!	#NULL!	#NULL!	#NULL!	#NULL!	#NULL!	#NULL!
369.0	#NULL!	#NULL!	#NULL!	#NULL!	#NULL!	#NULL!	#NULL!	#NULL!
243.0	#NULL!	#NULL!	#NULL!	#NULL!	7.0	20.2	27.20	10.2
260.0	322.0	73.0	123.0	41.50	#NULL!	#NULL!	#NULL!	#NULL!
68.0	#NULL!	#NULL!	#NULL!	#NULL!	#NULL!	#NULL!	#NULL!	#NULL!
68.0	#NULL!	#NULL!	#NULL!	#NULL!	#NULL!	#NULL!	#NULL!	#NULL!
267.0	#NULL!	#NULL!	#NULL!	#NULL!	#NULL!	#NULL!	#NULL!	#NULL!
98.0	#NULL!	#NULL!	#NULL!	#NULL!	#NULL!	#NULL!	#NULL!	#NULL!
85.0	#NULL!	#NULL!	#NULL!	#NULL!	#NULL!	#NULL!	#NULL!	#NULL!
78.0	#NULL!	#NULL!	#NULL!	#NULL!	#NULL!	#NULL!	#NULL!	#NULL!
85.0	#NULL!	#NULL!	#NULL!	#NULL!	#NULL!	#NULL!	#NULL!	#NULL!
28.0	#NULL!	#NULL!	#NULL!	#NULL!	#NULL!	#NULL!	#NULL!	#NULL!
125.0	#NULL!	#NULL!	#NULL!	#NULL!	#NULL!	#NULL!	#NULL!	#NULL!
254.0	123.0	#NULL!	#NULL!	#NULL!	#NULL!	#NULL!	#NULL!	#NULL!
247.0	#NULL!	#NULL!	#NULL!	#NULL!	#NULL!	#NULL!	#NULL!	#NULL!
125.0	#NULL!	#NULL!	#NULL!	#NULL!	#NULL!	#NULL!	#NULL!	#NULL!
200.0	155.0	506.0	94.2	10.40	#NULL!	#NULL!	#NULL!	#NULL!
139.0	#NULL!	#NULL!	#NULL!	#NULL!	11.6	28.5	40.00	11.3
158.0	#NULL!	#NULL!	#NULL!	#NULL!	#NULL!	#NULL!	#NULL!	#NULL!
184.0	#NULL!	#NULL!	#NULL!	#NULL!	#NULL!	#NULL!	#NULL!	#NULL!
168.0	167.0	#NULL!	#NULL!	#NULL!	#NULL!	#NULL!	#NULL!	#NULL!
245.0	177.0	271.0	74.2	11.20	9.1	33.0	42.00	73.0
181.0	#NULL!	373.0	54.9	13.50	#NULL!	#NULL!	#NULL!	#NULL!
169.0	#NULL!	262.0	25.5	8.60	26.8	7.8	34.60	165.8
99.0	111.0	439.0	36.1	12.90	13.0	32.0	45.00	118.0
943.0	#NULL!	160.0	46.2	27.20	8.0	33.2	41.20	2052.0
82.0	#NULL!	#NULL!	#NULL!	#NULL!	#NULL!	#NULL!	#NULL!	#NULL!
166.0	#NULL!	#NULL!	#NULL!	#NULL!	#NULL!	#NULL!	#NULL!	#NULL!
178.0	175.0	241.0	67.7	15.30	11.9	32.0	43.90	30.4
111.0	158.0	229.0	44.2	15.90	12.1	38.2	50.30	28.8
147.0	#NULL!	#NULL!	#NULL!	#NULL!	#NULL!	#NULL!	#NULL!	#NULL!
218.0	221.0	311.0	91.0	22.60	17.0	26.8	14.70	142.0
183.0	#NULL!	414.0	105.0	19.90	#NULL!	#NULL!	#NULL!	#NULL!
103.0	122.0	311.0	29.4	7.80	14.9	28.7	43.60	39.3
226.0	#NULL!	#NULL!	#NULL!	#NULL!	#NULL!	#NULL!	#NULL!	#NULL!
240.0	185.0	412.0	176.0	18.50	#NULL!	#NULL!	#NULL!	#NULL!
71.9	153.0	430.0	93.7	10.90	8.0	43.9	46.90	2.6

182.0	156.0	334.0	111.0	8.40	15.8	12.9	28.70	638.0
231.0	206.0	472.0	76.5	12.50	10.8	52.6	63.40	111.0
215.0	168.0	273.0	36.3	17.10	8.2	35.1	44.30	73.7
169.0	144.0	318.0	48.5	16.40	32.3	11.0	44.20	67.9
234.0	213.0	324.0	78.2	19.60	#NULL!	#NULL!	#NULL!	#NULL!
163.0	166.0	315.0	45.5	18.10	12.4	27.7	40.10	115.0
330.0	174.0	348.0	77.0	17.60	11.5	26.4	41.00	84.8
106.0	131.0	514.0	80.0	8.40	13.3	32.6	45.90	119.0
170.0	#NULL!	285.0	#NULL!	#NULL!	7.3	28.9	36.20	98.6
134.0	179.0	178.0	69.0	19.30	10.7	73.3	84.00	4.8
109.0	124.0	274.0	36.3	14.00	6.8	22.1	28.90	179.9
170.0	166.0	343.0	92.2	13.00	12.1	36.5	48.60	38.2
176.0	141.0	503.0	137.0	14.70	7.0	47.7	54.70	73.3
315.0	185.0	313.0	149.0	27.10	13.0	42.9	56.90	32.3
162.0	184.0	258.0	96.8	18.40	12.0	39.1	51.10	91.4
249.0	187.0	252.0	76.1	36.20	25.0	29.3	54.20	99.5
255.0	161.0	342.0	#NULL!	#NULL!	#NULL!	#NULL!	#NULL!	#NULL!
278.0	216.0	262.0	66.2	20.10	16.9	33.0	43.90	51.5
232.0	147.0	427.0	53.5	20.80	18.0	34.4	52.40	49.2
272.0	249.0	529.0	104.0	19.30	#NULL!	#NULL!	#NULL!	#NULL!
154.0	221.0	355.0	56.0	18.10	2.8	36.1	38.90	84.5
144.0	175.0	#NULL!	#NULL!	#NULL!	#NULL!	#NULL!	#NULL!	#NULL!
216.0	207.0	334.0	59.9	17.90	14.0	37.1	51.10	102.4
87.0	#NULL!	#NULL!	#NULL!	#NULL!	#NULL!	#NULL!	#NULL!	#NULL!
176.0	164.0	510.0	85.2	19.80	#NULL!	#NULL!	#NULL!	#NULL!
148.0	145.0	286.0	25.5	12.10	#NULL!	#NULL!	#NULL!	#NULL!
222.0	190.0	206.0	52.9	18.70	#NULL!	#NULL!	#NULL!	#NULL!
232.0	181.0	294.0	147.0	20.50	9.9	42.3	52.10	74.0
177.0	#NULL!	284.0	#NULL!	#NULL!	#NULL!	#NULL!	#NULL!	#NULL!
152.0	185.0	307.0	56.2	13.50	#NULL!	#NULL!	#NULL!	#NULL!
178.0	133.0	#NULL!	#NULL!	#NULL!	4.0	44.9	48.90	14.4
158.0	203.0	#NULL!	#NULL!	#NULL!	3.5	33.4	36.90	93.0
232.0	#NULL!	358.0	#NULL!	#NULL!	31.1	20.8	41.90	98.6
72.0	132.0	169.0	12.6	5.00	#NULL!	#NULL!	#NULL!	#NULL!
102.0	340.0	297.0	48.6	21.60	#NULL!	#NULL!	#NULL!	#NULL!
68.9	#NULL!	#NULL!	#NULL!	#NULL!	2.0	24.0	26.00	262.9
70.0	#NULL!	#NULL!	#NULL!	#NULL!	#NULL!	#NULL!	#NULL!	#NULL!
84.0	192.0	195.2	48.1	25.50	2.2	40.5	42.70	4.1
89.0	134.0	#NULL!	#NULL!	#NULL!	#NULL!	#NULL!	#NULL!	#NULL!
237.0	227.0	399.0	110.8	22.50	11.0	9.4	43.40	121.0
360.0	#NULL!	526.0	72.5	14.50	16.8	40.3	57.10	87.2
78.0	#NULL!	415.0	60.2	12.50	#NULL!	#NULL!	#NULL!	#NULL!
260.0	167.0	300.0	62.2	14.30	#NULL!	#NULL!	#NULL!	#NULL!
152.0	179.0	209.0	104.0	17.50	#NULL!	#NULL!	#NULL!	#NULL!
200.0	174.0	291.0	91.0	10.00	4.4	63.1	67.50	1.9
191.0	311.0	491.0	124.0	10.10	#NULL!	#NULL!	#NULL!	#NULL!
134.0	222.0	306.0	54.2	24.00	3.1	55.9	59.00	6.9
149.0	#NULL!	226.0	110.0	22.50	17.7	26.4	44.10	98.7
143.0	161.0	241.0	41.0	15.70	3.4	51.5	54.90	6.9
99.9	158.0	550.0	77.4	17.40	23.5	25.5	49.00	130.0
59.0	151.0	249.0	50.7	18.00	12.0	27.0	39.00	51.5
125.0	165.0	256.0	82.4	11.40	10.8	32.4	43.20	87.6
101.0	191.0	268.0	87.7	16.10	27.5	29.1	56.60	40.4
220.0	212.0	403.0	99.6	11.70	13.2	41.4	54.60	274.0

121.0	#NULL!	281.0	#NULL!	#NULL!	#NULL!	#NULL!	#NULL!	#NULL!
201.0	158.0	519.0	70.2	25.00	17.5	24.1	41.60	121.0
116.0	127.0	255.0	11.2	0.40	3.6	30.3	32.90	179.0
98.0	#NULL!	#NULL!	#NULL!	#NULL!	#NULL!	#NULL!	#NULL!	#NULL!
51.0	#NULL!	250.8	#NULL!	#NULL!	#NULL!	#NULL!	#NULL!	#NULL!
343.0	190.0	318.0	63.0	19.50	31.9	23.8	55.70	94.0
244.0	150.0	334.0	55.6	15.30	#NULL!	#NULL!	#NULL!	#NULL!
203.0	141.0	411.0	43.0	20.40	#NULL!	#NULL!	#NULL!	#NULL!
222.7	532.0	331.0	29.6	16.50	3.4	40.6	44.00	310.0
214.0	168.0	212.0	90.8	15.10	22.8	26.1	48.80	25.7
158.0	148.0	300.0	28.7	10.50	12.0	35.2	47.20	23.2
178.0	#NULL!	297.0	95.0	32.00	10.8	51.6	62.00	48.7
235.2	211.0	420.2	77.1	12.00	14.1	36.3	50.40	94.4
138.0	159.0	237.0	46.2	10.70	13.6	32.7	40.30	35.9
279.0	164.0	439.0	55.7	25.30	#NULL!	#NULL!	#NULL!	#NULL!
131.0	167.0	221.0	90.6	11.00	#NULL!	#NULL!	#NULL!	#NULL!
170.0	178.0	332.0	55.0	11.60	30.9	23.0	43.20	104.2
78.9	116.0	449.0	51.2	15.00	#NULL!	#NULL!	#NULL!	#NULL!
191.0	201.0	473.0	82.4	20.40	#NULL!	#NULL!	#NULL!	#NULL!
347.0	162.0	269.0	66.7	14.70	24.5	31.0	55.50	58.6
212.0	204.0	380.0	56.0	27.60	#NULL!	#NULL!	#NULL!	#NULL!
149.0	213.0	274.0	97.5	16.60	#NULL!	#NULL!	#NULL!	#NULL!
184.0	193.0	341.0	67.2	17.40	#NULL!	#NULL!	#NULL!	#NULL!
199.5	171.0	248.0	66.3	13.00	18.3	35.1	50.20	111.4
289.0	209.0	227.0	108.0	19.10	#NULL!	#NULL!	#NULL!	#NULL!
290.0	204.0	339.0	54.0	21.00	8.6	40.3	48.90	84.3
359.0	184.0	358.0	146.0	19.70	#NULL!	#NULL!	#NULL!	#NULL!
90.0	160.0	316.0	82.6	11.70	11.5	31.9	43.40	84.7
142.0	186.0	307.0	56.6	16.00	3.8	39.2	52.00	27.4
261.0	184.0	339.0	98.7	14.30	18.4	27.6	46.00	70.6
95.0	168.0	#NULL!	#NULL!	#NULL!	16.0	8.9	23.90	58.8
54.0	#NULL!	#NULL!	#NULL!	#NULL!	#NULL!	#NULL!	#NULL!	#NULL!
125.0	135.0	272.0	68.0	13.10	#NULL!	#NULL!	#NULL!	#NULL!
231.0	178.0	274.0	75.4	15.50	14.9	27.7	42.60	109.0
183.0	376.0	265.0	217.0	57.60	8.5	39.7	48.20	81.0
167.0	#NULL!	170.0	#NULL!	#NULL!	9.5	33.3	47.00	40.0
90.4	#NULL!	316.0	#NULL!	#NULL!	#NULL!	#NULL!	#NULL!	#NULL!
133.0	#NULL!	#NULL!	#NULL!	#NULL!	#NULL!	#NULL!	#NULL!	#NULL!
307.0	201.0	406.0	146.0	9.10	15.2	40.5	55.70	103.0
79.0	109.0	196.0	17.0	12.30	2.1	38.1	40.20	7.1
134.0	#NULL!	306.0	#NULL!	#NULL!	14.5	24.4	39.30	182.0
250.0	206.0	225.0	138.0	16.50	#NULL!	#NULL!	#NULL!	#NULL!
96.0	#NULL!	414.0	60.9	15.00	6.8	46.7	53.00	14.5
247.0	161.0	470.0	87.6	20.90	25.3	21.2	40.60	106.0
48.0	145.0	#NULL!	#NULL!	#NULL!	#NULL!	#NULL!	#NULL!	#NULL!
177.0	166.0	277.0	92.0	15.60	4.5	51.2	55.70	7.9
122.0	187.0	325.0	324.0	16.10	#NULL!	#NULL!	#NULL!	#NULL!
237.0	#NULL!	316.0	#NULL!	#NULL!	#NULL!	#NULL!	#NULL!	#NULL!
103.0	#NULL!	335.0	55.0	10.00	11.2	36.1	40.30	66.9
155.0	171.0	356.0	130.0	14.30	#NULL!	#NULL!	#NULL!	#NULL!
66.0	#NULL!	#NULL!	#NULL!	#NULL!	#NULL!	#NULL!	#NULL!	#NULL!
87.0	#NULL!	#NULL!	#NULL!	#NULL!	#NULL!	#NULL!	#NULL!	#NULL!
194.0	228.0	410.0	231.0	16.40	10.9	43.2	53.20	164.0
84.0	#NULL!	511.0	#NULL!	#NULL!	#NULL!	#NULL!	#NULL!	#NULL!

210.0	195.0	269.0	83.5	13.00	#NULL!	#NULL!	#NULL!	#NULL!
130.0	178.0	216.0	29.0	13.90	#NULL!	#NULL!	#NULL!	#NULL!
298.0	174.0	488.0	79.6	15.90	#NULL!	#NULL!	#NULL!	#NULL!
167.0	191.0	412.0	80.4	14.60	#NULL!	#NULL!	#NULL!	#NULL!
211.0	137.0	419.0	61.3	10.40	#NULL!	#NULL!	#NULL!	#NULL!
167.0	161.0	258.0	77.1	16.10	#NULL!	#NULL!	#NULL!	#NULL!
196.0	229.0	306.0	66.6	14.30	#NULL!	#NULL!	#NULL!	#NULL!
195.0	158.0	368.0	61.7	23.90	5.2	57.4	62.60	3.0
197.0	191.0	203.0	114.0	14.90	#NULL!	#NULL!	#NULL!	#NULL!
262.0	178.0	325.0	133.0	21.20	#NULL!	#NULL!	#NULL!	#NULL!
247.0	186.0	276.0	86.5	13.20	#NULL!	#NULL!	#NULL!	#NULL!
202.0	197.0	229.0	73.2	19.60	#NULL!	#NULL!	#NULL!	#NULL!
109.0	182.0	243.0	113.0	17.00	#NULL!	#NULL!	#NULL!	#NULL!
285.0	146.0	208.0	83.0	7.00	#NULL!	#NULL!	#NULL!	#NULL!
156.9	165.0	292.2	66.8	12.20	11.7	35.4	47.10	50.8
208.5	141.0	642.6	93.1	9.00	7.8	42.1	49.60	126.0
58.7	114.0	533.1	57.0	11.10	#NULL!	#NULL!	#NULL!	#NULL!
93.0	163.0	295.3	112.0	9.90	#NULL!	#NULL!	#NULL!	#NULL!
131.0	189.0	252.0	89.0	17.50	17.8	25.8	43.60	34.9
208.0	#NULL!	427.0	80.3	14.90	15.0	48.0	63.00	77.0
112.0	168.0	462.0	76.0	16.30	#NULL!	#NULL!	#NULL!	#NULL!
195.0	189.0	282.0	74.0	20.60	11.5	29.5	41.00	106.0
109.0	175.0	254.0	212.0	11.10	11.3	39.6	50.90	63.9
195.0	171.0	282.0	41.0	22.00	18.0	30.0	48.00	35.8
95.0	#NULL!	380.0	#NULL!	#NULL!	4.7	50.0	35.20	70.9
197.0	197.0	345.0	59.8	19.10	12.9	25.2	41.00	85.8
196.0	186.0	246.0	48.9	18.00	7.9	43.6	51.00	48.0
237.0	181.0	320.0	103.0	27.00	18.0	36.0	54.00	49.0
152.0	138.0	230.0	48.5	16.80	17.8	29.0	46.80	66.9
177.0	204.0	349.0	99.7	13.40	22.1	25.3	47.00	79.0
273.0	#NULL!	287.0	#NULL!	#NULL!	#NULL!	#NULL!	#NULL!	#NULL!
181.0	164.0	340.0	76.2	17.40	9.0	45.6	54.80	25.6
133.0	185.0	320.0	85.0	15.50	20.3	33.0	53.50	113.0
176.0	171.0	302.0	56.0	16.30	19.9	24.3	44.00	81.0
191.0	207.0	296.0	79.8	13.90	19.5	28.0	47.00	106.0
163.0	196.0	341.0	59.0	14.10	15.1	39.0	54.00	56.3
218.0	85.0	253.0	85.0	16.90	#NULL!	#NULL!	#NULL!	#NULL!
198.0	76.0	232.0	76.8	9.10	#NULL!	#NULL!	#NULL!	#NULL!
132.0	207.0	297.0	91.8	15.50	9.1	37.0	46.00	107.0
216.0	153.0	278.0	63.9	10.30	22.1	32.6	54.70	29.3
308.0	#NULL!	554.0	118.0	9.80	#NULL!	#NULL!	#NULL!	#NULL!
336.0	154.0	316.0	66.0	17.70	16.6	37.6	54.20	56.4
189.0	166.0	296.0	105.0	19.50	13.6	34.4	48.00	55.6
198.0	214.0	249.0	75.6	14.20	14.8	40.9	55.70	40.4
121.0	195.0	272.0	29.0	17.00	13.2	57.0	65.00	22.6
150.0	182.0	299.0	56.0	15.10	19.9	22.0	41.90	181.0
265.0	217.0	306.0	90.0	9.70	17.5	48.1	65.60	99.8
160.0	#NULL!	301.0	69.0	23.40	14.0	39.4	53.40	34.8
137.0	#NULL!	#NULL!	#NULL!	#NULL!	#NULL!	#NULL!	#NULL!	#NULL!
172.0	191.0	291.0	64.1	21.20	11.7	31.1	43.00	40.4
145.0	121.0	#NULL!	#NULL!	#NULL!	#NULL!	#NULL!	#NULL!	#NULL!
135.0	#NULL!	#NULL!	#NULL!	#NULL!	#NULL!	#NULL!	#NULL!	#NULL!
239.0	#NULL!	213.0	#NULL!	#NULL!	22.8	33.6	56.40	61.6
243.0	210.0	318.0	69.9	9.20	#NULL!	#NULL!	#NULL!	#NULL!

197.0	#NULL!	304.0	94.3	#NULL!	#NULL!	#NULL!	#NULL!	#NULL!
148.0	#NULL!	#NULL!	#NULL!	#NULL!	#NULL!	#NULL!	#NULL!	#NULL!
316.0	146.0	363.0	66.2	13.70	#NULL!	#NULL!	#NULL!	#NULL!
154.0	129.0	#NULL!	#NULL!	#NULL!	#NULL!	#NULL!	#NULL!	#NULL!
117.0	#NULL!	276.0	29.2	15.90	#NULL!	#NULL!	#NULL!	#NULL!
60.5	146.0	248.0	44.9	5.20	3.6	42.2	45.80	82.4
211.0	#NULL!	289.0	#NULL!	#NULL!	#NULL!	#NULL!	#NULL!	#NULL!
128.0	226.0	328.0	96.9	14.70	17.7	21.0	30.00	46.7
128.0	#NULL!	#NULL!	#NULL!	#NULL!	#NULL!	#NULL!	#NULL!	#NULL!
90.0	183.0	324.0	104.0	12.80	1.9	70.6	72.50	1.6
192.0	125.0	#NULL!	#NULL!	#NULL!	#NULL!	#NULL!	#NULL!	#NULL!
54.0	#NULL!	239.0	#NULL!	#NULL!	#NULL!	#NULL!	#NULL!	#NULL!
113.7	#NULL!	412.0	45.6	15.70	#NULL!	#NULL!	#NULL!	#NULL!
166.0	#NULL!	314.0	#NULL!	#NULL!	#NULL!	#NULL!	#NULL!	#NULL!
173.0	#NULL!	#NULL!	#NULL!	#NULL!	#NULL!	#NULL!	#NULL!	#NULL!
58.0	#NULL!	155.5	#NULL!	#NULL!	#NULL!	#NULL!	#NULL!	#NULL!
179.0	156.0	#NULL!	#NULL!	#NULL!	#NULL!	#NULL!	#NULL!	#NULL!
69.0	#NULL!	#NULL!	#NULL!	#NULL!	#NULL!	#NULL!	#NULL!	#NULL!
122.0	#NULL!	341.7	#NULL!	#NULL!	#NULL!	#NULL!	#NULL!	#NULL!
157.0	#NULL!	525.0	#NULL!	#NULL!	#NULL!	#NULL!	#NULL!	#NULL!
148.0	134.0	#NULL!	#NULL!	#NULL!	#NULL!	#NULL!	#NULL!	#NULL!
107.4	#NULL!	#NULL!	#NULL!	#NULL!	#NULL!	#NULL!	#NULL!	#NULL!
152.0	#NULL!	255.2	#NULL!	#NULL!	#NULL!	#NULL!	#NULL!	#NULL!
178.0	#NULL!	272.3	#NULL!	#NULL!	#NULL!	#NULL!	#NULL!	#NULL!
362.0	#NULL!	292.0	#NULL!	#NULL!	#NULL!	#NULL!	#NULL!	#NULL!
196.0	154.0	516.0	50.2	20.50	13.2	10.2	53.40	117.0
160.5	103.0	291.1	69.2	15.80	#NULL!	#NULL!	#NULL!	#NULL!
126.3	#NULL!	283.3	#NULL!	#NULL!	#NULL!	#NULL!	#NULL!	#NULL!
290.0	215.0	274.0	56.0	17.60	#NULL!	#NULL!	#NULL!	#NULL!
113.0	#NULL!	151.5	#NULL!	#NULL!	#NULL!	#NULL!	#NULL!	#NULL!
88.6	136.0	308.8	29.2	14.70	#NULL!	#NULL!	#NULL!	#NULL!
130.0	191.0	273.6	112.3	21.00	#NULL!	#NULL!	#NULL!	#NULL!
185.0	#NULL!	#NULL!	#NULL!	#NULL!	#NULL!	#NULL!	#NULL!	#NULL!
117.2	258.0	258.8	105.7	12.00	#NULL!	#NULL!	#NULL!	#NULL!
69.0	#NULL!	363.5	#NULL!	#NULL!	#NULL!	#NULL!	#NULL!	#NULL!
100.2	170.0	252.3	76.8	11.10	#NULL!	#NULL!	#NULL!	#NULL!
238.0	171.0	360.0	85.8	14.70	#NULL!	#NULL!	#NULL!	#NULL!
101.0	117.0	236.2	45.1	7.40	3.7	55.6	59.30	28.6
201.0	184.0	346.7	105.3	12.00	#NULL!	#NULL!	#NULL!	#NULL!
89.0	121.0	305.9	28.7	12.40	#NULL!	#NULL!	#NULL!	#NULL!
60.0	207.0	360.8	73.6	26.10	#NULL!	#NULL!	#NULL!	#NULL!
161.0	179.0	299.0	79.8	16.30	#NULL!	#NULL!	#NULL!	#NULL!
393.0	216.0	430.5	344.0	18.70	17.3	31.7	49.00	46.4
200.0	149.0	465.0	63.1	12.50	#NULL!	#NULL!	#NULL!	#NULL!
171.0	178.0	233.2	67.6	32.60	#NULL!	#NULL!	#NULL!	#NULL!
193.0	180.0	355.0	78.4	14.90	#NULL!	#NULL!	#NULL!	#NULL!
169.0	168.0	351.4	50.9	9.90	#NULL!	#NULL!	#NULL!	#NULL!
163.0	151.0	239.0	39.7	15.70	#NULL!	#NULL!	#NULL!	#NULL!
231.0	241.0	257.1	56.4	19.00	#NULL!	#NULL!	#NULL!	#NULL!
88.0	114.0	233.8	45.6	15.00	#NULL!	#NULL!	#NULL!	#NULL!
182.0	#NULL!	#NULL!	#NULL!	#NULL!	#NULL!	#NULL!	#NULL!	#NULL!
219.8	192.0	420.7	72.0	15.10	#NULL!	#NULL!	#NULL!	#NULL!
173.0	#NULL!	#NULL!	#NULL!	#NULL!	3.2	68.1	71.30	4.6
179.2	145.0	375.2	42.1	10.40	#NULL!	#NULL!	#NULL!	#NULL!

278.0	198.0	256.6	66.0	17.20	#NULL!	#NULL!	#NULL!	#NULL!
152.0	179.0	210.3	77.1	32.80	#NULL!	#NULL!	#NULL!	#NULL!
203.7	167.0	291.4	85.6	15.20	#NULL!	#NULL!	#NULL!	#NULL!
195.0	167.0	336.0	86.6	17.10	#NULL!	#NULL!	#NULL!	#NULL!
294.8	194.0	256.0	92.0	19.50	#NULL!	#NULL!	#NULL!	#NULL!
72.2	160.0	209.6	45.0	11.70	2.8	22.1	24.90	52.3
109.0	190.0	312.0	47.8	14.20	#NULL!	#NULL!	#NULL!	#NULL!
154.5	189.0	417.0	86.3	18.30	#NULL!	#NULL!	#NULL!	#NULL!
220.4	132.0	278.3	83.0	14.80	#NULL!	#NULL!	#NULL!	#NULL!
177.5	170.0	305.5	66.1	18.80	#NULL!	#NULL!	#NULL!	#NULL!
165.0	#NULL!	#NULL!	#NULL!	#NULL!	#NULL!	#NULL!	#NULL!	#NULL!
201.2	131.0	261.0	78.6	12.20	#NULL!	#NULL!	#NULL!	#NULL!
241.6	221.0	270.7	157.2	21.10	#NULL!	#NULL!	#NULL!	#NULL!
278.6	233.0	294.1	179.5	15.20	#NULL!	#NULL!	#NULL!	#NULL!
126.2	#NULL!	204.0	#NULL!	#NULL!	#NULL!	#NULL!	#NULL!	#NULL!
264.0	211.0	256.6	110.3	27.80	#NULL!	#NULL!	#NULL!	#NULL!
245.3	177.0	299.9	83.5	16.20	#NULL!	#NULL!	#NULL!	#NULL!
178.8	198.0	216.6	131.0	18.30	#NULL!	#NULL!	#NULL!	#NULL!
213.0	150.0	244.8	59.9	18.40	27.8	23.7	51.60	55.0
271.0	#NULL!	435.0	#NULL!	#NULL!	#NULL!	#NULL!	#NULL!	#NULL!
207.8	#NULL!	232.0	#NULL!	#NULL!	#NULL!	#NULL!	#NULL!	#NULL!
198.0	181.0	251.1	89.9	18.00	#NULL!	#NULL!	#NULL!	#NULL!
120.0	164.0	274.6	99.0	21.70	#NULL!	#NULL!	#NULL!	#NULL!
282.0	174.0	202.3	50.6	15.30	#NULL!	#NULL!	#NULL!	#NULL!
330.9	192.0	411.6	140.8	13.70	#NULL!	#NULL!	#NULL!	#NULL!
198.0	173.0	325.0	40.9	16.70	#NULL!	#NULL!	#NULL!	#NULL!
247.2	146.0	416.7	77.5	15.90	15.7	39.4	55.10	120.3
114.0	157.0	363.8	88.5	15.70	#NULL!	#NULL!	#NULL!	#NULL!
311.0	219.0	200.8	141.2	16.20	13.3	39.7	58.00	114.5
174.1	#NULL!	135.9	#NULL!	#NULL!	#NULL!	#NULL!	#NULL!	#NULL!
267.0	180.0	354.3	73.8	20.20	#NULL!	#NULL!	#NULL!	#NULL!
258.9	193.0	406.1	67.9	23.10	#NULL!	#NULL!	#NULL!	#NULL!
213.0	#NULL!	388.1	#NULL!	#NULL!	#NULL!	#NULL!	#NULL!	#NULL!
88.1	108.0	309.0	37.7	5.00	2.4	50.3	52.70	25.7
152.0	192.0	317.1	73.3	16.70	#NULL!	#NULL!	#NULL!	#NULL!
162.0	#NULL!	#NULL!	#NULL!	#NULL!	#NULL!	#NULL!	#NULL!	#NULL!
195.0	243.0	289.8	55.8	24.50	#NULL!	#NULL!	#NULL!	#NULL!
99.0	159.0	339.3	80.8	12.20	#NULL!	#NULL!	#NULL!	#NULL!
168.0	#NULL!	#NULL!	#NULL!	#NULL!	#NULL!	#NULL!	#NULL!	#NULL!
308.1	217.0	354.9	197.6	34.80	#NULL!	#NULL!	#NULL!	#NULL!
138.3	136.0	285.0	32.2	15.20	#NULL!	#NULL!	#NULL!	#NULL!
101.2	114.0	331.6	15.8	12.70	#NULL!	#NULL!	#NULL!	#NULL!
160.1	157.0	331.6	89.2	19.70	#NULL!	#NULL!	#NULL!	#NULL!
175.2	145.0	309.4	68.4	48.10	#NULL!	#NULL!	#NULL!	#NULL!
128.0	#NULL!	#NULL!	#NULL!	#NULL!	#NULL!	#NULL!	#NULL!	#NULL!
153.8	#NULL!	348.2	43.0	#NULL!	#NULL!	#NULL!	#NULL!	#NULL!
281.1	195.0	381.5	119.2	15.90	#NULL!	#NULL!	#NULL!	#NULL!
236.3	217.0	370.1	111.6	25.20	#NULL!	#NULL!	#NULL!	#NULL!
269.6	160.0	367.8	98.3	22.70	#NULL!	#NULL!	#NULL!	#NULL!
180.4	202.0	439.1	86.8	13.00	#NULL!	#NULL!	#NULL!	#NULL!
256.0	137.0	242.4	64.2	11.80	#NULL!	#NULL!	#NULL!	#NULL!
186.8	193.0	358.4	60.9	26.20	#NULL!	#NULL!	#NULL!	#NULL!
194.7	136.0	240.8	95.5	16.30	#NULL!	#NULL!	#NULL!	#NULL!
160.2	#NULL!	218.8	#NULL!	#NULL!	#NULL!	#NULL!	#NULL!	#NULL!

110.7	157.0	191.0	20.4	14.20	#NULL!	#NULL!	#NULL!	#NULL!
323.4	144.0	290.2	70.8	15.90	#NULL!	#NULL!	#NULL!	#NULL!
224.0	148.0	298.0	90.0	15.30	#NULL!	#NULL!	#NULL!	#NULL!
108.0	#NULL!	369.4	#NULL!	#NULL!	#NULL!	#NULL!	#NULL!	#NULL!
201.6	182.0	440.0	68.6	16.10	#NULL!	#NULL!	#NULL!	#NULL!
258.6	172.0	253.3	62.0	20.10	#NULL!	#NULL!	#NULL!	#NULL!
233.1	164.0	490.0	148.0	14.00	#NULL!	#NULL!	#NULL!	#NULL!
114.5	27.0	247.5	#NULL!	#NULL!	11.2	23.4	34.60	932.0
109.1	#NULL!	322.8	#NULL!	#NULL!	#NULL!	#NULL!	#NULL!	#NULL!
235.0	198.0	284.5	102.2	16.30	3.3	36.7	40.00	193.4
242.2	136.0	361.0	77.0	15.30	6.4	57.7	64.10	6.1
190.0	146.0	400.0	36.0	13.10	12.3	44.5	56.80	54.0
138.6	208.0	361.2	55.1	13.30	18.4	23.2	41.60	141.0
62.8	161.0	279.1	36.0	12.40	4.5	33.3	37.80	93.0
339.2	228.0	371.7	93.6	22.80	13.0	41.8	54.80	98.9
249.1	248.0	250.3	117.5	25.20	#NULL!	#NULL!	#NULL!	#NULL!
107.4	161.0	431.2	124.2	10.10	#NULL!	#NULL!	#NULL!	2.0
89.6	183.0	235.4	185.1	23.70	#NULL!	#NULL!	#NULL!	#NULL!
228.8	159.0	431.4	96.7	18.60	33.6	13.1	46.70	74.6
175.9	#NULL!	203.3	#NULL!	#NULL!	#NULL!	#NULL!	#NULL!	#NULL!
229.2	252.0	226.1	139.2	36.10	18.9	38.0	56.90	21.6
259.6	148.0	477.9	74.9	14.30	11.1	44.5	55.60	103.0
318.4	208.0	320.0	82.5	14.90	14.0	39.6	53.60	61.1
137.6	130.0	298.9	45.1	10.90	23.2	39.7	62.90	50.2
312.2	229.0	428.0	89.0	24.10	#NULL!	#NULL!	#NULL!	#NULL!
188.5	181.0	331.6	90.7	11.90	#NULL!	#NULL!	#NULL!	#NULL!
75.8	#NULL!	246.0	#NULL!	#NULL!	#NULL!	#NULL!	#NULL!	#NULL!
169.5	239.0	264.2	50.1	27.00	18.3	37.0	55.30	63.6
252.3	213.0	334.8	144.8	23.30	14.7	41.1	55.80	43.7
294.0	157.0	401.0	78.0	15.80	28.2	31.5	59.70	42.2
68.4	176.0	172.4	68.6	9.40	6.6	34.1	40.60	100.1
130.9	199.0	199.0	18.0	12.10	#NULL!	#NULL!	#NULL!	#NULL!
254.2	150.0	328.9	67.2	13.70	16.4	33.7	50.10	57.4
69.1	134.0	211.3	22.6	8.10	3.0	39.9	42.90	147.3
228.6	261.0	324.2	151.1	20.80	#NULL!	#NULL!	#NULL!	#NULL!
262.9	190.0	429.0	90.3	53.90	#NULL!	#NULL!	#NULL!	#NULL!
173.2	165.0	476.0	174.0	16.00	#NULL!	#NULL!	#NULL!	#NULL!
128.5	116.0	383.6	56.0	12.40	19.0	44.4	63.40	13.7
164.4	208.0	290.7	357.6	16.80	13.8	42.0	55.80	145.9
106.3	176.0	#NULL!	#NULL!	0.90	25.1	24.1	49.20	105.8
195.6	149.0	306.0	92.0	13.40	9.3	37.8	47.10	28.9
237.8	190.0	251.5	121.3	20.00	#NULL!	#NULL!	#NULL!	#NULL!
279.1	171.0	301.8	69.5	15.70	10.0	39.0	49.00	132.2
262.6	169.0	304.4	56.2	16.20	12.2	34.6	46.80	97.2
215.5	167.0	344.0	70.0	12.80	#NULL!	#NULL!	#NULL!	#NULL!
198.6	115.0	475.6	71.5	10.30	12.8	30.1	43.90	89.1
158.6	134.0	256.7	48.9	9.90	#NULL!	#NULL!	#NULL!	#NULL!
319.7	202.0	492.5	105.8	15.10	#NULL!	#NULL!	#NULL!	#NULL!
290.6	190.0	252.1	65.4	15.00	#NULL!	#NULL!	#NULL!	#NULL!
339.9	128.0	363.6	83.0	27.10	#NULL!	#NULL!	#NULL!	#NULL!
223.3	163.0	544.3	69.7	16.70	#NULL!	#NULL!	#NULL!	#NULL!
#NULL!	#NULL!	#NULL!	#NULL!	#NULL!	#NULL!	#NULL!	#NULL!	#NULL!
192.3	165.0	321.0	77.6	21.60	#NULL!	#NULL!	#NULL!	#NULL!
298.7	223.0	250.6	79.2	13.10	#NULL!	#NULL!	#NULL!	#NULL!

#NULL!	180.0	#NULL!	#NULL!	#NULL!	#NULL!	#NULL!	#NULL!	#NULL!
#NULL!	164.0	206.0	72.0	#NULL!	#NULL!	#NULL!	#NULL!	#NULL!
219.8	#NULL!	#NULL!	#NULL!	#NULL!	#NULL!	#NULL!	#NULL!	#NULL!
#NULL!	185.0	#NULL!	#NULL!	#NULL!	#NULL!	#NULL!	#NULL!	#NULL!
139.6	151.0	340.7	87.3	10.70	14.0	29.7	43.70	60.6
255.2	220.0	259.7	101.1	20.30	7.2	37.9	45.10	69.4
169.9	155.0	374.0	42.8	16.90	#NULL!	#NULL!	#NULL!	#NULL!
40.8	181.0	173.0	43.6	20.00	16.2	43.5	59.60	25.9
207.3	184.0	356.0	60.0	11.40	#NULL!	#NULL!	#NULL!	#NULL!
154.7	196.0	223.3	102.9	17.40	#NULL!	#NULL!	#NULL!	#NULL!
276.9	188.0	216.0	49.0	14.60	#NULL!	#NULL!	#NULL!	#NULL!
163.4	171.0	412.3	52.6	6.40	#NULL!	#NULL!	#NULL!	#NULL!
231.4	215.0	311.8	66.7	22.40	19.6	36.4	56.00	140.7
304.7	167.0	232.8	65.9	19.30	#NULL!	#NULL!	#NULL!	#NULL!
165.4	165.0	342.0	77.2	11.60	3.8	69.5	73.40	6.5
278.9	182.0	327.4	72.2	11.70	#NULL!	#NULL!	#NULL!	#NULL!
192.4	184.0	305.4	55.2	16.80	#NULL!	#NULL!	#NULL!	#NULL!
295.6	188.0	360.6	66.0	26.00	#NULL!	#NULL!	#NULL!	#NULL!
116.6	135.0	366.1	74.2	11.20	#NULL!	#NULL!	#NULL!	#NULL!
133.6	162.0	276.8	42.8	14.10	#NULL!	#NULL!	#NULL!	#NULL!
190.8	184.0	254.0	93.6	12.80	#NULL!	#NULL!	#NULL!	#NULL!
143.2	135.0	216.0	67.9	11.60	#NULL!	#NULL!	#NULL!	#NULL!
255.7	227.0	294.9	130.7	35.10	28.4	39.1	67.50	51.8
91.2	113.0	155.1	22.2	3.90	#NULL!	#NULL!	#NULL!	#NULL!
194.9	142.0	317.9	85.4	19.30	#NULL!	#NULL!	#NULL!	#NULL!
201.7	#NULL!	450.7	#NULL!	#NULL!	#NULL!	#NULL!	#NULL!	#NULL!
195.3	130.0	271.3	54.7	13.50	#NULL!	#NULL!	#NULL!	#NULL!
199.9	183.0	321.4	99.1	21.70	14.7	29.2	43.90	144.2
130.2	#NULL!	#NULL!	#NULL!	#NULL!	#NULL!	#NULL!	#NULL!	#NULL!
215.4	198.0	318.5	67.5	33.00	#NULL!	#NULL!	#NULL!	#NULL!
195.4	208.0	226.5	110.4	15.30	#NULL!	#NULL!	#NULL!	#NULL!
261.2	#NULL!	#NULL!	#NULL!	#NULL!	4.0	35.0	39.00	96.6
180.5	147.0	236.2	63.4	22.20	20.7	24.8	45.50	43.2
135.6	161.0	193.4	42.7	9.20	2.0	38.6	40.60	248.1
243.0	189.0	394.0	70.5	21.00	#NULL!	#NULL!	#NULL!	#NULL!
199.9	168.0	428.0	68.0	13.00	17.8	35.0	52.80	70.3
#NULL!	#NULL!	#NULL!	#NULL!	0.50	2.9	59.2	62.10	12.1
270.0	222.0	370.0	152.0	18.90	19.2	36.0	55.20	76.8
206.5	169.0	282.8	63.6	13.20	18.7	26.1	44.80	70.1
70.3	136.0	103.3	27.0	0.40	#NULL!	#NULL!	#NULL!	#NULL!
169.6	162.0	267.0	60.0	16.00	#NULL!	#NULL!	#NULL!	#NULL!
292.9	169.0	285.2	67.5	18.20	#NULL!	#NULL!	#NULL!	#NULL!
212.5	135.0	459.3	70.2	22.10	#NULL!	#NULL!	#NULL!	#NULL!
234.9	149.0	218.0	87.0	18.20	#NULL!	#NULL!	#NULL!	#NULL!
85.7	113.0	354.2	64.0	0.50	#NULL!	#NULL!	#NULL!	#NULL!
117.7	169.0	342.0	81.0	14.20	#NULL!	#NULL!	#NULL!	#NULL!
131.5	131.5	401.0	30.2	12.70	20.5	30.5	51.00	110.5
149.0	167.0	348.0	64.0	14.70	12.6	37.8	50.40	68.9
223.9	202.0	275.8	73.9	24.10	16.2	40.2	56.40	121.7
306.8	194.0	403.3	117.7	20.20	#NULL!	#NULL!	#NULL!	#NULL!
452.5	181.0	431.0	70.0	15.80	#NULL!	#NULL!	#NULL!	#NULL!
154.0	209.0	252.0	76.0	13.00	11.0	37.2	48.20	74.0
286.9	209.0	266.0	138.0	17.40	13.7	25.8	39.50	98.5
227.8	157.0	305.0	70.0	18.40	#NULL!	#NULL!	#NULL!	#NULL!

163.7	140.0	361.3	42.4	9.30	8.8	33.6	42.40	23.5
63.6	166.0	207.0	59.0	7.50	#NULL!	#NULL!	#NULL!	#NULL!
156.2	227.0	333.3	92.6	23.40	#NULL!	#NULL!	#NULL!	#NULL!
426.7	207.0	357.0	119.0	17.10	#NULL!	#NULL!	#NULL!	#NULL!
213.6	199.0	303.6	199.0	42.00	#NULL!	#NULL!	#NULL!	#NULL!
117.6	#NULL!	#NULL!	#NULL!	#NULL!	#NULL!	#NULL!	#NULL!	#NULL!
178.9	218.0	496.2	58.2	39.60	#NULL!	#NULL!	#NULL!	#NULL!
342.7	229.0	275.7	87.2	18.90	#NULL!	#NULL!	#NULL!	#NULL!
#NULL!	#NULL!	#NULL!	#NULL!	#NULL!	#NULL!	#NULL!	#NULL!	#NULL!
315.4	225.9	162.0	#NULL!	#NULL!	57.0	15.0	72.00	19.3
228.5	202.0	270.2	93.0	23.40	18.7	30.5	49.20	142.5
302.8	245.0	329.0	94.0	32.80	23.0	26.1	49.10	39.4
248.2	169.0	302.0	41.0	14.30	#NULL!	#NULL!	#NULL!	#NULL!
140.7	123.0	264.5	55.0	1.80	31.6	6.8	38.40	20.3
205.4	227.0	379.0	165.7	21.80	#NULL!	#NULL!	#NULL!	#NULL!
176.1	174.0	269.6	72.8	14.60	5.5	44.1	49.60	85.0
130.4	128.0	353.7	56.9	9.50	15.3	22.9	38.20	67.2
189.4	168.0	184.9	118.2	24.90	16.8	21.9	38.80	164.6
265.0	156.0	391.9	49.8	15.10	#NULL!	#NULL!	#NULL!	#NULL!
250.2	#NULL!	300.4	#NULL!	#NULL!	9.2	34.4	43.60	74.9
247.0	207.0	334.3	70.9	11.60	13.5	35.2	48.60	39.6
110.3	135.0	262.1	88.4	11.20	16.3	44.1	60.40	49.2
219.1	209.0	323.1	81.0	20.60	13.0	33.6	46.60	30.2
193.8	141.0	397.0	101.0	12.10	13.9	38.5	52.40	55.5
245.8	152.0	359.0	64.2	18.60	18.0	45.0	63.00	72.3
228.9	140.0	668.0	95.1	8.60	21.2	35.5	56.70	76.8
208.2	184.0	236.4	58.4	21.10	#NULL!	#NULL!	#NULL!	#NULL!
142.7	164.0	252.7	70.8	14.80	30.3	28.2	58.50	52.1
180.5	245.0	544.4	49.6	7.50	13.6	31.0	44.60	169.9
80.5	114.0	344.2	44.7	11.60	20.0	32.7	52.70	42.4
265.3	191.0	307.1	99.6	15.50	#NULL!	#NULL!	#NULL!	#NULL!
236.5	236.0	286.4	113.2	12.20	12.4	36.3	48.70	189.9
157.8	119.0	285.5	57.8	9.60	10.3	56.7	67.00	55.3
341.7	#NULL!	310.2	#NULL!	#NULL!	#NULL!	#NULL!	#NULL!	#NULL!
186.7	226.0	424.4	129.0	25.90	#NULL!	#NULL!	#NULL!	#NULL!
62.6	#NULL!	301.7	#NULL!	#NULL!	#NULL!	#NULL!	#NULL!	#NULL!
289.4	172.0	271.0	99.9	14.10	13.8	55.1	68.90	54.0
110.0	134.0	362.9	43.1	6.90	19.3	36.9	56.20	28.4
146.9	190.0	193.0	96.0	18.60	8.9	43.2	52.10	99.1
232.5	240.0	348.9	57.3	22.30	#NULL!	#NULL!	#NULL!	#NULL!
70.7	148.0	247.4	45.3	10.70	#NULL!	#NULL!	#NULL!	#NULL!
189.7	214.0	312.0	56.0	15.70	#NULL!	#NULL!	#NULL!	#NULL!
186.2	204.0	389.5	84.8	11.30	11.6	53.4	65.00	51.5
420.5	220.0	207.9	134.2	15.60	15.5	38.5	54.00	129.9
162.8	200.0	406.8	62.0	0.80	8.1	29.7	37.80	143.6
162.4	#NULL!	378.3	122.0	1.20	#NULL!	#NULL!	#NULL!	#NULL!
149.3	163.0	404.7	73.5	11.30	10.4	56.2	66.60	22.4
118.5	185.0	404.4	#NULL!	#NULL!	#NULL!	#NULL!	#NULL!	#NULL!
195.2	184.0	375.3	222.6	19.20	17.1	40.0	57.10	70.2
187.6	159.0	190.3	54.2	14.10	7.6	31.4	39.00	106.5
177.0	147.0	200.1	61.5	11.30	21.2	24.2	45.40	79.4
171.8	179.0	215.6	86.9	28.20	15.7	37.7	53.40	103.1
137.8	210.0	316.2	68.4	23.20	2.6	67.7	70.30	4.8
79.0	177.0	251.5	51.9	12.30	11.6	33.1	44.70	117.8

229.8	211.0	377.0	85.6	11.40	17.8	38.2	56.00	120.6
255.6	213.0	249.2	104.9	16.40	16.9	36.9	53.80	185.3
274.2	155.0	249.1	60.7	15.70	16.9	36.3	53.20	39.9
85.3	132.0	342.0	42.1	54.00	#NULL!	#NULL!	#NULL!	#NULL!
174.0	168.0	230.9	92.5	13.00	12.6	32.0	44.60	149.7
232.2	198.0	476.3	69.8	14.20	21.0	36.2	57.20	153.6
160.4	147.0	317.7	25.1	18.10	#NULL!	#NULL!	#NULL!	#NULL!
138.7	142.0	250.1	61.4	15.00	19.1	24.6	43.70	123.2
88.0	291.0	812.1	16.1	9.40	31.0	14.7	45.70	2551.9
100.1	#NULL!	173.1	59.0	0.70	#NULL!	#NULL!	#NULL!	#NULL!
231.6	187.0	389.7	87.6	21.50	29.5	23.0	52.50	98.8
267.7	228.0	270.2	125.2	17.30	19.8	30.7	50.50	133.9
324.4	161.0	275.1	89.8	10.30	11.4	40.3	51.70	98.3
236.6	270.0	237.0	151.1	26.30	19.6	39.5	59.10	84.7
197.9	191.0	279.9	66.1	11.40	3.1	39.9	43.00	20.7
133.7	174.0	186.0	48.6	21.10	1.9	71.2	73.10	1.8
279.9	184.0	298.1	79.2	13.30	#NULL!	#NULL!	#NULL!	#NULL!
173.8	193.0	277.7	115.7	13.80	12.2	31.0	43.20	79.5
257.0	221.0	255.0	85.0	18.10	11.0	34.6	45.60	92.9
227.4	245.0	259.0	100.0	22.30	#NULL!	#NULL!	#NULL!	#NULL!
182.2	178.0	310.0	50.0	16.30	15.8	34.4	50.20	13.1
125.7	101.0	430.5	61.0	0.80	#NULL!	#NULL!	#NULL!	#NULL!
95.5	171.0	379.5	75.9	13.10	17.0	36.5	53.50	33.1
151.0	134.0	333.1	75.6	13.30	8.6	52.8	61.40	41.9
201.7	#NULL!	252.3	84.0	1.20	2.6	52.3	54.90	24.1
185.7	180.0	404.7	36.9	11.50	7.4	36.1	43.60	90.6
288.4	229.0	331.1	120.4	10.70	#NULL!	#NULL!	#NULL!	#NULL!
246.9	151.0	323.3	105.1	15.50	7.0	34.4	41.40	98.3
186.2	190.0	251.4	59.9	11.80	10.5	32.6	43.10	164.1
159.8	172.0	215.4	84.8	15.20	23.7	20.0	43.70	123.6
210.6	#NULL!	213.1	#NULL!	#NULL!	#NULL!	#NULL!	#NULL!	#NULL!
275.7	#NULL!	282.1	#NULL!	#NULL!	#NULL!	#NULL!	#NULL!	#NULL!
204.8	160.0	323.9	80.3	15.10	14.5	33.1	47.60	90.6
202.7	224.0	332.4	90.8	13.50	13.8	53.6	67.40	64.5
92.2	146.0	#NULL!	#NULL!	#NULL!	#NULL!	#NULL!	#NULL!	#NULL!
207.7	238.0	377.9	89.6	13.70	26.9	27.3	54.20	83.7
202.8	183.0	299.0	59.0	20.70	#NULL!	#NULL!	#NULL!	#NULL!
164.5	170.0	580.3	87.0	10.80	14.2	49.4	63.60	67.7
184.3	135.0	318.1	96.3	10.30	21.3	30.9	52.20	72.5
209.5	264.0	171.2	111.7	24.90	22.4	36.8	59.20	105.4
#NULL!	205.0	#NULL!	44.0	#NULL!	#NULL!	#NULL!	#NULL!	#NULL!
308.2	220.0	316.2	143.0	18.10	12.8	35.3	48.10	127.4
304.5	200.0	357.8	186.4	17.30	16.7	40.7	57.40	128.6
249.6	331.1	249.6	103.0	18.30	#NULL!	#NULL!	#NULL!	#NULL!
199.0	224.0	293.0	108.0	23.00	#NULL!	#NULL!	#NULL!	#NULL!
156.4	227.0	491.7	96.1	13.10	#NULL!	#NULL!	#NULL!	#NULL!
239.2	330.0	418.4	61.6	22.80	#NULL!	#NULL!	#NULL!	#NULL!
215.5	172.0	287.2	83.8	30.10	13.4	29.2	42.60	158.0
314.6	230.0	419.4	96.1	21.20	11.7	47.2	58.90	22.1
188.1	164.0	426.8	73.7	15.00	6.7	46.9	53.60	29.2
128.9	231.0	315.1	109.5	21.30	8.5	53.0	61.50	31.3
220.9	180.0	233.7	161.2	19.60	10.0	50.8	60.80	72.1
272.5	143.0	371.4	62.0	12.40	16.3	34.7	51.00	105.1
307.6	190.0	426.2	130.4	16.20	19.1	36.1	55.20	24.8

515.7	515.7	451.5	95.7	20.80	20.3	40.7	61.00	15.2
135.6	#NULL!	348.8	#NULL!	#NULL!	#NULL!	#NULL!	#NULL!	#NULL!
179.8	153.0	295.5	59.2	15.80	#NULL!	#NULL!	#NULL!	#NULL!
177.9	211.0	167.5	105.5	20.20	21.9	36.5	58.40	134.4
312.0	154.0	293.0	129.5	17.70	#NULL!	#NULL!	#NULL!	#NULL!
314.9	193.0	453.0	90.0	13.20	22.4	40.1	62.50	94.9
422.9	185.0	527.0	124.0	13.50	#NULL!	#NULL!	#NULL!	#NULL!
#NULL!	#NULL!	#NULL!	#NULL!	#NULL!	#NULL!	#NULL!	#NULL!	#NULL!
264.5	214.0	363.0	180.0	14.20	17.8	39.9	57.70	52.7
247.2	186.0	326.0	186.0	15.50	15.2	48.6	63.80	22.1
420.5	174.0	331.9	94.6	17.10	19.6	46.6	66.20	17.5
445.5	169.0	244.8	88.7	19.90	#NULL!	#NULL!	#NULL!	#NULL!
300.7	189.0	396.2	107.9	16.40	#NULL!	#NULL!	#NULL!	#NULL!
409.8	176.0	291.7	177.7	16.20	#NULL!	#NULL!	#NULL!	#NULL!
185.5	212.0	463.0	64.1	15.40	15.4	44.7	60.10	75.7
228.7	186.0	416.3	82.6	23.60	#NULL!	#NULL!	#NULL!	#NULL!
258.9	185.0	328.8	71.5	15.60	#NULL!	#NULL!	#NULL!	#NULL!
54.5	238.0	336.7	16.3	3.60	5.3	77.8	83.10	4.3
165.5	156.0	413.7	67.1	11.90	#NULL!	#NULL!	#NULL!	#NULL!
233.5	151.0	350.6	71.9	17.10	7.1	55.4	62.50	32.4
233.6	134.0	358.5	101.0	16.60	#NULL!	#NULL!	#NULL!	#NULL!
154.3	201.0	424.6	84.1	13.10	#NULL!	#NULL!	#NULL!	#NULL!
257.2	222.0	343.2	21.0	11.80	#NULL!	#NULL!	#NULL!	#NULL!
173.1	177.0	261.9	55.3	16.70	#NULL!	#NULL!	#NULL!	#NULL!
207.9	144.0	233.3	71.1	16.60	#NULL!	#NULL!	#NULL!	#NULL!
185.2	179.0	307.0	67.7	15.80	10.9	41.8	52.70	139.4
332.7	193.0	327.0	78.0	1.10	3.6	58.9	62.50	11.0
217.1	183.0	256.2	53.9	17.80	#NULL!	#NULL!	#NULL!	#NULL!
#NULL!	226.0	#NULL!	178.0	2.20	#NULL!	#NULL!	#NULL!	#NULL!
240.2	181.0	293.3	88.8	17.40	#NULL!	#NULL!	#NULL!	#NULL!
281.5	242.0	273.3	138.5	21.30	#NULL!	#NULL!	#NULL!	#NULL!
170.7	170.0	404.6	69.8	21.00	#NULL!	#NULL!	#NULL!	#NULL!
134.0	209.0	372.9	30.0	17.10	#NULL!	#NULL!	#NULL!	#NULL!
172.1	182.0	321.0	57.0	16.30	#NULL!	#NULL!	#NULL!	#NULL!
335.8	176.0	271.0	96.7	13.00	#NULL!	#NULL!	#NULL!	#NULL!
112.2	189.0	209.6	70.3	70.00	10.5	32.7	43.20	124.4
172.0	212.0	360.3	95.3	20.60	#NULL!	#NULL!	#NULL!	#NULL!
269.2	142.0	329.8	97.3	22.00	#NULL!	#NULL!	#NULL!	#NULL!
191.3	198.0	375.7	94.1	17.00	17.7	47.7	65.40	106.8
144.4	144.0	312.2	58.9	13.00	#NULL!	#NULL!	#NULL!	#NULL!
248.3	159.0	283.5	90.5	13.40	#NULL!	#NULL!	#NULL!	#NULL!
74.6	3739.0	413.7	56.4	17.30	32.8	1.7	34.50	588.1
216.8	151.0	300.1	140.5	19.40	18.1	35.0	53.10	75.3
216.6	154.0	307.1	95.8	28.00	#NULL!	#NULL!	#NULL!	#NULL!
169.7	206.0	418.8	40.6	28.50	#NULL!	#NULL!	#NULL!	#NULL!
185.9	203.0	271.1	61.1	15.40	#NULL!	#NULL!	#NULL!	#NULL!
170.7	172.0	245.8	65.7	30.30	#NULL!	#NULL!	#NULL!	#NULL!
114.1	#NULL!	#NULL!	61.0	0.60	#NULL!	#NULL!	#NULL!	#NULL!
50.4	208.0	394.5	66.0	0.60	#NULL!	#NULL!	#NULL!	#NULL!
71.3	153.0	414.3	33.0	8.00	#NULL!	#NULL!	#NULL!	#NULL!
234.6	183.0	469.8	88.7	14.40	#NULL!	#NULL!	#NULL!	#NULL!
200.6	202.0	128.2	66.8	18.80	#NULL!	#NULL!	#NULL!	#NULL!
303.4	177.0	293.3	168.3	23.40	#NULL!	#NULL!	#NULL!	#NULL!
252.9	144.0	292.0	66.4	14.90	#NULL!	#NULL!	#NULL!	#NULL!

107.4	165.0	311.8	114.1	13.10	#NULL!	#NULL!	#NULL!	#NULL!
199.2	219.0	303.4	83.6	27.60	#NULL!	#NULL!	#NULL!	#NULL!
138.0	158.0	282.4	57.5	16.50	14.0	19.5	33.50	210.0
103.8	220.0	495.0	39.8	15.40	12.8	20.0	32.80	158.1
304.5	269.0	357.3	77.4	27.70	#NULL!	#NULL!	#NULL!	#NULL!
200.0	205.0	344.7	65.7	20.90	28.0	20.6	48.60	25.8
212.6	194.0	463.2	95.9	14.90	#NULL!	#NULL!	#NULL!	#NULL!
127.9	179.0	377.7	32.6	14.80	#NULL!	#NULL!	#NULL!	#NULL!
174.1	203.0	582.1	109.0	1.20	#NULL!	#NULL!	#NULL!	#NULL!
283.0	196.0	385.7	89.4	22.90	#NULL!	#NULL!	#NULL!	#NULL!
400.3	216.0	311.9	85.9	18.50	#NULL!	#NULL!	#NULL!	#NULL!
223.4	228.0	284.5	105.2	24.20	#NULL!	#NULL!	#NULL!	#NULL!
158.8	149.0	299.0	63.7	15.70	#NULL!	#NULL!	#NULL!	#NULL!
327.1	216.0	470.3	125.2	14.80	13.4	32.5	45.90	99.2
279.9	189.0	302.0	88.0	13.30	15.1	22.2	37.30	153.9
145.4	167.0	206.0	68.0	14.00	9.5	45.2	54.70	14.0
295.9	178.0	499.0	79.9	14.80	24.8	23.2	48.00	67.1
260.6	206.0	337.7	51.0	13.60	#NULL!	#NULL!	#NULL!	#NULL!
177.8	234.0	348.0	47.5	15.50	#NULL!	#NULL!	#NULL!	#NULL!
231.3	160.0	247.3	49.9	14.40	7.8	41.0	48.80	83.9
96.9	150.0	193.3	69.9	8.90	3.1	50.3	53.40	5.5
159.1	133.0	328.8	85.2	21.00	27.1	23.9	51.00	75.5
224.6	192.0	321.5	145.0	1.00	#NULL!	#NULL!	#NULL!	#NULL!
#NULL!	183.0	#NULL!	85.0	0.80	#NULL!	#NULL!	#NULL!	#NULL!
280.8	177.0	235.8	67.0	12.40	21.2	33.4	54.60	84.5
234.9	158.0	299.0	91.7	14.10	22.1	29.0	51.10	101.8
312.8	139.0	449.5	125.9	12.30	4.0	46.5	50.50	20.5
186.5	175.0	280.4	86.0	18.60	#NULL!	#NULL!	#NULL!	#NULL!
196.9	174.0	315.4	83.5	25.40	#NULL!	#NULL!	#NULL!	#NULL!
183.2	217.0	257.0	76.0	21.60	15.3	35.3	50.60	103.8
#NULL!	#NULL!	#NULL!	91.0	0.70	#NULL!	#NULL!	#NULL!	#NULL!
367.3	197.0	276.0	79.0	24.80	12.8	41.5	54.30	38.8
224.5	#NULL!	394.9	119.0	1.50	#NULL!	#NULL!	#NULL!	#NULL!
244.6	172.0	367.7	81.6	18.90	#NULL!	#NULL!	#NULL!	#NULL!
171.3	153.0	389.1	79.3	14.80	10.2	43.1	53.30	21.1
199.7	204.0	524.2	116.1	19.70	#NULL!	#NULL!	#NULL!	#NULL!
257.5	174.0	361.6	78.0	13.90	#NULL!	#NULL!	#NULL!	#NULL!
103.9	156.0	360.5	23.2	12.40	6.5	37.4	43.90	152.3
266.3	238.0	268.9	113.1	31.20	#NULL!	#NULL!	#NULL!	#NULL!
226.0	175.0	348.3	81.0	1.30	#NULL!	#NULL!	#NULL!	#NULL!
189.0	145.0	264.3	73.8	17.70	#NULL!	#NULL!	#NULL!	#NULL!
163.8	#NULL!	380.4	#NULL!	2.30	#NULL!	#NULL!	#NULL!	#NULL!
196.4	191.0	401.0	68.0	26.50	#NULL!	#NULL!	#NULL!	#NULL!
126.3	197.0	457.7	98.7	9.60	20.2	43.5	63.70	32.8
#NULL!	160.0	457.8	64.2	17.40	24.5	29.2	53.70	66.9
254.9	164.0	407.6	121.0	21.60	#NULL!	#NULL!	#NULL!	#NULL!
105.6	139.0	419.2	63.2	10.10	#NULL!	#NULL!	#NULL!	#NULL!
299.5	230.0	232.2	87.0	14.20	8.9	43.3	52.20	91.1
150.1	151.0	427.3	55.7	10.60	#NULL!	#NULL!	#NULL!	#NULL!
112.3	103.0	118.0	#NULL!	0.10	#NULL!	#NULL!	#NULL!	#NULL!
327.2	197.0	281.3	67.4	27.90	#NULL!	#NULL!	#NULL!	#NULL!
95.2	165.0	370.3	67.9	22.40	#NULL!	#NULL!	#NULL!	#NULL!
257.0	201.0	305.1	94.1	17.40	#NULL!	#NULL!	#NULL!	#NULL!
201.9	193.0	370.2	109.1	14.60	#NULL!	#NULL!	#NULL!	#NULL!

327.7	219.0	377.4	114.4	32.40	#NULL!	#NULL!	#NULL!	#NULL!
219.7	206.0	243.2	85.7	16.40	#NULL!	#NULL!	#NULL!	#NULL!
341.3	171.0	371.9	90.9	10.90	#NULL!	#NULL!	#NULL!	#NULL!
258.2	181.0	472.8	125.4	15.50	#NULL!	#NULL!	#NULL!	#NULL!
212.6	157.0	240.3	62.4	18.00	#NULL!	#NULL!	#NULL!	#NULL!
201.5	214.0	235.0	75.7	27.90	20.3	25.9	46.20	25.1
432.1	230.0	275.3	70.5	21.20	#NULL!	#NULL!	#NULL!	#NULL!
167.3	169.0	338.1	79.4	17.90	#NULL!	#NULL!	#NULL!	#NULL!
242.5	163.0	365.0	62.0	24.60	#NULL!	#NULL!	#NULL!	#NULL!
190.7	280.0	388.5	62.0	18.40	10.1	33.4	43.50	122.2
102.1	122.0	306.3	43.2	12.60	#NULL!	#NULL!	#NULL!	#NULL!
284.3	200.0	263.3	89.0	24.60	#NULL!	#NULL!	#NULL!	#NULL!
153.5	167.0	254.0	38.1	12.90	#NULL!	#NULL!	#NULL!	#NULL!
270.8	172.0	366.6	86.7	19.90	10.5	41.9	52.40	116.4
280.8	204.0	196.2	99.2	23.60	#NULL!	#NULL!	#NULL!	#NULL!
179.9	249.0	554.0	75.6	46.80	#NULL!	#NULL!	#NULL!	#NULL!
227.2	158.0	435.8	88.2	19.20	17.0	34.8	51.80	88.5
438.2	219.0	370.6	73.0	27.60	#NULL!	#NULL!	#NULL!	#NULL!
152.4	111.0	527.5	43.7	8.30	11.1	39.6	50.70	135.1
260.6	195.0	294.8	105.5	15.90	17.2	37.0	54.20	57.1
216.6	200.0	356.4	69.0	20.60	14.2	39.5	53.70	134.6
169.9	143.0	300.0	33.1	11.40	21.2	35.1	56.30	36.9
262.3	207.0	339.9	66.8	17.90	5.6	46.6	52.20	131.3
303.6	170.0	278.8	66.4	17.50	15.8	31.8	47.60	119.1
228.6	168.0	382.3	60.1	10.60	20.6	28.0	48.60	87.1
#NULL!	#NULL!	#NULL!	113.0	1.70	#NULL!	#NULL!	#NULL!	#NULL!
163.4	#NULL!	#NULL!	#NULL!	#NULL!	4.2	62.7	66.90	7.7
126.4	137.0	320.9	32.8	9.30	3.1	43.5	46.60	55.3
213.4	199.0	258.4	83.7	13.90	17.5	37.6	55.10	74.8
247.6	191.0	242.9	94.6	11.80	13.9	29.1	43.00	148.0
130.2	123.0	163.3	17.2	14.40	8.3	8.7	17.00	108.4
209.4	180.0	295.2	77.4	13.30	#NULL!	#NULL!	#NULL!	#NULL!
228.5	171.0	284.9	86.6	12.40	#NULL!	#NULL!	#NULL!	#NULL!
443.1	211.0	346.2	88.9	14.90	#NULL!	#NULL!	#NULL!	#NULL!
315.2	156.0	311.2	96.9	18.70	#NULL!	#NULL!	#NULL!	#NULL!
155.2	184.0	373.8	67.6	23.10	#NULL!	#NULL!	#NULL!	#NULL!
146.9	252.0	298.2	150.0	23.60	#NULL!	#NULL!	#NULL!	#NULL!
282.0	168.0	350.4	45.5	13.80	3.6	78.6	82.20	2.3
131.0	164.0	244.0	43.0	13.60	9.6	34.4	44.00	88.2
#NULL!	255.0	#NULL!	#NULL!	#NULL!	#NULL!	#NULL!	#NULL!	#NULL!
380.0	179.0	645.3	54.2	13.70	#NULL!	#NULL!	#NULL!	#NULL!
235.2	189.0	292.0	73.0	19.80	11.8	30.4	42.20	78.4
304.9	175.0	415.6	56.5	15.50	5.6	40.5	46.10	110.0
247.6	#NULL!	#NULL!	#NULL!	#NULL!	#NULL!	#NULL!	#NULL!	#NULL!
154.4	229.0	171.6	189.3	4.80	13.6	40.8	54.40	24.2
226.6	179.0	426.2	69.5	15.10	14.0	43.5	57.50	90.3
221.7	201.0	411.6	90.6	12.90	14.9	46.8	61.70	138.4
105.6	112.0	376.0	38.8	8.80	12.4	41.9	54.30	40.5
457.5	179.0	561.4	68.7	15.50	14.0	41.4	55.10	234.8
100.3	183.0	455.7	106.2	12.20	23.5	36.4	59.90	24.6
364.5	182.0	560.1	121.0	15.00	9.8	44.3	54.10	31.4
89.3	141.0	436.4	67.2	10.40	#NULL!	#NULL!	#NULL!	#NULL!
196.4	169.0	408.8	49.5	14.80	#NULL!	#NULL!	#NULL!	#NULL!
250.7	207.0	258.0	141.4	26.10	#NULL!	#NULL!	#NULL!	#NULL!

249.0	193.0	323.6	93.3	18.80	16.3	36.9	53.20	57.6
242.4	194.0	294.8	80.3	23.40	16.4	35.3	51.70	132.4
302.3	191.0	342.9	76.7	15.90	16.0	39.0	55.00	48.7
#NULL!	#NULL!	288.0	97.0	2.30	2.0	37.8	39.80	8.1
94.0	128.0	240.1	66.3	0.30	8.7	46.5	55.20	31.7
264.5	203.0	373.0	77.0	21.20	#NULL!	#NULL!	#NULL!	#NULL!
170.5	190.0	307.7	90.1	25.00	11.4	40.2	51.60	56.9
120.4	186.0	436.0	128.0	1.60	#NULL!	#NULL!	#NULL!	#NULL!
252.2	212.0	260.4	119.3	20.40	33.5	19.9	53.40	45.0
202.5	348.0	471.4	25.3	9.50	36.0	11.2	47.20	76.7
221.5	149.0	344.3	35.6	17.20	11.8	40.2	52.00	75.0
148.8	220.0	308.2	57.6	10.60	#NULL!	#NULL!	#NULL!	#NULL!
426.6	178.0	381.1	63.5	15.60	#NULL!	#NULL!	#NULL!	#NULL!
364.8	182.0	292.3	74.4	13.70	14.3	35.9	50.20	51.0
266.2	229.0	421.0	78.9	22.30	#NULL!	#NULL!	#NULL!	#NULL!
176.1	169.0	243.4	60.0	12.40	#NULL!	#NULL!	#NULL!	#NULL!
202.0	166.0	460.5	61.0	0.80	#NULL!	#NULL!	#NULL!	#NULL!
324.0	166.0	364.4	103.1	31.80	#NULL!	#NULL!	#NULL!	#NULL!
168.4	215.0	268.8	57.1	18.30	#NULL!	#NULL!	#NULL!	#NULL!
337.1	234.0	272.6	172.4	29.70	#NULL!	#NULL!	#NULL!	#NULL!
190.4	126.0	459.7	76.9	13.30	18.6	42.0	60.60	85.2
283.0	182.0	452.7	113.6	19.60	13.6	48.3	61.90	80.7
335.6	176.0	313.4	125.9	21.20	14.1	41.6	55.70	32.6
219.0	167.0	262.0	887.0	29.10	11.5	31.5	43.00	68.1
150.6	214.0	249.9	42.3	15.70	18.5	20.8	39.30	187.7
145.0	165.0	409.9	53.4	9.10	20.2	40.4	60.60	39.7
235.7	167.0	359.5	76.7	20.30	17.8	32.7	50.50	93.6
191.9	206.0	264.7	53.0	14.90	23.1	13.9	37.00	85.6
197.7	193.0	390.0	85.7	22.60	15.1	49.0	64.10	51.7
176.9	236.0	285.3	83.0	17.40	25.3	29.6	54.90	174.3
366.2	198.0	269.1	86.7	21.60	10.9	38.3	49.20	108.0
296.1	262.0	252.0	141.3	25.10	17.9	41.5	59.40	115.1
318.6	228.0	328.2	109.3	21.70	27.1	32.6	59.70	150.9
203.4	172.0	295.0	128.0	18.90	16.6	26.9	43.50	93.7
187.7	233.0	391.2	92.1	15.70	19.1	34.4	53.50	156.9
175.5	247.0	249.9	121.9	15.30	16.7	27.5	44.20	63.5
200.1	190.0	269.0	70.9	32.90	18.9	39.4	58.30	43.5
209.9	224.0	315.4	120.5	15.50	13.7	40.2	53.90	65.3
204.4	302.0	328.0	2909.0	17.30	#NULL!	#NULL!	#NULL!	#NULL!
211.7	#NULL!	173.6	#NULL!	#NULL!	11.4	33.9	45.30	120.6
#NULL!	#NULL!	#NULL!	#NULL!	#NULL!	#NULL!	#NULL!	#NULL!	#NULL!
380.7	178.0	249.7	73.5	11.70	21.6	24.1	45.70	125.2
332.0	#NULL!	440.9	#NULL!	#NULL!	9.9	44.8	54.70	89.2
409.5	194.0	418.3	99.9	30.20	31.7	14.0	45.70	135.9
255.4	194.0	294.4	64.6	13.30	10.6	45.2	55.80	97.4
250.7	183.0	350.0	75.8	22.30	26.3	29.6	55.90	142.6
307.5	191.0	244.6	78.9	25.70	8.0	36.8	44.80	58.0
224.4	165.0	408.9	80.0	12.40	#NULL!	#NULL!	#NULL!	#NULL!
278.3	236.0	260.7	126.8	17.60	10.2	33.5	43.70	43.3
205.5	213.0	281.3	109.8	25.50	8.4	32.2	40.60	100.8
373.0	199.0	337.3	115.9	16.90	11.8	33.1	44.90	74.3
237.5	154.0	319.3	103.5	21.90	17.9	21.5	39.40	43.9
280.1	180.0	275.9	95.7	34.60	8.4	28.8	37.20	124.9
183.0	174.0	464.5	68.1	11.50	16.8	35.7	52.50	109.9

75.5	253.0	258.6	75.0	32.20	7.0	1.3	8.30	858.3
157.5	239.0	258.9	61.9	13.70	9.8	45.1	54.90	53.5
252.8	248.0	313.3	67.1	25.20	16.4	25.9	42.30	57.1
422.6	180.0	602.4	65.3	10.80	31.1	16.4	47.60	32.4
286.3	240.0	356.3	77.3	30.10	7.9	44.8	52.70	136.6
238.5	231.0	311.6	166.6	30.40	11.9	36.0	47.90	57.1
254.4	214.0	385.3	81.8	20.20	10.6	38.4	49.00	57.8
349.7	184.0	308.7	90.4	16.90	21.0	33.8	54.80	86.6
151.5	130.0	323.2	41.4	10.90	22.1	36.6	58.70	53.9
117.0	178.0	432.0	65.0	0.40	#NULL!	#NULL!	#NULL!	#NULL!
257.1	253.0	323.9	134.1	18.30	9.6	54.4	64.00	17.2
117.3	216.0	517.7	47.4	10.50	16.6	25.3	41.90	202.5
328.1	226.0	261.3	118.6	22.00	25.8	11.9	37.70	48.3
178.5	190.0	338.8	36.6	16.90	13.7	39.7	53.40	59.0
165.3	197.0	375.3	77.1	20.20	8.8	45.7	54.50	63.1
177.8	215.0	261.0	518.0	33.90	13.4	30.7	44.10	71.8
201.9	149.0	447.1	35.7	13.30	23.3	36.0	59.30	105.4
304.2	225.0	418.4	83.5	15.10	11.7	44.5	56.20	62.9
98.4	178.0	237.0	40.3	13.90	15.9	31.6	47.50	80.8
185.8	199.0	279.8	49.0	10.40	#NULL!	#NULL!	#NULL!	#NULL!
260.9	207.0	406.9	84.9	18.70	17.3	27.3	44.60	74.0
164.4	264.0	307.1	103.0	0.60	18.9	10.1	29.00	160.2
188.0	#NULL!	340.5	#NULL!	#NULL!	#NULL!	#NULL!	#NULL!	#NULL!
131.5	158.0	348.7	67.5	11.90	16.3	34.8	51.10	59.6
265.8	190.0	160.9	144.6	17.90	23.6	32.8	56.40	76.5
243.2	196.0	212.3	47.1	14.10	9.5	53.3	62.80	75.9
78.9	166.0	640.8	47.8	8.90	7.6	40.0	47.60	160.5
210.3	146.0	367.0	69.1	10.70	23.4	22.0	45.40	76.5
212.6	245.0	293.9	94.8	24.50	10.7	34.2	44.90	56.1
223.2	229.0	323.9	115.9	24.00	19.6	38.2	57.80	57.0
213.6	244.0	311.7	90.4	28.00	15.5	31.2	46.70	74.2
304.3	198.0	343.8	109.4	22.90	15.2	42.0	57.20	114.9
238.2	194.0	292.0	96.0	12.70	26.7	18.2	44.90	252.5
177.8	153.0	409.0	61.0	12.30	17.6	35.3	52.90	38.7
209.4	1602.0	401.0	42.7	22.40	#NULL!	#NULL!	#NULL!	#NULL!
186.6	228.0	318.0	114.3	20.10	9.3	31.2	40.50	44.6
322.6	224.0	289.5	87.6	15.70	22.1	21.7	43.80	125.8
273.6	158.0	320.0	55.0	11.40	9.7	24.9	34.60	148.5
256.7	181.0	381.2	70.0	25.20	23.3	31.2	54.50	71.2
189.3	209.0	317.9	59.9	12.30	9.6	30.9	40.50	160.5
103.9	156.0	360.5	23.2	12.40	6.5	37.4	43.90	152.3
113.3	151.0	223.3	14.8	8.30	13.2	31.4	44.60	92.0
229.8	278.0	341.8	110.9	23.00	7.2	41.2	48.40	129.9
#NULL!	223.0	#NULL!	77.0	0.30	#NULL!	#NULL!	#NULL!	#NULL!
334.0	190.0	239.8	67.6	17.30	17.6	34.7	52.30	94.3
305.1	161.0	342.8	87.2	12.20	#NULL!	#NULL!	#NULL!	#NULL!
318.9	129.0	399.0	61.5	11.30	10.8	40.8	51.60	47.7
156.2	259.0	201.5	145.8	35.70	23.1	29.3	52.40	144.8
172.8	205.0	399.0	65.9	14.40	23.2	31.9	55.10	97.8
133.6	159.0	280.1	24.9	8.10	4.6	46.0	50.60	163.9
164.2	249.0	296.8	67.1	18.90	22.0	31.1	53.10	71.5
189.3	221.0	256.0	83.0	7.60	6.0	37.4	43.40	70.1
248.5	178.0	258.5	66.4	20.10	11.2	46.5	57.70	75.4
205.0	247.0	210.6	123.0	1.20	22.1	41.3	63.40	43.8

257.1	218.0	310.3	60.9	15.90	14.0	38.2	52.20	60.6
233.8	225.0	323.4	69.4	17.00	17.4	43.0	60.40	53.6
209.9	192.0	310.0	64.7	16.10	12.8	32.7	45.50	107.1
148.5	214.0	229.1	102.5	13.20	11.3	31.4	42.70	102.5
286.5	161.0	417.5	45.1	9.40	12.3	41.4	53.70	156.5
177.7	220.0	407.8	69.3	23.90	16.4	35.2	51.60	55.3
252.8	208.0	283.7	67.1	19.60	#NULL!	#NULL!	#NULL!	#NULL!
201.4	213.0	310.8	90.5	11.10	12.6	42.0	54.60	52.3
184.1	204.0	358.0	68.0	17.10	14.0	24.7	38.70	167.8
279.5	#NULL!	344.1	#NULL!	#NULL!	23.2	29.9	53.10	52.3
245.0	175.0	326.0	100.0	16.90	12.0	43.1	55.10	47.3
225.4	#NULL!	205.0	#NULL!	#NULL!	11.5	38.2	49.70	129.4
79.7	154.0	334.1	49.0	10.10	26.7	32.9	59.60	62.5
171.2	162.0	408.6	93.4	11.60	27.1	25.3	52.40	201.3
275.0	206.0	358.0	80.0	16.60	20.5	31.5	52.00	141.5
279.8	182.0	435.8	55.1	14.80	11.6	34.5	46.10	55.5
307.0	229.0	261.0	97.9	17.90	11.1	49.1	60.20	22.8
241.3	196.0	314.5	59.4	13.60	11.9	38.7	50.60	88.6
134.8	185.0	214.3	19.6	15.00	13.7	26.2	39.90	84.3
115.7	296.0	297.4	61.0	21.20	#NULL!	#NULL!	#NULL!	#NULL!
245.4	212.0	406.5	108.5	20.60	18.1	37.9	56.00	81.6
120.0	201.0	386.9	28.0	18.80	7.9	40.0	47.90	106.3
238.6	201.0	287.3	97.5	17.60	9.2	45.4	54.60	83.7
141.9	165.0	300.0	47.4	8.70	15.8	46.4	62.20	32.1
256.3	192.0	445.4	134.8	14.60	12.5	30.9	43.40	51.4
163.0	195.0	296.9	125.9	11.80	26.8	19.6	46.40	68.8
169.7	272.0	215.7	103.4	28.40	10.3	47.4	57.70	62.5
206.4	203.0	408.9	118.1	17.80	16.6	35.7	52.30	65.3
258.2	203.0	343.5	93.0	16.30	13.5	34.9	48.40	56.2
127.2	170.0	411.2	65.7	12.30	#NULL!	#NULL!	#NULL!	#NULL!
198.8	275.0	277.4	108.5	24.80	11.5	43.9	55.40	35.2
64.3	185.0	133.7	15.0	14.70	4.2	52.3	56.50	42.6
89.5	175.0	207.4	29.9	0.80	12.1	36.6	48.70	146.6
199.5	327.0	307.2	120.4	27.20	19.6	35.8	55.40	135.4
167.6	219.0	444.8	79.8	13.60	19.2	28.4	47.60	202.0
304.4	286.0	252.0	100.0	23.00	18.6	25.0	43.60	83.6
249.2	210.0	297.3	78.0	10.90	16.3	41.2	57.50	165.7
233.1	331.0	248.7	147.7	334.30	7.8	57.7	65.50	42.6
207.5	238.0	292.4	72.7	14.70	17.8	34.2	52.00	57.1
212.8	128.0	180.7	51.1	24.20	16.0	34.5	50.50	48.2
235.6	189.0	339.5	69.8	16.80	10.0	43.1	53.10	121.7
190.7	215.0	294.9	78.8	19.50	19.1	27.5	46.60	90.4
189.5	229.0	255.5	58.0	18.00	17.1	28.2	45.30	78.2
277.3	210.0	280.6	69.7	20.50	8.9	42.0	50.90	91.8
256.9	228.0	198.5	111.7	19.30	11.3	43.6	54.90	36.6
151.6	235.0	266.5	106.7	23.20	15.3	34.0	49.30	78.9
296.2	247.0	191.7	72.4	18.50	12.8	37.6	50.40	140.9
181.1	189.0	408.9	74.3	17.10	17.2	29.5	46.70	98.7
234.1	196.0	426.4	76.3	15.80	#NULL!	#NULL!	#NULL!	#NULL!
360.3	208.0	292.5	103.3	22.60	12.0	43.7	55.70	54.3
246.2	226.0	289.9	100.2	21.10	9.9	41.6	51.50	84.7
109.5	195.0	180.5	122.0	0.10	#NULL!	#NULL!	#NULL!	#NULL!
216.1	296.0	269.3	83.0	14.20	17.6	45.1	62.60	63.3
114.3	153.0	284.9	41.8	10.80	#NULL!	#NULL!	#NULL!	#NULL!

131.9	197.0	336.9	61.0	8.40	#NULL!	#NULL!	#NULL!	#NULL!
225.6	252.0	268.4	60.6	24.40	13.8	31.5	45.30	65.8
171.4	202.0	416.4	104.0	17.20	12.0	34.3	46.30	128.6
163.1	213.0	301.8	47.6	17.30	#NULL!	#NULL!	#NULL!	#NULL!
389.2	148.0	500.1	133.3	12.70	13.6	31.6	45.20	64.9
256.4	209.0	236.5	82.4	12.40	13.3	48.3	61.60	71.1
239.9	242.0	453.0	88.3	14.50	17.2	33.7	50.90	133.2
229.0	233.0	399.3	95.2	17.60	10.1	46.9	57.00	111.0
188.0	164.0	354.4	119.6	11.60	22.7	24.5	47.20	79.8
191.2	180.0	310.7	93.3	13.20	16.3	44.3	60.60	161.3
235.1	204.0	376.2	89.8	18.20	13.5	42.1	55.60	77.0
199.7	226.0	334.1	58.4	14.70	10.2	40.6	50.80	107.9
122.9	165.0	358.3	60.1	7.40	4.0	52.0	56.00	17.9
292.1	209.0	421.6	107.9	14.80	10.0	42.9	52.90	50.3
259.4	200.0	280.8	120.8	17.70	19.9	30.2	50.10	131.3
288.1	134.0	402.4	74.9	11.90	6.2	40.7	46.90	120.9
239.4	166.0	513.4	42.8	13.70	4.7	48.3	53.00	49.0
216.2	162.0	441.2	91.9	12.60	19.8	34.7	54.50	47.0
299.8	184.0	261.0	67.9	15.30	#NULL!	#NULL!	#NULL!	#NULL!
271.6	251.0	289.0	68.0	12.70	12.2	37.2	49.40	76.4
348.9	220.0	472.4	72.1	21.70	17.8	39.1	56.90	98.3
365.9	238.0	433.6	168.4	34.30	12.3	35.9	48.20	44.1
177.6	178.0	253.7	44.6	8.00	5.6	31.6	37.20	214.7
64.9	181.0	267.9	21.1	11.30	10.5	19.9	30.40	162.7
198.9	239.0	313.1	71.0	12.80	10.9	42.3	53.20	79.5
137.6	191.0	308.1	64.5	21.90	5.9	44.1	50.00	94.0
430.7	224.0	327.9	136.6	16.10	18.4	35.4	53.80	141.8
165.1	202.0	234.0	48.0	14.70	#NULL!	#NULL!	#NULL!	#NULL!
280.6	208.0	31.1	110.4	18.50	20.2	31.5	51.70	105.6
#NULL!	134.0	#NULL!	52.0	14.30	8.3	28.3	36.60	14.0
109.9	192.0	611.6	58.0	12.00	8.7	43.5	52.20	142.8
152.6	153.0	240.8	38.6	10.90	11.5	32.5	44.00	48.7
133.2	159.0	405.0	43.0	8.80	21.3	25.7	47.00	104.2
173.2	203.0	240.6	69.4	13.50	8.2	29.9	38.10	237.7
136.8	205.0	340.5	69.0	16.20	6.2	38.9	45.10	133.4
179.3	149.0	455.9	39.3	9.50	23.1	29.1	52.20	166.0
163.6	187.0	289.2	81.3	18.40	#NULL!	#NULL!	#NULL!	#NULL!
#NULL!	171.0	#NULL!	35.0	0.90	5.8	21.5	27.30	168.1
210.4	209.0	320.7	88.0	26.50	13.5	52.0	65.50	74.3
381.0	209.0	430.0	61.0	17.90	5.8	50.9	56.70	38.3
243.1	201.0	331.9	40.2	18.40	17.5	28.8	46.30	150.9
96.2	161.0	231.6	80.0	13.80	19.0	43.1	62.10	52.7
258.5	209.0	272.9	69.6	11.90	11.3	39.5	50.80	27.9
272.8	158.0	347.5	54.4	12.30	12.1	29.6	41.70	68.1
264.0	200.0	348.5	99.0	17.60	12.8	45.4	58.20	27.7
284.3	233.0	384.4	94.6	15.10	16.8	34.7	51.50	142.6
201.3	142.0	299.2	48.0	9.00	8.6	38.7	47.30	36.9
247.2	176.0	296.6	92.0	9.20	12.6	34.6	47.20	72.9
180.2	198.0	593.7	63.1	12.30	13.0	49.0	62.00	150.9
232.5	186.0	373.1	100.1	17.20	20.8	33.8	54.60	48.4
257.1	159.0	297.0	51.0	19.00	6.9	22.7	29.60	182.3
263.6	163.0	452.4	117.4	25.80	14.4	41.3	55.70	77.2
242.4	213.0	281.5	85.8	19.50	11.6	37.5	49.10	117.2
150.7	169.0	353.8	71.7	17.60	28.7	14.4	43.10	46.8

321.0	197.0	329.2	144.7	19.50	18.8	23.7	42.50	118.5
305.4	199.0	425.1	137.0	17.20	11.0	35.7	46.70	50.6
129.0	190.0	514.6	47.6	15.20	20.4	47.9	68.30	45.6
#NULL!	200.0	263.6	114.3	18.00	10.3	31.6	41.90	127.6
233.1	198.0	277.2	67.0	15.40	7.9	46.7	54.60	53.3
222.4	188.0	362.5	62.3	15.40	16.0	36.0	52.00	263.1
133.5	160.0	284.1	61.8	15.80	15.8	26.7	42.60	97.8
191.5	189.0	248.0	52.8	19.70	24.6	28.4	53.00	76.3
226.8	199.0	505.7	78.1	26.20	36.2	29.0	65.20	144.0
243.4	269.0	352.4	76.3	16.30	18.2	36.9	55.10	299.8
173.7	244.0	378.4	54.4	13.40	12.9	38.8	51.70	32.3
204.8	257.0	258.3	100.0	22.40	19.7	32.7	52.40	150.9
203.7	249.0	300.3	74.3	20.50	16.7	39.6	56.30	168.4
168.6	223.0	260.3	66.3	14.30	3.1	48.5	51.60	276.5
237.2	174.0	436.6	49.1	15.00	22.5	31.5	54.00	157.7
186.8	133.0	385.1	75.8	13.10	11.8	48.1	59.90	52.5
159.6	240.0	311.1	63.6	21.30	21.8	22.2	44.00	86.5
247.6	208.0	348.4	119.3	16.50	15.6	34.6	50.20	118.2
94.0	150.0	272.3	134.0	10.80	10.4	31.6	42.00	217.6
191.4	#NULL!	279.0	#NULL!	#NULL!	23.4	27.1	50.50	64.2
301.3	202.0	244.1	81.5	12.20	20.9	27.1	48.00	70.5
131.2	201.0	263.0	77.0	20.50	6.5	30.5	37.00	249.3
221.1	297.0	279.5	137.2	22.50	10.5	52.4	62.90	40.8
140.0	310.0	340.9	#NULL!	#NULL!	#NULL!	#NULL!	#NULL!	#NULL!
98.9	#NULL!	447.8	81.3	2.10	9.1	22.0	31.10	218.2
70.5	259.0	200.1	50.1	16.30	25.2	8.1	33.30	161.7
238.3	207.0	218.6	86.5	17.30	24.7	26.0	50.70	130.2
155.3	189.0	245.8	94.1	12.90	6.9	41.4	48.00	45.8
288.3	219.0	290.3	91.3	18.80	18.6	37.0	55.60	59.8
135.7	160.0	417.5	74.3	11.70	17.1	35.0	52.10	69.6
176.6	167.0	487.4	52.2	14.30	14.1	42.8	56.90	58.7
96.1	#NULL!	273.3	580.5	15.00	#NULL!	#NULL!	#NULL!	#NULL!
242.7	248.0	294.1	50.2	16.60	11.4	35.5	46.90	43.0
203.8	244.0	223.1	65.5	25.50	12.5	41.4	53.90	87.7
185.4	193.0	415.9	27.7	16.60	11.1	36.5	47.60	139.6
252.3	185.0	467.7	66.6	20.10	22.8	31.8	54.60	73.6
#NULL!	#NULL!	#NULL!	#NULL!	#NULL!	#NULL!	#NULL!	#NULL!	#NULL!
#NULL!	#NULL!	#NULL!	56.0	#NULL!	#NULL!	#NULL!	#NULL!	#NULL!
212.8	302.0	218.3	132.7	28.40	19.9	24.2	44.10	83.5
186.6	196.0	353.6	53.0	30.50	18.1	29.2	47.30	185.0
232.6	165.0	221.5	112.3	25.40	18.5	23.7	42.20	73.4
356.6	226.0	494.6	165.0	30.20	31.4	22.5	53.90	53.3
382.4	206.0	311.6	85.4	17.60	12.1	41.7	53.80	50.8
323.6	252.0	320.3	103.9	16.30	12.4	55.4	67.80	32.9
363.0	246.0	284.0	111.0	23.00	19.1	42.5	61.60	102.2
#NULL!	225.0	#NULL!	41.8	#NULL!	#NULL!	#NULL!	#NULL!	#NULL!
131.8	133.0	232.4	43.3	10.00	17.8	25.6	43.40	57.2
54.9	194.0	254.8	63.4	11.20	11.0	21.8	32.80	108.6
262.7	218.0	301.4	77.0	12.30	10.0	44.1	54.10	19.7
212.5	202.0	361.6	75.9	14.30	19.5	30.1	49.60	213.9
220.5	182.0	328.8	88.2	19.30	15.7	36.5	52.20	220.6
351.1	174.0	207.6	67.3	12.80	12.6	34.5	47.10	54.1
182.7	209.0	299.1	83.9	12.10	10.9	34.4	45.30	199.4
#NULL!	199.0	#NULL!	50.7	#NULL!	#NULL!	#NULL!	#NULL!	#NULL!

231.2	221.0	363.0	63.0	14.00	11.7	37.1	48.80	142.2
221.8	229.0	347.2	70.6	15.00	20.5	33.7	54.20	87.2
110.6	144.0	266.0	37.6	9.80	3.1	39.9	43.00	157.4
159.8	216.0	332.4	55.4	18.70	19.0	24.2	43.20	61.0
207.0	178.0	183.0	82.3	16.50	14.0	35.6	49.60	82.6
261.4	224.0	252.5	91.7	20.10	9.6	38.0	47.60	53.9
238.7	170.0	184.5	33.7	13.80	12.5	46.3	58.80	23.2
209.4	213.0	300.3	94.4	33.00	8.2	61.5	69.70	24.9
274.8	195.0	548.5	77.5	29.50	#NULL!	#NULL!	#NULL!	#NULL!
197.3	188.0	265.3	63.8	3.20	10.9	41.4	52.30	126.0
231.6	162.0	287.9	70.8	12.20	19.7	33.3	53.00	172.3
206.1	203.0	288.8	70.8	19.70	13.4	36.8	50.20	104.4
219.6	167.0	475.4	128.4	14.90	11.7	42.3	54.00	112.6
194.2	186.0	576.8	228.4	19.20	#NULL!	#NULL!	#NULL!	#NULL!
183.9	193.0	274.3	117.7	13.70	19.1	24.2	43.30	111.4
248.3	161.0	272.5	87.6	13.40	11.5	43.4	54.90	47.2
166.9	205.0	334.2	78.7	15.90	16.5	32.7	49.20	157.2
143.0	173.0	301.2	61.2	13.00	#NULL!	#NULL!	#NULL!	#NULL!
190.0	167.0	275.1	83.4	12.30	18.1	31.5	49.60	81.2
310.7	189.0	615.8	147.4	14.70	20.0	33.8	53.80	125.2
86.7	142.0	453.2	37.0	13.30	10.5	55.2	65.70	32.9
269.0	246.0	324.1	116.0	13.30	5.6	43.2	48.80	41.4
#NULL!	#NULL!	#NULL!	109.0	2.00	#NULL!	#NULL!	#NULL!	#NULL!
240.9	225.0	311.3	96.4	15.70	11.1	35.1	46.20	39.8
397.8	209.0	344.5	78.1	18.50	22.6	35.5	58.10	53.5
243.5	392.0	256.2	231.3	59.20	12.3	57.4	69.70	48.2
83.1	169.0	148.0	17.8	9.00	5.1	17.8	22.90	69.6
79.0	168.0	245.8	47.9	16.20	18.0	31.3	49.30	56.2
217.8	217.0	313.1	177.7	19.70	17.7	34.8	52.50	75.4
91.6	200.0	428.8	36.9	17.80	8.6	38.2	46.80	88.8
265.0	227.0	347.6	80.1	15.70	12.8	58.1	70.90	43.8
105.0	230.0	307.0	36.0	18.90	9.3	31.5	40.80	267.9
#NULL!	208.0	#NULL!	37.7	0.70	#NULL!	#NULL!	#NULL!	#NULL!
231.1	207.0	254.9	89.6	23.50	19.7	34.5	54.20	56.4
250.2	182.0	467.1	71.0	13.30	13.0	37.9	50.90	50.8
172.1	197.0	245.2	79.2	19.50	11.5	33.9	45.40	115.1
#NULL!	300.0	#NULL!	40.1	1.30	#NULL!	#NULL!	#NULL!	#NULL!
129.1	132.0	255.1	41.1	19.30	13.2	40.2	53.40	140.7
214.9	187.0	311.5	67.4	13.30	22.5	16.6	39.10	95.4
131.5	395.0	401.0	30.2	12.70	17.1	36.0	53.10	33.6
229.5	179.0	311.1	73.6	11.60	12.0	34.1	46.10	25.1
194.4	197.0	180.7	93.3	24.70	17.9	22.4	40.30	114.1
249.6	212.0	268.7	116.3	12.20	13.9	28.2	42.10	147.6
#NULL!	150.0	#NULL!	68.6	1.30	#NULL!	#NULL!	#NULL!	#NULL!
#NULL!	#NULL!	#NULL!	111.3	2.60	3.9	50.3	54.20	14.7
253.4	171.0	388.3	63.4	12.70	16.0	27.7	43.70	49.2
112.2	158.0	446.9	#NULL!	#NULL!	#NULL!	#NULL!	#NULL!	#NULL!
190.5	209.0	285.6	66.6	18.20	18.4	39.6	58.00	44.4
288.1	200.0	340.9	78.9	11.40	17.7	30.8	48.50	95.4
257.6	243.0	294.7	86.3	19.00	17.5	30.4	47.90	89.1
415.6	179.0	389.9	86.4	18.10	20.4	43.7	64.10	26.8
141.7	159.0	364.3	66.2	9.00	12.3	36.6	48.90	14.4
176.8	179.0	341.3	81.2	19.10	6.8	39.8	46.60	90.0
111.6	151.0	174.6	47.7	31.50	12.1	38.9	51.00	12.4

200.9	312.0	253.5	69.0	21.80	22.9	26.9	49.80	175.6
151.7	209.0	607.6	44.0	15.80	6.3	34.4	40.70	111.4
196.5	170.0	252.9	44.6	12.30	27.4	30.0	57.40	48.6
219.5	222.0	264.1	125.2	16.80	14.4	31.0	45.40	112.6
313.1	193.0	307.0	109.0	26.70	16.3	31.2	47.50	186.9
163.3	227.0	188.5	64.7	22.80	7.2	26.8	34.00	124.7
104.8	157.0	291.9	77.9	12.50	#NULL!	#NULL!	#NULL!	#NULL!
377.8	192.0	252.2	71.6	11.30	16.7	34.5	51.20	59.3
230.5	187.0	210.9	54.5	21.60	16.0	33.9	49.90	49.2
146.8	186.0	568.7	48.0	19.70	#NULL!	#NULL!	#NULL!	#NULL!
260.5	236.0	391.8	84.1	17.30	#NULL!	#NULL!	#NULL!	#NULL!
274.1	213.0	212.7	101.5	14.40	21.9	22.7	44.60	172.9
269.5	188.0	265.9	58.8	11.50	14.5	38.2	52.70	52.5
194.5	267.0	251.1	82.3	18.90	15.5	34.5	50.00	62.7
49.1	162.0	455.2	23.0	11.40	10.3	30.0	40.30	87.7
168.8	234.0	202.1	70.2	16.00	12.7	33.6	46.30	89.1
200.3	148.0	290.1	56.3	10.30	9.6	37.4	47.00	30.7
243.2	383.0	419.0	70.6	41.00	11.0	46.5	57.50	120.0
512.2	305.0	486.0	66.3	20.30	14.1	31.6	45.70	111.9
95.1	115.0	407.0	35.9	5.70	7.3	48.4	55.70	25.1
97.0	197.0	483.6	67.0	8.80	15.0	41.0	56.00	182.5
238.0	218.0	439.6	59.4	17.20	23.2	36.3	59.50	162.3
263.9	193.0	337.1	87.7	9.80	8.7	36.2	44.90	72.3
218.6	200.0	292.6	58.0	10.80	11.3	45.6	56.90	49.6
467.2	234.0	359.4	72.0	5.60	19.5	25.3	44.80	104.7
274.8	186.0	269.3	56.4	11.40	19.1	28.7	47.80	82.1
293.4	180.0	534.4	89.6	6.40	10.5	34.1	44.60	111.4
301.6	238.0	133.9	14.7	#NULL!	#NULL!	#NULL!	#NULL!	#NULL!
156.7	303.0	588.0	154.3	4.00	#NULL!	#NULL!	#NULL!	#NULL!
255.9	149.0	308.6	39.1	12.70	17.4	27.9	45.30	142.6
181.6	148.0	390.1	53.1	40.80	8.6	40.0	48.60	80.1
194.5	166.0	529.1	55.9	9.30	15.6	54.1	69.70	25.3
191.9	211.0	218.0	74.0	15.90	10.0	44.3	54.30	23.4
315.7	219.0	468.1	44.8	12.40	9.4	35.9	45.30	125.2
239.8	171.0	293.0	74.0	19.10	10.1	39.0	49.10	46.8
91.0	#NULL!	#NULL!	#NULL!	#NULL!	#NULL!	#NULL!	#NULL!	#NULL!
304.6	164.0	395.0	128.3	16.00	20.2	30.6	50.80	132.7
221.8	210.0	532.4	74.4	17.40	19.8	38.4	58.20	68.5
#NULL!	183.0	#NULL!	85.0	0.80	#NULL!	#NULL!	#NULL!	#NULL!
439.7	206.0	337.6	112.9	20.80	17.4	29.1	46.50	81.7
219.9	250.0	301.7	116.6	22.70	8.2	34.8	43.00	61.2
176.7	234.0	387.8	38.4	9.90	5.0	34.2	39.20	163.5
291.1	229.0	348.3	101.4	14.70	14.2	37.8	52.00	70.3
244.6	180.0	373.8	90.0	12.40	7.9	37.6	45.50	72.1
234.1	228.0	134.8	64.5	17.90	#NULL!	#NULL!	#NULL!	#NULL!
83.0	175.0	396.2	35.0	9.50	21.4	28.4	49.80	92.8
353.5	246.0	410.3	90.0	19.90	13.5	46.0	59.50	117.7
106.6	171.0	439.7	59.0	7.00	16.3	40.7	57.00	102.4
104.3	189.0	404.9	71.3	7.10	#NULL!	#NULL!	#NULL!	#NULL!
182.2	306.0	251.1	138.7	29.70	#NULL!	#NULL!	#NULL!	#NULL!
241.3	218.0	306.7	108.5	21.40	11.0	34.3	45.30	90.2
111.6	189.0	393.5	58.5	13.60	36.8	19.0	55.80	83.5
296.7	232.0	211.0	169.9	12.90	8.3	49.8	58.10	55.4
259.1	212.0	323.8	55.8	13.80	20.1	23.8	43.90	85.1

316.7	172.0	407.9	50.7	12.90	8.0	43.0	51.00	104.2
118.9	260.0	257.2	320.9	10.80	6.6	34.6	41.20	92.4
196.0	159.0	322.0	43.0	22.00	8.7	51.1	59.80	41.2
141.9	194.0	248.9	41.6	13.10	11.9	28.1	40.00	168.7
184.9	147.0	447.3	35.5	9.50	30.1	25.3	55.40	102.0
202.5	192.0	260.3	112.5	16.00	9.4	32.4	41.80	110.4
155.3	203.0	286.6	64.2	10.60	18.4	29.7	48.10	52.3
210.2	252.0	226.5	81.8	27.20	14.0	31.3	45.30	206.3
246.6	226.0	288.9	66.9	17.40	18.9	34.1	53.00	43.8
184.4	259.0	267.2	81.5	15.00	10.6	33.0	43.60	198.2
297.3	205.0	288.4	78.2	15.90	9.2	34.3	43.50	212.2
296.7	231.0	542.2	83.4	16.60	10.3	54.2	64.50	146.2
175.0	178.0	428.5	53.0	10.20	8.0	43.4	51.40	52.5
69.0	196.0	267.6	52.6	13.40	6.3	41.6	47.90	23.1
150.5	157.0	460.0	65.6	10.90	16.9	28.9	45.80	129.1
149.6	138.0	334.6	43.3	10.30	11.4	40.4	51.80	93.1
48.4	163.0	225.3	38.9	7.10	8.8	24.1	32.90	125.5
190.9	166.0	421.0	76.0	21.60	15.4	30.1	45.50	50.3
71.7	120.0	457.8	43.9	6.90	18.4	31.7	50.10	128.9
79.3	#NULL!	285.8	#NULL!	#NULL!	22.4	37.1	59.50	195.3
246.1	183.0	298.3	62.2	17.20	17.2	30.5	47.70	64.1
273.9	154.0	475.0	172.5	1.50	#NULL!	#NULL!	#NULL!	#NULL!
284.9	264.0	278.6	104.2	21.50	16.4	35.0	51.40	112.8
105.8	186.0	349.6	77.1	8.50	30.4	28.3	58.70	209.2
164.5	221.0	388.8	90.9	14.00	16.7	31.9	48.60	89.8
134.6	257.0	240.2	87.2	1.50	#NULL!	#NULL!	#NULL!	#NULL!
269.7	144.0	299.6	76.3	14.50	20.2	29.3	49.50	90.1
199.2	194.0	389.1	79.2	64.90	15.5	40.5	56.00	40.4
214.0	278.0	262.7	96.7	32.50	15.6	38.2	53.80	26.8
168.1	205.0	257.2	61.1	19.30	#NULL!	#NULL!	#NULL!	#NULL!
198.4	218.0	406.9	87.4	17.00	8.8	49.5	58.30	145.6
237.4	184.0	274.9	58.4	12.70	13.5	34.2	47.70	115.1
152.6	157.0	257.1	67.8	13.40	9.6	40.4	50.00	28.7
259.0	196.0	248.0	77.0	13.90	18.1	24.1	42.20	97.1
263.5	207.0	276.7	89.0	13.80	15.4	47.9	63.30	70.5
218.0	215.0	179.0	41.0	18.60	#NULL!	#NULL!	#NULL!	#NULL!
130.8	184.0	320.1	53.2	19.90	22.9	27.1	50.00	111.5
179.3	221.0	315.0	101.0	17.70	17.1	30.2	47.30	160.8
167.2	207.0	216.9	53.0	13.50	23.0	21.3	44.30	94.6
188.9	301.0	202.0	72.6	34.40	27.2	41.8	69.00	38.4
191.9	184.0	233.0	115.2	13.30	13.0	31.5	44.50	114.1
161.1	161.0	339.9	96.5	12.30	7.9	50.9	58.80	76.0
212.2	295.0	290.0	54.0	36.30	16.9	25.6	42.50	144.8
244.5	162.0	366.9	27.6	9.90	#NULL!	#NULL!	#NULL!	#NULL!
231.2	161.0	314.2	58.6	14.60	16.6	33.9	50.50	88.4
189.3	151.0	434.3	42.9	16.20	26.5	43.4	69.90	156.5
90.8	165.0	365.9	78.7	8.90	10.8	38.4	49.20	87.5
178.5	196.0	456.8	89.6	13.30	32.6	18.8	51.40	111.8
223.1	172.0	326.7	64.8	21.90	21.3	34.1	55.40	44.8
367.1	177.0	381.0	71.0	11.40	20.9	30.1	51.00	106.3
112.8	135.0	395.1	38.3	6.40	10.3	45.9	56.20	104.8
136.8	145.0	343.6	65.1	11.80	#NULL!	#NULL!	#NULL!	#NULL!
188.6	163.0	348.0	43.5	11.40	26.5	22.5	49.00	115.6
170.3	219.0	389.8	79.1	10.80	10.2	51.8	62.00	35.3

145.5	207.0	370.2	91.2	44.60	13.1	37.2	50.30	27.4
187.1	200.0	328.2	130.8	42.90	14.0	38.1	52.10	49.9
275.0	186.0	224.3	67.8	12.10	19.7	30.9	50.60	61.1
144.9	142.0	325.1	36.5	12.20	5.8	50.8	56.60	31.1
236.4	218.0	296.9	82.2	17.00	15.1	35.2	50.30	68.9
280.0	244.0	369.3	75.4	14.90	10.5	47.0	57.50	304.0
250.8	195.0	298.0	133.3	15.30	20.4	30.5	50.90	175.6
299.5	215.0	380.9	69.3	22.50	6.7	47.8	54.50	85.9
302.5	222.0	409.7	138.9	14.00	#NULL!	#NULL!	#NULL!	#NULL!
192.0	247.0	374.5	68.2	24.20	13.7	29.7	43.40	91.8
150.0	223.0	229.0	79.3	13.80	9.8	38.0	47.80	61.3
279.7	201.0	326.0	100.2	14.40	25.3	27.5	52.80	69.8
290.9	244.0	405.0	96.0	20.00	12.2	41.2	53.40	76.2
545.9	260.0	307.8	86.6	11.60	#NULL!	#NULL!	#NULL!	#NULL!
238.7	161.0	570.1	89.0	14.40	14.7	31.5	46.20	95.1
219.2	177.0	375.1	57.7	11.90	19.5	29.4	48.90	66.8
210.1	253.0	301.1	104.9	23.90	#NULL!	#NULL!	#NULL!	#NULL!
181.6	215.0	199.6	80.6	15.40	7.5	41.6	49.10	63.0
342.4	207.0	388.9	92.9	18.40	10.1	54.9	65.00	50.1
201.6	182.0	320.9	33.3	13.80	14.0	33.9	47.90	58.8
123.2	165.0	269.7	95.6	11.90	22.6	33.6	56.20	64.6
213.2	218.0	270.6	82.9	16.20	17.3	29.1	46.40	66.0

转铁蛋白	尿常规异常	尿糖阳性	尿蛋白阳性	尿红细胞阳	尿白细胞阳	胆红素阳性	尿胆原阳性	亚硝酸盐阳
#NULL!	0	0	0	0	0	0	0	0
#NULL!	1	0	0	0	0	1	1	0
#NULL!	1	0	1	1	1	0	0	1
#NULL!	0	0	0	0	0	0	0	0
#NULL!	1	0	0	0	1	0	0	0
2.36	0	0	0	0	0	0	0	0
#NULL!	0	0	0	0	0	0	0	0
#NULL!	0	0	0	0	0	0	0	0
#NULL!	0	0	0	0	0	0	0	0
#NULL!	0	0	0	0	0	0	0	0
#NULL!	0	0	0	0	0	0	0	0
#NULL!	0	0	0	0	0	0	0	0
2.52	0	0	0	0	0	0	0	0
#NULL!	1	0	0	1	1	0	0	0
#NULL!	1	0	1	0	0	0	0	0
#NULL!	0	0	0	0	0	0	0	0
1.34	1	0	0	1	1	0	0	0
2.93	1	0	0	0	0	0	0	0
#NULL!	0	0	0	0	0	0	0	0
#NULL!	0	0	0	0	0	0	0	0
#NULL!	1	0	0	0	0	0	0	0
#NULL!	1	0	0	0	0	0	0	0
#NULL!	0	0	0	0	0	0	0	0
#NULL!	0	0	0	0	0	0	0	0
#NULL!	0	0	0	0	0	0	0	0
#NULL!	0	0	0	0	0	0	0	0
#NULL!	1	0	0	0	0	0	0	0
#NULL!	0	0	0	0	0	0	0	0
#NULL!	0	0	0	0	0	0	0	0
#NULL!	1	0	0	1	0	0	0	0
#NULL!	0	0	0	0	0	0	0	0
#NULL!	0	0	0	0	0	0	0	0
#NULL!	0	0	0	0	0	0	0	0
#NULL!	0	0	0	0	0	0	0	0
#NULL!	1	1	1	0	0	0	0	0
#NULL!	0	0	0	0	0	0	0	0
#NULL!	0	0	0	0	0	0	0	0
#NULL!	0	0	0	0	0	0	0	0
#NULL!	1	0	1	0	1	0	0	0
#NULL!	0	0	0	0	0	0	0	0
3.02	0	0	0	0	0	0	0	0
#NULL!	1	0	0	1	0	0	0	0
#NULL!	0	0	0	0	0	0	0	0
#NULL!	0	0	0	0	0	0	0	0
#NULL!	0	0	0	0	0	0	0	0
#NULL!	0	0	0	0	0	0	0	0
#NULL!	0	0	0	0	0	0	0	0
#NULL!	0	0	0	0	0	0	0	0
#NULL!	1	0	0	0	1	0	0	0
#NULL!	0	0	0	0	0	0	0	0
2.50	0	0	0	0	0	0	0	0
#NULL!	0	0	0	0	0	0	0	0
#NULL!	1	0	1	0	1	0	0	0

#NULL!	0	0	0	0	0	0	0	0
2.11	1	0	0	1	0	0	0	0
#NULL!	0	0	0	0	0	0	0	0
#NULL!	1	0	1	1	0	0	0	0
#NULL!	0	0	0	0	0	0	0	0
#NULL!	0	0	0	0	0	0	0	0
#NULL!	0	0	0	0	0	0	0	0
#NULL!	1	0	1	0	0	0	0	0
#NULL!	1	0	1	0	0	0	0	0
#NULL!	0	0	0	0	0	0	0	0
#NULL!	0	0	0	0	0	0	0	0
#NULL!	0	0	0	0	0	0	0	0
2.04	0	0	0	0	0	0	0	0
#NULL!	0	0	0	0	0	0	0	0
#NULL!	0	0	0	0	0	0	0	0
#NULL!	0	0	0	0	0	0	0	0
#NULL!	1	0	0	0	0	0	0	0
#NULL!	0	0	0	0	0	0	0	0
#NULL!	0	0	0	0	0	0	0	0
#NULL!	0	0	0	0	0	0	0	0
#NULL!	0	0	0	0	0	0	0	0
#NULL!	1	0	0	1	0	0	0	0
#NULL!	1	0	0	0	1	0	0	0
#NULL!	0	0	0	0	0	0	0	0
#NULL!	0	0	0	0	0	0	0	0
2.45	1	0	0	1	0	0	0	0
#NULL!	0	0	0	0	0	0	0	0
#NULL!	0	0	0	0	0	0	0	1
2.88	1	0	0	0	1	0	0	0
#NULL!	0	0	0	0	0	0	0	0
#NULL!	0	0	0	0	0	0	0	0
#NULL!	0	0	0	0	0	0	0	0
#NULL!	1	0	0	1	0	0	0	0
#NULL!	0	0	0	0	0	0	0	0
#NULL!	0	0	0	0	0	0	0	0
#NULL!	1	0	1	0	0	0	0	0
#NULL!	0	0	0	0	0	0	0	0
#NULL!	1	0	0	0	1	0	0	0
#NULL!	0	0	0	0	0	0	0	0
#NULL!	0	0	0	0	0	0	0	0
#NULL!	0	0	0	0	0	0	0	0
#NULL!	0	0	0	0	0	0	0	0
#NULL!	0	0	0	0	0	0	0	0
#NULL!	1	0	0	0	1	0	0	0
#NULL!	0	0	0	0	0	0	0	0
#NULL!	1	1	0	0	0	0	0	0
#NULL!	1	0	0	1	0	0	0	0
#NULL!	0	0	0	0	0	0	0	0
#NULL!	0	0	0	0	0	0	0	0
#NULL!	0	0	0	0	0	0	0	0
#NULL!	1	0	0	0	1	0	0	0
#NULL!	0	0	0	0	0	0	0	0
2.36	0	0	0	0	0	0	0	0
2.67	0	0	0	0	0	0	0	0

#NULL!	0	0	0	0	0	0	0	0
#NULL!	0	0	0	0	0	0	0	0
#NULL!	0	0	0	0	0	0	0	0
#NULL!	0	0	0	0	0	0	0	0
#NULL!	0	0	0	0	0	0	0	0
#NULL!	0	0	0	0	0	0	0	0
3.08	1	0	0	0	1	0	0	0
#NULL!	0	0	0	0	0	0	0	0
3.06	0	0	0	0	0	0	0	0
2.74	0	0	0	0	0	0	0	0
#NULL!	0	0	0	0	0	0	0	0
#NULL!	0	0	0	0	0	0	0	0
2.61	0	0	0	0	0	0	0	0
2.49	0	0	0	0	0	0	0	0
#NULL!	1	0	0	0	0	0	0	0
#NULL!	1	0	0	0	0	0	0	0
#NULL!	1	1	0	0	0	0	0	0
#NULL!	0	0	0	0	0	0	0	0
#NULL!	0	0	0	0	0	0	0	0
#NULL!	0	0	0	0	0	0	0	0
#NULL!	0	0	0	0	0	0	0	0
#NULL!	0	0	0	0	0	0	0	0
#NULL!	0	0	0	0	0	0	0	0
#NULL!	0	0	0	0	0	0	0	0
#NULL!	0	0	0	0	0	0	0	0
#NULL!	0	0	0	0	0	0	0	0
#NULL!	0	0	0	0	0	0	0	0
1.96	0	0	0	0	0	0	0	0
#NULL!	0	0	0	0	0	0	0	0
#NULL!	0	0	0	0	0	0	0	0
1.97	0	0	0	0	0	0	0	0
#NULL!	0	0	0	0	0	0	0	0
#NULL!	1	0	0	1	0	0	0	0
#NULL!	1	0	0	1	0	0	0	0
#NULL!	0	0	0	0	0	0	0	0
#NULL!	1	0	1	0	0	0	0	0
#NULL!	1	0	0	0	0	0	0	0
#NULL!	1	0	0	0	1	0	0	0
#NULL!	0	0	0	0	0	0	0	0
#NULL!	0	0	0	0	0	0	0	0
1.91	0	0	0	0	0	0	0	0
#NULL!	0	0	0	0	0	0	0	0
#NULL!	1	0	0	0	1	0	0	0
#NULL!	0	0	0	0	0	0	0	0
#NULL!	0	0	0	0	0	0	0	0
2.19	0	0	0	0	0	0	0	0
#NULL!	0	0	0	0	0	0	0	0
#NULL!	1	0	0	1	0	1	1	0
#NULL!	0	0	0	0	0	0	0	0
#NULL!	0	0	0	0	0	0	0	0
#NULL!	0	0	0	0	0	0	0	0
#NULL!	0	0	0	0	0	0	0	0
#NULL!	1	0	0	0	0	0	0	0
#NULL!	0	0	0	0	0	0	0	0

#NULL!	0	0	0	0	0	0	0	0
#NULL!	1	0	0	0	1	0	0	0
3.41	1	0	0	0	0	0	0	0
#NULL!	0	0	0	0	0	0	0	0
#NULL!	1	0	0	0	0	0	0	0
#NULL!	1	0	0	0	0	0	0	0
#NULL!	0	0	0	0	0	0	0	0
#NULL!	0	0	0	0	0	0	0	0
0.26	1	0	1	0	0	0	0	0
#NULL!	0	0	0	0	0	0	0	0
#NULL!	0	0	0	0	0	0	0	0
#NULL!	0	0	0	0	0	0	0	0
#NULL!	1	0	0	0	0	0	0	0
#NULL!	0	0	0	0	0	0	0	0
#NULL!	1	0	0	1	0	0	0	0
#NULL!	0	0	0	0	0	0	0	0
#NULL!	0	0	0	0	0	0	0	0
#NULL!	0	0	0	0	0	0	0	0
#NULL!	1	0	0	1	0	0	0	0
#NULL!	0	0	0	0	0	0	0	0
#NULL!	0	0	0	0	0	0	0	0
#NULL!	1	0	0	0	0	0	0	0
#NULL!	1	0	0	0	1	0	1	0
2.69	0	0	0	0	0	0	0	0
#NULL!	0	0	0	0	0	0	0	0
#NULL!	1	0	1	1	0	0	0	0
#NULL!	0	0	0	0	0	0	0	0
1.94	0	0	0	0	0	0	0	0
1.91	0	0	0	0	0	0	0	0
2.75	0	0	0	0	0	0	0	0
#NULL!	0	0	0	0	0	0	0	0
#NULL!	1	0	1	0	0	0	0	0
#NULL!	1	0	0	0	0	0	1	0
#NULL!	1	0	1	0	0	0	0	1
#NULL!	1	0	0	0	0	0	0	0
#NULL!	1	0	1	0	1	1	1	0
#NULL!	1	0	0	0	0	0	0	0
#NULL!	0	0	0	0	0	0	0	0
1.43	0	0	0	0	0	0	0	0
#NULL!	0	0	0	0	0	0	0	0
#NULL!	0	0	0	0	0	0	0	0
#NULL!	0	0	0	0	0	0	0	0
#NULL!	0	0	0	0	0	0	0	0
#NULL!	0	0	0	0	0	0	0	0
1.85	0	0	0	0	0	0	0	0
#NULL!	0	0	0	0	0	0	0	0
2.86	0	0	0	0	0	0	0	0
#NULL!	0	0	0	0	0	0	0	0
#NULL!	1	0	0	1	0	0	0	0
#NULL!	0	0	0	0	0	0	0	0
1.81	0	0	0	0	0	0	0	0
#NULL!	0	0	0	0	0	0	0	0
#NULL!	0	0	0	0	0	0	0	0
#NULL!	0	0	0	0	0	0	0	0

#NULL!	0	0	0	0	0	0	0	0
#NULL!	0	0	0	0	0	0	0	0
#NULL!	0	0	0	0	0	0	0	0
#NULL!	0	0	0	0	0	0	0	0
#NULL!	0	0	0	0	0	0	0	0
#NULL!	0	0	0	0	0	0	0	0
3.70	0	0	0	0	0	0	0	0
#NULL!	0	0	0	0	0	0	0	0
2.87	0	0	0	0	0	0	0	0
#NULL!	0	0	0	0	0	0	0	0
#NULL!	0	0	0	0	0	0	0	0
#NULL!	0	0	0	0	0	0	0	0
#NULL!	0	0	0	0	0	0	0	0
#NULL!	0	0	0	0	0	0	0	0
#NULL!	0	0	0	0	0	0	0	0
#NULL!	0	0	0	0	0	0	0	0
2.52	0	0	0	0	0	0	0	0
#NULL!	0	0	0	0	0	0	0	0
2.93	0	0	0	0	0	0	0	0
3.37	0	0	0	0	0	0	0	0
#NULL!	0	0	0	0	0	0	0	0
#NULL!	0	0	0	0	0	0	0	0
#NULL!	0	0	0	0	0	0	0	0
#NULL!	0	0	0	0	0	0	0	0
#NULL!	0	0	0	0	0	0	0	0
#NULL!	0	0	0	0	0	0	0	0
0.35	0	0	0	0	0	0	0	0
#NULL!	0	0	0	0	0	0	0	0
#NULL!	0	0	0	0	0	0	0	0
#NULL!	0	0	0	0	0	0	0	0
1.43	0	0	0	0	0	0	0	0
2.61	0	0	0	0	0	0	0	0
#NULL!	0	0	0	0	0	0	0	0
#NULL!	0	0	0	0	0	0	0	0
#NULL!	0	0	0	0	0	0	0	0
#NULL!	0	0	0	0	0	0	0	0
#NULL!	0	0	0	0	0	0	0	0
#NULL!	0	0	0	0	0	0	0	0
#NULL!	0	0	0	0	0	0	0	0
2.31	0	0	0	0	0	0	0	0
#NULL!	0	0	0	0	0	0	0	0
#NULL!	0	0	0	0	0	0	0	0
#NULL!	0	0	0	0	0	0	0	0
#NULL!	0	0	0	0	0	0	0	0
#NULL!	0	0	0	0	0	0	0	0
2.00	0	0	0	0	0	0	0	0
#NULL!	0	0	0	0	0	0	0	0
2.31	0	0	0	0	0	0	0	0
#NULL!	0	0	0	0	0	0	0	0
#NULL!	0	0	0	0	0	0	0	0
#NULL!	0	0	0	0	0	0	0	0
#NULL!	0	0	0	0	0	0	0	0
#NULL!	0	0	0	0	0	0	0	0
#NULL!	0	0	0	0	0	0	0	0

3.40	0	0	0	0	0	0	0	0
#NULL!	0	0	0	0	0	0	0	0
#NULL!	0	0	0	0	0	0	0	0
1.17	0	0	0	0	0	0	0	0
#NULL!	0	0	0	0	0	0	0	0
#NULL!	0	0	0	0	0	0	0	0
#NULL!	0	0	0	0	0	0	0	0
#NULL!	0	0	0	0	0	0	0	0
#NULL!	0	0	0	0	0	0	0	0
#NULL!	0	0	0	0	0	0	0	0
#NULL!	0	0	0	0	0	0	0	0
#NULL!	0	0	0	0	0	0	0	0
#NULL!	0	0	0	0	0	0	0	0
#NULL!	0	0	0	0	0	0	0	0
#NULL!	0	0	0	0	0	0	0	0
#NULL!	0	0	0	0	0	0	0	0
#NULL!	0	0	0	0	0	0	0	0
#NULL!	0	0	0	0	0	0	0	0
#NULL!	0	0	0	0	0	0	0	0
2.20	0	0	0	0	0	0	0	0
#NULL!	0	0	0	0	0	0	0	0
#NULL!	0	0	0	0	0	0	0	0
#NULL!	0	0	0	0	0	0	0	0
#NULL!	0	0	0	0	0	0	0	0
#NULL!	0	0	0	0	0	0	0	0
#NULL!	0	0	0	0	0	0	0	0
#NULL!	0	0	0	0	0	0	0	0
#NULL!	0	0	0	0	0	0	0	0
#NULL!	0	0	0	0	0	0	0	0
#NULL!	0	0	0	0	0	0	0	0
#NULL!	0	0	0	0	0	0	0	0
#NULL!	0	0	0	0	0	0	0	0
#NULL!	0	0	0	0	0	0	0	0
#NULL!	0	0	0	0	0	0	0	0
1.81	0	0	0	0	0	0	0	0
#NULL!	0	0	0	0	0	0	0	0
#NULL!	0	0	0	0	0	0	0	0
#NULL!	0	0	0	0	0	0	0	0
2.10	0	0	0	0	0	0	0	0
#NULL!	0	0	0	0	0	0	0	0
1.65	0	0	0	0	0	0	0	0
2.24	0	0	0	0	0	0	0	0
1.89	0	0	0	0	0	0	0	0
#NULL!	0	0	0	0	0	0	0	0
#NULL!	0	0	0	0	0	0	0	0
2.31	0	0	0	0	0	0	0	0
2.47	0	0	0	0	0	0	0	0
#NULL!	0	0	0	0	0	0	0	0
2.15	0	0	0	0	0	0	0	0
#NULL!	0	0	0	0	0	0	0	0
2.20	0	0	0	0	0	0	0	0
#NULL!	0	0	0	0	0	0	0	0
#NULL!	0	0	0	0	0	0	0	0
2.39	0	0	0	0	0	0	0	0

1.43	0	0	0	0	0	0	0	0
3.28	0	0	0	0	0	0	0	0
2.34	0	0	0	0	0	0	0	0
2.34	0	0	0	0	0	0	0	0
#NULL!	0	0	0	0	0	0	0	0
2.14	0	0	0	0	0	0	0	0
2.11	0	0	0	0	0	0	0	0
2.31	0	0	0	0	0	0	0	0
2.03	0	0	0	0	0	0	0	0
4.41	0	0	0	0	0	0	0	0
1.59	0	0	0	0	0	0	0	0
2.39	0	0	0	0	0	0	0	0
2.70	0	0	0	0	0	0	0	0
2.77	0	0	0	0	0	0	0	0
2.67	0	0	0	0	0	0	0	0
2.57	0	0	0	0	0	0	0	0
#NULL!	0	0	0	0	0	0	0	0
2.14	0	0	0	0	0	0	0	0
2.53	0	0	0	0	0	0	0	0
#NULL!	0	0	0	0	0	0	0	0
1.93	0	0	0	0	0	0	0	0
#NULL!	0	0	0	0	0	0	0	0
2.22	0	0	0	0	0	0	0	0
#NULL!	0	0	0	0	0	0	0	0
#NULL!	0	0	0	0	0	0	0	0
#NULL!	0	0	0	0	0	0	0	0
#NULL!	0	0	0	0	0	0	0	0
2.60	0	0	0	0	0	0	0	0
#NULL!	0	0	0	0	0	0	0	0
#NULL!	0	0	0	0	0	0	0	0
2.25	0	0	0	0	0	0	0	0
1.82	0	0	0	0	0	0	0	0
2.14	0	0	0	0	0	0	0	0
#NULL!	0	0	0	0	0	0	0	0
#NULL!	0	0	0	0	0	0	0	0
1.23	0	0	0	0	0	0	0	0
#NULL!	0	0	0	0	0	0	0	0
2.04	0	0	0	0	0	0	0	0
#NULL!	0	0	0	0	0	0	0	0
2.27	0	0	0	0	0	0	0	0
2.76	0	0	0	0	0	0	0	0
#NULL!	0	0	0	0	0	0	0	0
#NULL!	0	0	0	0	0	0	0	0
#NULL!	0	0	0	0	0	0	0	0
3.34	0	0	0	0	0	0	0	0
#NULL!	0	0	0	0	0	0	0	0
2.75	0	0	0	0	0	0	0	0
2.02	0	0	0	0	0	0	0	0
2.59	0	0	0	0	0	0	0	0
2.25	0	0	0	0	0	0	0	0
1.50	0	0	0	0	0	0	0	0
2.02	0	0	0	0	0	0	0	0
2.45	0	0	0	0	0	0	0	0
2.61	0	0	0	0	0	0	0	0

#NULL!	0	0	0	0	0	0	0	0
1.92	0	0	0	0	0	0	0	0
1.61	0	0	0	0	0	0	0	0
#NULL!	0	0	0	0	0	0	0	0
#NULL!	0	0	0	0	0	0	0	0
2.59	0	0	0	0	0	0	0	0
#NULL!	0	0	0	0	0	0	0	0
#NULL!	0	0	0	0	0	0	0	0
1.09	0	0	0	0	0	0	0	0
2.20	0	0	0	0	0	0	0	0
2.27	0	0	0	0	0	0	0	0
2.94	0	0	0	0	0	0	0	0
2.26	0	0	0	0	0	0	0	0
2.20	0	0	0	0	0	0	0	0
#NULL!	0	0	0	0	0	0	0	0
#NULL!	0	0	0	0	0	0	0	0
1.09	0	0	0	0	0	0	0	0
#NULL!	0	0	0	0	0	0	0	0
#NULL!	0	0	0	0	0	0	0	0
2.56	0	0	0	0	0	0	0	0
#NULL!	0	0	0	0	0	0	0	0
#NULL!	0	0	0	0	0	0	0	0
#NULL!	0	0	0	0	0	0	0	0
2.40	0	0	0	0	0	0	0	0
#NULL!	0	0	0	0	0	0	0	0
2.34	0	0	0	0	0	0	0	0
#NULL!	0	0	0	0	0	0	0	0
2.13	0	0	0	0	0	0	0	0
2.40	0	0	0	0	0	0	0	0
2.17	0	0	0	0	0	0	0	0
1.15	0	0	0	0	0	0	0	0
#NULL!	0	0	0	0	0	0	0	0
#NULL!	0	0	0	0	0	0	0	0
2.05	0	0	0	0	0	0	0	0
2.08	0	0	0	0	0	0	0	0
2.04	0	0	0	0	0	0	0	0
#NULL!	0	0	0	0	0	0	0	0
#NULL!	0	0	0	0	0	0	0	0
2.53	0	0	0	0	0	0	0	0
1.09	0	0	0	0	0	0	0	0
1.93	0	0	0	0	0	0	0	0
#NULL!	0	0	0	0	0	0	0	0
2.54	0	0	0	0	0	0	0	0
2.34	0	0	0	0	0	0	0	0
#NULL!	0	0	0	0	0	0	0	0
2.73	0	0	0	0	0	0	0	0
#NULL!	0	0	0	0	0	0	0	0
#NULL!	0	0	0	0	0	0	0	0
2.30	0	0	0	0	0	0	0	0
#NULL!	0	0	0	0	0	0	0	0
#NULL!	0	0	0	0	0	0	0	0
#NULL!	0	0	0	0	0	0	0	0
2.67	0	0	0	0	0	0	0	0
#NULL!	0	0	0	0	0	0	0	0

#NULL!	0	0	0	0	0	0	0	0
#NULL!	0	0	0	0	0	0	0	0
#NULL!	0	0	0	0	0	0	0	0
#NULL!	0	0	0	0	0	0	0	0
#NULL!	0	0	0	0	0	0	0	0
#NULL!	0	0	0	0	0	0	0	0
#NULL!	0	0	0	0	0	0	0	0
3.17	0	0	0	0	0	0	0	0
#NULL!	0	0	0	0	0	0	0	0
#NULL!	0	0	0	0	0	0	0	0
#NULL!	0	0	0	0	0	0	0	0
#NULL!	0	0	0	0	0	0	0	0
#NULL!	0	0	0	0	0	0	0	0
#NULL!	0	0	0	0	0	0	0	0
2.24	0	0	0	0	0	0	0	0
2.44	0	0	0	0	0	0	0	0
#NULL!	0	0	0	0	0	0	0	0
#NULL!	0	0	0	0	0	0	0	0
2.10	0	0	0	0	0	0	0	0
3.10	0	0	0	0	0	0	0	0
#NULL!	0	0	0	0	0	0	0	0
1.92	0	0	0	0	0	0	0	0
2.60	0	0	0	0	0	0	0	0
2.30	0	0	0	0	0	0	0	0
2.10	0	0	0	0	0	0	0	0
2.03	0	0	0	0	0	0	0	0
2.41	0	0	0	0	0	0	0	0
2.74	0	0	0	0	0	0	0	0
2.39	0	0	0	0	0	0	0	0
2.32	0	0	0	0	0	0	0	0
#NULL!	0	0	0	0	0	0	0	0
2.77	0	0	0	0	0	0	0	0
2.65	0	0	0	0	0	0	0	0
2.13	0	0	0	0	0	0	0	0
2.36	0	0	0	0	0	0	0	0
2.74	0	0	0	0	0	0	0	0
#NULL!	0	0	0	0	0	0	0	0
#NULL!	0	0	0	0	0	0	0	0
2.26	0	0	0	0	0	0	0	0
2.74	0	0	0	0	0	0	0	0
#NULL!	0	0	0	0	0	0	0	0
2.69	0	0	0	0	0	0	0	0
2.44	0	0	0	0	0	0	0	0
2.83	0	0	0	0	0	0	0	0
3.17	0	0	0	0	0	0	0	0
2.07	0	0	0	0	0	0	0	0
3.33	0	0	0	0	0	0	0	0
2.60	0	0	0	0	0	0	0	0
#NULL!	0	0	0	0	0	0	0	0
3.07	0	0	0	0	0	0	0	0
#NULL!	0	0	0	0	0	0	0	0
#NULL!	0	0	0	0	0	0	0	0
2.36	0	0	0	0	0	0	0	0
#NULL!	0	0	0	0	0	0	0	0

#NULL!	0	0	0	0	0	0	0	0
#NULL!	0	0	0	0	0	0	0	0
#NULL!	0	0	0	0	0	0	0	0
#NULL!	0	0	0	0	0	0	0	0
#NULL!	0	0	0	0	0	0	0	0
2.05	0	0	0	0	0	0	0	0
#NULL!	0	0	0	0	0	0	0	0
2.04	0	0	0	0	0	0	0	0
#NULL!	0	0	0	0	0	0	0	0
3.27	0	0	0	0	0	0	0	0
#NULL!	0	0	0	0	0	0	0	0
#NULL!	0	0	0	0	0	0	0	0
#NULL!	0	0	0	0	0	0	0	0
#NULL!	0	0	0	0	0	0	0	0
#NULL!	0	0	0	0	0	0	0	0
#NULL!	0	0	0	0	0	0	0	0
#NULL!	0	0	0	0	0	0	0	0
#NULL!	0	0	0	0	0	0	0	0
#NULL!	0	0	0	0	0	0	0	0
#NULL!	0	0	0	0	0	0	0	0
#NULL!	0	0	0	0	0	0	0	0
#NULL!	0	0	0	0	0	0	0	0
#NULL!	0	0	0	0	0	0	0	0
#NULL!	0	0	0	0	0	0	0	0
#NULL!	0	0	0	0	0	0	0	0
2.50	0	0	0	0	0	0	0	0
#NULL!	0	0	0	0	0	0	0	0
#NULL!	0	0	0	0	0	0	0	0
#NULL!	0	0	0	0	0	0	0	0
#NULL!	0	0	0	0	0	0	0	0
#NULL!	0	0	0	0	0	0	0	0
#NULL!	0	0	0	0	0	0	0	0
#NULL!	0	0	0	0	0	0	0	0
#NULL!	0	0	0	0	0	0	0	0
#NULL!	0	0	0	0	0	0	0	0
#NULL!	0	0	0	0	0	0	0	0
#NULL!	0	0	0	0	0	0	0	0
2.84	0	0	0	0	0	0	0	0
#NULL!	0	0	0	0	0	0	0	0
#NULL!	0	0	0	0	0	0	0	0
#NULL!	0	0	0	0	0	0	0	0
#NULL!	0	0	0	0	0	0	0	0
2.32	0	0	0	0	0	0	0	0
#NULL!	0	0	0	0	0	0	0	0
#NULL!	0	0	0	0	0	0	0	0
#NULL!	0	0	0	0	0	0	0	0
#NULL!	0	0	0	0	0	0	0	0
#NULL!	0	0	0	0	0	0	0	0
#NULL!	0	0	0	0	0	0	0	0
#NULL!	0	0	0	0	0	0	0	0
#NULL!	0	0	0	0	0	0	0	0
#NULL!	0	0	0	0	0	0	0	0
3.39	0	0	0	0	0	0	0	0
#NULL!	0	0	0	0	0	0	0	0

#NULL!	0	0	0	0	0	0	0	0
#NULL!	0	0	0	0	0	0	0	0
#NULL!	0	0	0	0	0	0	0	0
#NULL!	0	0	0	0	0	0	0	0
#NULL!	0	0	0	0	0	0	0	0
1.16	0	0	0	0	0	0	0	0
#NULL!	0	0	0	0	0	0	0	0
#NULL!	0	0	0	0	0	0	0	0
#NULL!	0	0	0	0	0	0	0	0
#NULL!	0	0	0	0	0	0	0	0
#NULL!	0	0	0	0	0	0	0	0
#NULL!	0	0	0	0	0	0	0	0
#NULL!	0	0	0	0	0	0	0	0
#NULL!	0	0	0	0	0	0	0	0
#NULL!	0	0	0	0	0	0	0	0
#NULL!	0	0	0	0	0	0	0	0
#NULL!	0	0	0	0	0	0	0	0
#NULL!	0	0	0	0	0	0	0	0
2.44	0	0	0	0	0	0	0	0
#NULL!	0	0	0	0	0	0	0	0
#NULL!	0	0	0	0	0	0	0	0
#NULL!	0	0	0	0	0	0	0	0
#NULL!	0	0	0	0	0	0	0	0
#NULL!	0	0	0	0	0	0	0	0
#NULL!	0	0	0	0	0	0	0	0
#NULL!	0	0	0	0	0	0	0	0
2.69	1	0	1	0	0	0	0	0
#NULL!	0	0	0	0	0	0	0	0
2.36	0	0	0	0	0	0	0	0
#NULL!	0	0	0	0	0	0	0	0
#NULL!	0	0	0	0	0	0	0	0
#NULL!	0	0	0	0	0	0	0	0
#NULL!	1	0	0	0	0	0	0	1
2.48	1	0	0	0	0	1	1	0
#NULL!	0	0	0	0	0	0	0	0
#NULL!	0	0	0	0	0	0	0	0
#NULL!	0	0	0	0	0	0	0	0
#NULL!	0	0	0	0	0	0	0	0
#NULL!	0	0	0	0	0	0	0	0
#NULL!	0	0	0	0	0	0	0	0
#NULL!	0	0	0	0	0	0	0	0
#NULL!	1	0	0	0	0	0	0	0
#NULL!	0	0	0	0	0	0	0	0
#NULL!	1	0	0	0	0	1	0	0
#NULL!	0	0	0	0	0	0	0	0
#NULL!	0	0	0	0	0	0	0	0
#NULL!	0	0	0	0	0	0	0	0
#NULL!	0	0	0	0	0	0	0	0
#NULL!	0	0	0	0	0	0	0	0
#NULL!	1	0	0	0	0	0	0	0
#NULL!	1	0	0	0	1	0	1	0
#NULL!	0	0	0	0	0	0	0	0
#NULL!	1	0	0	1	1	0	0	0
#NULL!	0	0	0	0	0	0	0	0

#NULL!	1	0	0	0	1	0	0	0
#NULL!	1	0	0	1	0	0	0	0
#NULL!	0	0	0	0	0	0	0	0
#NULL!	0	0	0	0	0	0	0	0
#NULL!	1	0	0	0	1	0	0	0
#NULL!	0	0	0	0	0	0	0	0
#NULL!	1	0	0	0	1	0	0	0
1.59	1	0	1	0	1	1	1	0
#NULL!	1	0	0	0	1	1	0	0
1.94	1	0	0	0	0	0	1	0
2.86	0	0	0	0	0	0	0	0
2.59	0	0	0	0	0	0	0	0
2.10	0	0	0	0	0	0	0	0
2.00	1	0	0	1	0	0	0	0
2.70	0	0	0	0	0	0	0	0
#NULL!	0	0	0	0	0	0	0	0
#NULL!	1	0	0	0	0	0	1	0
#NULL!	0	0	0	0	0	0	0	0
2.40	0	0	0	0	0	0	0	0
#NULL!	1	0	0	0	1	0	0	0
2.80	0	0	0	0	0	0	0	0
2.70	0	0	0	0	0	0	0	0
2.60	0	0	0	0	0	0	0	0
3.20	0	0	0	0	0	0	0	0
#NULL!	0	0	0	0	0	0	0	0
#NULL!	0	0	0	0	0	0	0	0
#NULL!	1	0	0	0	0	0	0	0
2.64	1	0	0	0	0	0	0	0
2.61	0	0	0	0	0	0	0	0
2.84	1	0	1	0	0	0	1	0
2.14	1	0	0	0	0	0	0	0
#NULL!	1	0	0	0	0	0	1	0
2.28	0	0	0	0	0	0	0	0
2.03	1	0	0	0	0	0	0	0
#NULL!	0	0	0	0	0	0	0	0
#NULL!	0	0	0	0	0	0	0	0
#NULL!	1	0	1	0	0	0	0	0
3.61	0	0	0	0	0	0	0	0
3.04	0	0	0	0	0	0	0	0
2.33	0	0	0	0	0	0	0	0
2.17	0	0	0	0	0	0	0	0
#NULL!	0	0	0	0	0	0	0	0
2.39	0	0	0	0	0	0	0	0
2.21	1	0	0	1	1	0	0	0
#NULL!	1	0	0	0	1	0	0	0
2.02	0	0	0	0	0	0	0	0
#NULL!	0	0	0	0	0	0	0	0
#NULL!	0	0	0	0	0	0	0	0
#NULL!	0	0	0	0	0	0	0	0
#NULL!	0	0	0	0	0	0	0	0
#NULL!	1	0	0	0	1	0	0	0
#NULL!	1	0	0	0	0	0	0	0
#NULL!	0	0	0	0	0	0	0	0
#NULL!	1	0	0	1	1	0	0	0

#NULL!	1	0	0	0	1	0	0	0
#NULL!	1	0	0	0	0	0	0	0
#NULL!	1	0	0	0	0	0	1	0
#NULL!	0	0	0	0	0	0	0	0
2.27	1	0	0	0	0	0	0	0
2.15	1	0	0	0	1	1	0	0
#NULL!	0	0	0	0	0	0	0	0
2.76	0	0	0	0	0	0	0	0
#NULL!	0	0	0	0	0	0	0	0
#NULL!	1	0	0	0	0	0	1	0
#NULL!	1	0	0	1	0	0	0	0
#NULL!	0	0	0	0	0	0	0	0
2.45	0	0	0	0	0	0	0	0
#NULL!	0	0	0	0	0	0	0	0
3.55	0	0	0	0	0	0	0	0
#NULL!	1	0	1	0	0	0	0	0
#NULL!	0	0	0	0	0	0	0	0
#NULL!	0	0	0	0	0	0	0	0
#NULL!	0	0	0	0	0	0	0	0
#NULL!	0	0	0	0	0	0	0	0
#NULL!	0	0	0	0	0	0	0	0
#NULL!	0	0	0	0	0	0	0	0
3.12	0	0	0	0	0	0	0	0
#NULL!	0	0	0	0	0	0	0	0
#NULL!	#NULL!	#NULL!	#NULL!	#NULL!	#NULL!	#NULL!	#NULL!	#NULL!
#NULL!	0	0	0	0	0	0	0	0
#NULL!	0	0	0	0	0	0	0	0
2.05	0	0	0	0	0	0	0	0
#NULL!	0	0	0	0	0	0	0	0
#NULL!	1	1	0	0	0	0	0	0
#NULL!	#NULL!	#NULL!	#NULL!	#NULL!	#NULL!	#NULL!	#NULL!	#NULL!
1.79	0	0	0	0	0	0	0	0
2.20	0	0	0	0	0	0	0	0
1.82	1	0	0	0	0	0	1	0
#NULL!	0	0	0	0	0	0	0	0
2.61	0	0	0	0	0	0	0	0
3.06	0	0	0	0	0	0	0	0
2.86	0	0	0	0	0	0	0	0
2.11	0	0	0	0	0	0	0	0
#NULL!	0	0	0	0	0	0	0	0
#NULL!	0	0	0	0	0	0	0	0
#NULL!	0	0	0	0	0	0	0	0
#NULL!	0	0	0	0	0	0	0	0
#NULL!	0	0	0	0	0	0	0	0
#NULL!	0	0	0	0	0	0	0	0
#NULL!	1	0	0	0	0	0	1	0
2.53	1	0	0	0	1	0	0	0
2.64	0	0	0	0	0	0	0	0
2.61	0	0	0	0	0	0	0	0
#NULL!	#NULL!	#NULL!	#NULL!	#NULL!	#NULL!	#NULL!	#NULL!	#NULL!
#NULL!	0	0	0	0	0	0	0	0
2.59	0	0	0	0	0	0	0	0
2.13	0	0	0	0	0	0	0	0
#NULL!	0	0	0	0	0	0	0	0

2.05	0	0	0	0	0	0	0	0
#NULL!	0	0	0	0	0	0	0	0
#NULL!	#NULL!	#NULL!	#NULL!	#NULL!	#NULL!	#NULL!	#NULL!	#NULL!
#NULL!	0	0	0	0	0	0	0	0
#NULL!	1	0	0	0	1	0	0	0
#NULL!	1	0	0	0	1	0	0	0
#NULL!	1	0	1	0	0	0	0	0
#NULL!	#NULL!	#NULL!	#NULL!	#NULL!	#NULL!	#NULL!	#NULL!	#NULL!
#NULL!	0	0	0	0	0	0	0	0
2.57	#NULL!	#NULL!	#NULL!	#NULL!	#NULL!	#NULL!	#NULL!	#NULL!
2.28	#NULL!	#NULL!	#NULL!	#NULL!	#NULL!	#NULL!	#NULL!	#NULL!
2.45	0	0	0	0	0	0	0	0
#NULL!	0	0	0	0	0	0	0	0
1.86	1	1	0	0	0	0	0	0
#NULL!	0	0	0	0	0	0	0	0
2.50	0	0	0	0	0	0	0	0
1.79	0	0	0	0	0	0	0	0
1.89	0	0	0	0	0	0	0	0
#NULL!	0	0	0	0	0	0	0	0
2.19	#NULL!	#NULL!	#NULL!	#NULL!	#NULL!	#NULL!	#NULL!	#NULL!
2.39	1	0	0	0	0	1	0	0
2.87	#NULL!	#NULL!	#NULL!	#NULL!	#NULL!	#NULL!	#NULL!	#NULL!
2.25	0	0	0	0	0	0	0	0
2.57	#NULL!	#NULL!	#NULL!	#NULL!	#NULL!	#NULL!	#NULL!	#NULL!
2.97	1	0	0	1	0	0	0	0
2.74	#NULL!	#NULL!	#NULL!	#NULL!	#NULL!	#NULL!	#NULL!	#NULL!
#NULL!	0	0	0	0	0	0	0	0
2.72	#NULL!	#NULL!	#NULL!	#NULL!	#NULL!	#NULL!	#NULL!	#NULL!
2.08	1	0	0	0	0	1	0	0
2.55	#NULL!	#NULL!	#NULL!	#NULL!	#NULL!	#NULL!	#NULL!	#NULL!
#NULL!	0	0	0	0	0	0	0	0
2.37	0	0	0	0	0	0	0	0
3.25	0	0	0	0	0	0	0	0
#NULL!	#NULL!	#NULL!	#NULL!	#NULL!	#NULL!	#NULL!	#NULL!	#NULL!
#NULL!	#NULL!	#NULL!	#NULL!	#NULL!	#NULL!	#NULL!	#NULL!	#NULL!
#NULL!	1	0	0	1	1	0	0	0
3.36	1	0	1	1	0	0	0	0
2.67	#NULL!	#NULL!	#NULL!	#NULL!	#NULL!	#NULL!	#NULL!	#NULL!
2.72	0	0	0	0	0	0	0	0
#NULL!	1	0	0	1	1	0	0	0
#NULL!	0	0	0	0	0	0	0	0
#NULL!	0	0	0	0	0	0	0	0
2.96	1	0	0	0	1	0	0	0
2.48	0	0	0	0	0	0	0	0
1.70	1	0	0	0	1	1	0	0
#NULL!	0	0	0	0	0	0	0	0
3.23	#NULL!	#NULL!	#NULL!	#NULL!	#NULL!	#NULL!	#NULL!	#NULL!
#NULL!	0	0	0	0	0	0	0	0
2.89	1	0	0	0	1	0	0	0
1.74	1	0	0	0	1	0	0	0
2.05	1	0	0	0	1	0	0	0
2.46	#NULL!	#NULL!	#NULL!	#NULL!	#NULL!	#NULL!	#NULL!	#NULL!
3.38	#NULL!	#NULL!	#NULL!	#NULL!	#NULL!	#NULL!	#NULL!	#NULL!
2.12	1	0	0	1	1	0	0	0

2.70	0	0	0	0	0	0	0	0
2.52	0	0	0	0	0	0	0	0
2.47	0	0	0	0	0	0	0	0
#NULL!	#NULL!	#NULL!	#NULL!	#NULL!	#NULL!	#NULL!	#NULL!	#NULL!
2.07	1	0	0	0	1	0	0	0
2.62	0	0	0	0	0	0	0	0
#NULL!	0	0	0	0	0	0	0	0
2.03	0	0	0	0	0	0	0	0
2.01	0	0	0	0	0	0	0	0
#NULL!	0	0	0	0	0	0	0	0
2.13	0	0	0	0	0	0	0	0
2.37	1	0	1	0	1	0	0	0
2.38	0	0	0	0	0	0	0	0
2.63	0	0	0	0	0	0	0	0
2.02	0	0	0	0	0	0	0	0
3.41	0	0	0	0	0	0	0	0
#NULL!	0	0	0	0	0	0	0	0
1.98	0	0	0	0	0	0	0	0
2.09	1	1	0	1	1	0	0	0
#NULL!	1	0	0	0	1	0	0	0
2.49	1	0	0	0	1	0	0	0
#NULL!	0	0	0	0	0	0	0	0
2.61	#NULL!	#NULL!	#NULL!	#NULL!	#NULL!	#NULL!	#NULL!	#NULL!
3.00	#NULL!	#NULL!	#NULL!	#NULL!	#NULL!	#NULL!	#NULL!	#NULL!
2.58	1	0	0	0	1	0	0	0
2.05	1	0	0	0	0	0	1	0
#NULL!	1	0	0	0	0	0	1	0
1.87	#NULL!	#NULL!	#NULL!	#NULL!	#NULL!	#NULL!	#NULL!	#NULL!
1.94	0	0	0	0	0	0	0	0
2.08	0	0	0	0	0	0	0	0
#NULL!	#NULL!	#NULL!	#NULL!	#NULL!	#NULL!	#NULL!	#NULL!	#NULL!
#NULL!	1	0	0	0	0	1	1	0
2.09	1	0	0	0	1	0	0	0
3.10	0	0	0	0	0	0	0	0
#NULL!	0	0	0	0	0	0	0	0
2.51	0	0	0	0	0	0	0	0
#NULL!	#NULL!	#NULL!	#NULL!	#NULL!	#NULL!	#NULL!	#NULL!	#NULL!
3.03	0	0	0	0	0	0	0	0
2.42	0	0	0	0	0	0	0	0
2.58	0	0	0	0	0	0	0	0
#NULL!	1	0	0	0	0	0	0	0
2.24	0	0	0	0	0	0	0	0
2.67	#NULL!	#NULL!	#NULL!	#NULL!	#NULL!	#NULL!	#NULL!	#NULL!
#NULL!	0	0	0	0	0	0	0	0
#NULL!	0	0	0	0	0	0	0	0
#NULL!	0	0	0	0	0	0	0	0
#NULL!	0	0	0	0	0	0	0	0
1.93	0	0	0	0	0	0	0	0
2.74	1	0	1	0	0	0	0	0
2.58	1	0	1	0	0	0	0	0
2.87	0	0	0	0	0	0	0	0
2.97	#NULL!	#NULL!	#NULL!	#NULL!	#NULL!	#NULL!	#NULL!	#NULL!
2.48	1	0	0	0	0	0	1	0
2.59	1	0	0	1	0	0	0	0

2.74	0	0	0	0	0	0	0	0
#NULL!	1	0	0	1	0	0	0	0
#NULL!	0	0	0	0	0	0	0	0
2.62	0	0	0	0	0	0	0	0
#NULL!	0	0	0	0	0	0	0	0
3.04	#NULL!	#NULL!	#NULL!	#NULL!	#NULL!	#NULL!	#NULL!	#NULL!
#NULL!	#NULL!	#NULL!	#NULL!	#NULL!	#NULL!	#NULL!	#NULL!	#NULL!
#NULL!	0	0	0	0	0	0	0	0
2.45	0	0	0	0	0	0	0	0
2.69	0	0	0	0	0	0	0	0
3.02	1	0	0	0	0	0	1	0
#NULL!	0	0	0	0	0	0	0	0
#NULL!	0	0	0	0	0	0	0	0
#NULL!	1	0	1	0	0	0	0	0
2.78	0	0	0	0	0	0	0	0
#NULL!	1	0	0	0	0	0	1	0
#NULL!	0	0	0	0	0	0	0	0
3.45	0	0	0	0	0	0	0	0
#NULL!	0	0	0	0	0	0	0	0
2.97	0	0	0	0	0	0	0	0
#NULL!	0	0	0	0	0	0	0	0
#NULL!	0	0	0	0	0	0	0	0
#NULL!	1	0	0	0	1	0	0	0
#NULL!	1	0	0	0	1	0	0	0
#NULL!	0	0	0	0	0	0	0	0
2.61	#NULL!	#NULL!	#NULL!	#NULL!	#NULL!	#NULL!	#NULL!	#NULL!
2.91	0	0	0	0	0	0	0	0
#NULL!	#NULL!	#NULL!	#NULL!	#NULL!	#NULL!	#NULL!	#NULL!	#NULL!
#NULL!	0	0	0	0	0	0	0	0
#NULL!	0	0	0	0	0	0	0	0
#NULL!	0	0	0	0	0	0	0	0
#NULL!	0	0	0	0	0	0	0	0
#NULL!	0	0	0	0	0	0	0	0
#NULL!	0	0	0	0	0	0	0	0
#NULL!	0	0	0	0	0	0	0	0
2.08	1	0	0	0	1	0	0	0
#NULL!	1	0	0	1	0	0	0	0
#NULL!	0	0	0	0	0	0	0	0
3.19	1	0	0	1	0	0	0	0
#NULL!	0	0	0	0	0	0	0	0
#NULL!	0	0	0	0	0	0	0	0
1.63	1	0	1	0	0	0	0	0
2.56	0	0	0	0	0	0	0	0
#NULL!	#NULL!	#NULL!	#NULL!	#NULL!	#NULL!	#NULL!	#NULL!	#NULL!
#NULL!	0	0	0	0	0	0	0	0
#NULL!	0	0	0	0	0	0	0	0
#NULL!	#NULL!	#NULL!	#NULL!	#NULL!	#NULL!	#NULL!	#NULL!	#NULL!
#NULL!	0	0	0	0	0	0	0	0
#NULL!	1	0	0	0	1	0	0	0
#NULL!	1	0	0	1	1	0	0	0
#NULL!	1	0	1	0	1	0	0	0
#NULL!	0	0	0	0	0	0	0	0
#NULL!	1	0	0	0	0	0	0	0
#NULL!	1	0	0	1	1	0	0	0

#NULL!	0	0	0	0	0	0	0	0
#NULL!	0	0	0	0	0	0	0	0
1.67	0	0	0	0	0	0	0	0
1.40	1	0	1	0	1	1	1	0
#NULL!	0	0	0	0	0	0	0	0
2.15	0	0	0	0	0	0	0	0
#NULL!	#NULL!	#NULL!	#NULL!	#NULL!	#NULL!	#NULL!	#NULL!	#NULL!
#NULL!	0	0	0	0	0	0	0	0
#NULL!	0	0	0	0	0	0	0	0
#NULL!	#NULL!	#NULL!	#NULL!	#NULL!	#NULL!	#NULL!	#NULL!	#NULL!
#NULL!	0	0	0	0	0	0	0	0
#NULL!	0	0	0	0	0	0	0	0
#NULL!	0	0	0	0	0	0	0	0
2.09	0	0	0	0	0	0	0	0
1.97	1	0	0	0	1	0	0	0
2.62	0	0	0	0	0	0	0	0
2.32	1	0	0	0	0	0	0	0
#NULL!	1	0	0	0	1	0	0	0
#NULL!	0	0	0	0	0	0	0	0
2.17	0	0	0	0	0	0	0	0
2.49	1	0	1	0	1	0	0	0
2.40	0	0	0	0	0	0	0	0
#NULL!	1	0	1	0	0	0	0	0
#NULL!	1	0	0	0	1	0	0	0
2.58	0	0	0	0	0	0	0	0
2.35	0	0	0	0	0	0	0	0
2.28	1	0	0	0	1	0	1	0
#NULL!	0	0	0	0	0	0	0	0
#NULL!	1	0	1	0	1	0	0	0
2.61	#NULL!	#NULL!	#NULL!	#NULL!	#NULL!	#NULL!	#NULL!	#NULL!
#NULL!	1	0	0	0	1	0	0	0
2.66	0	0	0	0	0	0	0	0
#NULL!	0	0	0	0	0	0	0	0
#NULL!	0	0	0	0	0	0	0	0
2.46	1	0	0	0	1	0	0	0
#NULL!	1	0	0	0	1	0	0	0
#NULL!	1	0	0	0	1	0	0	0
2.19	1	0	0	0	1	0	0	0
#NULL!	0	0	0	0	0	0	0	0
#NULL!	0	0	0	0	0	0	0	0
#NULL!	0	0	0	0	0	0	0	0
#NULL!	1	0	0	0	1	0	0	0
#NULL!	0	0	0	0	0	0	0	0
3.00	#NULL!	#NULL!	#NULL!	#NULL!	#NULL!	#NULL!	#NULL!	#NULL!
2.51	0	0	0	0	0	0	0	0
#NULL!	0	0	0	0	0	0	0	0
#NULL!	1	0	0	0	1	0	0	0
2.55	#NULL!	#NULL!	#NULL!	#NULL!	#NULL!	#NULL!	#NULL!	#NULL!
#NULL!	1	0	0	0	1	0	0	0
#NULL!	1	0	#NULL!	0	1	0	0	0
#NULL!	0	0	0	0	0	0	0	0
#NULL!	1	0	0	0	1	0	0	0
#NULL!	0	0	0	0	0	0	0	0
#NULL!	0	0	0	0	0	0	0	0

#NULL!	0	0	0	0	0	0	0	0
#NULL!	1	0	0	0	1	0	0	0
#NULL!	#NULL!	#NULL!	#NULL!	#NULL!	#NULL!	#NULL!	#NULL!	#NULL!
#NULL!	1	0	0	1	0	0	0	0
#NULL!	1	0	0	0	1	0	0	0
2.20	0	0	0	0	0	0	0	0
#NULL!	0	0	0	0	0	0	0	0
#NULL!	1	0	0	0	1	0	0	0
#NULL!	0	0	0	0	0	0	0	0
2.20	0	0	0	0	0	0	0	0
#NULL!	0	0	0	0	0	0	0	0
#NULL!	0	0	0	0	0	0	0	0
#NULL!	#NULL!	#NULL!	#NULL!	#NULL!	#NULL!	#NULL!	#NULL!	#NULL!
2.49	0	0	0	0	0	0	0	0
#NULL!	0	0	0	0	0	0	0	0
#NULL!	0	0	0	0	0	0	0	0
2.47	0	0	0	0	0	0	0	0
#NULL!	#NULL!	#NULL!	#NULL!	#NULL!	#NULL!	#NULL!	#NULL!	#NULL!
2.21	1	0	0	0	0	0	0	0
2.76	0	0	0	0	0	0	0	0
2.50	0	0	0	0	0	0	0	0
2.69	0	0	0	0	0	0	0	0
2.44	0	0	0	0	0	0	0	0
2.35	0	0	0	0	0	0	0	0
2.30	1	0	0	0	0	0	1	0
#NULL!	0	0	0	0	0	0	0	0
3.10	0	0	0	0	0	0	0	0
2.17	1	0	0	0	1	0	0	1
2.52	0	0	0	0	0	0	0	0
2.04	0	0	0	0	0	0	0	0
0.86	0	0	0	0	0	0	0	0
#NULL!	0	0	0	0	0	0	0	0
#NULL!	0	0	0	0	0	0	0	0
#NULL!	0	0	0	0	0	0	0	0
#NULL!	0	0	0	0	0	0	0	0
#NULL!	0	0	0	0	0	0	0	0
#NULL!	0	0	0	0	0	0	0	0
3.82	0	0	0	0	0	0	0	0
2.27	0	0	0	0	0	0	0	0
#NULL!	0	0	0	0	0	0	0	0
#NULL!	0	0	0	0	0	0	0	0
2.17	0	0	0	0	0	0	0	0
2.16	0	0	0	0	0	0	0	0
#NULL!	0	0	0	0	0	0	0	0
2.69	0	0	0	0	0	0	0	0
2.77	1	0	0	0	1	0	0	0
3.05	0	0	0	0	0	0	0	0
2.44	1	0	1	0	1	0	1	0
2.68	0	0	0	0	0	0	0	0
3.00	1	0	1	0	1	0	0	0
2.47	0	0	0	0	0	0	0	0
#NULL!	0	0	0	0	0	0	0	0
#NULL!	0	0	0	0	0	0	0	0
#NULL!	0	0	0	0	0	0	0	0

2.63	0	0	0	0	0	0	0	0
2.22	0	0	0	0	0	0	0	0
2.55	1	0	0	0	1	0	0	0
1.98	1	0	0	0	0	0	0	0
2.68	0	0	0	0	0	0	0	0
#NULL!	1	0	0	0	0	0	0	0
2.50	0	0	0	0	0	0	0	0
#NULL!	1	0	0	1	1	0	0	0
2.45	0	0	0	0	0	0	0	0
2.12	0	0	0	0	0	0	0	0
2.46	0	0	0	0	0	0	0	0
#NULL!	0	0	0	0	0	0	0	0
#NULL!	0	0	0	0	0	0	0	0
2.37	0	0	0	0	0	0	0	0
#NULL!	0	0	0	0	0	0	0	0
#NULL!	0	0	0	0	0	0	0	0
#NULL!	0	0	0	0	0	0	0	0
#NULL!	0	0	0	0	0	0	0	0
#NULL!	0	0	0	0	0	0	0	0
#NULL!	0	0	0	0	0	0	0	0
3.02	1	0	0	0	0	0	1	0
3.13	0	0	0	0	0	0	0	0
2.91	0	0	0	0	0	0	0	0
2.42	0	0	0	0	0	0	0	0
1.88	0	0	0	0	0	0	0	0
3.09	#NULL!	#NULL!	#NULL!	#NULL!	#NULL!	#NULL!	#NULL!	#NULL!
2.51	1	0	1	0	0	0	0	0
1.76	0	0	0	0	0	0	0	0
3.29	0	0	0	0	0	0	0	0
2.62	0	0	0	0	0	0	0	0
2.49	#NULL!	#NULL!	#NULL!	#NULL!	#NULL!	#NULL!	#NULL!	#NULL!
2.79	1	0	0	1	0	0	0	0
2.79	1	0	0	0	1	0	0	0
2.36	0	0	0	0	0	0	0	0
2.47	0	0	0	0	0	0	0	0
2.19	0	0	0	0	0	0	0	0
2.90	#NULL!	#NULL!	#NULL!	#NULL!	#NULL!	#NULL!	#NULL!	#NULL!
2.78	1	0	0	0	1	0	0	0
#NULL!	0	0	0	0	0	0	0	0
2.38	0	0	0	0	0	0	0	0
#NULL!	1	0	0	0	0	0	0	0
2.33	#NULL!	#NULL!	#NULL!	#NULL!	#NULL!	#NULL!	#NULL!	#NULL!
2.56	#NULL!	#NULL!	#NULL!	#NULL!	#NULL!	#NULL!	#NULL!	#NULL!
2.16	#NULL!	#NULL!	#NULL!	#NULL!	#NULL!	#NULL!	#NULL!	#NULL!
2.96	0	0	0	0	0	0	0	0
3.05	1	0	0	0	0	0	1	0
2.59	0	0	0	0	0	0	0	0
#NULL!	1	0	0	0	0	0	0	0
2.37	1	0	0	1	0	0	0	0
2.28	0	0	0	0	0	0	0	0
2.57	0	0	0	0	0	0	0	0
2.12	1	0	0	1	0	0	0	0
1.97	1	0	1	1	1	0	0	0
2.98	0	0	0	0	0	0	0	0

0.27	1	1	1	1	0	0	0	0
2.93	1	0	0	0	1	0	0	0
1.99	0	0	0	0	0	0	0	0
2.20	0	0	0	0	0	0	0	0
2.71	1	0	0	0	1	0	0	0
2.53	0	0	0	0	0	0	0	0
2.71	0	0	0	0	0	0	0	0
2.82	1	0	0	0	1	0	0	0
3.09	0	0	0	0	0	0	0	0
#NULL!	0	0	0	0	0	0	0	0
3.19	0	0	0	0	0	0	0	0
2.23	1	0	1	0	0	0	0	0
2.04	1	0	0	0	1	0	0	0
2.55	1	0	0	0	1	0	0	0
2.58	1	0	0	1	0	0	0	0
2.41	1	0	0	0	1	0	0	0
2.93	0	0	0	0	0	0	0	0
2.92	0	0	0	0	0	0	0	0
2.40	0	0	0	0	0	0	0	0
#NULL!	1	0	0	0	0	0	0	0
2.10	0	0	0	0	0	0	0	0
1.50	0	0	0	0	0	0	0	0
#NULL!	1	0	0	0	1	1	0	0
2.45	1	0	0	0	1	0	0	0
2.79	0	0	0	0	0	0	0	0
3.22	1	0	0	0	1	0	0	0
2.42	1	0	0	1	1	0	0	0
2.42	0	0	0	0	0	0	0	0
2.35	0	0	0	0	0	0	0	0
2.83	0	0	0	0	0	0	0	0
2.46	0	0	0	0	0	0	0	0
3.05	0	0	0	0	0	0	0	0
2.43	1	0	0	0	0	0	0	0
2.81	0	0	0	0	0	0	0	0
#NULL!	1	0	0	0	0	1	0	0
2.26	0	0	0	0	0	0	0	0
2.29	1	0	0	1	1	0	0	0
1.80	0	0	0	0	0	0	0	0
2.75	1	0	0	0	1	0	0	0
2.11	1	0	0	0	0	0	0	0
2.19	0	0	0	0	0	0	0	0
2.27	1	0	0	1	0	0	0	0
2.41	1	0	0	1	1	0	0	0
#NULL!	0	0	0	0	0	0	0	0
2.31	1	0	0	1	0	0	0	0
#NULL!	0	0	0	0	0	0	0	0
2.55	0	0	0	0	0	0	0	0
2.56	0	0	0	0	0	0	0	0
2.60	0	0	0	0	0	0	0	0
2.19	0	0	0	0	0	0	0	0
2.53	1	0	0	1	0	0	0	0
2.24	1	0	0	0	1	0	0	0
2.66	1	0	0	0	1	0	0	0
2.99	0	0	0	0	0	0	0	0

2.54	0	0	0	0	0	0	0	0
2.87	1	0	0	0	1	0	0	0
2.05	0	0	0	0	0	0	0	0
2.23	1	0	0	1	1	0	0	0
2.42	0	0	0	0	0	0	0	0
2.30	0	0	0	0	0	0	0	0
#NULL!	1	0	0	0	0	0	0	0
2.45	0	0	0	0	0	0	0	0
2.11	0	0	0	0	0	0	0	0
2.56	0	0	0	0	0	0	0	0
2.78	1	0	0	1	0	0	0	0
2.22	1	0	0	0	0	0	1	0
2.77	0	0	0	0	0	0	0	0
2.45	0	0	0	0	0	0	0	0
2.40	0	0	0	0	0	0	0	0
2.31	0	0	0	0	0	0	0	0
2.83	0	0	0	0	0	0	0	0
2.48	0	0	0	0	0	0	0	0
1.73	1	0	0	1	1	0	0	0
#NULL!	#NULL!	#NULL!	#NULL!	#NULL!	#NULL!	#NULL!	#NULL!	#NULL!
2.73	0	0	0	0	0	0	0	0
2.09	1	0	1	0	0	0	0	0
2.61	0	0	0	0	0	0	0	0
2.85	0	0	0	0	0	0	0	0
2.17	0	0	0	0	0	0	0	0
2.14	0	0	0	0	0	0	0	0
2.66	0	0	0	0	0	0	0	0
2.61	0	0	0	0	0	0	0	0
2.21	0	0	0	0	0	0	0	0
#NULL!	1	0	1	0	0	0	1	0
2.61	0	0	0	0	0	0	0	0
2.74	0	0	0	0	0	0	0	0
2.39	1	0	1	1	0	0	0	0
2.61	1	0	0	0	1	0	0	0
2.25	0	0	0	0	0	0	0	0
2.26	1	0	0	0	1	0	0	0
2.58	1	0	0	0	1	0	0	0
3.04	1	0	1	0	0	0	0	0
2.44	1	0	0	0	1	0	0	0
2.24	0	0	0	0	0	0	0	0
2.44	0	0	0	0	0	0	0	0
1.86	1	0	0	0	1	0	0	0
2.01	0	0	0	0	0	0	0	0
2.16	0	0	0	0	0	0	0	0
2.43	0	0	0	0	0	0	0	0
2.13	0	0	0	0	0	0	0	0
2.39	0	0	0	0	0	0	0	0
2.16	1	0	0	0	0	0	0	0
#NULL!	0	0	0	0	0	0	0	0
2.62	0	0	0	0	0	0	0	0
2.45	0	0	0	0	0	0	0	0
#NULL!	1	1	0	0	0	0	0	0
2.94	0	0	0	0	0	0	0	0
#NULL!	0	0	0	0	0	0	0	0

#NULL!	0	0	0	0	0	0	0	0
2.07	0	0	0	0	0	0	0	0
2.30	0	0	0	0	0	0	0	0
#NULL!	1	0	0	0	0	0	0	0
2.29	1	0	0	0	1	0	0	0
3.21	0	0	0	0	0	0	0	0
2.47	0	0	0	0	0	0	0	0
2.92	0	0	0	0	0	0	0	0
2.25	0	0	0	0	0	0	0	0
2.99	0	0	0	0	0	0	0	0
2.83	0	0	0	0	0	0	0	0
2.77	0	0	0	0	0	0	0	0
2.96	0	0	0	0	0	0	0	0
2.73	0	0	0	0	0	0	0	0
2.53	1	0	0	0	1	0	0	0
2.59	1	0	0	0	0	0	1	0
2.68	0	0	0	0	0	0	0	0
2.84	0	0	0	0	0	0	0	0
#NULL!	0	0	0	0	0	0	0	0
2.44	0	0	0	0	0	0	0	0
2.76	0	0	0	0	0	0	0	0
2.42	0	0	0	0	0	0	0	0
1.95	0	0	0	0	0	0	0	0
1.42	1	0	0	0	0	0	0	0
2.57	0	0	0	0	0	0	0	0
2.45	0	0	0	0	0	0	0	0
2.54	0	0	0	0	0	0	0	0
#NULL!	0	0	0	0	0	0	0	0
2.58	1	0	0	0	0	0	1	0
2.17	0	0	0	0	0	0	0	0
2.43	1	0	0	0	0	0	0	0
2.22	0	0	0	0	0	0	0	0
2.57	0	0	0	0	0	0	0	0
1.87	0	0	0	0	0	0	0	0
2.18	0	0	0	0	0	0	0	0
2.50	0	0	0	0	0	0	0	0
#NULL!	0	0	0	0	0	0	0	0
1.31	1	0	1	1	0	0	0	0
3.00	1	0	1	0	0	0	1	0
2.61	1	0	1	1	0	0	0	0
2.21	0	0	0	0	0	0	0	0
2.93	0	0	0	0	0	0	0	0
2.54	0	0	0	0	0	0	0	0
2.15	0	0	0	0	0	0	0	0
2.67	0	0	0	0	0	0	0	0
2.52	0	0	0	0	0	0	0	0
2.41	0	0	0	0	0	0	0	0
2.32	0	0	0	0	0	0	0	0
2.94	0	0	0	0	0	0	0	0
2.66	0	0	0	0	0	0	0	0
1.67	1	0	1	1	0	0	0	0
2.53	0	0	0	0	0	0	0	0
2.29	0	0	0	0	0	0	0	0
1.92	0	0	0	0	0	0	0	0

2.00	0	0	0	0	0	0	0	0
2.15	0	0	0	0	0	0	0	0
3.31	0	0	0	0	0	0	0	0
2.02	0	0	0	0	0	0	0	0
2.50	0	0	0	0	0	0	0	0
2.74	0	0	0	0	0	0	0	0
2.11	0	0	0	0	0	0	0	0
2.78	0	0	0	0	0	0	0	0
3.41	0	0	0	0	0	0	0	0
2.57	0	0	0	0	0	0	0	0
2.57	0	0	0	0	0	0	0	0
2.70	0	0	0	0	0	0	0	0
2.81	0	0	0	0	0	0	0	0
2.50	0	0	0	0	0	0	0	0
2.58	1	0	1	0	0	0	0	0
2.79	#NULL!	#NULL!	#NULL!	#NULL!	#NULL!	#NULL!	#NULL!	#NULL!
2.14	0	0	0	0	0	0	0	0
2.53	#NULL!	#NULL!	#NULL!	#NULL!	#NULL!	#NULL!	#NULL!	#NULL!
2.02	1	0	1	0	1	0	0	0
2.49	1	0	0	0	0	0	0	0
2.45	0	0	0	0	0	0	0	0
1.98	1	0	0	0	0	0	0	0
3.26	0	0	0	0	0	0	0	0
#NULL!	1	0	1	0	1	0	0	0
1.74	0	0	0	0	0	0	0	0
1.77	0	0	0	0	0	0	0	0
2.38	0	0	0	0	0	0	0	0
2.34	0	0	0	0	0	0	0	0
2.53	0	0	0	0	0	0	0	0
2.39	0	0	0	0	0	0	0	0
2.74	0	0	0	0	0	0	0	0
#NULL!	0	0	0	0	0	0	0	0
2.21	0	0	0	0	0	0	0	0
2.63	0	0	0	0	0	0	0	0
2.24	0	0	0	0	0	0	0	0
2.69	0	0	0	0	0	0	0	0
#NULL!	1	1	1	1	0	0	0	0
#NULL!	0	0	0	0	0	0	0	0
2.13	0	0	0	0	0	0	0	0
2.17	0	0	0	0	0	0	0	0
1.96	0	0	0	0	0	0	0	0
2.30	1	0	1	0	0	0	1	0
2.42	1	0	1	0	0	0	0	0
2.99	0	0	0	0	0	0	0	0
3.17	0	0	0	0	0	0	0	0
#NULL!	1	0	0	0	0	0	0	0
2.05	0	0	0	0	0	0	0	0
1.50	#NULL!	#NULL!	#NULL!	#NULL!	#NULL!	#NULL!	#NULL!	#NULL!
2.39	1	0	1	0	0	0	1	0
2.14	0	0	0	0	0	0	0	0
2.32	0	0	0	0	0	0	0	0
2.15	#NULL!	#NULL!	#NULL!	#NULL!	#NULL!	#NULL!	#NULL!	#NULL!
2.08	0	0	0	0	0	0	0	0
#NULL!	0	0	0	0	0	0	0	0

2.73	0	0	0	0	0	0	0	0
2.24	0	0	0	0	0	0	0	0
1.96	0	0	0	0	0	0	0	0
2.06	0	0	0	0	0	0	0	0
2.21	#NULL!	#NULL!	#NULL!	#NULL!	#NULL!	#NULL!	#NULL!	#NULL!
2.10	0	0	0	0	0	0	0	0
2.82	0	0	0	0	0	0	0	0
3.11	0	0	0	0	0	0	0	0
#NULL!	1	0	1	0	0	0	0	0
2.48	0	0	0	0	0	0	0	0
2.32	#NULL!	#NULL!	#NULL!	#NULL!	#NULL!	#NULL!	#NULL!	#NULL!
2.17	0	0	0	0	0	0	0	0
2.51	0	0	0	0	0	0	0	0
#NULL!	0	0	0	0	0	0	0	0
2.03	1	0	1	1	0	0	0	0
2.39	0	0	0	0	0	0	0	0
2.20	1	0	1	0	0	0	0	0
#NULL!	0	0	0	0	0	0	0	0
2.29	0	0	0	0	0	0	0	0
2.44	0	0	0	0	0	0	0	0
3.05	#NULL!	#NULL!	#NULL!	#NULL!	#NULL!	#NULL!	#NULL!	#NULL!
2.25	0	0	0	0	0	0	0	0
#NULL!	1	0	1	1	1	0	0	0
2.32	1	0	1	0	0	0	0	0
2.57	0	0	0	0	0	0	0	0
2.77	0	0	0	0	0	0	0	0
1.10	0	0	0	0	0	0	0	0
2.34	0	0	0	0	0	0	0	0
2.32	0	0	0	0	0	0	0	0
2.05	1	0	1	0	0	0	1	0
3.22	#NULL!	#NULL!	#NULL!	#NULL!	#NULL!	#NULL!	#NULL!	#NULL!
2.32	#NULL!	#NULL!	#NULL!	#NULL!	#NULL!	#NULL!	#NULL!	#NULL!
#NULL!	1	0	1	0	0	0	0	0
2.64	1	0	1	0	0	0	0	0
2.61	#NULL!	#NULL!	#NULL!	#NULL!	#NULL!	#NULL!	#NULL!	#NULL!
2.30	0	0	0	0	0	0	0	0
#NULL!	0	0	0	0	0	0	0	0
2.42	0	0	0	0	0	0	0	0
1.85	0	0	0	0	0	0	0	0
2.70	1	0	0	0	1	0	0	0
2.16	0	0	0	0	0	0	0	0
1.98	0	0	0	0	0	0	0	0
1.87	1	0	1	0	0	0	1	0
#NULL!	0	0	0	0	0	0	0	0
2.82	#NULL!	#NULL!	#NULL!	#NULL!	#NULL!	#NULL!	#NULL!	#NULL!
2.23	#NULL!	#NULL!	#NULL!	#NULL!	#NULL!	#NULL!	#NULL!	#NULL!
#NULL!	#NULL!	#NULL!	#NULL!	#NULL!	#NULL!	#NULL!	#NULL!	#NULL!
2.92	1	0	1	1	0	0	1	0
2.40	#NULL!	#NULL!	#NULL!	#NULL!	#NULL!	#NULL!	#NULL!	#NULL!
2.34	0	0	0	0	0	0	0	0
2.96	#NULL!	#NULL!	#NULL!	#NULL!	#NULL!	#NULL!	#NULL!	#NULL!
2.41	0	0	0	0	0	0	0	0
2.29	0	0	0	0	0	0	0	0
2.53	0	0	0	0	0	0	0	0

2.28	#NULL!	#NULL!	#NULL!	#NULL!	#NULL!	#NULL!	#NULL!	#NULL!
1.93	1	0	1	0	0	0	0	0
2.75	0	0	0	0	0	0	0	0
2.29	1	0	0	1	0	0	0	0
2.12	1	0	0	0	0	0	0	0
1.58	1	0	1	0	0	0	0	0
#NULL!	0	0	0	0	0	0	0	0
2.26	0	0	0	0	0	0	0	0
2.25	0	0	0	0	0	0	0	0
#NULL!	0	0	0	0	0	0	0	0
#NULL!	1	0	1	1	0	0	1	0
2.29	0	0	0	0	0	0	0	0
2.80	1	0	1	0	0	0	0	0
2.49	#NULL!	#NULL!	#NULL!	#NULL!	#NULL!	#NULL!	#NULL!	#NULL!
1.84	1	0	0	1	0	0	0	0
2.34	1	0	1	0	0	0	0	0
2.49	0	0	0	0	0	0	0	0
2.63	0	0	0	0	0	0	0	0
2.11	0	0	0	0	0	0	0	0
2.84	#NULL!	#NULL!	#NULL!	#NULL!	#NULL!	#NULL!	#NULL!	#NULL!
2.89	#NULL!	#NULL!	#NULL!	#NULL!	#NULL!	#NULL!	#NULL!	#NULL!
2.89	0	0	0	0	0	0	0	0
2.40	1	0	0	0	0	0	0	0
2.92	0	0	0	0	0	0	0	0
2.29	1	0	1	0	0	0	1	0
2.29	#NULL!	#NULL!	#NULL!	#NULL!	#NULL!	#NULL!	#NULL!	#NULL!
2.35	1	0	1	0	0	0	0	0
#NULL!	#NULL!	#NULL!	#NULL!	#NULL!	#NULL!	#NULL!	#NULL!	#NULL!
#NULL!	#NULL!	#NULL!	#NULL!	#NULL!	#NULL!	#NULL!	#NULL!	#NULL!
2.15	0	0	0	0	0	0	0	0
2.57	0	0	0	0	0	0	0	0
3.37	0	0	0	0	0	0	0	0
2.73	0	0	0	0	0	0	0	0
2.08	0	0	0	0	0	0	0	0
2.50	0	0	0	0	0	0	0	0
#NULL!	0	0	0	0	0	0	0	0
2.41	0	0	0	0	0	0	0	0
2.80	1	0	1	0	0	0	0	0
#NULL!	1	0	1	0	0	0	0	0
2.40	#NULL!	#NULL!	#NULL!	#NULL!	#NULL!	#NULL!	#NULL!	#NULL!
2.02	#NULL!	#NULL!	#NULL!	#NULL!	#NULL!	#NULL!	#NULL!	#NULL!
2.12	1	0	1	0	0	0	0	0
2.58	0	0	0	0	0	0	0	0
2.17	1	0	1	0	0	0	0	0
#NULL!	0	0	0	0	0	0	0	0
2.41	1	0	1	0	0	0	1	0
2.79	1	0	1	0	0	0	0	0
2.93	1	0	1	0	0	0	0	0
#NULL!	0	0	0	0	0	0	0	0
#NULL!	0	0	0	0	0	0	0	0
2.26	0	0	0	0	0	0	0	0
2.65	0	0	0	0	0	0	0	0
2.95	1	0	1	0	0	0	0	0
2.31	1	0	1	0	0	0	0	0

2.60	0	0	0	0	0	0	0	0
1.91	0	0	0	0	0	0	0	0
3.09	1	0	1	0	0	0	0	0
2.19	0	0	0	0	0	0	0	0
2.52	1	0	0	0	0	0	1	0
2.10	#NULL!	#NULL!	#NULL!	#NULL!	#NULL!	#NULL!	#NULL!	#NULL!
2.35	0	0	0	0	0	0	0	0
2.23	0	0	0	0	0	0	0	0
2.73	0	0	0	0	0	0	0	0
2.19	1	0	1	0	0	0	0	0
2.14	0	0	0	0	0	0	0	0
3.31	1	0	0	1	0	0	0	0
2.60	0	0	0	0	0	0	0	0
2.44	0	0	0	0	0	0	0	0
2.24	0	0	0	0	0	0	0	0
2.55	1	0	0	0	0	0	0	0
1.59	0	0	0	0	0	0	0	0
2.27	0	0	0	0	0	0	0	0
2.46	1	0	1	0	0	0	1	0
2.91	0	0	0	0	0	0	0	0
2.35	0	0	0	0	0	0	0	0
#NULL!	1	0	1	1	0	0	0	0
2.36	0	0	0	0	0	0	0	0
2.62	1	0	1	0	0	0	0	0
2.35	1	0	0	1	0	0	0	0
#NULL!	0	0	0	0	0	0	0	0
2.38	0	0	0	0	0	0	0	0
2.65	1	0	1	0	0	0	0	0
2.56	0	0	0	0	0	0	0	0
#NULL!	0	0	0	0	0	0	0	0
2.75	0	0	0	0	0	0	0	0
2.39	0	0	0	0	0	0	0	0
2.41	0	0	0	0	0	0	0	0
2.39	0	0	0	0	0	0	0	0
2.87	0	0	0	0	0	0	0	0
#NULL!	0	0	0	0	0	0	0	0
2.36	#NULL!	#NULL!	#NULL!	#NULL!	#NULL!	#NULL!	#NULL!	#NULL!
2.56	0	0	0	0	0	0	0	0
1.97	1	0	0	1	1	0	0	0
3.27	#NULL!	#NULL!	#NULL!	#NULL!	#NULL!	#NULL!	#NULL!	#NULL!
2.19	0	0	0	0	0	0	0	0
2.77	0	0	0	0	0	0	0	0
2.06	#NULL!	#NULL!	#NULL!	#NULL!	#NULL!	#NULL!	#NULL!	#NULL!
#NULL!	1	0	1	0	0	0	1	0
2.43	0	0	0	0	0	0	0	0
3.37	#NULL!	#NULL!	#NULL!	#NULL!	#NULL!	#NULL!	#NULL!	#NULL!
2.39	0	0	0	0	0	0	0	0
2.32	0	0	0	0	0	0	0	0
2.70	0	0	0	0	0	0	0	0
2.81	#NULL!	#NULL!	#NULL!	#NULL!	#NULL!	#NULL!	#NULL!	#NULL!
2.75	0	0	0	0	#NULL!	0	0	0
#NULL!	0	0	0	0	0	0	0	0
2.19	0	0	0	0	0	0	0	0
2.90	0	0	0	0	0	0	0	0

2.34	0	0	0	0	0	0	0	0
2.42	0	0	0	0	0	0	0	0
2.32	0	0	0	0	0	0	0	0
2.66	0	0	0	0	0	0	0	0
2.44	0	0	0	0	0	0	0	0
2.81	0	0	0	0	0	0	0	0
2.32	1	0	1	0	0	0	0	0
2.48	0	0	0	0	0	0	0	0
#NULL!	0	0	0	0	0	0	0	0
1.86	0	0	0	0	0	0	0	0
2.16	0	0	0	0	0	0	0	0
2.36	1	0	1	0	1	0	0	0
2.94	0	0	0	0	0	0	0	0
#NULL!	1	0	0	0	0	1	0	0
2.19	0	0	0	0	0	0	0	0
2.11	#NULL!	#NULL!	#NULL!	#NULL!	#NULL!	#NULL!	#NULL!	#NULL!
#NULL!	0	0	0	0	0	0	0	0
2.33	#NULL!	#NULL!	#NULL!	#NULL!	#NULL!	#NULL!	#NULL!	#NULL!
2.91	0	0	0	0	0	0	0	0
2.24	0	0	0	0	0	0	0	0
2.60	0	0	0	0	0	0	0	0
2.26	0	0	0	0	0	0	0	0

酮体阳性	大便常规异	大便红细胞	大便白细胞	大便潜血	胃镜阳性	肠镜阳性	内镜阳性	B超
0	0	0	0	0	1	3	1	0
1	0	0	0	0	3	0	0	0
1	1	0	0	1	0	0	0	0
0	0	0	0	0	3	1	1	0
1	0	0	0	0	1	3	1	1
0	0	0	0	0	0	3	0	0
0	0	0	0	0	1	0	1	0
0	0	0	0	0	1	3	1	1
0	0	0	0	0	3	1	1	0
0	0	0	0	0	0	3	0	0
0	1	0	0	1	0	3	0	0
0	0	0	0	0	1	0	1	0
0	0	0	0	0	1	3	1	1
0	0	0	0	0	3	0	0	1
1	0	0	0	0	3	1	1	0
0	0	0	0	0	0	1	1	0
0	1	0	1	1	3	1	1	1
1	0	0	0	0	1	3	1	0
0	1	0	0	1	1	3	1	0
0	0	0	0	0	3	1	1	0
1	0	0	0	0	1	3	1	0
1	1	0	0	1	1	3	1	0
0	0	0	0	0	0	1	1	0
0	0	0	0	0	1	3	1	0
0	0	0	0	0	1	3	1	0
0	0	0	0	0	1	0	1	0
1	1	0	0	1	1	0	1	0
0	0	0	0	0	1	3	1	0
0	0	0	0	0	3	1	1	0
1	1	0	0	1	1	0	1	0
0	1	0	0	1	1	3	1	0
0	0	0	0	0	1	3	1	1
0	0	0	0	0	3	1	1	0
0	0	0	0	0	3	1	1	0
0	0	0	0	0	1	3	1	0
0	0	0	0	0	0	0	0	1
0	0	0	0	0	3	0	0	0
0	0	0	0	0	0	3	0	1
0	0	0	0	0	1	3	1	1
0	0	0	0	0	1	3	1	0
0	0	0	0	0	3	0	0	0
0	0	0	0	0	0	3	0	1
0	0	0	0	0	1	3	1	0
0	0	0	0	0	1	1	1	0
0	1	0	0	1	3	1	1	0
0	0	0	0	0	1	1	1	1
0	0	0	0	1	0	0	0	1
0	0	0	0	0	1	3	1	0
0	0	0	0	0	0	3	0	0
0	0	0	0	0	1	3	1	0
1	0	0	0	0	0	3	0	0
0	1	0	0	1	0	1	1	1
0	0	0	0	0	1	3	1	1

1	1	0	0	1	1	1	1	0
0	0	0	0	0	1	3	1	1
0	1	1	0	1	1	0	1	0
0	1	0	0	1	1	3	1	1
0	0	0	0	0	0	3	0	0
0	0	0	0	0	1	0	1	0
0	0	0	0	0	3	1	1	1
1	0	0	0	0	1	3	1	1
1	0	0	0	0	1	1	1	0
0	0	0	0	0	1	3	1	0
0	1	1	0	1	3	1	1	1
0	1	1	0	1	1	3	1	1
0	0	0	0	0	3	1	1	0
0	0	0	0	0	0	0	0	0
0	0	0	0	0	0	3	0	0
0	1	1	0	1	1	3	1	0
1	1	0	0	1	0	1	1	0
0	0	0	0	0	3	1	1	0
0	0	0	0	0	3	1	1	0
0	1	1	1	1	3	1	1	0
0	1	1	0	0	1	3	1	1
0	0	0	0	0	1	3	1	1
0	0	0	0	0	1	3	1	0
0	0	0	0	0	3	1	1	0
0	0	0	0	0	1	3	1	1
0	0	0	0	0	1	3	1	0
0	0	0	0	0	0	3	0	0
0	0	0	0	0	3	1	1	0
0	1	0	0	1	0	0	0	0
0	1	0	0	1	3	1	1	0
0	0	0	0	0	3	0	0	1
0	0	0	0	0	3	1	1	1
0	0	0	0	0	1	1	1	0
0	1	0	0	1	1	1	1	0
0	1	0	0	1	3	0	0	1
0	0	0	0	0	1	3	1	0
0	0	0	0	0	1	3	1	0
0	1	0	0	1	3	1	1	0
0	0	0	0	0	1	3	1	1
0	0	0	0	0	0	1	1	1
0	0	0	0	0	1	3	1	0
0	0	0	0	0	0	1	1	1
0	0	0	0	0	0	3	0	0
0	0	0	0	0	0	3	0	0
0	0	0	0	0	0	3	0	0
1	0	0	0	0	1	3	1	0
0	0	0	0	0	1	1	1	0
0	0	0	0	0	0	3	0	0
0	0	0	0	0	1	3	1	1
0	0	0	0	0	1	3	1	0
0	0	0	0	0	1	3	1	1
0	0	0	0	0	0	3	0	1
0	0	0	0	0	0	3	0	0
0	1	0	0	1	1	3	1	1

0	1	0	0	1	3	1	1	1
0	0	0	0	0	3	1	1	0
0	0	0	0	0	1	3	1	0
0	0	0	0	0	3	0	0	1
0	0	0	0	0	0	3	0	0
0	1	0	0	1	1	3	1	1
0	0	0	0	0	0	3	0	1
0	1	0	0	1	1	3	1	0
0	1	0	0	1	1	3	1	1
0	0	0	0	0	1	3	1	0
0	0	0	0	0	1	3	1	0
0	0	0	0	0	1	3	1	0
0	0	0	0	0	1	3	1	1
0	0	0	0	0	1	3	1	1
1	0	0	0	0	1	3	1	0
1	0	0	0	0	0	3	0	0
0	0	0	0	0	0	3	0	0
0	0	0	0	0	0	1	1	1
0	0	0	0	0	0	3	0	1
0	0	0	0	0	0	3	0	1
0	0	0	0	0	0	3	0	1
0	0	0	0	0	1	3	1	1
0	1	0	0	1	3	1	1	0
0	0	0	0	0	1	0	1	0
0	1	0	0	1	3	1	1	0
0	1	0	0	1	3	1	1	0
0	0	0	0	0	1	3	1	1
0	0	0	0	0	1	3	1	0
0	0	0	0	0	1	3	1	0
0	1	0	0	1	1	3	1	1
0	0	0	0	0	0	0	0	0
0	1	0	0	1	3	1	1	1
0	0	0	0	0	1	3	1	1
0	0	0	0	0	3	0	0	0
0	1	0	0	1	1	0	1	0
0	0	0	0	0	1	3	1	0
1	0	0	0	0	0	3	0	0
0	0	0	0	0	0	0	0	0
0	0	0	0	0	0	3	0	1
0	0	0	0	0	0	3	0	1
0	1	0	0	1	1	0	1	1
0	0	0	0	0	1	3	1	1
0	0	0	0	0	0	3	0	1
0	1	0	0	1	1	3	1	1
0	1	0	0	1	0	0	0	0
0	1	1	0	1	1	0	1	1
0	0	0	0	0	0	3	0	0
1	0	0	0	0	1	1	1	0
0	0	0	0	0	0	3	0	1
0	0	0	0	0	1	3	1	0
0	0	0	0	0	3	1	1	0
0	0	0	0	0	0	3	0	1
0	1	0	0	1	3	1	1	0
0	0	0	0	0	0	3	0	0

0	0	0	0	0	0	3	0	0
0	1	0	0	1	0	3	0	1
1	1	0	0	1	1	3	1	1
0	0	0	0	0	0	1	1	1
0	1	0	0	1	3	1	1	0
1	0	0	0	0	1	3	1	1
0	0	0	0	0	0	3	0	1
0	0	0	0	0	0	3	0	0
0	0	0	0	0	1	3	1	1
0	0	0	0	0	1	1	1	1
0	0	0	0	0	0	3	0	1
0	0	0	0	0	3	0	0	0
0	1	0	0	1	3	1	1	0
0	0	0	0	0	1	3	1	1
0	0	0	0	0	0	3	0	1
0	0	0	0	0	0	3	0	0
0	0	0	0	0	0	3	0	0
0	0	0	0	0	3	0	0	1
0	0	0	0	0	1	3	1	0
0	0	0	0	0	0	3	0	1
0	0	0	0	0	0	3	0	1
0	1	1	0	1	1	3	1	0
0	0	0	0	0	0	3	0	1
0	0	0	0	0	0	3	0	1
0	0	0	0	0	3	1	1	0
1	1	0	0	1	1	3	1	0
0	0	0	0	0	1	1	1	0
0	0	0	0	0	1	3	1	1
0	0	0	0	0	3	0	0	0
0	0	0	0	0	1	3	1	1
0	0	0	0	0	1	1	1	1
1	0	0	0	0	0	3	0	0
0	1	1	0	1	3	1	1	0
0	0	0	0	0	1	3	1	1
1	0	0	0	0	0	3	0	1
1	1	0	0	1	1	3	1	1
0	1	0	0	1	3	1	1	0
0	0	0	0	0	0	3	0	1
0	0	0	0	0	1	3	1	0
0	0	0	0	0	1	3	1	0
0	0	0	0	0	1	3	1	0
0	0	0	0	0	1	3	1	0
0	0	0	0	0	1	3	1	0
0	0	0	0	0	1	3	1	1
0	0	0	0	0	1	3	1	1
0	0	0	0	0	1	3	1	0
0	0	0	0	0	1	3	1	1
0	0	0	0	0	1	3	1	0
0	0	0	0	0	3	1	1	0
0	0	0	0	0	0	3	0	0
0	0	0	0	0	1	3	1	0
0	0	0	0	0	0	3	0	0
0	1	0	0	1	3	1	1	1
0	0	0	0	0	3	1	1	1

0	0	0	0	0	1	3	1	0
0	0	0	0	0	3	1	1	0
0	0	0	0	0	1	3	1	0
0	1	0	0	1	3	0	0	0
0	0	0	0	0	0	3	0	0
0	0	0	0	0	1	3	1	0
0	0	0	0	0	1	3	1	1
0	1	0	0	1	1	3	1	1
0	0	0	0	0	0	1	1	1
0	0	0	0	0	1	3	1	0
0	1	0	0	1	1	3	1	0
0	0	0	0	0	3	0	0	1
0	0	0	0	0	0	3	0	0
0	0	0	0	0	1	3	1	1
0	0	0	0	0	3	1	1	0
0	0	0	0	0	1	3	1	0
0	0	0	0	0	1	3	1	0
0	0	0	0	0	3	1	1	0
0	0	0	0	0	0	3	0	0
0	1	1	0	1	3	0	0	0
0	0	0	0	0	1	3	1	1
0	0	0	0	0	0	3	0	0
0	0	0	0	0	0	3	0	0
0	0	0	0	0	1	3	1	1
0	1	1	0	1	1	3	1	0
0	0	0	0	0	0	3	0	1
0	0	0	0	0	1	3	1	0
0	0	0	0	0	1	3	1	0
0	0	0	0	0	1	3	1	1
0	1	1	0	1	1	3	1	0
0	0	0	0	0	1	3	1	1
0	0	0	0	0	3	1	1	1
0	0	0	0	0	1	3	1	0
0	1	0	0	1	3	1	1	0
0	0	0	0	0	3	1	1	1
0	0	0	0	0	0	3	0	0
0	1	0	0	1	1	3	1	0
0	0	0	0	0	3	0	0	0
0	0	0	0	0	1	0	1	1
0	0	0	0	0	3	1	1	0
0	0	0	0	0	1	3	1	1
0	0	0	0	0	1	3	1	1
0	1	0	0	1	1	3	1	0
0	0	0	0	0	1	3	1	1
0	1	0	0	1	3	1	1	0
0	0	0	0	0	1	3	1	1
0	0	0	0	0	1	3	1	0
0	0	0	0	0	0	3	0	0
0	0	0	0	0	1	3	1	0
0	0	0	0	0	3	1	1	0
0	1	0	0	1	0	3	0	0
0	0	0	0	0	0	3	0	0
0	0	0	0	0	0	3	0	0
0	1	1	0	1	3	1	1	0

0	1	0	0	1	0	3	0	0
0	0	0	0	0	3	0	0	0
0	0	0	0	0	3	1	1	0
0	1	1	0	1	0	1	1	0
0	0	0	0	0	0	3	0	0
0	1	1	0	1	1	3	1	0
0	0	0	0	0	3	0	0	0
0	0	0	0	0	0	3	0	0
0	0	0	0	0	3	0	0	0
0	0	0	0	0	1	3	1	0
0	1	0	0	1	1	3	1	0
0	0	0	0	0	0	3	0	0
0	1	0	0	1	1	3	1	1
0	0	0	0	0	1	3	1	0
0	1	0	0	1	1	3	1	0
0	0	0	0	0	1	1	1	0
0	1	1	0	1	3	1	1	0
0	1	0	0	1	1	1	1	0
0	1	1	0	1	3	1	1	0
0	0	0	0	0	3	0	0	0
0	0	0	0	0	0	3	0	1
0	0	0	0	0	1	3	1	0
0	0	0	0	0	3	1	1	0
0	0	0	0	0	1	3	1	0
0	0	0	0	0	0	3	0	0
0	0	0	0	0	1	3	1	0
0	0	0	0	0	0	3	0	1
0	0	0	0	0	0	3	0	0
0	0	0	0	0	1	3	1	0
0	0	0	0	0	0	3	0	1
0	0	0	0	0	1	3	1	0
0	0	0	0	0	0	3	0	0
0	0	0	0	0	3	1	1	0
0	1	1	0	1	1	3	1	0
0	0	0	0	0	1	3	1	0
0	0	0	0	0	0	3	0	1
0	0	0	0	0	0	3	0	1
0	0	0	0	0	1	3	1	0
0	1	1	0	1	0	1	1	1
0	0	0	0	0	0	1	1	0
0	0	0	0	0	1	3	1	0
0	0	0	0	0	3	0	0	0
0	1	1	0	1	1	1	1	0
0	1	0	0	1	1	3	1	0
0	0	0	0	0	0	3	0	0
0	0	0	0	0	1	0	0	0
0	0	0	0	0	3	0	0	1
0	0	0	0	0	0	0	0	0
0	1	0	0	1	1	1	1	0
0	0	0	0	0	3	1	1	1
0	0	0	0	0	3	1	1	0
0	0	0	0	0	1	3	1	0
0	1	0	0	1	0	0	0	0
0	1	1	0	1	1	0	1	0

0	0	0	0	0	0	1	1	0
0	1	0	0	1	1	1	1	0
0	0	0	0	0	3	1	1	0
0	0	0	0	0	1	1	1	0
0	0	0	0	0	1	3	1	1
0	0	0	0	0	1	3	1	1
0	1	1	0	1	1	1	1	1
0	0	0	0	0	3	1	1	0
0	0	0	0	0	0	3	0	0
0	0	0	0	0	3	0	0	0
0	0	0	0	0	0	3	0	0
0	0	0	0	0	3	0	0	1
0	1	0	0	1	0	1	1	0
0	0	0	0	0	1	0	1	0
0	0	0	0	0	3	0	0	1
0	0	0	0	0	1	3	1	0
0	0	0	0	0	1	3	1	0
0	0	0	0	0	0	0	0	0
0	0	0	0	0	1	0	1	0
0	0	0	0	0	3	0	0	1
0	1	0	0	1	1	3	1	1
0	0	0	0	0	1	0	1	0
0	0	0	0	0	0	1	1	1
0	0	0	0	0	3	0	0	0
0	0	0	0	0	3	1	1	0
0	0	0	0	0	1	3	1	0
0	0	0	0	0	0	3	0	0
0	0	0	0	0	0	3	0	0
0	0	0	0	0	3	0	0	0
0	0	0	0	0	3	0	0	0
0	0	0	0	0	0	0	0	0
0	0	0	0	0	3	1	1	0
0	0	0	0	0	0	0	0	0
0	0	0	0	0	1	3	1	1
0	0	0	0	0	1	3	1	0
0	0	0	0	0	1	1	1	1
0	0	0	0	0	1	3	1	0
0	1	0	0	1	1	1	1	0
0	0	0	0	0	1	3	1	0
0	0	0	0	0	0	0	0	0
0	0	0	0	0	1	1	1	0
0	0	0	0	0	1	3	1	0
0	0	0	0	0	1	1	1	1
0	0	0	0	0	1	3	1	0
0	0	0	0	0	1	1	1	0
0	0	0	0	0	1	3	1	0
0	0	0	0	0	1	0	1	0
0	0	0	0	0	1	1	1	0
0	0	0	0	0	3	1	1	0
0	0	0	0	0	1	1	1	0
0	0	0	0	0	3	1	1	1
0	0	0	0	0	0	0	0	1
0	0	0	0	0	1	3	1	0
0	0	0	0	0	1	0	1	1

0	0	0	0	0	0	1	1	1
0	0	0	0	0	1	1	1	0
0	0	0	0	0	1	3	1	1
0	0	0	0	0	1	3	1	0
0	0	0	0	0	1	1	1	1
0	0	0	0	0	0	3	0	0
0	0	0	0	0	0	0	0	0
0	0	0	0	0	0	0	0	0
0	0	0	0	0	1	1	1	1
0	0	0	0	0	1	0	1	1
0	0	0	0	0	1	3	1	0
0	0	0	0	0	1	0	1	0
0	0	0	0	0	0	1	1	1
0	1	0	0	1	3	0	0	1
0	0	0	0	0	1	3	1	1
0	0	0	0	0	1	3	1	0
0	1	0	0	1	0	0	0	0
0	0	0	0	0	1	3	1	0
0	0	0	0	0	3	0	0	0
0	0	0	0	0	0	3	0	0
0	0	0	0	0	0	0	0	0
0	1	0	0	1	0	0	0	1
0	0	0	0	0	1	0	1	1
0	0	0	0	0	0	0	0	1
0	0	0	0	0	1	3	1	0
0	1	0	0	1	3	0	0	0
0	0	0	0	0	1	0	1	1
0	0	0	0	0	1	0	1	1
0	0	0	0	0	0	3	0	0
0	0	0	0	0	1	0	1	1
0	0	0	0	0	1	0	1	1
0	0	0	0	0	3	0	0	0
0	0	0	0	0	1	1	1	1
0	0	0	0	0	1	0	1	0
0	0	0	0	0	1	3	1	0
0	0	0	0	0	0	0	0	0
0	0	0	0	0	0	3	0	0
0	0	0	0	0	1	1	1	1
0	0	0	0	0	1	3	1	0
0	1	0	0	1	3	1	1	0
0	0	0	0	0	1	3	1	0
0	0	0	0	0	3	0	0	1
0	0	0	0	0	1	0	1	1
0	0	0	0	0	0	1	1	1
0	1	1	0	1	1	0	1	0
0	1	0	0	1	0	0	0	0
0	0	0	0	0	1	3	1	1
0	0	0	0	0	0	0	0	0
0	0	0	0	0	0	0	0	1
0	0	0	0	0	3	0	0	1
0	0	0	0	0	1	3	1	0
0	0	0	0	0	1	3	1	0
0	0	0	0	0	1	1	1	1
0	1	0	0	1	0	3	0	1

0	0	0	0	0	1	0	1	0
0	0	0	0	0	0	1	1	1
0	0	0	0	0	3	1	1	1
0	0	0	0	0	0	1	1	1
0	0	0	0	0	1	3	1	0
0	0	0	0	0	1	3	1	0
0	0	0	0	0	0	0	0	1
0	1	0	0	1	0	3	0	1
0	0	0	0	0	3	0	0	1
0	0	0	0	0	3	1	1	0
0	0	0	0	0	0	0	0	0
0	0	0	0	0	3	0	0	1
0	0	0	0	0	3	0	0	1
0	0	0	0	0	0	3	0	1
0	0	0	0	0	3	0	0	0
0	0	0	0	0	3	1	1	1
0	0	0	0	0	1	1	1	0
0	0	0	0	0	1	3	1	1
0	0	0	0	0	1	0	1	0
0	0	0	0	0	1	3	1	1
0	0	0	0	0	0	3	0	0
0	0	0	0	0	3	0	0	1
0	0	0	0	0	3	0	0	1
0	0	0	0	0	3	1	1	0
0	0	0	0	0	1	1	1	0
0	0	0	0	0	0	0	0	1
0	0	0	0	0	0	1	1	1
0	0	0	0	0	3	0	0	0
0	0	0	0	0	3	0	0	1
0	0	0	0	0	0	0	0	0
0	0	0	0	0	1	3	1	0
0	0	0	0	0	0	0	0	1
0	0	0	0	0	0	1	1	0
0	0	0	0	0	0	0	0	0
0	0	0	0	0	1	1	1	0
0	0	0	0	0	1	1	1	1
0	0	0	0	0	1	3	1	0
0	0	0	0	0	3	1	1	0
0	0	0	0	0	3	0	0	1
0	0	0	0	0	1	0	1	1
0	0	0	0	0	3	0	0	1
0	0	0	0	0	0	1	1	1
0	0	0	0	0	1	0	1	1
0	0	0	0	0	1	3	1	1
0	0	0	0	0	0	3	0	0
0	0	0	0	0	0	3	0	1
0	0	0	0	0	0	0	0	1
0	0	0	0	0	0	0	0	1
0	0	0	0	0	3	1	1	0
0	0	0	0	0	1	3	1	0
0	1	0	0	1	3	0	0	1
0	1	0	0	1	1	3	1	0
0	0	0	0	0	0	1	1	1
0	0	0	0	0	3	1	1	1

0	0	0	0	0	0	3	0	1
0	0	0	0	0	1	0	1	0
0	0	0	0	0	1	3	1	1
0	0	0	0	0	0	3	0	0
0	0	0	0	0	3	1	1	0
0	1	0	0	1	3	0	0	0
0	0	0	0	0	0	3	0	0
0	0	0	0	0	3	1	1	0
0	0	0	0	0	0	1	1	0
0	0	0	0	0	1	0	1	1
0	0	0	0	0	3	1	1	0
0	0	0	0	0	1	3	1	1
0	0	0	0	0	1	0	1	1
0	0	0	0	0	0	0	0	0
0	1	0	1	0	1	0	1	1
0	0	0	0	0	1	0	1	0
0	1	1	0	1	1	0	1	0
0	0	0	0	0	3	1	1	0
0	0	0	0	0	0	3	0	1
0	1	0	0	1	3	1	1	0
0	0	0	0	0	1	3	1	0
0	1	1	1	1	1	3	1	0
0	0	0	0	0	1	1	1	0
0	0	0	0	0	1	3	1	1
0	0	0	0	0	1	0	1	0
0	0	0	0	0	3	0	0	0
0	0	0	0	0	0	3	0	0
0	0	0	0	0	1	1	1	0
0	0	0	0	0	1	3	1	0
0	0	0	0	0	1	3	1	0
0	1	1	0	1	3	1	1	0
0	0	0	0	0	0	1	1	0
0	0	0	0	0	3	0	0	1
0	0	0	0	0	1	3	1	0
0	1	0	0	1	1	1	1	1
0	0	0	0	0	0	1	1	0
0	0	0	0	0	0	1	1	0
0	0	0	0	0	1	3	1	1
0	0	0	0	0	0	1	1	1
0	0	0	0	0	1	0	1	0
0	0	0	0	0	1	0	1	0
0	0	0	0	0	1	3	1	1
0	0	0	0	0	3	1	1	0
0	0	0	0	0	1	1	1	0
0	0	0	0	0	0	0	0	0
0	0	0	0	0	0	1	1	1
0	0	0	0	0	0	0	0	0
0	0	0	0	0	1	0	1	0
0	0	0	0	0	0	0	0	1
0	0	0	0	0	1	0	1	1
0	0	0	0	0	0	3	0	0
0	0	0	0	0	1	0	1	0
0	1	0	0	1	0	3	0	0
0	0	0	0	0	3	0	0	0

0	0	0	0	0	0	0	0	0
0	0	0	0	0	0	0	0	0
0	0	0	0	0	1	1	1	1
0	0	0	0	0	0	0	0	0
0	1	0	0	1	3	1	1	0
0	1	0	0	1	1	1	1	1
0	0	0	0	0	0	3	0	0
0	0	0	0	0	1	0	1	1
0	0	0	0	0	0	1	1	1
0	0	0	0	0	1	1	1	1
0	0	0	0	0	1	3	1	0
0	0	0	0	0	3	1	1	1
0	1	0	0	1	3	1	1	0
0	0	0	0	0	0	0	0	1
0	0	0	0	0	1	3	1	1
0	0	0	0	0	1	0	1	0
0	0	0	0	0	0	0	0	0
0	0	0	0	0	0	0	0	0
0	0	0	0	0	1	3	1	1
0	0	0	0	0	0	0	0	0
0	0	0	0	0	3	1	1	0
0	0	0	0	0	1	3	1	0
0	0	0	0	0	1	3	1	0
0	0	0	0	0	0	0	0	0
0	0	0	0	0	3	1	1	1
0	0	0	0	0	0	0	0	0
0	0	0	0	0	1	3	1	1
0	0	0	0	0	1	0	1	1
0	0	0	0	0	0	0	0	0
0	0	0	0	0	1	0	1	1
0	0	0	0	0	1	3	1	1
0	0	0	0	0	3	1	1	0
0	1	0	1	1	1	3	1	1
1	0	0	0	0	1	3	1	1
0	0	0	0	0	1	3	1	1
0	0	0	0	0	1	3	1	0
0	0	0	0	0	3	1	1	0
0	0	0	0	0	3	0	0	0
0	0	0	0	0	0	1	1	0
0	0	0	0	0	3	0	0	1
0	0	0	0	0	0	0	0	0
1	0	0	0	0	3	1	1	1
0	0	0	0	0	3	1	1	0
1	0	0	0	0	1	3	1	1
0	0	0	0	0	1	1	1	0
0	1	0	0	1	1	1	1	0
0	0	0	0	0	1	0	1	0
0	0	0	0	0	0	3	0	0
0	0	0	0	0	3	0	0	0
1	0	0	0	0	0	0	0	0
0	0	0	0	0	0	3	0	0
0	0	0	0	0	1	3	1	1
0	0	0	0	0	3	1	1	1
0	0	0	0	0	1	3	1	1

0	0	0	0	0	1	1	1	0
0	1	0	0	1	1	0	1	1
0	0	0	0	0	0	3	0	1
0	0	0	0	0	3	0	0	0
0	0	0	0	0	0	3	0	1
0	0	0	0	0	3	0	0	1
0	0	0	0	0	1	0	1	0
0	0	0	0	0	0	3	0	1
0	0	0	0	0	1	3	1	3
0	1	0	0	1	1	1	1	1
0	1	0	0	1	1	1	1	1
0	0	0	0	0	1	1	1	0
0	0	0	0	0	1	0	1	1
0	1	0	0	1	3	1	1	1
0	0	0	0	0	0	0	0	1
0	0	0	0	0	1	0	1	1
0	0	0	0	0	1	3	1	0
0	0	0	0	0	1	3	1	0
0	0	0	0	0	0	0	0	1
0	1	0	0	1	1	3	1	1
0	1	1	1	1	3	0	0	3
0	0	0	0	0	1	0	1	1
0	0	0	0	0	0	0	0	3
0	0	0	0	0	1	0	1	1
0	0	0	0	0	1	1	1	1
0	0	0	0	0	1	0	1	1
1	1	0	0	1	1	3	1	0
1	0	0	0	0	3	1	1	0
0	0	0	0	0	1	1	1	1
0	0	0	0	0	1	0	1	3
0	1	0	0	1	1	3	1	1
1	1	0	0	1	1	3	1	1
0	0	0	0	0	0	0	0	1
1	0	0	0	0	1	0	1	1
0	0	0	0	0	1	0	1	3
0	0	0	0	0	1	0	1	1
0	0	0	0	0	1	0	1	1
0	0	0	0	0	1	3	1	0
0	0	0	0	0	0	0	0	0
0	0	0	0	0	1	1	1	1
0	0	0	0	0	0	0	0	1
0	0	0	0	0	0	0	0	1
0	0	0	0	0	0	0	0	1
0	0	0	0	0	1	3	1	0
0	0	0	0	0	0	0	0	1
0	0	0	0	0	1	1	1	1
0	0	0	0	0	0	3	0	1
0	0	0	0	0	0	1	1	1
0	0	0	0	0	1	1	1	1
0	0	0	0	0	0	1	1	1
0	0	0	0	0	0	0	0	0
1	1	0	0	1	0	3	0	1
0	0	0	0	0	1	0	1	1
0	1	0	0	1	0	0	0	1

0	0	0	0	0	0	0	0	1
1	1	0	0	1	1	0	1	3
0	0	0	0	0	1	0	1	1
0	0	0	0	0	1	1	1	1
1	0	0	0	0	0	0	0	1
0	0	0	0	0	1	1	1	1
0	0	0	0	0	0	1	1	1
0	0	0	0	0	3	1	1	3
0	0	0	0	0	1	0	1	0
0	0	0	0	0	1	1	1	1
1	0	0	0	0	0	1	1	1
0	0	0	0	0	3	1	1	3
0	0	0	0	0	1	1	1	1
0	0	0	0	0	1	0	1	1
0	1	0	0	1	0	1	1	1
0	0	0	0	0	0	0	0	1
0	0	0	0	0	0	0	0	0
0	0	0	0	0	0	0	0	1
0	0	0	0	0	1	1	1	3
0	0	0	0	0	0	1	1	1
0	0	0	0	0	0	0	0	1
0	0	0	0	0	0	3	0	1
0	0	0	0	0	1	1	1	1
0	1	#NULL!	0	1	1	3	1	1
#NULL!	#NULL!	#NULL!	#NULL!	#NULL!	0	0	0	1
0	#NULL!	#NULL!	#NULL!	#NULL!	1	1	1	0
0	#NULL!	#NULL!	#NULL!	#NULL!	0	1	1	1
0	0	0	0	0	0	1	1	1
0	1	0	0	1	1	1	1	3
0	0	0	0	0	1	3	1	0
#NULL!	0	0	0	0	1	0	1	1
0	0	0	0	0	1	3	1	1
0	0	0	0	0	0	1	1	1
0	0	0	0	0	1	0	1	1
0	0	0	0	0	1	0	1	1
0	0	0	0	0	0	0	0	1
0	1	0	0	1	0	3	0	0
0	0	0	0	0	1	1	1	1
0	0	0	0	0	0	1	1	1
0	1	0	0	1	1	3	1	1
0	0	0	0	0	3	0	0	1
0	0	0	0	0	0	1	1	1
0	0	0	0	0	1	0	1	3
0	0	0	0	0	3	0	0	1
0	1	0	0	1	0	1	1	3
0	0	0	0	0	1	3	1	0
0	0	0	0	0	0	3	0	1
0	0	0	0	0	0	0	0	1
0	#NULL!	#NULL!	#NULL!	#NULL!	0	0	0	1
#NULL!	#NULL!	#NULL!	#NULL!	#NULL!	3	1	1	0
0	0	0	0	0	0	0	0	0
0	0	0	0	0	0	1	1	1
0	0	0	0	0	1	0	1	1
0	0	0	0	0	0	3	0	3

0	0	0	0	0	0	1	1	0
0	0	0	0	0	0	1	1	0
#NULL!	#NULL!	#NULL!	#NULL!	#NULL!	1	1	1	1
0	0	0	0	0	0	1	1	1
0	0	0	0	0	1	0	1	1
0	#NULL!	#NULL!	#NULL!	#NULL!	0	3	0	1
0	0	0	0	0	1	3	1	1
#NULL!	#NULL!	#NULL!	#NULL!	#NULL!	1	0	1	0
0	#NULL!	#NULL!	#NULL!	#NULL!	1	1	1	1
#NULL!	#NULL!	#NULL!	#NULL!	#NULL!	1	1	1	3
#NULL!	0	0	0	0	0	0	0	0
0	0	0	0	0	0	0	0	1
0	0	0	0	0	0	0	0	0
0	1	1	1	0	1	3	1	1
0	0	0	0	0	1	3	1	0
0	0	0	0	0	1	1	1	0
0	0	0	0	0	0	1	1	1
0	0	0	0	0	1	1	1	1
0	0	0	0	0	0	0	0	0
#NULL!	#NULL!	#NULL!	#NULL!	#NULL!	0	0	0	1
0	1	0	0	1	0	0	0	0
#NULL!	#NULL!	#NULL!	#NULL!	#NULL!	1	3	1	1
0	0	0	0	0	0	0	0	1
#NULL!	0	0	0	0	0	1	1	1
0	#NULL!	#NULL!	#NULL!	#NULL!	0	0	0	1
#NULL!	0	0	0	0	0	0	0	0
0	0	0	0	0	0	0	0	3
#NULL!	0	0	0	0	0	0	0	0
0	0	0	0	0	0	1	1	1
#NULL!	0	0	0	0	0	0	0	0
0	0	0	0	0	0	3	0	1
0	0	0	0	0	3	1	1	0
0	0	0	0	0	3	1	1	0
#NULL!	#NULL!	#NULL!	#NULL!	#NULL!	3	0	0	0
#NULL!	#NULL!	#NULL!	#NULL!	#NULL!	0	3	0	0
0	0	0	0	0	0	0	0	0
0	0	0	0	0	0	0	0	1
#NULL!	0	0	0	0	0	0	0	0
0	0	0	0	0	0	0	0	1
0	0	0	0	0	1	3	1	0
0	0	0	0	0	1	0	1	1
0	1	0	0	1	3	0	0	0
0	0	0	0	0	0	1	1	1
0	0	0	0	0	0	0	0	0
0	#NULL!	#NULL!	#NULL!	#NULL!	3	1	1	1
0	0	0	0	0	1	3	1	0
#NULL!	1	0	0	1	0	0	0	0
0	1	0	0	1	1	0	1	0
0	0	0	0	0	1	0	1	3
0	0	0	0	0	3	0	0	1
0	1	0	0	1	0	3	0	0
#NULL!	#NULL!	#NULL!	#NULL!	#NULL!	0	0	0	0
#NULL!	0	0	0	0	1	0	1	1
0	#NULL!	#NULL!	#NULL!	#NULL!	3	1	1	1

0	0	0	0	0	0	0	0	0
0	#NULL!	#NULL!	#NULL!	#NULL!	3	1	1	1
0	0	0	0	0	1	0	1	1
#NULL!	#NULL!	#NULL!	#NULL!	#NULL!	0	1	1	1
0	0	0	0	0	1	1	1	1
0	0	0	0	0	3	1	1	1
0	0	0	0	0	0	0	0	0
0	0	0	0	0	1	3	1	0
0	0	0	0	0	1	0	1	1
0	0	0	0	0	1	3	1	3
0	0	0	0	0	1	0	1	0
0	0	0	0	0	0	0	0	1
0	0	0	0	0	0	0	0	0
0	0	0	0	0	0	0	0	0
0	1	0	0	1	0	1	1	3
0	1	0	0	1	1	3	1	1
0	0	0	0	0	0	0	0	1
0	0	0	0	0	1	1	1	1
0	1	0	0	1	3	1	1	0
0	#NULL!	#NULL!	#NULL!	#NULL!	1	0	0	0
0	0	0	0	0	0	0	0	3
0	1	0	0	1	1	1	1	0
#NULL!	#NULL!	#NULL!	#NULL!	#NULL!	0	0	0	1
#NULL!	0	0	0	0	1	0	1	0
0	1	0	0	1	1	3	1	1
0	0	0	0	0	0	1	1	1
0	0	0	0	0	0	3	0	0
#NULL!	0	0	0	0	0	0	0	0
0	0	0	0	0	0	1	1	0
0	0	0	0	0	0	0	0	1
#NULL!	#NULL!	#NULL!	#NULL!	#NULL!	1	1	1	3
0	#NULL!	#NULL!	#NULL!	#NULL!	0	3	0	1
0	0	0	0	0	1	1	1	0
0	0	0	0	0	1	0	1	0
0	0	0	0	0	1	1	1	1
0	0	0	0	0	0	1	1	1
#NULL!	0	0	0	0	0	0	0	1
0	0	0	0	0	1	0	1	0
0	0	0	0	0	1	0	1	0
0	0	0	0	0	0	0	0	0
1	1	0	0	1	1	3	1	3
0	0	0	0	0	1	1	1	0
#NULL!	#NULL!	#NULL!	#NULL!	#NULL!	1	1	1	1
0	0	0	0	0	0	0	0	1
0	0	0	0	0	1	0	1	1
0	#NULL!	#NULL!	#NULL!	#NULL!	0	0	0	0
0	0	0	0	0	0	1	1	1
0	0	0	0	0	0	0	0	1
0	0	0	0	0	1	0	0	3
0	#NULL!	#NULL!	#NULL!	#NULL!	0	0	0	3
0	0	0	0	0	3	1	1	1
#NULL!	#NULL!	#NULL!	#NULL!	#NULL!	1	0	1	1
0	0	0	0	0	0	0	0	1
0	#NULL!	#NULL!	#NULL!	#NULL!	3	0	0	1

0	0	0	0	0	0	0	0	3
0	1	0	0	1	0	0	0	1
0	0	0	0	0	0	3	0	3
0	#NULL!	#NULL!	#NULL!	#NULL!	1	0	1	1
0	0	0	0	0	0	3	0	0
#NULL!	#NULL!	#NULL!	#NULL!	#NULL!	1	0	1	1
#NULL!	#NULL!	#NULL!	#NULL!	#NULL!	1	0	1	1
0	#NULL!	#NULL!	#NULL!	#NULL!	1	1	1	3
0	0	0	0	0	0	1	1	0
0	0	0	0	0	0	0	0	1
0	#NULL!	#NULL!	#NULL!	#NULL!	1	3	1	0
0	0	0	0	0	0	1	1	0
0	0	0	0	0	3	0	0	1
0	0	0	0	0	1	3	1	3
0	0	0	0	0	0	1	1	1
0	0	0	0	0	1	0	1	0
0	#NULL!	#NULL!	#NULL!	#NULL!	0	1	1	0
0	0	0	0	0	1	0	1	1
0	0	0	0	0	1	1	1	1
0	0	0	0	0	0	0	0	1
0	0	0	0	0	1	3	1	3
0	#NULL!	#NULL!	#NULL!	#NULL!	1	0	1	0
0	0	0	0	0	1	3	1	1
0	0	0	0	0	0	0	0	0
0	0	0	0	0	1	0	1	0
#NULL!	#NULL!	#NULL!	#NULL!	#NULL!	0	0	0	1
0	1	0	0	1	0	1	1	0
#NULL!	#NULL!	#NULL!	#NULL!	#NULL!	0	0	0	1
0	0	0	0	0	1	0	0	1
0	0	0	0	0	1	0	1	1
0	0	0	0	0	3	0	0	1
0	0	0	0	0	0	0	0	1
0	1	0	0	1	1	1	1	0
0	0	0	0	0	1	0	1	1
0	0	0	0	0	3	0	0	1
1	0	0	0	0	0	1	1	0
0	0	0	0	0	3	0	0	1
0	0	0	0	0	3	0	0	0
0	0	0	0	0	0	0	0	1
0	0	0	0	0	0	0	0	0
0	0	0	0	0	0	1	1	1
0	0	0	0	0	1	3	1	0
0	0	0	0	0	1	0	1	3
#NULL!	#NULL!	#NULL!	#NULL!	#NULL!	0	0	0	0
0	0	0	0	0	1	0	1	1
0	0	0	0	0	1	0	1	1
#NULL!	#NULL!	#NULL!	#NULL!	#NULL!	1	0	1	1
0	0	0	0	0	1	0	1	1
0	0	0	0	0	0	3	0	3
1	0	0	0	0	1	0	1	1
1	0	0	0	0	1	0	1	1
0	#NULL!	#NULL!	#NULL!	#NULL!	0	0	0	1
1	#NULL!	#NULL!	#NULL!	#NULL!	3	0	0	1
0	0	0	0	0	1	0	1	1

0	#NULL!	#NULL!	#NULL!	#NULL!	1	3	1	3
0	0	0	0	0	0	1	1	1
0	1	0	0	1	3	1	1	3
1	0	0	0	0	1	1	1	1
0	#NULL!	#NULL!	#NULL!	#NULL!	0	1	1	1
0	0	0	0	0	3	0	0	0
#NULL!	0	0	0	0	1	3	1	1
0	0	0	0	0	1	0	1	0
0	1	0	0	1	1	1	1	0
#NULL!	#NULL!	#NULL!	#NULL!	#NULL!	1	0	1	1
0	1	0	0	1	0	0	0	0
0	0	0	0	0	0	0	0	3
0	0	0	0	0	1	0	1	0
0	1	0	0	1	0	1	1	1
0	0	0	0	0	0	1	1	0
0	0	0	0	0	1	3	1	1
1	0	0	0	0	0	1	1	1
0	0	0	0	0	0	0	0	1
0	0	0	0	0	0	1	1	0
0	0	0	0	0	1	3	1	1
0	0	0	0	0	1	0	1	3
0	0	0	0	0	0	0	0	1
0	0	0	0	0	1	0	1	1
1	0	0	0	0	0	0	0	3
0	0	0	0	0	0	0	0	1
0	0	0	0	0	1	0	1	0
1	1	0	0	1	3	1	1	0
0	0	0	0	0	1	0	1	0
0	0	0	0	0	0	0	0	1
#NULL!	#NULL!	#NULL!	#NULL!	#NULL!	0	0	0	1
0	#NULL!	#NULL!	#NULL!	#NULL!	0	0	0	1
0	0	0	0	0	0	1	1	1
0	0	0	0	0	1	1	1	0
0	0	0	0	0	0	3	0	1
0	1	0	0	1	0	0	0	1
0	0	0	0	0	1	3	1	1
0	0	0	0	0	1	3	1	0
1	0	0	0	0	1	1	1	1
0	1	0	0	1	1	0	1	1
0	0	0	0	0	0	0	0	0
0	0	0	0	0	1	0	1	0
1	#NULL!	#NULL!	#NULL!	#NULL!	0	1	1	3
0	0	0	0	0	3	0	0	0
#NULL!	0	0	0	0	1	3	1	0
0	0	0	0	0	0	3	0	0
0	0	0	0	0	1	1	1	1
0	0	0	0	0	0	0	0	0
#NULL!	#NULL!	#NULL!	#NULL!	#NULL!	3	0	0	1
0	0	0	0	0	0	1	1	1
0	1	0	0	1	1	1	1	3
0	0	0	0	0	1	3	1	1
0	0	0	0	0	3	0	0	0
0	0	0	0	0	3	1	1	3
0	1	0	0	1	3	0	0	0

0	1	0	0	1	0	1	1	1
0	1	0	0	1	1	1	1	1
#NULL!	#NULL!	#NULL!	#NULL!	#NULL!	1	1	1	1
0	1	0	0	1	1	0	1	1
0	0	0	0	0	1	0	1	0
0	0	0	0	0	1	3	1	1
0	0	0	0	0	1	1	1	1
0	0	0	0	0	0	0	0	1
0	0	0	0	0	3	0	0	1
0	1	0	0	1	3	1	1	3
0	0	0	0	0	0	0	0	3
0	0	0	0	0	3	0	0	0
#NULL!	#NULL!	#NULL!	#NULL!	#NULL!	1	1	1	0
0	0	0	0	0	0	3	0	1
0	0	0	0	0	3	0	0	3
0	0	0	0	0	1	1	1	1
0	0	0	0	0	1	1	1	0
#NULL!	#NULL!	#NULL!	#NULL!	#NULL!	0	3	0	1
1	0	0	0	0	0	0	0	1
0	0	0	0	0	0	0	0	0
0	0	0	0	0	0	0	0	0
0	0	0	0	0	1	1	1	1
0	0	0	0	0	3	0	0	1
0	0	0	0	0	1	0	1	0
0	0	0	0	0	0	0	0	1
0	#NULL!	#NULL!	#NULL!	#NULL!	3	1	1	3
0	0	0	0	0	1	1	1	0
0	0	0	0	0	0	0	0	1
0	0	0	0	0	1	1	1	1
0	0	0	0	0	1	0	1	1
0	1	0	0	1	1	1	1	1
0	0	0	0	0	0	0	0	1
0	0	0	0	0	1	1	1	1
0	0	0	0	0	0	0	0	1
0	0	0	0	0	1	0	1	1
0	0	0	0	0	0	1	1	1
0	0	0	0	0	0	0	0	1
0	1	0	0	1	1	3	0	1
0	0	0	0	0	1	1	1	1
0	0	0	0	0	3	1	1	0
0	0	0	0	0	1	0	1	1
0	1	0	0	1	1	0	1	1
0	0	0	0	0	0	0	0	1
0	0	0	0	0	0	3	0	1
0	0	0	0	0	0	0	0	0
0	0	0	0	0	0	1	1	1
0	0	0	0	0	1	1	1	1
0	0	0	0	0	1	1	1	0
0	0	0	0	0	1	3	1	3
1	0	0	0	0	0	0	0	1
0	0	0	0	0	1	0	1	1
0	0	0	0	0	0	1	1	0
0	0	0	0	0	1	0	1	1
0	0	0	0	0	0	1	1	1

0	0	0	0	0	0	0	0	1
0	0	0	0	0	1	0	1	0
0	0	0	0	0	1	0	1	1
1	1	1	1	1	1	1	1	1
0	0	0	0	0	0	0	0	1
1	0	0	0	0	0	1	1	1
0	0	0	0	0	1	1	1	0
0	0	0	0	0	1	0	1	3
0	0	0	0	0	0	0	0	1
0	0	0	0	0	0	1	1	1
0	0	0	0	0	1	0	1	0
0	0	0	0	0	0	0	0	0
0	0	0	0	0	1	0	1	0
0	0	0	0	0	1	1	1	0
0	0	0	0	0	0	0	0	0
0	0	0	0	0	1	0	1	1
0	0	0	0	0	1	3	1	3
0	0	0	0	0	1	0	1	1
0	0	0	0	0	0	0	0	1
0	0	0	0	0	1	1	1	0
0	0	0	0	0	1	0	1	1
0	0	0	0	0	1	0	1	1
0	0	0	0	0	0	0	0	3
0	#NULL!	#NULL!	#NULL!	#NULL!	1	0	1	3
0	0	0	0	0	0	1	1	3
#NULL!	#NULL!	#NULL!	#NULL!	#NULL!	0	0	0	1
0	0	0	0	0	0	0	0	0
0	0	0	0	0	0	0	0	1
0	0	0	0	0	1	0	1	3
0	0	0	0	0	0	1	1	1
#NULL!	#NULL!	#NULL!	#NULL!	#NULL!	1	0	1	1
1	0	0	0	0	1	0	1	1
0	0	0	0	0	0	0	0	3
0	0	0	0	0	1	0	1	1
0	0	0	0	0	0	0	0	1
0	0	0	0	0	0	1	1	0
#NULL!	#NULL!	#NULL!	#NULL!	#NULL!	0	0	0	1
0	0	0	0	0	0	0	0	1
0	0	0	0	0	1	3	1	1
0	1	0	0	1	0	1	1	1
1	1	1	1	1	3	1	1	3
#NULL!	0	0	0	0	0	1	1	0
#NULL!	0	0	0	0	0	1	1	1
#NULL!	1	0	0	1	0	1	1	0
0	0	0	0	0	1	0	1	1
0	0	0	0	0	1	0	1	3
0	0	0	0	0	0	0	0	1
1	0	0	0	0	1	3	1	3
0	0	0	0	0	1	0	1	0
0	0	0	0	0	0	3	0	1
0	0	0	0	0	0	1	1	0
0	0	0	0	0	0	1	1	0
0	0	0	0	0	1	1	1	1
0	0	0	0	0	1	1	1	1

0	1	0	0	1	0	3	0	1
0	0	0	0	0	0	1	1	1
0	0	0	0	0	3	1	1	3
0	0	0	0	0	0	0	0	0
0	0	0	0	0	3	0	0	1
0	0	0	0	0	3	0	0	0
0	0	0	0	0	1	0	0	1
0	0	0	0	0	0	0	0	1
0	0	0	0	0	1	3	1	1
0	0	0	0	0	1	3	1	1
0	0	0	0	0	1	1	1	1
1	1	0	0	1	1	1	1	1
0	1	0	0	1	0	1	1	1
0	1	0	0	1	1	1	1	1
0	0	0	0	0	0	0	0	1
0	0	0	0	0	3	0	0	0
0	0	0	0	0	1	0	1	1
0	0	0	0	0	0	1	1	1
0	0	0	0	0	3	0	0	1
1	1	0	0	1	1	3	1	1
0	1	0	0	1	0	0	0	0
0	0	0	0	0	0	3	0	1
1	#NULL!	#NULL!	#NULL!	#NULL!	1	3	1	1
0	0	0	0	0	0	0	0	1
0	1	0	0	1	0	0	0	1
0	0	0	0	0	1	0	1	1
0	0	0	0	0	0	1	1	0
0	0	0	0	0	0	0	0	1
0	0	0	0	0	0	0	0	0
0	#NULL!	#NULL!	#NULL!	#NULL!	1	3	1	3
0	0	0	0	0	3	0	0	1
0	0	0	0	0	0	1	1	1
1	0	0	0	0	3	0	0	1
0	0	0	0	0	0	0	0	0
0	#NULL!	#NULL!	#NULL!	#NULL!	0	3	0	1
0	0	0	0	0	0	1	1	0
0	0	0	0	0	3	0	0	1
0	0	0	0	0	0	1	1	3
0	0	0	0	0	1	0	1	1
1	1	0	0	1	0	3	0	1
0	1	0	0	1	0	1	1	1
0	0	0	0	0	0	0	0	1
0	0	0	0	0	0	1	1	1
0	0	0	0	0	1	3	1	3
0	0	0	0	0	0	1	1	1
0	0	0	0	0	0	0	0	0
0	0	0	0	0	0	0	0	0
0	0	0	0	0	1	1	1	1
0	0	0	0	0	0	0	0	1
0	1	0	0	1	1	3	1	1
0	0	0	0	0	0	0	0	1
0	0	0	0	0	1	3	1	0
0	0	0	0	0	0	1	1	1
0	0	0	0	0	0	0	0	0

0	0	0	0	0	0	0	0	0
0	0	0	0	0	0	1	1	0
0	0	0	0	0	0	0	0	1
0	0	0	0	0	1	0	1	0
0	1	0	0	1	1	0	1	1
0	0	0	0	0	0	0	0	1
1	0	0	0	0	1	1	1	1
0	0	0	0	0	1	1	1	0
0	0	0	0	0	1	0	1	1
0	0	0	0	0	0	0	0	0
0	1	0	0	1	1	1	1	1
0	1	0	0	1	1	1	1	1
0	0	0	0	0	0	0	0	1
0	0	0	0	0	0	0	0	0
0	1	0	0	1	0	0	0	1
0	0	0	0	0	0	0	0	1
0	0	0	0	0	0	0	0	1
0	0	0	0	0	0	0	0	1
1	1	0	0	1	0	3	0	1
#NULL!	#NULL!	#NULL!	#NULL!	#NULL!	1	3	1	3
0	0	0	0	0	0	1	1	1
1	1	0	0	1	1	0	1	1
0	0	0	0	0	1	0	1	1
0	0	0	0	0	0	0	0	1
0	0	0	0	0	1	0	1	1
0	0	0	0	0	0	0	0	0
0	0	0	0	0	0	0	0	1
0	0	0	0	0	1	0	1	0
0	0	0	0	0	3	0	0	0
1	0	0	0	0	3	1	1	3
0	1	0	0	1	3	1	1	1
0	1	0	0	1	1	0	1	1
0	1	0	0	1	3	1	1	3
0	0	0	0	0	0	0	0	1
0	0	0	0	0	0	0	0	0
0	0	0	0	0	3	0	0	0
0	0	0	0	0	0	0	0	1
0	0	0	0	0	3	1	1	1
0	0	0	0	0	0	1	1	0
0	0	0	0	0	3	1	1	0
0	1	0	0	1	0	3	0	1
0	1	0	0	1	0	0	0	1
0	1	0	0	1	0	1	1	1
0	1	0	0	1	0	0	0	1
0	0	0	0	0	0	3	0	1
0	0	0	0	0	0	0	0	1
0	1	0	0	1	3	0	0	1
1	0	0	0	0	0	3	0	1
0	0	0	0	0	3	1	1	3
0	0	0	0	0	1	0	1	1
0	0	0	0	0	1	0	1	3
0	0	0	0	0	0	3	0	1
0	0	0	0	0	1	3	1	0
0	0	0	0	0	1	1	1	1

0	0	0	0	0	1	3	1	1
0	0	0	0	0	0	0	0	0
0	0	0	0	0	0	0	0	0
1	0	0	0	0	1	1	1	0
0	1	0	0	1	1	0	1	1
0	0	0	0	0	0	0	0	1
0	0	0	0	0	1	0	1	1
0	0	0	0	0	0	0	0	1
0	0	0	0	0	1	1	1	3
0	0	0	0	0	1	3	1	1
0	0	0	0	0	0	1	1	3
0	0	0	0	0	0	1	1	0
0	0	0	0	0	3	1	1	0
0	0	0	0	0	0	1	1	0
0	1	0	0	1	1	1	1	1
0	0	0	0	0	3	0	0	1
0	0	0	0	0	1	1	1	1
0	0	0	0	0	0	3	0	0
0	0	0	0	0	1	0	1	1
0	0	0	0	0	0	0	0	3
0	0	0	0	0	0	0	0	0
0	0	0	0	0	1	1	1	1
0	0	0	0	0	1	0	1	1
1	1	0	0	1	1	1	1	0
0	1	0	0	1	1	1	1	3
0	0	0	0	0	1	3	1	0
0	0	0	0	0	0	0	0	1
0	0	0	0	0	0	3	0	3
0	0	0	0	0	1	0	1	1
0	0	0	0	0	0	0	0	1
1	1	0	0	1	1	3	1	1
0	0	0	0	0	1	1	1	1
0	0	0	0	0	1	0	1	0
0	0	0	0	0	1	1	1	1
0	0	0	0	0	3	0	0	1
0	0	0	0	0	0	3	0	1
0	0	0	0	0	1	3	1	3
1	1	0	0	1	1	1	1	3
0	0	0	0	0	1	0	1	0
0	0	0	0	0	1	1	1	1
0	0	0	0	0	1	0	1	1
0	0	0	0	0	1	0	1	1
0	0	0	0	0	1	1	1	1
0	0	0	0	0	1	1	1	1
0	0	0	0	0	1	0	1	0
0	0	0	0	0	3	1	1	1
0	0	0	0	0	0	1	1	0
0	0	0	0	0	0	1	1	0
0	0	0	0	0	1	1	1	1
0	0	0	0	0	1	0	1	1
1	0	0	0	0	1	0	1	0
0	0	0	0	0	1	0	1	1
0	0	0	0	0	0	0	0	1
0	0	0	0	0	3	0	0	3

0	0	0	0	0	3	1	1	1
0	1	0	1	1	3	1	1	1
0	0	0	0	0	1	1	1	1
0	0	0	0	0	3	0	0	0
0	0	0	0	0	1	1	1	1
0	1	0	0	1	0	1	1	1
0	0	0	0	0	3	0	0	1
0	0	0	0	0	1	0	1	1
0	0	0	0	0	1	0	1	0
0	0	0	0	0	0	0	0	1
0	0	0	0	0	0	0	0	0
0	0	0	0	0	0	0	0	0
0	0	0	0	0	0	0	0	0
0	1	0	0	1	0	1	1	1
1	#NULL!	#NULL!	#NULL!	#NULL!	1	3	1	1
#NULL!	0	0	0	0	1	1	1	0
0	1	1	0	1	0	1	1	0
#NULL!	#NULL!	#NULL!	#NULL!	#NULL!	0	3	0	0
1	#NULL!	#NULL!	#NULL!	#NULL!	1	3	1	0
1	1	0	1	0	1	3	1	3
0	0	0	0	0	3	0	0	0
1	1	0	1	1	3	1	1	1
0	#NULL!	#NULL!	#NULL!	#NULL!	1	1	1	3
0	0	0	0	0	1	1	1	1
0	#NULL!	#NULL!	#NULL!	#NULL!	1	3	1	3
0	1	0	0	1	0	1	1	1
0	0	0	0	0	0	0	0	0
0	0	0	0	0	1	0	1	3
0	0	0	0	0	1	1	1	0
0	0	0	0	0	1	0	1	1
0	0	0	0	0	1	0	1	0
0	1	0	0	1	1	3	1	1
0	0	0	0	0	1	0	1	3
0	0	0	0	0	0	1	1	0
0	1	0	1	1	1	3	1	0
0	1	0	0	1	0	0	0	1
0	1	0	0	1	1	3	1	3
0	0	0	0	0	0	0	0	1
0	0	0	0	0	0	0	0	0
0	0	0	0	0	1	0	1	0
0	0	0	0	0	0	1	1	1
0	1	0	1	1	1	0	1	0
0	#NULL!	#NULL!	#NULL!	#NULL!	0	0	0	0
0	0	0	0	0	1	0	1	0
0	0	0	0	0	3	1	1	0
1	0	0	0	0	1	3	1	0
0	0	0	0	0	1	0	1	1
#NULL!	1	0	0	1	1	1	1	1
0	0	0	0	0	0	0	0	1
0	0	0	0	0	1	1	1	0
0	0	0	0	0	1	1	1	1
#NULL!	#NULL!	#NULL!	#NULL!	#NULL!	0	1	1	0
0	#NULL!	#NULL!	#NULL!	#NULL!	1	3	1	3
0	#NULL!	#NULL!	#NULL!	#NULL!	1	3	1	3

0	0	0	0	0	0	0	0	0
0	0	0	0	0	3	1	1	1
0	0	0	0	0	1	0	1	0
0	0	0	0	0	0	0	0	0
#NULL!	0	0	0	0	1	0	1	1
0	0	0	0	0	1	0	1	0
0	0	0	0	0	1	0	1	0
0	1	0	1	0	1	1	1	0
1	0	0	0	0	3	1	1	1
0	0	0	0	0	3	0	0	1
#NULL!	#NULL!	#NULL!	#NULL!	#NULL!	1	0	1	1
0	0	0	0	0	0	0	0	0
0	0	0	0	0	3	0	0	0
0	0	0	0	0	3	1	1	1
1	1	0	0	1	0	0	0	1
0	0	0	0	0	1	1	1	1
0	0	0	0	0	1	0	1	1
0	0	0	0	0	3	1	1	0
0	0	0	0	0	3	1	1	3
0	0	0	0	0	1	0	1	1
#NULL!	#NULL!	#NULL!	#NULL!	#NULL!	1	0	1	1
0	0	0	0	0	0	0	0	1
0	0	0	0	0	3	1	1	1
1	0	0	0	0	0	0	0	1
0	0	0	0	0	3	0	0	0
0	0	0	0	0	1	1	1	3
0	1	0	0	1	1	0	1	3
0	#NULL!	#NULL!	#NULL!	#NULL!	1	0	1	0
0	0	0	0	0	1	1	1	1
0	0	0	0	0	0	0	0	0
#NULL!	0	0	0	0	1	1	1	1
#NULL!	1	0	0	1	1	0	1	0
1	0	0	0	0	1	3	1	3
0	0	0	0	0	0	1	1	1
#NULL!	#NULL!	#NULL!	#NULL!	#NULL!	1	0	1	0
0	0	0	0	0	1	0	0	0
0	0	0	0	0	0	3	0	1
0	0	0	0	0	0	1	1	0
0	1	0	0	1	0	0	0	1
0	0	0	0	0	0	3	0	1
0	0	0	0	0	1	0	1	1
0	0	0	0	0	0	1	1	0
0	0	0	0	0	0	0	0	0
0	0	0	0	0	1	1	1	1
#NULL!	1	0	0	1	0	1	1	0
#NULL!	#NULL!	#NULL!	#NULL!	#NULL!	0	0	0	0
#NULL!	#NULL!	#NULL!	#NULL!	#NULL!	1	1	1	3
0	1	0	0	1	0	0	0	1
#NULL!	#NULL!	#NULL!	#NULL!	#NULL!	1	1	1	1
0	0	0	0	0	1	0	1	1
#NULL!	#NULL!	#NULL!	#NULL!	#NULL!	1	1	1	1
0	0	0	0	0	0	0	0	0
0	0	0	0	0	1	3	1	0
0	1	0	0	1	1	3	1	1

#NULL!	0	0	0	0	1	1	1	0
1	0	0	0	0	0	0	0	1
0	#NULL!	#NULL!	#NULL!	#NULL!	1	3	1	1
0	0	0	0	0	1	0	1	0
1	0	0	0	0	0	3	0	0
0	#NULL!	#NULL!	#NULL!	#NULL!	1	3	1	1
0	0	0	0	0	1	0	1	1
0	0	0	0	0	0	3	0	0
0	0	0	0	0	1	0	1	3
0	0	0	0	0	1	0	1	0
0	0	0	0	0	0	1	1	1
0	0	0	0	0	3	0	0	1
0	0	0	0	0	0	0	0	0
#NULL!	0	0	0	0	3	1	1	1
0	1	0	0	1	1	1	1	1
0	0	0	0	0	1	0	1	0
0	0	0	0	0	0	0	0	0
0	0	0	0	0	0	0	0	1
0	0	0	0	0	0	0	0	1
#NULL!	#NULL!	#NULL!	#NULL!	#NULL!	1	0	1	1
#NULL!	#NULL!	#NULL!	#NULL!	#NULL!	0	0	0	0
0	#NULL!	#NULL!	#NULL!	#NULL!	0	0	0	1
1	0	0	0	0	0	0	0	0
0	0	0	0	0	1	0	1	0
0	#NULL!	#NULL!	#NULL!	#NULL!	1	1	1	1
#NULL!	#NULL!	#NULL!	#NULL!	#NULL!	0	0	0	0
0	0	0	0	0	0	1	1	1
#NULL!	#NULL!	#NULL!	#NULL!	#NULL!	1	3	1	3
#NULL!	#NULL!	#NULL!	#NULL!	#NULL!	1	3	1	3
0	0	0	0	0	1	0	1	1
0	0	0	0	0	0	0	0	1
0	#NULL!	#NULL!	#NULL!	#NULL!	1	1	1	0
0	0	0	0	0	0	0	0	1
0	0	0	0	0	1	3	1	1
0	0	0	0	0	1	0	1	3
0	0	0	0	0	1	0	1	0
0	0	0	0	0	0	0	0	1
0	1	0	1	0	1	1	1	1
0	0	0	0	0	0	0	0	1
#NULL!	1	0	0	1	1	1	1	0
#NULL!	#NULL!	#NULL!	#NULL!	#NULL!	0	1	1	0
1	1	0	1	1	1	1	1	1
0	0	0	0	0	1	1	1	0
0	0	0	0	0	1	1	1	3
0	0	0	0	0	1	0	1	1
0	0	0	0	0	1	0	1	1
0	0	0	0	0	1	0	1	1
1	0	0	0	0	0	0	0	0
0	0	0	0	0	1	3	1	1
0	0	0	0	0	3	1	1	1
0	0	0	0	0	0	1	1	0
0	0	0	0	0	1	0	1	1
1	0	0	0	0	0	1	1	0
0	0	0	0	0	1	0	1	1

0	0	0	0	0	0	0	0	1
0	0	0	0	0	0	1	1	0
0	1	0	0	1	0	1	1	1
0	1	0	0	1	3	1	1	1
0	0	0	0	0	1	0	1	0
#NULL!	0	0	0	0	1	0	1	0
0	#NULL!	#NULL!	#NULL!	#NULL!	0	0	0	0
0	0	0	0	0	0	1	1	1
0	0	0	0	0	0	0	0	0
0	0	0	0	0	1	0	1	0
0	0	0	0	0	0	0	0	0
0	#NULL!	#NULL!	#NULL!	#NULL!	1	0	1	0
0	0	0	0	0	1	3	1	1
0	0	0	0	0	1	1	1	0
0	0	0	0	0	0	0	0	1
1	0	0	0	0	1	0	1	0
0	0	0	0	0	0	1	1	1
0	0	0	0	0	1	1	1	1
0	1	0	1	0	0	1	1	1
0	0	0	0	0	1	3	1	0
0	0	0	0	0	1	3	1	0
0	1	0	1	0	1	0	1	0
0	0	0	0	0	1	1	1	3
0	0	0	0	0	0	3	0	0
0	0	0	0	0	0	0	0	0
0	0	0	0	0	3	1	1	0
0	0	0	0	0	1	1	1	0
0	0	0	0	0	1	0	1	0
0	#NULL!	#NULL!	#NULL!	#NULL!	0	0	0	0
0	1	0	1	0	0	3	0	3
0	0	0	0	0	0	1	1	0
0	0	0	0	0	0	0	0	1
0	#NULL!	#NULL!	#NULL!	#NULL!	1	0	1	0
0	0	0	0	0	1	1	1	0
0	0	0	0	0	0	3	0	0
0	0	0	0	0	1	1	1	1
#NULL!	#NULL!	#NULL!	#NULL!	#NULL!	1	0	1	1
0	0	0	0	0	3	0	1	1
1	0	0	0	0	1	0	1	3
#NULL!	#NULL!	#NULL!	#NULL!	#NULL!	1	0	1	1
0	0	0	0	0	1	0	1	1
0	0	0	0	0	0	0	0	1
#NULL!	0	0	0	0	0	0	0	0
0	0	0	0	0	1	3	1	1
0	0	0	0	0	1	1	1	0
#NULL!	#NULL!	#NULL!	#NULL!	#NULL!	0	1	1	3
0	0	0	0	0	1	0	1	3
0	1	0	1	0	0	0	0	1
0	#NULL!	#NULL!	#NULL!	#NULL!	1	0	1	0
#NULL!	0	0	0	0	1	1	1	1
0	0	0	0	0	1	0	1	1
0	0	0	0	0	0	0	0	0
0	0	0	0	0	1	0	1	0
0	#NULL!	#NULL!	#NULL!	#NULL!	1	0	1	1

0	0	0	0	0	0	0	0	1
0	0	0	0	0	1	0	1	1
0	0	0	0	0	0	1	1	1
0	0	0	0	0	0	0	0	1
0	0	0	0	0	1	3	1	1
0	0	0	0	0	0	1	1	1
0	0	0	0	0	1	0	1	0
0	#NULL!	#NULL!	#NULL!	#NULL!	1	0	1	1
0	1	0	1	0	0	0	0	1
0	#NULL!	#NULL!	#NULL!	#NULL!	1	0	1	1
0	0	0	0	0	0	0	0	1
0	0	0	0	0	0	0	0	3
0	0	0	0	0	0	1	1	0
0	0	0	0	0	1	3	1	1
0	0	0	0	0	1	0	1	1
#NULL!	#NULL!	#NULL!	#NULL!	#NULL!	0	0	0	0
0	0	0	0	0	3	1	1	3
#NULL!	#NULL!	#NULL!	#NULL!	#NULL!	1	0	1	1
0	0	0	0	0	0	1	1	0
0	0	0	0	0	1	0	1	1
0	0	0	0	0	1	0	1	0
0	0	0	0	0	3	0	0	1

影像阳性
0
0
0
0
0
0
0
0
0
0
0
0
1
0
0
0
0
0
0
0
1
0
0
0
0
0
0
0
0
0
0
0
0
0
0
0
0
0
0
0
0
1
0
0
0
0
0
0
1
1
1
1
1

1
1
1
1
0
0
1
0
1
0
1
1
1
1
0
0
1
0
1
0
1
0
1
1
0
0
0
1
1
0
1
0
1
1
0
0
0
0
1
1
0
1
0
0
1
1
1
1
1
0
1
1
0
0

0
0
0
1
0
0
1
0
0
1
0
0
0
0
0
0
0
1
1
1
0
0
0
0
0
0
1
1
1
1
1
0
1
1
0
0
0
1
1
1
1
0
0
0
1
1
0
1
1
0
0
1
0
0

0
0
0
0
0
0
1
0
1
1
0
0
0
1
1
1
1
1
0
0
0
0
1
1
0
0
1
0
1
1
1
1
1
0
0
1
0
1
0
0
1
0
1
1
1
0
1
0
0
0
0
0
0
0

0
0
1
0
0
1
1
1
0
0
1
0
0
0
0
0
1
0
0
0
1
0
0
1
1
0
1
0
0
0
1
0
0
0
1
0
0
0
1
0
1
0
0
0
0
1
0
1
0
0
0
1
0
0

1
1
0
0
1
1
0
1
1
1
0
1
0
0
1
1
1
1
0
1
1
1
0
0
1
0
0
1
0
0
0
0
0
0
0
1
1
0
1
0
0
1
1
1
1
0
1
0
1
0
1
1
1
1

0
1
1
1
0
1
0
1
1
0
1
1
1
0
1
1
0
0
1
0
0
0
0
1
1
0
1
0
1
0
0
0
0
1
0
0
1
1
0
0
0
0
1
1
0
0
0
0
1
0
0
1
1
0

0
0
1
1
1
1
0
0
1
0
0
1
0
1
0
1
1
0
0
0
0
1
1
1
1
0
1
0
0
1
1
1
1
0
0
1
0
0
1
1
0
1
0
1
0
1
0
0
1
0
0
1
1
0

1
0
1
1
1
0
1
0
1
1
1
1
1
1
1
1
0
0
1
0
0
0
1
0
1
1
0
1
1
0
1
1
1
1
1
1
0
1
1
0
1
1
1
1
0
1
1
1
0
0
1
0
1
1

0

0

1

0

0

1

1

0

0

1

0

1

0

0

1

0

0

1

1

0

0

1

0

1

1

0

0

0

1

1

0

0

0

1

1

1

1

1

0

0

1

1

0

0

0

0

0

0

1

1

0

1

1

1

1
1
1
1
1
1
0
1
1
1
0
1
0
0
1
1
0
0
1
0
0
0
0
1
1
1
1
1
0
0
1
0
1
1
0
1
0
0
1
1
0
0
1
1
0
0
1
1
1
1
1
0
1
1

0
0
1
1
0
1
0
0
1
1
1
1
1
1
1
1
0
0
1
1
3
1
1
1
1
1
1
3
3
1
1
1
1
1
0
3
1
3
1
1
1
1
1
0
1
1
0
1
1
0
0
0
1
1

1
3
0
1
1
1
1
1
0
1
1
3
1
1
1
1
1
1
1
1
1
1
1
1
1
1
1
1
1
0
1
1
1
1
1
1
0
1
1
1
1
1
0
1
1
1
1
1
1
0
0
1
1
0

0
0
1
1
1
1
1
0
1
1
1
1
0
1
0
1
1
1
1
1
0
1
1
1
1
0
0
1
1
0
1
1
1
1
1
0
1
0
1
1
1
1
1
1
1
0
1
1
0
1
1
1
1
1

1
1
1
1
1
1
1
1
1
1
0
1
0
1
1
1
1
1
0
0
1
1
1
0
1
1
0
1
1
1
1
1
1
0
1
1
1
1
0
0
1
1
1
1
1
1
1
1
1
1
1
1
1
1

0
1
1
1
1
1
1
1
1
1
1
1
1
1
1
0
1
1
1
1
1
1
1
0
0
1
1
1
1
1
1
1
0
1
1
1
1
1
1
1
1
0
3
1
1
1
1
1
1
1
1
1
1
1

1
1
1
1
1
0
1
0
0
1
0
1
0
1
1
1
1
1
0
1
1
1
1
1
1
1
0
0
1
1
1
1
1
1
1
1
1
1
1
0
0
1
1
1
1
1
0
1
1
1
1
0
0
1

1
1
1
1
0
1
1
1
1
1
1
1
0
1
1
1
1
1
1
1
0
1
1
1
1
3
1
1
1
1
1
1
1
1
1
1
1
1
1
0
1
1
1
1
1
1
1
0
1
1
1
0
1
1

1
1
1
1
1
1
1
0
1
1
1
1
0
0
1
1
1
1
1
0
1
1
0
0
1
1
0
1
0
1
1
1
1
1
1
0
1
1
1
1
3
1
1
1
1
0
1
1
0
1
1
1
1
1

1
1
0
1
1
0
1
1
1
1
1
1
1
1
1
0
1
1
1
1
0
1
1
1
1
1
1
1
1
1
1
1
1
0
1
0
1
1
1
1
1
1
1
1
1
0
1
1
1
1
1
1
1
0

1
1
1
1
1
1
1
1
1
0
1
1
1
0
1
1
1
1
1
3
1
1
1
1
1
1
1
1
1
3
1
1
1
1
0
0
1
1
1
1
1
1
1
1
1
1
1
1
1
1
0
1
1
1

1
1
1
0
1
1
1
1
1
1
1
1
1
0
1
1
1
0
1
1
0
1
1
1
1
0
1
1
1
1
1
1
1
1
1
1
1
1
1
1
1
1
1
1
0
1
0
1
1
1
0
1
1
3

1
1
1
0
1
1
1
1
1
1
1
1
0
1
1
0
0
0
1
3
3
1
0
1
1
1
0
3
1
0
1
1
1
3
0
3
1
1
0
0
0
1
0
0
3
1
0
0
3
0
1
0
3
1

3
3
0
3
0
3
3
3
1
1
1
1
1
1
1
3
0
1
0
3
0
1
1
0
0
0
1
0
1
0
1
0
0
1
0
0
3
1
3
1
0
1
3
1
1
0
0
0
1
3
0
0
0
1

0
0
0
0
0
0
1
3
0
0
0
0
0
0
1
0
0
1
3
1
0
1
0
0
1
1
1
1
1
0
0
1
0
3
0
1
1
0
0
3
0
1
1
1
1
3
1
1
0
1
1
1
3
1

3
3
3
1
0
0
1
0
1
1
0
0
0
0
1
0
1
1
0
0
0
3
1
0
1
1
0
3
3
3
1
1
3
0
0
0
1
3
0
0
3
1
3
1
1
1
0
1
0
1
3
0
1
0

0
1
1
1
3
1
0
3
1
1
1
1
0
1
3
0
3
1
1
0
0
3
